# Towards a DNA Barcode Reference Database for Spiders and Harvestmen of Germany

**DOI:** 10.1371/journal.pone.0162624

**Published:** 2016-09-28

**Authors:** Jonas J. Astrin, Hubert Höfer, Jörg Spelda, Joachim Holstein, Steffen Bayer, Lars Hendrich, Bernhard A. Huber, Karl-Hinrich Kielhorn, Hans-Joachim Krammer, Martin Lemke, Juan Carlos Monje, Jérôme Morinière, Björn Rulik, Malte Petersen, Hannah Janssen, Christoph Muster

**Affiliations:** 1 ZFMK: Zoologisches Forschungsmuseum Alexander Koenig, Bonn, Germany; 2 SMNK: Staatliches Museum für Naturkunde Karlsruhe, Karlsruhe, Germany; 3 ZSM: Zoologische Staatssammlung München, München, Germany; 4 SMNS: Staatliches Museum für Naturkunde Stuttgart, Stuttgart, Germany; 5 Karl-Hinrich Kielhorn, Berlin, Germany; 6 Martin Lemke, Lübeck, Germany; 7 Zoologisches Institut und Museum, Universität Greifswald, Greifswald, Germany; Scientific Research Centre of the Slovenian Academy of Sciences and Art, SLOVENIA

## Abstract

As part of the German Barcode of Life campaign, over 3500 arachnid specimens have been collected and analyzed: ca. 3300 Araneae and 200 Opiliones, belonging to almost 600 species (median: 4 individuals/species). This covers about 60% of the spider fauna and more than 70% of the harvestmen fauna recorded for Germany. The overwhelming majority of species could be readily identified through DNA barcoding: median distances between closest species lay around 9% in spiders and 13% in harvestmen, while in 95% of the cases, intraspecific distances were below 2.5% and 8% respectively, with intraspecific medians at 0.3% and 0.2%. However, almost 20 spider species, most notably in the family Lycosidae, could not be separated through DNA barcoding (although many of them present discrete morphological differences). Conspicuously high interspecific distances were found in even more cases, hinting at cryptic species in some instances. A new program is presented: DiStats calculates the statistics needed to meet DNA barcode release criteria. Furthermore, new generic COI primers useful for a wide range of taxa (also other than arachnids) are introduced.

## Introduction

Long-term monitoring of biodiversity is one of the most important challenges in conservation biology. To evaluate the conservation status and anthropogenic impact of habitats, sufficient knowledge on species composition of natural environments is needed on a regional level. For many if not most invertebrate taxa, we are still far from achieving this goal. One promising approach to meet this challenge is DNA barcoding [1], a technique that uses the easy to homologize, well-quantifiable, discrete taxonomic characters contained in DNA sequence data for standardized, rapid, and relatively cheap species identification. DNA barcoding depends on low levels of intraspecific variation coupled with marked genetic differentiation between species (the 'barcoding gap', investigated in spiders in [2–4]).

With more than 45,800 described species [5], spiders are among the most diverse animal orders [6]. They are abundant in all terrestrial habitats. As ubiquitous predators, they occupy a key position in food webs. Many species show preferences for specific habitat structures or environmental factors, e.g. temperature, humidity, shading [7], which turns them into potential indicators [8]. Easy to observe and document, spiders are seen as a model group for ecological studies [9,10].

The spider fauna of Germany, comprising approximately 1000 species [11], is well known, and checklists and red lists of endangered species have been published for Germany and most of its federal states (see [11] and references therein). The 'Arachnologische Gesellschaft e.V.' (www.arages.de) offers regularly updated occurrence maps, based on a steadily growing database. Therefore, spiders are regularly used in habitat assessments, biodiversity inventories, and ecological studies (e.g. [12–18]). Spiders are particularly promising as indicators of sustainable forest management [19], habitat structure [20], successional stages [21,22], or conservation value [23,24]. There have been several attempts to classify spiders according to their habitat or niche preferences in Germany or Central Europe [7,25–30] and to use these data to classify habitats or assess habitat quality by identifying the proportion of rare, endangered, stenotopic, or character species (e.g. [18,31,32]). Identification of German spiders is facilitated by the online keys for spiders of Europe at www.araneae.unibe.ch [33]. However, morphological identification to species level requires adult specimens in most instances. About 80–200 spider species can occur in a near-natural habitat in Germany, of which only a small fraction can be directly recorded and identified in the field (pers. obs., H. Höfer, C. Muster). For an ecologically meaningful assessment or a close to complete inventory, much more time needs to be invested to capture, process (often meaning dissection of sexual organs) and identify the (adult) spiders, requiring considerable expertise. Regularly, several specimens remain that have to be checked by the few available taxonomic specialists with sufficient knowledge on morphological variability in the respective species and with access to reference collections.

With some 6500 species worldwide, harvestmen (Opiliones) constitute the third-largest order of arachnids [34]. Currently, 52 species have been recorded from Germany [35]. The omnivorous harvestmen constitute a regular component of terrestrial faunas, with highest densities in damp and shaded habitats [36]. Their use in applied and ecological studies is explained by the existence of both stenotopic species with strict microhabitat requirements (and often limited geographic ranges) and invasive species that exhibit immense colonization potential [37,38]. Determination of most German taxa is reliably achievable using the work of [39]. However, recent studies have revealed high levels of cryptic diversity in Central Europe [40–42], suggesting a promising perspective for DNA barcoding in this taxon.

The use of mitochondrial COI barcodes [43] from an extensive reference database of spider and harvestmen species will aid non-specialists in the determination of these groups. Species that have hitherto been problematic or even impossible to identify morphologically–either in general or for a particular sex–may be reliably discriminated. Even though not frequent in Germany, there are still many spider species in which one of the (dimorphic) sexes is still unknown, and barcoding can provide the link between sexes (demonstrated e.g. in [44]). Moreover, disputed instances of synonymy may be resolved [45]. Not least, a considerable advantage of barcoding is the possibility to identify juvenile specimens [3,46–49]. This will not only make inventories more complete, but will also allow species-level inclusion of juveniles into ecological analyses. Tapping into this rich material resource will allow studying more ecological questions without the necessity for exhaustive and expensive sampling. A future broad application of routine DNA barcoding in spiders is facilitated through mass-trapping, since some of the sampling solutions employed in traps preserve DNA well enough for barcoding [50,51]. The method further holds the potential to reveal cryptic species or to identify cases where morphological plasticity may have been over-interpreted. Barcoding may thus act as a catalyst for alpha taxonomy [52]. While introgression events [53], retention of ancestral polymorphisms [49], nuclear mitochondrial pseudo-genes [54] or endosymbionts (*Wolbachia* bacteria etc.) [55,56] all pose potential problems to DNA barcoding approaches, a growing number of studies show the general feasibility of DNA barcoding for arachnids [2–4,46,47,57–66].

The German Barcode of Life (GBOL) campaign is implemented by a national network of ca. 20 biodiversity research institutions and more than 200 taxon specialists [67]. It pursues the goal to establish a DNA barcode library of as many animal, fungal and plant species as possible that occur in Germany. The project aims at collecting, if possible, ten specimens per species, from locations as distinct as possible throughout the country in order to capture genetic variability. Some species with wider ranges may also include specimens collected in neighboring countries.

Natural history collections constitute the core infrastructure of GBOL, taking into account that barcoding projects produce a valuable legacy of vouchers (morphological specimens and molecular samples alike) which become relevant in subsequent studies due to the high quality of the underlying taxonomic assignments and granularity of the metadata. These vouchers form the physical foundation that future monitoring projects will be based on, warranting continuous testability, validation, and coherent expansion of the barcoding reference database–ideally for centuries to come.

Within the GBOL consortium, arachnids have received wide attention, as no less than four GBOL institutes and their respective external arachnologist partners collaborate intensively on compiling a national molecular inventory of spiders and harvestmen: Staatliches Museum für Naturkunde Karlsruhe (SMNK), Staatliches Museum für Naturkunde Stuttgart (SMNS), Zoologisches Forschungsmuseum Alexander Koenig (ZFMK), and Zoologische Staatssammlung München (ZSM). Acari are being investigated in another GBOL subproject (by Senckenberg Museum für Naturkunde Görlitz, SMNG).

For spiders, country-focused, taxonomically broad barcoding datasets have so far been published for Canada [61] (1018 species covered), for Slovenia and for Switzerland [66] (together 298 species) and, in a pilot project, for the Netherlands [68] (31 species) (for a list of ongoing European projects, see http://www.araneae.unibe.ch/barcoding/content/15/Barcoding-of-European-spiders). The present study contributes the first dataset of a spider barcoding campaign for Germany and the first dataset worldwide of this kind for harvestmen.

## Materials and Methods

### Sampling

**Fig 1:**
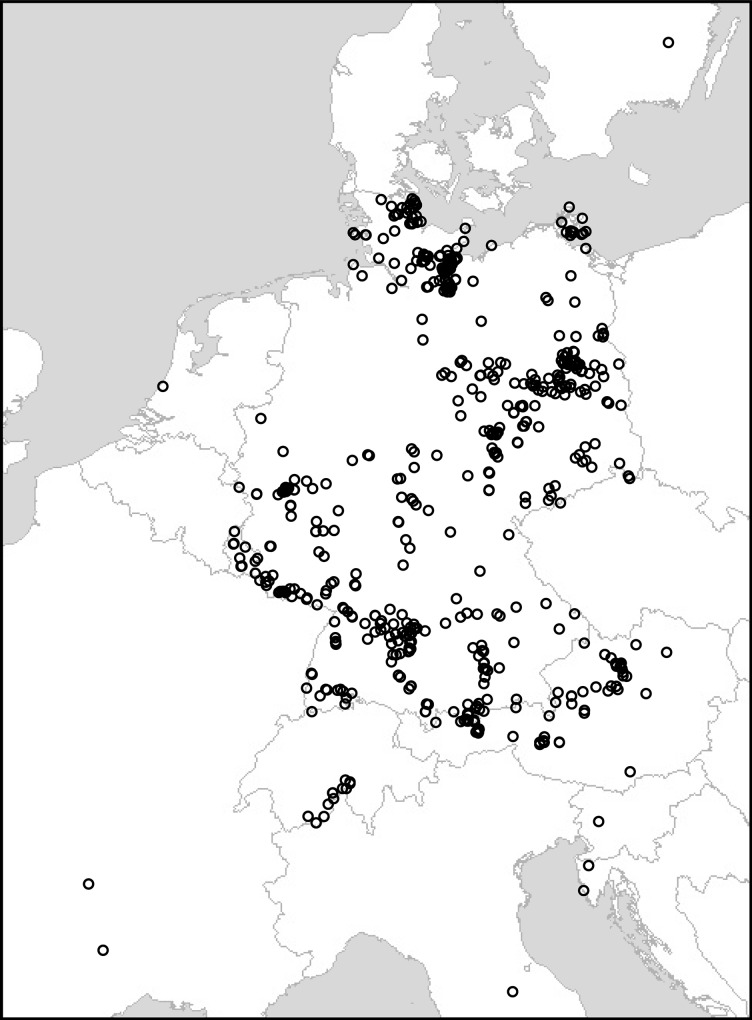
Geographic sampling of arachnid specimens underlying the present study. Image produced using GPS Visualizer (www.gpsvisualizer.com).

For this study, 3537 arachnid specimens, 3339 Araneae and 198 Opiliones, were sampled from Germany (91% of the material) and neighboring countries. Within Germany, 24% of the specimens were collected in Baden-Württemberg and 13% in Schleswig-Holstein. Most other German states were represented by 6–10% of the German specimens each. Thuringia (0.1%), Hesse (1%) and Rhineland-Palatinate (2%) were less well represented, as were the city-states and the comparatively small Saarland (1%). Fig 1 illustrates the sampling pattern.

SMNK and external partners are responsible for and contributed 14% of the specimens, SMNS and partners 10%, ZFMK and partners 56%, ZSM and partners 20%.

**Fig 2:**
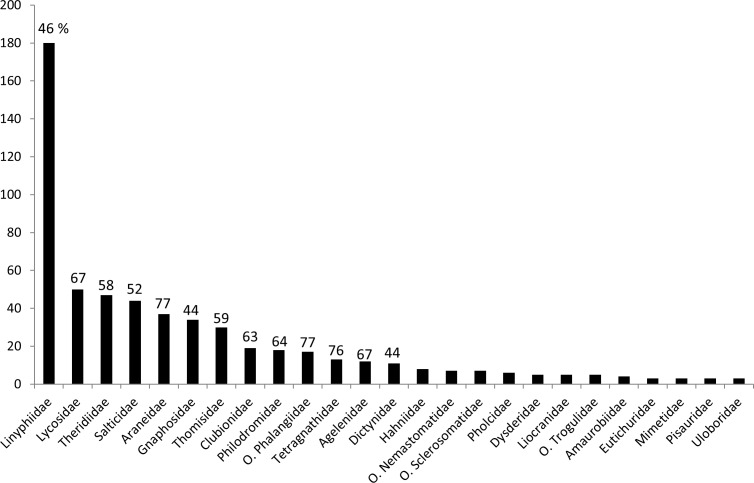
Number of species sampled per family (excluding families represented only by 1 or 2 species in this study). Numbers above bars are percentages showing species coverage for Germany, as derived from the checklists mentioned in the text. Family names prefixed with "O." belong to Opiliones, all others are spider families.

To date 598 morphological arachnid species (561 spp. in spiders *vs*. 37 in harvestmen) in 269 genera (246 *vs*. 23) and 50 families (44 *vs*. 6) could be integrated. Setting this into relation with the German checklists [11,35], species coverage is 57% for spiders and 71% for harvestmen. Species numbers are plotted for the more frequent families in Fig 2.

**Fig 3:**
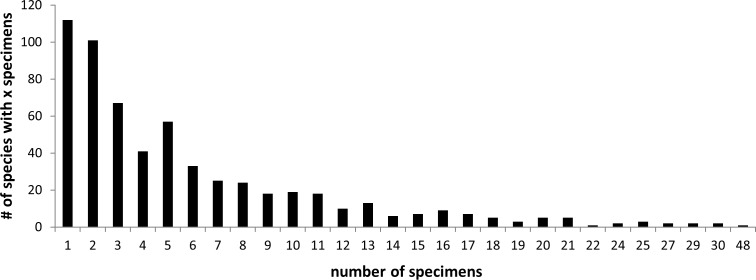
Number of specimens sampled per species. 19% of the species were 'singletons', while the median representation lay at 4 individuals per species.

The species in the dataset were represented by 6 individuals on average (median: 4). Almost 19%, i.e. 112 species, were 'singletons'. With 48 specimens, *Pardosa lugubris* was the species with most individuals; all other species were represented by 30 or fewer individuals (see Fig 3).

Most individuals (98%) were collected specifically for GBOL between the years 2011 and 2015. The oldest specimen processed in this study was collected in 2003.

Collecting was mostly done by hand, and most specimens were killed and preserved directly in 96% or 100% ethanol. 7% of the specimens were initially preserved in 70% water-diluted ethanol and 8% were collected in propylene glycol. The latter was used as capture fluid in pitfall traps; soon after identification, tissue for DNA extraction was transferred to absolute ethanol.

All material used in this study is property of the federal states of the involved institutions. Material acquired by these institutions is only accepted after a check that it was collected in compliance with national and international laws, regulations and conventions and that the material is free from third party rights. Furthermore, in order to become certified as a GBOL collector, it is required to accept the project's general terms and conditions, which demand abiding by the regulations of the Convention on Biological Diversity and national legislations. Field work permits were issued by the following authorities: Bayerisches Staatsministerium für Umwelt und Gesundheit, München; Regierungspräsidium Stuttgart; Regierungspräsidium Karlsruhe; Struktur- und Genehmigungsdirektion Koblenz; Kreisverwaltung Rhein-Sieg-Kreis, Amt für Natur- und Landschaftsschutz; Amt für Umwelt, Verbraucherschutz und Lokale Agenda, Untere Landschaftsbehörde, Bonn; Nationalparkamt Müritz, Hohenzieritz; Biosphärenreservatsverwaltung Niedersächsische Elbtalaue, Hitzacker; Nationalparkforstamt Eifel, Schleiden-Gemünd; Amt für das Biosphärenreservat Südost-Rügen, Putbus; Landesamt für Umwelt, Naturschutz und Geologie Mecklenburg-Vorpommern, Güstrow; Landesamt für Landwirtschaft, Umwelt und ländliche Räume Schleswig-Holstein, Flintbek; Landrat Kreis Herzogtum Lauenburg; Landrat Kreis Rendsburg-Eckernförde, Fachdienste untere Naturschutzbehörde. The permits cover state forests, public land and protected areas as well as the five species of Arachnida protected in Germany: *Arctosa cinerea*, *Dolomedes fimbriatus*, *Dolomedes plantarius*, *Eresus cinnabarinus* and *Philaeus chrysops*.

Altogether, over 100 collectors contributed material. Field data for all analyzed specimens can be accessed in S1 Table. Juvenile specimens analyzed belong to taxa that are easily identifiable also in juvenile stage (e.g. based on coloration) or for which problematic ('look-alike') congeners do not occur in the study area. Juveniles that clustered conspicuously in the tree were removed from the dataset.

All morphological specimen vouchers and also molecular vouchers (DNA and often tissue) are deposited at and are available from the following four German public collections (permanent repositories): Staatliches Museum für Naturkunde Karlsruhe (SMNK), Staatliches Museum für Naturkunde Stuttgart (SMNS), Zoologisches Forschungsmuseum Alexander Koenig (ZFMK), Bonn, Zoologische Staatssammlung München (ZSM). All voucher numbers are given in S1 Table. The voucher IDs in S1 Table as well as the names in the trees include the institutional code, so that the association of a given sample to one of the GBOL partner institutes can be easily established.

Sequence data are available on BOLD [69] via DOI dx.doi.org/10.5883/DS-GBOLARA, and on GenBank. Specimen data will also be accessible, alongside specimen images, through the GBOL portal (www.bolgermany.de).

### Molecular methods

Analyses were performed mostly in three separate laboratories: ZFMK, SMNS, and Canadian Centre for DNA Barcoding (CCDB) in Guelph. SMNS and ZFMK used their own facilities (up to the point of sequencing), SMNK samples were processed at both SMNS and ZFMK, ZSM samples at CCDB.

Total genomic DNA was usually isolated from legs. In very small specimens (especially many Linyphiidae) at ZFMK and SMNS, DNA was extracted non-destructively from whole specimens which were recovered after lysis (cf. [70]).

At ZSM, single legs were removed from each specimen and sent in 96 well lysis plates to CCDB for standardized DNA extraction, PCR amplification and bidirectional Sanger sequencing. CCDB lab protocols are available under www.ccdb.ca/resources.php.

At ZFMK and SMNS, silica-based methods were employed to extract DNA. SMNS followed the protocol by [71] with Pall AcroPrepTM 96 filter plates (Pall Corporation, Port Washington, NY, USA), while a Qiagen (Hilden, Germany) BioSprint96 magnetic bead extractor and corresponding kits were used at ZFMK.

Polymerase chain reaction for the 5' part of the mitochondrial cytochrome *c* oxidase subunit 1 (COI) gene was carried out, at ZFMK, in total reaction mixes of 20 μl, including 2 μl of undiluted DNA template, 0.8 μl of each primer (10 pmol/μl), and standard amounts of the reagents provided with the 'Multiplex PCR' kit from Qiagen (Hilden, Germany). At SMNS, PCR reactions of 25 μl volume contained 4 μl DNA, 5 units of Taq KAPA extra polymerase (KAPABIOYSYTEMS, Boston, USA), 10 μl of 5x KAPA Taq extra buffer, 3 μl of MgCl_2_ (25 mM; both solutions provided by the manufacturer), 1 μl of each primer (10 pmol/μl), and 1 μl of 10 mM dNTP mix (Bioline, Luckenwalde, Germany).

The PCR primers used (also for sequencing) are given in Table 1.

**Table 1:** List of primers used for amplification and sequencing of the 5' part of the mitochondrial COI gene.

Primer name	Sequence	Publication	Used at
LCO1490	5'-GGTCAACAAATCATAAAGATATTGG	Folmer et al. 1994	SMNS, CCDB for ZSM, ZFMK
HCO2198	5'-TAAACTTCAGGGTGACCAAAAAATCA	Folmer et al. 1994	SMNS, CCDB for ZSM, ZFMK
LepF1	5'-ATTCAACCAATCATAAAGATATTGG	Hebert et al. 2004	CCDB for ZSM
LepR1	5'-TAAACTTCTGGATGTCCAAAAAATCA	Hebert et al. 2004	CCDB for ZSM
C_LepFolF	cocktail of LepF1 and LCO1490	www.boldystem.org/index.php/Public_Primer_PrimerSearch	CCDB for ZSM
C_LepFolR	cocktail of LepR1 and HCO2198	www.boldystem.org/index.php/Public_Primer_PrimerSearch	CCDB for ZSM
LCO1490-JJ	5'-CHACWAAYCATAAAGATATYGG	Astrin & Stüben 2008	ZFMK
HCO2198-JJ	5'-AWACTTCVGGRTGVCCAAARAATCA	Astrin & Stüben 2008	ZFMK
LCO1490-JJ2	5'-CHACWAAYCAYAARGAYATYGG	new	ZFMK
HCO2198-JJ2	5'-ANACTTCNGGRTGNCCAAARAATCA	new	ZFMK
LCO1490-JJ4a	5'-CNACNAAYCAYARRGAYATYGG	new	ZFMK
HCO2198-JJ4a	5'-AIACYTCNGGRTGICCAAARAATC	new	ZFMK
LCO1490-JJ4	5'-CIACIAAYCAYAARGAYATYGG	new	ZFMK
HCO2198-JJ4	5'-ANACTTCNGGRTGNCCAAARAATC	new	ZFMK

Species—also many others than arachnids—with strongly modified binding sites could usually be successfully amplified at ZFMK with a set of newly (manually) designed, highly degenerate primers (most often using combination LCO1490-JJ2 & HCO2198-JJ2). The combination LCO1490-JJ and HCO2198-JJ constitutes the standard set of primers used at ZFMK. The standard set of primers used at CCDB for ZSM was the combination C_LepFolF and C_LepFolR, a cocktail consisting of the primers listed above.

Thermal cycling was performed, at ZFMK, on Applied Biosystems 2720 Thermal Cyclers (Life Technologies, Carlsbad, CA, USA), using a PCR program with two cycle sets, as a combination of a 'touchdown' and a 'step-up' routine: first cycle set (15 repeats): 35 s denaturation at 94°C, 90 s annealing at 55°C (−1°C per cycle) and 90 s extension at 72°C. Second cycle set (25 repeats): 35 s denaturation at 94°C, 90 s annealing at 45°C, and 90 s extension at 72°C. At SMNS, PCR amplification was carried out in a Labcycler by SensoQuest (Göttingen, Germany). PCR conditions were: 35 cycles of 60 s denaturation at 93°C, 90 s annealing at 50°C and 60 s extension at 72°C.

PCR products were subsequently sent for bidirectional Sanger sequencing to various companies: ZFMK to BGI (Hong Kong, China) and Macrogen (Amsterdam, Netherlands), SMNS to LGC Genomics (Berlin, Germany) in 2013 and from 2014 on to GATC Biotech (Konstanz, Germany).

DNA sequence alignment was performed using parallelized MAFFT ver. 7.123 [72]. PAUP* ver. 4.0b10 [73] was used for p-distance transformations and for evaluating base composition and information content. Statistical parsimony networks [74] were calculated with the TCS algorithm [75] in PopART (http://popart.otago.ac.nz). Statistical evaluation of the data was performed using SPSS, R (box plots), Species Identifier ver. 1.7.7–3 [76] (extraction of 'splits' and 'lumps'), and the Perl script DiStats (intraspecific distances, individualized data on closest species pairs and on most distant congeners). DiStats has been developed for this study and is available, including documentation, under GitHub (https://github.com/mptrsen/distats) and through the ZFMK homepage (www.zfmk.de/en/research/research-centres-and-groups/distats). The script uses FASTA as input format, calculates *p*-distances or K2P distances and can be parallelized in order to process large datasets. It can produce two output files: a table with statistics for each species and optionally also the matrix of all pairwise distances in the dataset. For an alignment containing 1000 COI barcode sequences, the analysis will take around 6 minutes when using a single thread (on a 3.4 GHz processor). DiStats has an algorithmic complexity (*O*) of approximately *O*(n^2^), which means that run time increases exponentially with the number of input sequences (*n*). Using multiple CPU threads reduces the run time by a factor of 1/*c*, where *c* is the number of threads.

PAUP was also used for reconstruction of a phenetic neighbor-joining (NJ; [77]) tree as a quick molecular identification check. Phylogenetic reconstructions using Maximum Likelihood (ML; [78]) were performed with RAxML ver. 7.3.0 [79]. Evolutionary model selection for the ML analysis was implemented, using hierarchical likelihood ratio testing, in ModelGenerator ver. 0.85 [80] and indicated GTR + I + Γ as the best-fitting model [81]. The COI dataset was partitioned to treat 3rd codon positions separately from 1st and 2nd positions. The analysis was run for 1 million generations and included 1000 bootstrap replicates. For tree rooting purposes in NJ and ML analyses, we chose a mite sequence from BOLD as outgroup (see S1 Alignment).

## Results

Average COI sequence length for the 3537 sequences was 650 bp. To accommodate many slightly shorter sequences, while avoiding genetic distance artifacts, alignment length was set to 653 bp. Sequences shorter than 500 bp were excluded from the analysis. The shortest included sequence was composed of 509 residues.

The dataset comprised 2099 distinct haplotypes, meaning that 1438 sequences were non-unique.

Among nucleotides, there was a compositional bias towards AT: 67.5%, which is close to levels previously reported for spiders (e.g. [4,82,83]). In detail, overall base composition was: A 25.3, C 13.3, G 19.2, T 42.2%.

Altogether, 5,572,791 pairwise distances were computed for spiders; of these, 17,867 were intraspecific distances. For Opiliones, there were 19,503 pairwise distances of which 896 were distances between conspecific specimens. Table 2 gives an overview of intra- and interspecific distances, separated by order. Table 3 summarizes distances for all closest species pairs and for the most distant congeneric pairs; S2 and S3 Tables illustrate these in more detail, giving individual statistics by taxon. S2 Table (spiders) and S3 Table (harvestmen) furthermore indicate intraspecific distance ranges and central tendencies for all analyzed species. S4 Table individually lists the highest intraspecific distances in the dataset, while S5 Table gives the lowest interspecific distances.

**Table 2:** Estimators used to characterize genetic distance structure in the dataset.

[%]	intraspecific				interspecific			
	median	mean	range	95^th^ perc.	median	mean	range	5^th^ perc.
Araneae	0.3	0.7	0.0–10.1	2.5	17.5	17.4	0* - 28.2	13.6
Opiliones	0.2	1.3	0.0–8.9	8.1	19.3	19.4	7.0–30.1	13.6
Aran. K2P	0.3	0.7	0.0–11	2.6	20.0	20.0	0.0–35.6	15.0
Opil. K2P	0.2	1.3	0.0–9.5	8.7	22.6	22.7	7.5–38.6	15.1

The upper two rows indicate uncorrected distances for spiders and harvestmen, respectively, while the third and fourth rows give K2P distances (as required for a barcode data release). Median and mean distances are given for both intraspecific and interspecific distances, along with the range between the smallest and largest observation in the respective data category. *: There were cases of shared haplotypes among species, see text.

**Table 3:** Statistics for closest species pairs and most distant congeneric pairs.

[%]	closest species pairs		most distant congener pairs	
	range	median (of all dist. for species pairs)	range	median (of largest distances)
Araneae	0.0* - 20.1 (22.0)	9.2	1.8–20.2	11.8
Opiliones	7.0–21.8 (22.8)	13.8	12.6–18.8	14.9
Araneae K2P	0.0–21.0 (28.6)	9.9	1.9–23.8	12.8
Opiliones K2P	7.5–24.0 (29.0)	15.5	13.7–22.0	16.5

The upper two rows indicate uncorrected distances for spiders and harvestmen, respectively, while the third and fourth rows give K2P distances (as required for a barcode data release). The range for the closest species pairs indicates the minimum and maximum among all *smallest* pairwise distances between closest species pairs. If a closest species pair is represented by several individuals, there may be larger distances as well: the maximal closest species distance in the respective dataset is indicated in parentheses. While for the closest species pairs no classificatorial (genus) background information was used, the last two columns in this table orient themselves at distances from representatives within the *same genus* (but different species). The range for the most distant congener pairs extracts the extremes among these *maximal* pairwise distances between farthest congeneric species pairs. Information on the individual closest species pairs and on the respective most distant congeneric species pairs is given in S2 Table for spiders and S3 Table for harvestmen.

* There were cases of shared haplotypes among species, see text.

The range covered by intraspecific (*p*-)distances was similar for spiders (0–10%) and harvestmen (0–9%), with an arithmetic mean of 0.7% in spiders and 1.3% in harvestmen. The influence of high outliers was stronger in the comparatively small harvestman dataset (median at 0.2% vs. mean at 1.3%), caused mostly by the deep splits within *Mitopus morio* (Fabricius, 1779) and *Phalangium opilio* Linnaeus, 1758 (both discussed below), but also in *Nemastoma lugubre* (Müller, 1776) (see S4 Table).

The interspecific distance range varied for the two arachnid orders: 0.0–28% in spiders, 7–30% in harvestmen. In four cases, haplotypes were shared among nominal spider species (see S5 Table, all discussed below): *Enoplognatha latimana* Hippa & Oksala, 1982 */ E*. *ovata* (Clerck, 1757)*; Pardosa lugubris* (Walckenaer, 1802) */ P*. *saltans* Töpfer-Hofmann, 2000*; Tibellus maritimus* (Menge, 1875) */ T*. *oblongus* (Walckenaer, 1802)*; Xysticus audax* (Schrank, 1803) */ X*. *cristatus* (Clerck, 1757). Interspecific arithmetic means were 17% for spiders, 19% for harvestmen.

Mean intraspecific distances varied considerably among families. For the best-represented spider families, these lay between 0.4% (Agelenidae, Lycosidae, Philodromidae) and 1.0% (Tetragnathidae). Clubionidae, Linyphiidae and Theridiidae had mean intraspecific distances of 0.6%, Gnaphosidae 0.7%, Araneidae 0.8%, Salticidae and Thomisidae 0.9%.

**Fig 4:**
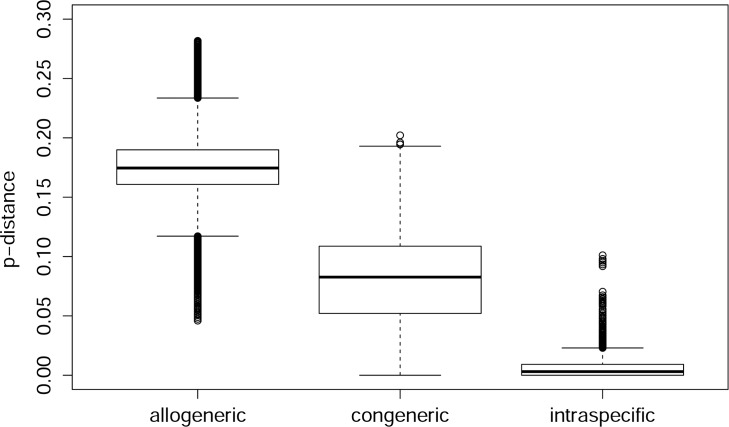
Box plot of *p*-distances for the order Araneae. Sorted by distance category: between specimens of different genera (allogeneric), between specimens belonging to different species, but to the same genus (congeneric), and between specimens that belong to the same species (intraspecific). Boxes indicate interquartile range (IQR: between upper [Q3] and lower [Q1] quartile). Black bars designate medians, whiskers indicate values within 1.5 × IQR beneath Q1 or 1.5 × above Q3. Circles depict outliers (above or below 1.5 × IQR).

**Fig 5:**
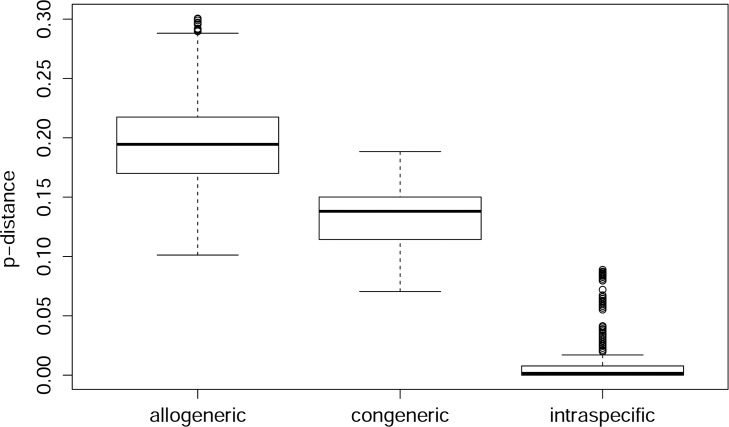
Box plot of *p*-distances for the order Opiliones. Legend: see Fig 4.

Table 2 and the box plots (Figs 4 and 5) indicate that a universal barcoding gap is absent from the dataset. However, most of the species separate well; when ignoring the 5% most extreme outliers (a hypothetical scenario not surpassing the usual significance threshold), the barcoding gap for harvestmen would span 5.5% and for spiders even 11% (see Table 2). The median distance for closest species pairs was 9% in spiders and 13% in harvestmen.

A phenetic reconstruction using Neighbor Joining (NJ; S1 Fig) and a phylogenetic reconstruction using Maximum Likelihood (ML; S2 Fig) delivered trees in which the species overwhelmingly formed monophyletic clusters (but see discussion –several of the cases with conspicuous distances were also recovered as paraphyletic and polyphyletic). ML bootstrap analysis predominantly indicated very high support for species-level nodes, but did usually not allow much insight into deeper tree topology.

## Discussion

Many previous studies using COI have shown that species differentiation via DNA barcoding is generally feasible and promising in arachnids (mostly spiders: e.g. [2–4,46,47,57–66]–but see e.g. [84–87]). This also applies to the present dataset. For most analyzed species, a so-called 'barcoding gap' exists: intraspecific sequence divergence levels are clearly lower than interspecific divergence to the nearest neighbor taxon in the dataset. This general tendency becomes evident from Table 2 and Table 3: the medians (and also the arithmetic means) of all distances between closest species lie around 9% in spiders and 13% in harvestmen, while in 95% of the cases, intraspecific distances are below 2.5% and 8%, with intraspecific medians at 0.7% and 0.2%.

However, despite the overall high suitability for barcoding of the dataset, we also encountered 19 currently valid species (3% of the dataset, all of them spiders) that are neither recovered monophyletic in the trees, nor in which the maximum intraspecific distance exceeds the distance to the nearest neighbor. Species determination via DNA barcoding fails in these instances. Since many if not most of the involved species pairs show discrete morphological differences, the explanation of such discrepancies between morphology and molecules should be regarded as a chance rather than a nuisance: it demands differentiated evolutionary hypotheses and directs further in-depth study that may result in intriguing biological insights [88].

**Fig 6:**
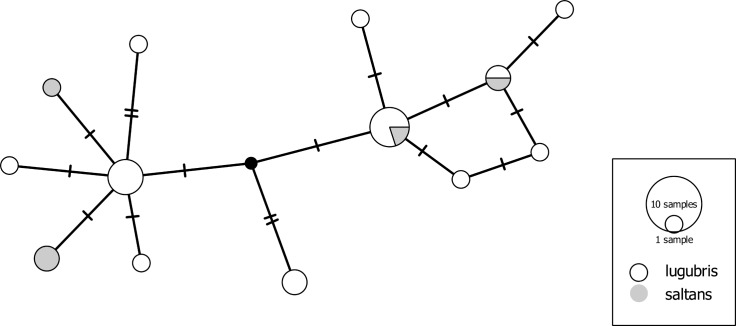
Haplotype network of the species pair *Pardosa lugubris*/*saltans*. To guarantee unequivocal morphological determination, only males were included. Small black dot indicates a hypothetical haplotype.

**Fig 7:**
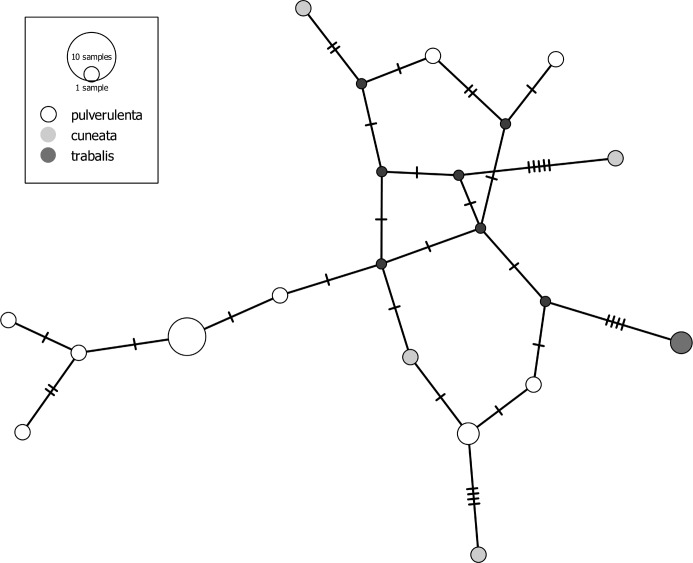
Haplotype network of three species of the *Alopecosa pulverulenta* group. To guarantee unequivocal morphological determination, only males were included (except for one female of *A*. *trabalis*). Small black dots indicate hypothetical haplotypes.

Overall, the dataset contains 26 species with *p*-distances to the nearest interspecific neighbors below 2%. The most striking examples for difficult taxon separation from the GBOL dataset concern wolf spiders (Lycosidae). Wolf spiders alone contribute half of the 'barcode-resistant' cases mentioned above. The species pair *Pardosa lugubris/saltans*, for example, shows a pattern of completely intermixed haplotypes (Fig 6). It has been noted previously that "individuals of the *P*. *lugubris* group [containing additional species, e.g. *P*. *alacris*] cannot be identified by DNA barcoding, nor by ITS2 and 28S" [89]. The species in this complex are arguably well isolated by courtship behavior, while females, in particular, pose challenges also to morphological identification [90] (the latter were identified based mostly on [91]). To our surprise, a similar pattern of nonexistent haplotype segregation was detected in *Alopecosa cuneata* (Clerck, 1757) and *Alopecosa pulverulenta* (Clerck, 1757) (Fig 7). These are two of the most abundant spider species in Central European grassland ecosystems. Males are readily distinguished, even in the field, by the distinctive swelling of the front tibiae in *A*. *cuneata*. Furthermore, they show differences in details of the sexual organs and in courtship behavior [92]. Further examples of very low COI differentiation in Lycosidae include species within the *Pardosa pullata* group (S3 Fig) and several species pairs in the *P*. *monticola* group (*P*. *agrestis* (Westring, 1861) */ P*. *torrentum* Simon, 1876*; P*. *agrestis / P*. *palustris* (Linnaeus, 1758)*; P*. *agrestis / P*. *monticola* (Clerck, 1757)*; P*. *palustris / P*. *torrentum*). We speculate that the shallow mitochondrial divergence in many of the analyzed Lycosidae (but see [2]) may be related to the complex courtship behavior of these spiders [93]. A plausible mechanism is accelerated speciation through sexual selection. This could lead to fixation rates in male behavioral traits that exceed those of (putatively) neutral mitochondrial genes, as demonstrated for some jumping spiders (Salticidae) by [94]. These findings offer a promising perspective for detailed evolutionary and ethological studies.

In families other than wolf spiders, we encountered considerably fewer cases without barcoding gaps. In crab spiders (Thomisidae), the species pair *Xysticus audax/cristatus* is notorious for the difficult separation of females, while the male palps are clearly distinct [95]. In our data, the haplotypes of *X*. *audax* and *X*. *cristatus* are intermingled, while they are separated from the related species *X*. *gallicus* Simon, 1875 and *X*. *kochi* Thorell, 1872 (S4 Fig). Equally, *Tibellus maritimus* and *T*. *oblongus* (Philodromidae) are not separable by their COI sequences (S5 Fig), although morphological discrimination is rather straightforward. This result is supported by Canadian specimens as well [61]. In the comb-footed spiders (Theridiidae) we encountered two examples of very limited or even absent COI differentiation. *Enoplognatha latimana* and *E*. *ovata* were not separated until 1982 [96]. Both species are widespread and abundant vegetation dwellers in Central Europe, often occurring syntopically. Although differences in the male palps are distinct and constant, the COI haplotypes are not segregated. An ongoing study at the University of Bern with more comprehensive sampling and comprising additional molecular markers questions the taxonomic status of these two nominal species [89]. Strikingly similar species are *Parasteatoda tepidariorum* (C. L. Koch, 1841) and *P*. *simulans* (Thorell, 1875). In the tree, the only sequence of *P*. *simulans* is nested within the relatively homogenous clade of *P*. *tepidariorum*. The only more or less solid morphological differences between these two species are the size dimensions (*P*. *tepidariorum* being significantly larger than *P*. *simulans*). Several authors have doubted the species status of *P*. *simulans* or treated it as subspecies of *P*. *tepidariorum* (e.g. [97–99]). In Germany, *P*. *tepidariorum* is usually found in buildings, while *P*. *simulans* also occurs outside, e.g. on the bark of trees. It is well conceivable that specimens living in marginal habitats stay smaller and develop a slightly different coloration pattern, representing ecological morphs within a species.

Linyphiidae, the most speciose spider family in Central Europe, contains a few examples of conspicuously shallow COI divergences among congeneric species (although most do not infringe a barcoding gap). These include *Agyneta ressli* (Wunderlich, 1973) */ A*. *rurestris* (C. L. Koch, 1836)*; Gongylidiellum murcidum* Simon, 1884 */ G*. *vivum* (O. Pickard-Cambridge, 1875)*; Hypomma bituberculatum* (Wider, 1834) */ H*. *cornutum* (Blackwall, 1833) */ H*. *fulvum* (Bösenberg, 1902); and *Tapinocyba affinis* Lessert, 1907 */ T*. *pallens* (O. Pickard-Cambridge, 1872). In all these instances, the species are distinguishable by consistent differences in at least the male sexual organs, even though distinction is subtle in some cases.

The processes behind the incomplete mitochondrial segregation in species of the latter families are possibly different from wolf spiders, which have a complex visual and acoustic courtship behavior. Alternative evolutionary explanations include the existence of distinct morphs within polymorphic species (e.g. [100,101]) or mitochondrial introgression, which has so far rarely been reported from spiders [102,103]. Detailed studies are required for each individual taxon to uncover the underlying mechanisms.

In recent years, great attention has been paid to the detection of cryptic diversity as reflected in deep intraspecific splits. Many new species have been described based on deep COI divergence within morphologically similar taxa (e.g. [104–109]). The GBOL dataset contains 48 species with a maximum intraspecific barcode divergence of > 3% (26 species when looking at a maximal intraspecific distance of > 4%). Interestingly, the proportion of species with conspicuously large intraspecific variation is considerably higher in Opiliones than in Araneae: 27% in harvestmen versus 8% in spiders (or 15% vs. 4% when using 4% as cutoff). This finding suggests that more cryptic diversity is to be expected in harvestmen than in spiders, a result that may be related to the comparatively reduced character complexity in the sexual organs of harvestmen.

A frequently observed pattern in our data is a single outlier haplotype found alongside a cluster of closely related sequences (e.g. in *Aelurillus v-insignitus* (Clerck, 1757); *Nemastoma lugubre* (Müller, 1776); *Steatoda bipunctata* (Linnaeus, 1758); *Clubiona corticalis* (Walckenaer, 1802); *Hypsosinga albovittata* (Westring, 1851); *Pardosa hortensis* (Thorell, 1872); *Centromerus pabulator* (O. Pickard-Cambridge, 1875); *Tetragnatha obtusa* C. L. Koch, 1837; *Steatoda albomaculata* (De Geer, 1778); *Robertus lividus* (Blackwall, 1836); *Xysticus lanio* C. L. Koch, 1835; in order of descending divergence). In the case of the largest intraspecific barcode divergence, *Aelurillus v-insignitus* (maximum *p*-distance 10.1%), we can trace back the deep split to differences between specimens of the gray and black morphs, which are well distinguished morphologically [110] and may represent separate species. Likewise, the split within *Steatoda bipunctata* (maximum *p*-distance 7%) is corroborated by external evidence, as our outlier specimen from Berlin shares an identical barcode with two specimens from Canada/Nova Scotia (submitted to BOLD by G. Blagoev and colleagues), hinting at a so far unrecognized sibling species.

In other cases of single outlier sequences we refrain from further interpretation. Although we took greatest care in the detection of numts (nuclear mitochondrial DNA) [111] and processing errors, sequencing artefacts cannot be completely ruled out.

**Fig 8:**
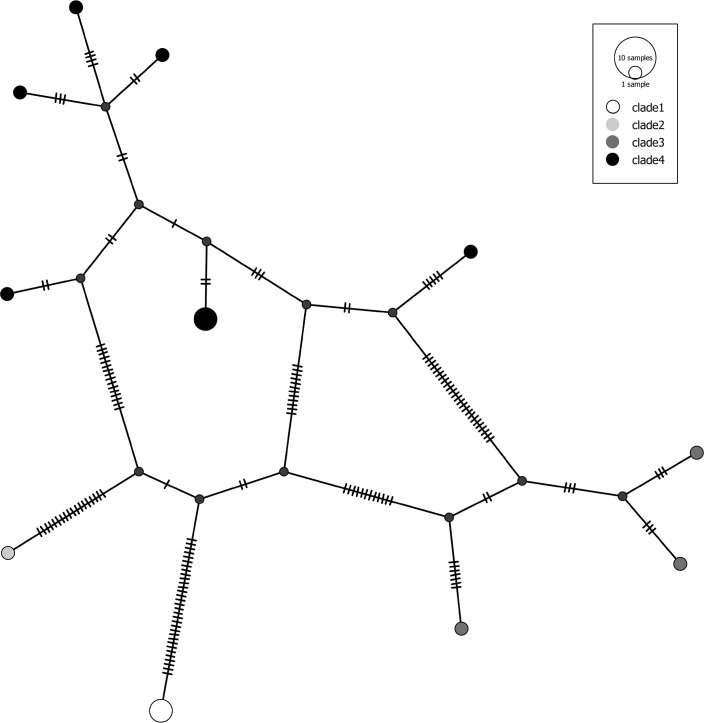
Haplotype network of *Mitopus morio*.

Nonetheless, numerous examples remain of currently valid species where (multiple) sequences fall into two or more clearly distinct COI clusters. A representative case is the opilionid *Mitopus morio* (Fabricius, 1779), the harvestman species with the widest geographic distribution and the highest abundance in European mountain ecosystems. The taxon shows a remarkable altitudinal variation in leg length and dorsal coloration pattern. [41] investigated the genetic structure along two altitudinal transects in the Alps and found three deeply diverged lineages which, however, did not correspond to leg morphometric variants. The 15 GBOL sequences of *Mitopus morio* fell into four deeply diverged clades (Fig 8 and S1 Fig). Sequences of clade 1 originate from the German Alps and the Bavarian Forest, the single specimen of clade 2 comes from the surroundings of Berlin, clade 3 is restricted to the Alps (Karwendel and Wallis), while clade 4 appears widespread in Central Europe. Thus, specimens of three clades occurr in the Alps and may well correspond to the lineages described by [41]. However, the true diversity in Central Europe may be even higher and available names currently in synonymy of *M*. *morio* may deserve revalidation (e.g. *Mitopus ericaeus* Jennings, 1962 from Great Britain). A similar situation applies to the common harvestman *Phalangium opilio* Linnaeus, 1758. This species is known for extreme variation in body size and in length of the conspicuous process on the second cheliceral segment, but due to the apparently continuous variation, all morphological variants have been considered conspecific [39]. The 12 GBOL sequences of *Phalangium opilio* split into two clades that are separated by *p*-distances of 8.3–8.7% and show a sympatric distribution in Germany.

Also for several spider species, sequences fall into two deeply diverged clusters: *Tmarus piger* (Walckenaer, 1802); *Micaria pulicaria* (Sundevall, 1831); *Nigma walckenaeri* (Roewer, 1951); *Sitticus pubescens* (Fabricius, 1775); *Hahnia nava* (Blackwall, 1841); *Euophrys frontalis* (Walckenaer, 1802); *Salticus scenicus* (Clerck, 1757); *Xysticus kochi* Thorell, 1872; *Theridion familiare* O. Pickard-Cambridge, 1871; *Haplodrassus dalmatensis* (L. Koch, 1866); *Heliophanus flavipes* (Hahn, 1832), or even into multiple clusters: *Tetragnatha extensa* (Linnaeus, 1758), *Drassodes lapidosus* (Walckenaer, 1802), *Neon reticulatus* (Blackwall, 1853), *Haplodrassus signifer* (C. L. Koch, 1839) (ordered by descending genetic divergence, see S5 Table for the respective distance values). All these species show pairwise intraspecific distances between 3% and 7%. One plausible explanation for comparably high intraspecific divergence is isolation by distance in dispersal-limited species. We did not find indications for allopatric distribution of clusters in any of these species, but the limited size of the datasets in most species precludes more detailed analyses at this stage. Without doubt, all these taxa deserve a thorough taxonomic reconsideration. The GBOL dataset can be a convenient starting point to that end; it offers useful guidance for taxonomists to select promising study objects.

Finally, the GBOL data provide five new records for Germany: one on national scale, and seven at federal state level. The species *Sibianor larae* Logunov, 2001 and *Evansia merens* O. Pickard-Cambridge, 1900 have been recorded for the first time in Baden-Württemberg, in mountainous, relatively humid heathland in the Black Forest. *S*. *larae* has been recorded in the same type of open country habitat in the Netherlands [112]. *Oreonetides glacialis* (L. Koch, 1872) could be recorded for the first time in Bavaria (arguably also for the whole country). It was collected–in both sexes–as the dominant spider species on a barren karst plateau on the Zugspitze (at 2600 m.a.s.l.; leg. J. Spelda, S. Friedrich & R. Melzer) among scree, the typical habitat for this species. The crab spider *Xysticus acerbus* Thorell, 1872 is the first record for Mecklenburg-Vorpommern (leg. C. Muster). Finally, for Schleswig-Holstein, *Hahnia ononidum* Simon, 1875, *Mermessus trilobatus* (Emerton, 1882), *Glyphesis servulus* (Simon, 1881) (leg. M. Lemke) all represent new records.

*Pholcus* Walckenaer, 1805 is the most species-rich genus in Pholcidae, with most of the currently 329 species in tropical and subtropical regions (www.pholcidae.de). Only two widespread species have previously been recorded in Germany: the cosmopolitan synanthropic *Pholcus phalangioides* (Fuesslin, 1775) and the Mediterranean to Central Asian anthropophilic *P*. *opilionoides* (Schrank, 1781). Several further representatives of the genus occur in and around human buildings and have probably for this reason attained wide distributional ranges [113]. Among them is the East European to Central Asian *P*. *alticeps* Spassky, 1932, whose most western record so far was from Poland [113]. Our sequenced specimen originates from eastern Germany (Sachsen, Dresden-Kaditz). At the same locality, a vital population of *P*. *alticeps* (adult males and females as well as juveniles) was observed in June 2015 (leg. C. Muster), co-existing with *P*. *phalangioides*. Specimens were collected from a cellar, a barn, and outdoors from the wall of a building. Thus, the species is probably well established at this locality.

## Conclusion

For ca. 60% of the German spider fauna and ca. 70% of the country's harvestman fauna, the dataset and material basis provided through this study enable fast, reliable and reproducible species identification via barcoding and highlight the species with inherent problems connected to this type of identification or to current taxonomy.

Building extensive, carefully validated reference databases ('libraries') is the most relevant prerequisite for successful DNA barcoding applications. In this context, our project should considerably facilitate DNA-based species identification of Araneae and Opiliones in Germany, for non-specialists as well as for large-scale biodiversity monitoring endeavors. The latter is envisioned in a campaign currently proposed for Germany and is already implemented (on a much smaller scale) in the second phase of the German Barcode of Life Project. Within this project, compiling the reference database and reference collections is still an ongoing effort, for arachnids as well as for many other taxa.

## Supporting Information

S1 Alignment Sequence data for the 3538 analyzed arachnid specimens. Includes the mite outgroup retrieved from BOLD: BOLDMSACA57112_OG_Acari. FASTA-formatted. See S1 Table for more details on specimens.
(FAS)

S1 Fig Neighbor Joining tree. PDF can be searched for species names. Apart from ID and species name, life stage, sex and coordinates of collecting locality are given. See S1 Table for more details on individual specimens in the tree.
(PDF)

S2 Fig Maximum Likelihood tree with bootstrap values. The analysis was run for 1 million generations and includes 1000 bootstrap replicates. Apart from ID and species name, life stage, sex and coordinates of collecting locality are given. See S1 Table for more details on individual specimens in the tree.
(PDF)

S3 Fig Haplotype network of three species of the *Pardosa pullata* group. Small black dots indicate hypothetical haplotypes.
(TIF)

S4 Fig Haplotype network of the species complex *Xysticus audax* and *X*. *cristatus*, along with their closest relatives. To guarantee unequivocal morphological determination, only males were included. Small black dots indicate hypothetical haplotypes.
(TIF)

S5 Fig Haplotype network of the species pair *Tibellus maritimus* and *T*. *oblongus*. Small black dots indicate hypothetical haplotypes.
(TIF)

S1 Table Field data and IDs for all analyzed specimens. This table lists collecting date and location (incl. GPS coordinates), collector, taxonomy, identifier, preservation fluid, life stage and sex for the specimens analyzed. Sample IDs in this table correspond to those given in S1 Fig (NJ tree) and S2 Fig (ML tree), as well as in S4 Table ('splits') and S5 Table ('lumps'). Please note that while working on the release dataset, some species names have changed: *Dictyna civica* -> *Brigittea civica*, *Dictyna latens* -> *Brigittea latens*, *Hahnia difficilis* -> *Iberina difficilis*, *Hahnia montana* -> *Iberina montana*, *Lepthyphantes keyserlingi* -> *Ipa keyserlingi*, *Titanoeca psammophila* -> *Titanoeca spominima*. For these species, the old names are used throughout the article and related materials.
(XLSX)

S2 Table List of closest species pairs and most distant congeneric species pairs for spiders. Statistics, individual by species, for three types of distance comparisons (intraspecific, closest interspecific, largest congeneric): minimal, maximal, mean and median *intraspecific* genetic distances; *closest species* (by distance) and minimal, maximal and median distance separating the two species; genetically most distant species within the same genus along with maximal distance in separating the two species. In case of identical distances to reference species, two or more rows under the same species name are used for listing all these cases–one line for each allogeneric comparison (note: the DiStats script used to compute these values considers the full number of decimal places during comparison/sorting of distances, even if output is set to contain only 2 decimal places, as in DiStats default mode).
(XLSX)

S3 Table List of closest species pairs and most distant congeneric species pairs for harvestmen. Legend: see caption for S2 Table.
(XLSX)

S4 Table Highest intraspecific distances, 'splits'. This table contains the 352 pairwise comparisons with the highest conspecific *p*-distances in the dataset, ranging from 10 to 3% (range 5 to 3% given in gray, denoting an 'uncertainty zone' for average species limits in this scenario). 164 comparisons have values above 4%. Specimens are identified through species name and ID (see S1 Table for more details).
(XLSX)

S5 Table Lowest interspecific distances, 'lumps'. This table contains the 731 pairwise comparisons with the lowest allospecific (but congeneric) *p*-distances in the dataset, ranging from 0 to 5%. 353 comparisons have values below 3%. Specimens are identified through species name and ID (see S1 Table for more details).
(XLSX)
