# Supplementary material for: Towards a DNA Barcode Reference Database for Spiders and Harvestmen of Germany
Source: PLoS One. 2016 Sep 28;11(9):e0162624. doi: 10.1371/journal.pone.0162624 (PMC5040438; doi:10.1371/journal.pone.0162624)
Supplement: S1 Fig — PDF can be searched for species names. Apart from ID and species name, life stage, sex and coordinates of collecting locality are given. See S1 Table for more details on individual specimens in the tree. (PDF) [file pone.0162624.s002.pdf]

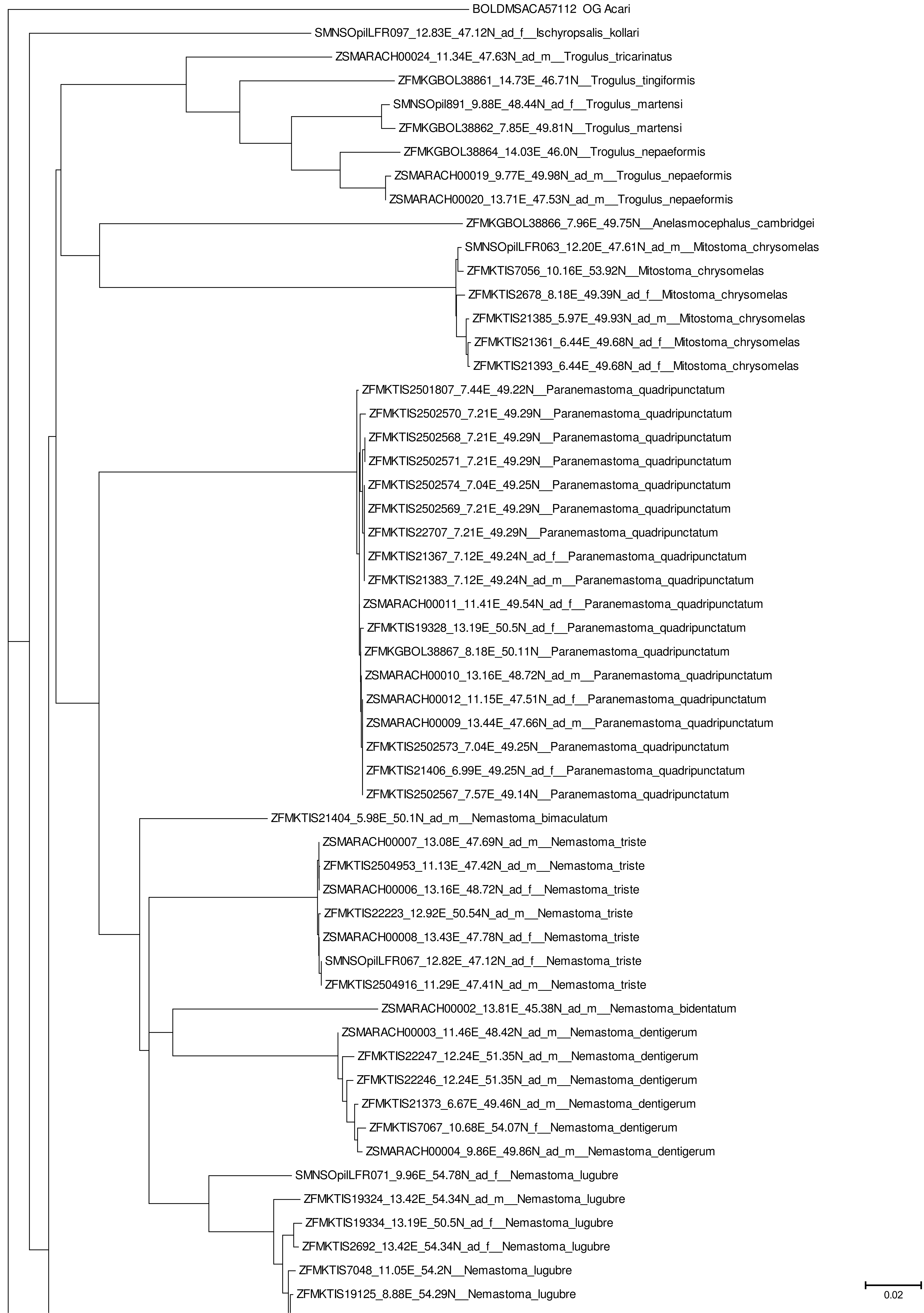

BOLDMSACA57112 OG Acari
SMNSOpilLFR097_12.83E_47.12N_ad_f__Ischyropsalis_kollari
ZSMARACH00024_11.34E_47.63N_ad_m__Trogulus_tricarinatus
ZFMKGBOL38861_14.73E_46.71N__Trogulus_tingiformis
SMNSOpil891_9.88E_48.44N_ad_f__Trogulus_martensi
ZFMKGBOL38862_7.85E_49.81N__Trogulus_martensi
ZFMKGBOL38864_14.03E_46.0N__Trogulus_nepaeformis
ZSMARACH00019_9.77E_49.98N_ad_m__Trogulus_nepaeformis
ZSMARACH00020_13.71E_47.53N_ad_m__Trogulus_nepaeformis
ZFMKGBOL38866_7.96E_49.75N__Anelasmocephalus_cambridgei
SMNSOpilLFR063_12.20E_47.61N_ad_m__Mitostoma_chrysomelas
ZFMKTIS7056_10.16E_53.92N__Mitostoma_chrysomelas
ZFMKTIS2678_8.18E_49.39N_ad_f__Mitostoma_chrysomelas
ZFMKTIS21385_5.97E_49.93N_ad_m__Mitostoma_chrysomelas
ZFMKTIS21361_6.44E_49.68N_ad_f__Mitostoma_chrysomelas
ZFMKTIS21393_6.44E_49.68N_ad_f__Mitostoma_chrysomelas
ZFMKTIS2501807_7.44E_49.22N__Paranemastoma_quadripunctatum
ZFMKTIS2502570_7.21E_49.29N__Paranemastoma_quadripunctatum
ZFMKTIS2502568_7.21E_49.29N__Paranemastoma_quadripunctatum
ZFMKTIS2502571_7.21E_49.29N__Paranemastoma_quadripunctatum
ZFMKTIS2502574_7.04E_49.25N__Paranemastoma_quadripunctatum
ZFMKTIS2502569_7.21E_49.29N__Paranemastoma_quadripunctatum
ZFMKTIS22707_7.21E_49.29N__Paranemastoma_quadripunctatum
ZFMKTIS21367_7.12E_49.24N_ad_f__Paranemastoma_quadripunctatum
ZFMKTIS21383_7.12E_49.24N_ad_m__Paranemastoma_quadripunctatum
ZSMARACH00011_11.41E_49.54N_ad_f__Paranemastoma_quadripunctatum
ZFMKTIS19328_13.19E_50.5N_ad_f__Paranemastoma_quadripunctatum
ZFMKGBOL38867_8.18E_50.11N__Paranemastoma_quadripunctatum
ZSMARACH00010_13.16E_48.72N_ad_m__Paranemastoma_quadripunctatum
ZSMARACH00012_11.15E_47.51N_ad_f__Paranemastoma_quadripunctatum
ZSMARACH00009_13.44E_47.66N_ad_m__Paranemastoma_quadripunctatum
ZFMKTIS2502573_7.04E_49.25N__Paranemastoma_quadripunctatum
ZFMKTIS21406_6.99E_49.25N_ad_f__Paranemastoma_quadripunctatum
ZFMKTIS2502567_7.57E_49.14N__Paranemastoma_quadripunctatum
ZFMKTIS21404_5.98E_50.1N_ad_m__Nemastoma_bimaculatum
ZSMARACH00007_13.08E_47.69N_ad_m__Nemastoma_triste
ZFMKTIS2504953_11.13E_47.42N_ad_m__Nemastoma_triste
ZSMARACH00006_13.16E_48.72N_ad_f__Nemastoma_triste
ZFMKTIS22223_12.92E_50.54N_ad_m__Nemastoma_triste
ZSMARACH00008_13.43E_47.78N_ad_f__Nemastoma_triste
SMNSOpilLFR067_12.82E_47.12N_ad_f__Nemastoma_triste
ZFMKTIS2504916_11.29E_47.41N_ad_m__Nemastoma_triste
ZSMARACH00002_13.81E_45.38N_ad_m__Nemastoma_bidentatum
ZSMARACH00003_11.46E_48.42N_ad_m__Nemastoma_dentigerum
ZFMKTIS22247_12.24E_51.35N_ad_m__Nemastoma_dentigerum
ZFMKTIS22246_12.24E_51.35N_ad_m__Nemastoma_dentigerum
ZFMKTIS21373_6.67E_49.46N_ad_m__Nemastoma_dentigerum
ZFMKTIS7067_10.68E_54.07N_f__Nemastoma_dentigerum
ZSMARACH00004_9.86E_49.86N_ad_m__Nemastoma_dentigerum
SMNSOpilLFR071_9.96E_54.78N_ad_f__Nemastoma_lugubre
ZFMKTIS19324_13.42E_54.34N_ad_m__Nemastoma_lugubre
ZFMKTIS19334_13.19E_50.5N_ad_f__Nemastoma_lugubre
ZFMKTIS2692_13.42E_54.34N_ad_f__Nemastoma_lugubre
ZFMKTIS7048_11.05E_54.2N__Nemastoma_lugubre
ZFMKTIS19125_8.88E_54.29N__Nemastoma_lugubre
0.02

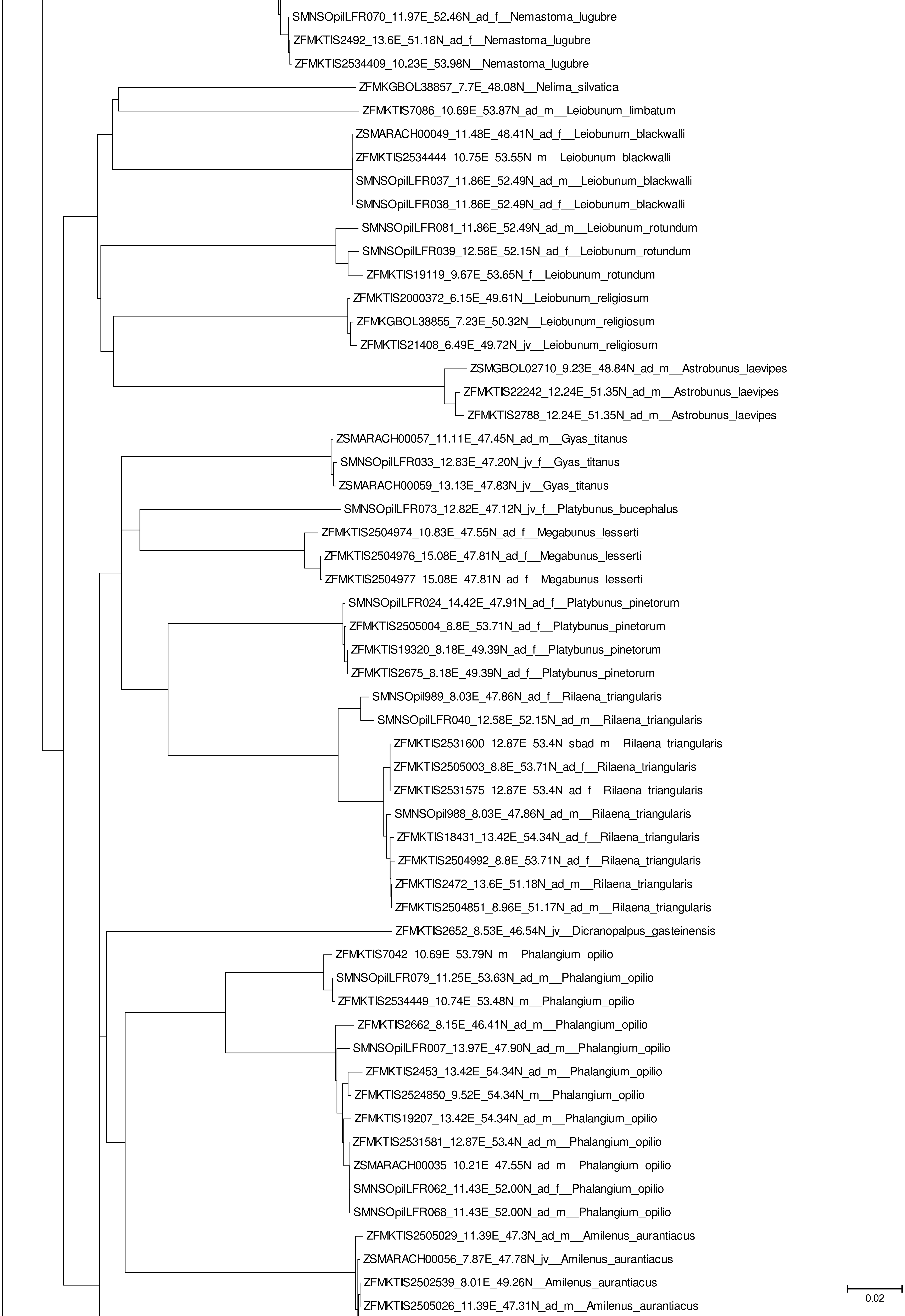

SMNSOpilLFR070_11.97E_52.46N_ad_f__Nemastoma_lugubre
ZFMKTIS2492_13.6E_51.18N_ad_f__Nemastoma_lugubre
ZFMKTIS2534409_10.23E_53.98N__Nemastoma_lugubre
ZFMKGBOL38857_7.7E_48.08N__Nelima_silvatica
ZFMKTIS7086_10.69E_53.87N_ad_m__Leiobunum_limbatum
ZSMARACH00049_11.48E_48.41N_ad_f__Leiobunum_blackwalli
ZFMKTIS2534444_10.75E_53.55N_m__Leiobunum_blackwalli
SMNSOpilLFR037_11.86E_52.49N_ad_m__Leiobunum_blackwalli
SMNSOpilLFR038_11.86E_52.49N_ad_f__Leiobunum_blackwalli
SMNSOpilLFR081_11.86E_52.49N_ad_m__Leiobunum_rotundum
SMNSOpilLFR039_12.58E_52.15N_ad_f__Leiobunum_rotundum
ZFMKTIS19119_9.67E_53.65N_f__Leiobunum_rotundum
ZFMKTIS2000372_6.15E_49.61N__Leiobunum_religiosum
ZFMKGBOL38855_7.23E_50.32N__Leiobunum_religiosum
ZFMKTIS21408_6.49E_49.72N_jv__Leiobunum_religiosum
ZSMGBOL02710_9.23E_48.84N_ad_m__Astrobunus_laevipes
ZFMKTIS22242_12.24E_51.35N_ad_m__Astrobunus_laevipes
ZFMKTIS2788_12.24E_51.35N_ad_m__Astrobunus_laevipes
ZSMARACH00057_11.11E_47.45N_ad_m__Gyas_titanus
SMNSOpilLFR033_12.83E_47.20N_jv_f__Gyas_titanus
ZSMARACH00059_13.13E_47.83N_jv__Gyas_titanus
SMNSOpilLFR073_12.82E_47.12N_jv_f__Platybunus_bucephalus
ZFMKTIS2504974_10.83E_47.55N_ad_f__Megabunus_lesserti
ZFMKTIS2504976_15.08E_47.81N_ad_f__Megabunus_lesserti
ZFMKTIS2504977_15.08E_47.81N_ad_f__Megabunus_lesserti
SMNSOpilLFR024_14.42E_47.91N_ad_f__Platybunus_pinetorum
ZFMKTIS2505004_8.8E_53.71N_ad_f__Platybunus_pinetorum
ZFMKTIS19320_8.18E_49.39N_ad_f__Platybunus_pinetorum
ZFMKTIS2675_8.18E_49.39N_ad_f__Platybunus_pinetorum
SMNSOpil989_8.03E_47.86N_ad_f__Rilaena_triangularis
SMNSOpilLFR040_12.58E_52.15N_ad_m__Rilaena_triangularis
ZFMKTIS2531600_12.87E_53.4N_sbad_m__Rilaena_triangularis
ZFMKTIS2505003_8.8E_53.71N_ad_f__Rilaena_triangularis
ZFMKTIS2531575_12.87E_53.4N_ad_f__Rilaena_triangularis
SMNSOpil988_8.03E_47.86N_ad_m__Rilaena_triangularis
ZFMKTIS18431_13.42E_54.34N_ad_f__Rilaena_triangularis
ZFMKTIS2504992_8.8E_53.71N_ad_f__Rilaena_triangularis
ZFMKTIS2472_13.6E_51.18N_ad_m__Rilaena_triangularis
ZFMKTIS2504851_8.96E_51.17N_ad_m__Rilaena_triangularis
ZFMKTIS2652_8.53E_46.54N_jv__Dicranopalpus_gasteinensis
ZFMKTIS7042_10.69E_53.79N_m__Phalangium_opilio
SMNSOpilLFR079_11.25E_53.63N_ad_m__Phalangium_opilio
ZFMKTIS2534449_10.74E_53.48N_m__Phalangium_opilio
ZFMKTIS2662_8.15E_46.41N_ad_m__Phalangium_opilio
SMNSOpilLFR007_13.97E_47.90N_ad_m__Phalangium_opilio
ZFMKTIS2453_13.42E_54.34N_ad_m__Phalangium_opilio
ZFMKTIS2524850_9.52E_54.34N_m__Phalangium_opilio
ZFMKTIS19207_13.42E_54.34N_ad_m__Phalangium_opilio
ZFMKTIS2531581_12.87E_53.4N_ad_m__Phalangium_opilio
ZSMARACH00035_10.21E_47.55N_ad_m__Phalangium_opilio
SMNSOpilLFR062_11.43E_52.00N_ad_f__Phalangium_opilio
SMNSOpilLFR068_11.43E_52.00N_ad_m__Phalangium_opilio
ZFMKTIS2505029_11.39E_47.3N_ad_m__Amilenus_aurantiacus
ZSMARACH00056_7.87E_47.78N_jv__Amilenus_aurantiacus
ZFMKTIS2502539_8.01E_49.26N__Amilenus_aurantiacus
ZFMKTIS2505026_11.39E_47.31N_ad_m__Amilenus_aurantiacus
0.02

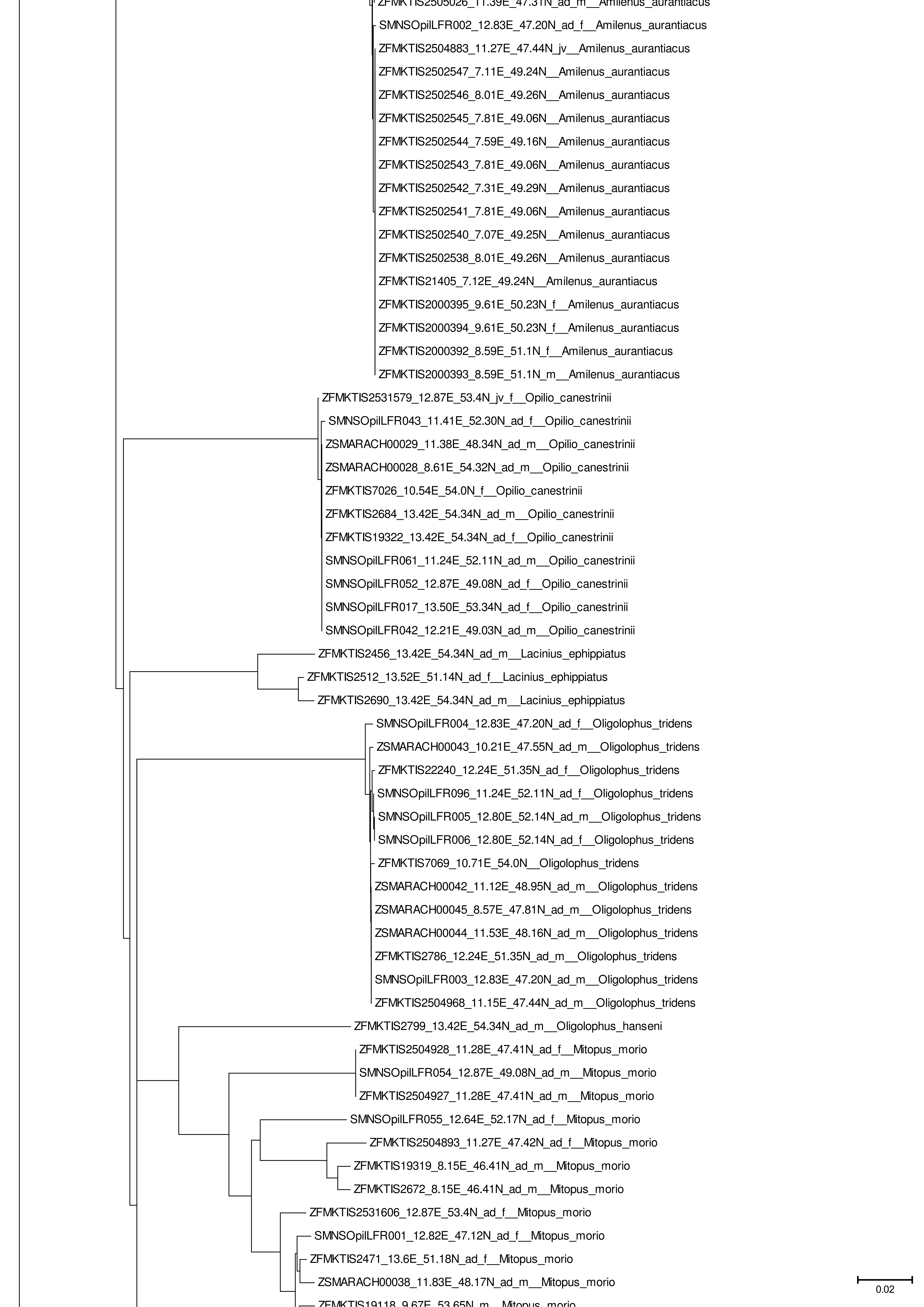
ZFMKTIS2505026_11.39E_47.31N_ad_m__Amilenus_aurantiacus
SMNSOpilLFR002_12.83E_47.20N_ad_f__Amilenus_aurantiacus
ZFMKTIS2504883_11.27E_47.44N_jv__Amilenus_aurantiacus
ZFMKTIS2502547_7.11E_49.24N__Amilenus_aurantiacus
ZFMKTIS2502546_8.01E_49.26N__Amilenus_aurantiacus
ZFMKTIS2502545_7.81E_49.06N__Amilenus_aurantiacus
ZFMKTIS2502544_7.59E_49.16N__Amilenus_aurantiacus
ZFMKTIS2502543_7.81E_49.06N__Amilenus_aurantiacus
ZFMKTIS2502542_7.31E_49.29N__Amilenus_aurantiacus
ZFMKTIS2502541_7.81E_49.06N__Amilenus_aurantiacus
ZFMKTIS2502540_7.07E_49.25N__Amilenus_aurantiacus
ZFMKTIS2502538_8.01E_49.26N__Amilenus_aurantiacus
ZFMKTIS21405_7.12E_49.24N__Amilenus_aurantiacus
ZFMKTIS2000395_9.61E_50.23N_f__Amilenus_aurantiacus
ZFMKTIS2000394_9.61E_50.23N_f__Amilenus_aurantiacus
ZFMKTIS2000392_8.59E_51.1N_f__Amilenus_aurantiacus
ZFMKTIS2000393_8.59E_51.1N_m__Amilenus_aurantiacus
ZFMKTIS2531579_12.87E_53.4N_jv_f__Opilio_canestrinii
SMNSOpilLFR043_11.41E_52.30N_ad_f__Opilio_canestrinii
ZSMARACH00029_11.38E_48.34N_ad_m__Opilio_canestrinii
ZSMARACH00028_8.61E_54.32N_ad_m__Opilio_canestrinii
ZFMKTIS7026_10.54E_54.0N_f__Opilio_canestrinii
ZFMKTIS2684_13.42E_54.34N_ad_m__Opilio_canestrinii
ZFMKTIS19322_13.42E_54.34N_ad_f__Opilio_canestrinii
SMNSOpilLFR061_11.24E_52.11N_ad_m__Opilio_canestrinii
SMNSOpilLFR052_12.87E_49.08N_ad_f__Opilio_canestrinii
SMNSOpilLFR017_13.50E_53.34N_ad_f__Opilio_canestrinii
SMNSOpilLFR042_12.21E_49.03N_ad_m__Opilio_canestrinii
ZFMKTIS2456_13.42E_54.34N_ad_m__Lacinius_ephippiatus
ZFMKTIS2512_13.52E_51.14N_ad_f__Lacinius_ephippiatus
ZFMKTIS2690_13.42E_54.34N_ad_m__Lacinius_ephippiatus
SMNSOpilLFR004_12.83E_47.20N_ad_f__Oligolophus_tridens
ZSMARACH00043_10.21E_47.55N_ad_m__Oligolophus_tridens
ZFMKTIS22240_12.24E_51.35N_ad_f__Oligolophus_tridens
SMNSOpilLFR096_11.24E_52.11N_ad_f__Oligolophus_tridens
SMNSOpilLFR005_12.80E_52.14N_ad_m__Oligolophus_tridens
SMNSOpilLFR006_12.80E_52.14N_ad_f__Oligolophus_tridens
ZFMKTIS7069_10.71E_54.0N__Oligolophus_tridens
ZSMARACH00042_11.12E_48.95N_ad_m__Oligolophus_tridens
ZSMARACH00045_8.57E_47.81N_ad_m__Oligolophus_tridens
ZSMARACH00044_11.53E_48.16N_ad_m__Oligolophus_tridens
ZFMKTIS2786_12.24E_51.35N_ad_m__Oligolophus_tridens
SMNSOpilLFR003_12.83E_47.20N_ad_m__Oligolophus_tridens
ZFMKTIS2504968_11.15E_47.44N_ad_m__Oligolophus_tridens
ZFMKTIS2799_13.42E_54.34N_ad_m__Oligolophus_hanseni
ZFMKTIS2504928_11.28E_47.41N_ad_f__Mitopus_morio
SMNSOpilLFR054_12.87E_49.08N_ad_m__Mitopus_morio
ZFMKTIS2504927_11.28E_47.41N_ad_m__Mitopus_morio
SMNSOpilLFR055_12.64E_52.17N_ad_f__Mitopus_morio
ZFMKTIS2504893_11.27E_47.42N_ad_f__Mitopus_morio
ZFMKTIS19319_8.15E_46.41N_ad_m__Mitopus_morio
ZFMKTIS2672_8.15E_46.41N_ad_m__Mitopus_morio
ZFMKTIS2531606_12.87E_53.4N_ad_f__Mitopus_morio
SMNSOpilLFR001_12.82E_47.12N_ad_f__Mitopus_morio
ZFMKTIS2471_13.6E_51.18N_ad_f__Mitopus_morio
ZSMARACH00038_11.83E_48.17N_ad_m__Mitopus_morio
ZFMKTIS19118_9.67E_53.65N_m__Mitopus_morio
0.02

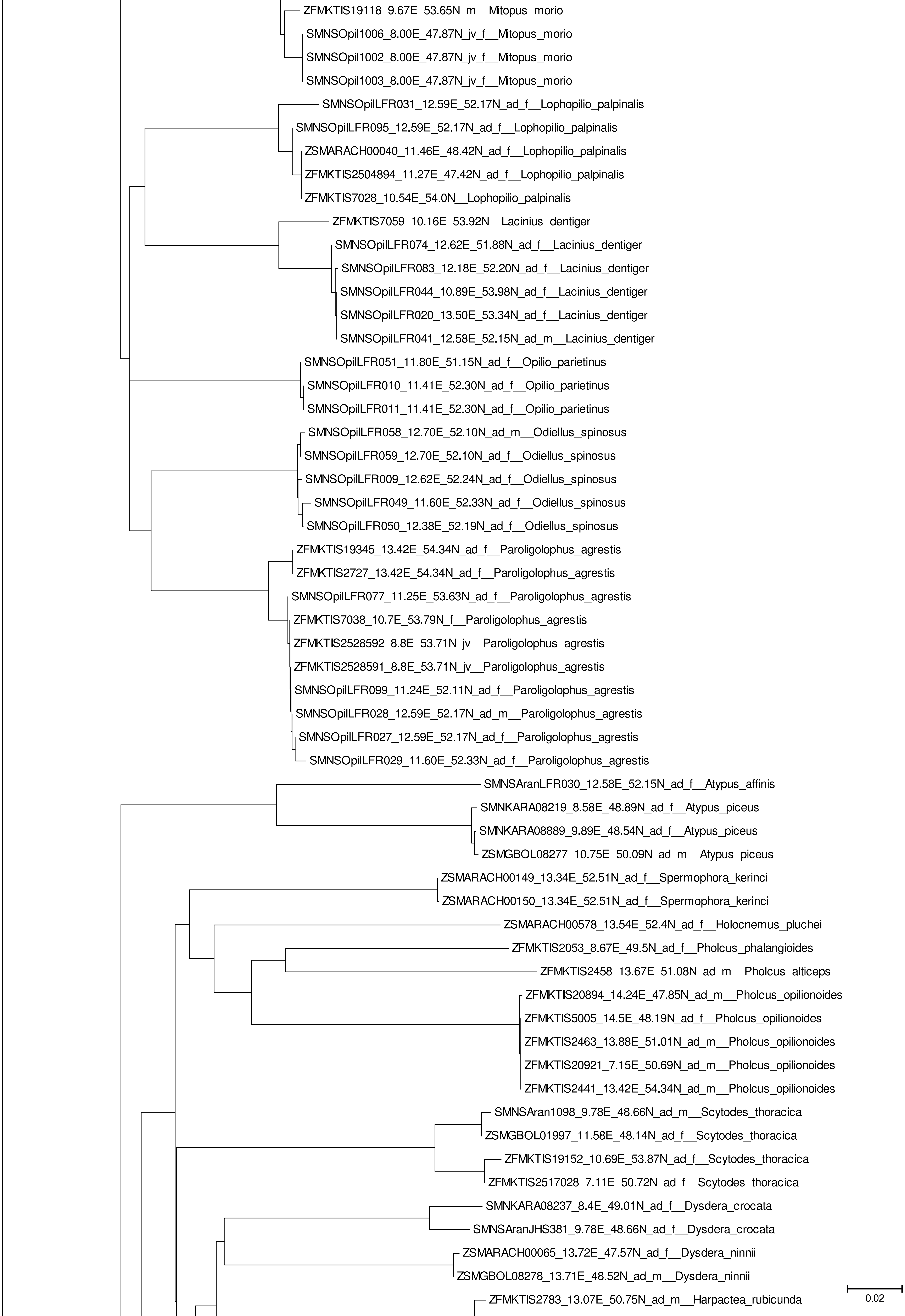
ZFMKTIS19118_9.67E_53.65N_m__Mitopus_morio
SMNSOpil1006_8.00E_47.87N_jv_f__Mitopus_morio
SMNSOpil1002_8.00E_47.87N_jv_f__Mitopus_morio
SMNSOpil1003_8.00E_47.87N_jv_f__Mitopus_morio
SMNSOpilLFR031_12.59E_52.17N_ad_f__Lophopilio_palpinalis
SMNSOpilLFR095_12.59E_52.17N_ad_f__Lophopilio_palpinalis
ZSMARACH00040_11.46E_48.42N_ad_f__Lophopilio_palpinalis
ZFMKTIS2504894_11.27E_47.42N_ad_f__Lophopilio_palpinalis
ZFMKTIS7028_10.54E_54.0N__Lophopilio_palpinalis
ZFMKTIS7059_10.16E_53.92N__Lacinius_dentiger
SMNSOpilLFR074_12.62E_51.88N_ad_f__Lacinius_dentiger
SMNSOpilLFR083_12.18E_52.20N_ad_f__Lacinius_dentiger
SMNSOpilLFR044_10.89E_53.98N_ad_f__Lacinius_dentiger
SMNSOpilLFR020_13.50E_53.34N_ad_f__Lacinius_dentiger
SMNSOpilLFR041_12.58E_52.15N_ad_m__Lacinius_dentiger
SMNSOpilLFR051_11.80E_51.15N_ad_f__Opilio_parietinus
SMNSOpilLFR010_11.41E_52.30N_ad_f__Opilio_parietinus
SMNSOpilLFR011_11.41E_52.30N_ad_f__Opilio_parietinus
SMNSOpilLFR058_12.70E_52.10N_ad_m__Odiellus_spinosus
SMNSOpilLFR059_12.70E_52.10N_ad_f__Odiellus_spinosus
SMNSOpilLFR009_12.62E_52.24N_ad_f__Odiellus_spinosus
SMNSOpilLFR049_11.60E_52.33N_ad_f__Odiellus_spinosus
SMNSOpilLFR050_12.38E_52.19N_ad_f__Odiellus_spinosus
ZFMKTIS19345_13.42E_54.34N_ad_f__Paroligolophus_agrestis
ZFMKTIS2727_13.42E_54.34N_ad_f__Paroligolophus_agrestis
SMNSOpilLFR077_11.25E_53.63N_ad_f__Paroligolophus_agrestis
ZFMKTIS7038_10.7E_53.79N_f__Paroligolophus_agrestis
ZFMKTIS2528592_8.8E_53.71N_jv__Paroligolophus_agrestis
ZFMKTIS2528591_8.8E_53.71N_jv__Paroligolophus_agrestis
SMNSOpilLFR099_11.24E_52.11N_ad_f__Paroligolophus_agrestis
SMNSOpilLFR028_12.59E_52.17N_ad_m__Paroligolophus_agrestis
SMNSOpilLFR027_12.59E_52.17N_ad_f__Paroligolophus_agrestis
SMNSOpilLFR029_11.60E_52.33N_ad_f__Paroligolophus_agrestis
SMNSAranLFR030_12.58E_52.15N_ad_f__Atypus_affinis
SMNKARA08219_8.58E_48.89N_ad_f__Atypus_piceus
SMNKARA08889_9.89E_48.54N_ad_f__Atypus_piceus
ZSMGBOL08277_10.75E_50.09N_ad_m__Atypus_piceus
ZSMARACH00149_13.34E_52.51N_ad_f__Spermophora_kerinci
ZSMARACH00150_13.34E_52.51N_ad_f__Spermophora_kerinci
ZSMARACH00578_13.54E_52.4N_ad_f__Holocnemus_pluchei
ZFMKTIS2053_8.67E_49.5N_ad_f__Pholcus_phalangioides
ZFMKTIS2458_13.67E_51.08N_ad_m__Pholcus_alticeps
ZFMKTIS20894_14.24E_47.85N_ad_m__Pholcus_opilionoides
ZFMKTIS5005_14.5E_48.19N_ad_f__Pholcus_opilionoides
ZFMKTIS2463_13.88E_51.01N_ad_m__Pholcus_opilionoides
ZFMKTIS20921_7.15E_50.69N_ad_m__Pholcus_opilionoides
ZFMKTIS2441_13.42E_54.34N_ad_m__Pholcus_opilionoides
SMNSAran1098_9.78E_48.66N_ad_m__Scytodes_thoracica
ZSMGBOL01997_11.58E_48.14N_ad_f__Scytodes_thoracica
ZFMKTIS19152_10.69E_53.87N_ad_f__Scytodes_thoracica
ZFMKTIS2517028_7.11E_50.72N_ad_f__Scytodes_thoracica
SMNKARA08237_8.4E_49.01N_ad_f__Dysdera_crocata
SMNSAranJHS381_9.78E_48.66N_ad_f__Dysdera_crocata
ZSMARACH00065_13.72E_47.57N_ad_f__Dysdera_ninnii
ZSMGBOL08278_13.71E_48.52N_ad_m__Dysdera_ninnii
ZFMKTIS2783_13.07E_50.75N_ad_m__Harpactea_rubicunda
0.02

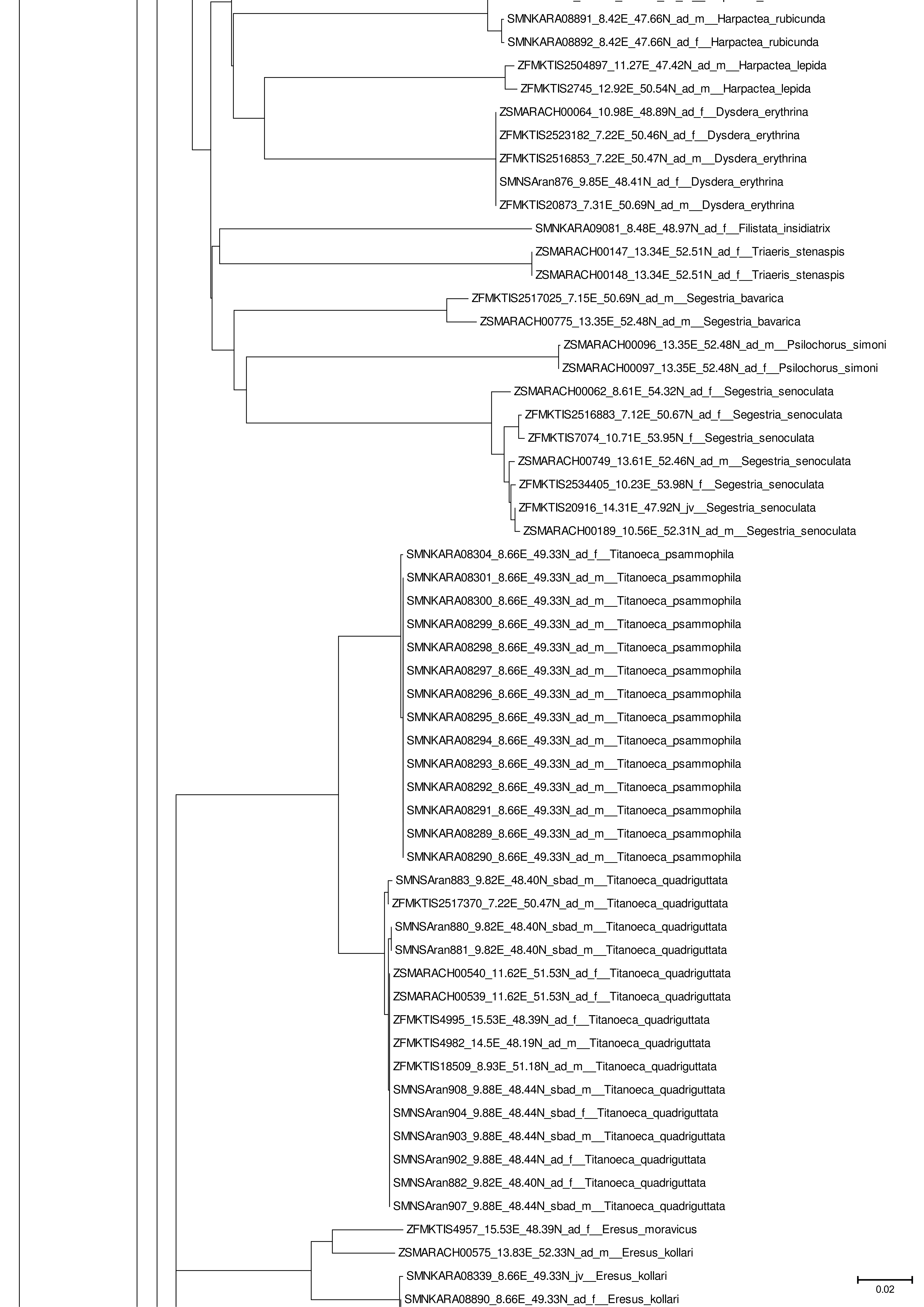

SMNKARA08891_8.42E_47.66N_ad_m__Harpactea_rubicunda
SMNKARA08892_8.42E_47.66N_ad_f__Harpactea_rubicunda
ZFMKTIS2504897_11.27E_47.42N_ad_m__Harpactea_lepida
ZFMKTIS2745_12.92E_50.54N_ad_m__Harpactea_lepida
ZSMARACH00064_10.98E_48.89N_ad_f__Dysdera_erythrina
ZFMKTIS2523182_7.22E_50.46N_ad_f__Dysdera_erythrina
ZFMKTIS2516853_7.22E_50.47N_ad_m__Dysdera_erythrina
SMNSAran876_9.85E_48.41N_ad_f__Dysdera_erythrina
ZFMKTIS20873_7.31E_50.69N_ad_m__Dysdera_erythrina
SMNKARA09081_8.48E_48.97N_ad_f__Filistata_insidiatrix
ZSMARACH00147_13.34E_52.51N_ad_f__Triaeris_stenaspis
ZSMARACH00148_13.34E_52.51N_ad_f__Triaeris_stenaspis
ZFMKTIS2517025_7.15E_50.69N_ad_m__Segestria_bavarica
ZSMARACH00775_13.35E_52.48N_ad_m__Segestria_bavarica
ZSMARACH00096_13.35E_52.48N_ad_m__Psilochorus_simoni
ZSMARACH00097_13.35E_52.48N_ad_f__Psilochorus_simoni
ZSMARACH00062_8.61E_54.32N_ad_f__Segestria_senoculata
ZFMKTIS2516883_7.12E_50.67N_ad_f__Segestria_senoculata
ZFMKTIS7074_10.71E_53.95N_f__Segestria_senoculata
ZSMARACH00749_13.61E_52.46N_ad_m__Segestria_senoculata
ZFMKTIS2534405_10.23E_53.98N_f__Segestria_senoculata
ZFMKTIS20916_14.31E_47.92N_jv__Segestria_senoculata
ZSMARACH00189_10.56E_52.31N_ad_m__Segestria_senoculata
SMNKARA08304_8.66E_49.33N_ad_f__Titanoeca_psammophila
SMNKARA08301_8.66E_49.33N_ad_m__Titanoeca_psammophila
SMNKARA08300_8.66E_49.33N_ad_m__Titanoeca_psammophila
SMNKARA08299_8.66E_49.33N_ad_m__Titanoeca_psammophila
SMNKARA08298_8.66E_49.33N_ad_m__Titanoeca_psammophila
SMNKARA08297_8.66E_49.33N_ad_m__Titanoeca_psammophila
SMNKARA08296_8.66E_49.33N_ad_m__Titanoeca_psammophila
SMNKARA08295_8.66E_49.33N_ad_m__Titanoeca_psammophila
SMNKARA08294_8.66E_49.33N_ad_m__Titanoeca_psammophila
SMNKARA08293_8.66E_49.33N_ad_m__Titanoeca_psammophila
SMNKARA08292_8.66E_49.33N_ad_m__Titanoeca_psammophila
SMNKARA08291_8.66E_49.33N_ad_m__Titanoeca_psammophila
SMNKARA08289_8.66E_49.33N_ad_m__Titanoeca_psammophila
SMNKARA08290_8.66E_49.33N_ad_m__Titanoeca_psammophila
SMNSAran883_9.82E_48.40N_sbad_m__Titanoeca_quadriguttata
ZFMKTIS2517370_7.22E_50.47N_ad_m__Titanoeca_quadriguttata
SMNSAran880_9.82E_48.40N_sbad_m__Titanoeca_quadriguttata
SMNSAran881_9.82E_48.40N_sbad_m__Titanoeca_quadriguttata
ZSMARACH00540_11.62E_51.53N_ad_f__Titanoeca_quadriguttata
ZSMARACH00539_11.62E_51.53N_ad_f__Titanoeca_quadriguttata
ZFMKTIS4995_15.53E_48.39N_ad_f__Titanoeca_quadriguttata
ZFMKTIS4982_14.5E_48.19N_ad_m__Titanoeca_quadriguttata
ZFMKTIS18509_8.93E_51.18N_ad_m__Titanoeca_quadriguttata
SMNSAran908_9.88E_48.44N_sbad_m__Titanoeca_quadriguttata
SMNSAran904_9.88E_48.44N_sbad_f__Titanoeca_quadriguttata
SMNSAran903_9.88E_48.44N_sbad_m__Titanoeca_quadriguttata
SMNSAran902_9.88E_48.44N_ad_f__Titanoeca_quadriguttata
SMNSAran882_9.82E_48.40N_ad_f__Titanoeca_quadriguttata
SMNSAran907_9.88E_48.44N_sbad_m__Titanoeca_quadriguttata
ZFMKTIS4957_15.53E_48.39N_ad_f__Eresus_moravicus
ZSMARACH00575_13.83E_52.33N_ad_m__Eresus_kollari
SMNKARA08339_8.66E_49.33N_jv__Eresus_kollari
SMNKARA08890_8.66E_49.33N_ad_f__Eresus_kollari
0.02

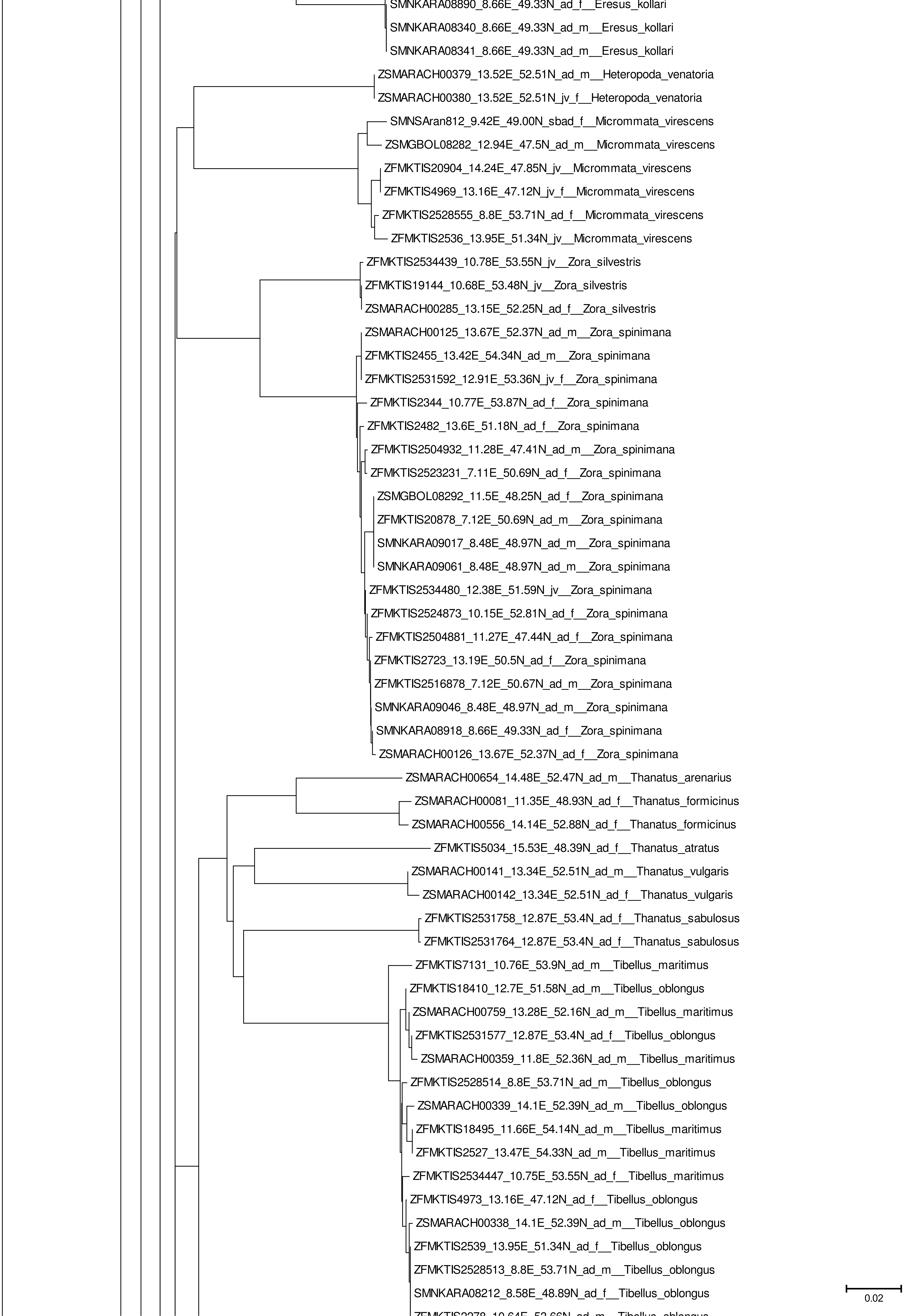

SMNKARA08890_8.66E_49.33N_ad_f__Eresus_kollari
SMNKARA08340_8.66E_49.33N_ad_m__Eresus_kollari
SMNKARA08341_8.66E_49.33N_ad_m__Eresus_kollari
ZSMARACH00379_13.52E_52.51N_ad_m__Heteropoda_venatoria
ZSMARACH00380_13.52E_52.51N_jv_f__Heteropoda_venatoria
SMNSAran812_9.42E_49.00N_sbad_f__Micrommata_virescens
ZSMGBOL08282_12.94E_47.5N_ad_m__Micrommata_virescens
ZFMKTIS20904_14.24E_47.85N_jv__Micrommata_virescens
ZFMKTIS4969_13.16E_47.12N_jv_f__Micrommata_virescens
ZFMKTIS2528555_8.8E_53.71N_ad_f__Micrommata_virescens
ZFMKTIS2536_13.95E_51.34N_jv__Micrommata_virescens
ZFMKTIS2534439_10.78E_53.55N_jv__Zora_silvestris
ZFMKTIS19144_10.68E_53.48N_jv__Zora_silvestris
ZSMARACH00285_13.15E_52.25N_ad_f__Zora_silvestris
ZSMARACH00125_13.67E_52.37N_ad_m__Zora_spinimana
ZFMKTIS2455_13.42E_54.34N_ad_m__Zora_spinimana
ZFMKTIS2531592_12.91E_53.36N_jv_f__Zora_spinimana
ZFMKTIS2344_10.77E_53.87N_ad_f__Zora_spinimana
ZFMKTIS2482_13.6E_51.18N_ad_f__Zora_spinimana
ZFMKTIS2504932_11.28E_47.41N_ad_m__Zora_spinimana
ZFMKTIS2523231_7.11E_50.69N_ad_f__Zora_spinimana
ZSMGBOL08292_11.5E_48.25N_ad_f__Zora_spinimana
ZFMKTIS20878_7.12E_50.69N_ad_m__Zora_spinimana
SMNKARA09017_8.48E_48.97N_ad_m__Zora_spinimana
SMNKARA09061_8.48E_48.97N_ad_m__Zora_spinimana
ZFMKTIS2534480_12.38E_51.59N_jv__Zora_spinimana
ZFMKTIS2524873_10.15E_52.81N_ad_f__Zora_spinimana
ZFMKTIS2504881_11.27E_47.44N_ad_f__Zora_spinimana
ZFMKTIS2723_13.19E_50.5N_ad_f__Zora_spinimana
ZFMKTIS2516878_7.12E_50.67N_ad_m__Zora_spinimana
SMNKARA09046_8.48E_48.97N_ad_m__Zora_spinimana
SMNKARA08918_8.66E_49.33N_ad_f__Zora_spinimana
ZSMARACH00126_13.67E_52.37N_ad_f__Zora_spinimana
ZSMARACH00654_14.48E_52.47N_ad_m__Thanatus_arenarius
ZSMARACH00081_11.35E_48.93N_ad_f__Thanatus_formicinus
ZSMARACH00556_14.14E_52.88N_ad_f__Thanatus_formicinus
ZFMKTIS5034_15.53E_48.39N_ad_f__Thanatus_atratus
ZSMARACH00141_13.34E_52.51N_ad_m__Thanatus_vulgaris
ZSMARACH00142_13.34E_52.51N_ad_f__Thanatus_vulgaris
ZFMKTIS2531758_12.87E_53.4N_ad_f__Thanatus_sabulosus
ZFMKTIS2531764_12.87E_53.4N_ad_f__Thanatus_sabulosus
ZFMKTIS7131_10.76E_53.9N_ad_m__Tibellus_maritimus
ZFMKTIS18410_12.7E_51.58N_ad_m__Tibellus_oblongus
ZSMARACH00759_13.28E_52.16N_ad_m__Tibellus_maritimus
ZFMKTIS2531577_12.87E_53.4N_ad_f__Tibellus_oblongus
ZSMARACH00359_11.8E_52.36N_ad_m__Tibellus_maritimus
ZFMKTIS2528514_8.8E_53.71N_ad_m__Tibellus_oblongus
ZSMARACH00339_14.1E_52.39N_ad_m__Tibellus_oblongus
ZFMKTIS18495_11.66E_54.14N_ad_m__Tibellus_maritimus
ZFMKTIS2527_13.47E_54.33N_ad_m__Tibellus_maritimus
ZFMKTIS2534447_10.75E_53.55N_ad_f__Tibellus_maritimus
ZFMKTIS4973_13.16E_47.12N_ad_f__Tibellus_oblongus
ZSMARACH00338_14.1E_52.39N_ad_m__Tibellus_oblongus
ZFMKTIS2539_13.95E_51.34N_ad_f__Tibellus_oblongus
ZFMKTIS2528513_8.8E_53.71N_ad_m__Tibellus_oblongus
SMNKARA08212_8.58E_48.89N_ad_f__Tibellus_oblongus
0.02

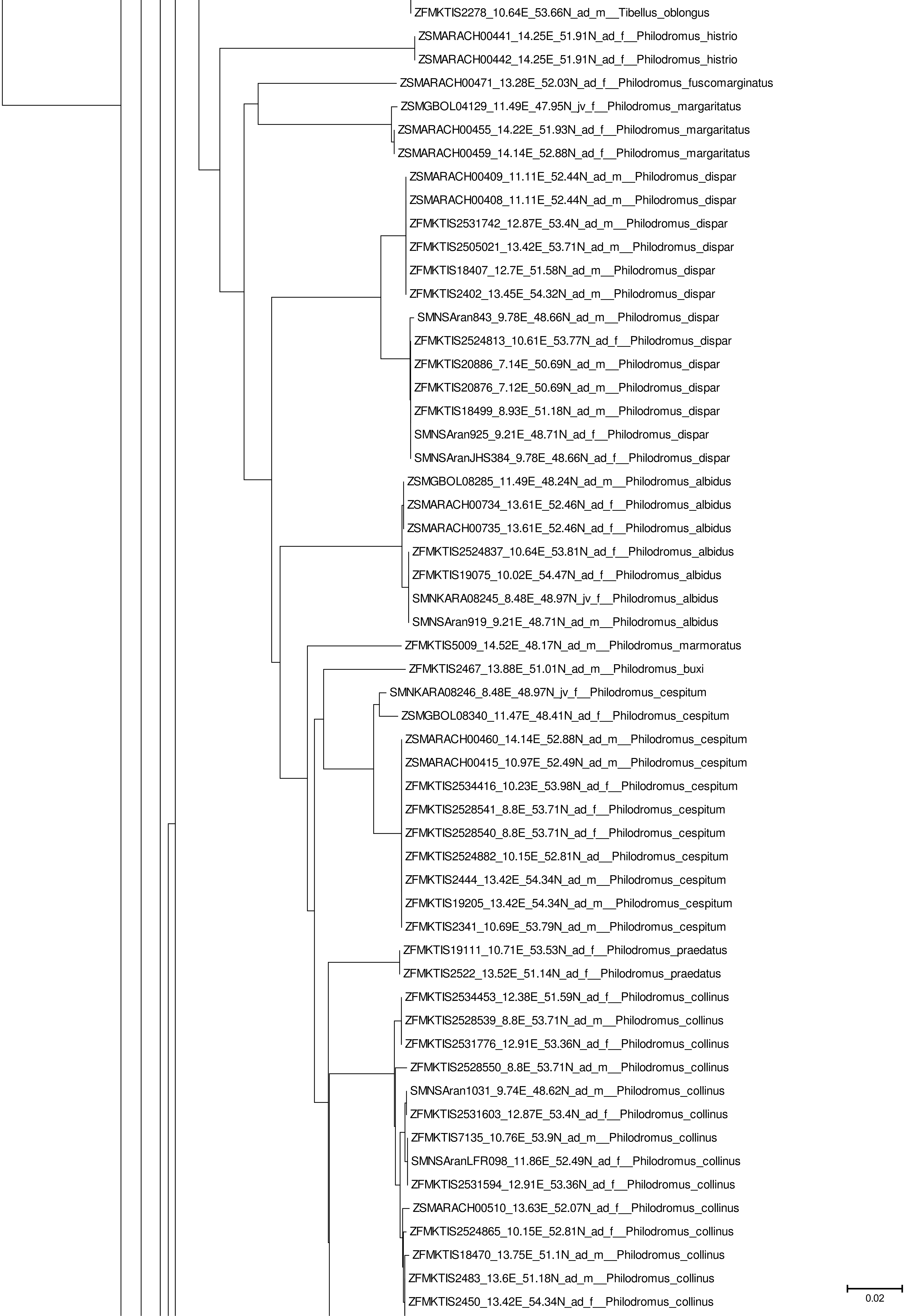
ZFMKTIS2278_10.64E_53.66N_ad_m__Tibellus_oblongus
ZSMARACH00441_14.25E_51.91N_ad_f__Philodromus_histrio
ZSMARACH00442_14.25E_51.91N_ad_f__Philodromus_histrio
ZSMARACH00471_13.28E_52.03N_ad_f__Philodromus_fuscomarginatus
ZSMGBOL04129_11.49E_47.95N_jv_f__Philodromus_margaritatus
ZSMARACH00455_14.22E_51.93N_ad_f__Philodromus_margaritatus
ZSMARACH00459_14.14E_52.88N_ad_f__Philodromus_margaritatus
ZSMARACH00409_11.11E_52.44N_ad_m__Philodromus_dispar
ZSMARACH00408_11.11E_52.44N_ad_m__Philodromus_dispar
ZFMKTIS2531742_12.87E_53.4N_ad_m__Philodromus_dispar
ZFMKTIS2505021_13.42E_53.71N_ad_m__Philodromus_dispar
ZFMKTIS18407_12.7E_51.58N_ad_m__Philodromus_dispar
ZFMKTIS2402_13.45E_54.32N_ad_m__Philodromus_dispar
SMNSAran843_9.78E_48.66N_ad_m__Philodromus_dispar
ZFMKTIS2524813_10.61E_53.77N_ad_f__Philodromus_dispar
ZFMKTIS20886_7.14E_50.69N_ad_m__Philodromus_dispar
ZFMKTIS20876_7.12E_50.69N_ad_m__Philodromus_dispar
ZFMKTIS18499_8.93E_51.18N_ad_m__Philodromus_dispar
SMNSAran925_9.21E_48.71N_ad_f__Philodromus_dispar
SMNSAranJHS384_9.78E_48.66N_ad_f__Philodromus_dispar
ZSMGBOL08285_11.49E_48.24N_ad_m__Philodromus_albidus
ZSMARACH00734_13.61E_52.46N_ad_f__Philodromus_albidus
ZSMARACH00735_13.61E_52.46N_ad_f__Philodromus_albidus
ZFMKTIS2524837_10.64E_53.81N_ad_f__Philodromus_albidus
ZFMKTIS19075_10.02E_54.47N_ad_f__Philodromus_albidus
SMNKARA08245_8.48E_48.97N_jv_f__Philodromus_albidus
SMNSAran919_9.21E_48.71N_ad_m__Philodromus_albidus
ZFMKTIS5009_14.52E_48.17N_ad_m__Philodromus_marmoratus
ZFMKTIS2467_13.88E_51.01N_ad_m__Philodromus_buxi
SMNKARA08246_8.48E_48.97N_jv_f__Philodromus_cespitum
ZSMGBOL08340_11.47E_48.41N_ad_f__Philodromus_cespitum
ZSMARACH00460_14.14E_52.88N_ad_m__Philodromus_cespitum
ZSMARACH00415_10.97E_52.49N_ad_m__Philodromus_cespitum
ZFMKTIS2534416_10.23E_53.98N_ad_f__Philodromus_cespitum
ZFMKTIS2528541_8.8E_53.71N_ad_f__Philodromus_cespitum
ZFMKTIS2528540_8.8E_53.71N_ad_f__Philodromus_cespitum
ZFMKTIS2524882_10.15E_52.81N_ad__Philodromus_cespitum
ZFMKTIS2444_13.42E_54.34N_ad_m__Philodromus_cespitum
ZFMKTIS19205_13.42E_54.34N_ad_m__Philodromus_cespitum
ZFMKTIS2341_10.69E_53.79N_ad_m__Philodromus_cespitum
ZFMKTIS19111_10.71E_53.53N_ad_f__Philodromus_praedatus
ZFMKTIS2522_13.52E_51.14N_ad_f__Philodromus_praedatus
ZFMKTIS2534453_12.38E_51.59N_ad_f__Philodromus_collinus
ZFMKTIS2528539_8.8E_53.71N_ad_m__Philodromus_collinus
ZFMKTIS2531776_12.91E_53.36N_ad_f__Philodromus_collinus
ZFMKTIS2528550_8.8E_53.71N_ad_m__Philodromus_collinus
SMNSAran1031_9.74E_48.62N_ad_m__Philodromus_collinus
ZFMKTIS2531603_12.87E_53.4N_ad_f__Philodromus_collinus
ZFMKTIS7135_10.76E_53.9N_ad_m__Philodromus_collinus
SMNSAranLFR098_11.86E_52.49N_ad_f__Philodromus_collinus
ZFMKTIS2531594_12.91E_53.36N_ad_f__Philodromus_collinus
ZSMARACH00510_13.63E_52.07N_ad_f__Philodromus_collinus
ZFMKTIS2524865_10.15E_52.81N_ad_f__Philodromus_collinus
ZFMKTIS18470_13.75E_51.1N_ad_m__Philodromus_collinus
ZFMKTIS2483_13.6E_51.18N_ad_m__Philodromus_collinus
ZFMKTIS2450_13.42E_54.34N_ad_f__Philodromus_collinus
0.02

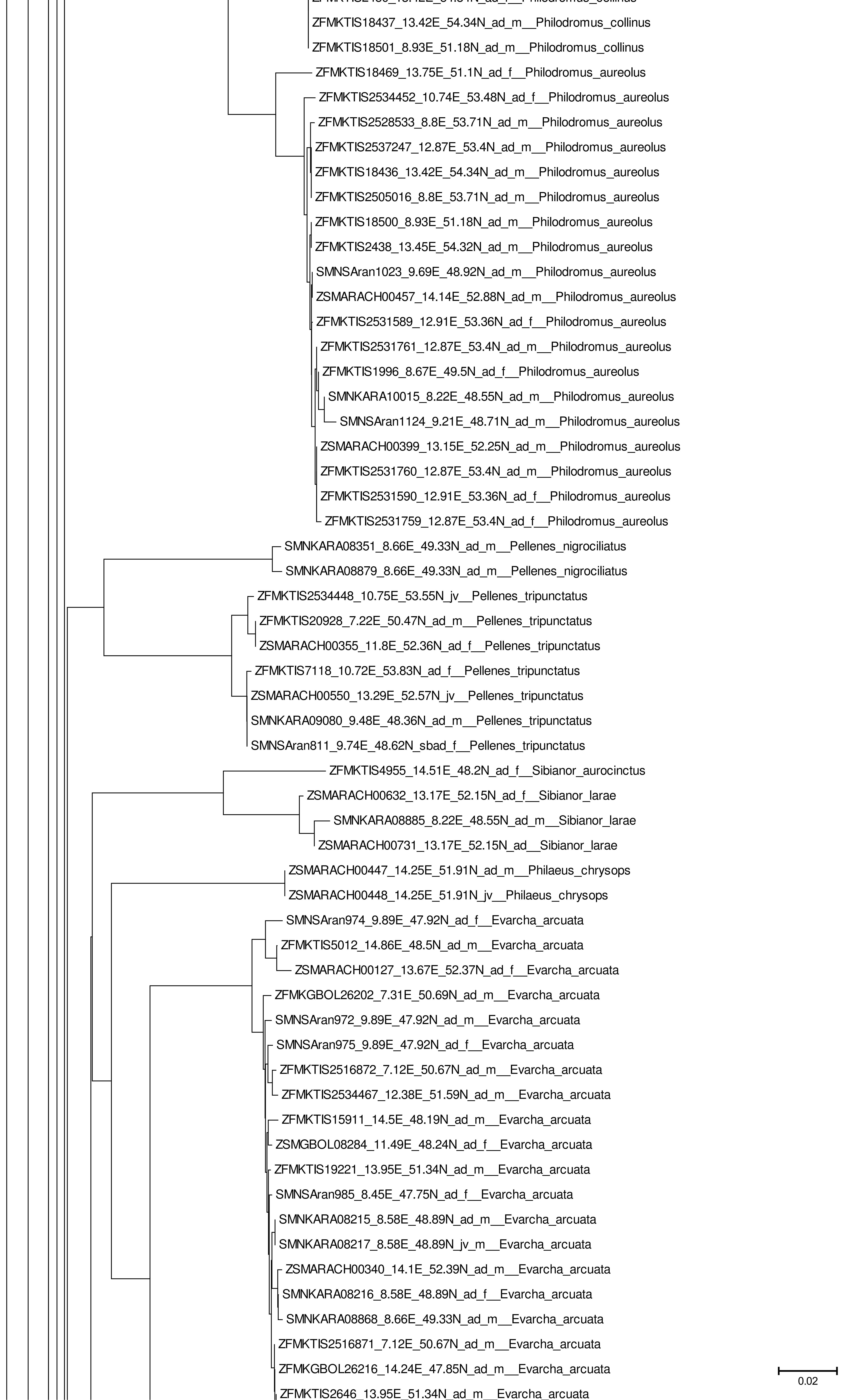

ZFMKTIS18437_13.42E_54.34N_ad_m__Philodromus_collinus
ZFMKTIS18501_8.93E_51.18N_ad_m__Philodromus_collinus
ZFMKTIS18469_13.75E_51.1N_ad_f__Philodromus_aureolus
ZFMKTIS2534452_10.74E_53.48N_ad_f__Philodromus_aureolus
ZFMKTIS2528533_8.8E_53.71N_ad_m__Philodromus_aureolus
ZFMKTIS2537247_12.87E_53.4N_ad_m__Philodromus_aureolus
ZFMKTIS18436_13.42E_54.34N_ad_m__Philodromus_aureolus
ZFMKTIS2505016_8.8E_53.71N_ad_m__Philodromus_aureolus
ZFMKTIS18500_8.93E_51.18N_ad_m__Philodromus_aureolus
ZFMKTIS2438_13.45E_54.32N_ad_m__Philodromus_aureolus
SMNSAran1023_9.69E_48.92N_ad_m__Philodromus_aureolus
ZSMARACH00457_14.14E_52.88N_ad_m__Philodromus_aureolus
ZFMKTIS2531589_12.91E_53.36N_ad_f__Philodromus_aureolus
ZFMKTIS2531761_12.87E_53.4N_ad_m__Philodromus_aureolus
ZFMKTIS1996_8.67E_49.5N_ad_f__Philodromus_aureolus
SMNKARA10015_8.22E_48.55N_ad_m__Philodromus_aureolus
SMNSAran1124_9.21E_48.71N_ad_m__Philodromus_aureolus
ZSMARACH00399_13.15E_52.25N_ad_m__Philodromus_aureolus
ZFMKTIS2531760_12.87E_53.4N_ad_m__Philodromus_aureolus
ZFMKTIS2531590_12.91E_53.36N_ad_f__Philodromus_aureolus
ZFMKTIS2531759_12.87E_53.4N_ad_f__Philodromus_aureolus
SMNKARA08351_8.66E_49.33N_ad_m__Pellenes_nigrociliatus
SMNKARA08879_8.66E_49.33N_ad_m__Pellenes_nigrociliatus
ZFMKTIS2534448_10.75E_53.55N_jv__Pellenes_tripunctatus
ZFMKTIS20928_7.22E_50.47N_ad_m__Pellenes_tripunctatus
ZSMARACH00355_11.8E_52.36N_ad_f__Pellenes_tripunctatus
ZFMKTIS7118_10.72E_53.83N_ad_f__Pellenes_tripunctatus
ZSMARACH00550_13.29E_52.57N_jv__Pellenes_tripunctatus
SMNKARA09080_9.48E_48.36N_ad_m__Pellenes_tripunctatus
SMNSAran811_9.74E_48.62N_sbad_f__Pellenes_tripunctatus
ZFMKTIS4955_14.51E_48.2N_ad_f__Sibianor_aurocinctus
ZSMARACH00632_13.17E_52.15N_ad_f__Sibianor_larae
SMNKARA08885_8.22E_48.55N_ad_m__Sibianor_larae
ZSMARACH00731_13.17E_52.15N_ad__Sibianor_larae
ZSMARACH00447_14.25E_51.91N_ad_m__Philaeus_chrysops
ZSMARACH00448_14.25E_51.91N_jv__Philaeus_chrysops
SMNSAran974_9.89E_47.92N_ad_f__Evarcha_arcuata
ZFMKTIS5012_14.86E_48.5N_ad_m__Evarcha_arcuata
ZSMARACH00127_13.67E_52.37N_ad_f__Evarcha_arcuata
ZFMKGBOL26202_7.31E_50.69N_ad_m__Evarcha_arcuata
SMNSAran972_9.89E_47.92N_ad_m__Evarcha_arcuata
SMNSAran975_9.89E_47.92N_ad_f__Evarcha_arcuata
ZFMKTIS2516872_7.12E_50.67N_ad_m__Evarcha_arcuata
ZFMKTIS2534467_12.38E_51.59N_ad_m__Evarcha_arcuata
ZFMKTIS15911_14.5E_48.19N_ad_m__Evarcha_arcuata
ZSMGBOL08284_11.49E_48.24N_ad_f__Evarcha_arcuata
ZFMKTIS19221_13.95E_51.34N_ad_m__Evarcha_arcuata
SMNSAran985_8.45E_47.75N_ad_f__Evarcha_arcuata
SMNKARA08215_8.58E_48.89N_ad_m__Evarcha_arcuata
SMNKARA08217_8.58E_48.89N_jv_m__Evarcha_arcuata
ZSMARACH00340_14.1E_52.39N_ad_m__Evarcha_arcuata
SMNKARA08216_8.58E_48.89N_ad_f__Evarcha_arcuata
SMNKARA08868_8.66E_49.33N_ad_m__Evarcha_arcuata
ZFMKTIS2516871_7.12E_50.67N_ad_m__Evarcha_arcuata
ZFMKGBOL26216_14.24E_47.85N_ad_m__Evarcha_arcuata
ZFMKTIS2646_13.95E_51.34N_ad_m__Evarcha_arcuata
0.02

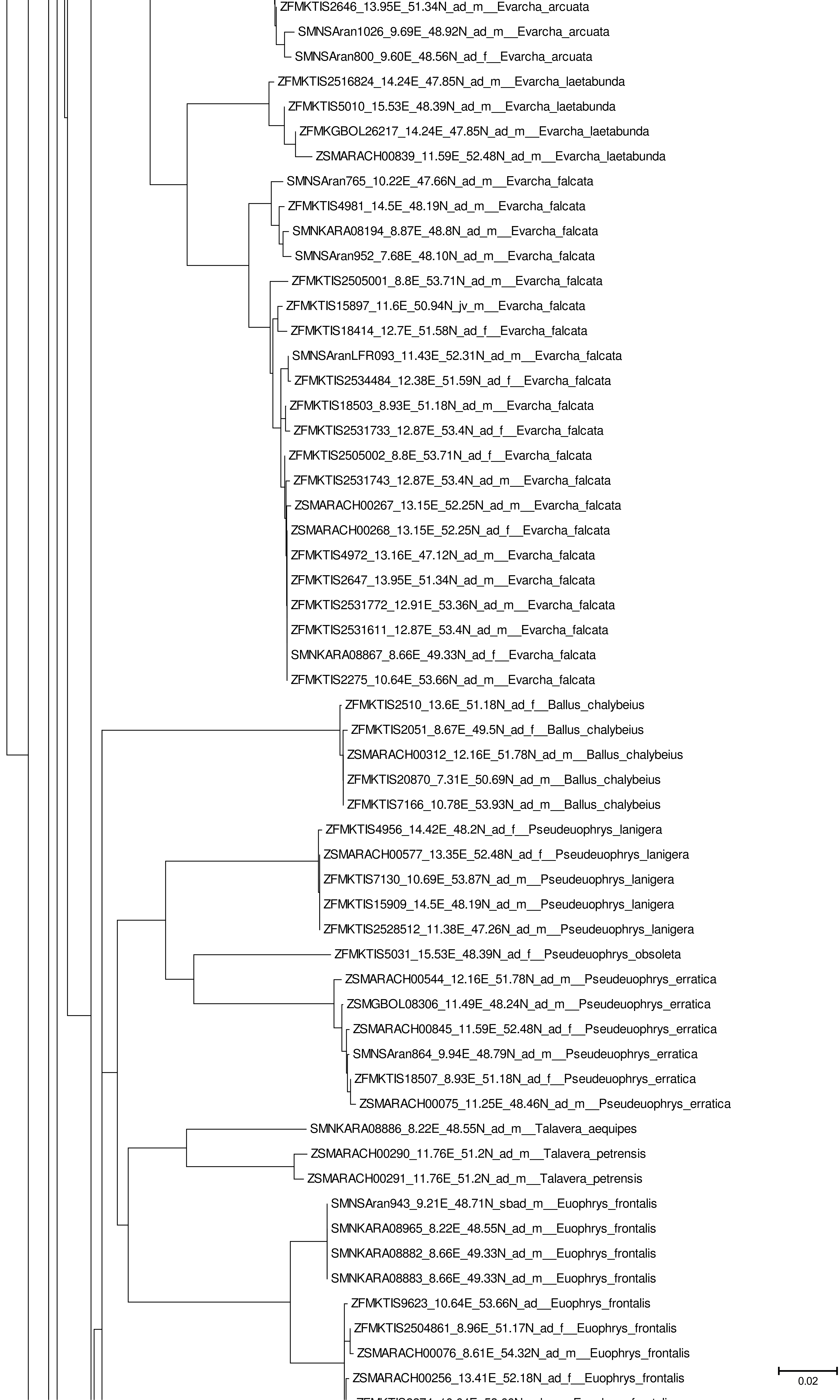

ZFMKTIS2646_13.95E_51.34N_ad_m__Evarcha_arcuata
SMNSAran1026_9.69E_48.92N_ad_m__Evarcha_arcuata
SMNSAran800_9.60E_48.56N_ad_f__Evarcha_arcuata
ZFMKTIS2516824_14.24E_47.85N_ad_m__Evarcha_laetabunda
ZFMKTIS5010_15.53E_48.39N_ad_m__Evarcha_laetabunda
ZFMKGBOL26217_14.24E_47.85N_ad_m__Evarcha_laetabunda
ZSMARACH00839_11.59E_52.48N_ad_m__Evarcha_laetabunda
SMNSAran765_10.22E_47.66N_ad_m__Evarcha_falcata
ZFMKTIS4981_14.5E_48.19N_ad_m__Evarcha_falcata
SMNKARA08194_8.87E_48.8N_ad_m__Evarcha_falcata
SMNSAran952_7.68E_48.10N_ad_m__Evarcha_falcata
ZFMKTIS2505001_8.8E_53.71N_ad_m__Evarcha_falcata
ZFMKTIS15897_11.6E_50.94N_jv_m__Evarcha_falcata
ZFMKTIS18414_12.7E_51.58N_ad_f__Evarcha_falcata
SMNSAranLFR093_11.43E_52.31N_ad_m__Evarcha_falcata
ZFMKTIS2534484_12.38E_51.59N_ad_f__Evarcha_falcata
ZFMKTIS18503_8.93E_51.18N_ad_m__Evarcha_falcata
ZFMKTIS2531733_12.87E_53.4N_ad_f__Evarcha_falcata
ZFMKTIS2505002_8.8E_53.71N_ad_f__Evarcha_falcata
ZFMKTIS2531743_12.87E_53.4N_ad_m__Evarcha_falcata
ZSMARACH00267_13.15E_52.25N_ad_m__Evarcha_falcata
ZSMARACH00268_13.15E_52.25N_ad_f__Evarcha_falcata
ZFMKTIS4972_13.16E_47.12N_ad_m__Evarcha_falcata
ZFMKTIS2647_13.95E_51.34N_ad_m__Evarcha_falcata
ZFMKTIS2531772_12.91E_53.36N_ad_m__Evarcha_falcata
ZFMKTIS2531611_12.87E_53.4N_ad_m__Evarcha_falcata
SMNKARA08867_8.66E_49.33N_ad_f__Evarcha_falcata
ZFMKTIS2275_10.64E_53.66N_ad_m__Evarcha_falcata
ZFMKTIS2510_13.6E_51.18N_ad_f__Ballus_chalybeius
ZFMKTIS2051_8.67E_49.5N_ad_f__Ballus_chalybeius
ZSMARACH00312_12.16E_51.78N_ad_m__Ballus_chalybeius
ZFMKTIS20870_7.31E_50.69N_ad_m__Ballus_chalybeius
ZFMKTIS7166_10.78E_53.93N_ad_m__Ballus_chalybeius
ZFMKTIS4956_14.42E_48.2N_ad_f__Pseudeuophrys_lanigera
ZSMARACH00577_13.35E_52.48N_ad_f__Pseudeuophrys_lanigera
ZFMKTIS7130_10.69E_53.87N_ad_m__Pseudeuophrys_lanigera
ZFMKTIS15909_14.5E_48.19N_ad_m__Pseudeuophrys_lanigera
ZFMKTIS2528512_11.38E_47.26N_ad_m__Pseudeuophrys_lanigera
ZFMKTIS5031_15.53E_48.39N_ad_f__Pseudeuophrys_obsoleta
ZSMARACH00544_12.16E_51.78N_ad_m__Pseudeuophrys_erratica
ZSMGBOL08306_11.49E_48.24N_ad_m__Pseudeuophrys_erratica
ZSMARACH00845_11.59E_52.48N_ad_f__Pseudeuophrys_erratica
SMNSAran864_9.94E_48.79N_ad_m__Pseudeuophrys_erratica
ZFMKTIS18507_8.93E_51.18N_ad_f__Pseudeuophrys_erratica
ZSMARACH00075_11.25E_48.46N_ad_m__Pseudeuophrys_erratica
SMNKARA08886_8.22E_48.55N_ad_m__Talavera_aequipes
ZSMARACH00290_11.76E_51.2N_ad_m__Talavera_petrensis
ZSMARACH00291_11.76E_51.2N_ad_m__Talavera_petrensis
SMNSAran943_9.21E_48.71N_sbad_m__Euophrys_frontalis
SMNKARA08965_8.22E_48.55N_ad_m__Euophrys_frontalis
SMNKARA08882_8.66E_49.33N_ad_m__Euophrys_frontalis
SMNKARA08883_8.66E_49.33N_ad_m__Euophrys_frontalis
ZFMKTIS9623_10.64E_53.66N_ad__Euophrys_frontalis
ZFMKTIS2504861_8.96E_51.17N_ad_f__Euophrys_frontalis
ZSMARACH00076_8.61E_54.32N_ad_m__Euophrys_frontalis
ZSMARACH00256_13.41E_52.18N_ad_f__Euophrys_frontalis
0.02

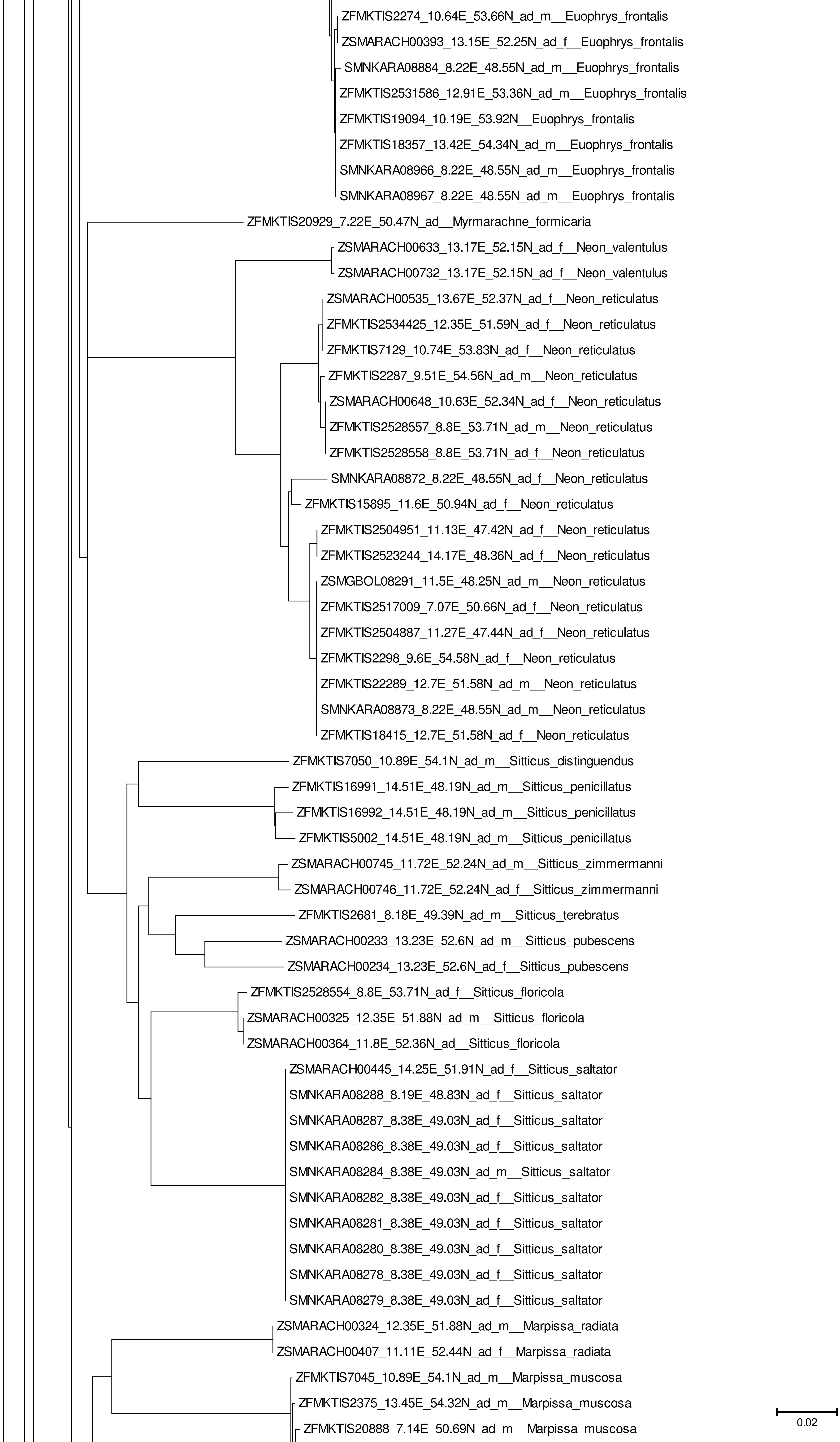
ZFMKTIS2274_10.64E_53.66N_ad_m__Euophrys_frontalis
ZSMARACH00393_13.15E_52.25N_ad_f__Euophrys_frontalis
SMNKARA08884_8.22E_48.55N_ad_m__Euophrys_frontalis
ZFMKTIS2531586_12.91E_53.36N_ad_m__Euophrys_frontalis
ZFMKTIS19094_10.19E_53.92N__Euophrys_frontalis
ZFMKTIS18357_13.42E_54.34N_ad_m__Euophrys_frontalis
SMNKARA08966_8.22E_48.55N_ad_m__Euophrys_frontalis
SMNKARA08967_8.22E_48.55N_ad_m__Euophrys_frontalis
ZFMKTIS20929_7.22E_50.47N_ad__Myrmarachne_formicaria
ZSMARACH00633_13.17E_52.15N_ad_f__Neon_valentulus
ZSMARACH00732_13.17E_52.15N_ad_f__Neon_valentulus
ZSMARACH00535_13.67E_52.37N_ad_f__Neon_reticulatus
ZFMKTIS2534425_12.35E_51.59N_ad_f__Neon_reticulatus
ZFMKTIS7129_10.74E_53.83N_ad_f__Neon_reticulatus
ZFMKTIS2287_9.51E_54.56N_ad_m__Neon_reticulatus
ZSMARACH00648_10.63E_52.34N_ad_f__Neon_reticulatus
ZFMKTIS2528557_8.8E_53.71N_ad_m__Neon_reticulatus
ZFMKTIS2528558_8.8E_53.71N_ad_f__Neon_reticulatus
SMNKARA08872_8.22E_48.55N_ad_f__Neon_reticulatus
ZFMKTIS15895_11.6E_50.94N_ad_f__Neon_reticulatus
ZFMKTIS2504951_11.13E_47.42N_ad_f__Neon_reticulatus
ZFMKTIS2523244_14.17E_48.36N_ad_f__Neon_reticulatus
ZSMGBOL08291_11.5E_48.25N_ad_m__Neon_reticulatus
ZFMKTIS2517009_7.07E_50.66N_ad_f__Neon_reticulatus
ZFMKTIS2504887_11.27E_47.44N_ad_f__Neon_reticulatus
ZFMKTIS2298_9.6E_54.58N_ad_f__Neon_reticulatus
ZFMKTIS22289_12.7E_51.58N_ad_m__Neon_reticulatus
SMNKARA08873_8.22E_48.55N_ad_m__Neon_reticulatus
ZFMKTIS18415_12.7E_51.58N_ad_f__Neon_reticulatus
ZFMKTIS7050_10.89E_54.1N_ad_m__Sitticus_distinguendus
ZFMKTIS16991_14.51E_48.19N_ad_m__Sitticus_penicillatus
ZFMKTIS16992_14.51E_48.19N_ad_m__Sitticus_penicillatus
ZFMKTIS5002_14.51E_48.19N_ad_m__Sitticus_penicillatus
ZSMARACH00745_11.72E_52.24N_ad_m__Sitticus_zimmermanni
ZSMARACH00746_11.72E_52.24N_ad_f__Sitticus_zimmermanni
ZFMKTIS2681_8.18E_49.39N_ad_m__Sitticus_terebratus
ZSMARACH00233_13.23E_52.6N_ad_m__Sitticus_pubescens
ZSMARACH00234_13.23E_52.6N_ad_f__Sitticus_pubescens
ZFMKTIS2528554_8.8E_53.71N_ad_f__Sitticus_floricola
ZSMARACH00325_12.35E_51.88N_ad_m__Sitticus_floricola
ZSMARACH00364_11.8E_52.36N_ad__Sitticus_floricola
ZSMARACH00445_14.25E_51.91N_ad_f__Sitticus_saltator
SMNKARA08288_8.19E_48.83N_ad_f__Sitticus_saltator
SMNKARA08287_8.38E_49.03N_ad_f__Sitticus_saltator
SMNKARA08286_8.38E_49.03N_ad_f__Sitticus_saltator
SMNKARA08284_8.38E_49.03N_ad_m__Sitticus_saltator
SMNKARA08282_8.38E_49.03N_ad_f__Sitticus_saltator
SMNKARA08281_8.38E_49.03N_ad_f__Sitticus_saltator
SMNKARA08280_8.38E_49.03N_ad_f__Sitticus_saltator
SMNKARA08278_8.38E_49.03N_ad_f__Sitticus_saltator
SMNKARA08279_8.38E_49.03N_ad_f__Sitticus_saltator
ZSMARACH00324_12.35E_51.88N_ad_m__Marpissa_radiata
ZSMARACH00407_11.11E_52.44N_ad_f__Marpissa_radiata
ZFMKTIS7045_10.89E_54.1N_ad_m__Marpissa_muscosa
ZFMKTIS2375_13.45E_54.32N_ad_m__Marpissa_muscosa
ZFMKTIS20888_7.14E_50.69N_ad_m__Marpissa_muscosa
0.02

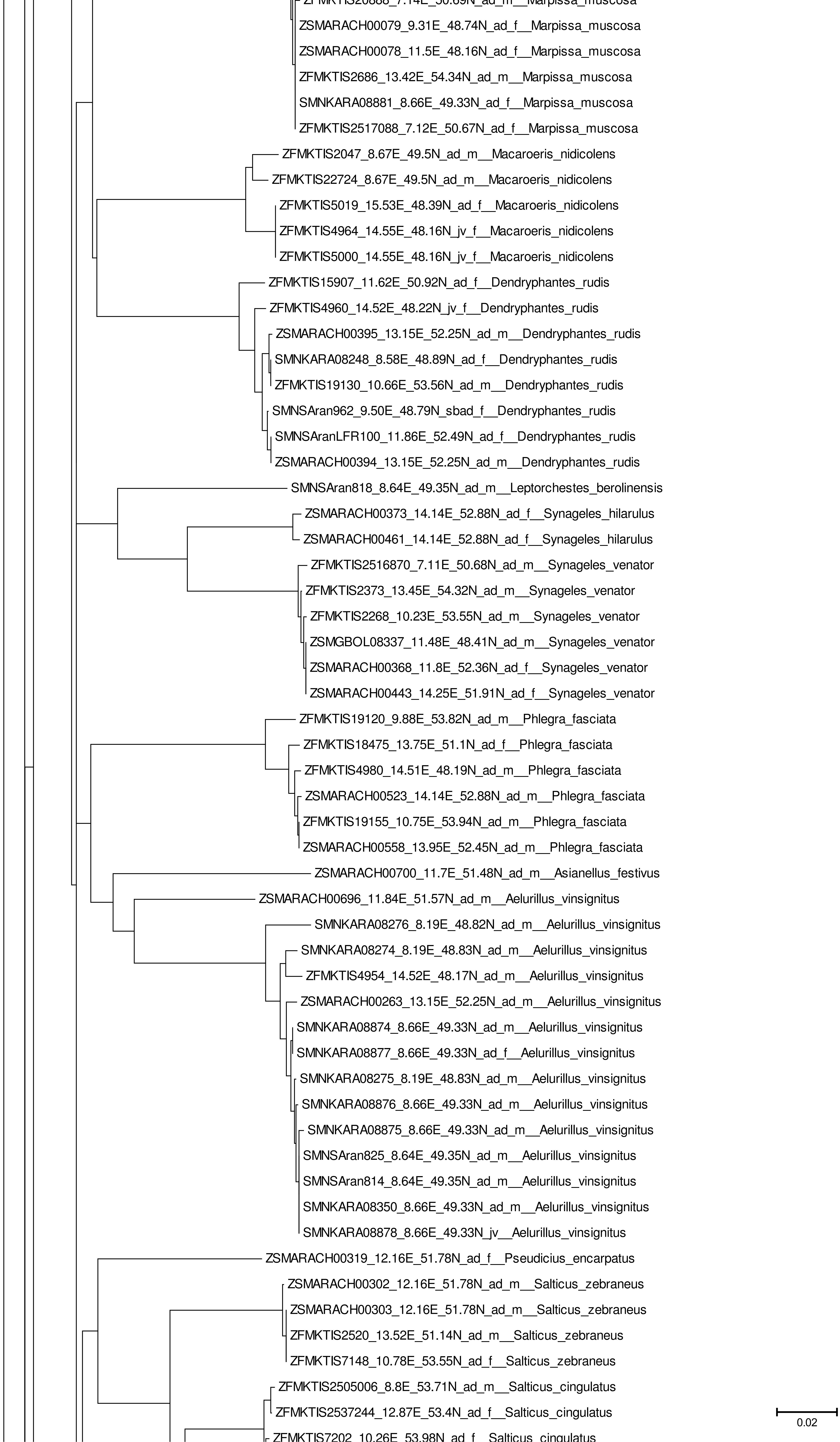
ZSMARACH00079_9.31E_48.74N_ad_f__Marpissa_muscosa
ZSMARACH00078_11.5E_48.16N_ad_f__Marpissa_muscosa
ZFMKTIS2686_13.42E_54.34N_ad_m__Marpissa_muscosa
SMNKARA08881_8.66E_49.33N_ad_f__Marpissa_muscosa
ZFMKTIS2517088_7.12E_50.67N_ad_f__Marpissa_muscosa
ZFMKTIS2047_8.67E_49.5N_ad_m__Macaroeris_nidicolens
ZFMKTIS22724_8.67E_49.5N_ad_m__Macaroeris_nidicolens
ZFMKTIS5019_15.53E_48.39N_ad_f__Macaroeris_nidicolens
ZFMKTIS4964_14.55E_48.16N_jv_f__Macaroeris_nidicolens
ZFMKTIS5000_14.55E_48.16N_jv_f__Macaroeris_nidicolens
ZFMKTIS15907_11.62E_50.92N_ad_f__Dendryphantes_rudis
ZFMKTIS4960_14.52E_48.22N_jv_f__Dendryphantes_rudis
ZSMARACH00395_13.15E_52.25N_ad_m__Dendryphantes_rudis
SMNKARA08248_8.58E_48.89N_ad_f__Dendryphantes_rudis
ZFMKTIS19130_10.66E_53.56N_ad_m__Dendryphantes_rudis
SMNSAran962_9.50E_48.79N_sbad_f__Dendryphantes_rudis
SMNSAranLFR100_11.86E_52.49N_ad_f__Dendryphantes_rudis
ZSMARACH00394_13.15E_52.25N_ad_m__Dendryphantes_rudis
SMNSAran818_8.64E_49.35N_ad_m__Leptorchestes_berolinensis
ZSMARACH00373_14.14E_52.88N_ad_f__Synageles_hilarulus
ZSMARACH00461_14.14E_52.88N_ad_f__Synageles_hilarulus
ZFMKTIS2516870_7.11E_50.68N_ad_m__Synageles_venator
ZFMKTIS2373_13.45E_54.32N_ad_m__Synageles_venator
ZFMKTIS2268_10.23E_53.55N_ad_m__Synageles_venator
ZSMGBOL08337_11.48E_48.41N_ad_m__Synageles_venator
ZSMARACH00368_11.8E_52.36N_ad_f__Synageles_venator
ZSMARACH00443_14.25E_51.91N_ad_f__Synageles_venator
ZFMKTIS19120_9.88E_53.82N_ad_m__Phlegra_fasciata
ZFMKTIS18475_13.75E_51.1N_ad_f__Phlegra_fasciata
ZFMKTIS4980_14.51E_48.19N_ad_m__Phlegra_fasciata
ZSMARACH00523_14.14E_52.88N_ad_m__Phlegra_fasciata
ZFMKTIS19155_10.75E_53.94N_ad_m__Phlegra_fasciata
ZSMARACH00558_13.95E_52.45N_ad_m__Phlegra_fasciata
ZSMARACH00700_11.7E_51.48N_ad_m__Asianellus_festivus
ZSMARACH00696_11.84E_51.57N_ad_m__Aelurillus_vinsignitus
SMNKARA08276_8.19E_48.82N_ad_m__Aelurillus_vinsignitus
SMNKARA08274_8.19E_48.83N_ad_m__Aelurillus_vinsignitus
ZFMKTIS4954_14.52E_48.17N_ad_m__Aelurillus_vinsignitus
ZSMARACH00263_13.15E_52.25N_ad_m__Aelurillus_vinsignitus
SMNKARA08874_8.66E_49.33N_ad_m__Aelurillus_vinsignitus
SMNKARA08877_8.66E_49.33N_ad_f__Aelurillus_vinsignitus
SMNKARA08275_8.19E_48.83N_ad_m__Aelurillus_vinsignitus
SMNKARA08876_8.66E_49.33N_ad_m__Aelurillus_vinsignitus
SMNKARA08875_8.66E_49.33N_ad_m__Aelurillus_vinsignitus
SMNSAran825_8.64E_49.35N_ad_m__Aelurillus_vinsignitus
SMNSAran814_8.64E_49.35N_ad_m__Aelurillus_vinsignitus
SMNKARA08350_8.66E_49.33N_ad_m__Aelurillus_vinsignitus
SMNKARA08878_8.66E_49.33N_jv__Aelurillus_vinsignitus
ZSMARACH00319_12.16E_51.78N_ad_f__Pseudicius_encarpatus
ZSMARACH00302_12.16E_51.78N_ad_m__Salticus_zebraneus
ZSMARACH00303_12.16E_51.78N_ad_m__Salticus_zebraneus
ZFMKTIS2520_13.52E_51.14N_ad_m__Salticus_zebraneus
ZFMKTIS7148_10.78E_53.55N_ad_f__Salticus_zebraneus
ZFMKTIS2505006_8.8E_53.71N_ad_m__Salticus_cingulatus
ZFMKTIS2537244_12.87E_53.4N_ad_f__Salticus_cingulatus
ZFMKTIS7202_10.26E_53.98N_ad_f__Salticus_cingulatus
0.02

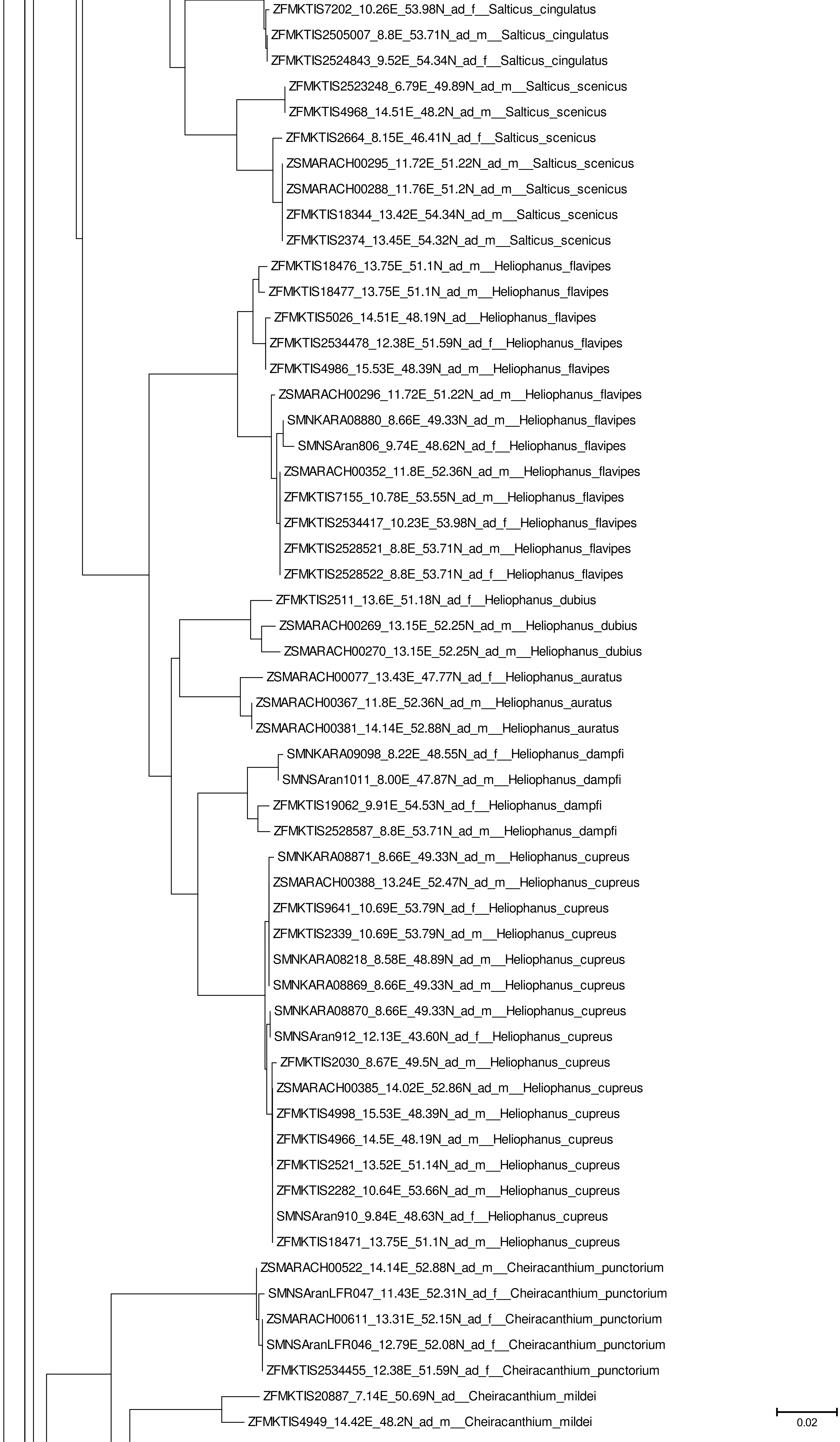
ZFMKTIS7202_10.26E_53.98N_ad_f__Salticus_cingulatus
ZFMKTIS2505007_8.8E_53.71N_ad_m__Salticus_cingulatus
ZFMKTIS2524843_9.52E_54.34N_ad_f__Salticus_cingulatus
ZFMKTIS2523248_6.79E_49.89N_ad_m__Salticus_scenicus
ZFMKTIS4968_14.51E_48.2N_ad_m__Salticus_scenicus
ZFMKTIS2664_8.15E_46.41N_ad_f__Salticus_scenicus
ZSMARACH00295_11.72E_51.22N_ad_m__Salticus_scenicus
ZSMARACH00288_11.76E_51.2N_ad_m__Salticus_scenicus
ZFMKTIS18344_13.42E_54.34N_ad_m__Salticus_scenicus
ZFMKTIS2374_13.45E_54.32N_ad_m__Salticus_scenicus
ZFMKTIS18476_13.75E_51.1N_ad_m__Heliophanus_flavipes
ZFMKTIS18477_13.75E_51.1N_ad_m__Heliophanus_flavipes
ZFMKTIS5026_14.51E_48.19N_ad__Heliophanus_flavipes
ZFMKTIS2534478_12.38E_51.59N_ad_f__Heliophanus_flavipes
ZFMKTIS4986_15.53E_48.39N_ad_m__Heliophanus_flavipes
ZSMARACH00296_11.72E_51.22N_ad_m__Heliophanus_flavipes
SMNKARA08880_8.66E_49.33N_ad_m__Heliophanus_flavipes
SMNSAran806_9.74E_48.62N_ad_f__Heliophanus_flavipes
ZSMARACH00352_11.8E_52.36N_ad_m__Heliophanus_flavipes
ZFMKTIS7155_10.78E_53.55N_ad_m__Heliophanus_flavipes
ZFMKTIS2534417_10.23E_53.98N_ad_f__Heliophanus_flavipes
ZFMKTIS2528521_8.8E_53.71N_ad_m__Heliophanus_flavipes
ZFMKTIS2528522_8.8E_53.71N_ad_f__Heliophanus_flavipes
ZFMKTIS2511_13.6E_51.18N_ad_f__Heliophanus_dubius
ZSMARACH00269_13.15E_52.25N_ad_m__Heliophanus_dubius
ZSMARACH00270_13.15E_52.25N_ad_m__Heliophanus_dubius
ZSMARACH00077_13.43E_47.77N_ad_f__Heliophanus_auratus
ZSMARACH00367_11.8E_52.36N_ad_m__Heliophanus_auratus
ZSMARACH00381_14.14E_52.88N_ad_m__Heliophanus_auratus
SMNKARA09098_8.22E_48.55N_ad_f__Heliophanus_dampfi
SMNSAran1011_8.00E_47.87N_ad_m__Heliophanus_dampfi
ZFMKTIS19062_9.91E_54.53N_ad_f__Heliophanus_dampfi
ZFMKTIS2528587_8.8E_53.71N_ad_m__Heliophanus_dampfi
SMNKARA08871_8.66E_49.33N_ad_m__Heliophanus_cupreus
ZSMARACH00388_13.24E_52.47N_ad_m__Heliophanus_cupreus
ZFMKTIS9641_10.69E_53.79N_ad_f__Heliophanus_cupreus
ZFMKTIS2339_10.69E_53.79N_ad_m__Heliophanus_cupreus
SMNKARA08218_8.58E_48.89N_ad_m__Heliophanus_cupreus
SMNKARA08869_8.66E_49.33N_ad_m__Heliophanus_cupreus
SMNKARA08870_8.66E_49.33N_ad_m__Heliophanus_cupreus
SMNSAran912_12.13E_43.60N_ad_f__Heliophanus_cupreus
ZFMKTIS2030_8.67E_49.5N_ad_m__Heliophanus_cupreus
ZSMARACH00385_14.02E_52.86N_ad_m__Heliophanus_cupreus
ZFMKTIS4998_15.53E_48.39N_ad_m__Heliophanus_cupreus
ZFMKTIS4966_14.5E_48.19N_ad_m__Heliophanus_cupreus
ZFMKTIS2521_13.52E_51.14N_ad_m__Heliophanus_cupreus
ZFMKTIS2282_10.64E_53.66N_ad_m__Heliophanus_cupreus
SMNSAran910_9.84E_48.63N_ad_f__Heliophanus_cupreus
ZFMKTIS18471_13.75E_51.1N_ad_m__Heliophanus_cupreus
ZSMARACH00522_14.14E_52.88N_ad_m__Cheiracanthium_punctorium
SMNSAranLFR047_11.43E_52.31N_ad_f__Cheiracanthium_punctorium
ZSMARACH00611_13.31E_52.15N_ad_f__Cheiracanthium_punctorium
SMNSAranLFR046_12.79E_52.08N_ad_f__Cheiracanthium_punctorium
ZFMKTIS2534455_12.38E_51.59N_ad_f__Cheiracanthium_punctorium
ZFMKTIS20887_7.14E_50.69N_ad__Cheiracanthium_mildei
ZFMKTIS4949_14.42E_48.2N_ad_m__Cheiracanthium_mildei
0.02

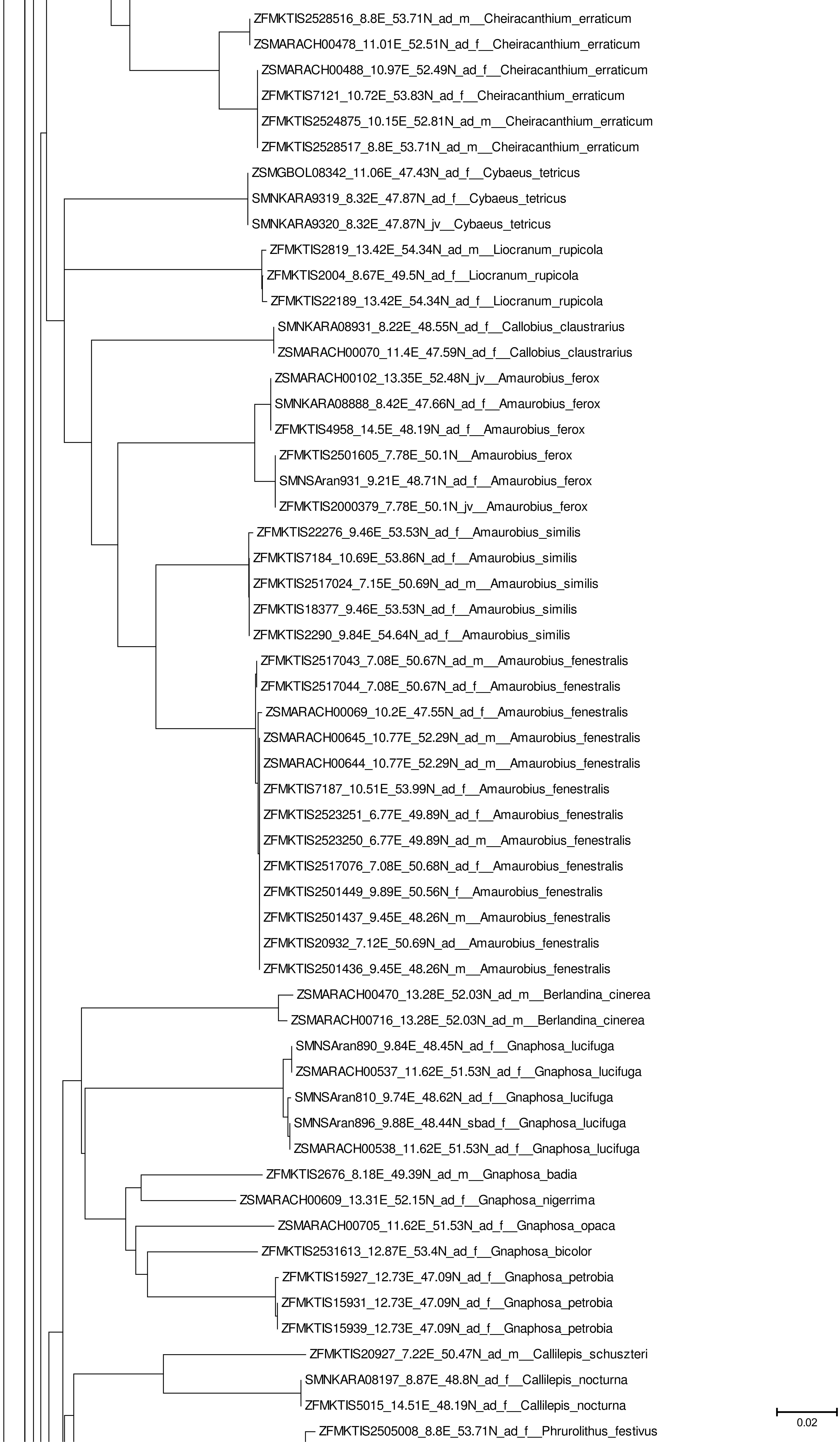
ZFMKTIS2528516_8.8E_53.71N_ad_m__Cheiracanthium_erraticum
ZSMARACH00478_11.01E_52.51N_ad_f__Cheiracanthium_erraticum
ZSMARACH00488_10.97E_52.49N_ad_f__Cheiracanthium_erraticum
ZFMKTIS7121_10.72E_53.83N_ad_f__Cheiracanthium_erraticum
ZFMKTIS2524875_10.15E_52.81N_ad_m__Cheiracanthium_erraticum
ZFMKTIS2528517_8.8E_53.71N_ad_m__Cheiracanthium_erraticum
ZSMGBOL08342_11.06E_47.43N_ad_f__Cybaeus_tetricus
SMNKARA9319_8.32E_47.87N_ad_f__Cybaeus_tetricus
SMNKARA9320_8.32E_47.87N_jv__Cybaeus_tetricus
ZFMKTIS2819_13.42E_54.34N_ad_m__Liocranum_rupicola
ZFMKTIS2004_8.67E_49.5N_ad_f__Liocranum_rupicola
ZFMKTIS22189_13.42E_54.34N_ad_f__Liocranum_rupicola
SMNKARA08931_8.22E_48.55N_ad_f__Callobius_claustrarius
ZSMARACH00070_11.4E_47.59N_ad_f__Callobius_claustrarius
ZSMARACH00102_13.35E_52.48N_jv__Amaurobius_ferox
SMNKARA08888_8.42E_47.66N_ad_f__Amaurobius_ferox
ZFMKTIS4958_14.5E_48.19N_ad_f__Amaurobius_ferox
ZFMKTIS2501605_7.78E_50.1N__Amaurobius_ferox
SMNSAran931_9.21E_48.71N_ad_f__Amaurobius_ferox
ZFMKTIS2000379_7.78E_50.1N_jv__Amaurobius_ferox
ZFMKTIS22276_9.46E_53.53N_ad_f__Amaurobius_similis
ZFMKTIS7184_10.69E_53.86N_ad_f__Amaurobius_similis
ZFMKTIS2517024_7.15E_50.69N_ad_m__Amaurobius_similis
ZFMKTIS18377_9.46E_53.53N_ad_f__Amaurobius_similis
ZFMKTIS2290_9.84E_54.64N_ad_f__Amaurobius_similis
ZFMKTIS2517043_7.08E_50.67N_ad_m__Amaurobius_fenestralis
ZFMKTIS2517044_7.08E_50.67N_ad_f__Amaurobius_fenestralis
ZSMARACH00069_10.2E_47.55N_ad_f__Amaurobius_fenestralis
ZSMARACH00645_10.77E_52.29N_ad_m__Amaurobius_fenestralis
ZSMARACH00644_10.77E_52.29N_ad_m__Amaurobius_fenestralis
ZFMKTIS7187_10.51E_53.99N_ad_f__Amaurobius_fenestralis
ZFMKTIS2523251_6.77E_49.89N_ad_f__Amaurobius_fenestralis
ZFMKTIS2523250_6.77E_49.89N_ad_m__Amaurobius_fenestralis
ZFMKTIS2517076_7.08E_50.68N_ad_f__Amaurobius_fenestralis
ZFMKTIS2501449_9.89E_50.56N_f__Amaurobius_fenestralis
ZFMKTIS2501437_9.45E_48.26N_m__Amaurobius_fenestralis
ZFMKTIS20932_7.12E_50.69N_ad__Amaurobius_fenestralis
ZFMKTIS2501436_9.45E_48.26N_m__Amaurobius_fenestralis
ZSMARACH00470_13.28E_52.03N_ad_m__Berlandina_cinerea
ZSMARACH00716_13.28E_52.03N_ad_m__Berlandina_cinerea
SMNSAran890_9.84E_48.45N_ad_f__Gnaphosa_lucifuga
ZSMARACH00537_11.62E_51.53N_ad_f__Gnaphosa_lucifuga
SMNSAran810_9.74E_48.62N_ad_f__Gnaphosa_lucifuga
SMNSAran896_9.88E_48.44N_sbad_f__Gnaphosa_lucifuga
ZSMARACH00538_11.62E_51.53N_ad_f__Gnaphosa_lucifuga
ZFMKTIS2676_8.18E_49.39N_ad_m__Gnaphosa_badia
ZSMARACH00609_13.31E_52.15N_ad_f__Gnaphosa_nigerrima
ZSMARACH00705_11.62E_51.53N_ad_f__Gnaphosa_opaca
ZFMKTIS2531613_12.87E_53.4N_ad_f__Gnaphosa_bicolor
ZFMKTIS15927_12.73E_47.09N_ad_f__Gnaphosa_petrobia
ZFMKTIS15931_12.73E_47.09N_ad_f__Gnaphosa_petrobia
ZFMKTIS15939_12.73E_47.09N_ad_f__Gnaphosa_petrobia
ZFMKTIS20927_7.22E_50.47N_ad_m__Callilepis_schuszteri
SMNKARA08197_8.87E_48.8N_ad_f__Callilepis_nocturna
ZFMKTIS5015_14.51E_48.19N_ad_f__Callilepis_nocturna
0.02
ZFMKTIS2505008_8.8E_53.71N_ad_f__Phrurolithus_festivus

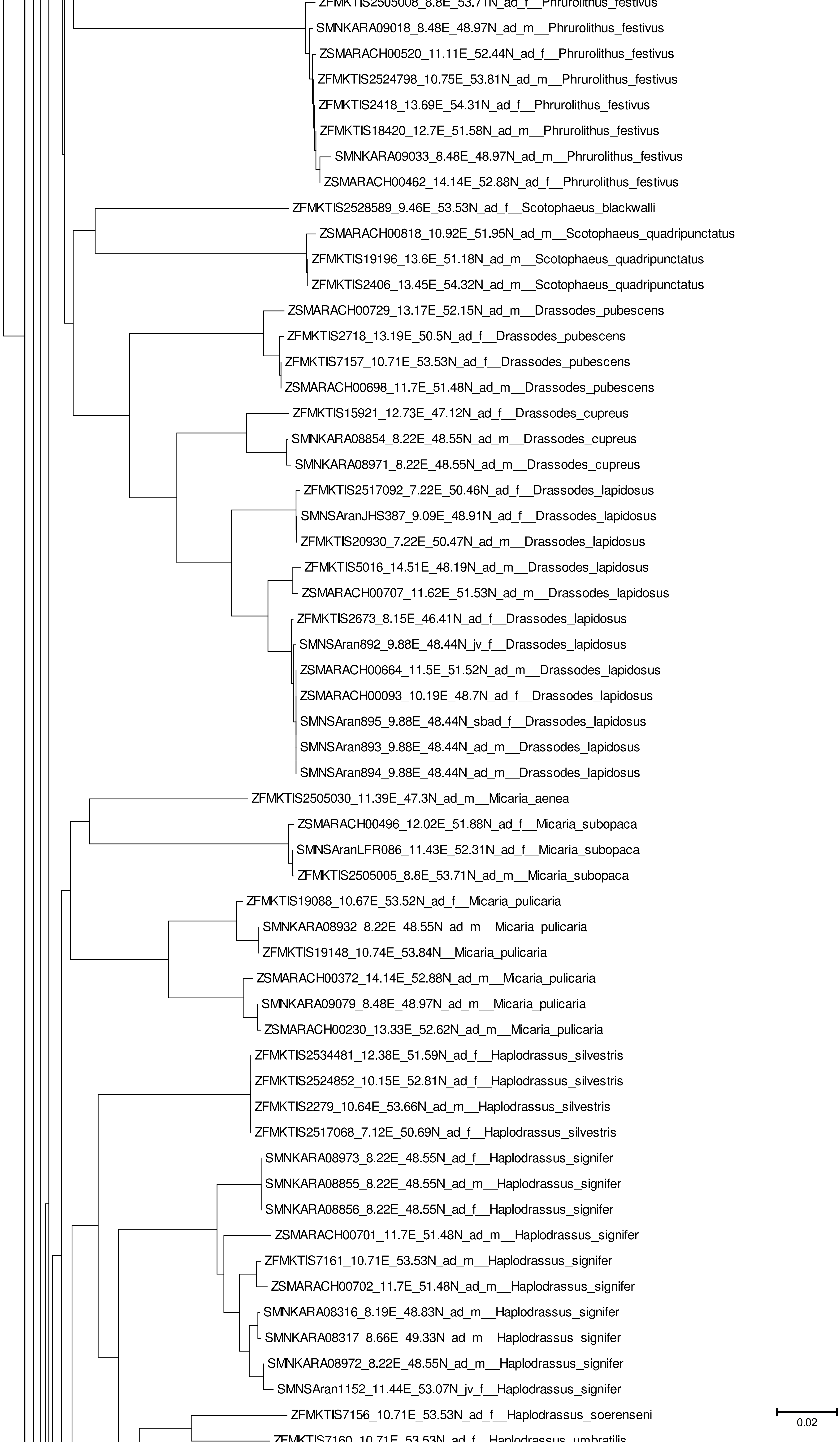
ZFMKTIS2505008_8.8E_53.71N_ad_f__Phrurolithus_festivus
SMNKARA09018_8.48E_48.97N_ad_m__Phrurolithus_festivus
ZSMARACH00520_11.11E_52.44N_ad_f__Phrurolithus_festivus
ZFMKTIS2524798_10.75E_53.81N_ad_m__Phrurolithus_festivus
ZFMKTIS2418_13.69E_54.31N_ad_f__Phrurolithus_festivus
ZFMKTIS18420_12.7E_51.58N_ad_m__Phrurolithus_festivus
SMNKARA09033_8.48E_48.97N_ad_m__Phrurolithus_festivus
ZSMARACH00462_14.14E_52.88N_ad_f__Phrurolithus_festivus
ZFMKTIS2528589_9.46E_53.53N_ad_f__Scotophaeus_blackwalli
ZSMARACH00818_10.92E_51.95N_ad_m__Scotophaeus_quadripunctatus
ZFMKTIS19196_13.6E_51.18N_ad_m__Scotophaeus_quadripunctatus
ZFMKTIS2406_13.45E_54.32N_ad_m__Scotophaeus_quadripunctatus
ZSMARACH00729_13.17E_52.15N_ad_m__Drassodes_pubescens
ZFMKTIS2718_13.19E_50.5N_ad_f__Drassodes_pubescens
ZFMKTIS7157_10.71E_53.53N_ad_f__Drassodes_pubescens
ZSMARACH00698_11.7E_51.48N_ad_m__Drassodes_pubescens
ZFMKTIS15921_12.73E_47.12N_ad_f__Drassodes_cupreus
SMNKARA08854_8.22E_48.55N_ad_m__Drassodes_cupreus
SMNKARA08971_8.22E_48.55N_ad_m__Drassodes_cupreus
ZFMKTIS2517092_7.22E_50.46N_ad_f__Drassodes_lapidosus
SMNSAranJHS387_9.09E_48.91N_ad_f__Drassodes_lapidosus
ZFMKTIS20930_7.22E_50.47N_ad_m__Drassodes_lapidosus
ZFMKTIS5016_14.51E_48.19N_ad_m__Drassodes_lapidosus
ZSMARACH00707_11.62E_51.53N_ad_m__Drassodes_lapidosus
ZFMKTIS2673_8.15E_46.41N_ad_f__Drassodes_lapidosus
SMNSAran892_9.88E_48.44N_jv_f__Drassodes_lapidosus
ZSMARACH00664_11.5E_51.52N_ad_m__Drassodes_lapidosus
ZSMARACH00093_10.19E_48.7N_ad_f__Drassodes_lapidosus
SMNSAran895_9.88E_48.44N_sbad_f__Drassodes_lapidosus
SMNSAran893_9.88E_48.44N_ad_m__Drassodes_lapidosus
SMNSAran894_9.88E_48.44N_ad_m__Drassodes_lapidosus
ZFMKTIS2505030_11.39E_47.3N_ad_m__Micaria_aenea
ZSMARACH00496_12.02E_51.88N_ad_f__Micaria_subopaca
SMNSAranLFR086_11.43E_52.31N_ad_f__Micaria_subopaca
ZFMKTIS2505005_8.8E_53.71N_ad_m__Micaria_subopaca
ZFMKTIS19088_10.67E_53.52N_ad_f__Micaria_pulicaria
SMNKARA08932_8.22E_48.55N_ad_m__Micaria_pulicaria
ZFMKTIS19148_10.74E_53.84N__Micaria_pulicaria
ZSMARACH00372_14.14E_52.88N_ad_m__Micaria_pulicaria
SMNKARA09079_8.48E_48.97N_ad_m__Micaria_pulicaria
ZSMARACH00230_13.33E_52.62N_ad_m__Micaria_pulicaria
ZFMKTIS2534481_12.38E_51.59N_ad_f__Haplodrassus_silvestris
ZFMKTIS2524852_10.15E_52.81N_ad_f__Haplodrassus_silvestris
ZFMKTIS2279_10.64E_53.66N_ad_m__Haplodrassus_silvestris
ZFMKTIS2517068_7.12E_50.69N_ad_f__Haplodrassus_silvestris
SMNKARA08973_8.22E_48.55N_ad_f__Haplodrassus_signifer
SMNKARA08855_8.22E_48.55N_ad_m__Haplodrassus_signifer
SMNKARA08856_8.22E_48.55N_ad_f__Haplodrassus_signifer
ZSMARACH00701_11.7E_51.48N_ad_m__Haplodrassus_signifer
ZFMKTIS7161_10.71E_53.53N_ad_m__Haplodrassus_signifer
ZSMARACH00702_11.7E_51.48N_ad_m__Haplodrassus_signifer
SMNKARA08316_8.19E_48.83N_ad_m__Haplodrassus_signifer
SMNKARA08317_8.66E_49.33N_ad_m__Haplodrassus_signifer
SMNKARA08972_8.22E_48.55N_ad_m__Haplodrassus_signifer
SMNSAran1152_11.44E_53.07N_jv_f__Haplodrassus_signifer
ZFMKTIS7156_10.71E_53.53N_ad_f__Haplodrassus_soerenseni
0.02

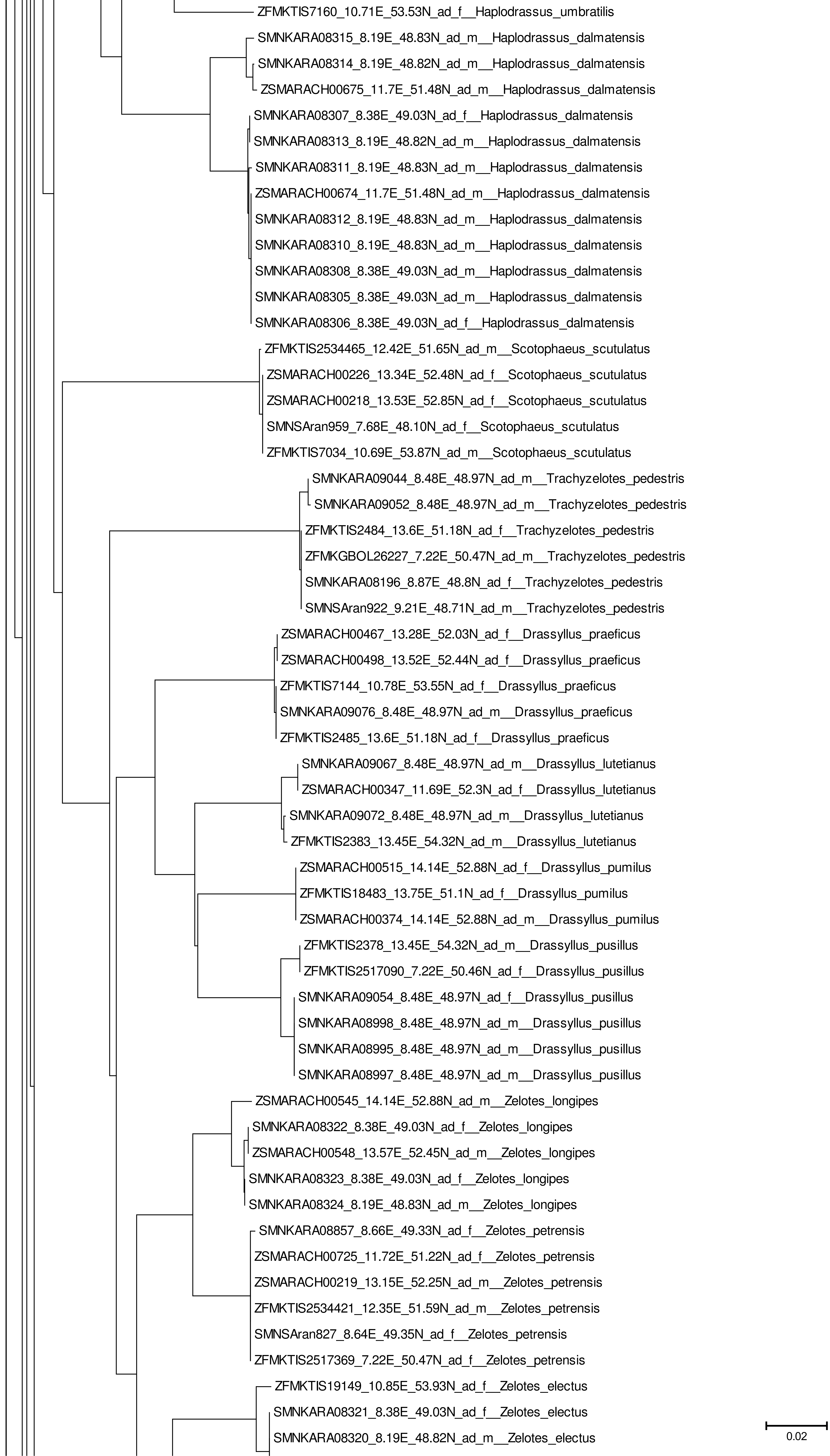
ZFMKTIS7160_10.71E_53.53N_ad_f__Haplodrassus_umbratilis
SMNKARA08315_8.19E_48.83N_ad_m__Haplodrassus_dalmatensis
SMNKARA08314_8.19E_48.82N_ad_m__Haplodrassus_dalmatensis
ZSMARACH00675_11.7E_51.48N_ad_m__Haplodrassus_dalmatensis
SMNKARA08307_8.38E_49.03N_ad_f__Haplodrassus_dalmatensis
SMNKARA08313_8.19E_48.82N_ad_m__Haplodrassus_dalmatensis
SMNKARA08311_8.19E_48.83N_ad_m__Haplodrassus_dalmatensis
ZSMARACH00674_11.7E_51.48N_ad_m__Haplodrassus_dalmatensis
SMNKARA08312_8.19E_48.83N_ad_m__Haplodrassus_dalmatensis
SMNKARA08310_8.19E_48.83N_ad_m__Haplodrassus_dalmatensis
SMNKARA08308_8.38E_49.03N_ad_m__Haplodrassus_dalmatensis
SMNKARA08305_8.38E_49.03N_ad_m__Haplodrassus_dalmatensis
SMNKARA08306_8.38E_49.03N_ad_f__Haplodrassus_dalmatensis
ZFMKTIS2534465_12.42E_51.65N_ad_m__Scotophaeus_scutulatus
ZSMARACH00226_13.34E_52.48N_ad_f__Scotophaeus_scutulatus
ZSMARACH00218_13.53E_52.85N_ad_f__Scotophaeus_scutulatus
SMNSAran959_7.68E_48.10N_ad_f__Scotophaeus_scutulatus
ZFMKTIS7034_10.69E_53.87N_ad_m__Scotophaeus_scutulatus
SMNKARA09044_8.48E_48.97N_ad_m__Trachyzelotes_pedestris
SMNKARA09052_8.48E_48.97N_ad_m__Trachyzelotes_pedestris
ZFMKTIS2484_13.6E_51.18N_ad_f__Trachyzelotes_pedestris
ZFMKGBOL26227_7.22E_50.47N_ad_m__Trachyzelotes_pedestris
SMNKARA08196_8.87E_48.8N_ad_f__Trachyzelotes_pedestris
SMNSAran922_9.21E_48.71N_ad_m__Trachyzelotes_pedestris
ZSMARACH00467_13.28E_52.03N_ad_f__Drassyllus_praeficus
ZSMARACH00498_13.52E_52.44N_ad_f__Drassyllus_praeficus
ZFMKTIS7144_10.78E_53.55N_ad_f__Drassyllus_praeficus
SMNKARA09076_8.48E_48.97N_ad_m__Drassyllus_praeficus
ZFMKTIS2485_13.6E_51.18N_ad_f__Drassyllus_praeficus
SMNKARA09067_8.48E_48.97N_ad_m__Drassyllus_lutetianus
ZSMARACH00347_11.69E_52.3N_ad_f__Drassyllus_lutetianus
SMNKARA09072_8.48E_48.97N_ad_m__Drassyllus_lutetianus
ZFMKTIS2383_13.45E_54.32N_ad_m__Drassyllus_lutetianus
ZSMARACH00515_14.14E_52.88N_ad_f__Drassyllus_pumilus
ZFMKTIS18483_13.75E_51.1N_ad_f__Drassyllus_pumilus
ZSMARACH00374_14.14E_52.88N_ad_m__Drassyllus_pumilus
ZFMKTIS2378_13.45E_54.32N_ad_m__Drassyllus_pusillus
ZFMKTIS2517090_7.22E_50.46N_ad_f__Drassyllus_pusillus
SMNKARA09054_8.48E_48.97N_ad_f__Drassyllus_pusillus
SMNKARA08998_8.48E_48.97N_ad_m__Drassyllus_pusillus
SMNKARA08995_8.48E_48.97N_ad_m__Drassyllus_pusillus
SMNKARA08997_8.48E_48.97N_ad_m__Drassyllus_pusillus
ZSMARACH00545_14.14E_52.88N_ad_m__Zelotes_longipes
SMNKARA08322_8.38E_49.03N_ad_f__Zelotes_longipes
ZSMARACH00548_13.57E_52.45N_ad_m__Zelotes_longipes
SMNKARA08323_8.38E_49.03N_ad_f__Zelotes_longipes
SMNKARA08324_8.19E_48.83N_ad_m__Zelotes_longipes
SMNKARA08857_8.66E_49.33N_ad_f__Zelotes_petrensis
ZSMARACH00725_11.72E_51.22N_ad_f__Zelotes_petrensis
ZSMARACH00219_13.15E_52.25N_ad_m__Zelotes_petrensis
ZFMKTIS2534421_12.35E_51.59N_ad_m__Zelotes_petrensis
SMNSAran827_8.64E_49.35N_ad_f__Zelotes_petrensis
ZFMKTIS2517369_7.22E_50.47N_ad_f__Zelotes_petrensis
ZFMKTIS19149_10.85E_53.93N_ad_f__Zelotes_electus
SMNKARA08321_8.38E_49.03N_ad_f__Zelotes_electus
SMNKARA08320_8.19E_48.82N_ad_m__Zelotes_electus
0.02

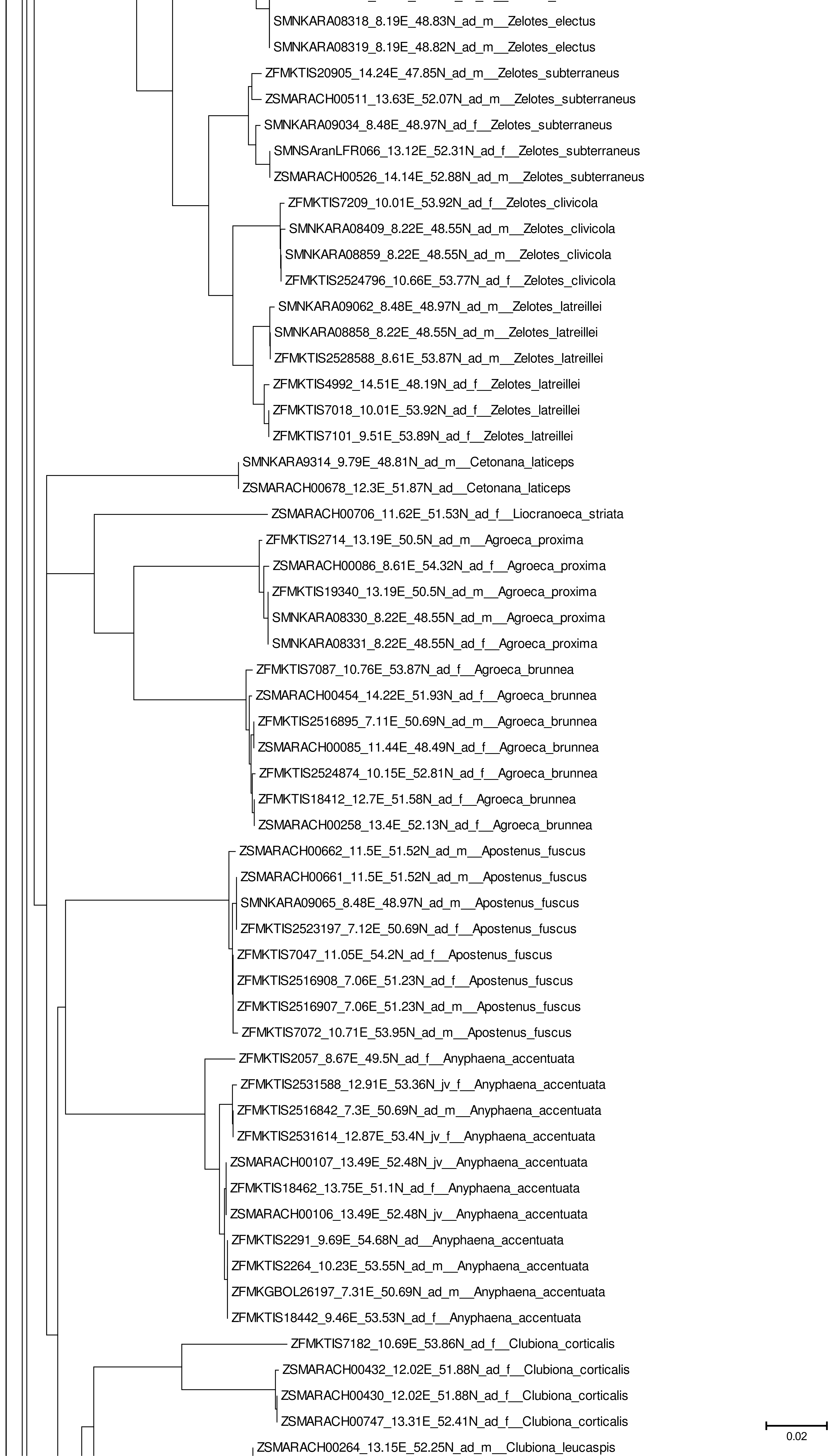

SMNKARA08318_8.19E_48.83N_ad_m__Zelotes_electus
SMNKARA08319_8.19E_48.82N_ad_m__Zelotes_electus
ZFMKTIS20905_14.24E_47.85N_ad_m__Zelotes_subterraneus
ZSMARACH00511_13.63E_52.07N_ad_m__Zelotes_subterraneus
SMNKARA09034_8.48E_48.97N_ad_f__Zelotes_subterraneus
SMNSAranLFR066_13.12E_52.31N_ad_f__Zelotes_subterraneus
ZSMARACH00526_14.14E_52.88N_ad_m__Zelotes_subterraneus
ZFMKTIS7209_10.01E_53.92N_ad_f__Zelotes_clivicola
SMNKARA08409_8.22E_48.55N_ad_m__Zelotes_clivicola
SMNKARA08859_8.22E_48.55N_ad_m__Zelotes_clivicola
ZFMKTIS2524796_10.66E_53.77N_ad_f__Zelotes_clivicola
SMNKARA09062_8.48E_48.97N_ad_m__Zelotes_latreillei
SMNKARA08858_8.22E_48.55N_ad_m__Zelotes_latreillei
ZFMKTIS2528588_8.61E_53.87N_ad_m__Zelotes_latreillei
ZFMKTIS4992_14.51E_48.19N_ad_f__Zelotes_latreillei
ZFMKTIS7018_10.01E_53.92N_ad_f__Zelotes_latreillei
ZFMKTIS7101_9.51E_53.89N_ad_f__Zelotes_latreillei
SMNKARA9314_9.79E_48.81N_ad_m__Cetonana_laticeps
ZSMARACH00678_12.3E_51.87N_ad__Cetonana_laticeps
ZSMARACH00706_11.62E_51.53N_ad_f__Liocranoeca_striata
ZFMKTIS2714_13.19E_50.5N_ad_m__Agroeca_proxima
ZSMARACH00086_8.61E_54.32N_ad_f__Agroeca_proxima
ZFMKTIS19340_13.19E_50.5N_ad_m__Agroeca_proxima
SMNKARA08330_8.22E_48.55N_ad_m__Agroeca_proxima
SMNKARA08331_8.22E_48.55N_ad_f__Agroeca_proxima
ZFMKTIS7087_10.76E_53.87N_ad_f__Agroeca_brunnea
ZSMARACH00454_14.22E_51.93N_ad_f__Agroeca_brunnea
ZFMKTIS2516895_7.11E_50.69N_ad_m__Agroeca_brunnea
ZSMARACH00085_11.44E_48.49N_ad_f__Agroeca_brunnea
ZFMKTIS2524874_10.15E_52.81N_ad_f__Agroeca_brunnea
ZFMKTIS18412_12.7E_51.58N_ad_f__Agroeca_brunnea
ZSMARACH00258_13.4E_52.13N_ad_f__Agroeca_brunnea
ZSMARACH00662_11.5E_51.52N_ad_m__Apostenus_fuscus
ZSMARACH00661_11.5E_51.52N_ad_m__Apostenus_fuscus
SMNKARA09065_8.48E_48.97N_ad_m__Apostenus_fuscus
ZFMKTIS2523197_7.12E_50.69N_ad_f__Apostenus_fuscus
ZFMKTIS7047_11.05E_54.2N_ad_f__Apostenus_fuscus
ZFMKTIS2516908_7.06E_51.23N_ad_f__Apostenus_fuscus
ZFMKTIS2516907_7.06E_51.23N_ad_m__Apostenus_fuscus
ZFMKTIS7072_10.71E_53.95N_ad_m__Apostenus_fuscus
ZFMKTIS2057_8.67E_49.5N_ad_f__Anyphaena_accentuata
ZFMKTIS2531588_12.91E_53.36N_jv_f__Anyphaena_accentuata
ZFMKTIS2516842_7.3E_50.69N_ad_m__Anyphaena_accentuata
ZFMKTIS2531614_12.87E_53.4N_jv_f__Anyphaena_accentuata
ZSMARACH00107_13.49E_52.48N_jv__Anyphaena_accentuata
ZFMKTIS18462_13.75E_51.1N_ad_f__Anyphaena_accentuata
ZSMARACH00106_13.49E_52.48N_jv__Anyphaena_accentuata
ZFMKTIS2291_9.69E_54.68N_ad__Anyphaena_accentuata
ZFMKTIS2264_10.23E_53.55N_ad_m__Anyphaena_accentuata
ZFMKGBOL26197_7.31E_50.69N_ad_m__Anyphaena_accentuata
ZFMKTIS18442_9.46E_53.53N_ad_f__Anyphaena_accentuata
ZFMKTIS7182_10.69E_53.86N_ad_f__Clubiona_corticalis
ZSMARACH00432_12.02E_51.88N_ad_f__Clubiona_corticalis
ZSMARACH00430_12.02E_51.88N_ad_f__Clubiona_corticalis
ZSMARACH00747_13.31E_52.41N_ad_f__Clubiona_corticalis
ZSMARACH00264_13.15E_52.25N_ad_m__Clubiona_leucaspis
0.02

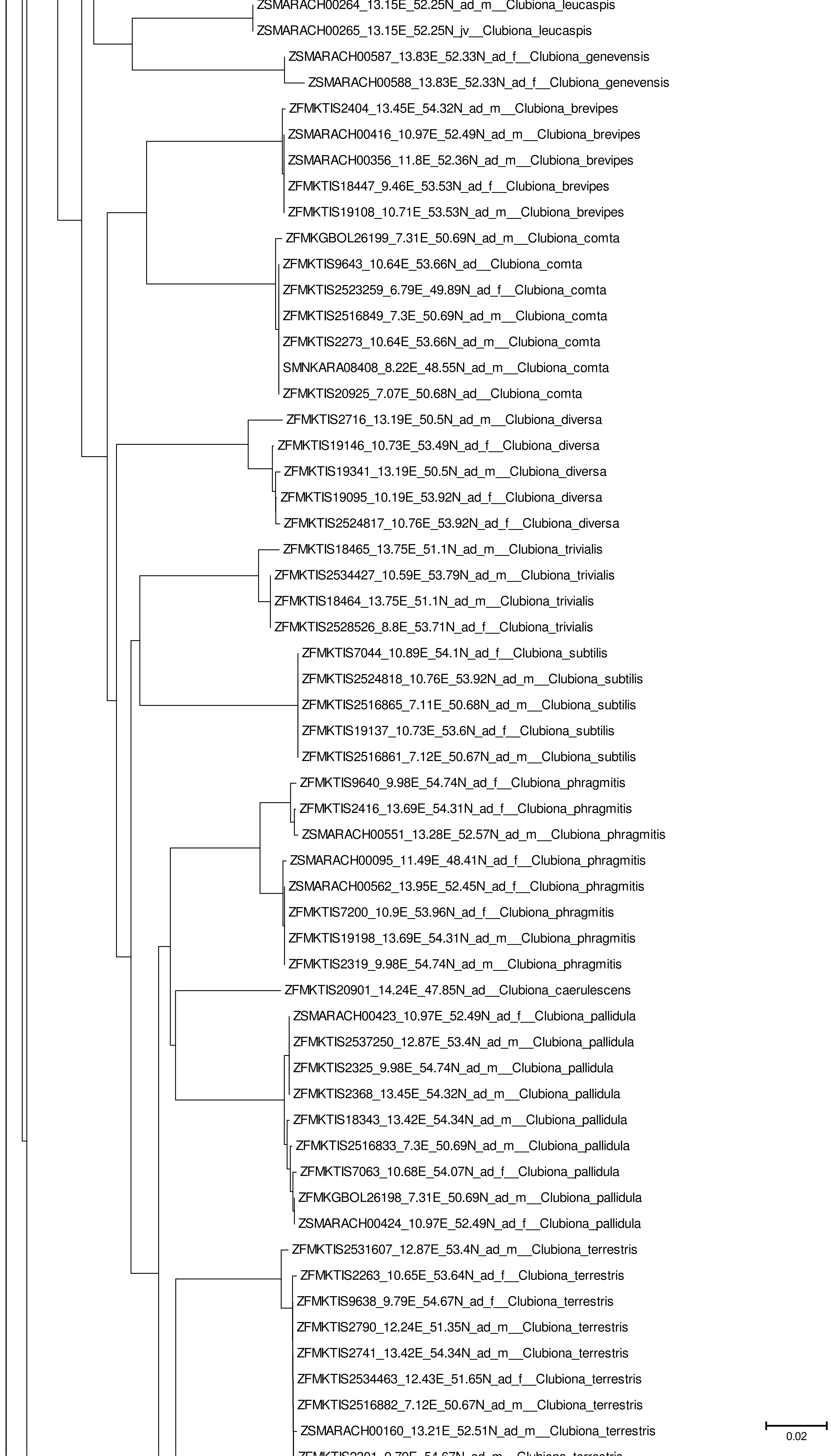
ZSMARACH00264_13.15E_52.25N_ad_m__Clubiona_leucaspis
ZSMARACH00265_13.15E_52.25N_jv__Clubiona_leucaspis
ZSMARACH00587_13.83E_52.33N_ad_f__Clubiona_genevensis
ZSMARACH00588_13.83E_52.33N_ad_f__Clubiona_genevensis
ZFMKTIS2404_13.45E_54.32N_ad_m__Clubiona_brevipes
ZSMARACH00416_10.97E_52.49N_ad_m__Clubiona_brevipes
ZSMARACH00356_11.8E_52.36N_ad_m__Clubiona_brevipes
ZFMKTIS18447_9.46E_53.53N_ad_f__Clubiona_brevipes
ZFMKTIS19108_10.71E_53.53N_ad_m__Clubiona_brevipes
ZFMKGBOL26199_7.31E_50.69N_ad_m__Clubiona_comta
ZFMKTIS9643_10.64E_53.66N_ad__Clubiona_comta
ZFMKTIS2523259_6.79E_49.89N_ad_f__Clubiona_comta
ZFMKTIS2516849_7.3E_50.69N_ad_m__Clubiona_comta
ZFMKTIS2273_10.64E_53.66N_ad_m__Clubiona_comta
SMNKARA08408_8.22E_48.55N_ad_m__Clubiona_comta
ZFMKTIS20925_7.07E_50.68N_ad__Clubiona_comta
ZFMKTIS2716_13.19E_50.5N_ad_m__Clubiona_diversa
ZFMKTIS19146_10.73E_53.49N_ad_f__Clubiona_diversa
ZFMKTIS19341_13.19E_50.5N_ad_m__Clubiona_diversa
ZFMKTIS19095_10.19E_53.92N_ad_f__Clubiona_diversa
ZFMKTIS2524817_10.76E_53.92N_ad_f__Clubiona_diversa
ZFMKTIS18465_13.75E_51.1N_ad_m__Clubiona_trivialis
ZFMKTIS2534427_10.59E_53.79N_ad_m__Clubiona_trivialis
ZFMKTIS18464_13.75E_51.1N_ad_m__Clubiona_trivialis
ZFMKTIS2528526_8.8E_53.71N_ad_f__Clubiona_trivialis
ZFMKTIS7044_10.89E_54.1N_ad_f__Clubiona_subtilis
ZFMKTIS2524818_10.76E_53.92N_ad_m__Clubiona_subtilis
ZFMKTIS2516865_7.11E_50.68N_ad_m__Clubiona_subtilis
ZFMKTIS19137_10.73E_53.6N_ad_f__Clubiona_subtilis
ZFMKTIS2516861_7.12E_50.67N_ad_m__Clubiona_subtilis
ZFMKTIS9640_9.98E_54.74N_ad_f__Clubiona_phragmitis
ZFMKTIS2416_13.69E_54.31N_ad_f__Clubiona_phragmitis
ZSMARACH00551_13.28E_52.57N_ad_m__Clubiona_phragmitis
ZSMARACH00095_11.49E_48.41N_ad_f__Clubiona_phragmitis
ZSMARACH00562_13.95E_52.45N_ad_f__Clubiona_phragmitis
ZFMKTIS7200_10.9E_53.96N_ad_f__Clubiona_phragmitis
ZFMKTIS19198_13.69E_54.31N_ad_m__Clubiona_phragmitis
ZFMKTIS2319_9.98E_54.74N_ad_m__Clubiona_phragmitis
ZFMKTIS20901_14.24E_47.85N_ad__Clubiona_caerulescens
ZSMARACH00423_10.97E_52.49N_ad_f__Clubiona_pallidula
ZFMKTIS2537250_12.87E_53.4N_ad_m__Clubiona_pallidula
ZFMKTIS2325_9.98E_54.74N_ad_m__Clubiona_pallidula
ZFMKTIS2368_13.45E_54.32N_ad_m__Clubiona_pallidula
ZFMKTIS18343_13.42E_54.34N_ad_m__Clubiona_pallidula
ZFMKTIS2516833_7.3E_50.69N_ad_m__Clubiona_pallidula
ZFMKTIS7063_10.68E_54.07N_ad_f__Clubiona_pallidula
ZFMKGBOL26198_7.31E_50.69N_ad_m__Clubiona_pallidula
ZSMARACH00424_10.97E_52.49N_ad_f__Clubiona_pallidula
ZFMKTIS2531607_12.87E_53.4N_ad_m__Clubiona_terrestris
ZFMKTIS2263_10.65E_53.64N_ad_f__Clubiona_terrestris
ZFMKTIS9638_9.79E_54.67N_ad_f__Clubiona_terrestris
ZFMKTIS2790_12.24E_51.35N_ad_m__Clubiona_terrestris
ZFMKTIS2741_13.42E_54.34N_ad_m__Clubiona_terrestris
ZFMKTIS2534463_12.43E_51.65N_ad_f__Clubiona_terrestris
ZFMKTIS2516882_7.12E_50.67N_ad_m__Clubiona_terrestris
ZSMARACH00160_13.21E_52.51N_ad_m__Clubiona_terrestris
0.02

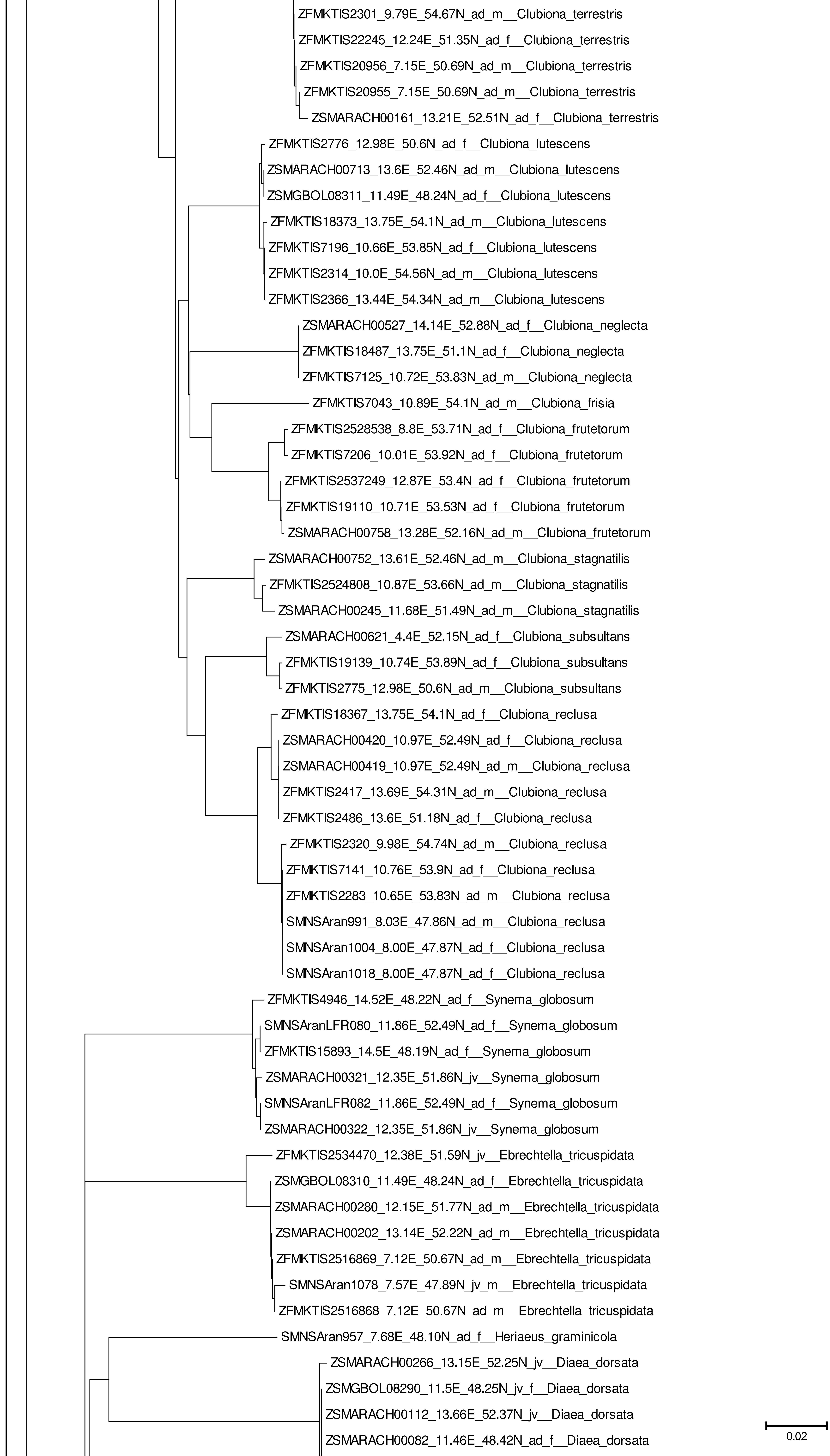
ZFMKTIS2301_9.79E_54.67N_ad_m__Clubiona_terrestris
ZFMKTIS22245_12.24E_51.35N_ad_f__Clubiona_terrestris
ZFMKTIS20956_7.15E_50.69N_ad_m__Clubiona_terrestris
ZFMKTIS20955_7.15E_50.69N_ad_m__Clubiona_terrestris
ZSMARACH00161_13.21E_52.51N_ad_f__Clubiona_terrestris
ZFMKTIS2776_12.98E_50.6N_ad_f__Clubiona_lutescens
ZSMARACH00713_13.6E_52.46N_ad_m__Clubiona_lutescens
ZSMGBOL08311_11.49E_48.24N_ad_f__Clubiona_lutescens
ZFMKTIS18373_13.75E_54.1N_ad_m__Clubiona_lutescens
ZFMKTIS7196_10.66E_53.85N_ad_f__Clubiona_lutescens
ZFMKTIS2314_10.0E_54.56N_ad_m__Clubiona_lutescens
ZFMKTIS2366_13.44E_54.34N_ad_m__Clubiona_lutescens
ZSMARACH00527_14.14E_52.88N_ad_f__Clubiona_neglecta
ZFMKTIS18487_13.75E_51.1N_ad_f__Clubiona_neglecta
ZFMKTIS7125_10.72E_53.83N_ad_m__Clubiona_neglecta
ZFMKTIS7043_10.89E_54.1N_ad_m__Clubiona_frisia
ZFMKTIS2528538_8.8E_53.71N_ad_f__Clubiona_frutetorum
ZFMKTIS7206_10.01E_53.92N_ad_f__Clubiona_frutetorum
ZFMKTIS2537249_12.87E_53.4N_ad_f__Clubiona_frutetorum
ZFMKTIS19110_10.71E_53.53N_ad_f__Clubiona_frutetorum
ZSMARACH00758_13.28E_52.16N_ad_m__Clubiona_frutetorum
ZSMARACH00752_13.61E_52.46N_ad_m__Clubiona_stagnatilis
ZFMKTIS2524808_10.87E_53.66N_ad_m__Clubiona_stagnatilis
ZSMARACH00245_11.68E_51.49N_ad_m__Clubiona_stagnatilis
ZSMARACH00621_4.4E_52.15N_ad_f__Clubiona_subsultans
ZFMKTIS19139_10.74E_53.89N_ad_f__Clubiona_subsultans
ZFMKTIS2775_12.98E_50.6N_ad_m__Clubiona_subsultans
ZFMKTIS18367_13.75E_54.1N_ad_f__Clubiona_reclusa
ZSMARACH00420_10.97E_52.49N_ad_f__Clubiona_reclusa
ZSMARACH00419_10.97E_52.49N_ad_m__Clubiona_reclusa
ZFMKTIS2417_13.69E_54.31N_ad_m__Clubiona_reclusa
ZFMKTIS2486_13.6E_51.18N_ad_f__Clubiona_reclusa
ZFMKTIS2320_9.98E_54.74N_ad_m__Clubiona_reclusa
ZFMKTIS7141_10.76E_53.9N_ad_f__Clubiona_reclusa
ZFMKTIS2283_10.65E_53.83N_ad_m__Clubiona_reclusa
SMNSAran991_8.03E_47.86N_ad_m__Clubiona_reclusa
SMNSAran1004_8.00E_47.87N_ad_f__Clubiona_reclusa
SMNSAran1018_8.00E_47.87N_ad_f__Clubiona_reclusa
ZFMKTIS4946_14.52E_48.22N_ad_f__Synema_globosum
SMNSAranLFR080_11.86E_52.49N_ad_f__Synema_globosum
ZFMKTIS15893_14.5E_48.19N_ad_f__Synema_globosum
ZSMARACH00321_12.35E_51.86N_jv__Synema_globosum
SMNSAranLFR082_11.86E_52.49N_ad_f__Synema_globosum
ZSMARACH00322_12.35E_51.86N_jv__Synema_globosum
ZFMKTIS2534470_12.38E_51.59N_jv__Ebrechtella_tricuspidata
ZSMGBOL08310_11.49E_48.24N_ad_f__Ebrechtella_tricuspidata
ZSMARACH00280_12.15E_51.77N_ad_m__Ebrechtella_tricuspidata
ZSMARACH00202_13.14E_52.22N_ad_m__Ebrechtella_tricuspidata
ZFMKTIS2516869_7.12E_50.67N_ad_m__Ebrechtella_tricuspidata
SMNSAran1078_7.57E_47.89N_jv_m__Ebrechtella_tricuspidata
ZFMKTIS2516868_7.12E_50.67N_ad_m__Ebrechtella_tricuspidata
SMNSAran957_7.68E_48.10N_ad_f__Heriaeus_graminicola
ZSMARACH00266_13.15E_52.25N_jv__Diaea_dorsata
ZSMGBOL08290_11.5E_48.25N_jv_f__Diaea_dorsata
ZSMARACH00112_13.66E_52.37N_jv__Diaea_dorsata
ZSMARACH00082_11.46E_48.42N_ad_f__Diaea_dorsata
0.02

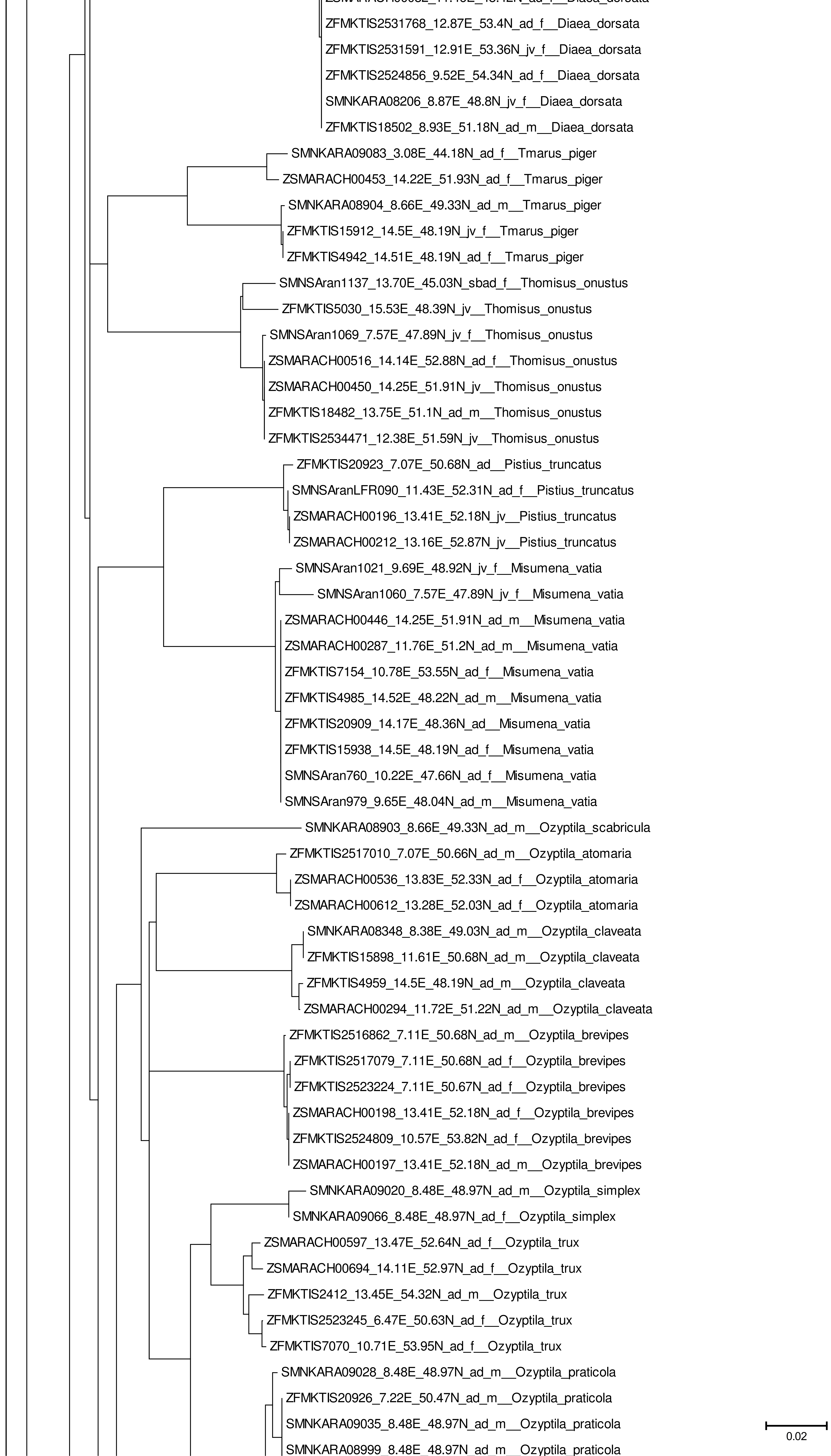
ZFMKTIS2531768_12.87E_53.4N_ad_f__Diaea_dorsata
ZFMKTIS2531591_12.91E_53.36N_jv_f__Diaea_dorsata
ZFMKTIS2524856_9.52E_54.34N_ad_f__Diaea_dorsata
SMNKARA08206_8.87E_48.8N_jv_f__Diaea_dorsata
ZFMKTIS18502_8.93E_51.18N_ad_m__Diaea_dorsata
SMNKARA09083_3.08E_44.18N_ad_f__Tmarus_piger
ZSMARACH00453_14.22E_51.93N_ad_f__Tmarus_piger
SMNKARA08904_8.66E_49.33N_ad_m__Tmarus_piger
ZFMKTIS15912_14.5E_48.19N_jv_f__Tmarus_piger
ZFMKTIS4942_14.51E_48.19N_ad_f__Tmarus_piger
SMNSAran1137_13.70E_45.03N_sbad_f__Thomisus_onustus
ZFMKTIS5030_15.53E_48.39N_jv__Thomisus_onustus
SMNSAran1069_7.57E_47.89N_jv_f__Thomisus_onustus
ZSMARACH00516_14.14E_52.88N_ad_f__Thomisus_onustus
ZSMARACH00450_14.25E_51.91N_jv__Thomisus_onustus
ZFMKTIS18482_13.75E_51.1N_ad_m__Thomisus_onustus
ZFMKTIS2534471_12.38E_51.59N_jv__Thomisus_onustus
ZFMKTIS20923_7.07E_50.68N_ad__Pistius_truncatus
SMNSAranLFR090_11.43E_52.31N_ad_f__Pistius_truncatus
ZSMARACH00196_13.41E_52.18N_jv__Pistius_truncatus
ZSMARACH00212_13.16E_52.87N_jv__Pistius_truncatus
SMNSAran1021_9.69E_48.92N_jv_f__Misumena_vatia
SMNSAran1060_7.57E_47.89N_jv_f__Misumena_vatia
ZSMARACH00446_14.25E_51.91N_ad_m__Misumena_vatia
ZSMARACH00287_11.76E_51.2N_ad_m__Misumena_vatia
ZFMKTIS7154_10.78E_53.55N_ad_f__Misumena_vatia
ZFMKTIS4985_14.52E_48.22N_ad_m__Misumena_vatia
ZFMKTIS20909_14.17E_48.36N_ad__Misumena_vatia
ZFMKTIS15938_14.5E_48.19N_ad_f__Misumena_vatia
SMNSAran760_10.22E_47.66N_ad_f__Misumena_vatia
SMNSAran979_9.65E_48.04N_ad_m__Misumena_vatia
SMNKARA08903_8.66E_49.33N_ad_m__Ozyptila_scabricula
ZFMKTIS2517010_7.07E_50.66N_ad_m__Ozyptila_atomaria
ZSMARACH00536_13.83E_52.33N_ad_f__Ozyptila_atomaria
ZSMARACH00612_13.28E_52.03N_ad_f__Ozyptila_atomaria
SMNKARA08348_8.38E_49.03N_ad_m__Ozyptila_claveata
ZFMKTIS15898_11.61E_50.68N_ad_m__Ozyptila_claveata
ZFMKTIS4959_14.5E_48.19N_ad_m__Ozyptila_claveata
ZSMARACH00294_11.72E_51.22N_ad_m__Ozyptila_claveata
ZFMKTIS2516862_7.11E_50.68N_ad_m__Ozyptila_brevipes
ZFMKTIS2517079_7.11E_50.68N_ad_f__Ozyptila_brevipes
ZFMKTIS2523224_7.11E_50.67N_ad_f__Ozyptila_brevipes
ZSMARACH00198_13.41E_52.18N_ad_f__Ozyptila_brevipes
ZFMKTIS2524809_10.57E_53.82N_ad_f__Ozyptila_brevipes
ZSMARACH00197_13.41E_52.18N_ad_m__Ozyptila_brevipes
SMNKARA09020_8.48E_48.97N_ad_m__Ozyptila_simplex
SMNKARA09066_8.48E_48.97N_ad_f__Ozyptila_simplex
ZSMARACH00597_13.47E_52.64N_ad_f__Ozyptila_trux
ZSMARACH00694_14.11E_52.97N_ad_f__Ozyptila_trux
ZFMKTIS2412_13.45E_54.32N_ad_m__Ozyptila_trux
ZFMKTIS2523245_6.47E_50.63N_ad_f__Ozyptila_trux
ZFMKTIS7070_10.71E_53.95N_ad_f__Ozyptila_trux
SMNKARA09028_8.48E_48.97N_ad_m__Ozyptila_praticola
ZFMKTIS20926_7.22E_50.47N_ad_m__Ozyptila_praticola
SMNKARA09035_8.48E_48.97N_ad_m__Ozyptila_praticola
SMNKARA08999_8.48E_48.97N_ad_m__Ozyptila_praticola
0.02

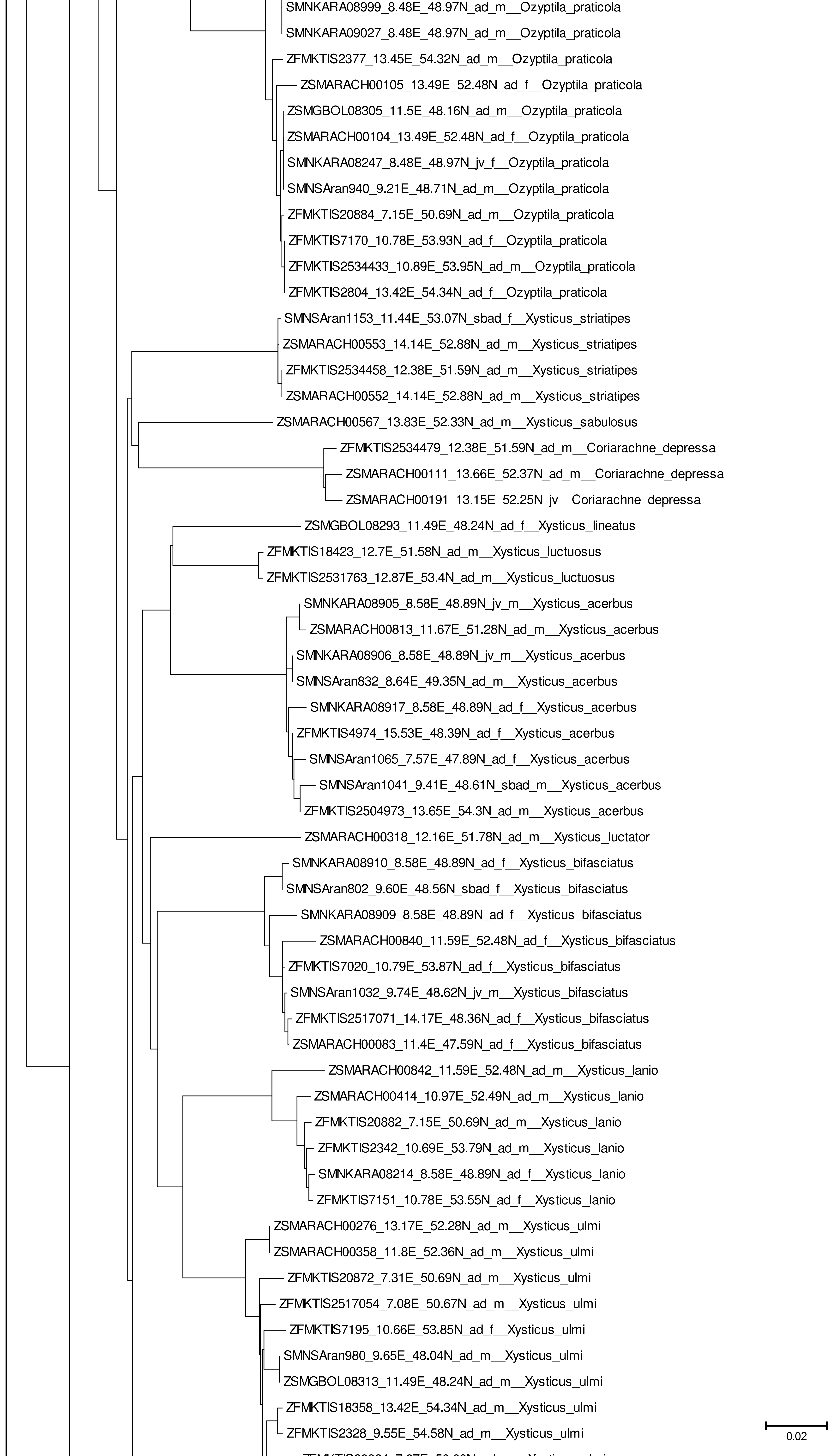
SMNKARA08999_8.48E_48.97N_ad_m__Ozyptila_praticola
SMNKARA09027_8.48E_48.97N_ad_m__Ozyptila_praticola
ZFMKTIS2377_13.45E_54.32N_ad_m__Ozyptila_praticola
ZSMARACH00105_13.49E_52.48N_ad_f__Ozyptila_praticola
ZSMGBOL08305_11.5E_48.16N_ad_m__Ozyptila_praticola
ZSMARACH00104_13.49E_52.48N_ad_f__Ozyptila_praticola
SMNKARA08247_8.48E_48.97N_jv_f__Ozyptila_praticola
SMNSAran940_9.21E_48.71N_ad_m__Ozyptila_praticola
ZFMKTIS20884_7.15E_50.69N_ad_m__Ozyptila_praticola
ZFMKTIS7170_10.78E_53.93N_ad_f__Ozyptila_praticola
ZFMKTIS2534433_10.89E_53.95N_ad_m__Ozyptila_praticola
ZFMKTIS2804_13.42E_54.34N_ad_f__Ozyptila_praticola
SMNSAran1153_11.44E_53.07N_sbad_f__Xysticus_striatipes
ZSMARACH00553_14.14E_52.88N_ad_m__Xysticus_striatipes
ZFMKTIS2534458_12.38E_51.59N_ad_m__Xysticus_striatipes
ZSMARACH00552_14.14E_52.88N_ad_m__Xysticus_striatipes
ZSMARACH00567_13.83E_52.33N_ad_m__Xysticus_sabulosus
ZFMKTIS2534479_12.38E_51.59N_ad_m__Coriarachne_depressa
ZSMARACH00111_13.66E_52.37N_ad_m__Coriarachne_depressa
ZSMARACH00191_13.15E_52.25N_jv__Coriarachne_depressa
ZSMGBOL08293_11.49E_48.24N_ad_f__Xysticus_lineatus
ZFMKTIS18423_12.7E_51.58N_ad_m__Xysticus_luctuosus
ZFMKTIS2531763_12.87E_53.4N_ad_m__Xysticus_luctuosus
SMNKARA08905_8.58E_48.89N_jv_m__Xysticus_acerbus
ZSMARACH00813_11.67E_51.28N_ad_m__Xysticus_acerbus
SMNKARA08906_8.58E_48.89N_jv_m__Xysticus_acerbus
SMNSAran832_8.64E_49.35N_ad_m__Xysticus_acerbus
SMNKARA08917_8.58E_48.89N_ad_f__Xysticus_acerbus
ZFMKTIS4974_15.53E_48.39N_ad_f__Xysticus_acerbus
SMNSAran1065_7.57E_47.89N_ad_f__Xysticus_acerbus
SMNSAran1041_9.41E_48.61N_sbad_m__Xysticus_acerbus
ZFMKTIS2504973_13.65E_54.3N_ad_m__Xysticus_acerbus
ZSMARACH00318_12.16E_51.78N_ad_m__Xysticus_luctator
SMNKARA08910_8.58E_48.89N_ad_f__Xysticus_bifasciatus
SMNSAran802_9.60E_48.56N_sbad_f__Xysticus_bifasciatus
SMNKARA08909_8.58E_48.89N_ad_f__Xysticus_bifasciatus
ZSMARACH00840_11.59E_52.48N_ad_f__Xysticus_bifasciatus
ZFMKTIS7020_10.79E_53.87N_ad_f__Xysticus_bifasciatus
SMNSAran1032_9.74E_48.62N_jv_m__Xysticus_bifasciatus
ZFMKTIS2517071_14.17E_48.36N_ad_f__Xysticus_bifasciatus
ZSMARACH00083_11.4E_47.59N_ad_f__Xysticus_bifasciatus
ZSMARACH00842_11.59E_52.48N_ad_m__Xysticus_lanio
ZSMARACH00414_10.97E_52.49N_ad_m__Xysticus_lanio
ZFMKTIS20882_7.15E_50.69N_ad_m__Xysticus_lanio
ZFMKTIS2342_10.69E_53.79N_ad_m__Xysticus_lanio
SMNKARA08214_8.58E_48.89N_ad_f__Xysticus_lanio
ZFMKTIS7151_10.78E_53.55N_ad_f__Xysticus_lanio
ZSMARACH00276_13.17E_52.28N_ad_m__Xysticus_ulmi
ZSMARACH00358_11.8E_52.36N_ad_m__Xysticus_ulmi
ZFMKTIS20872_7.31E_50.69N_ad_m__Xysticus_ulmi
ZFMKTIS2517054_7.08E_50.67N_ad_m__Xysticus_ulmi
ZFMKTIS7195_10.66E_53.85N_ad_f__Xysticus_ulmi
SMNSAran980_9.65E_48.04N_ad_m__Xysticus_ulmi
ZSMGBOL08313_11.49E_48.24N_ad_m__Xysticus_ulmi
ZFMKTIS18358_13.42E_54.34N_ad_m__Xysticus_ulmi
ZFMKTIS2328_9.55E_54.58N_ad_m__Xysticus_ulmi
0.02

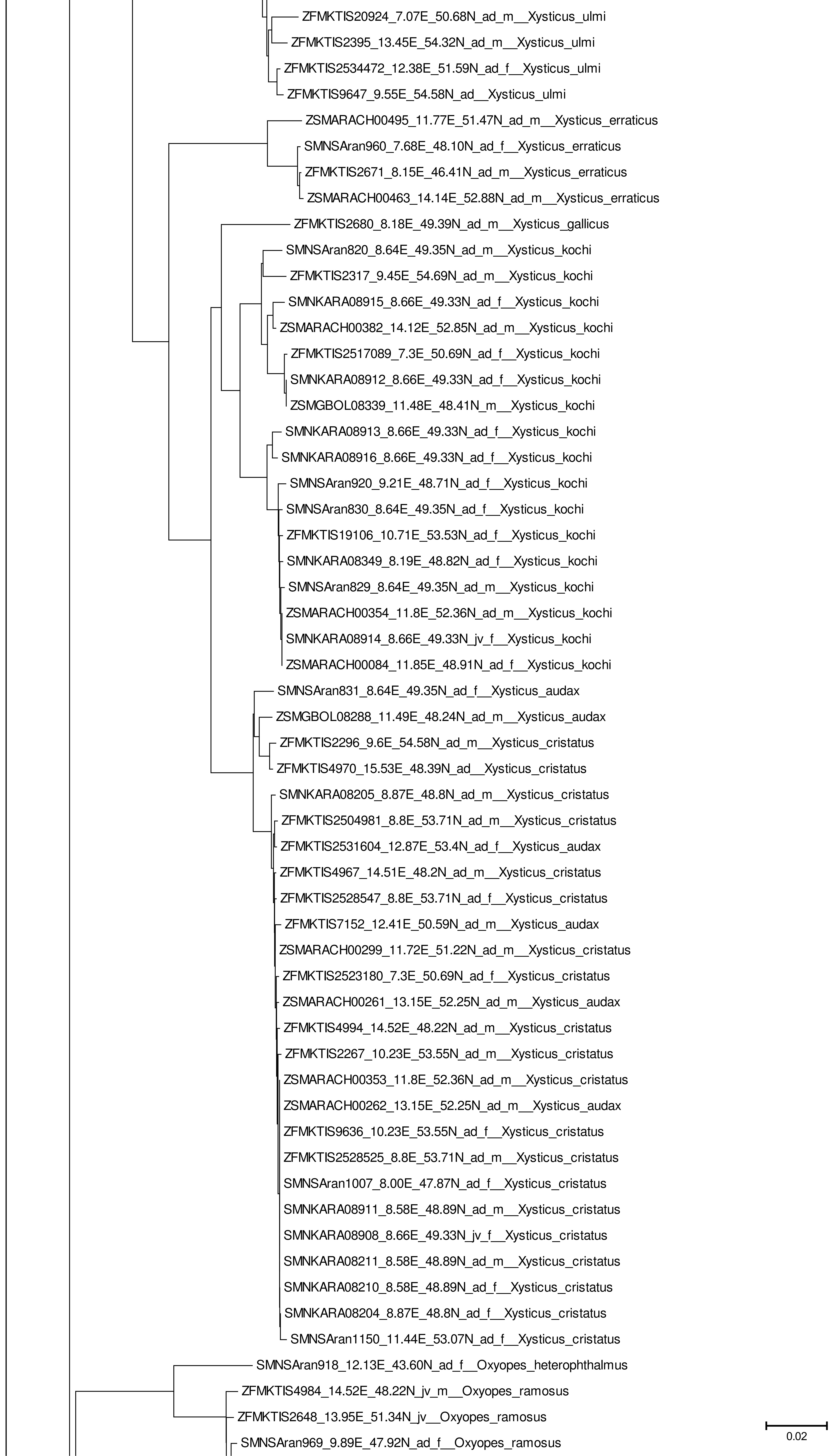

ZFMKTIS20924_7.07E_50.68N_ad_m__Xysticus_ulmi
ZFMKTIS2395_13.45E_54.32N_ad_m__Xysticus_ulmi
ZFMKTIS2534472_12.38E_51.59N_ad_f__Xysticus_ulmi
ZFMKTIS9647_9.55E_54.58N_ad__Xysticus_ulmi
ZSMARACH00495_11.77E_51.47N_ad_m__Xysticus_erraticus
SMNSAran960_7.68E_48.10N_ad_f__Xysticus_erraticus
ZFMKTIS2671_8.15E_46.41N_ad_m__Xysticus_erraticus
ZSMARACH00463_14.14E_52.88N_ad_m__Xysticus_erraticus
ZFMKTIS2680_8.18E_49.39N_ad_m__Xysticus_gallicus
SMNSAran820_8.64E_49.35N_ad_m__Xysticus_kochi
ZFMKTIS2317_9.45E_54.69N_ad_m__Xysticus_kochi
SMNKARA08915_8.66E_49.33N_ad_f__Xysticus_kochi
ZSMARACH00382_14.12E_52.85N_ad_m__Xysticus_kochi
ZFMKTIS2517089_7.3E_50.69N_ad_f__Xysticus_kochi
SMNKARA08912_8.66E_49.33N_ad_f__Xysticus_kochi
ZSMGBOL08339_11.48E_48.41N_m__Xysticus_kochi
SMNKARA08913_8.66E_49.33N_ad_f__Xysticus_kochi
SMNKARA08916_8.66E_49.33N_ad_f__Xysticus_kochi
SMNSAran920_9.21E_48.71N_ad_f__Xysticus_kochi
SMNSAran830_8.64E_49.35N_ad_f__Xysticus_kochi
ZFMKTIS19106_10.71E_53.53N_ad_f__Xysticus_kochi
SMNKARA08349_8.19E_48.82N_ad_f__Xysticus_kochi
SMNSAran829_8.64E_49.35N_ad_m__Xysticus_kochi
ZSMARACH00354_11.8E_52.36N_ad_m__Xysticus_kochi
SMNKARA08914_8.66E_49.33N_jv_f__Xysticus_kochi
ZSMARACH00084_11.85E_48.91N_ad_f__Xysticus_kochi
SMNSAran831_8.64E_49.35N_ad_f__Xysticus_audax
ZSMGBOL08288_11.49E_48.24N_ad_m__Xysticus_audax
ZFMKTIS2296_9.6E_54.58N_ad_m__Xysticus_cristatus
ZFMKTIS4970_15.53E_48.39N_ad__Xysticus_cristatus
SMNKARA08205_8.87E_48.8N_ad_m__Xysticus_cristatus
ZFMKTIS2504981_8.8E_53.71N_ad_m__Xysticus_cristatus
ZFMKTIS2531604_12.87E_53.4N_ad_f__Xysticus_audax
ZFMKTIS4967_14.51E_48.2N_ad_m__Xysticus_cristatus
ZFMKTIS2528547_8.8E_53.71N_ad_f__Xysticus_cristatus
ZFMKTIS7152_12.41E_50.59N_ad_m__Xysticus_audax
ZSMARACH00299_11.72E_51.22N_ad_m__Xysticus_cristatus
ZFMKTIS2523180_7.3E_50.69N_ad_f__Xysticus_cristatus
ZSMARACH00261_13.15E_52.25N_ad_m__Xysticus_audax
ZFMKTIS4994_14.52E_48.22N_ad_m__Xysticus_cristatus
ZFMKTIS2267_10.23E_53.55N_ad_m__Xysticus_cristatus
ZSMARACH00353_11.8E_52.36N_ad_m__Xysticus_cristatus
ZSMARACH00262_13.15E_52.25N_ad_m__Xysticus_audax
ZFMKTIS9636_10.23E_53.55N_ad_f__Xysticus_cristatus
ZFMKTIS2528525_8.8E_53.71N_ad_m__Xysticus_cristatus
SMNSAran1007_8.00E_47.87N_ad_f__Xysticus_cristatus
SMNKARA08911_8.58E_48.89N_ad_m__Xysticus_cristatus
SMNKARA08908_8.66E_49.33N_jv_f__Xysticus_cristatus
SMNKARA08211_8.58E_48.89N_ad_m__Xysticus_cristatus
SMNKARA08210_8.58E_48.89N_ad_f__Xysticus_cristatus
SMNKARA08204_8.87E_48.8N_ad_f__Xysticus_cristatus
SMNSAran1150_11.44E_53.07N_ad_f__Xysticus_cristatus
SMNSAran918_12.13E_43.60N_ad_f__Oxyopes_heterophthalmus
ZFMKTIS4984_14.52E_48.22N_jv_m__Oxyopes_ramosus
ZFMKTIS2648_13.95E_51.34N_jv__Oxyopes_ramosus
SMNSAran969_9.89E_47.92N_ad_f__Oxyopes_ramosus
0.02

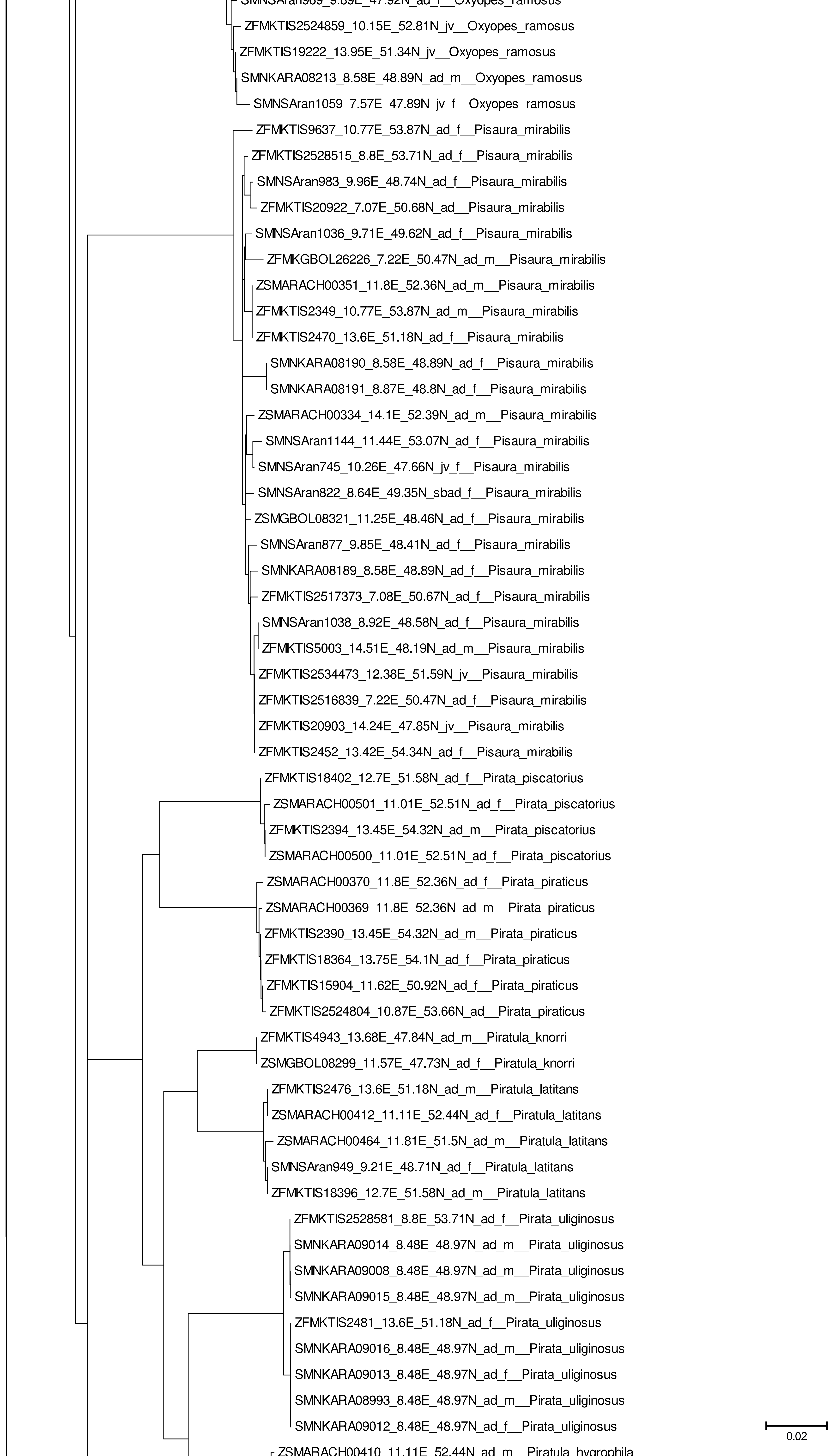
ZFMKTIS2524859_10.15E_52.81N_jv__Oxyopes_ramosus
ZFMKTIS19222_13.95E_51.34N_jv__Oxyopes_ramosus
SMNKARA08213_8.58E_48.89N_ad_m__Oxyopes_ramosus
SMNSAran1059_7.57E_47.89N_jv_f__Oxyopes_ramosus
ZFMKTIS9637_10.77E_53.87N_ad_f__Pisaura_mirabilis
ZFMKTIS2528515_8.8E_53.71N_ad_f__Pisaura_mirabilis
SMNSAran983_9.96E_48.74N_ad_f__Pisaura_mirabilis
ZFMKTIS20922_7.07E_50.68N_ad__Pisaura_mirabilis
SMNSAran1036_9.71E_49.62N_ad_f__Pisaura_mirabilis
ZFMKGBOL26226_7.22E_50.47N_ad_m__Pisaura_mirabilis
ZSMARACH00351_11.8E_52.36N_ad_m__Pisaura_mirabilis
ZFMKTIS2349_10.77E_53.87N_ad_m__Pisaura_mirabilis
ZFMKTIS2470_13.6E_51.18N_ad_f__Pisaura_mirabilis
SMNKARA08190_8.58E_48.89N_ad_f__Pisaura_mirabilis
SMNKARA08191_8.87E_48.8N_ad_f__Pisaura_mirabilis
ZSMARACH00334_14.1E_52.39N_ad_m__Pisaura_mirabilis
SMNSAran1144_11.44E_53.07N_ad_f__Pisaura_mirabilis
SMNSAran745_10.26E_47.66N_jv_f__Pisaura_mirabilis
SMNSAran822_8.64E_49.35N_sbad_f__Pisaura_mirabilis
ZSMGBOL08321_11.25E_48.46N_ad_f__Pisaura_mirabilis
SMNSAran877_9.85E_48.41N_ad_f__Pisaura_mirabilis
SMNKARA08189_8.58E_48.89N_ad_f__Pisaura_mirabilis
ZFMKTIS2517373_7.08E_50.67N_ad_f__Pisaura_mirabilis
SMNSAran1038_8.92E_48.58N_ad_f__Pisaura_mirabilis
ZFMKTIS5003_14.51E_48.19N_ad_m__Pisaura_mirabilis
ZFMKTIS2534473_12.38E_51.59N_jv__Pisaura_mirabilis
ZFMKTIS2516839_7.22E_50.47N_ad_f__Pisaura_mirabilis
ZFMKTIS20903_14.24E_47.85N_jv__Pisaura_mirabilis
ZFMKTIS2452_13.42E_54.34N_ad_f__Pisaura_mirabilis
ZFMKTIS18402_12.7E_51.58N_ad_f__Pirata_piscatorius
ZSMARACH00501_11.01E_52.51N_ad_f__Pirata_piscatorius
ZFMKTIS2394_13.45E_54.32N_ad_m__Pirata_piscatorius
ZSMARACH00500_11.01E_52.51N_ad_f__Pirata_piscatorius
ZSMARACH00370_11.8E_52.36N_ad_f__Pirata_piraticus
ZSMARACH00369_11.8E_52.36N_ad_m__Pirata_piraticus
ZFMKTIS2390_13.45E_54.32N_ad_m__Pirata_piraticus
ZFMKTIS18364_13.75E_54.1N_ad_f__Pirata_piraticus
ZFMKTIS15904_11.62E_50.92N_ad_f__Pirata_piraticus
ZFMKTIS2524804_10.87E_53.66N_ad__Pirata_piraticus
ZFMKTIS4943_13.68E_47.84N_ad_m__Piratula_knorri
ZSMGBOL08299_11.57E_47.73N_ad_f__Piratula_knorri
ZFMKTIS2476_13.6E_51.18N_ad_m__Piratula_latitans
ZSMARACH00412_11.11E_52.44N_ad_f__Piratula_latitans
ZSMARACH00464_11.81E_51.5N_ad_m__Piratula_latitans
SMNSAran949_9.21E_48.71N_ad_f__Piratula_latitans
ZFMKTIS18396_12.7E_51.58N_ad_m__Piratula_latitans
ZFMKTIS2528581_8.8E_53.71N_ad_f__Pirata_uliginosus
SMNKARA09014_8.48E_48.97N_ad_m__Pirata_uliginosus
SMNKARA09008_8.48E_48.97N_ad_m__Pirata_uliginosus
SMNKARA09015_8.48E_48.97N_ad_m__Pirata_uliginosus
ZFMKTIS2481_13.6E_51.18N_ad_f__Pirata_uliginosus
SMNKARA09016_8.48E_48.97N_ad_m__Pirata_uliginosus
SMNKARA09013_8.48E_48.97N_ad_f__Pirata_uliginosus
SMNKARA08993_8.48E_48.97N_ad_m__Pirata_uliginosus
SMNKARA09012_8.48E_48.97N_ad_f__Pirata_uliginosus
0.02

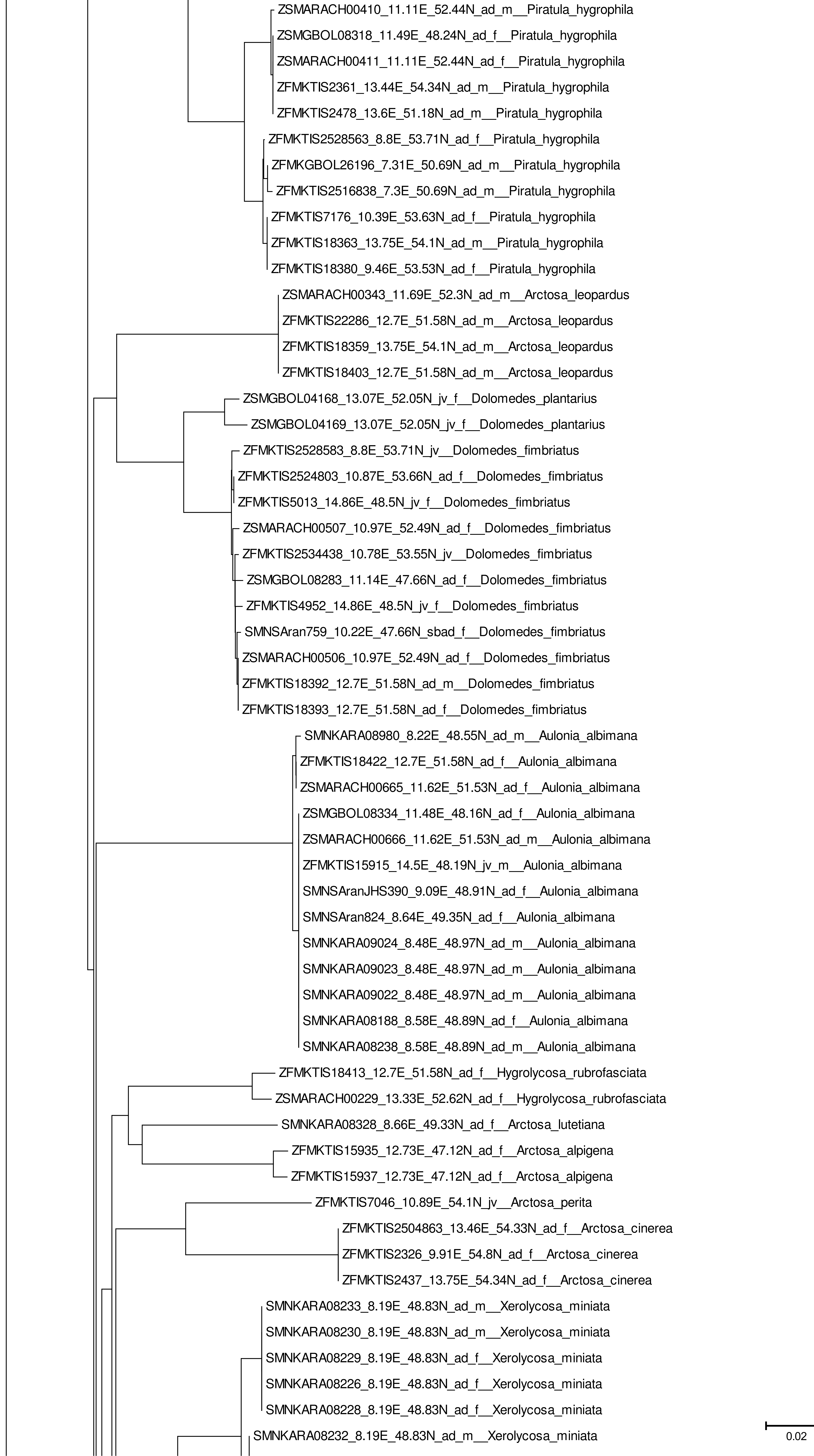
ZSMARACH00410_11.11E_52.44N_ad_m__Piratula_hygrophila
ZSMGBOL08318_11.49E_48.24N_ad_f__Piratula_hygrophila
ZSMARACH00411_11.11E_52.44N_ad_f__Piratula_hygrophila
ZFMKTIS2361_13.44E_54.34N_ad_m__Piratula_hygrophila
ZFMKTIS2478_13.6E_51.18N_ad_m__Piratula_hygrophila
ZFMKTIS2528563_8.8E_53.71N_ad_f__Piratula_hygrophila
ZFMKGBOL26196_7.31E_50.69N_ad_m__Piratula_hygrophila
ZFMKTIS2516838_7.3E_50.69N_ad_m__Piratula_hygrophila
ZFMKTIS7176_10.39E_53.63N_ad_f__Piratula_hygrophila
ZFMKTIS18363_13.75E_54.1N_ad_m__Piratula_hygrophila
ZFMKTIS18380_9.46E_53.53N_ad_f__Piratula_hygrophila
ZSMARACH00343_11.69E_52.3N_ad_m__Arctosa_leopardus
ZFMKTIS22286_12.7E_51.58N_ad_m__Arctosa_leopardus
ZFMKTIS18359_13.75E_54.1N_ad_m__Arctosa_leopardus
ZFMKTIS18403_12.7E_51.58N_ad_m__Arctosa_leopardus
ZSMGBOL04168_13.07E_52.05N_jv_f__Dolomedes_plantarius
ZSMGBOL04169_13.07E_52.05N_jv_f__Dolomedes_plantarius
ZFMKTIS2528583_8.8E_53.71N_jv__Dolomedes_fimbriatus
ZFMKTIS2524803_10.87E_53.66N_ad_f__Dolomedes_fimbriatus
ZFMKTIS5013_14.86E_48.5N_jv_f__Dolomedes_fimbriatus
ZSMARACH00507_10.97E_52.49N_ad_f__Dolomedes_fimbriatus
ZFMKTIS2534438_10.78E_53.55N_jv__Dolomedes_fimbriatus
ZSMGBOL08283_11.14E_47.66N_ad_f__Dolomedes_fimbriatus
ZFMKTIS4952_14.86E_48.5N_jv_f__Dolomedes_fimbriatus
SMNSAran759_10.22E_47.66N_sbad_f__Dolomedes_fimbriatus
ZSMARACH00506_10.97E_52.49N_ad_f__Dolomedes_fimbriatus
ZFMKTIS18392_12.7E_51.58N_ad_m__Dolomedes_fimbriatus
ZFMKTIS18393_12.7E_51.58N_ad_f__Dolomedes_fimbriatus
SMNKARA08980_8.22E_48.55N_ad_m__Aulonia_albimana
ZFMKTIS18422_12.7E_51.58N_ad_f__Aulonia_albimana
ZSMARACH00665_11.62E_51.53N_ad_f__Aulonia_albimana
ZSMGBOL08334_11.48E_48.16N_ad_f__Aulonia_albimana
ZSMARACH00666_11.62E_51.53N_ad_m__Aulonia_albimana
ZFMKTIS15915_14.5E_48.19N_jv_m__Aulonia_albimana
SMNSAranJHS390_9.09E_48.91N_ad_f__Aulonia_albimana
SMNSAran824_8.64E_49.35N_ad_f__Aulonia_albimana
SMNKARA09024_8.48E_48.97N_ad_m__Aulonia_albimana
SMNKARA09023_8.48E_48.97N_ad_m__Aulonia_albimana
SMNKARA09022_8.48E_48.97N_ad_m__Aulonia_albimana
SMNKARA08188_8.58E_48.89N_ad_f__Aulonia_albimana
SMNKARA08238_8.58E_48.89N_ad_m__Aulonia_albimana
ZFMKTIS18413_12.7E_51.58N_ad_f__Hygrolycosa_rubrofasciata
ZSMARACH00229_13.33E_52.62N_ad_f__Hygrolycosa_rubrofasciata
SMNKARA08328_8.66E_49.33N_ad_f__Arctosa_lutetiana
ZFMKTIS15935_12.73E_47.12N_ad_f__Arctosa_alpigena
ZFMKTIS15937_12.73E_47.12N_ad_f__Arctosa_alpigena
ZFMKTIS7046_10.89E_54.1N_jv__Arctosa_perita
ZFMKTIS2504863_13.46E_54.33N_ad_f__Arctosa_cinerea
ZFMKTIS2326_9.91E_54.8N_ad_f__Arctosa_cinerea
ZFMKTIS2437_13.75E_54.34N_ad_f__Arctosa_cinerea
SMNKARA08233_8.19E_48.83N_ad_m__Xerolycosa_miniata
SMNKARA08230_8.19E_48.83N_ad_m__Xerolycosa_miniata
SMNKARA08229_8.19E_48.83N_ad_f__Xerolycosa_miniata
SMNKARA08226_8.19E_48.83N_ad_f__Xerolycosa_miniata
SMNKARA08228_8.19E_48.83N_ad_f__Xerolycosa_miniata
SMNKARA08232_8.19E_48.83N_ad_m__Xerolycosa_miniata
0.02

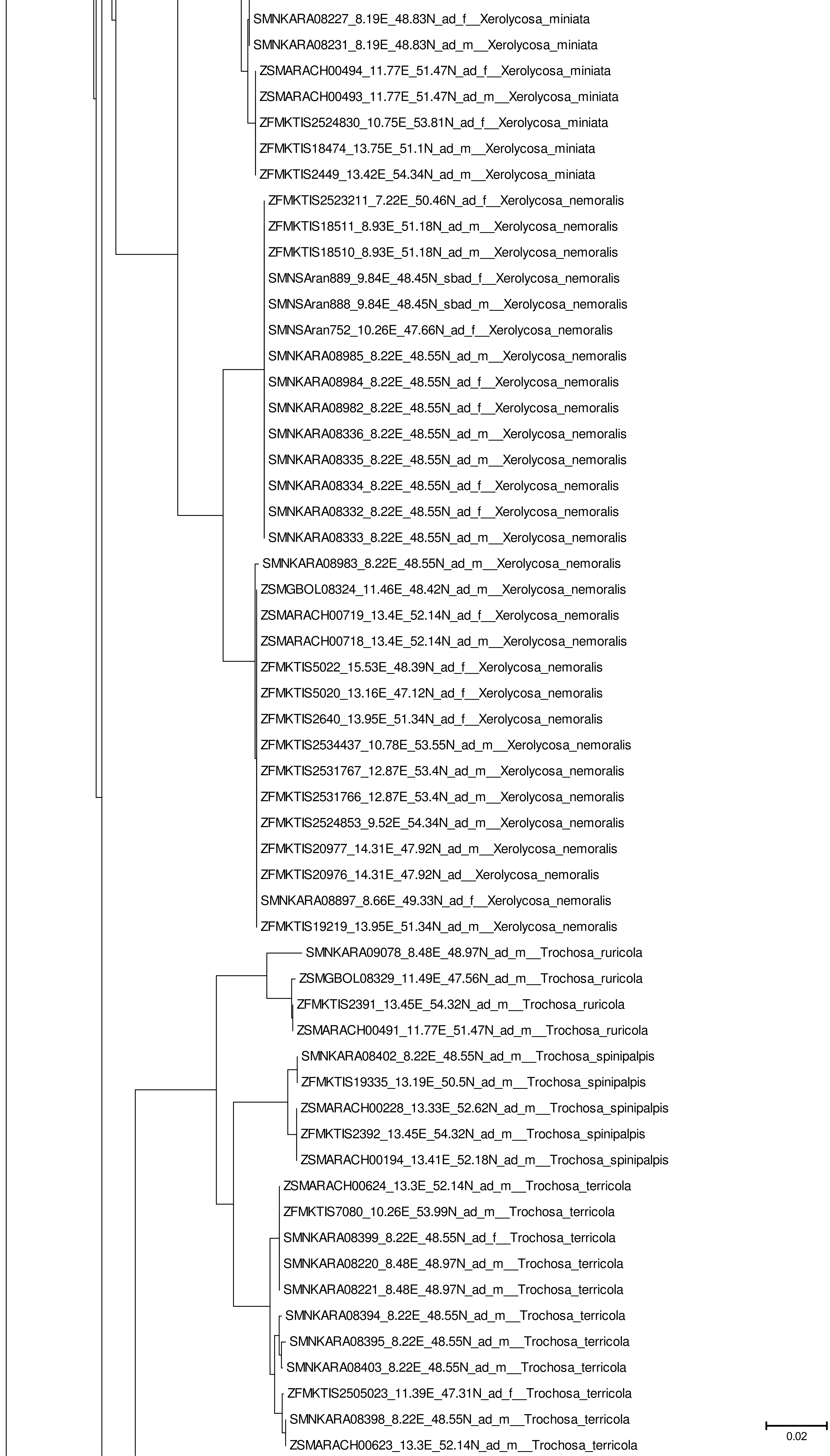
SMNKARA08227_8.19E_48.83N_ad_f__Xerolycosa_miniata
SMNKARA08231_8.19E_48.83N_ad_m__Xerolycosa_miniata
ZSMARACH00494_11.77E_51.47N_ad_f__Xerolycosa_miniata
ZSMARACH00493_11.77E_51.47N_ad_m__Xerolycosa_miniata
ZFMKTIS2524830_10.75E_53.81N_ad_f__Xerolycosa_miniata
ZFMKTIS18474_13.75E_51.1N_ad_m__Xerolycosa_miniata
ZFMKTIS2449_13.42E_54.34N_ad_m__Xerolycosa_miniata
ZFMKTIS2523211_7.22E_50.46N_ad_f__Xerolycosa_nemoralis
ZFMKTIS18511_8.93E_51.18N_ad_m__Xerolycosa_nemoralis
ZFMKTIS18510_8.93E_51.18N_ad_m__Xerolycosa_nemoralis
SMNSAran889_9.84E_48.45N_sbad_f__Xerolycosa_nemoralis
SMNSAran888_9.84E_48.45N_sbad_m__Xerolycosa_nemoralis
SMNSAran752_10.26E_47.66N_ad_f__Xerolycosa_nemoralis
SMNKARA08985_8.22E_48.55N_ad_m__Xerolycosa_nemoralis
SMNKARA08984_8.22E_48.55N_ad_f__Xerolycosa_nemoralis
SMNKARA08982_8.22E_48.55N_ad_f__Xerolycosa_nemoralis
SMNKARA08336_8.22E_48.55N_ad_m__Xerolycosa_nemoralis
SMNKARA08335_8.22E_48.55N_ad_m__Xerolycosa_nemoralis
SMNKARA08334_8.22E_48.55N_ad_f__Xerolycosa_nemoralis
SMNKARA08332_8.22E_48.55N_ad_f__Xerolycosa_nemoralis
SMNKARA08333_8.22E_48.55N_ad_m__Xerolycosa_nemoralis
SMNKARA08983_8.22E_48.55N_ad_m__Xerolycosa_nemoralis
ZSMGBOL08324_11.46E_48.42N_ad_m__Xerolycosa_nemoralis
ZSMARACH00719_13.4E_52.14N_ad_f__Xerolycosa_nemoralis
ZSMARACH00718_13.4E_52.14N_ad_m__Xerolycosa_nemoralis
ZFMKTIS5022_15.53E_48.39N_ad_f__Xerolycosa_nemoralis
ZFMKTIS5020_13.16E_47.12N_ad_f__Xerolycosa_nemoralis
ZFMKTIS2640_13.95E_51.34N_ad_f__Xerolycosa_nemoralis
ZFMKTIS2534437_10.78E_53.55N_ad_m__Xerolycosa_nemoralis
ZFMKTIS2531767_12.87E_53.4N_ad_m__Xerolycosa_nemoralis
ZFMKTIS2531766_12.87E_53.4N_ad_m__Xerolycosa_nemoralis
ZFMKTIS2524853_9.52E_54.34N_ad_m__Xerolycosa_nemoralis
ZFMKTIS20977_14.31E_47.92N_ad_m__Xerolycosa_nemoralis
ZFMKTIS20976_14.31E_47.92N_ad__Xerolycosa_nemoralis
SMNKARA08897_8.66E_49.33N_ad_f__Xerolycosa_nemoralis
ZFMKTIS19219_13.95E_51.34N_ad_m__Xerolycosa_nemoralis
SMNKARA09078_8.48E_48.97N_ad_m__Trochosa_ruricola
ZSMGBOL08329_11.49E_47.56N_ad_m__Trochosa_ruricola
ZFMKTIS2391_13.45E_54.32N_ad_m__Trochosa_ruricola
ZSMARACH00491_11.77E_51.47N_ad_m__Trochosa_ruricola
SMNKARA08402_8.22E_48.55N_ad_m__Trochosa_spinipalpis
ZFMKTIS19335_13.19E_50.5N_ad_m__Trochosa_spinipalpis
ZSMARACH00228_13.33E_52.62N_ad_m__Trochosa_spinipalpis
ZFMKTIS2392_13.45E_54.32N_ad_m__Trochosa_spinipalpis
ZSMARACH00194_13.41E_52.18N_ad_m__Trochosa_spinipalpis
ZSMARACH00624_13.3E_52.14N_ad_m__Trochosa_terricola
ZFMKTIS7080_10.26E_53.99N_ad_m__Trochosa_terricola
SMNKARA08399_8.22E_48.55N_ad_f__Trochosa_terricola
SMNKARA08220_8.48E_48.97N_ad_m__Trochosa_terricola
SMNKARA08221_8.48E_48.97N_ad_m__Trochosa_terricola
SMNKARA08394_8.22E_48.55N_ad_m__Trochosa_terricola
SMNKARA08395_8.22E_48.55N_ad_m__Trochosa_terricola
SMNKARA08403_8.22E_48.55N_ad_m__Trochosa_terricola
ZFMKTIS2505023_11.39E_47.31N_ad_f__Trochosa_terricola
SMNKARA08398_8.22E_48.55N_ad_m__Trochosa_terricola
ZSMARACH00623_13.3E_52.14N_ad_m__Trochosa_terricola
0.02

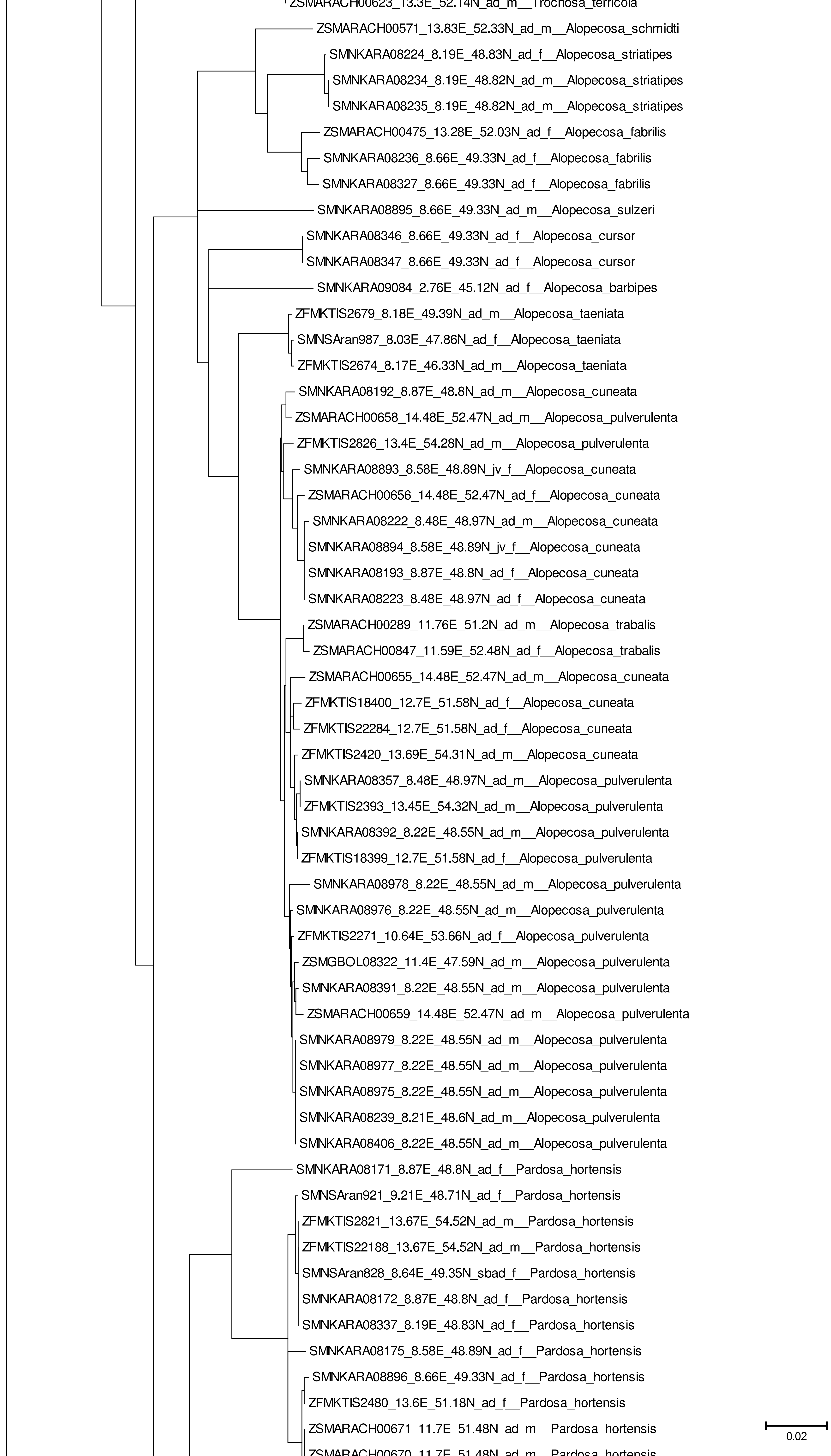

ZSMARACH00623_13.3E_52.14N_ad_m__Trochosa_terricola
ZSMARACH00571_13.83E_52.33N_ad_m__Alopecosa_schmidti
SMNKARA08224_8.19E_48.83N_ad_f__Alopecosa_striatipes
SMNKARA08234_8.19E_48.82N_ad_m__Alopecosa_striatipes
SMNKARA08235_8.19E_48.82N_ad_m__Alopecosa_striatipes
ZSMARACH00475_13.28E_52.03N_ad_f__Alopecosa_fabrilis
SMNKARA08236_8.66E_49.33N_ad_f__Alopecosa_fabrilis
SMNKARA08327_8.66E_49.33N_ad_f__Alopecosa_fabrilis
SMNKARA08895_8.66E_49.33N_ad_m__Alopecosa_sulzeri
SMNKARA08346_8.66E_49.33N_ad_f__Alopecosa_cursor
SMNKARA08347_8.66E_49.33N_ad_f__Alopecosa_cursor
SMNKARA09084_2.76E_45.12N_ad_f__Alopecosa_barbipes
ZFMKTIS2679_8.18E_49.39N_ad_m__Alopecosa_taeniata
SMNSAran987_8.03E_47.86N_ad_f__Alopecosa_taeniata
ZFMKTIS2674_8.17E_46.33N_ad_m__Alopecosa_taeniata
SMNKARA08192_8.87E_48.8N_ad_m__Alopecosa_cuneata
ZSMARACH00658_14.48E_52.47N_ad_m__Alopecosa_pulverulenta
ZFMKTIS2826_13.4E_54.28N_ad_m__Alopecosa_pulverulenta
SMNKARA08893_8.58E_48.89N_jv_f__Alopecosa_cuneata
ZSMARACH00656_14.48E_52.47N_ad_f__Alopecosa_cuneata
SMNKARA08222_8.48E_48.97N_ad_m__Alopecosa_cuneata
SMNKARA08894_8.58E_48.89N_jv_f__Alopecosa_cuneata
SMNKARA08193_8.87E_48.8N_ad_f__Alopecosa_cuneata
SMNKARA08223_8.48E_48.97N_ad_f__Alopecosa_cuneata
ZSMARACH00289_11.76E_51.2N_ad_m__Alopecosa_trabalis
ZSMARACH00847_11.59E_52.48N_ad_f__Alopecosa_trabalis
ZSMARACH00655_14.48E_52.47N_ad_m__Alopecosa_cuneata
ZFMKTIS18400_12.7E_51.58N_ad_f__Alopecosa_cuneata
ZFMKTIS22284_12.7E_51.58N_ad_f__Alopecosa_cuneata
ZFMKTIS2420_13.69E_54.31N_ad_m__Alopecosa_cuneata
SMNKARA08357_8.48E_48.97N_ad_m__Alopecosa_pulverulenta
ZFMKTIS2393_13.45E_54.32N_ad_m__Alopecosa_pulverulenta
SMNKARA08392_8.22E_48.55N_ad_m__Alopecosa_pulverulenta
ZFMKTIS18399_12.7E_51.58N_ad_f__Alopecosa_pulverulenta
SMNKARA08978_8.22E_48.55N_ad_m__Alopecosa_pulverulenta
SMNKARA08976_8.22E_48.55N_ad_m__Alopecosa_pulverulenta
ZFMKTIS2271_10.64E_53.66N_ad_f__Alopecosa_pulverulenta
ZSMGBOL08322_11.4E_47.59N_ad_m__Alopecosa_pulverulenta
SMNKARA08391_8.22E_48.55N_ad_m__Alopecosa_pulverulenta
ZSMARACH00659_14.48E_52.47N_ad_m__Alopecosa_pulverulenta
SMNKARA08979_8.22E_48.55N_ad_m__Alopecosa_pulverulenta
SMNKARA08977_8.22E_48.55N_ad_m__Alopecosa_pulverulenta
SMNKARA08975_8.22E_48.55N_ad_m__Alopecosa_pulverulenta
SMNKARA08239_8.21E_48.6N_ad_m__Alopecosa_pulverulenta
SMNKARA08406_8.22E_48.55N_ad_m__Alopecosa_pulverulenta
SMNKARA08171_8.87E_48.8N_ad_f__Pardosa_hortensis
SMNSAran921_9.21E_48.71N_ad_f__Pardosa_hortensis
ZFMKTIS2821_13.67E_54.52N_ad_m__Pardosa_hortensis
ZFMKTIS22188_13.67E_54.52N_ad_m__Pardosa_hortensis
SMNSAran828_8.64E_49.35N_sbad_f__Pardosa_hortensis
SMNKARA08172_8.87E_48.8N_ad_f__Pardosa_hortensis
SMNKARA08337_8.19E_48.83N_ad_f__Pardosa_hortensis
SMNKARA08175_8.58E_48.89N_ad_f__Pardosa_hortensis
SMNKARA08896_8.66E_49.33N_ad_f__Pardosa_hortensis
ZFMKTIS2480_13.6E_51.18N_ad_f__Pardosa_hortensis
ZSMARACH00671_11.7E_51.48N_ad_m__Pardosa_hortensis
ZSMARACH00670_11.7E_51.48N_ad_m__Pardosa_hortensis
0.02

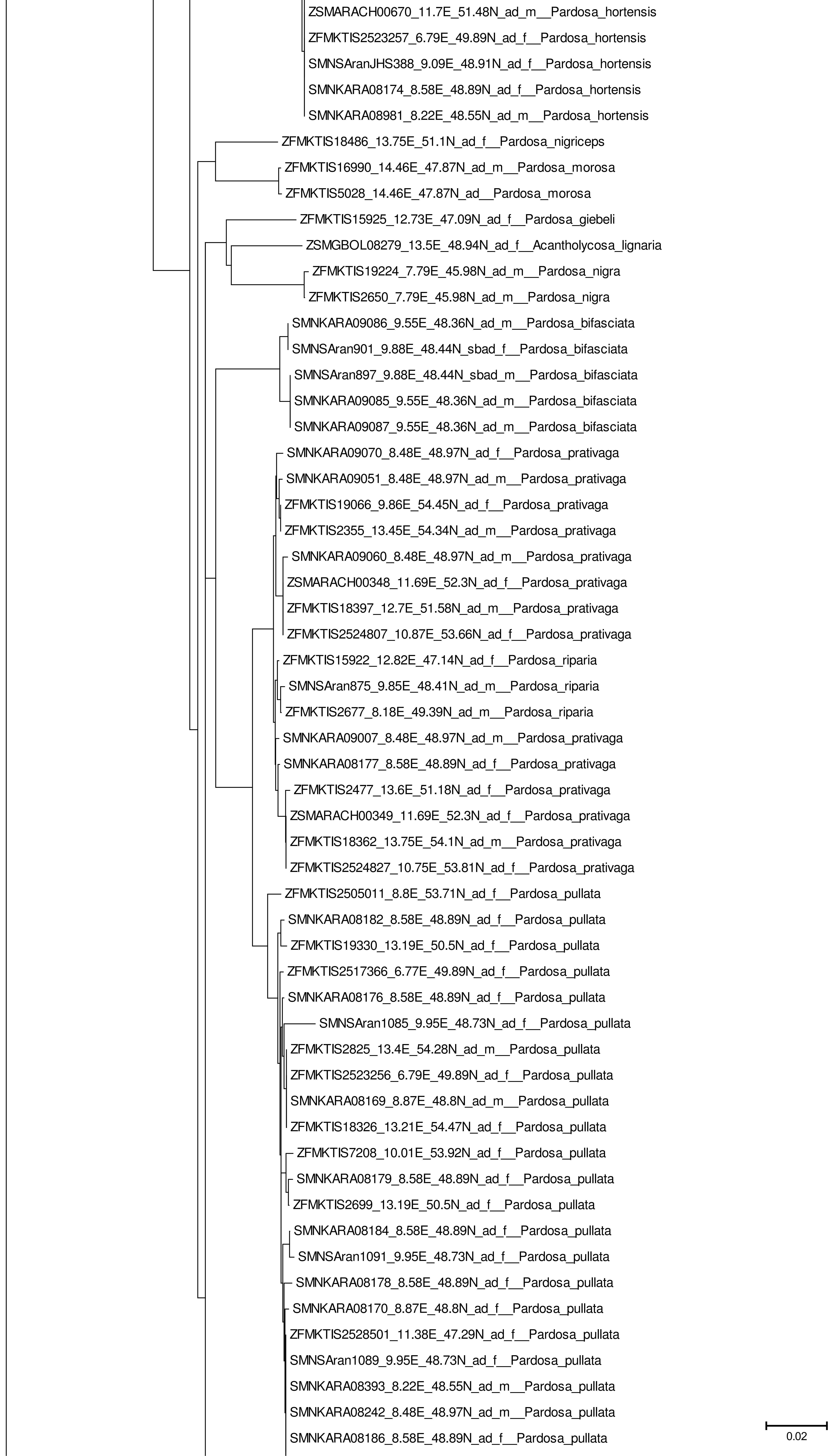
ZSMARACH00670_11.7E_51.48N_ad_m__Pardosa_hortensis
ZFMKTIS2523257_6.79E_49.89N_ad_f__Pardosa_hortensis
SMNSAranJHS388_9.09E_48.91N_ad_f__Pardosa_hortensis
SMNKARA08174_8.58E_48.89N_ad_f__Pardosa_hortensis
SMNKARA08981_8.22E_48.55N_ad_m__Pardosa_hortensis
ZFMKTIS18486_13.75E_51.1N_ad_f__Pardosa_nigriceps
ZFMKTIS16990_14.46E_47.87N_ad_m__Pardosa_morosa
ZFMKTIS5028_14.46E_47.87N_ad__Pardosa_morosa
ZFMKTIS15925_12.73E_47.09N_ad_f__Pardosa_giebeli
ZSMGBOL08279_13.5E_48.94N_ad_f__Acantholycosa_lignaria
ZFMKTIS19224_7.79E_45.98N_ad_m__Pardosa_nigra
ZFMKTIS2650_7.79E_45.98N_ad_m__Pardosa_nigra
SMNKARA09086_9.55E_48.36N_ad_m__Pardosa_bifasciata
SMNSAran901_9.88E_48.44N_sbad_f__Pardosa_bifasciata
SMNSAran897_9.88E_48.44N_sbad_m__Pardosa_bifasciata
SMNKARA09085_9.55E_48.36N_ad_m__Pardosa_bifasciata
SMNKARA09087_9.55E_48.36N_ad_m__Pardosa_bifasciata
SMNKARA09070_8.48E_48.97N_ad_f__Pardosa_prativaga
SMNKARA09051_8.48E_48.97N_ad_m__Pardosa_prativaga
ZFMKTIS19066_9.86E_54.45N_ad_f__Pardosa_prativaga
ZFMKTIS2355_13.45E_54.34N_ad_m__Pardosa_prativaga
SMNKARA09060_8.48E_48.97N_ad_m__Pardosa_prativaga
ZSMARACH00348_11.69E_52.3N_ad_f__Pardosa_prativaga
ZFMKTIS18397_12.7E_51.58N_ad_m__Pardosa_prativaga
ZFMKTIS2524807_10.87E_53.66N_ad_f__Pardosa_prativaga
ZFMKTIS15922_12.82E_47.14N_ad_f__Pardosa_riparia
SMNSAran875_9.85E_48.41N_ad_m__Pardosa_riparia
ZFMKTIS2677_8.18E_49.39N_ad_m__Pardosa_riparia
SMNKARA09007_8.48E_48.97N_ad_m__Pardosa_prativaga
SMNKARA08177_8.58E_48.89N_ad_f__Pardosa_prativaga
ZFMKTIS2477_13.6E_51.18N_ad_f__Pardosa_prativaga
ZSMARACH00349_11.69E_52.3N_ad_f__Pardosa_prativaga
ZFMKTIS18362_13.75E_54.1N_ad_m__Pardosa_prativaga
ZFMKTIS2524827_10.75E_53.81N_ad_f__Pardosa_prativaga
ZFMKTIS2505011_8.8E_53.71N_ad_f__Pardosa_pullata
SMNKARA08182_8.58E_48.89N_ad_f__Pardosa_pullata
ZFMKTIS19330_13.19E_50.5N_ad_f__Pardosa_pullata
ZFMKTIS2517366_6.77E_49.89N_ad_f__Pardosa_pullata
SMNKARA08176_8.58E_48.89N_ad_f__Pardosa_pullata
SMNSAran1085_9.95E_48.73N_ad_f__Pardosa_pullata
ZFMKTIS2825_13.4E_54.28N_ad_m__Pardosa_pullata
ZFMKTIS2523256_6.79E_49.89N_ad_f__Pardosa_pullata
SMNKARA08169_8.87E_48.8N_ad_m__Pardosa_pullata
ZFMKTIS18326_13.21E_54.47N_ad_f__Pardosa_pullata
ZFMKTIS7208_10.01E_53.92N_ad_f__Pardosa_pullata
SMNKARA08179_8.58E_48.89N_ad_f__Pardosa_pullata
ZFMKTIS2699_13.19E_50.5N_ad_f__Pardosa_pullata
SMNKARA08184_8.58E_48.89N_ad_f__Pardosa_pullata
SMNSAran1091_9.95E_48.73N_ad_f__Pardosa_pullata
SMNKARA08178_8.58E_48.89N_ad_f__Pardosa_pullata
SMNKARA08170_8.87E_48.8N_ad_f__Pardosa_pullata
ZFMKTIS2528501_11.38E_47.29N_ad_f__Pardosa_pullata
SMNSAran1089_9.95E_48.73N_ad_f__Pardosa_pullata
SMNKARA08393_8.22E_48.55N_ad_m__Pardosa_pullata
SMNKARA08242_8.48E_48.97N_ad_m__Pardosa_pullata
SMNKARA08186_8.58E_48.89N_ad_f__Pardosa_pullata
0.02

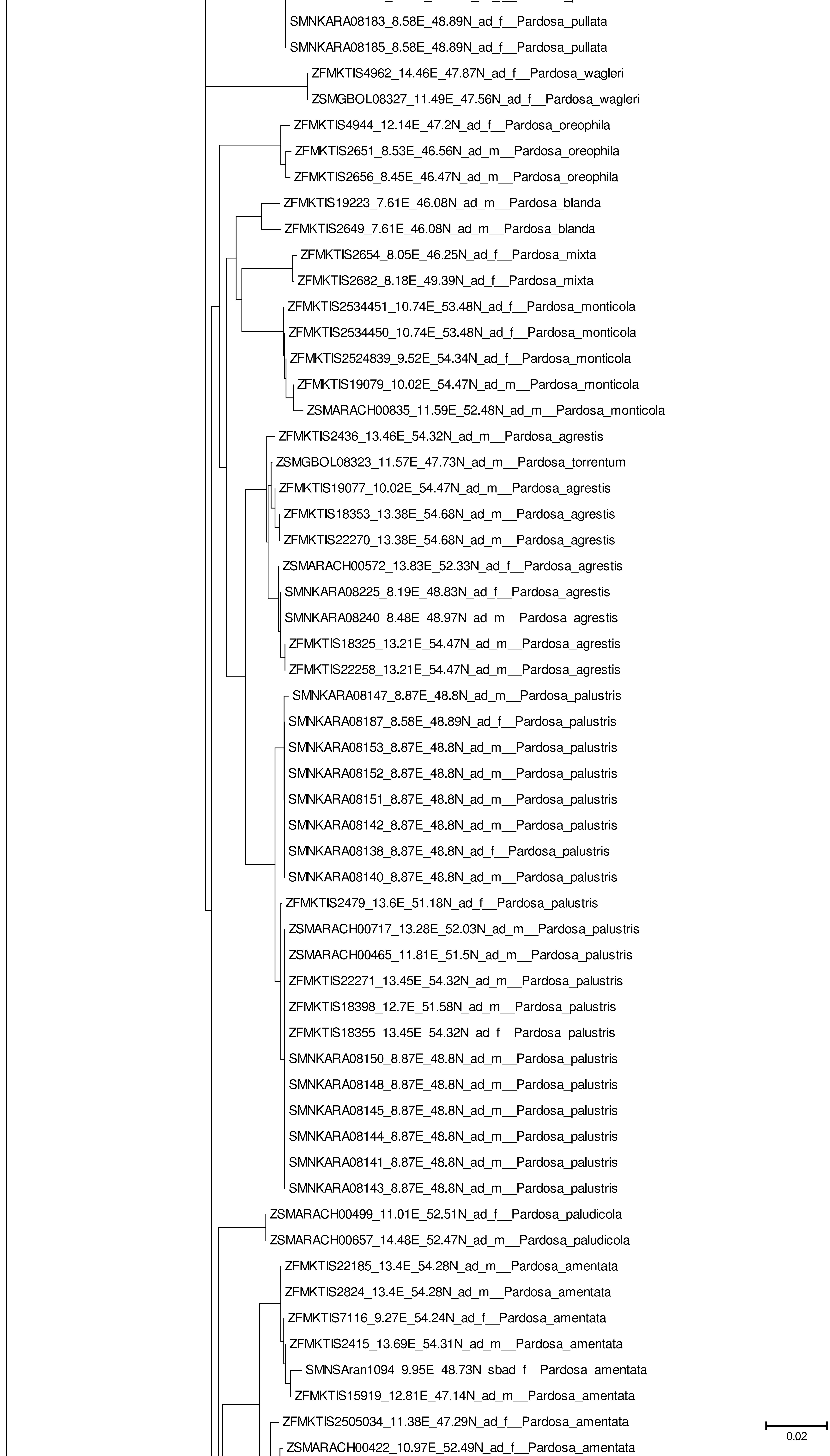
SMNKARA08183_8.58E_48.89N_ad_f__Pardosa_pullata
SMNKARA08185_8.58E_48.89N_ad_f__Pardosa_pullata
ZFMKTIS4962_14.46E_47.87N_ad_f__Pardosa_wagleri
ZSMGBOL08327_11.49E_47.56N_ad_f__Pardosa_wagleri
ZFMKTIS4944_12.14E_47.2N_ad_f__Pardosa_oreophila
ZFMKTIS2651_8.53E_46.56N_ad_m__Pardosa_oreophila
ZFMKTIS2656_8.45E_46.47N_ad_m__Pardosa_oreophila
ZFMKTIS19223_7.61E_46.08N_ad_m__Pardosa_blanda
ZFMKTIS2649_7.61E_46.08N_ad_m__Pardosa_blanda
ZFMKTIS2654_8.05E_46.25N_ad_f__Pardosa_mixta
ZFMKTIS2682_8.18E_49.39N_ad_f__Pardosa_mixta
ZFMKTIS2534451_10.74E_53.48N_ad_f__Pardosa_monticola
ZFMKTIS2534450_10.74E_53.48N_ad_f__Pardosa_monticola
ZFMKTIS2524839_9.52E_54.34N_ad_f__Pardosa_monticola
ZFMKTIS19079_10.02E_54.47N_ad_m__Pardosa_monticola
ZSMARACH00835_11.59E_52.48N_ad_m__Pardosa_monticola
ZFMKTIS2436_13.46E_54.32N_ad_m__Pardosa_agrestis
ZSMGBOL08323_11.57E_47.73N_ad_m__Pardosa_torrentum
ZFMKTIS19077_10.02E_54.47N_ad_m__Pardosa_agrestis
ZFMKTIS18353_13.38E_54.68N_ad_m__Pardosa_agrestis
ZFMKTIS22270_13.38E_54.68N_ad_m__Pardosa_agrestis
ZSMARACH00572_13.83E_52.33N_ad_f__Pardosa_agrestis
SMNKARA08225_8.19E_48.83N_ad_f__Pardosa_agrestis
SMNKARA08240_8.48E_48.97N_ad_m__Pardosa_agrestis
ZFMKTIS18325_13.21E_54.47N_ad_m__Pardosa_agrestis
ZFMKTIS22258_13.21E_54.47N_ad_m__Pardosa_agrestis
SMNKARA08147_8.87E_48.8N_ad_m__Pardosa_palustris
SMNKARA08187_8.58E_48.89N_ad_f__Pardosa_palustris
SMNKARA08153_8.87E_48.8N_ad_m__Pardosa_palustris
SMNKARA08152_8.87E_48.8N_ad_m__Pardosa_palustris
SMNKARA08151_8.87E_48.8N_ad_m__Pardosa_palustris
SMNKARA08142_8.87E_48.8N_ad_m__Pardosa_palustris
SMNKARA08138_8.87E_48.8N_ad_f__Pardosa_palustris
SMNKARA08140_8.87E_48.8N_ad_m__Pardosa_palustris
ZFMKTIS2479_13.6E_51.18N_ad_f__Pardosa_palustris
ZSMARACH00717_13.28E_52.03N_ad_m__Pardosa_palustris
ZSMARACH00465_11.81E_51.5N_ad_m__Pardosa_palustris
ZFMKTIS22271_13.45E_54.32N_ad_m__Pardosa_palustris
ZFMKTIS18398_12.7E_51.58N_ad_m__Pardosa_palustris
ZFMKTIS18355_13.45E_54.32N_ad_f__Pardosa_palustris
SMNKARA08150_8.87E_48.8N_ad_m__Pardosa_palustris
SMNKARA08148_8.87E_48.8N_ad_m__Pardosa_palustris
SMNKARA08145_8.87E_48.8N_ad_m__Pardosa_palustris
SMNKARA08144_8.87E_48.8N_ad_m__Pardosa_palustris
SMNKARA08141_8.87E_48.8N_ad_m__Pardosa_palustris
SMNKARA08143_8.87E_48.8N_ad_m__Pardosa_palustris
ZSMARACH00499_11.01E_52.51N_ad_f__Pardosa_paludicola
ZSMARACH00657_14.48E_52.47N_ad_m__Pardosa_paludicola
ZFMKTIS22185_13.4E_54.28N_ad_m__Pardosa_amentata
ZFMKTIS2824_13.4E_54.28N_ad_m__Pardosa_amentata
ZFMKTIS7116_9.27E_54.24N_ad_f__Pardosa_amentata
ZFMKTIS2415_13.69E_54.31N_ad_m__Pardosa_amentata
SMNSAran1094_9.95E_48.73N_sbad_f__Pardosa_amentata
ZFMKTIS15919_12.81E_47.14N_ad_m__Pardosa_amentata
ZFMKTIS2505034_11.38E_47.29N_ad_f__Pardosa_amentata
ZSMARACH00422_10.97E_52.49N_ad_f__Pardosa_amentata
0.02

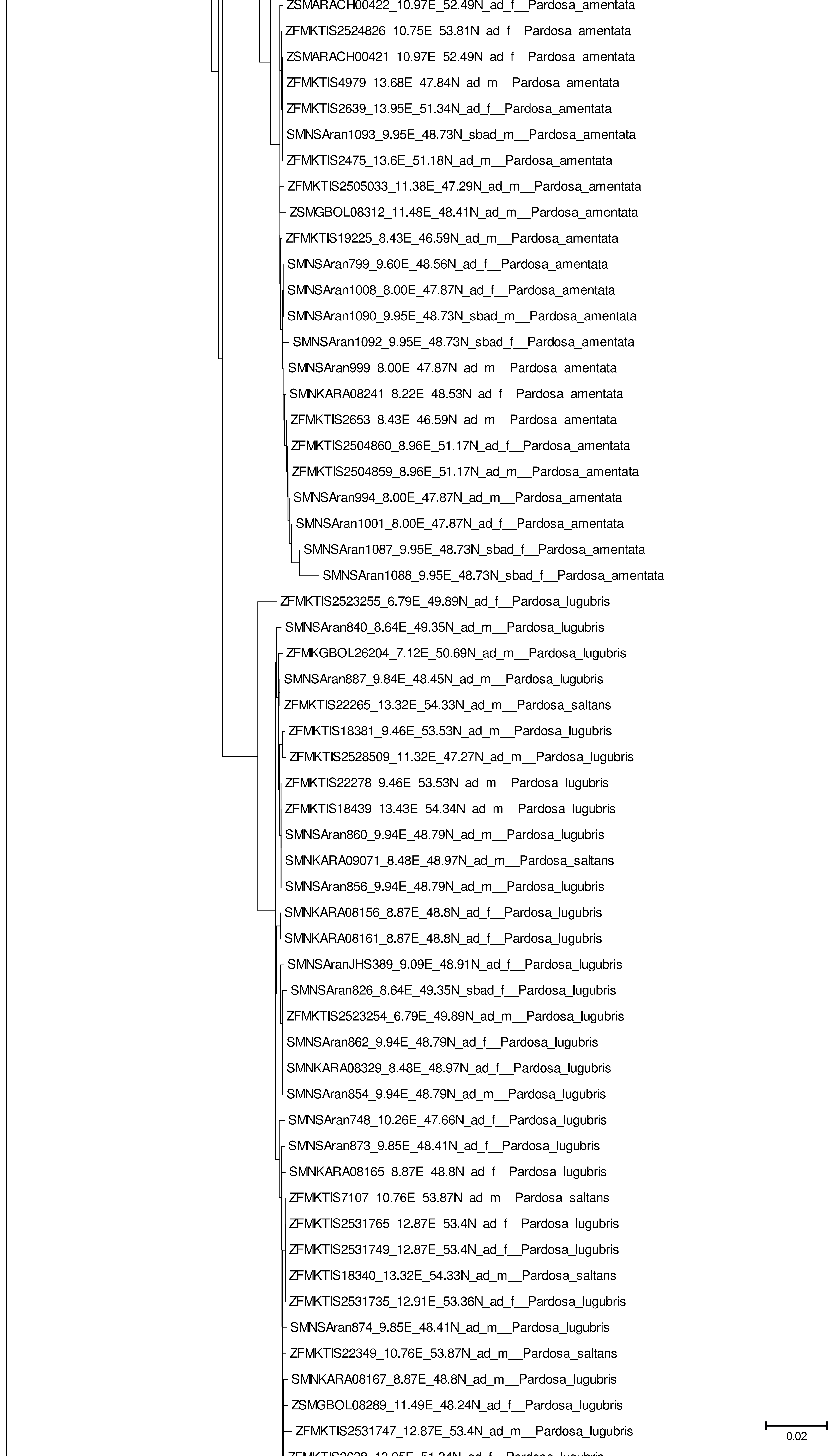
ZSMARACH00422_10.97E_52.49N_ad_f__Pardosa_amentata
ZFMKTIS2524826_10.75E_53.81N_ad_f__Pardosa_amentata
ZSMARACH00421_10.97E_52.49N_ad_f__Pardosa_amentata
ZFMKTIS4979_13.68E_47.84N_ad_m__Pardosa_amentata
ZFMKTIS2639_13.95E_51.34N_ad_f__Pardosa_amentata
SMNSAran1093_9.95E_48.73N_sbad_m__Pardosa_amentata
ZFMKTIS2475_13.6E_51.18N_ad_m__Pardosa_amentata
ZFMKTIS2505033_11.38E_47.29N_ad_m__Pardosa_amentata
ZSMGBOL08312_11.48E_48.41N_ad_m__Pardosa_amentata
ZFMKTIS19225_8.43E_46.59N_ad_m__Pardosa_amentata
SMNSAran799_9.60E_48.56N_ad_f__Pardosa_amentata
SMNSAran1008_8.00E_47.87N_ad_f__Pardosa_amentata
SMNSAran1090_9.95E_48.73N_sbad_m__Pardosa_amentata
SMNSAran1092_9.95E_48.73N_sbad_f__Pardosa_amentata
SMNSAran999_8.00E_47.87N_ad_m__Pardosa_amentata
SMNKARA08241_8.22E_48.53N_ad_f__Pardosa_amentata
ZFMKTIS2653_8.43E_46.59N_ad_m__Pardosa_amentata
ZFMKTIS2504860_8.96E_51.17N_ad_f__Pardosa_amentata
ZFMKTIS2504859_8.96E_51.17N_ad_m__Pardosa_amentata
SMNSAran994_8.00E_47.87N_ad_m__Pardosa_amentata
SMNSAran1001_8.00E_47.87N_ad_f__Pardosa_amentata
SMNSAran1087_9.95E_48.73N_sbad_f__Pardosa_amentata
SMNSAran1088_9.95E_48.73N_sbad_f__Pardosa_amentata
ZFMKTIS2523255_6.79E_49.89N_ad_f__Pardosa_lugubris
SMNSAran840_8.64E_49.35N_ad_m__Pardosa_lugubris
ZFMKGBOL26204_7.12E_50.69N_ad_m__Pardosa_lugubris
SMNSAran887_9.84E_48.45N_ad_m__Pardosa_lugubris
ZFMKTIS22265_13.32E_54.33N_ad_m__Pardosa_saltans
ZFMKTIS18381_9.46E_53.53N_ad_m__Pardosa_lugubris
ZFMKTIS2528509_11.32E_47.27N_ad_m__Pardosa_lugubris
ZFMKTIS22278_9.46E_53.53N_ad_m__Pardosa_lugubris
ZFMKTIS18439_13.43E_54.34N_ad_m__Pardosa_lugubris
SMNSAran860_9.94E_48.79N_ad_m__Pardosa_lugubris
SMNKARA09071_8.48E_48.97N_ad_m__Pardosa_saltans
SMNSAran856_9.94E_48.79N_ad_m__Pardosa_lugubris
SMNKARA08156_8.87E_48.8N_ad_f__Pardosa_lugubris
SMNKARA08161_8.87E_48.8N_ad_f__Pardosa_lugubris
SMNSAranJHS389_9.09E_48.91N_ad_f__Pardosa_lugubris
SMNSAran826_8.64E_49.35N_sbad_f__Pardosa_lugubris
ZFMKTIS2523254_6.79E_49.89N_ad_m__Pardosa_lugubris
SMNSAran862_9.94E_48.79N_ad_f__Pardosa_lugubris
SMNKARA08329_8.48E_48.97N_ad_f__Pardosa_lugubris
SMNSAran854_9.94E_48.79N_ad_m__Pardosa_lugubris
SMNSAran748_10.26E_47.66N_ad_f__Pardosa_lugubris
SMNSAran873_9.85E_48.41N_ad_f__Pardosa_lugubris
SMNKARA08165_8.87E_48.8N_ad_f__Pardosa_lugubris
ZFMKTIS7107_10.76E_53.87N_ad_m__Pardosa_saltans
ZFMKTIS2531765_12.87E_53.4N_ad_f__Pardosa_lugubris
ZFMKTIS2531749_12.87E_53.4N_ad_f__Pardosa_lugubris
ZFMKTIS18340_13.32E_54.33N_ad_m__Pardosa_saltans
ZFMKTIS2531735_12.91E_53.36N_ad_f__Pardosa_lugubris
SMNSAran874_9.85E_48.41N_ad_m__Pardosa_lugubris
ZFMKTIS22349_10.76E_53.87N_ad_m__Pardosa_saltans
SMNKARA08167_8.87E_48.8N_ad_m__Pardosa_lugubris
ZSMGBOL08289_11.49E_48.24N_ad_f__Pardosa_lugubris
ZFMKTIS2531747_12.87E_53.4N_ad_m__Pardosa_lugubris
0.02

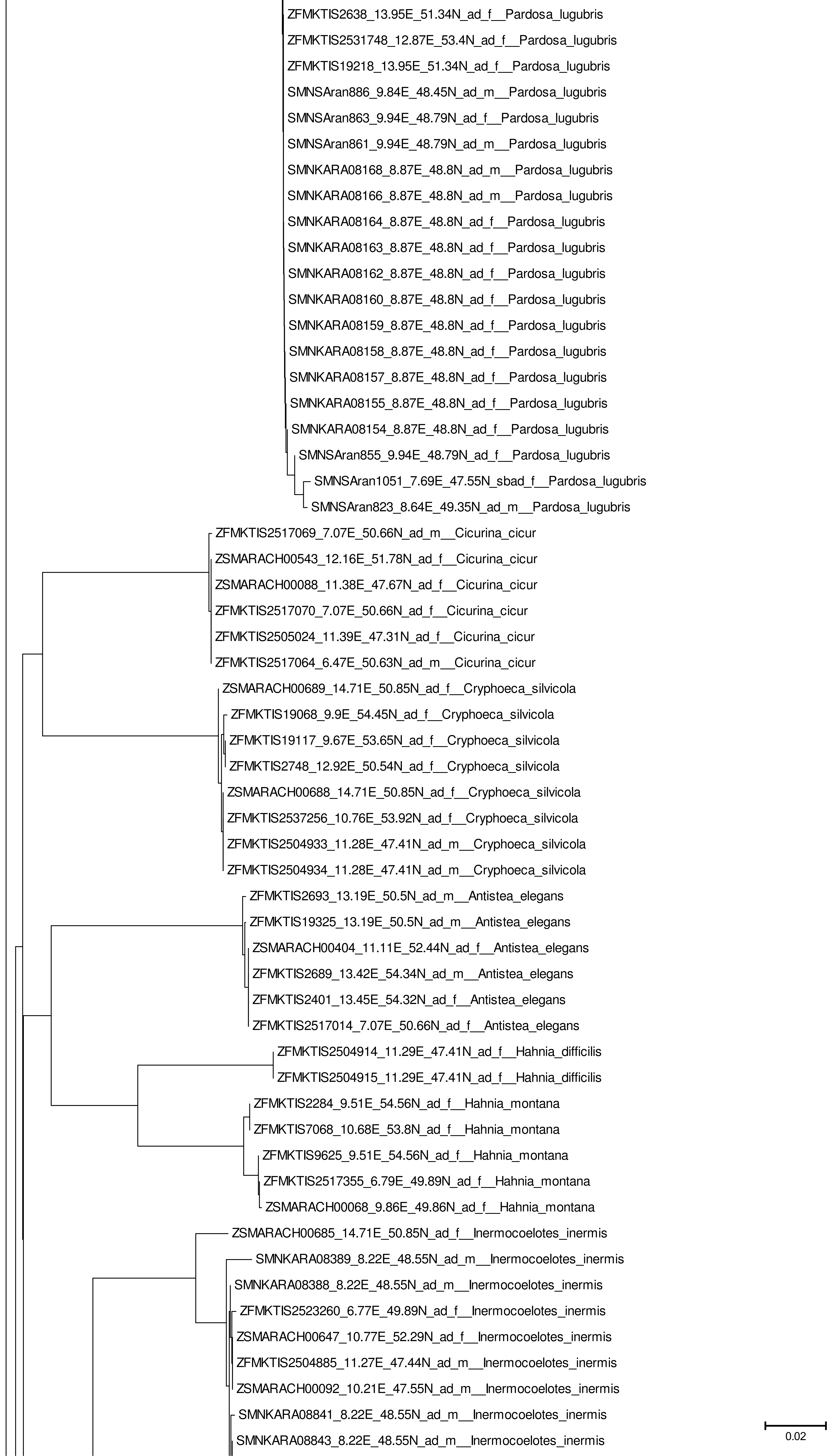

ZFMKTIS2638_13.95E_51.34N_ad_f__Pardosa_lugubris
ZFMKTIS2531748_12.87E_53.4N_ad_f__Pardosa_lugubris
ZFMKTIS19218_13.95E_51.34N_ad_f__Pardosa_lugubris
SMNSAran886_9.84E_48.45N_ad_m__Pardosa_lugubris
SMNSAran863_9.94E_48.79N_ad_f__Pardosa_lugubris
SMNSAran861_9.94E_48.79N_ad_m__Pardosa_lugubris
SMNKARA08168_8.87E_48.8N_ad_m__Pardosa_lugubris
SMNKARA08166_8.87E_48.8N_ad_m__Pardosa_lugubris
SMNKARA08164_8.87E_48.8N_ad_f__Pardosa_lugubris
SMNKARA08163_8.87E_48.8N_ad_f__Pardosa_lugubris
SMNKARA08162_8.87E_48.8N_ad_f__Pardosa_lugubris
SMNKARA08160_8.87E_48.8N_ad_f__Pardosa_lugubris
SMNKARA08159_8.87E_48.8N_ad_f__Pardosa_lugubris
SMNKARA08158_8.87E_48.8N_ad_f__Pardosa_lugubris
SMNKARA08157_8.87E_48.8N_ad_f__Pardosa_lugubris
SMNKARA08155_8.87E_48.8N_ad_f__Pardosa_lugubris
SMNKARA08154_8.87E_48.8N_ad_f__Pardosa_lugubris
SMNSAran855_9.94E_48.79N_ad_f__Pardosa_lugubris
SMNSAran1051_7.69E_47.55N_sbad_f__Pardosa_lugubris
SMNSAran823_8.64E_49.35N_ad_m__Pardosa_lugubris
ZFMKTIS2517069_7.07E_50.66N_ad_m__Cicurina_cicur
ZSMARACH00543_12.16E_51.78N_ad_f__Cicurina_cicur
ZSMARACH00088_11.38E_47.67N_ad_f__Cicurina_cicur
ZFMKTIS2517070_7.07E_50.66N_ad_f__Cicurina_cicur
ZFMKTIS2505024_11.39E_47.31N_ad_f__Cicurina_cicur
ZFMKTIS2517064_6.47E_50.63N_ad_m__Cicurina_cicur
ZSMARACH00689_14.71E_50.85N_ad_f__Cryphoeca_silvicola
ZFMKTIS19068_9.9E_54.45N_ad_f__Cryphoeca_silvicola
ZFMKTIS19117_9.67E_53.65N_ad_f__Cryphoeca_silvicola
ZFMKTIS2748_12.92E_50.54N_ad_f__Cryphoeca_silvicola
ZSMARACH00688_14.71E_50.85N_ad_f__Cryphoeca_silvicola
ZFMKTIS2537256_10.76E_53.92N_ad_f__Cryphoeca_silvicola
ZFMKTIS2504933_11.28E_47.41N_ad_m__Cryphoeca_silvicola
ZFMKTIS2504934_11.28E_47.41N_ad_m__Cryphoeca_silvicola
ZFMKTIS2693_13.19E_50.5N_ad_m__Antistea_elegans
ZFMKTIS19325_13.19E_50.5N_ad_m__Antistea_elegans
ZSMARACH00404_11.11E_52.44N_ad_f__Antistea_elegans
ZFMKTIS2689_13.42E_54.34N_ad_m__Antistea_elegans
ZFMKTIS2401_13.45E_54.32N_ad_f__Antistea_elegans
ZFMKTIS2517014_7.07E_50.66N_ad_f__Antistea_elegans
ZFMKTIS2504914_11.29E_47.41N_ad_f__Hahnia_difficilis
ZFMKTIS2504915_11.29E_47.41N_ad_f__Hahnia_difficilis
ZFMKTIS2284_9.51E_54.56N_ad_f__Hahnia_montana
ZFMKTIS7068_10.68E_53.8N_ad_f__Hahnia_montana
ZFMKTIS9625_9.51E_54.56N_ad_f__Hahnia_montana
ZFMKTIS2517355_6.79E_49.89N_ad_f__Hahnia_montana
ZSMARACH00068_9.86E_49.86N_ad_f__Hahnia_montana
ZSMARACH00685_14.71E_50.85N_ad_f__Inermocoelotes_inermis
SMNKARA08389_8.22E_48.55N_ad_m__Inermocoelotes_inermis
SMNKARA08388_8.22E_48.55N_ad_m__Inermocoelotes_inermis
ZFMKTIS2523260_6.77E_49.89N_ad_f__Inermocoelotes_inermis
ZSMARACH00647_10.77E_52.29N_ad_f__Inermocoelotes_inermis
ZFMKTIS2504885_11.27E_47.44N_ad_m__Inermocoelotes_inermis
ZSMARACH00092_10.21E_47.55N_ad_m__Inermocoelotes_inermis
SMNKARA08841_8.22E_48.55N_ad_m__Inermocoelotes_inermis
SMNKARA08843_8.22E_48.55N_ad_m__Inermocoelotes_inermis
0.02

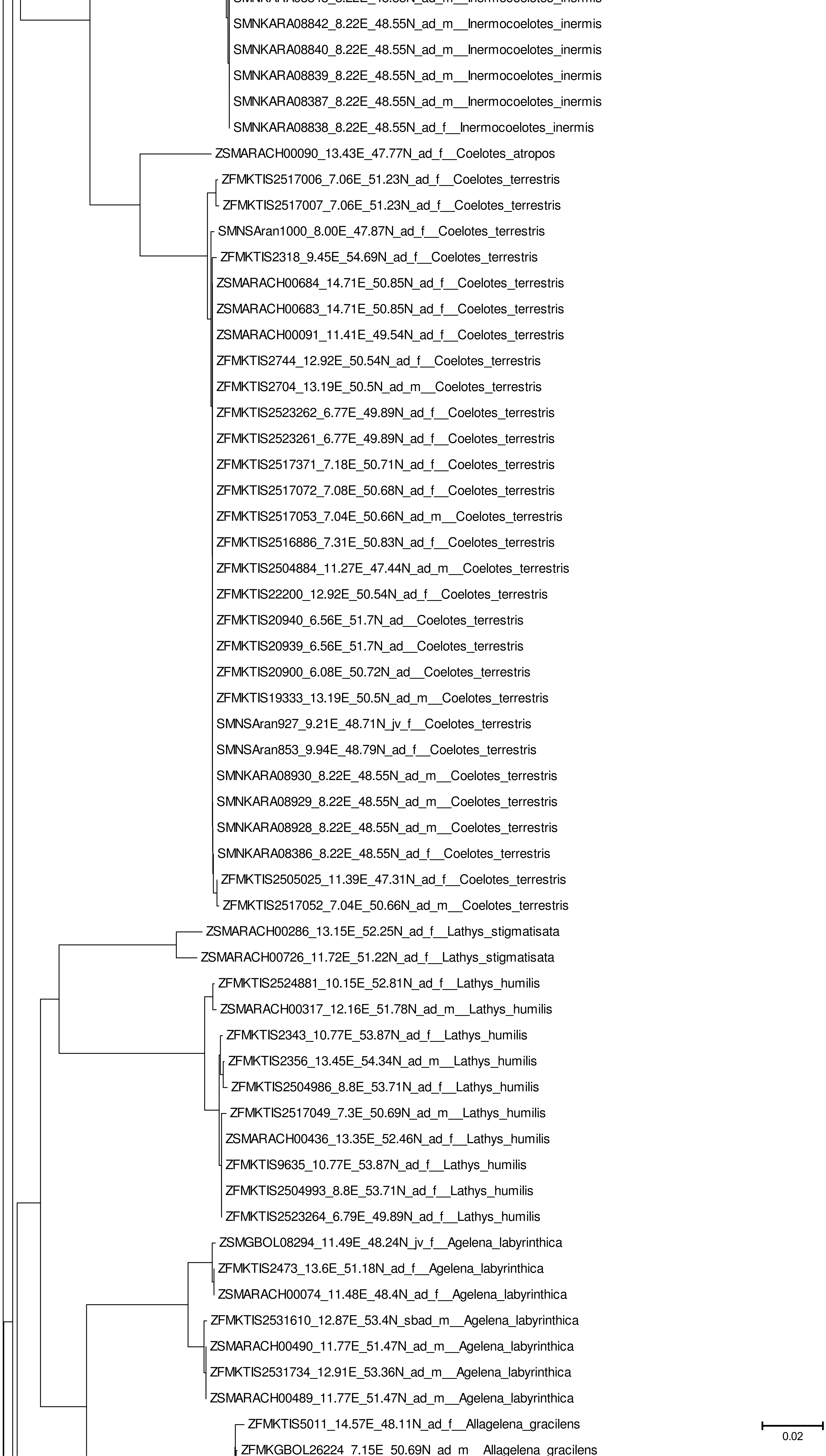
SMNKARA08842_8.22E_48.55N_ad_m__Inermocoelotes_inermis
SMNKARA08840_8.22E_48.55N_ad_m__Inermocoelotes_inermis
SMNKARA08839_8.22E_48.55N_ad_m__Inermocoelotes_inermis
SMNKARA08387_8.22E_48.55N_ad_m__Inermocoelotes_inermis
SMNKARA08838_8.22E_48.55N_ad_f__Inermocoelotes_inermis
ZSMARACH00090_13.43E_47.77N_ad_f__Coelotes_atropos
ZFMKTIS2517006_7.06E_51.23N_ad_f__Coelotes_terrestris
ZFMKTIS2517007_7.06E_51.23N_ad_f__Coelotes_terrestris
SMNSAran1000_8.00E_47.87N_ad_f__Coelotes_terrestris
ZFMKTIS2318_9.45E_54.69N_ad_f__Coelotes_terrestris
ZSMARACH00684_14.71E_50.85N_ad_f__Coelotes_terrestris
ZSMARACH00683_14.71E_50.85N_ad_f__Coelotes_terrestris
ZSMARACH00091_11.41E_49.54N_ad_f__Coelotes_terrestris
ZFMKTIS2744_12.92E_50.54N_ad_f__Coelotes_terrestris
ZFMKTIS2704_13.19E_50.5N_ad_m__Coelotes_terrestris
ZFMKTIS2523262_6.77E_49.89N_ad_f__Coelotes_terrestris
ZFMKTIS2523261_6.77E_49.89N_ad_f__Coelotes_terrestris
ZFMKTIS2517371_7.18E_50.71N_ad_f__Coelotes_terrestris
ZFMKTIS2517072_7.08E_50.68N_ad_f__Coelotes_terrestris
ZFMKTIS2517053_7.04E_50.66N_ad_m__Coelotes_terrestris
ZFMKTIS2516886_7.31E_50.83N_ad_f__Coelotes_terrestris
ZFMKTIS2504884_11.27E_47.44N_ad_m__Coelotes_terrestris
ZFMKTIS22200_12.92E_50.54N_ad_f__Coelotes_terrestris
ZFMKTIS20940_6.56E_51.7N_ad__Coelotes_terrestris
ZFMKTIS20939_6.56E_51.7N_ad__Coelotes_terrestris
ZFMKTIS20900_6.08E_50.72N_ad__Coelotes_terrestris
ZFMKTIS19333_13.19E_50.5N_ad_m__Coelotes_terrestris
SMNSAran927_9.21E_48.71N_jv_f__Coelotes_terrestris
SMNSAran853_9.94E_48.79N_ad_f__Coelotes_terrestris
SMNKARA08930_8.22E_48.55N_ad_m__Coelotes_terrestris
SMNKARA08929_8.22E_48.55N_ad_m__Coelotes_terrestris
SMNKARA08928_8.22E_48.55N_ad_m__Coelotes_terrestris
SMNKARA08386_8.22E_48.55N_ad_f__Coelotes_terrestris
ZFMKTIS2505025_11.39E_47.31N_ad_f__Coelotes_terrestris
ZFMKTIS2517052_7.04E_50.66N_ad_m__Coelotes_terrestris
ZSMARACH00286_13.15E_52.25N_ad_f__Lathys_stigmatisata
ZSMARACH00726_11.72E_51.22N_ad_f__Lathys_stigmatisata
ZFMKTIS2524881_10.15E_52.81N_ad_f__Lathys_humilis
ZSMARACH00317_12.16E_51.78N_ad_m__Lathys_humilis
ZFMKTIS2343_10.77E_53.87N_ad_f__Lathys_humilis
ZFMKTIS2356_13.45E_54.34N_ad_m__Lathys_humilis
ZFMKTIS2504986_8.8E_53.71N_ad_f__Lathys_humilis
ZFMKTIS2517049_7.3E_50.69N_ad_m__Lathys_humilis
ZSMARACH00436_13.35E_52.46N_ad_f__Lathys_humilis
ZFMKTIS9635_10.77E_53.87N_ad_f__Lathys_humilis
ZFMKTIS2504993_8.8E_53.71N_ad_f__Lathys_humilis
ZFMKTIS2523264_6.79E_49.89N_ad_f__Lathys_humilis
ZSMGBOL08294_11.49E_48.24N_jv_f__Agelena_labyrinthica
ZFMKTIS2473_13.6E_51.18N_ad_f__Agelena_labyrinthica
ZSMARACH00074_11.48E_48.4N_ad_f__Agelena_labyrinthica
ZFMKTIS2531610_12.87E_53.4N_sbad_m__Agelena_labyrinthica
ZSMARACH00490_11.77E_51.47N_ad_m__Agelena_labyrinthica
ZFMKTIS2531734_12.91E_53.36N_ad_m__Agelena_labyrinthica
ZSMARACH00489_11.77E_51.47N_ad_m__Agelena_labyrinthica
ZFMKTIS5011_14.57E_48.11N_ad_f__Allagelena_gracilens
ZFMKGBOL26224_7.15E_50.69N_ad_m__Allagelena_gracilens
0.02

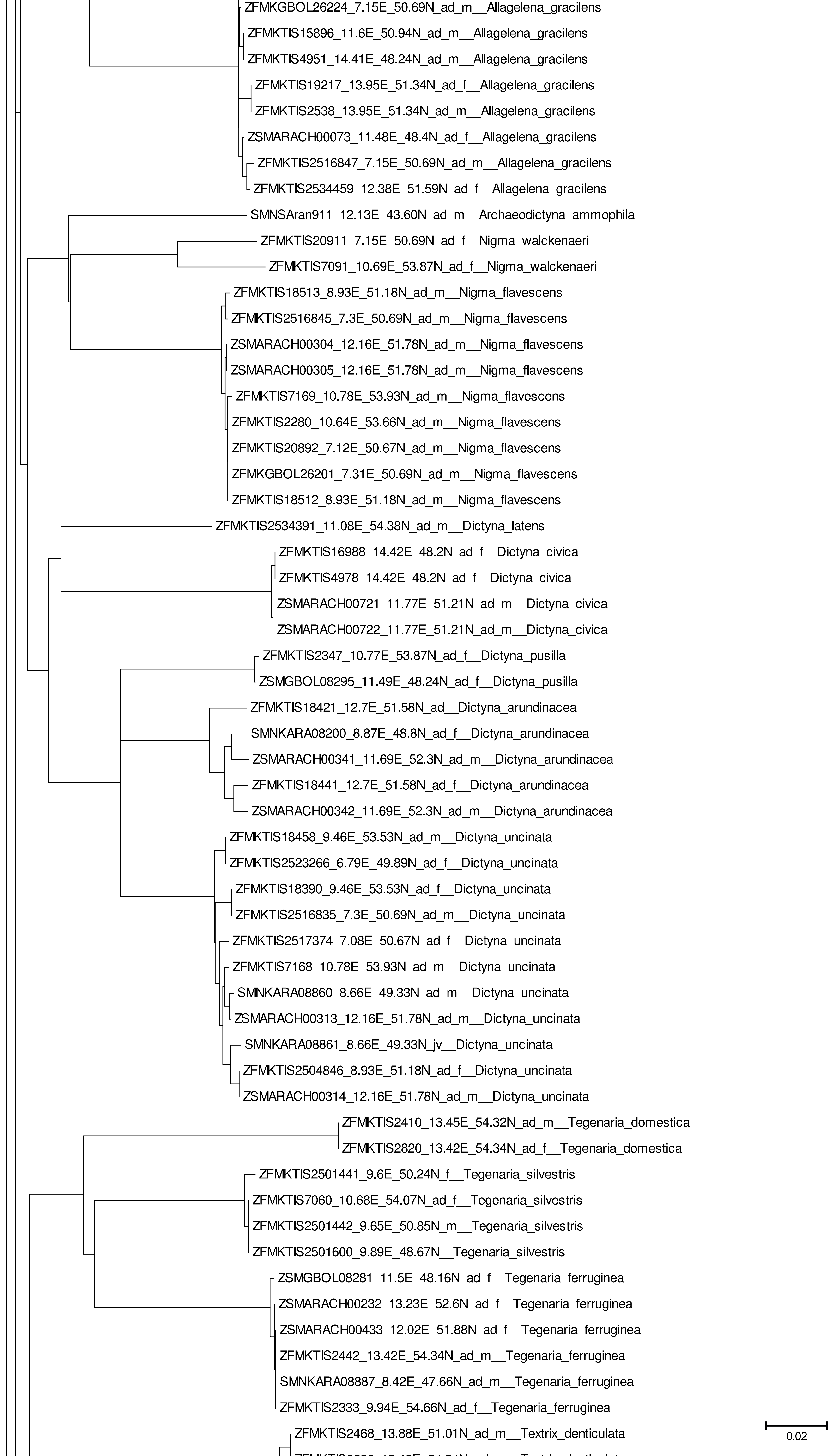
ZFMKGBOL26224_7.15E_50.69N_ad_m__Allagelena_gracilens
ZFMKTIS15896_11.6E_50.94N_ad_m__Allagelena_gracilens
ZFMKTIS4951_14.41E_48.24N_ad_m__Allagelena_gracilens
ZFMKTIS19217_13.95E_51.34N_ad_f__Allagelena_gracilens
ZFMKTIS2538_13.95E_51.34N_ad_m__Allagelena_gracilens
ZSMARACH00073_11.48E_48.4N_ad_f__Allagelena_gracilens
ZFMKTIS2516847_7.15E_50.69N_ad_m__Allagelena_gracilens
ZFMKTIS2534459_12.38E_51.59N_ad_f__Allagelena_gracilens
SMNSAran911_12.13E_43.60N_ad_m__Archaeodictyna_ammophila
ZFMKTIS20911_7.15E_50.69N_ad_f__Nigma_walckenaeri
ZFMKTIS7091_10.69E_53.87N_ad_f__Nigma_walckenaeri
ZFMKTIS18513_8.93E_51.18N_ad_m__Nigma_flavescens
ZFMKTIS2516845_7.3E_50.69N_ad_m__Nigma_flavescens
ZSMARACH00304_12.16E_51.78N_ad_m__Nigma_flavescens
ZSMARACH00305_12.16E_51.78N_ad_m__Nigma_flavescens
ZFMKTIS7169_10.78E_53.93N_ad_m__Nigma_flavescens
ZFMKTIS2280_10.64E_53.66N_ad_m__Nigma_flavescens
ZFMKTIS20892_7.12E_50.67N_ad_m__Nigma_flavescens
ZFMKGBOL26201_7.31E_50.69N_ad_m__Nigma_flavescens
ZFMKTIS18512_8.93E_51.18N_ad_m__Nigma_flavescens
ZFMKTIS2534391_11.08E_54.38N_ad_m__Dictyna_latens
ZFMKTIS16988_14.42E_48.2N_ad_f__Dictyna_civica
ZFMKTIS4978_14.42E_48.2N_ad_f__Dictyna_civica
ZSMARACH00721_11.77E_51.21N_ad_m__Dictyna_civica
ZSMARACH00722_11.77E_51.21N_ad_m__Dictyna_civica
ZFMKTIS2347_10.77E_53.87N_ad_f__Dictyna_pusilla
ZSMGBOL08295_11.49E_48.24N_ad_f__Dictyna_pusilla
ZFMKTIS18421_12.7E_51.58N_ad__Dictyna_arundinacea
SMNKARA08200_8.87E_48.8N_ad_f__Dictyna_arundinacea
ZSMARACH00341_11.69E_52.3N_ad_m__Dictyna_arundinacea
ZFMKTIS18441_12.7E_51.58N_ad_f__Dictyna_arundinacea
ZSMARACH00342_11.69E_52.3N_ad_m__Dictyna_arundinacea
ZFMKTIS18458_9.46E_53.53N_ad_m__Dictyna_uncinata
ZFMKTIS2523266_6.79E_49.89N_ad_f__Dictyna_uncinata
ZFMKTIS18390_9.46E_53.53N_ad_f__Dictyna_uncinata
ZFMKTIS2516835_7.3E_50.69N_ad_m__Dictyna_uncinata
ZFMKTIS2517374_7.08E_50.67N_ad_f__Dictyna_uncinata
ZFMKTIS7168_10.78E_53.93N_ad_m__Dictyna_uncinata
SMNKARA08860_8.66E_49.33N_ad_m__Dictyna_uncinata
ZSMARACH00313_12.16E_51.78N_ad_m__Dictyna_uncinata
SMNKARA08861_8.66E_49.33N_jv__Dictyna_uncinata
ZFMKTIS2504846_8.93E_51.18N_ad_f__Dictyna_uncinata
ZSMARACH00314_12.16E_51.78N_ad_m__Dictyna_uncinata
ZFMKTIS2410_13.45E_54.32N_ad_m__Tegenaria_domestica
ZFMKTIS2820_13.42E_54.34N_ad_f__Tegenaria_domestica
ZFMKTIS2501441_9.6E_50.24N_f__Tegenaria_silvestris
ZFMKTIS7060_10.68E_54.07N_ad_f__Tegenaria_silvestris
ZFMKTIS2501442_9.65E_50.85N_m__Tegenaria_silvestris
ZFMKTIS2501600_9.89E_48.67N__Tegenaria_silvestris
ZSMGBOL08281_11.5E_48.16N_ad_f__Tegenaria_ferruginea
ZSMARACH00232_13.23E_52.6N_ad_f__Tegenaria_ferruginea
ZSMARACH00433_12.02E_51.88N_ad_f__Tegenaria_ferruginea
ZFMKTIS2442_13.42E_54.34N_ad_m__Tegenaria_ferruginea
SMNKARA08887_8.42E_47.66N_ad_m__Tegenaria_ferruginea
ZFMKTIS2333_9.94E_54.66N_ad_f__Tegenaria_ferruginea
ZFMKTIS2468_13.88E_51.01N_ad_m__Textrix_denticulata
0.02

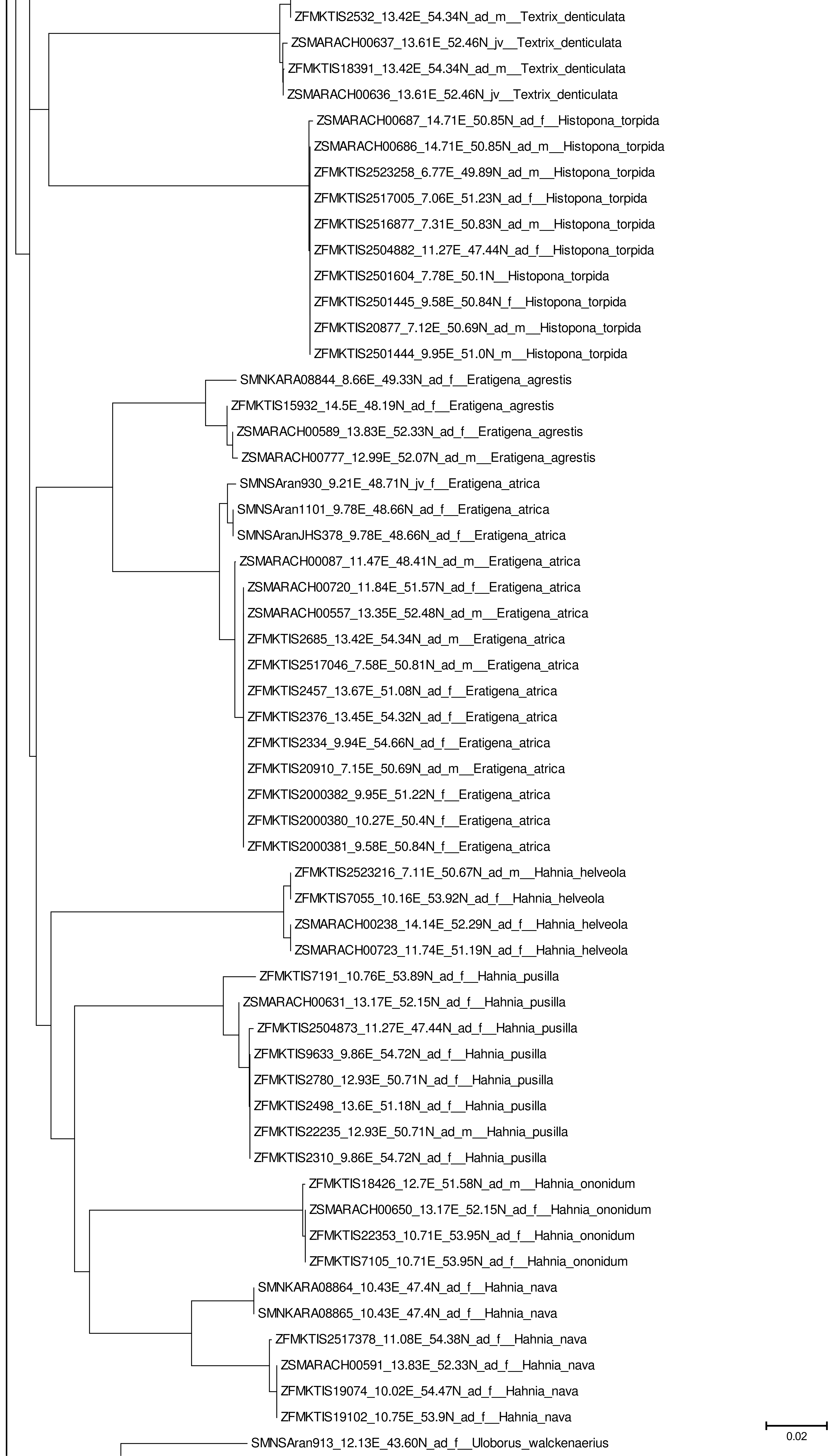
ZFMKTIS2532_13.42E_54.34N_ad_m__Textrix_denticulata
ZSMARACH00637_13.61E_52.46N_jv__Textrix_denticulata
ZFMKTIS18391_13.42E_54.34N_ad_m__Textrix_denticulata
ZSMARACH00636_13.61E_52.46N_jv__Textrix_denticulata
ZSMARACH00687_14.71E_50.85N_ad_f__Histopona_torpida
ZSMARACH00686_14.71E_50.85N_ad_m__Histopona_torpida
ZFMKTIS2523258_6.77E_49.89N_ad_m__Histopona_torpida
ZFMKTIS2517005_7.06E_51.23N_ad_f__Histopona_torpida
ZFMKTIS2516877_7.31E_50.83N_ad_m__Histopona_torpida
ZFMKTIS2504882_11.27E_47.44N_ad_f__Histopona_torpida
ZFMKTIS2501604_7.78E_50.1N__Histopona_torpida
ZFMKTIS2501445_9.58E_50.84N_f__Histopona_torpida
ZFMKTIS20877_7.12E_50.69N_ad_m__Histopona_torpida
ZFMKTIS2501444_9.95E_51.0N_m__Histopona_torpida
SMNKARA08844_8.66E_49.33N_ad_f__Eratigena_agrestis
ZFMKTIS15932_14.5E_48.19N_ad_f__Eratigena_agrestis
ZSMARACH00589_13.83E_52.33N_ad_f__Eratigena_agrestis
ZSMARACH00777_12.99E_52.07N_ad_m__Eratigena_agrestis
SMNSAran930_9.21E_48.71N_jv_f__Eratigena_atrica
SMNSAran1101_9.78E_48.66N_ad_f__Eratigena_atrica
SMNSAranJHS378_9.78E_48.66N_ad_f__Eratigena_atrica
ZSMARACH00087_11.47E_48.41N_ad_m__Eratigena_atrica
ZSMARACH00720_11.84E_51.57N_ad_f__Eratigena_atrica
ZSMARACH00557_13.35E_52.48N_ad_m__Eratigena_atrica
ZFMKTIS2685_13.42E_54.34N_ad_m__Eratigena_atrica
ZFMKTIS2517046_7.58E_50.81N_ad_m__Eratigena_atrica
ZFMKTIS2457_13.67E_51.08N_ad_f__Eratigena_atrica
ZFMKTIS2376_13.45E_54.32N_ad_f__Eratigena_atrica
ZFMKTIS2334_9.94E_54.66N_ad_f__Eratigena_atrica
ZFMKTIS20910_7.15E_50.69N_ad_m__Eratigena_atrica
ZFMKTIS2000382_9.95E_51.22N_f__Eratigena_atrica
ZFMKTIS2000380_10.27E_50.4N_f__Eratigena_atrica
ZFMKTIS2000381_9.58E_50.84N_f__Eratigena_atrica
ZFMKTIS2523216_7.11E_50.67N_ad_m__Hahnia_helveola
ZFMKTIS7055_10.16E_53.92N_ad_f__Hahnia_helveola
ZSMARACH00238_14.14E_52.29N_ad_f__Hahnia_helveola
ZSMARACH00723_11.74E_51.19N_ad_f__Hahnia_helveola
ZFMKTIS7191_10.76E_53.89N_ad_f__Hahnia_pusilla
ZSMARACH00631_13.17E_52.15N_ad_f__Hahnia_pusilla
ZFMKTIS2504873_11.27E_47.44N_ad_f__Hahnia_pusilla
ZFMKTIS9633_9.86E_54.72N_ad_f__Hahnia_pusilla
ZFMKTIS2780_12.93E_50.71N_ad_f__Hahnia_pusilla
ZFMKTIS2498_13.6E_51.18N_ad_f__Hahnia_pusilla
ZFMKTIS22235_12.93E_50.71N_ad_m__Hahnia_pusilla
ZFMKTIS2310_9.86E_54.72N_ad_f__Hahnia_pusilla
ZFMKTIS18426_12.7E_51.58N_ad_m__Hahnia_ononidum
ZSMARACH00650_13.17E_52.15N_ad_f__Hahnia_ononidum
ZFMKTIS22353_10.71E_53.95N_ad_f__Hahnia_ononidum
ZFMKTIS7105_10.71E_53.95N_ad_f__Hahnia_ononidum
SMNKARA08864_10.43E_47.4N_ad_f__Hahnia_nava
SMNKARA08865_10.43E_47.4N_ad_f__Hahnia_nava
ZFMKTIS2517378_11.08E_54.38N_ad_f__Hahnia_nava
ZSMARACH00591_13.83E_52.33N_ad_f__Hahnia_nava
ZFMKTIS19074_10.02E_54.47N_ad_f__Hahnia_nava
ZFMKTIS19102_10.75E_53.9N_ad_f__Hahnia_nava
0.02
SMNSAran913_12.13E_43.60N_ad_f__Uloborus_walckenaerius

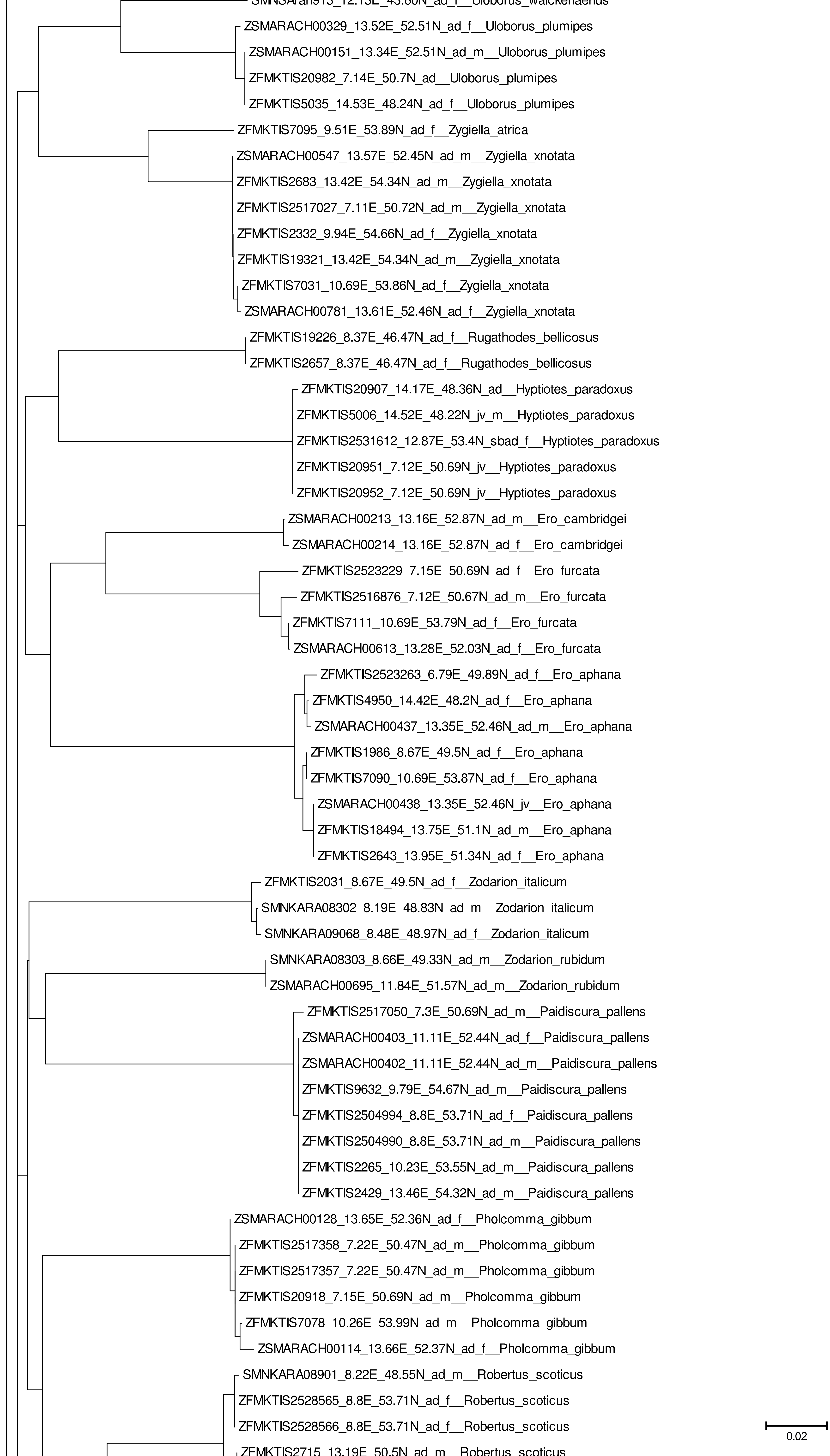
SMNSAran913_12.13E_45.60N_ad_f__Uloborus_walckenaerius
ZSMARACH00329_13.52E_52.51N_ad_f__Uloborus_plumipes
ZSMARACH00151_13.34E_52.51N_ad_m__Uloborus_plumipes
ZFMKTIS20982_7.14E_50.7N_ad__Uloborus_plumipes
ZFMKTIS5035_14.53E_48.24N_ad_f__Uloborus_plumipes
ZFMKTIS7095_9.51E_53.89N_ad_f__Zygiella_atrica
ZSMARACH00547_13.57E_52.45N_ad_m__Zygiella_xnotata
ZFMKTIS2683_13.42E_54.34N_ad_m__Zygiella_xnotata
ZFMKTIS2517027_7.11E_50.72N_ad_m__Zygiella_xnotata
ZFMKTIS2332_9.94E_54.66N_ad_f__Zygiella_xnotata
ZFMKTIS19321_13.42E_54.34N_ad_m__Zygiella_xnotata
ZFMKTIS7031_10.69E_53.86N_ad_f__Zygiella_xnotata
ZSMARACH00781_13.61E_52.46N_ad_f__Zygiella_xnotata
ZFMKTIS19226_8.37E_46.47N_ad_f__Rugathodes_bellicosus
ZFMKTIS2657_8.37E_46.47N_ad_f__Rugathodes_bellicosus
ZFMKTIS20907_14.17E_48.36N_ad__Hyptiotes_paradoxus
ZFMKTIS5006_14.52E_48.22N_jv_m__Hyptiotes_paradoxus
ZFMKTIS2531612_12.87E_53.4N_sbad_f__Hyptiotes_paradoxus
ZFMKTIS20951_7.12E_50.69N_jv__Hyptiotes_paradoxus
ZFMKTIS20952_7.12E_50.69N_jv__Hyptiotes_paradoxus
ZSMARACH00213_13.16E_52.87N_ad_m__Ero_cambridgei
ZSMARACH00214_13.16E_52.87N_ad_f__Ero_cambridgei
ZFMKTIS2523229_7.15E_50.69N_ad_f__Ero_furcata
ZFMKTIS2516876_7.12E_50.67N_ad_m__Ero_furcata
ZFMKTIS7111_10.69E_53.79N_ad_f__Ero_furcata
ZSMARACH00613_13.28E_52.03N_ad_f__Ero_furcata
ZFMKTIS2523263_6.79E_49.89N_ad_f__Ero_aphana
ZFMKTIS4950_14.42E_48.2N_ad_f__Ero_aphana
ZSMARACH00437_13.35E_52.46N_ad_m__Ero_aphana
ZFMKTIS1986_8.67E_49.5N_ad_f__Ero_aphana
ZFMKTIS7090_10.69E_53.87N_ad_f__Ero_aphana
ZSMARACH00438_13.35E_52.46N_jv__Ero_aphana
ZFMKTIS18494_13.75E_51.1N_ad_m__Ero_aphana
ZFMKTIS2643_13.95E_51.34N_ad_f__Ero_aphana
ZFMKTIS2031_8.67E_49.5N_ad_f__Zodarion_italicum
SMNKARA08302_8.19E_48.83N_ad_m__Zodarion_italicum
SMNKARA09068_8.48E_48.97N_ad_f__Zodarion_italicum
SMNKARA08303_8.66E_49.33N_ad_m__Zodarion_rubidum
ZSMARACH00695_11.84E_51.57N_ad_m__Zodarion_rubidum
ZFMKTIS2517050_7.3E_50.69N_ad_m__Paidiscura_pallens
ZSMARACH00403_11.11E_52.44N_ad_f__Paidiscura_pallens
ZSMARACH00402_11.11E_52.44N_ad_m__Paidiscura_pallens
ZFMKTIS9632_9.79E_54.67N_ad_m__Paidiscura_pallens
ZFMKTIS2504994_8.8E_53.71N_ad_f__Paidiscura_pallens
ZFMKTIS2504990_8.8E_53.71N_ad_m__Paidiscura_pallens
ZFMKTIS2265_10.23E_53.55N_ad_m__Paidiscura_pallens
ZFMKTIS2429_13.46E_54.32N_ad_m__Paidiscura_pallens
ZSMARACH00128_13.65E_52.36N_ad_f__Pholcomma_gibbum
ZFMKTIS2517358_7.22E_50.47N_ad_m__Pholcomma_gibbum
ZFMKTIS2517357_7.22E_50.47N_ad_m__Pholcomma_gibbum
ZFMKTIS20918_7.15E_50.69N_ad_m__Pholcomma_gibbum
ZFMKTIS7078_10.26E_53.99N_ad_m__Pholcomma_gibbum
ZSMARACH00114_13.66E_52.37N_ad_f__Pholcomma_gibbum
SMNKARA08901_8.22E_48.55N_ad_m__Robertus_scoticus
ZFMKTIS2528565_8.8E_53.71N_ad_f__Robertus_scoticus
ZFMKTIS2528566_8.8E_53.71N_ad_f__Robertus_scoticus
ZFMKTIS2715_13.19E_50.5N_ad_m__Robertus_scoticus
0.02

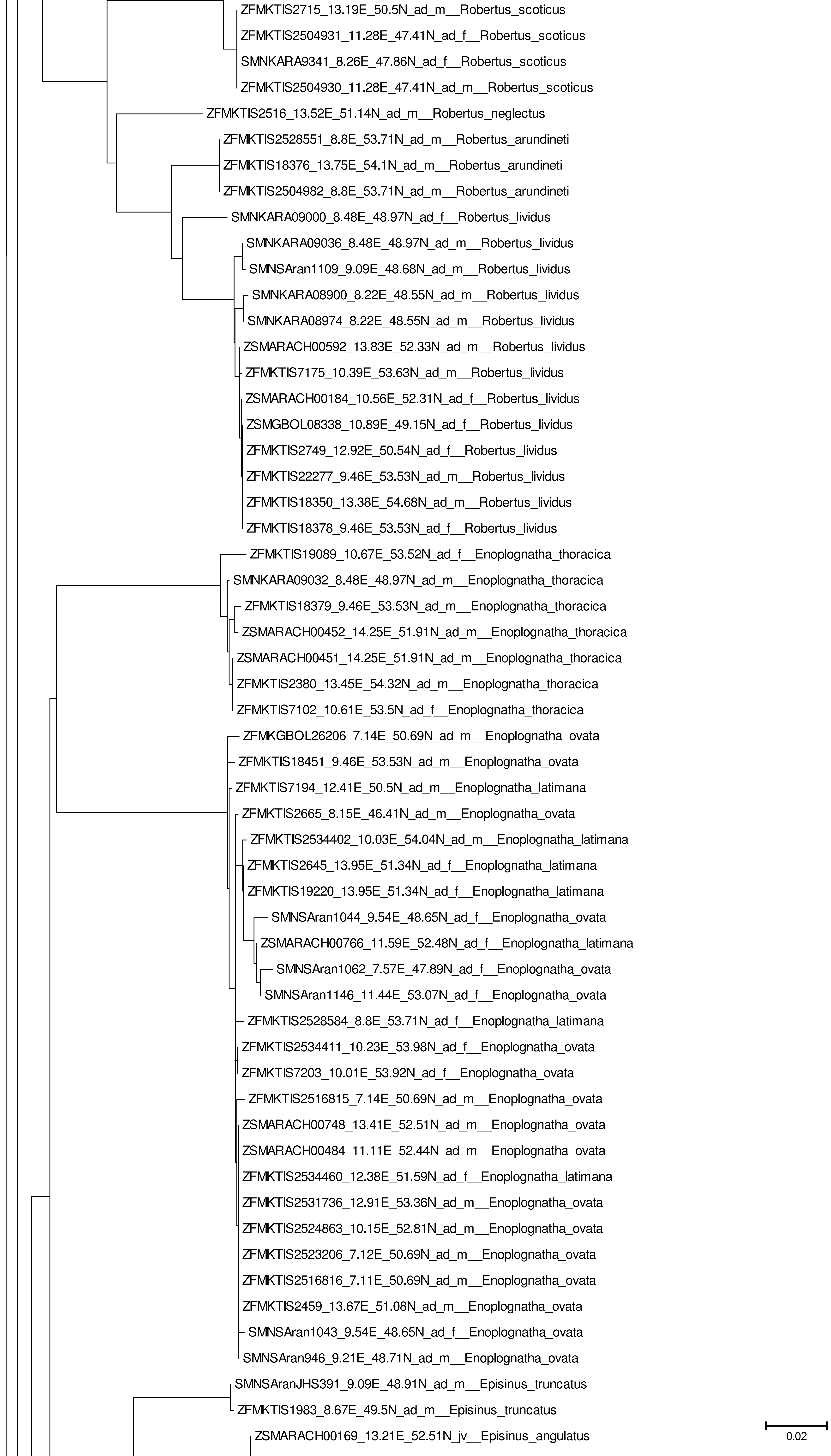
ZFMKTIS2715_13.19E_50.5N_ad_m__Robertus_scoticus
ZFMKTIS2504931_11.28E_47.41N_ad_f__Robertus_scoticus
SMNKARA9341_8.26E_47.86N_ad_f__Robertus_scoticus
ZFMKTIS2504930_11.28E_47.41N_ad_m__Robertus_scoticus
ZFMKTIS2516_13.52E_51.14N_ad_m__Robertus_neglectus
ZFMKTIS2528551_8.8E_53.71N_ad_m__Robertus_arundineti
ZFMKTIS18376_13.75E_54.1N_ad_m__Robertus_arundineti
ZFMKTIS2504982_8.8E_53.71N_ad_m__Robertus_arundineti
SMNKARA09000_8.48E_48.97N_ad_f__Robertus_lividus
SMNKARA09036_8.48E_48.97N_ad_m__Robertus_lividus
SMNSAran1109_9.09E_48.68N_ad_m__Robertus_lividus
SMNKARA08900_8.22E_48.55N_ad_m__Robertus_lividus
SMNKARA08974_8.22E_48.55N_ad_m__Robertus_lividus
ZSMARACH00592_13.83E_52.33N_ad_m__Robertus_lividus
ZFMKTIS7175_10.39E_53.63N_ad_m__Robertus_lividus
ZSMARACH00184_10.56E_52.31N_ad_f__Robertus_lividus
ZSMGBOL08338_10.89E_49.15N_ad_f__Robertus_lividus
ZFMKTIS2749_12.92E_50.54N_ad_f__Robertus_lividus
ZFMKTIS22277_9.46E_53.53N_ad_m__Robertus_lividus
ZFMKTIS18350_13.38E_54.68N_ad_m__Robertus_lividus
ZFMKTIS18378_9.46E_53.53N_ad_f__Robertus_lividus
ZFMKTIS19089_10.67E_53.52N_ad_f__Enoplognatha_thoracica
SMNKARA09032_8.48E_48.97N_ad_m__Enoplognatha_thoracica
ZFMKTIS18379_9.46E_53.53N_ad_m__Enoplognatha_thoracica
ZSMARACH00452_14.25E_51.91N_ad_m__Enoplognatha_thoracica
ZSMARACH00451_14.25E_51.91N_ad_m__Enoplognatha_thoracica
ZFMKTIS2380_13.45E_54.32N_ad_m__Enoplognatha_thoracica
ZFMKTIS7102_10.61E_53.5N_ad_f__Enoplognatha_thoracica
ZFMKGBOL26206_7.14E_50.69N_ad_m__Enoplognatha_ovata
ZFMKTIS18451_9.46E_53.53N_ad_m__Enoplognatha_ovata
ZFMKTIS7194_12.41E_50.5N_ad_m__Enoplognatha_latimana
ZFMKTIS2665_8.15E_46.41N_ad_m__Enoplognatha_ovata
ZFMKTIS2534402_10.03E_54.04N_ad_m__Enoplognatha_latimana
ZFMKTIS2645_13.95E_51.34N_ad_f__Enoplognatha_latimana
ZFMKTIS19220_13.95E_51.34N_ad_f__Enoplognatha_latimana
SMNSAran1044_9.54E_48.65N_ad_f__Enoplognatha_ovata
ZSMARACH00766_11.59E_52.48N_ad_f__Enoplognatha_latimana
SMNSAran1062_7.57E_47.89N_ad_f__Enoplognatha_ovata
SMNSAran1146_11.44E_53.07N_ad_f__Enoplognatha_ovata
ZFMKTIS2528584_8.8E_53.71N_ad_f__Enoplognatha_latimana
ZFMKTIS2534411_10.23E_53.98N_ad_f__Enoplognatha_ovata
ZFMKTIS7203_10.01E_53.92N_ad_f__Enoplognatha_ovata
ZFMKTIS2516815_7.14E_50.69N_ad_m__Enoplognatha_ovata
ZSMARACH00748_13.41E_52.51N_ad_m__Enoplognatha_ovata
ZSMARACH00484_11.11E_52.44N_ad_m__Enoplognatha_ovata
ZFMKTIS2534460_12.38E_51.59N_ad_f__Enoplognatha_latimana
ZFMKTIS2531736_12.91E_53.36N_ad_m__Enoplognatha_ovata
ZFMKTIS2524863_10.15E_52.81N_ad_m__Enoplognatha_ovata
ZFMKTIS2523206_7.12E_50.69N_ad_m__Enoplognatha_ovata
ZFMKTIS2516816_7.11E_50.69N_ad_m__Enoplognatha_ovata
ZFMKTIS2459_13.67E_51.08N_ad_m__Enoplognatha_ovata
SMNSAran1043_9.54E_48.65N_ad_f__Enoplognatha_ovata
SMNSAran946_9.21E_48.71N_ad_m__Enoplognatha_ovata
SMNSAranJHS391_9.09E_48.91N_ad_m__Episinus_truncatus
ZFMKTIS1983_8.67E_49.5N_ad_m__Episinus_truncatus
ZSMARACH00169_13.21E_52.51N_jv__Episinus_angulatus
0.02

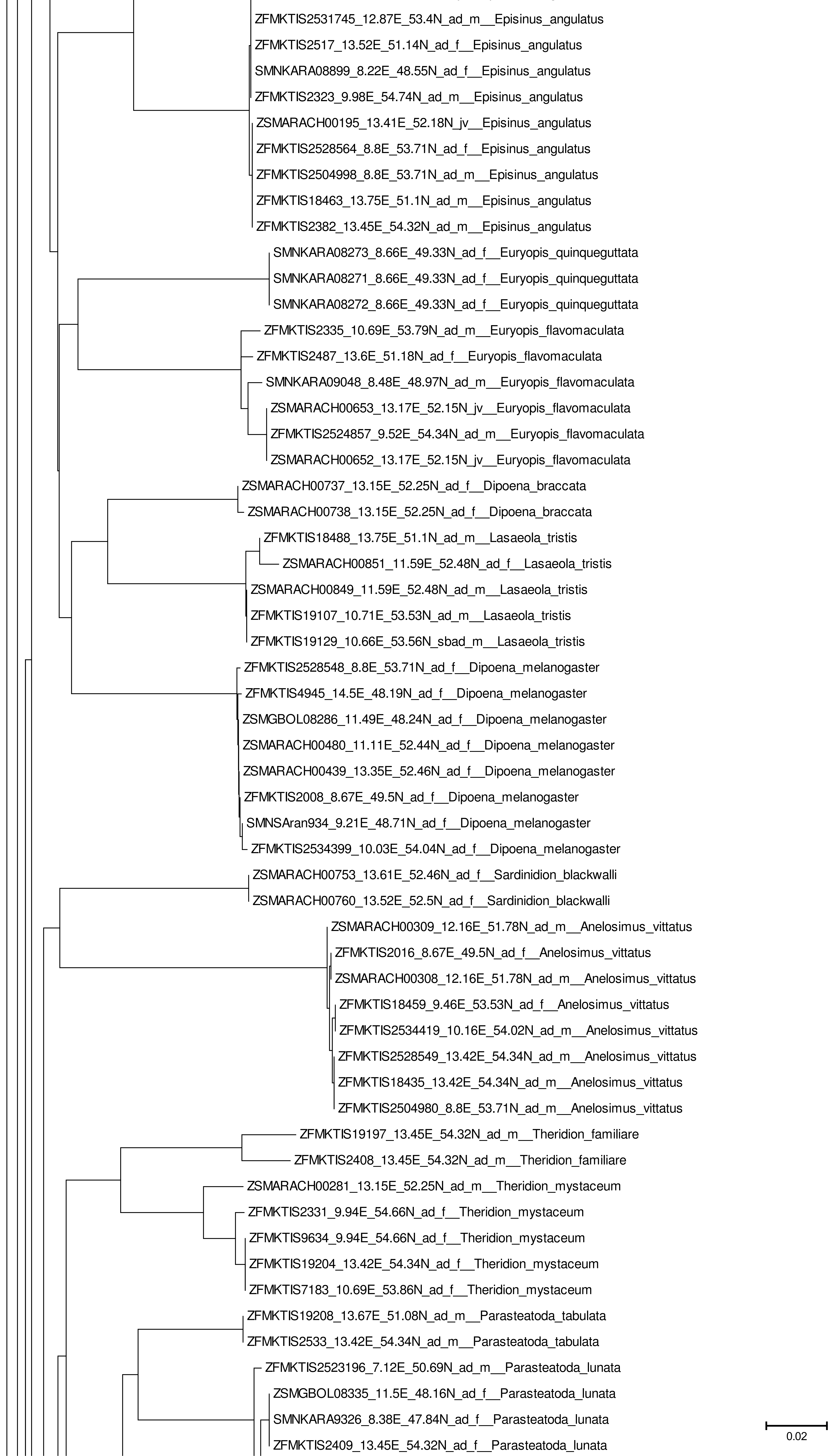

ZFMKTIS2531745_12.87E_53.4N_ad_m__Episinus_angulatus
ZFMKTIS2517_13.52E_51.14N_ad_f__Episinus_angulatus
SMNKARA08899_8.22E_48.55N_ad_f__Episinus_angulatus
ZFMKTIS2323_9.98E_54.74N_ad_m__Episinus_angulatus
ZSMARACH00195_13.41E_52.18N_jv__Episinus_angulatus
ZFMKTIS2528564_8.8E_53.71N_ad_f__Episinus_angulatus
ZFMKTIS2504998_8.8E_53.71N_ad_m__Episinus_angulatus
ZFMKTIS18463_13.75E_51.1N_ad_m__Episinus_angulatus
ZFMKTIS2382_13.45E_54.32N_ad_m__Episinus_angulatus
SMNKARA08273_8.66E_49.33N_ad_f__Euryopis_quinqueguttata
SMNKARA08271_8.66E_49.33N_ad_f__Euryopis_quinqueguttata
SMNKARA08272_8.66E_49.33N_ad_f__Euryopis_quinqueguttata
ZFMKTIS2335_10.69E_53.79N_ad_m__Euryopis_flavomaculata
ZFMKTIS2487_13.6E_51.18N_ad_f__Euryopis_flavomaculata
SMNKARA09048_8.48E_48.97N_ad_m__Euryopis_flavomaculata
ZSMARACH00653_13.17E_52.15N_jv__Euryopis_flavomaculata
ZFMKTIS2524857_9.52E_54.34N_ad_m__Euryopis_flavomaculata
ZSMARACH00652_13.17E_52.15N_jv__Euryopis_flavomaculata
ZSMARACH00737_13.15E_52.25N_ad_f__Dipoena_braccata
ZSMARACH00738_13.15E_52.25N_ad_f__Dipoena_braccata
ZFMKTIS18488_13.75E_51.1N_ad_m__Lasaeola_tristis
ZSMARACH00851_11.59E_52.48N_ad_f__Lasaeola_tristis
ZSMARACH00849_11.59E_52.48N_ad_m__Lasaeola_tristis
ZFMKTIS19107_10.71E_53.53N_ad_m__Lasaeola_tristis
ZFMKTIS19129_10.66E_53.56N_sbad_m__Lasaeola_tristis
ZFMKTIS2528548_8.8E_53.71N_ad_f__Dipoena_melanogaster
ZFMKTIS4945_14.5E_48.19N_ad_f__Dipoena_melanogaster
ZSMGBOL08286_11.49E_48.24N_ad_f__Dipoena_melanogaster
ZSMARACH00480_11.11E_52.44N_ad_f__Dipoena_melanogaster
ZSMARACH00439_13.35E_52.46N_ad_f__Dipoena_melanogaster
ZFMKTIS2008_8.67E_49.5N_ad_f__Dipoena_melanogaster
SMNSAran934_9.21E_48.71N_ad_f__Dipoena_melanogaster
ZFMKTIS2534399_10.03E_54.04N_ad_f__Dipoena_melanogaster
ZSMARACH00753_13.61E_52.46N_ad_f__Sardinidion_blackwalli
ZSMARACH00760_13.52E_52.5N_ad_f__Sardinidion_blackwalli
ZSMARACH00309_12.16E_51.78N_ad_m__Anelosimus_vittatus
ZFMKTIS2016_8.67E_49.5N_ad_f__Anelosimus_vittatus
ZSMARACH00308_12.16E_51.78N_ad_m__Anelosimus_vittatus
ZFMKTIS18459_9.46E_53.53N_ad_f__Anelosimus_vittatus
ZFMKTIS2534419_10.16E_54.02N_ad_m__Anelosimus_vittatus
ZFMKTIS2528549_13.42E_54.34N_ad_m__Anelosimus_vittatus
ZFMKTIS18435_13.42E_54.34N_ad_m__Anelosimus_vittatus
ZFMKTIS2504980_8.8E_53.71N_ad_m__Anelosimus_vittatus
ZFMKTIS19197_13.45E_54.32N_ad_m__Theridion_familiare
ZFMKTIS2408_13.45E_54.32N_ad_m__Theridion_familiare
ZSMARACH00281_13.15E_52.25N_ad_m__Theridion_mystaceum
ZFMKTIS2331_9.94E_54.66N_ad_f__Theridion_mystaceum
ZFMKTIS9634_9.94E_54.66N_ad_f__Theridion_mystaceum
ZFMKTIS19204_13.42E_54.34N_ad_f__Theridion_mystaceum
ZFMKTIS7183_10.69E_53.86N_ad_f__Theridion_mystaceum
ZFMKTIS19208_13.67E_51.08N_ad_m__Parasteatoda_tabulata
ZFMKTIS2533_13.42E_54.34N_ad_m__Parasteatoda_tabulata
ZFMKTIS2523196_7.12E_50.69N_ad_m__Parasteatoda_lunata
ZSMGBOL08335_11.5E_48.16N_ad_f__Parasteatoda_lunata
SMNKARA9326_8.38E_47.84N_ad_f__Parasteatoda_lunata
ZFMKTIS2409_13.45E_54.32N_ad_f__Parasteatoda_lunata
0.02

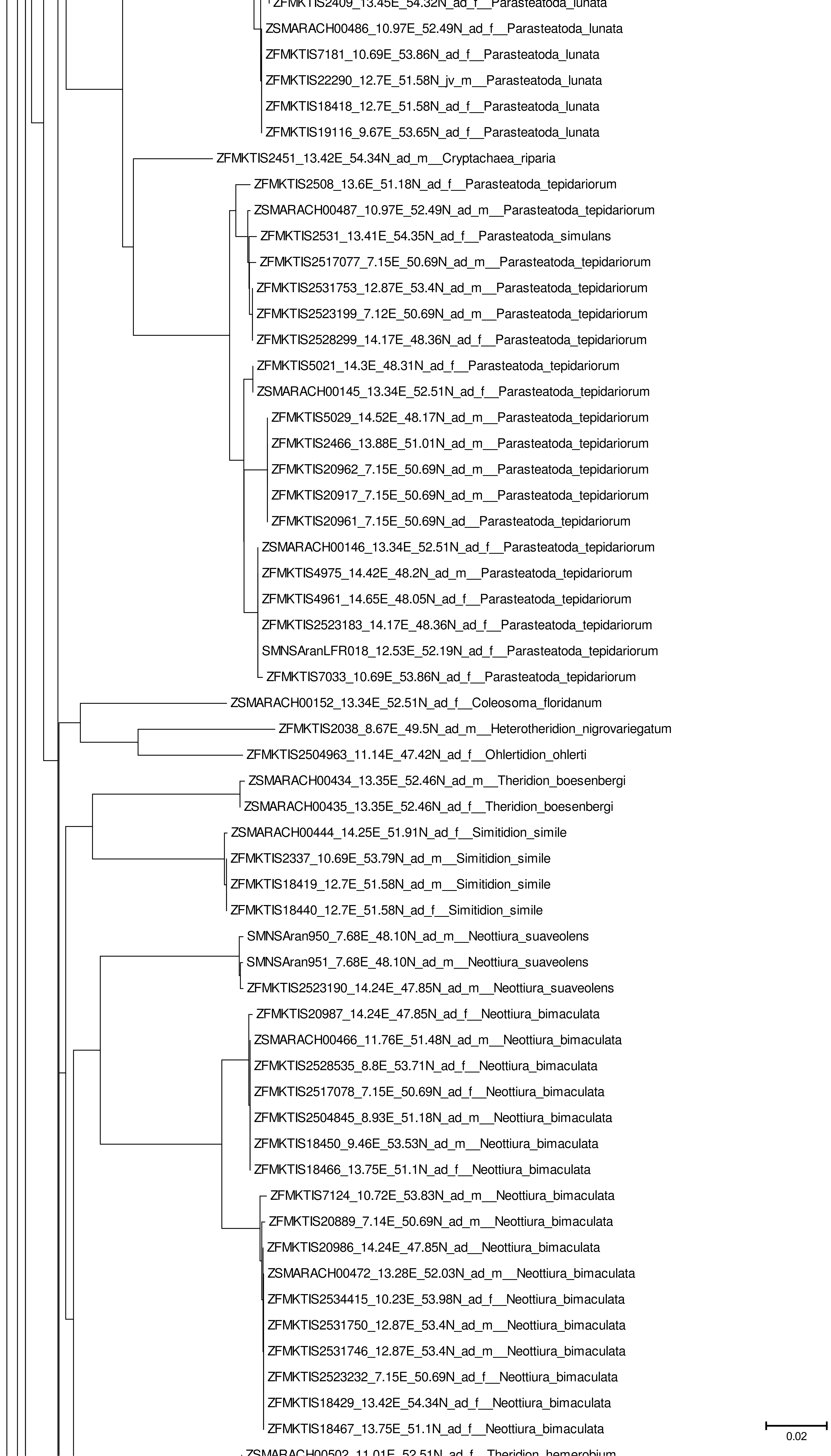
ZFMKTIS2409_13.45E_54.32N_ad_f__Parasteatoda_lunata
ZSMARACH00486_10.97E_52.49N_ad_f__Parasteatoda_lunata
ZFMKTIS7181_10.69E_53.86N_ad_f__Parasteatoda_lunata
ZFMKTIS22290_12.7E_51.58N_jv_m__Parasteatoda_lunata
ZFMKTIS18418_12.7E_51.58N_ad_f__Parasteatoda_lunata
ZFMKTIS19116_9.67E_53.65N_ad_f__Parasteatoda_lunata
ZFMKTIS2451_13.42E_54.34N_ad_m__Cryptachaea_riparia
ZFMKTIS2508_13.6E_51.18N_ad_f__Parasteatoda_tepidariorum
ZSMARACH00487_10.97E_52.49N_ad_m__Parasteatoda_tepidariorum
ZFMKTIS2531_13.41E_54.35N_ad_f__Parasteatoda_simulans
ZFMKTIS2517077_7.15E_50.69N_ad_m__Parasteatoda_tepidariorum
ZFMKTIS2531753_12.87E_53.4N_ad_m__Parasteatoda_tepidariorum
ZFMKTIS2523199_7.12E_50.69N_ad_m__Parasteatoda_tepidariorum
ZFMKTIS2528299_14.17E_48.36N_ad_f__Parasteatoda_tepidariorum
ZFMKTIS5021_14.3E_48.31N_ad_f__Parasteatoda_tepidariorum
ZSMARACH00145_13.34E_52.51N_ad_f__Parasteatoda_tepidariorum
ZFMKTIS5029_14.52E_48.17N_ad_m__Parasteatoda_tepidariorum
ZFMKTIS2466_13.88E_51.01N_ad_m__Parasteatoda_tepidariorum
ZFMKTIS20962_7.15E_50.69N_ad_m__Parasteatoda_tepidariorum
ZFMKTIS20917_7.15E_50.69N_ad_m__Parasteatoda_tepidariorum
ZFMKTIS20961_7.15E_50.69N_ad__Parasteatoda_tepidariorum
ZSMARACH00146_13.34E_52.51N_ad_f__Parasteatoda_tepidariorum
ZFMKTIS4975_14.42E_48.2N_ad_m__Parasteatoda_tepidariorum
ZFMKTIS4961_14.65E_48.05N_ad_f__Parasteatoda_tepidariorum
ZFMKTIS2523183_14.17E_48.36N_ad_f__Parasteatoda_tepidariorum
SMNSAranLFR018_12.53E_52.19N_ad_f__Parasteatoda_tepidariorum
ZFMKTIS7033_10.69E_53.86N_ad_f__Parasteatoda_tepidariorum
ZSMARACH00152_13.34E_52.51N_ad_f__Coleosoma_floridanum
ZFMKTIS2038_8.67E_49.5N_ad_m__Heterotheridion_nigrovariegatum
ZFMKTIS2504963_11.14E_47.42N_ad_f__Ohlertidion_ohlerti
ZSMARACH00434_13.35E_52.46N_ad_m__Theridion_boesenbergi
ZSMARACH00435_13.35E_52.46N_ad_f__Theridion_boesenbergi
ZSMARACH00444_14.25E_51.91N_ad_f__Simitidion_simile
ZFMKTIS2337_10.69E_53.79N_ad_m__Simitidion_simile
ZFMKTIS18419_12.7E_51.58N_ad_m__Simitidion_simile
ZFMKTIS18440_12.7E_51.58N_ad_f__Simitidion_simile
SMNSAran950_7.68E_48.10N_ad_m__Neottiura_suaveolens
SMNSAran951_7.68E_48.10N_ad_m__Neottiura_suaveolens
ZFMKTIS2523190_14.24E_47.85N_ad_m__Neottiura_suaveolens
ZFMKTIS20987_14.24E_47.85N_ad_f__Neottiura_bimaculata
ZSMARACH00466_11.76E_51.48N_ad_m__Neottiura_bimaculata
ZFMKTIS2528535_8.8E_53.71N_ad_f__Neottiura_bimaculata
ZFMKTIS2517078_7.15E_50.69N_ad_f__Neottiura_bimaculata
ZFMKTIS2504845_8.93E_51.18N_ad_m__Neottiura_bimaculata
ZFMKTIS18450_9.46E_53.53N_ad_m__Neottiura_bimaculata
ZFMKTIS18466_13.75E_51.1N_ad_f__Neottiura_bimaculata
ZFMKTIS7124_10.72E_53.83N_ad_m__Neottiura_bimaculata
ZFMKTIS20889_7.14E_50.69N_ad_m__Neottiura_bimaculata
ZFMKTIS20986_14.24E_47.85N_ad__Neottiura_bimaculata
ZSMARACH00472_13.28E_52.03N_ad_m__Neottiura_bimaculata
ZFMKTIS2534415_10.23E_53.98N_ad_f__Neottiura_bimaculata
ZFMKTIS2531750_12.87E_53.4N_ad_m__Neottiura_bimaculata
ZFMKTIS2531746_12.87E_53.4N_ad_m__Neottiura_bimaculata
ZFMKTIS2523232_7.15E_50.69N_ad_f__Neottiura_bimaculata
ZFMKTIS18429_13.42E_54.34N_ad_f__Neottiura_bimaculata
ZFMKTIS18467_13.75E_51.1N_ad_f__Neottiura_bimaculata
0.02
ZSMARACH00502_11.01E_52.51N_ad_f__Theridion_hemerobium

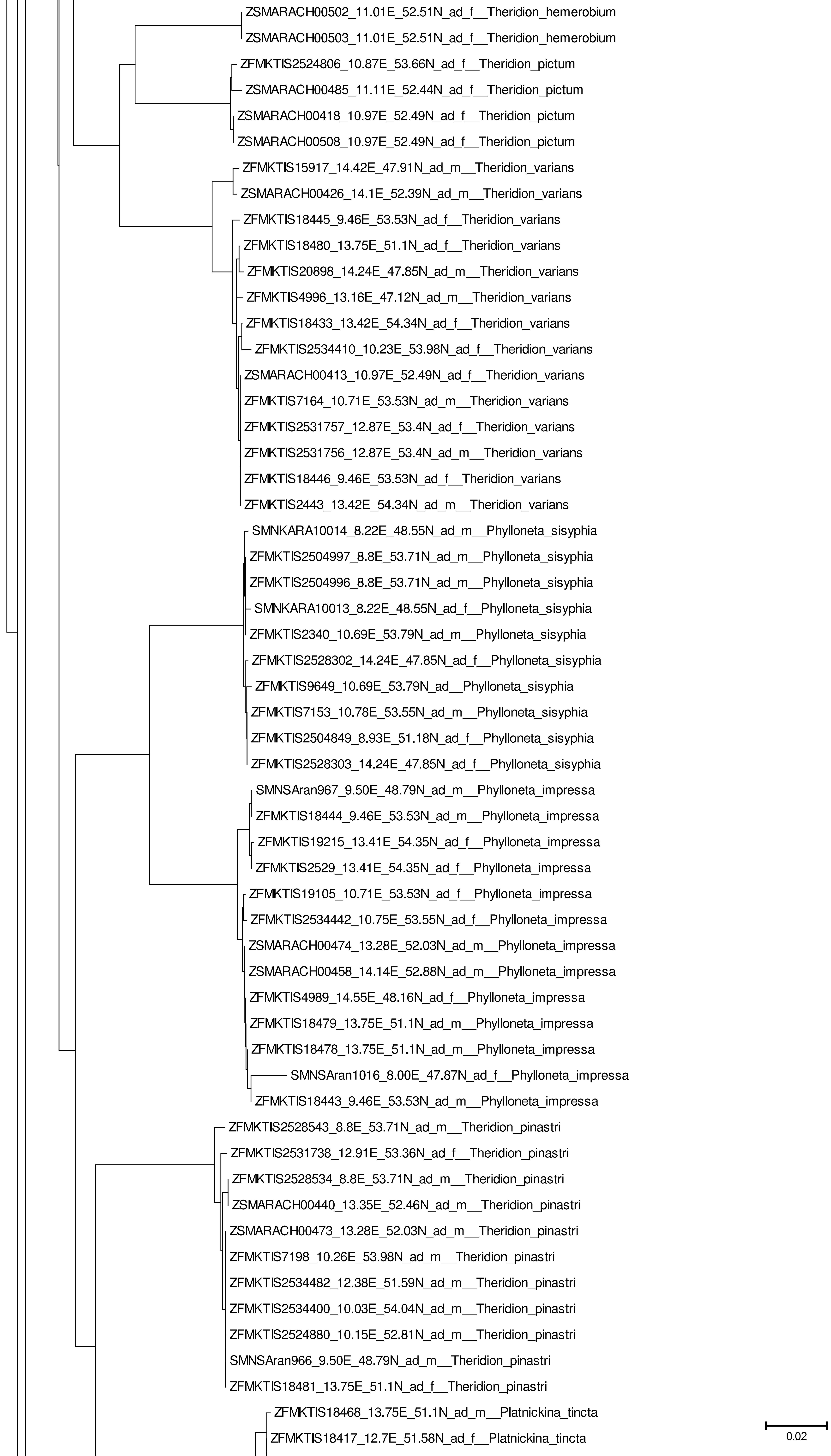
ZSMARACH00502_11.01E_52.51N_ad_f__Theridion_hemerobium
ZSMARACH00503_11.01E_52.51N_ad_f__Theridion_hemerobium
ZFMKTIS2524806_10.87E_53.66N_ad_f__Theridion_pictum
ZSMARACH00485_11.11E_52.44N_ad_f__Theridion_pictum
ZSMARACH00418_10.97E_52.49N_ad_f__Theridion_pictum
ZSMARACH00508_10.97E_52.49N_ad_f__Theridion_pictum
ZFMKTIS15917_14.42E_47.91N_ad_m__Theridion_varians
ZSMARACH00426_14.1E_52.39N_ad_m__Theridion_varians
ZFMKTIS18445_9.46E_53.53N_ad_f__Theridion_varians
ZFMKTIS18480_13.75E_51.1N_ad_f__Theridion_varians
ZFMKTIS20898_14.24E_47.85N_ad_m__Theridion_varians
ZFMKTIS4996_13.16E_47.12N_ad_m__Theridion_varians
ZFMKTIS18433_13.42E_54.34N_ad_f__Theridion_varians
ZFMKTIS2534410_10.23E_53.98N_ad_f__Theridion_varians
ZSMARACH00413_10.97E_52.49N_ad_f__Theridion_varians
ZFMKTIS7164_10.71E_53.53N_ad_m__Theridion_varians
ZFMKTIS2531757_12.87E_53.4N_ad_f__Theridion_varians
ZFMKTIS2531756_12.87E_53.4N_ad_m__Theridion_varians
ZFMKTIS18446_9.46E_53.53N_ad_f__Theridion_varians
ZFMKTIS2443_13.42E_54.34N_ad_m__Theridion_varians
SMNKARA10014_8.22E_48.55N_ad_m__Phylloneta_sisyphia
ZFMKTIS2504997_8.8E_53.71N_ad_m__Phylloneta_sisyphia
ZFMKTIS2504996_8.8E_53.71N_ad_m__Phylloneta_sisyphia
SMNKARA10013_8.22E_48.55N_ad_f__Phylloneta_sisyphia
ZFMKTIS2340_10.69E_53.79N_ad_m__Phylloneta_sisyphia
ZFMKTIS2528302_14.24E_47.85N_ad_f__Phylloneta_sisyphia
ZFMKTIS9649_10.69E_53.79N_ad__Phylloneta_sisyphia
ZFMKTIS7153_10.78E_53.55N_ad_m__Phylloneta_sisyphia
ZFMKTIS2504849_8.93E_51.18N_ad_f__Phylloneta_sisyphia
ZFMKTIS2528303_14.24E_47.85N_ad_f__Phylloneta_sisyphia
SMNSAran967_9.50E_48.79N_ad_m__Phylloneta_impressa
ZFMKTIS18444_9.46E_53.53N_ad_m__Phylloneta_impressa
ZFMKTIS19215_13.41E_54.35N_ad_f__Phylloneta_impressa
ZFMKTIS2529_13.41E_54.35N_ad_f__Phylloneta_impressa
ZFMKTIS19105_10.71E_53.53N_ad_f__Phylloneta_impressa
ZFMKTIS2534442_10.75E_53.55N_ad_f__Phylloneta_impressa
ZSMARACH00474_13.28E_52.03N_ad_m__Phylloneta_impressa
ZSMARACH00458_14.14E_52.88N_ad_m__Phylloneta_impressa
ZFMKTIS4989_14.55E_48.16N_ad_f__Phylloneta_impressa
ZFMKTIS18479_13.75E_51.1N_ad_m__Phylloneta_impressa
ZFMKTIS18478_13.75E_51.1N_ad_m__Phylloneta_impressa
SMNSAran1016_8.00E_47.87N_ad_f__Phylloneta_impressa
ZFMKTIS18443_9.46E_53.53N_ad_m__Phylloneta_impressa
ZFMKTIS2528543_8.8E_53.71N_ad_m__Theridion_pinastri
ZFMKTIS2531738_12.91E_53.36N_ad_f__Theridion_pinastri
ZFMKTIS2528534_8.8E_53.71N_ad_m__Theridion_pinastri
ZSMARACH00440_13.35E_52.46N_ad_m__Theridion_pinastri
ZSMARACH00473_13.28E_52.03N_ad_m__Theridion_pinastri
ZFMKTIS7198_10.26E_53.98N_ad_m__Theridion_pinastri
ZFMKTIS2534482_12.38E_51.59N_ad_m__Theridion_pinastri
ZFMKTIS2534400_10.03E_54.04N_ad_m__Theridion_pinastri
ZFMKTIS2524880_10.15E_52.81N_ad_m__Theridion_pinastri
SMNSAran966_9.50E_48.79N_ad_m__Theridion_pinastri
ZFMKTIS18481_13.75E_51.1N_ad_f__Theridion_pinastri
ZFMKTIS18468_13.75E_51.1N_ad_m__Platnickina_tincta
ZFMKTIS18417_12.7E_51.58N_ad_f__Platnickina_tincta
0.02

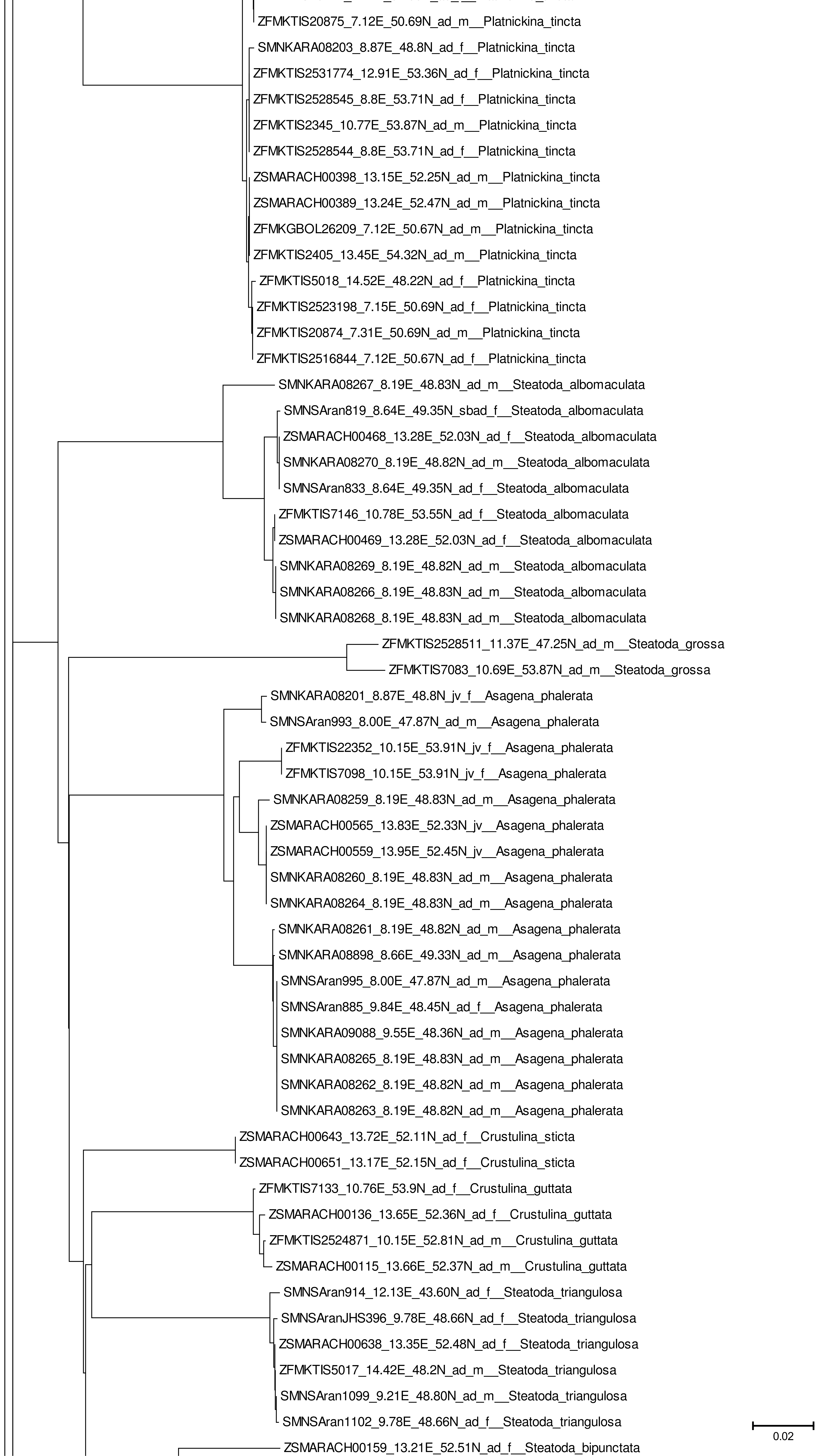
ZFMKTIS20875_7.12E_50.69N_ad_m__Platnickina_tincta
SMNKARA08203_8.87E_48.8N_ad_f__Platnickina_tincta
ZFMKTIS2531774_12.91E_53.36N_ad_f__Platnickina_tincta
ZFMKTIS2528545_8.8E_53.71N_ad_f__Platnickina_tincta
ZFMKTIS2345_10.77E_53.87N_ad_m__Platnickina_tincta
ZFMKTIS2528544_8.8E_53.71N_ad_f__Platnickina_tincta
ZSMARACH00398_13.15E_52.25N_ad_m__Platnickina_tincta
ZSMARACH00389_13.24E_52.47N_ad_m__Platnickina_tincta
ZFMKGBOL26209_7.12E_50.67N_ad_m__Platnickina_tincta
ZFMKTIS2405_13.45E_54.32N_ad_m__Platnickina_tincta
ZFMKTIS5018_14.52E_48.22N_ad_f__Platnickina_tincta
ZFMKTIS2523198_7.15E_50.69N_ad_f__Platnickina_tincta
ZFMKTIS20874_7.31E_50.69N_ad_m__Platnickina_tincta
ZFMKTIS2516844_7.12E_50.67N_ad_f__Platnickina_tincta
SMNKARA08267_8.19E_48.83N_ad_m__Steatoda_albomaculata
SMNSAran819_8.64E_49.35N_sbad_f__Steatoda_albomaculata
ZSMARACH00468_13.28E_52.03N_ad_f__Steatoda_albomaculata
SMNKARA08270_8.19E_48.82N_ad_m__Steatoda_albomaculata
SMNSAran833_8.64E_49.35N_ad_f__Steatoda_albomaculata
ZFMKTIS7146_10.78E_53.55N_ad_f__Steatoda_albomaculata
ZSMARACH00469_13.28E_52.03N_ad_f__Steatoda_albomaculata
SMNKARA08269_8.19E_48.82N_ad_m__Steatoda_albomaculata
SMNKARA08266_8.19E_48.83N_ad_m__Steatoda_albomaculata
SMNKARA08268_8.19E_48.83N_ad_m__Steatoda_albomaculata
ZFMKTIS2528511_11.37E_47.25N_ad_m__Steatoda_grossa
ZFMKTIS7083_10.69E_53.87N_ad_m__Steatoda_grossa
SMNKARA08201_8.87E_48.8N_jv_f__Asagena_phalerata
SMNSAran993_8.00E_47.87N_ad_m__Asagena_phalerata
ZFMKTIS22352_10.15E_53.91N_jv_f__Asagena_phalerata
ZFMKTIS7098_10.15E_53.91N_jv_f__Asagena_phalerata
SMNKARA08259_8.19E_48.83N_ad_m__Asagena_phalerata
ZSMARACH00565_13.83E_52.33N_jv__Asagena_phalerata
ZSMARACH00559_13.95E_52.45N_jv__Asagena_phalerata
SMNKARA08260_8.19E_48.83N_ad_m__Asagena_phalerata
SMNKARA08264_8.19E_48.83N_ad_m__Asagena_phalerata
SMNKARA08261_8.19E_48.82N_ad_m__Asagena_phalerata
SMNKARA08898_8.66E_49.33N_ad_m__Asagena_phalerata
SMNSAran995_8.00E_47.87N_ad_m__Asagena_phalerata
SMNSAran885_9.84E_48.45N_ad_f__Asagena_phalerata
SMNKARA09088_9.55E_48.36N_ad_m__Asagena_phalerata
SMNKARA08265_8.19E_48.83N_ad_m__Asagena_phalerata
SMNKARA08262_8.19E_48.82N_ad_m__Asagena_phalerata
SMNKARA08263_8.19E_48.82N_ad_m__Asagena_phalerata
ZSMARACH00643_13.72E_52.11N_ad_f__Crustulina_sticta
ZSMARACH00651_13.17E_52.15N_ad_f__Crustulina_sticta
ZFMKTIS7133_10.76E_53.9N_ad_f__Crustulina_guttata
ZSMARACH00136_13.65E_52.36N_ad_f__Crustulina_guttata
ZFMKTIS2524871_10.15E_52.81N_ad_m__Crustulina_guttata
ZSMARACH00115_13.66E_52.37N_ad_m__Crustulina_guttata
SMNSAran914_12.13E_43.60N_ad_f__Steatoda_triangulosa
SMNSAranJHS396_9.78E_48.66N_ad_f__Steatoda_triangulosa
ZSMARACH00638_13.35E_52.48N_ad_f__Steatoda_triangulosa
ZFMKTIS5017_14.42E_48.2N_ad_m__Steatoda_triangulosa
SMNSAran1099_9.21E_48.80N_ad_m__Steatoda_triangulosa
SMNSAran1102_9.78E_48.66N_ad_f__Steatoda_triangulosa
0.02
ZSMARACH00159_13.21E_52.51N_ad_f__Steatoda_bipunctata

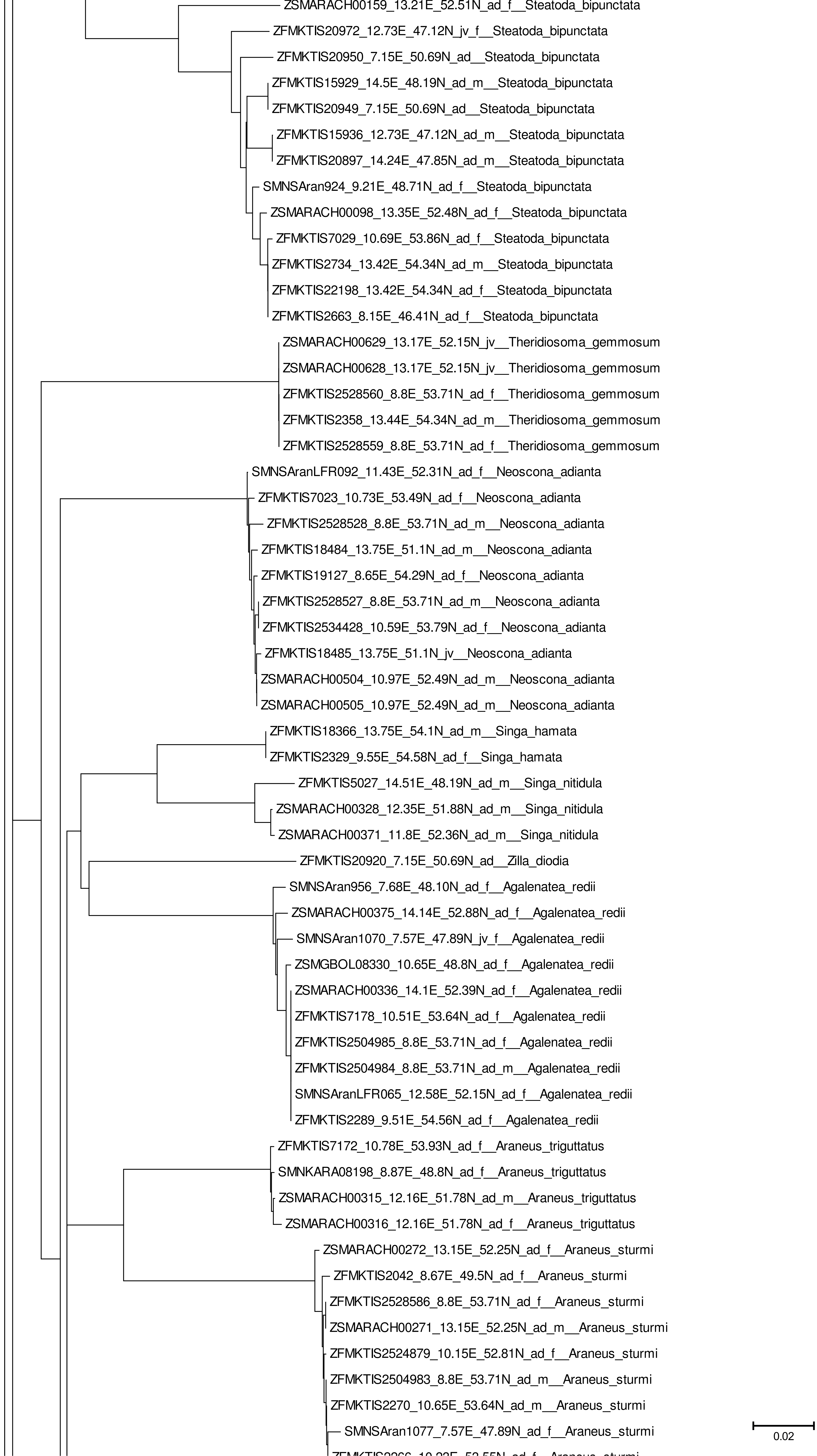

ZSMARACH00159_13.21E_52.51N_ad_f__Steatoda_bipunctata
ZFMKTIS20972_12.73E_47.12N_jv_f__Steatoda_bipunctata
ZFMKTIS20950_7.15E_50.69N_ad__Steatoda_bipunctata
ZFMKTIS15929_14.5E_48.19N_ad_m__Steatoda_bipunctata
ZFMKTIS20949_7.15E_50.69N_ad__Steatoda_bipunctata
ZFMKTIS15936_12.73E_47.12N_ad_m__Steatoda_bipunctata
ZFMKTIS20897_14.24E_47.85N_ad_m__Steatoda_bipunctata
SMNSAran924_9.21E_48.71N_ad_f__Steatoda_bipunctata
ZSMARACH00098_13.35E_52.48N_ad_f__Steatoda_bipunctata
ZFMKTIS7029_10.69E_53.86N_ad_f__Steatoda_bipunctata
ZFMKTIS2734_13.42E_54.34N_ad_m__Steatoda_bipunctata
ZFMKTIS22198_13.42E_54.34N_ad_f__Steatoda_bipunctata
ZFMKTIS2663_8.15E_46.41N_ad_f__Steatoda_bipunctata
ZSMARACH00629_13.17E_52.15N_jv__Theridiosoma_gemmosum
ZSMARACH00628_13.17E_52.15N_jv__Theridiosoma_gemmosum
ZFMKTIS2528560_8.8E_53.71N_ad_f__Theridiosoma_gemmosum
ZFMKTIS2358_13.44E_54.34N_ad_m__Theridiosoma_gemmosum
ZFMKTIS2528559_8.8E_53.71N_ad_f__Theridiosoma_gemmosum
SMNSAranLFR092_11.43E_52.31N_ad_f__Neoscona_adianta
ZFMKTIS7023_10.73E_53.49N_ad_f__Neoscona_adianta
ZFMKTIS2528528_8.8E_53.71N_ad_m__Neoscona_adianta
ZFMKTIS18484_13.75E_51.1N_ad_m__Neoscona_adianta
ZFMKTIS19127_8.65E_54.29N_ad_f__Neoscona_adianta
ZFMKTIS2528527_8.8E_53.71N_ad_m__Neoscona_adianta
ZFMKTIS2534428_10.59E_53.79N_ad_f__Neoscona_adianta
ZFMKTIS18485_13.75E_51.1N_jv__Neoscona_adianta
ZSMARACH00504_10.97E_52.49N_ad_m__Neoscona_adianta
ZSMARACH00505_10.97E_52.49N_ad_m__Neoscona_adianta
ZFMKTIS18366_13.75E_54.1N_ad_m__Singa_hamata
ZFMKTIS2329_9.55E_54.58N_ad_f__Singa_hamata
ZFMKTIS5027_14.51E_48.19N_ad_m__Singa_nitidula
ZSMARACH00328_12.35E_51.88N_ad_m__Singa_nitidula
ZSMARACH00371_11.8E_52.36N_ad_m__Singa_nitidula
ZFMKTIS20920_7.15E_50.69N_ad__Zilla_diodia
SMNSAran956_7.68E_48.10N_ad_f__Agalenatea_redii
ZSMARACH00375_14.14E_52.88N_ad_f__Agalenatea_redii
SMNSAran1070_7.57E_47.89N_jv_f__Agalenatea_redii
ZSMGBOL08330_10.65E_48.8N_ad_f__Agalenatea_redii
ZSMARACH00336_14.1E_52.39N_ad_f__Agalenatea_redii
ZFMKTIS7178_10.51E_53.64N_ad_f__Agalenatea_redii
ZFMKTIS2504985_8.8E_53.71N_ad_f__Agalenatea_redii
ZFMKTIS2504984_8.8E_53.71N_ad_m__Agalenatea_redii
SMNSAranLFR065_12.58E_52.15N_ad_f__Agalenatea_redii
ZFMKTIS2289_9.51E_54.56N_ad_f__Agalenatea_redii
ZFMKTIS7172_10.78E_53.93N_ad_f__Araneus_triguttatus
SMNKARA08198_8.87E_48.8N_ad_f__Araneus_triguttatus
ZSMARACH00315_12.16E_51.78N_ad_m__Araneus_triguttatus
ZSMARACH00316_12.16E_51.78N_ad_f__Araneus_triguttatus
ZSMARACH00272_13.15E_52.25N_ad_f__Araneus_sturmi
ZFMKTIS2042_8.67E_49.5N_ad_f__Araneus_sturmi
ZFMKTIS2528586_8.8E_53.71N_ad_f__Araneus_sturmi
ZSMARACH00271_13.15E_52.25N_ad_m__Araneus_sturmi
ZFMKTIS2524879_10.15E_52.81N_ad_f__Araneus_sturmi
ZFMKTIS2504983_8.8E_53.71N_ad_m__Araneus_sturmi
ZFMKTIS2270_10.65E_53.64N_ad_m__Araneus_sturmi
SMNSAran1077_7.57E_47.89N_ad_f__Araneus_sturmi
0.02

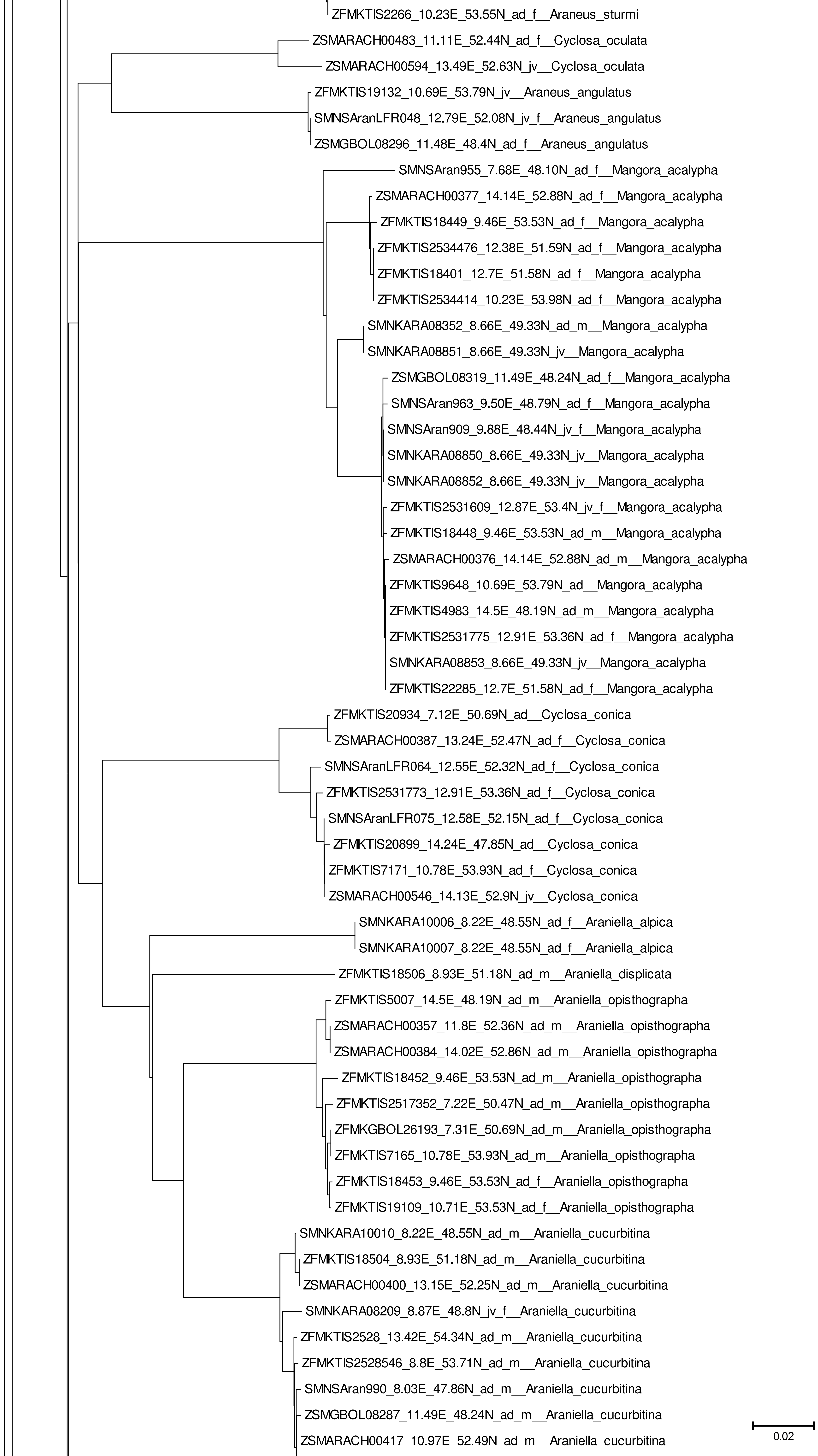
ZFMKTIS2266_10.23E_53.55N_ad_f__Araneus_sturmi
ZSMARACH00483_11.11E_52.44N_ad_f__Cyclosa_oculata
ZSMARACH00594_13.49E_52.63N_jv__Cyclosa_oculata
ZFMKTIS19132_10.69E_53.79N_jv__Araneus_angulatus
SMNSAranLFR048_12.79E_52.08N_jv_f__Araneus_angulatus
ZSMGBOL08296_11.48E_48.4N_ad_f__Araneus_angulatus
SMNSAran955_7.68E_48.10N_ad_f__Mangora_acalypha
ZSMARACH00377_14.14E_52.88N_ad_f__Mangora_acalypha
ZFMKTIS18449_9.46E_53.53N_ad_f__Mangora_acalypha
ZFMKTIS2534476_12.38E_51.59N_ad_f__Mangora_acalypha
ZFMKTIS18401_12.7E_51.58N_ad_f__Mangora_acalypha
ZFMKTIS2534414_10.23E_53.98N_ad_f__Mangora_acalypha
SMNKARA08352_8.66E_49.33N_ad_m__Mangora_acalypha
SMNKARA08851_8.66E_49.33N_jv__Mangora_acalypha
ZSMGBOL08319_11.49E_48.24N_ad_f__Mangora_acalypha
SMNSAran963_9.50E_48.79N_ad_f__Mangora_acalypha
SMNSAran909_9.88E_48.44N_jv_f__Mangora_acalypha
SMNKARA08850_8.66E_49.33N_jv__Mangora_acalypha
SMNKARA08852_8.66E_49.33N_jv__Mangora_acalypha
ZFMKTIS2531609_12.87E_53.4N_jv_f__Mangora_acalypha
ZFMKTIS18448_9.46E_53.53N_ad_m__Mangora_acalypha
ZSMARACH00376_14.14E_52.88N_ad_m__Mangora_acalypha
ZFMKTIS9648_10.69E_53.79N_ad__Mangora_acalypha
ZFMKTIS4983_14.5E_48.19N_ad_m__Mangora_acalypha
ZFMKTIS2531775_12.91E_53.36N_ad_f__Mangora_acalypha
SMNKARA08853_8.66E_49.33N_jv__Mangora_acalypha
ZFMKTIS22285_12.7E_51.58N_ad_f__Mangora_acalypha
ZFMKTIS20934_7.12E_50.69N_ad__Cyclosa_conica
ZSMARACH00387_13.24E_52.47N_ad_f__Cyclosa_conica
SMNSAranLFR064_12.55E_52.32N_ad_f__Cyclosa_conica
ZFMKTIS2531773_12.91E_53.36N_ad_f__Cyclosa_conica
SMNSAranLFR075_12.58E_52.15N_ad_f__Cyclosa_conica
ZFMKTIS20899_14.24E_47.85N_ad__Cyclosa_conica
ZFMKTIS7171_10.78E_53.93N_ad_f__Cyclosa_conica
ZSMARACH00546_14.13E_52.9N_jv__Cyclosa_conica
SMNKARA10006_8.22E_48.55N_ad_f__Araniella_alpica
SMNKARA10007_8.22E_48.55N_ad_f__Araniella_alpica
ZFMKTIS18506_8.93E_51.18N_ad_m__Araniella_displicata
ZFMKTIS5007_14.5E_48.19N_ad_m__Araniella_opisthographa
ZSMARACH00357_11.8E_52.36N_ad_m__Araniella_opisthographa
ZSMARACH00384_14.02E_52.86N_ad_m__Araniella_opisthographa
ZFMKTIS18452_9.46E_53.53N_ad_m__Araniella_opisthographa
ZFMKTIS2517352_7.22E_50.47N_ad_m__Araniella_opisthographa
ZFMKGBOL26193_7.31E_50.69N_ad_m__Araniella_opisthographa
ZFMKTIS7165_10.78E_53.93N_ad_m__Araniella_opisthographa
ZFMKTIS18453_9.46E_53.53N_ad_f__Araniella_opisthographa
ZFMKTIS19109_10.71E_53.53N_ad_f__Araniella_opisthographa
SMNKARA10010_8.22E_48.55N_ad_m__Araniella_cucurbitina
ZFMKTIS18504_8.93E_51.18N_ad_m__Araniella_cucurbitina
ZSMARACH00400_13.15E_52.25N_ad_m__Araniella_cucurbitina
SMNKARA08209_8.87E_48.8N_jv_f__Araniella_cucurbitina
ZFMKTIS2528_13.42E_54.34N_ad_m__Araniella_cucurbitina
ZFMKTIS2528546_8.8E_53.71N_ad_m__Araniella_cucurbitina
SMNSAran990_8.03E_47.86N_ad_m__Araniella_cucurbitina
ZSMGBOL08287_11.49E_48.24N_ad_m__Araniella_cucurbitina
ZSMARACH00417_10.97E_52.49N_ad_m__Araniella_cucurbitina
0.02

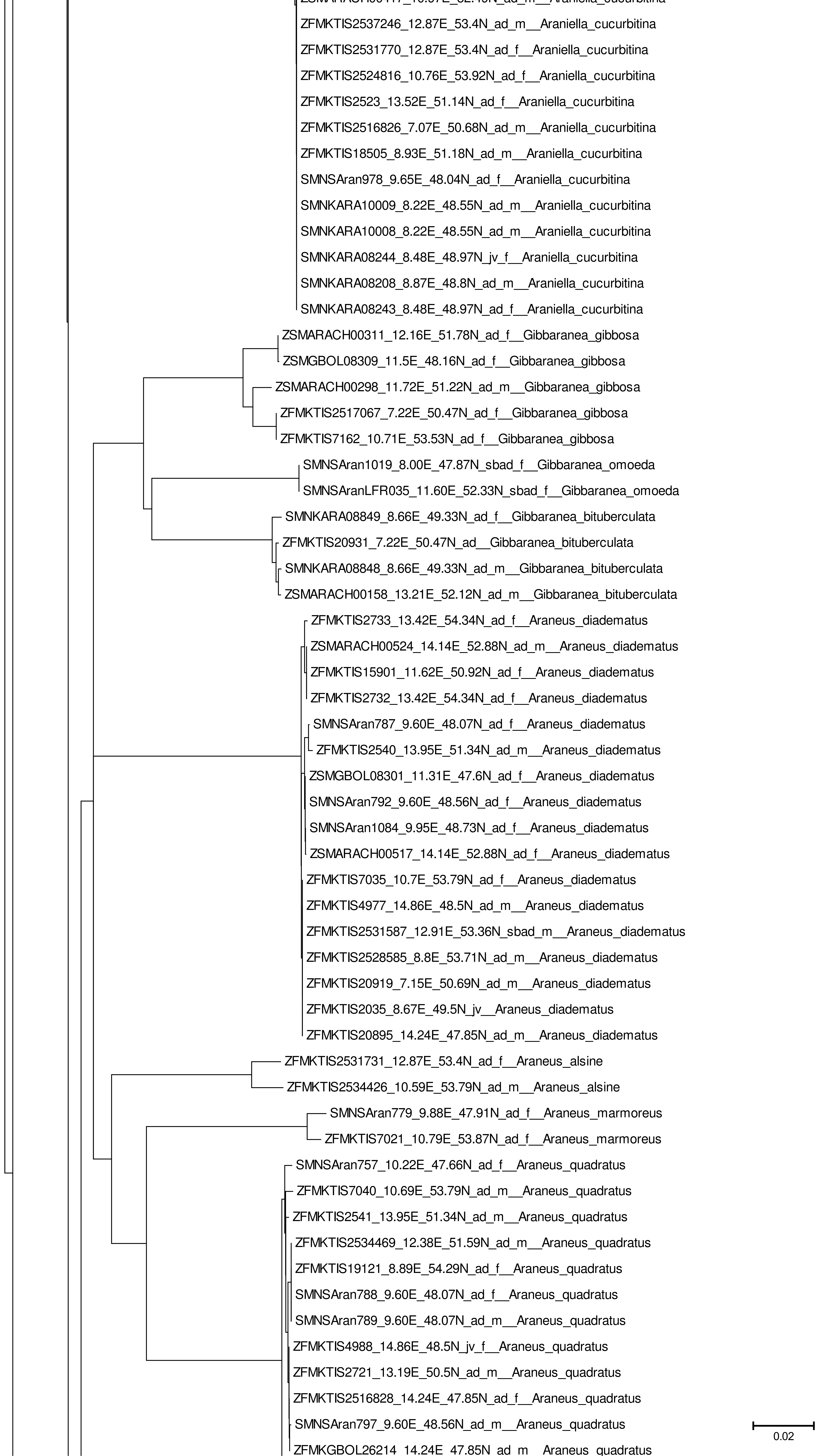

ZFMKTIS2537246_12.87E_53.4N_ad_m__Araniella_cucurbitina
ZFMKTIS2531770_12.87E_53.4N_ad_f__Araniella_cucurbitina
ZFMKTIS2524816_10.76E_53.92N_ad_f__Araniella_cucurbitina
ZFMKTIS2523_13.52E_51.14N_ad_f__Araniella_cucurbitina
ZFMKTIS2516826_7.07E_50.68N_ad_m__Araniella_cucurbitina
ZFMKTIS18505_8.93E_51.18N_ad_m__Araniella_cucurbitina
SMNSAran978_9.65E_48.04N_ad_f__Araniella_cucurbitina
SMNKARA10009_8.22E_48.55N_ad_m__Araniella_cucurbitina
SMNKARA10008_8.22E_48.55N_ad_m__Araniella_cucurbitina
SMNKARA08244_8.48E_48.97N_jv_f__Araniella_cucurbitina
SMNKARA08208_8.87E_48.8N_ad_m__Araniella_cucurbitina
SMNKARA08243_8.48E_48.97N_ad_f__Araniella_cucurbitina
ZSMARACH00311_12.16E_51.78N_ad_f__Gibbaranea_gibbosa
ZSMGBOL08309_11.5E_48.16N_ad_f__Gibbaranea_gibbosa
ZSMARACH00298_11.72E_51.22N_ad_m__Gibbaranea_gibbosa
ZFMKTIS2517067_7.22E_50.47N_ad_f__Gibbaranea_gibbosa
ZFMKTIS7162_10.71E_53.53N_ad_f__Gibbaranea_gibbosa
SMNSAran1019_8.00E_47.87N_sbad_f__Gibbaranea_omoeda
SMNSAranLFR035_11.60E_52.33N_sbad_f__Gibbaranea_omoeda
SMNKARA08849_8.66E_49.33N_ad_f__Gibbaranea_bituberculata
ZFMKTIS20931_7.22E_50.47N_ad__Gibbaranea_bituberculata
SMNKARA08848_8.66E_49.33N_ad_m__Gibbaranea_bituberculata
ZSMARACH00158_13.21E_52.12N_ad_m__Gibbaranea_bituberculata
ZFMKTIS2733_13.42E_54.34N_ad_f__Araneus_diadematus
ZSMARACH00524_14.14E_52.88N_ad_m__Araneus_diadematus
ZFMKTIS15901_11.62E_50.92N_ad_f__Araneus_diadematus
ZFMKTIS2732_13.42E_54.34N_ad_f__Araneus_diadematus
SMNSAran787_9.60E_48.07N_ad_f__Araneus_diadematus
ZFMKTIS2540_13.95E_51.34N_ad_m__Araneus_diadematus
ZSMGBOL08301_11.31E_47.6N_ad_f__Araneus_diadematus
SMNSAran792_9.60E_48.56N_ad_f__Araneus_diadematus
SMNSAran1084_9.95E_48.73N_ad_f__Araneus_diadematus
ZSMARACH00517_14.14E_52.88N_ad_f__Araneus_diadematus
ZFMKTIS7035_10.7E_53.79N_ad_f__Araneus_diadematus
ZFMKTIS4977_14.86E_48.5N_ad_m__Araneus_diadematus
ZFMKTIS2531587_12.91E_53.36N_sbad_m__Araneus_diadematus
ZFMKTIS2528585_8.8E_53.71N_ad_m__Araneus_diadematus
ZFMKTIS20919_7.15E_50.69N_ad_m__Araneus_diadematus
ZFMKTIS2035_8.67E_49.5N_jv__Araneus_diadematus
ZFMKTIS20895_14.24E_47.85N_ad_m__Araneus_diadematus
ZFMKTIS2531731_12.87E_53.4N_ad_f__Araneus_alsine
ZFMKTIS2534426_10.59E_53.79N_ad_m__Araneus_alsine
SMNSAran779_9.88E_47.91N_ad_f__Araneus_marmoreus
ZFMKTIS7021_10.79E_53.87N_ad_f__Araneus_marmoreus
SMNSAran757_10.22E_47.66N_ad_f__Araneus_quadratus
ZFMKTIS7040_10.69E_53.79N_ad_m__Araneus_quadratus
ZFMKTIS2541_13.95E_51.34N_ad_m__Araneus_quadratus
ZFMKTIS2534469_12.38E_51.59N_ad_m__Araneus_quadratus
ZFMKTIS19121_8.89E_54.29N_ad_f__Araneus_quadratus
SMNSAran788_9.60E_48.07N_ad_f__Araneus_quadratus
SMNSAran789_9.60E_48.07N_ad_m__Araneus_quadratus
ZFMKTIS4988_14.86E_48.5N_jv_f__Araneus_quadratus
ZFMKTIS2721_13.19E_50.5N_ad_m__Araneus_quadratus
ZFMKTIS2516828_14.24E_47.85N_ad_f__Araneus_quadratus
SMNSAran797_9.60E_48.56N_ad_m__Araneus_quadratus
ZFMKGBOL26214_14.24E_47.85N_ad_m__Araneus_quadratus
0.02

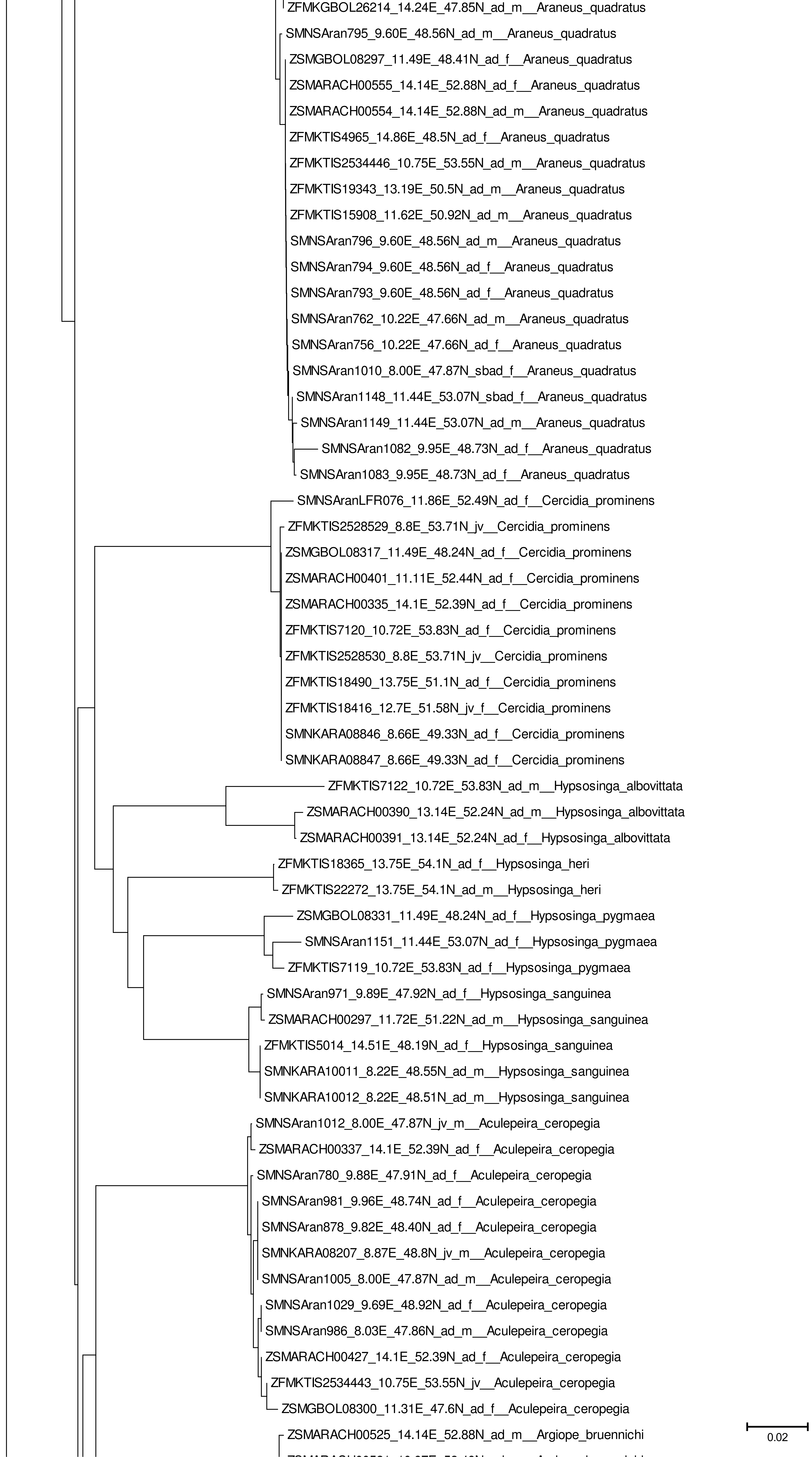

ZFMKGBOL26214_14.24E_47.85N_ad_m__Araneus_quadratus
SMNSAran795_9.60E_48.56N_ad_m__Araneus_quadratus
ZSMGBOL08297_11.49E_48.41N_ad_f__Araneus_quadratus
ZSMARACH00555_14.14E_52.88N_ad_f__Araneus_quadratus
ZSMARACH00554_14.14E_52.88N_ad_m__Araneus_quadratus
ZFMKTIS4965_14.86E_48.5N_ad_f__Araneus_quadratus
ZFMKTIS2534446_10.75E_53.55N_ad_m__Araneus_quadratus
ZFMKTIS19343_13.19E_50.5N_ad_m__Araneus_quadratus
ZFMKTIS15908_11.62E_50.92N_ad_m__Araneus_quadratus
SMNSAran796_9.60E_48.56N_ad_m__Araneus_quadratus
SMNSAran794_9.60E_48.56N_ad_f__Araneus_quadratus
SMNSAran793_9.60E_48.56N_ad_f__Araneus_quadratus
SMNSAran762_10.22E_47.66N_ad_m__Araneus_quadratus
SMNSAran756_10.22E_47.66N_ad_f__Araneus_quadratus
SMNSAran1010_8.00E_47.87N_sbad_f__Araneus_quadratus
SMNSAran1148_11.44E_53.07N_sbad_f__Araneus_quadratus
SMNSAran1149_11.44E_53.07N_ad_m__Araneus_quadratus
SMNSAran1082_9.95E_48.73N_ad_f__Araneus_quadratus
SMNSAran1083_9.95E_48.73N_ad_f__Araneus_quadratus
SMNSAranLFR076_11.86E_52.49N_ad_f__Cercidia_prominens
ZFMKTIS2528529_8.8E_53.71N_jv__Cercidia_prominens
ZSMGBOL08317_11.49E_48.24N_ad_f__Cercidia_prominens
ZSMARACH00401_11.11E_52.44N_ad_f__Cercidia_prominens
ZSMARACH00335_14.1E_52.39N_ad_f__Cercidia_prominens
ZFMKTIS7120_10.72E_53.83N_ad_f__Cercidia_prominens
ZFMKTIS2528530_8.8E_53.71N_jv__Cercidia_prominens
ZFMKTIS18490_13.75E_51.1N_ad_f__Cercidia_prominens
ZFMKTIS18416_12.7E_51.58N_jv_f__Cercidia_prominens
SMNKARA08846_8.66E_49.33N_ad_f__Cercidia_prominens
SMNKARA08847_8.66E_49.33N_ad_f__Cercidia_prominens
ZFMKTIS7122_10.72E_53.83N_ad_m__Hypsosinga_albovittata
ZSMARACH00390_13.14E_52.24N_ad_m__Hypsosinga_albovittata
ZSMARACH00391_13.14E_52.24N_ad_f__Hypsosinga_albovittata
ZFMKTIS18365_13.75E_54.1N_ad_f__Hypsosinga_heri
ZFMKTIS22272_13.75E_54.1N_ad_m__Hypsosinga_heri
ZSMGBOL08331_11.49E_48.24N_ad_f__Hypsosinga_pygmaea
SMNSAran1151_11.44E_53.07N_ad_f__Hypsosinga_pygmaea
ZFMKTIS7119_10.72E_53.83N_ad_f__Hypsosinga_pygmaea
SMNSAran971_9.89E_47.92N_ad_f__Hypsosinga_sanguinea
ZSMARACH00297_11.72E_51.22N_ad_m__Hypsosinga_sanguinea
ZFMKTIS5014_14.51E_48.19N_ad_f__Hypsosinga_sanguinea
SMNKARA10011_8.22E_48.55N_ad_m__Hypsosinga_sanguinea
SMNKARA10012_8.22E_48.51N_ad_m__Hypsosinga_sanguinea
SMNSAran1012_8.00E_47.87N_jv_m__Aculepeira_ceropegia
ZSMARACH00337_14.1E_52.39N_ad_f__Aculepeira_ceropegia
SMNSAran780_9.88E_47.91N_ad_f__Aculepeira_ceropegia
SMNSAran981_9.96E_48.74N_ad_f__Aculepeira_ceropegia
SMNSAran878_9.82E_48.40N_ad_f__Aculepeira_ceropegia
SMNKARA08207_8.87E_48.8N_jv_m__Aculepeira_ceropegia
SMNSAran1005_8.00E_47.87N_ad_m__Aculepeira_ceropegia
SMNSAran1029_9.69E_48.92N_ad_f__Aculepeira_ceropegia
SMNSAran986_8.03E_47.86N_ad_m__Aculepeira_ceropegia
ZSMARACH00427_14.1E_52.39N_ad_f__Aculepeira_ceropegia
ZFMKTIS2534443_10.75E_53.55N_jv__Aculepeira_ceropegia
ZSMGBOL08300_11.31E_47.6N_ad_f__Aculepeira_ceropegia
ZSMARACH00525_14.14E_52.88N_ad_m__Argiope_bruennichi
0.02

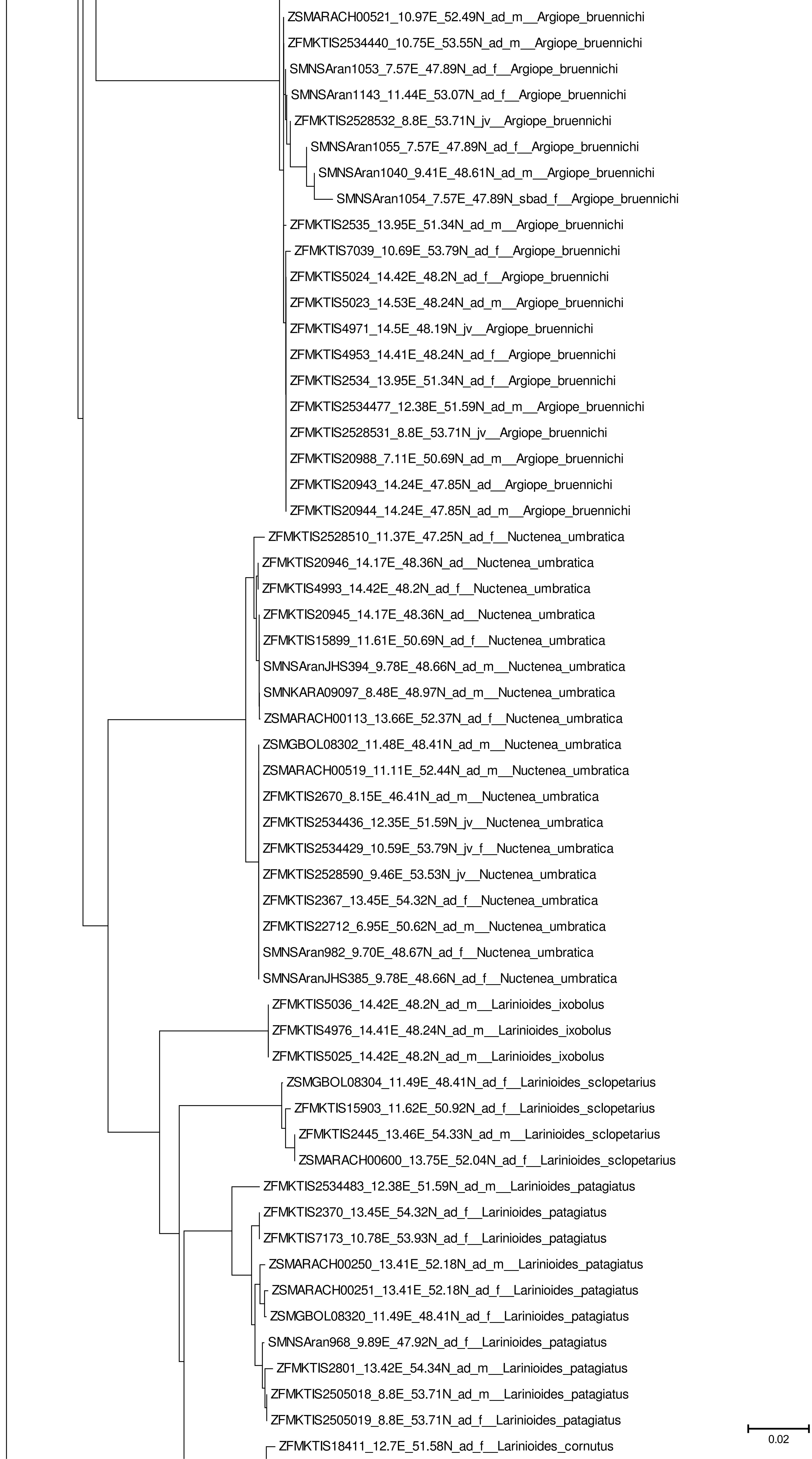
ZSMARACH00521_10.97E_52.49N_ad_m__Argiope_bruennichi
ZFMKTIS2534440_10.75E_53.55N_ad_m__Argiope_bruennichi
SMNSAran1053_7.57E_47.89N_ad_f__Argiope_bruennichi
SMNSAran1143_11.44E_53.07N_ad_f__Argiope_bruennichi
ZFMKTIS2528532_8.8E_53.71N_jv__Argiope_bruennichi
SMNSAran1055_7.57E_47.89N_ad_f__Argiope_bruennichi
SMNSAran1040_9.41E_48.61N_ad_m__Argiope_bruennichi
SMNSAran1054_7.57E_47.89N_sbad_f__Argiope_bruennichi
ZFMKTIS2535_13.95E_51.34N_ad_m__Argiope_bruennichi
ZFMKTIS7039_10.69E_53.79N_ad_f__Argiope_bruennichi
ZFMKTIS5024_14.42E_48.2N_ad_f__Argiope_bruennichi
ZFMKTIS5023_14.53E_48.24N_ad_m__Argiope_bruennichi
ZFMKTIS4971_14.5E_48.19N_jv__Argiope_bruennichi
ZFMKTIS4953_14.41E_48.24N_ad_f__Argiope_bruennichi
ZFMKTIS2534_13.95E_51.34N_ad_f__Argiope_bruennichi
ZFMKTIS2534477_12.38E_51.59N_ad_m__Argiope_bruennichi
ZFMKTIS2528531_8.8E_53.71N_jv__Argiope_bruennichi
ZFMKTIS20988_7.11E_50.69N_ad_m__Argiope_bruennichi
ZFMKTIS20943_14.24E_47.85N_ad__Argiope_bruennichi
ZFMKTIS20944_14.24E_47.85N_ad_m__Argiope_bruennichi
ZFMKTIS2528510_11.37E_47.25N_ad_f__Nuctenea_umbratica
ZFMKTIS20946_14.17E_48.36N_ad__Nuctenea_umbratica
ZFMKTIS4993_14.42E_48.2N_ad_f__Nuctenea_umbratica
ZFMKTIS20945_14.17E_48.36N_ad__Nuctenea_umbratica
ZFMKTIS15899_11.61E_50.69N_ad_f__Nuctenea_umbratica
SMNSAranJHS394_9.78E_48.66N_ad_m__Nuctenea_umbratica
SMNKARA09097_8.48E_48.97N_ad_m__Nuctenea_umbratica
ZSMARACH00113_13.66E_52.37N_ad_f__Nuctenea_umbratica
ZSMGBOL08302_11.48E_48.41N_ad_m__Nuctenea_umbratica
ZSMARACH00519_11.11E_52.44N_ad_m__Nuctenea_umbratica
ZFMKTIS2670_8.15E_46.41N_ad_m__Nuctenea_umbratica
ZFMKTIS2534436_12.35E_51.59N_jv__Nuctenea_umbratica
ZFMKTIS2534429_10.59E_53.79N_jv_f__Nuctenea_umbratica
ZFMKTIS2528590_9.46E_53.53N_jv__Nuctenea_umbratica
ZFMKTIS2367_13.45E_54.32N_ad_f__Nuctenea_umbratica
ZFMKTIS22712_6.95E_50.62N_ad_m__Nuctenea_umbratica
SMNSAran982_9.70E_48.67N_ad_f__Nuctenea_umbratica
SMNSAranJHS385_9.78E_48.66N_ad_f__Nuctenea_umbratica
ZFMKTIS5036_14.42E_48.2N_ad_m__Larinioides_ixobolus
ZFMKTIS4976_14.41E_48.24N_ad_m__Larinioides_ixobolus
ZFMKTIS5025_14.42E_48.2N_ad_m__Larinioides_ixobolus
ZSMGBOL08304_11.49E_48.41N_ad_f__Larinioides_sclopetarius
ZFMKTIS15903_11.62E_50.92N_ad_f__Larinioides_sclopetarius
ZFMKTIS2445_13.46E_54.33N_ad_m__Larinioides_sclopetarius
ZSMARACH00600_13.75E_52.04N_ad_f__Larinioides_sclopetarius
ZFMKTIS2534483_12.38E_51.59N_ad_m__Larinioides_patagiatus
ZFMKTIS2370_13.45E_54.32N_ad_f__Larinioides_patagiatus
ZFMKTIS7173_10.78E_53.93N_ad_f__Larinioides_patagiatus
ZSMARACH00250_13.41E_52.18N_ad_m__Larinioides_patagiatus
ZSMARACH00251_13.41E_52.18N_ad_f__Larinioides_patagiatus
ZSMGBOL08320_11.49E_48.41N_ad_f__Larinioides_patagiatus
SMNSAran968_9.89E_47.92N_ad_f__Larinioides_patagiatus
ZFMKTIS2801_13.42E_54.34N_ad_m__Larinioides_patagiatus
ZFMKTIS2505018_8.8E_53.71N_ad_m__Larinioides_patagiatus
ZFMKTIS2505019_8.8E_53.71N_ad_f__Larinioides_patagiatus
ZFMKTIS18411_12.7E_51.58N_ad_f__Larinioides_cornutus
0.02

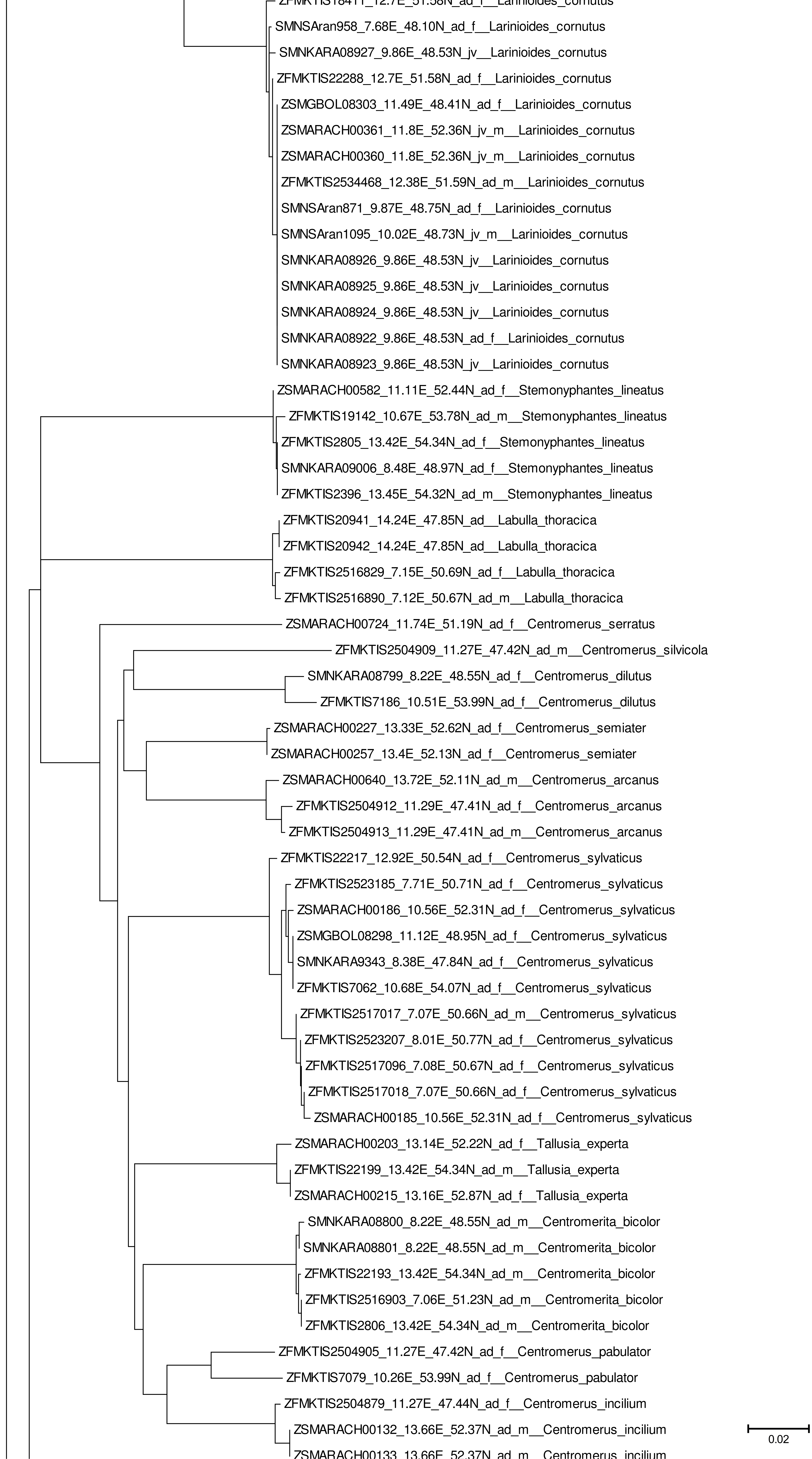
SMNSAran958_7.68E_48.10N_ad_f__Larinioides_cornutus
SMNKARA08927_9.86E_48.53N_jv__Larinioides_cornutus
ZFMKTIS22288_12.7E_51.58N_ad_f__Larinioides_cornutus
ZSMGBOL08303_11.49E_48.41N_ad_f__Larinioides_cornutus
ZSMARACH00361_11.8E_52.36N_jv_m__Larinioides_cornutus
ZSMARACH00360_11.8E_52.36N_jv_m__Larinioides_cornutus
ZFMKTIS2534468_12.38E_51.59N_ad_m__Larinioides_cornutus
SMNSAran871_9.87E_48.75N_ad_f__Larinioides_cornutus
SMNSAran1095_10.02E_48.73N_jv_m__Larinioides_cornutus
SMNKARA08926_9.86E_48.53N_jv__Larinioides_cornutus
SMNKARA08925_9.86E_48.53N_jv__Larinioides_cornutus
SMNKARA08924_9.86E_48.53N_jv__Larinioides_cornutus
SMNKARA08922_9.86E_48.53N_ad_f__Larinioides_cornutus
SMNKARA08923_9.86E_48.53N_jv__Larinioides_cornutus
ZSMARACH00582_11.11E_52.44N_ad_f__Stemonyphantes_lineatus
ZFMKTIS19142_10.67E_53.78N_ad_m__Stemonyphantes_lineatus
ZFMKTIS2805_13.42E_54.34N_ad_f__Stemonyphantes_lineatus
SMNKARA09006_8.48E_48.97N_ad_f__Stemonyphantes_lineatus
ZFMKTIS2396_13.45E_54.32N_ad_m__Stemonyphantes_lineatus
ZFMKTIS20941_14.24E_47.85N_ad__Labulla_thoracica
ZFMKTIS20942_14.24E_47.85N_ad__Labulla_thoracica
ZFMKTIS2516829_7.15E_50.69N_ad_f__Labulla_thoracica
ZFMKTIS2516890_7.12E_50.67N_ad_m__Labulla_thoracica
ZSMARACH00724_11.74E_51.19N_ad_f__Centromerus_serratus
ZFMKTIS2504909_11.27E_47.42N_ad_m__Centromerus_silvicola
SMNKARA08799_8.22E_48.55N_ad_f__Centromerus_dilutus
ZFMKTIS7186_10.51E_53.99N_ad_f__Centromerus_dilutus
ZSMARACH00227_13.33E_52.62N_ad_f__Centromerus_semiater
ZSMARACH00257_13.4E_52.13N_ad_f__Centromerus_semiater
ZSMARACH00640_13.72E_52.11N_ad_m__Centromerus_arcanus
ZFMKTIS2504912_11.29E_47.41N_ad_f__Centromerus_arcanus
ZFMKTIS2504913_11.29E_47.41N_ad_m__Centromerus_arcanus
ZFMKTIS22217_12.92E_50.54N_ad_f__Centromerus_sylvaticus
ZFMKTIS2523185_7.71E_50.71N_ad_f__Centromerus_sylvaticus
ZSMARACH00186_10.56E_52.31N_ad_f__Centromerus_sylvaticus
ZSMGBOL08298_11.12E_48.95N_ad_f__Centromerus_sylvaticus
SMNKARA9343_8.38E_47.84N_ad_f__Centromerus_sylvaticus
ZFMKTIS7062_10.68E_54.07N_ad_f__Centromerus_sylvaticus
ZFMKTIS2517017_7.07E_50.66N_ad_m__Centromerus_sylvaticus
ZFMKTIS2523207_8.01E_50.77N_ad_f__Centromerus_sylvaticus
ZFMKTIS2517096_7.08E_50.67N_ad_f__Centromerus_sylvaticus
ZFMKTIS2517018_7.07E_50.66N_ad_f__Centromerus_sylvaticus
ZSMARACH00185_10.56E_52.31N_ad_f__Centromerus_sylvaticus
ZSMARACH00203_13.14E_52.22N_ad_f__Tallusia_experta
ZFMKTIS22199_13.42E_54.34N_ad_m__Tallusia_experta
ZSMARACH00215_13.16E_52.87N_ad_f__Tallusia_experta
SMNKARA08800_8.22E_48.55N_ad_m__Centromerita_bicolor
SMNKARA08801_8.22E_48.55N_ad_m__Centromerita_bicolor
ZFMKTIS22193_13.42E_54.34N_ad_m__Centromerita_bicolor
ZFMKTIS2516903_7.06E_51.23N_ad_m__Centromerita_bicolor
ZFMKTIS2806_13.42E_54.34N_ad_m__Centromerita_bicolor
ZFMKTIS2504905_11.27E_47.42N_ad_f__Centromerus_pabulator
ZFMKTIS7079_10.26E_53.99N_ad_f__Centromerus_pabulator
ZFMKTIS2504879_11.27E_47.44N_ad_f__Centromerus_incilium
ZSMARACH00132_13.66E_52.37N_ad_m__Centromerus_incilium
0.02

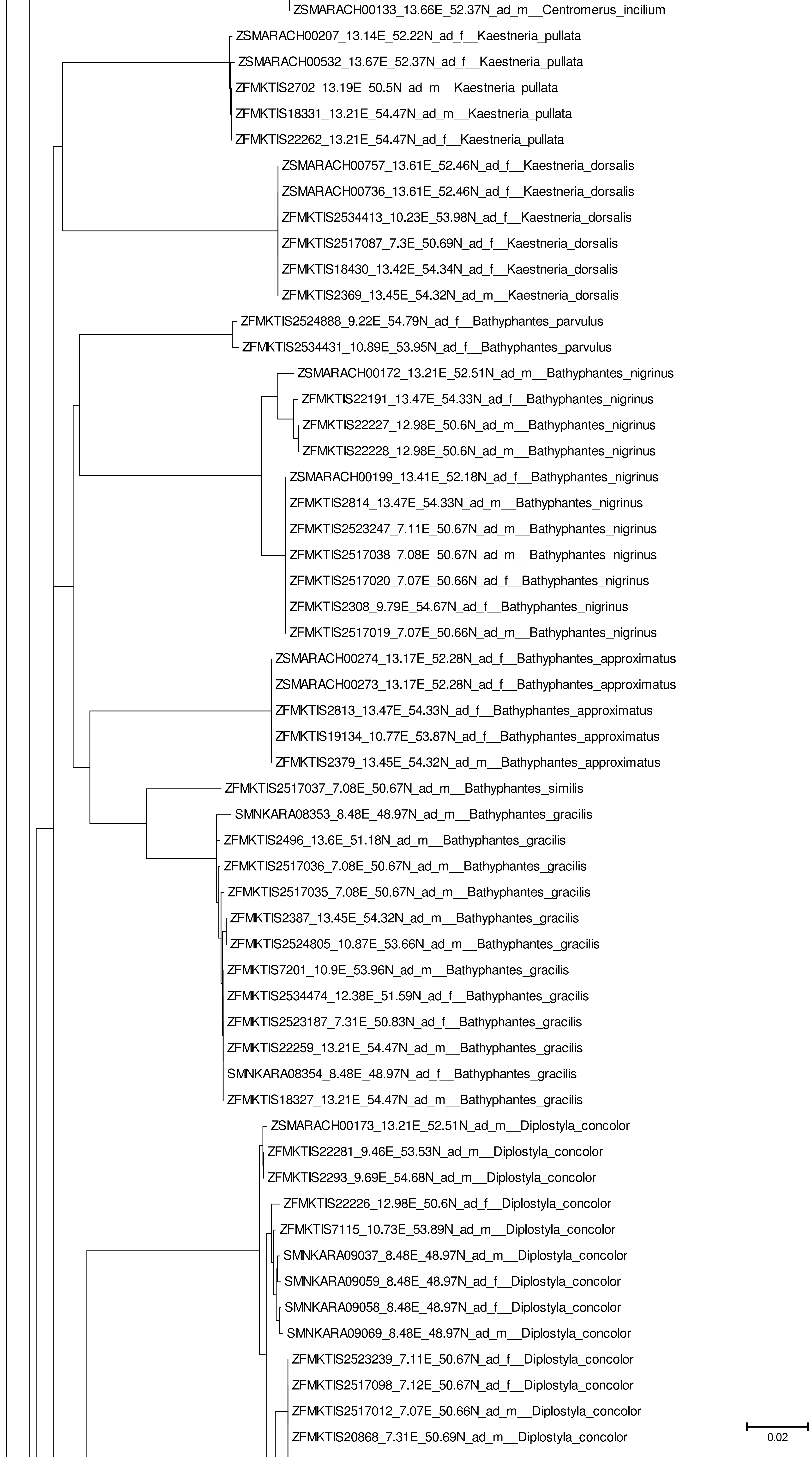
ZSMARACH00133_13.66E_52.37N_ad_m__Centromerus_incilium
ZSMARACH00207_13.14E_52.22N_ad_f__Kaestneria_pullata
ZSMARACH00532_13.67E_52.37N_ad_f__Kaestneria_pullata
ZFMKTIS2702_13.19E_50.5N_ad_m__Kaestneria_pullata
ZFMKTIS18331_13.21E_54.47N_ad_m__Kaestneria_pullata
ZFMKTIS22262_13.21E_54.47N_ad_f__Kaestneria_pullata
ZSMARACH00757_13.61E_52.46N_ad_f__Kaestneria_dorsalis
ZSMARACH00736_13.61E_52.46N_ad_f__Kaestneria_dorsalis
ZFMKTIS2534413_10.23E_53.98N_ad_f__Kaestneria_dorsalis
ZFMKTIS2517087_7.3E_50.69N_ad_f__Kaestneria_dorsalis
ZFMKTIS18430_13.42E_54.34N_ad_f__Kaestneria_dorsalis
ZFMKTIS2369_13.45E_54.32N_ad_m__Kaestneria_dorsalis
ZFMKTIS2524888_9.22E_54.79N_ad_f__Bathyphantes_parvulus
ZFMKTIS2534431_10.89E_53.95N_ad_f__Bathyphantes_parvulus
ZSMARACH00172_13.21E_52.51N_ad_m__Bathyphantes_nigrinus
ZFMKTIS22191_13.47E_54.33N_ad_f__Bathyphantes_nigrinus
ZFMKTIS22227_12.98E_50.6N_ad_m__Bathyphantes_nigrinus
ZFMKTIS22228_12.98E_50.6N_ad_m__Bathyphantes_nigrinus
ZSMARACH00199_13.41E_52.18N_ad_f__Bathyphantes_nigrinus
ZFMKTIS2814_13.47E_54.33N_ad_m__Bathyphantes_nigrinus
ZFMKTIS2523247_7.11E_50.67N_ad_m__Bathyphantes_nigrinus
ZFMKTIS2517038_7.08E_50.67N_ad_m__Bathyphantes_nigrinus
ZFMKTIS2517020_7.07E_50.66N_ad_f__Bathyphantes_nigrinus
ZFMKTIS2308_9.79E_54.67N_ad_f__Bathyphantes_nigrinus
ZFMKTIS2517019_7.07E_50.66N_ad_m__Bathyphantes_nigrinus
ZSMARACH00274_13.17E_52.28N_ad_f__Bathyphantes_approximatus
ZSMARACH00273_13.17E_52.28N_ad_f__Bathyphantes_approximatus
ZFMKTIS2813_13.47E_54.33N_ad_f__Bathyphantes_approximatus
ZFMKTIS19134_10.77E_53.87N_ad_f__Bathyphantes_approximatus
ZFMKTIS2379_13.45E_54.32N_ad_m__Bathyphantes_approximatus
ZFMKTIS2517037_7.08E_50.67N_ad_m__Bathyphantes_similis
SMNKARA08353_8.48E_48.97N_ad_m__Bathyphantes_gracilis
ZFMKTIS2496_13.6E_51.18N_ad_m__Bathyphantes_gracilis
ZFMKTIS2517036_7.08E_50.67N_ad_m__Bathyphantes_gracilis
ZFMKTIS2517035_7.08E_50.67N_ad_m__Bathyphantes_gracilis
ZFMKTIS2387_13.45E_54.32N_ad_m__Bathyphantes_gracilis
ZFMKTIS2524805_10.87E_53.66N_ad_m__Bathyphantes_gracilis
ZFMKTIS7201_10.9E_53.96N_ad_m__Bathyphantes_gracilis
ZFMKTIS2534474_12.38E_51.59N_ad_f__Bathyphantes_gracilis
ZFMKTIS2523187_7.31E_50.83N_ad_f__Bathyphantes_gracilis
ZFMKTIS22259_13.21E_54.47N_ad_m__Bathyphantes_gracilis
SMNKARA08354_8.48E_48.97N_ad_f__Bathyphantes_gracilis
ZFMKTIS18327_13.21E_54.47N_ad_m__Bathyphantes_gracilis
ZSMARACH00173_13.21E_52.51N_ad_m__Diplostyla_concolor
ZFMKTIS22281_9.46E_53.53N_ad_m__Diplostyla_concolor
ZFMKTIS2293_9.69E_54.68N_ad_m__Diplostyla_concolor
ZFMKTIS22226_12.98E_50.6N_ad_f__Diplostyla_concolor
ZFMKTIS7115_10.73E_53.89N_ad_m__Diplostyla_concolor
SMNKARA09037_8.48E_48.97N_ad_m__Diplostyla_concolor
SMNKARA09059_8.48E_48.97N_ad_f__Diplostyla_concolor
SMNKARA09058_8.48E_48.97N_ad_f__Diplostyla_concolor
SMNKARA09069_8.48E_48.97N_ad_m__Diplostyla_concolor
ZFMKTIS2523239_7.11E_50.67N_ad_f__Diplostyla_concolor
ZFMKTIS2517098_7.12E_50.67N_ad_f__Diplostyla_concolor
ZFMKTIS2517012_7.07E_50.66N_ad_m__Diplostyla_concolor
ZFMKTIS20868_7.31E_50.69N_ad_m__Diplostyla_concolor
0.02

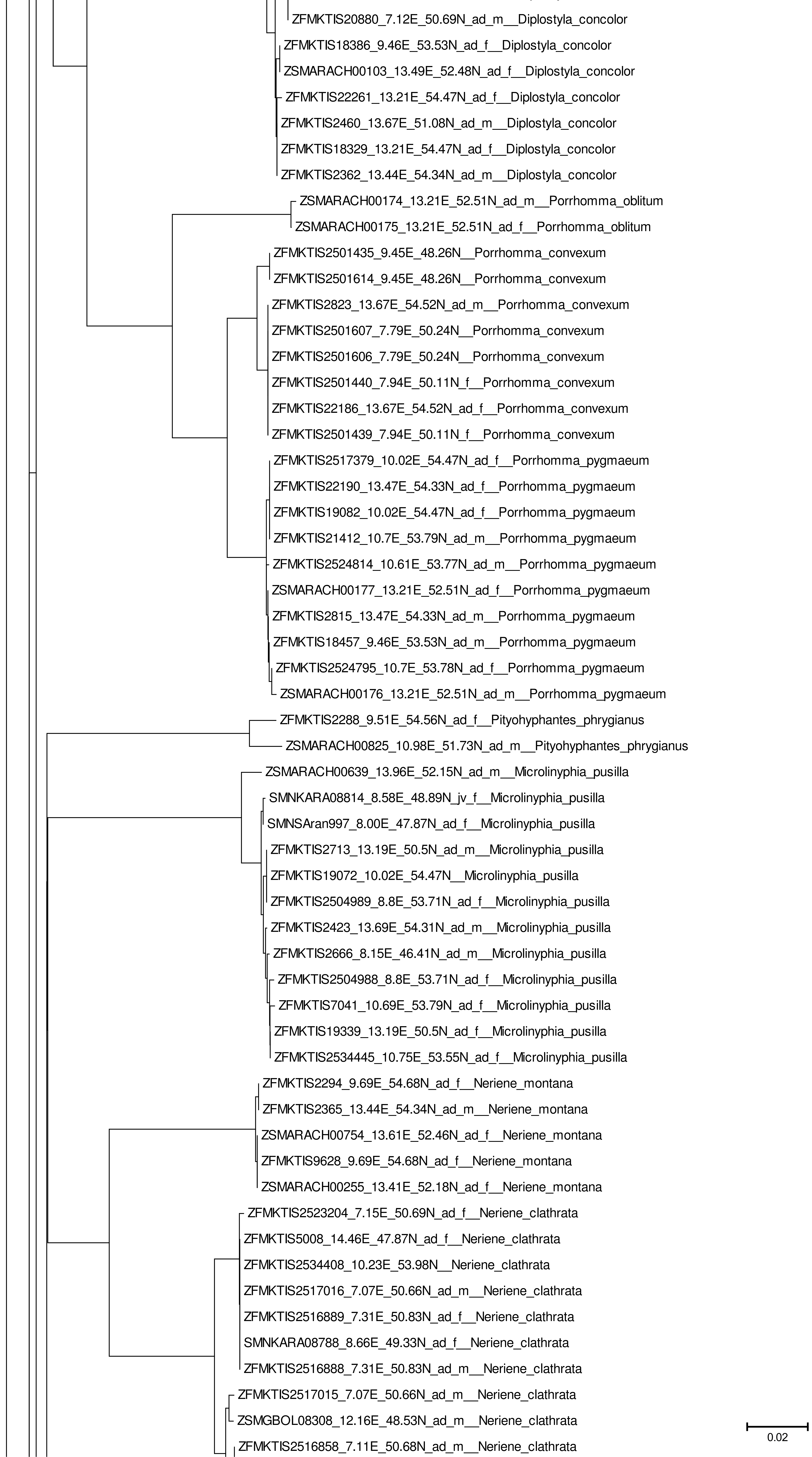
ZFMKTIS20880_7.12E_50.69N_ad_m__Diplostyla_concolor
ZFMKTIS18386_9.46E_53.53N_ad_f__Diplostyla_concolor
ZSMARACH00103_13.49E_52.48N_ad_f__Diplostyla_concolor
ZFMKTIS22261_13.21E_54.47N_ad_f__Diplostyla_concolor
ZFMKTIS2460_13.67E_51.08N_ad_m__Diplostyla_concolor
ZFMKTIS18329_13.21E_54.47N_ad_f__Diplostyla_concolor
ZFMKTIS2362_13.44E_54.34N_ad_m__Diplostyla_concolor
ZSMARACH00174_13.21E_52.51N_ad_m__Porrhomma_oblitum
ZSMARACH00175_13.21E_52.51N_ad_f__Porrhomma_oblitum
ZFMKTIS2501435_9.45E_48.26N__Porrhomma_convexum
ZFMKTIS2501614_9.45E_48.26N__Porrhomma_convexum
ZFMKTIS2823_13.67E_54.52N_ad_m__Porrhomma_convexum
ZFMKTIS2501607_7.79E_50.24N__Porrhomma_convexum
ZFMKTIS2501606_7.79E_50.24N__Porrhomma_convexum
ZFMKTIS2501440_7.94E_50.11N_f__Porrhomma_convexum
ZFMKTIS22186_13.67E_54.52N_ad_f__Porrhomma_convexum
ZFMKTIS2501439_7.94E_50.11N_f__Porrhomma_convexum
ZFMKTIS2517379_10.02E_54.47N_ad_f__Porrhomma_pygmaeum
ZFMKTIS22190_13.47E_54.33N_ad_f__Porrhomma_pygmaeum
ZFMKTIS19082_10.02E_54.47N_ad_f__Porrhomma_pygmaeum
ZFMKTIS21412_10.7E_53.79N_ad_m__Porrhomma_pygmaeum
ZFMKTIS2524814_10.61E_53.77N_ad_m__Porrhomma_pygmaeum
ZSMARACH00177_13.21E_52.51N_ad_f__Porrhomma_pygmaeum
ZFMKTIS2815_13.47E_54.33N_ad_m__Porrhomma_pygmaeum
ZFMKTIS18457_9.46E_53.53N_ad_m__Porrhomma_pygmaeum
ZFMKTIS2524795_10.7E_53.78N_ad_f__Porrhomma_pygmaeum
ZSMARACH00176_13.21E_52.51N_ad_m__Porrhomma_pygmaeum
ZFMKTIS2288_9.51E_54.56N_ad_f__Pityohyphantes_phrygianus
ZSMARACH00825_10.98E_51.73N_ad_m__Pityohyphantes_phrygianus
ZSMARACH00639_13.96E_52.15N_ad_m__Microlinyphia_pusilla
SMNKARA08814_8.58E_48.89N_jv_f__Microlinyphia_pusilla
SMNSAran997_8.00E_47.87N_ad_f__Microlinyphia_pusilla
ZFMKTIS2713_13.19E_50.5N_ad_m__Microlinyphia_pusilla
ZFMKTIS19072_10.02E_54.47N__Microlinyphia_pusilla
ZFMKTIS2504989_8.8E_53.71N_ad_f__Microlinyphia_pusilla
ZFMKTIS2423_13.69E_54.31N_ad_m__Microlinyphia_pusilla
ZFMKTIS2666_8.15E_46.41N_ad_m__Microlinyphia_pusilla
ZFMKTIS2504988_8.8E_53.71N_ad_f__Microlinyphia_pusilla
ZFMKTIS7041_10.69E_53.79N_ad_f__Microlinyphia_pusilla
ZFMKTIS19339_13.19E_50.5N_ad_f__Microlinyphia_pusilla
ZFMKTIS2534445_10.75E_53.55N_ad_f__Microlinyphia_pusilla
ZFMKTIS2294_9.69E_54.68N_ad_f__Neriene_montana
ZFMKTIS2365_13.44E_54.34N_ad_m__Neriene_montana
ZSMARACH00754_13.61E_52.46N_ad_f__Neriene_montana
ZFMKTIS9628_9.69E_54.68N_ad_f__Neriene_montana
ZSMARACH00255_13.41E_52.18N_ad_f__Neriene_montana
ZFMKTIS2523204_7.15E_50.69N_ad_f__Neriene_clathrata
ZFMKTIS5008_14.46E_47.87N_ad_f__Neriene_clathrata
ZFMKTIS2534408_10.23E_53.98N__Neriene_clathrata
ZFMKTIS2517016_7.07E_50.66N_ad_m__Neriene_clathrata
ZFMKTIS2516889_7.31E_50.83N_ad_f__Neriene_clathrata
SMNKARA08788_8.66E_49.33N_ad_f__Neriene_clathrata
ZFMKTIS2516888_7.31E_50.83N_ad_m__Neriene_clathrata
ZFMKTIS2517015_7.07E_50.66N_ad_m__Neriene_clathrata
ZSMGBOL08308_12.16E_48.53N_ad_m__Neriene_clathrata
ZFMKTIS2516858_7.11E_50.68N_ad_m__Neriene_clathrata
0.02

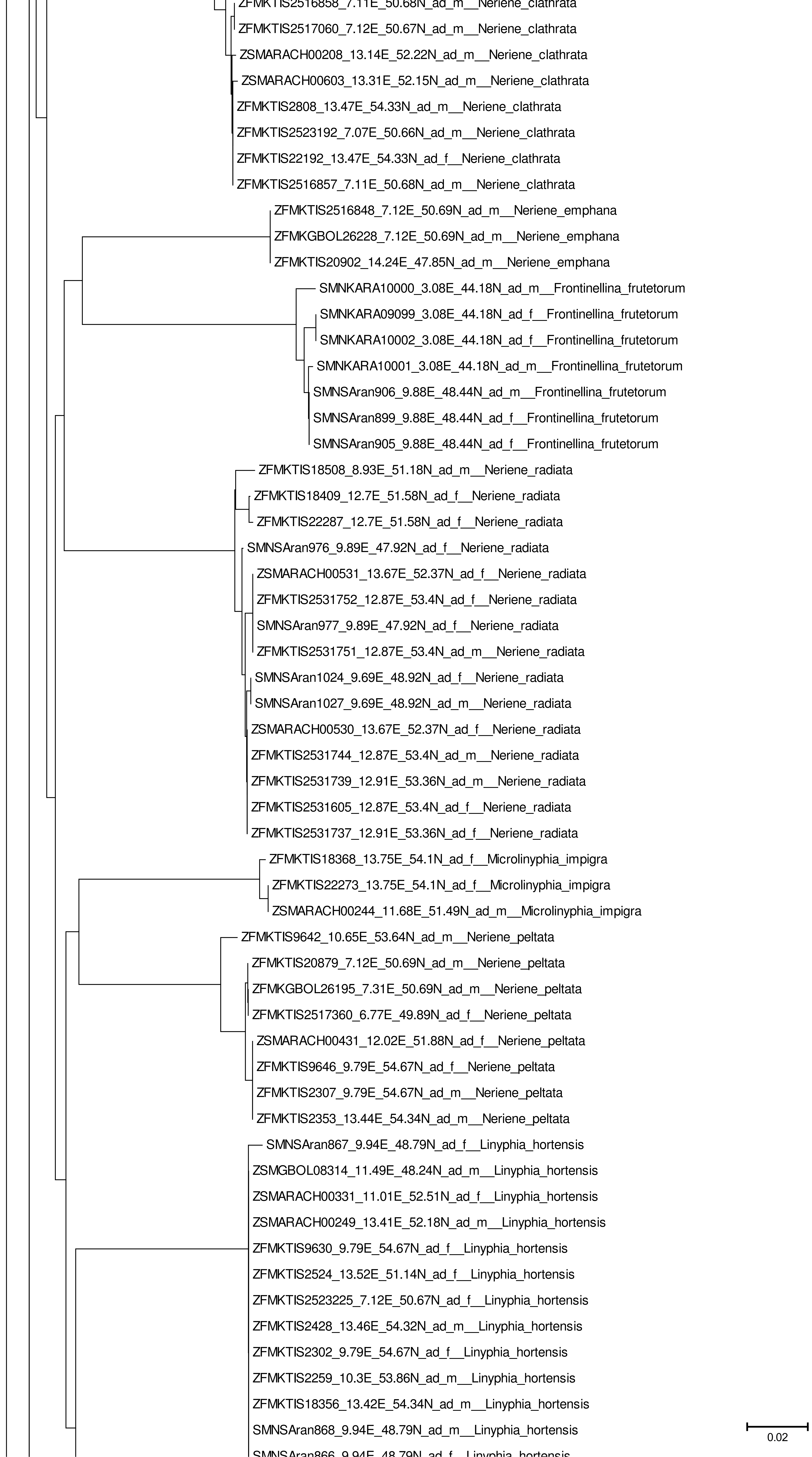
ZFMKTIS2516858_7.11E_50.68N_ad_m__Neriene_clathrata
ZFMKTIS2517060_7.12E_50.67N_ad_m__Neriene_clathrata
ZSMARACH00208_13.14E_52.22N_ad_m__Neriene_clathrata
ZSMARACH00603_13.31E_52.15N_ad_m__Neriene_clathrata
ZFMKTIS2808_13.47E_54.33N_ad_m__Neriene_clathrata
ZFMKTIS2523192_7.07E_50.66N_ad_m__Neriene_clathrata
ZFMKTIS22192_13.47E_54.33N_ad_f__Neriene_clathrata
ZFMKTIS2516857_7.11E_50.68N_ad_m__Neriene_clathrata
ZFMKTIS2516848_7.12E_50.69N_ad_m__Neriene_emphana
ZFMKGBOL26228_7.12E_50.69N_ad_m__Neriene_emphana
ZFMKTIS20902_14.24E_47.85N_ad_m__Neriene_emphana
SMNKARA10000_3.08E_44.18N_ad_m__Frontinellina_frutetorum
SMNKARA09099_3.08E_44.18N_ad_f__Frontinellina_frutetorum
SMNKARA10002_3.08E_44.18N_ad_f__Frontinellina_frutetorum
SMNKARA10001_3.08E_44.18N_ad_m__Frontinellina_frutetorum
SMNSAran906_9.88E_48.44N_ad_m__Frontinellina_frutetorum
SMNSAran899_9.88E_48.44N_ad_f__Frontinellina_frutetorum
SMNSAran905_9.88E_48.44N_ad_f__Frontinellina_frutetorum
ZFMKTIS18508_8.93E_51.18N_ad_m__Neriene_radiata
ZFMKTIS18409_12.7E_51.58N_ad_f__Neriene_radiata
ZFMKTIS22287_12.7E_51.58N_ad_f__Neriene_radiata
SMNSAran976_9.89E_47.92N_ad_f__Neriene_radiata
ZSMARACH00531_13.67E_52.37N_ad_f__Neriene_radiata
ZFMKTIS2531752_12.87E_53.4N_ad_f__Neriene_radiata
SMNSAran977_9.89E_47.92N_ad_f__Neriene_radiata
ZFMKTIS2531751_12.87E_53.4N_ad_m__Neriene_radiata
SMNSAran1024_9.69E_48.92N_ad_f__Neriene_radiata
SMNSAran1027_9.69E_48.92N_ad_m__Neriene_radiata
ZSMARACH00530_13.67E_52.37N_ad_f__Neriene_radiata
ZFMKTIS2531744_12.87E_53.4N_ad_m__Neriene_radiata
ZFMKTIS2531739_12.91E_53.36N_ad_m__Neriene_radiata
ZFMKTIS2531605_12.87E_53.4N_ad_f__Neriene_radiata
ZFMKTIS2531737_12.91E_53.36N_ad_f__Neriene_radiata
ZFMKTIS18368_13.75E_54.1N_ad_f__Microlinyphia_impigra
ZFMKTIS22273_13.75E_54.1N_ad_f__Microlinyphia_impigra
ZSMARACH00244_11.68E_51.49N_ad_m__Microlinyphia_impigra
ZFMKTIS9642_10.65E_53.64N_ad_m__Neriene_peltata
ZFMKTIS20879_7.12E_50.69N_ad_m__Neriene_peltata
ZFMKGBOL26195_7.31E_50.69N_ad_m__Neriene_peltata
ZFMKTIS2517360_6.77E_49.89N_ad_f__Neriene_peltata
ZSMARACH00431_12.02E_51.88N_ad_f__Neriene_peltata
ZFMKTIS9646_9.79E_54.67N_ad_f__Neriene_peltata
ZFMKTIS2307_9.79E_54.67N_ad_m__Neriene_peltata
ZFMKTIS2353_13.44E_54.34N_ad_m__Neriene_peltata
SMNSAran867_9.94E_48.79N_ad_f__Linyphia_hortensis
ZSMGBOL08314_11.49E_48.24N_ad_m__Linyphia_hortensis
ZSMARACH00331_11.01E_52.51N_ad_f__Linyphia_hortensis
ZSMARACH00249_13.41E_52.18N_ad_m__Linyphia_hortensis
ZFMKTIS9630_9.79E_54.67N_ad_f__Linyphia_hortensis
ZFMKTIS2524_13.52E_51.14N_ad_f__Linyphia_hortensis
ZFMKTIS2523225_7.12E_50.67N_ad_f__Linyphia_hortensis
ZFMKTIS2428_13.46E_54.32N_ad_m__Linyphia_hortensis
ZFMKTIS2302_9.79E_54.67N_ad_f__Linyphia_hortensis
ZFMKTIS2259_10.3E_53.86N_ad_m__Linyphia_hortensis
ZFMKTIS18356_13.42E_54.34N_ad_m__Linyphia_hortensis
SMNSAran868_9.94E_48.79N_ad_m__Linyphia_hortensis
SMNSAran866_9.94E_48.79N_ad_f__Linyphia_hortensis
0.02

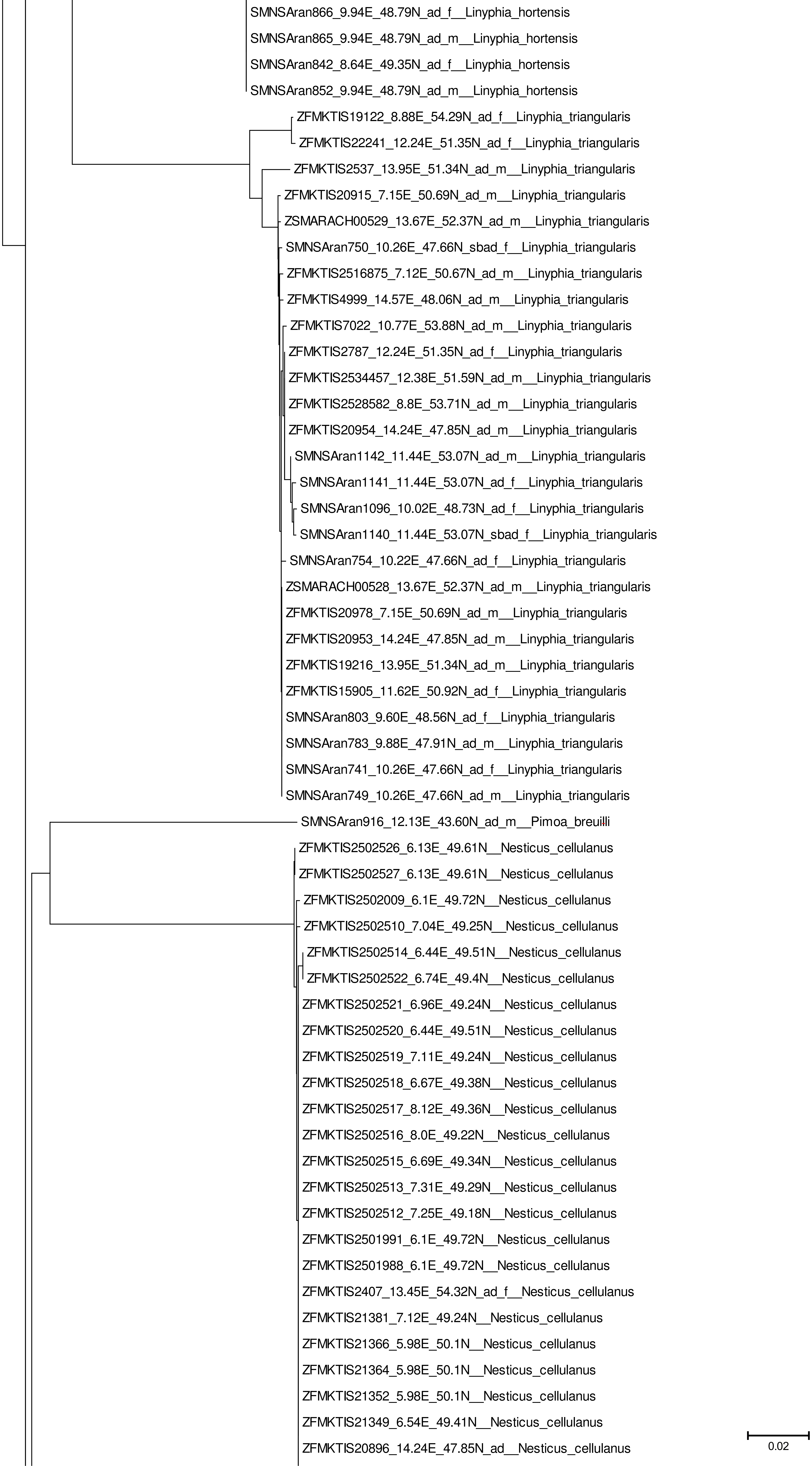

SMNSAran866_9.94E_48.79N_ad_f__Linyphia_hortensis
SMNSAran865_9.94E_48.79N_ad_m__Linyphia_hortensis
SMNSAran842_8.64E_49.35N_ad_f__Linyphia_hortensis
SMNSAran852_9.94E_48.79N_ad_m__Linyphia_hortensis
ZFMKTIS19122_8.88E_54.29N_ad_f__Linyphia_triangularis
ZFMKTIS22241_12.24E_51.35N_ad_f__Linyphia_triangularis
ZFMKTIS2537_13.95E_51.34N_ad_m__Linyphia_triangularis
ZFMKTIS20915_7.15E_50.69N_ad_m__Linyphia_triangularis
ZSMARACH00529_13.67E_52.37N_ad_m__Linyphia_triangularis
SMNSAran750_10.26E_47.66N_sbad_f__Linyphia_triangularis
ZFMKTIS2516875_7.12E_50.67N_ad_m__Linyphia_triangularis
ZFMKTIS4999_14.57E_48.06N_ad_m__Linyphia_triangularis
ZFMKTIS7022_10.77E_53.88N_ad_m__Linyphia_triangularis
ZFMKTIS2787_12.24E_51.35N_ad_f__Linyphia_triangularis
ZFMKTIS2534457_12.38E_51.59N_ad_m__Linyphia_triangularis
ZFMKTIS2528582_8.8E_53.71N_ad_m__Linyphia_triangularis
ZFMKTIS20954_14.24E_47.85N_ad_m__Linyphia_triangularis
SMNSAran1142_11.44E_53.07N_ad_m__Linyphia_triangularis
SMNSAran1141_11.44E_53.07N_ad_f__Linyphia_triangularis
SMNSAran1096_10.02E_48.73N_ad_f__Linyphia_triangularis
SMNSAran1140_11.44E_53.07N_sbad_f__Linyphia_triangularis
SMNSAran754_10.22E_47.66N_ad_f__Linyphia_triangularis
ZSMARACH00528_13.67E_52.37N_ad_m__Linyphia_triangularis
ZFMKTIS20978_7.15E_50.69N_ad_m__Linyphia_triangularis
ZFMKTIS20953_14.24E_47.85N_ad_m__Linyphia_triangularis
ZFMKTIS19216_13.95E_51.34N_ad_m__Linyphia_triangularis
ZFMKTIS15905_11.62E_50.92N_ad_f__Linyphia_triangularis
SMNSAran803_9.60E_48.56N_ad_f__Linyphia_triangularis
SMNSAran783_9.88E_47.91N_ad_m__Linyphia_triangularis
SMNSAran741_10.26E_47.66N_ad_f__Linyphia_triangularis
SMNSAran749_10.26E_47.66N_ad_m__Linyphia_triangularis
SMNSAran916_12.13E_43.60N_ad_m__Pimoa_breuilli
ZFMKTIS2502526_6.13E_49.61N__Nesticus_cellulanus
ZFMKTIS2502527_6.13E_49.61N__Nesticus_cellulanus
ZFMKTIS2502009_6.1E_49.72N__Nesticus_cellulanus
ZFMKTIS2502510_7.04E_49.25N__Nesticus_cellulanus
ZFMKTIS2502514_6.44E_49.51N__Nesticus_cellulanus
ZFMKTIS2502522_6.74E_49.4N__Nesticus_cellulanus
ZFMKTIS2502521_6.96E_49.24N__Nesticus_cellulanus
ZFMKTIS2502520_6.44E_49.51N__Nesticus_cellulanus
ZFMKTIS2502519_7.11E_49.24N__Nesticus_cellulanus
ZFMKTIS2502518_6.67E_49.38N__Nesticus_cellulanus
ZFMKTIS2502517_8.12E_49.36N__Nesticus_cellulanus
ZFMKTIS2502516_8.0E_49.22N__Nesticus_cellulanus
ZFMKTIS2502515_6.69E_49.34N__Nesticus_cellulanus
ZFMKTIS2502513_7.31E_49.29N__Nesticus_cellulanus
ZFMKTIS2502512_7.25E_49.18N__Nesticus_cellulanus
ZFMKTIS2501991_6.1E_49.72N__Nesticus_cellulanus
ZFMKTIS2501988_6.1E_49.72N__Nesticus_cellulanus
ZFMKTIS2407_13.45E_54.32N_ad_f__Nesticus_cellulanus
ZFMKTIS21381_7.12E_49.24N__Nesticus_cellulanus
ZFMKTIS21366_5.98E_50.1N__Nesticus_cellulanus
ZFMKTIS21364_5.98E_50.1N__Nesticus_cellulanus
ZFMKTIS21352_5.98E_50.1N__Nesticus_cellulanus
ZFMKTIS21349_6.54E_49.41N__Nesticus_cellulanus
ZFMKTIS20896_14.24E_47.85N_ad__Nesticus_cellulanus
0.02

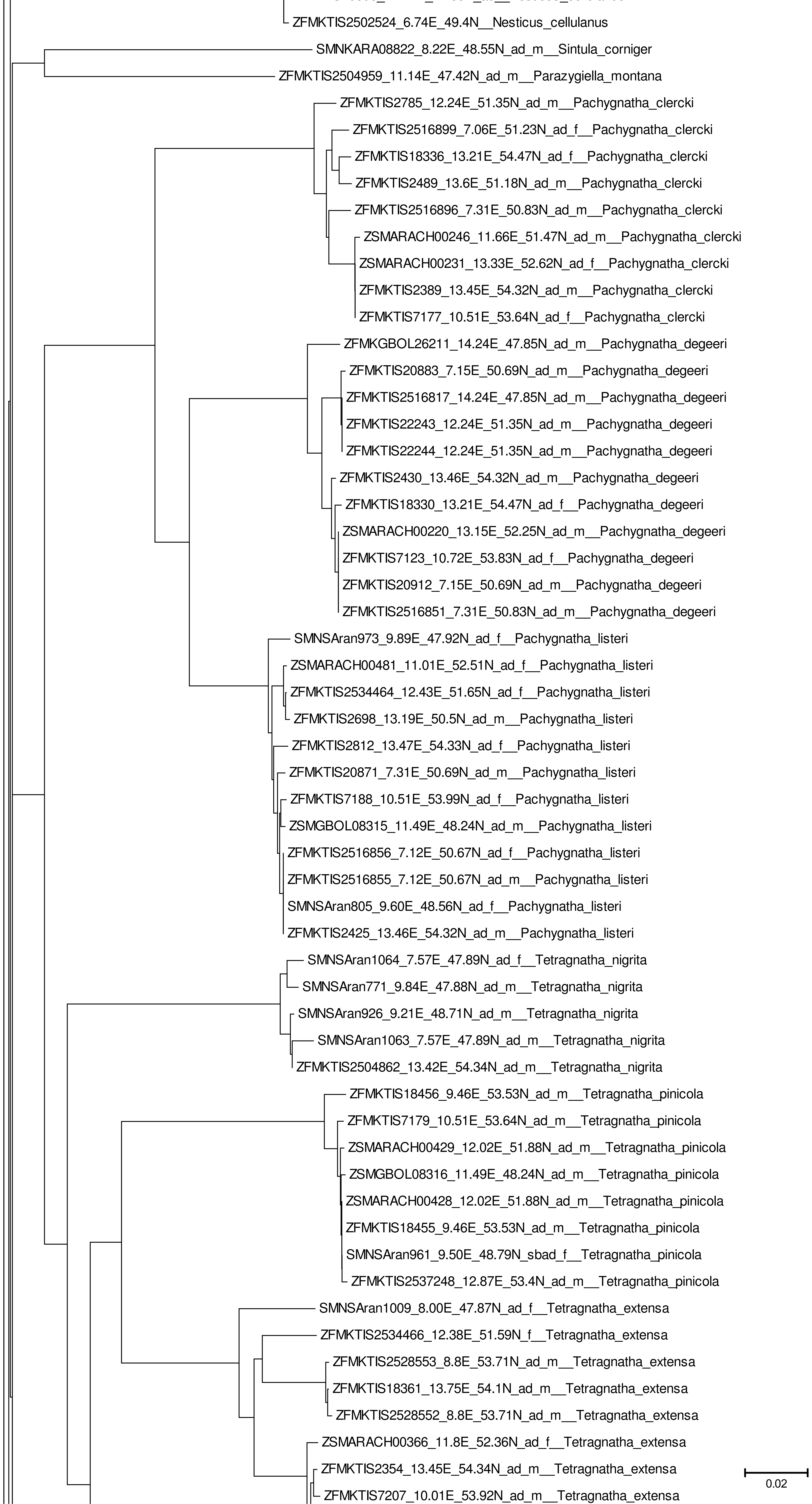
ZFMKTIS2502524_6.74E_49.4N__Nesticus_cellulanus
SMNKARA08822_8.22E_48.55N_ad_m__Sintula_corniger
ZFMKTIS2504959_11.14E_47.42N_ad_m__Parazygiella_montana
ZFMKTIS2785_12.24E_51.35N_ad_m__Pachygnatha_clercki
ZFMKTIS2516899_7.06E_51.23N_ad_f__Pachygnatha_clercki
ZFMKTIS18336_13.21E_54.47N_ad_f__Pachygnatha_clercki
ZFMKTIS2489_13.6E_51.18N_ad_m__Pachygnatha_clercki
ZFMKTIS2516896_7.31E_50.83N_ad_m__Pachygnatha_clercki
ZSMARACH00246_11.66E_51.47N_ad_m__Pachygnatha_clercki
ZSMARACH00231_13.33E_52.62N_ad_f__Pachygnatha_clercki
ZFMKTIS2389_13.45E_54.32N_ad_m__Pachygnatha_clercki
ZFMKTIS7177_10.51E_53.64N_ad_f__Pachygnatha_clercki
ZFMKGBOL26211_14.24E_47.85N_ad_m__Pachygnatha_degeeri
ZFMKTIS20883_7.15E_50.69N_ad_m__Pachygnatha_degeeri
ZFMKTIS2516817_14.24E_47.85N_ad_m__Pachygnatha_degeeri
ZFMKTIS22243_12.24E_51.35N_ad_m__Pachygnatha_degeeri
ZFMKTIS22244_12.24E_51.35N_ad_m__Pachygnatha_degeeri
ZFMKTIS2430_13.46E_54.32N_ad_m__Pachygnatha_degeeri
ZFMKTIS18330_13.21E_54.47N_ad_f__Pachygnatha_degeeri
ZSMARACH00220_13.15E_52.25N_ad_m__Pachygnatha_degeeri
ZFMKTIS7123_10.72E_53.83N_ad_f__Pachygnatha_degeeri
ZFMKTIS20912_7.15E_50.69N_ad_m__Pachygnatha_degeeri
ZFMKTIS2516851_7.31E_50.83N_ad_m__Pachygnatha_degeeri
SMNSAran973_9.89E_47.92N_ad_f__Pachygnatha_listeri
ZSMARACH00481_11.01E_52.51N_ad_f__Pachygnatha_listeri
ZFMKTIS2534464_12.43E_51.65N_ad_f__Pachygnatha_listeri
ZFMKTIS2698_13.19E_50.5N_ad_m__Pachygnatha_listeri
ZFMKTIS2812_13.47E_54.33N_ad_f__Pachygnatha_listeri
ZFMKTIS20871_7.31E_50.69N_ad_m__Pachygnatha_listeri
ZFMKTIS7188_10.51E_53.99N_ad_f__Pachygnatha_listeri
ZSMGBOL08315_11.49E_48.24N_ad_m__Pachygnatha_listeri
ZFMKTIS2516856_7.12E_50.67N_ad_f__Pachygnatha_listeri
ZFMKTIS2516855_7.12E_50.67N_ad_m__Pachygnatha_listeri
SMNSAran805_9.60E_48.56N_ad_f__Pachygnatha_listeri
ZFMKTIS2425_13.46E_54.32N_ad_m__Pachygnatha_listeri
SMNSAran1064_7.57E_47.89N_ad_f__Tetragnatha_nigrita
SMNSAran771_9.84E_47.88N_ad_m__Tetragnatha_nigrita
SMNSAran926_9.21E_48.71N_ad_m__Tetragnatha_nigrita
SMNSAran1063_7.57E_47.89N_ad_m__Tetragnatha_nigrita
ZFMKTIS2504862_13.42E_54.34N_ad_m__Tetragnatha_nigrita
ZFMKTIS18456_9.46E_53.53N_ad_m__Tetragnatha_pinicola
ZFMKTIS7179_10.51E_53.64N_ad_m__Tetragnatha_pinicola
ZSMARACH00429_12.02E_51.88N_ad_m__Tetragnatha_pinicola
ZSMGBOL08316_11.49E_48.24N_ad_m__Tetragnatha_pinicola
ZSMARACH00428_12.02E_51.88N_ad_m__Tetragnatha_pinicola
ZFMKTIS18455_9.46E_53.53N_ad_m__Tetragnatha_pinicola
SMNSAran961_9.50E_48.79N_sbad_f__Tetragnatha_pinicola
ZFMKTIS2537248_12.87E_53.4N_ad_m__Tetragnatha_pinicola
SMNSAran1009_8.00E_47.87N_ad_f__Tetragnatha_extensa
ZFMKTIS2534466_12.38E_51.59N_f__Tetragnatha_extensa
ZFMKTIS2528553_8.8E_53.71N_ad_m__Tetragnatha_extensa
ZFMKTIS18361_13.75E_54.1N_ad_m__Tetragnatha_extensa
ZFMKTIS2528552_8.8E_53.71N_ad_m__Tetragnatha_extensa
ZSMARACH00366_11.8E_52.36N_ad_f__Tetragnatha_extensa
ZFMKTIS2354_13.45E_54.34N_ad_m__Tetragnatha_extensa
ZFMKTIS7207_10.01E_53.92N_ad_m__Tetragnatha_extensa
0.02

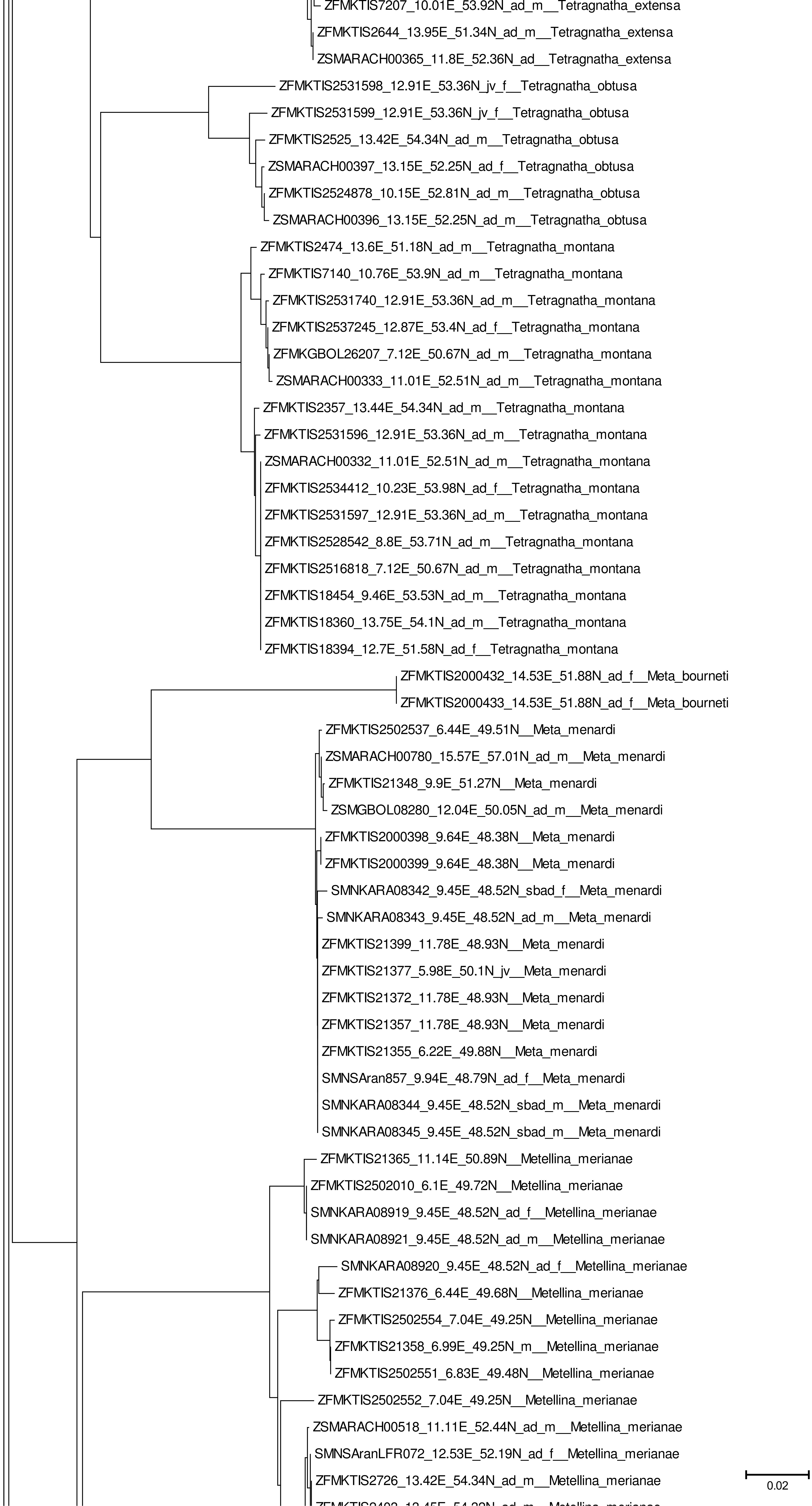
ZFMKTIS7207_10.01E_53.92N_ad_m__Tetragnatha_extensa
ZFMKTIS2644_13.95E_51.34N_ad_m__Tetragnatha_extensa
ZSMARACH00365_11.8E_52.36N_ad__Tetragnatha_extensa
ZFMKTIS2531598_12.91E_53.36N_jv_f__Tetragnatha_obtusa
ZFMKTIS2531599_12.91E_53.36N_jv_f__Tetragnatha_obtusa
ZFMKTIS2525_13.42E_54.34N_ad_m__Tetragnatha_obtusa
ZSMARACH00397_13.15E_52.25N_ad_f__Tetragnatha_obtusa
ZFMKTIS2524878_10.15E_52.81N_ad_m__Tetragnatha_obtusa
ZSMARACH00396_13.15E_52.25N_ad_m__Tetragnatha_obtusa
ZFMKTIS2474_13.6E_51.18N_ad_m__Tetragnatha_montana
ZFMKTIS7140_10.76E_53.9N_ad_m__Tetragnatha_montana
ZFMKTIS2531740_12.91E_53.36N_ad_m__Tetragnatha_montana
ZFMKTIS2537245_12.87E_53.4N_ad_f__Tetragnatha_montana
ZFMKGBOL26207_7.12E_50.67N_ad_m__Tetragnatha_montana
ZSMARACH00333_11.01E_52.51N_ad_m__Tetragnatha_montana
ZFMKTIS2357_13.44E_54.34N_ad_m__Tetragnatha_montana
ZFMKTIS2531596_12.91E_53.36N_ad_m__Tetragnatha_montana
ZSMARACH00332_11.01E_52.51N_ad_m__Tetragnatha_montana
ZFMKTIS2534412_10.23E_53.98N_ad_f__Tetragnatha_montana
ZFMKTIS2531597_12.91E_53.36N_ad_m__Tetragnatha_montana
ZFMKTIS2528542_8.8E_53.71N_ad_m__Tetragnatha_montana
ZFMKTIS2516818_7.12E_50.67N_ad_m__Tetragnatha_montana
ZFMKTIS18454_9.46E_53.53N_ad_m__Tetragnatha_montana
ZFMKTIS18360_13.75E_54.1N_ad_m__Tetragnatha_montana
ZFMKTIS18394_12.7E_51.58N_ad_f__Tetragnatha_montana
ZFMKTIS2000432_14.53E_51.88N_ad_f__Meta_bourneti
ZFMKTIS2000433_14.53E_51.88N_ad_f__Meta_bourneti
ZFMKTIS2502537_6.44E_49.51N__Meta_menardi
ZSMARACH00780_15.57E_57.01N_ad_m__Meta_menardi
ZFMKTIS21348_9.9E_51.27N__Meta_menardi
ZSMGBOL08280_12.04E_50.05N_ad_m__Meta_menardi
ZFMKTIS2000398_9.64E_48.38N__Meta_menardi
ZFMKTIS2000399_9.64E_48.38N__Meta_menardi
SMNKARA08342_9.45E_48.52N_sbad_f__Meta_menardi
SMNKARA08343_9.45E_48.52N_ad_m__Meta_menardi
ZFMKTIS21399_11.78E_48.93N__Meta_menardi
ZFMKTIS21377_5.98E_50.1N_jv__Meta_menardi
ZFMKTIS21372_11.78E_48.93N__Meta_menardi
ZFMKTIS21357_11.78E_48.93N__Meta_menardi
ZFMKTIS21355_6.22E_49.88N__Meta_menardi
SMNSAran857_9.94E_48.79N_ad_f__Meta_menardi
SMNKARA08344_9.45E_48.52N_sbad_m__Meta_menardi
SMNKARA08345_9.45E_48.52N_sbad_m__Meta_menardi
ZFMKTIS21365_11.14E_50.89N__Metellina_merianae
ZFMKTIS2502010_6.1E_49.72N__Metellina_merianae
SMNKARA08919_9.45E_48.52N_ad_f__Metellina_merianae
SMNKARA08921_9.45E_48.52N_ad_m__Metellina_merianae
SMNKARA08920_9.45E_48.52N_ad_f__Metellina_merianae
ZFMKTIS21376_6.44E_49.68N__Metellina_merianae
ZFMKTIS2502554_7.04E_49.25N__Metellina_merianae
ZFMKTIS21358_6.99E_49.25N_m__Metellina_merianae
ZFMKTIS2502551_6.83E_49.48N__Metellina_merianae
ZFMKTIS2502552_7.04E_49.25N__Metellina_merianae
ZSMARACH00518_11.11E_52.44N_ad_m__Metellina_merianae
SMNSAranLFR072_12.53E_52.19N_ad_f__Metellina_merianae
ZFMKTIS2726_13.42E_54.34N_ad_m__Metellina_merianae
0.02

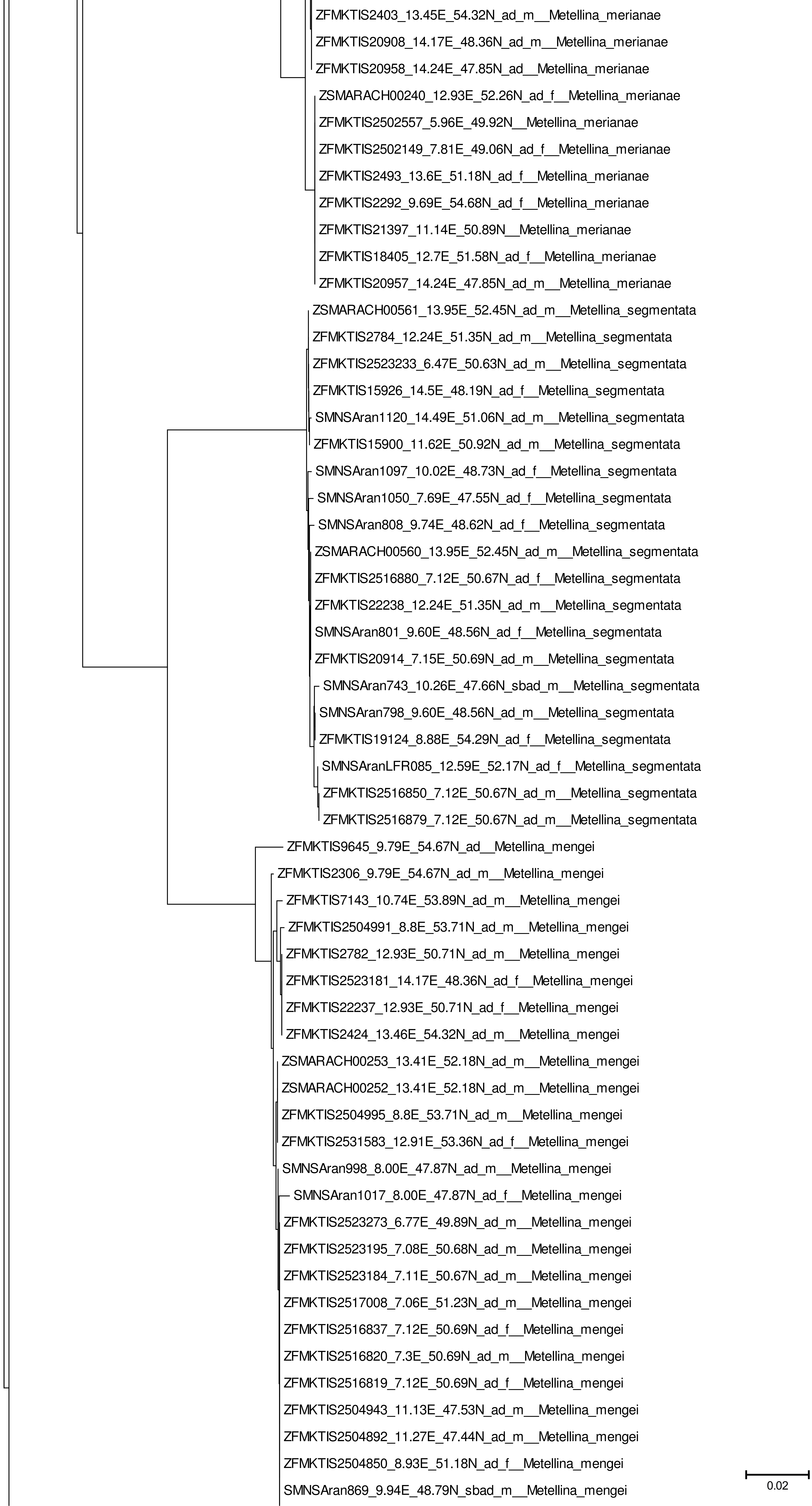
ZFMKTIS2403_13.45E_54.32N_ad_m__Metellina_merianae
ZFMKTIS20908_14.17E_48.36N_ad_m__Metellina_merianae
ZFMKTIS20958_14.24E_47.85N_ad__Metellina_merianae
ZSMARACH00240_12.93E_52.26N_ad_f__Metellina_merianae
ZFMKTIS2502557_5.96E_49.92N__Metellina_merianae
ZFMKTIS2502149_7.81E_49.06N_ad_f__Metellina_merianae
ZFMKTIS2493_13.6E_51.18N_ad_f__Metellina_merianae
ZFMKTIS2292_9.69E_54.68N_ad_f__Metellina_merianae
ZFMKTIS21397_11.14E_50.89N__Metellina_merianae
ZFMKTIS18405_12.7E_51.58N_ad_f__Metellina_merianae
ZFMKTIS20957_14.24E_47.85N_ad_m__Metellina_merianae
ZSMARACH00561_13.95E_52.45N_ad_m__Metellina_segmentata
ZFMKTIS2784_12.24E_51.35N_ad_m__Metellina_segmentata
ZFMKTIS2523233_6.47E_50.63N_ad_m__Metellina_segmentata
ZFMKTIS15926_14.5E_48.19N_ad_f__Metellina_segmentata
SMNSAran1120_14.49E_51.06N_ad_m__Metellina_segmentata
ZFMKTIS15900_11.62E_50.92N_ad_m__Metellina_segmentata
SMNSAran1097_10.02E_48.73N_ad_f__Metellina_segmentata
SMNSAran1050_7.69E_47.55N_ad_f__Metellina_segmentata
SMNSAran808_9.74E_48.62N_ad_f__Metellina_segmentata
ZSMARACH00560_13.95E_52.45N_ad_m__Metellina_segmentata
ZFMKTIS2516880_7.12E_50.67N_ad_f__Metellina_segmentata
ZFMKTIS22238_12.24E_51.35N_ad_m__Metellina_segmentata
SMNSAran801_9.60E_48.56N_ad_f__Metellina_segmentata
ZFMKTIS20914_7.15E_50.69N_ad_m__Metellina_segmentata
SMNSAran743_10.26E_47.66N_sbad_m__Metellina_segmentata
SMNSAran798_9.60E_48.56N_ad_m__Metellina_segmentata
ZFMKTIS19124_8.88E_54.29N_ad_f__Metellina_segmentata
SMNSAranLFR085_12.59E_52.17N_ad_f__Metellina_segmentata
ZFMKTIS2516850_7.12E_50.67N_ad_m__Metellina_segmentata
ZFMKTIS2516879_7.12E_50.67N_ad_m__Metellina_segmentata
ZFMKTIS9645_9.79E_54.67N_ad__Metellina_mengei
ZFMKTIS2306_9.79E_54.67N_ad_m__Metellina_mengei
ZFMKTIS7143_10.74E_53.89N_ad_m__Metellina_mengei
ZFMKTIS2504991_8.8E_53.71N_ad_m__Metellina_mengei
ZFMKTIS2782_12.93E_50.71N_ad_m__Metellina_mengei
ZFMKTIS2523181_14.17E_48.36N_ad_f__Metellina_mengei
ZFMKTIS22237_12.93E_50.71N_ad_f__Metellina_mengei
ZFMKTIS2424_13.46E_54.32N_ad_m__Metellina_mengei
ZSMARACH00253_13.41E_52.18N_ad_m__Metellina_mengei
ZSMARACH00252_13.41E_52.18N_ad_m__Metellina_mengei
ZFMKTIS2504995_8.8E_53.71N_ad_m__Metellina_mengei
ZFMKTIS2531583_12.91E_53.36N_ad_f__Metellina_mengei
SMNSAran998_8.00E_47.87N_ad_m__Metellina_mengei
SMNSAran1017_8.00E_47.87N_ad_f__Metellina_mengei
ZFMKTIS2523273_6.77E_49.89N_ad_m__Metellina_mengei
ZFMKTIS2523195_7.08E_50.68N_ad_m__Metellina_mengei
ZFMKTIS2523184_7.11E_50.67N_ad_m__Metellina_mengei
ZFMKTIS2517008_7.06E_51.23N_ad_m__Metellina_mengei
ZFMKTIS2516837_7.12E_50.69N_ad_f__Metellina_mengei
ZFMKTIS2516820_7.3E_50.69N_ad_m__Metellina_mengei
ZFMKTIS2516819_7.12E_50.69N_ad_f__Metellina_mengei
ZFMKTIS2504943_11.13E_47.53N_ad_m__Metellina_mengei
ZFMKTIS2504892_11.27E_47.44N_ad_m__Metellina_mengei
ZFMKTIS2504850_8.93E_51.18N_ad_f__Metellina_mengei
SMNSAran869_9.94E_48.79N_sbad_m__Metellina_mengei
0.02

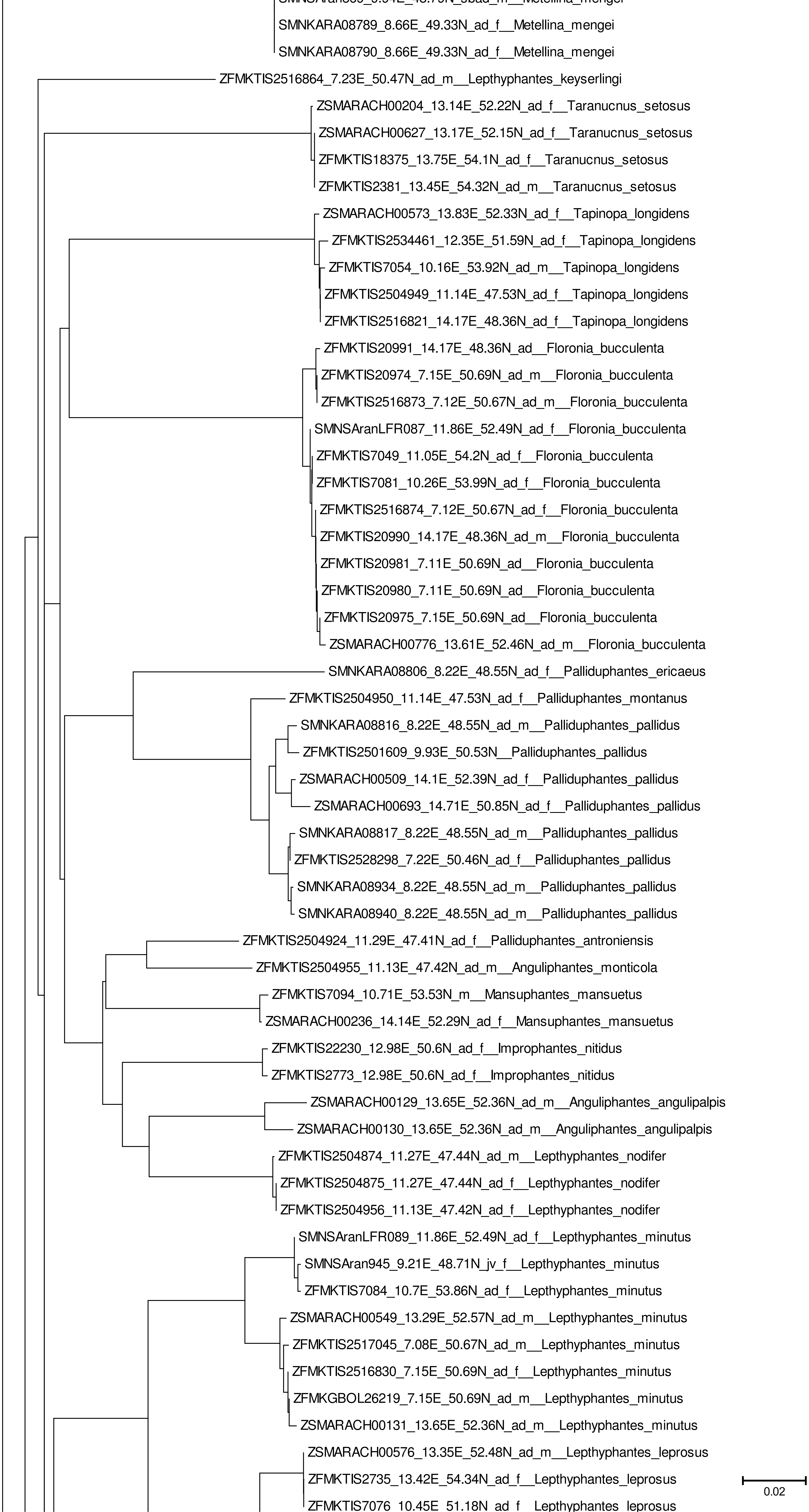
SMNKARA08789_8.66E_49.33N_ad_f__Metellina_mengei
SMNKARA08790_8.66E_49.33N_ad_f__Metellina_mengei
ZFMKTIS2516864_7.23E_50.47N_ad_m__Lepthyphantes_keyserlingi
ZSMARACH00204_13.14E_52.22N_ad_f__Taranucnus_setosus
ZSMARACH00627_13.17E_52.15N_ad_f__Taranucnus_setosus
ZFMKTIS18375_13.75E_54.1N_ad_f__Taranucnus_setosus
ZFMKTIS2381_13.45E_54.32N_ad_m__Taranucnus_setosus
ZSMARACH00573_13.83E_52.33N_ad_f__Tapinopa_longidens
ZFMKTIS2534461_12.35E_51.59N_ad_f__Tapinopa_longidens
ZFMKTIS7054_10.16E_53.92N_ad_m__Tapinopa_longidens
ZFMKTIS2504949_11.14E_47.53N_ad_f__Tapinopa_longidens
ZFMKTIS2516821_14.17E_48.36N_ad_f__Tapinopa_longidens
ZFMKTIS20991_14.17E_48.36N_ad__Floronia_bucculenta
ZFMKTIS20974_7.15E_50.69N_ad_m__Floronia_bucculenta
ZFMKTIS2516873_7.12E_50.67N_ad_m__Floronia_bucculenta
SMNSAranLFR087_11.86E_52.49N_ad_f__Floronia_bucculenta
ZFMKTIS7049_11.05E_54.2N_ad_f__Floronia_bucculenta
ZFMKTIS7081_10.26E_53.99N_ad_f__Floronia_bucculenta
ZFMKTIS2516874_7.12E_50.67N_ad_f__Floronia_bucculenta
ZFMKTIS20990_14.17E_48.36N_ad_m__Floronia_bucculenta
ZFMKTIS20981_7.11E_50.69N_ad__Floronia_bucculenta
ZFMKTIS20980_7.11E_50.69N_ad__Floronia_bucculenta
ZFMKTIS20975_7.15E_50.69N_ad__Floronia_bucculenta
ZSMARACH00776_13.61E_52.46N_ad_m__Floronia_bucculenta
SMNKARA08806_8.22E_48.55N_ad_f__Palliduphantes_ericaeus
ZFMKTIS2504950_11.14E_47.53N_ad_f__Palliduphantes_montanus
SMNKARA08816_8.22E_48.55N_ad_m__Palliduphantes_pallidus
ZFMKTIS2501609_9.93E_50.53N__Palliduphantes_pallidus
ZSMARACH00509_14.1E_52.39N_ad_f__Palliduphantes_pallidus
ZSMARACH00693_14.71E_50.85N_ad_f__Palliduphantes_pallidus
SMNKARA08817_8.22E_48.55N_ad_m__Palliduphantes_pallidus
ZFMKTIS2528298_7.22E_50.46N_ad_f__Palliduphantes_pallidus
SMNKARA08934_8.22E_48.55N_ad_m__Palliduphantes_pallidus
SMNKARA08940_8.22E_48.55N_ad_m__Palliduphantes_pallidus
ZFMKTIS2504924_11.29E_47.41N_ad_f__Palliduphantes_antroniensis
ZFMKTIS2504955_11.13E_47.42N_ad_m__Anguliphantes_monticola
ZFMKTIS7094_10.71E_53.53N_m__Mansuphantes_mansuetus
ZSMARACH00236_14.14E_52.29N_ad_f__Mansuphantes_mansuetus
ZFMKTIS22230_12.98E_50.6N_ad_f__Improphantes_nitidus
ZFMKTIS2773_12.98E_50.6N_ad_f__Improphantes_nitidus
ZSMARACH00129_13.65E_52.36N_ad_m__Anguliphantes_angulipalpis
ZSMARACH00130_13.65E_52.36N_ad_m__Anguliphantes_angulipalpis
ZFMKTIS2504874_11.27E_47.44N_ad_m__Lepthyphantes_nodifer
ZFMKTIS2504875_11.27E_47.44N_ad_f__Lepthyphantes_nodifer
ZFMKTIS2504956_11.13E_47.42N_ad_f__Lepthyphantes_nodifer
SMNSAranLFR089_11.86E_52.49N_ad_f__Lepthyphantes_minutus
SMNSAran945_9.21E_48.71N_jv_f__Lepthyphantes_minutus
ZFMKTIS7084_10.7E_53.86N_ad_f__Lepthyphantes_minutus
ZSMARACH00549_13.29E_52.57N_ad_m__Lepthyphantes_minutus
ZFMKTIS2517045_7.08E_50.67N_ad_m__Lepthyphantes_minutus
ZFMKTIS2516830_7.15E_50.69N_ad_f__Lepthyphantes_minutus
ZFMKGBOL26219_7.15E_50.69N_ad_m__Lepthyphantes_minutus
ZSMARACH00131_13.65E_52.36N_ad_m__Lepthyphantes_minutus
ZSMARACH00576_13.35E_52.48N_ad_m__Lepthyphantes_leprosus
ZFMKTIS2735_13.42E_54.34N_ad_f__Lepthyphantes_leprosus
ZFMKTIS7076_10.45E_51.18N_ad_f__Lepthyphantes_leprosus
0.02

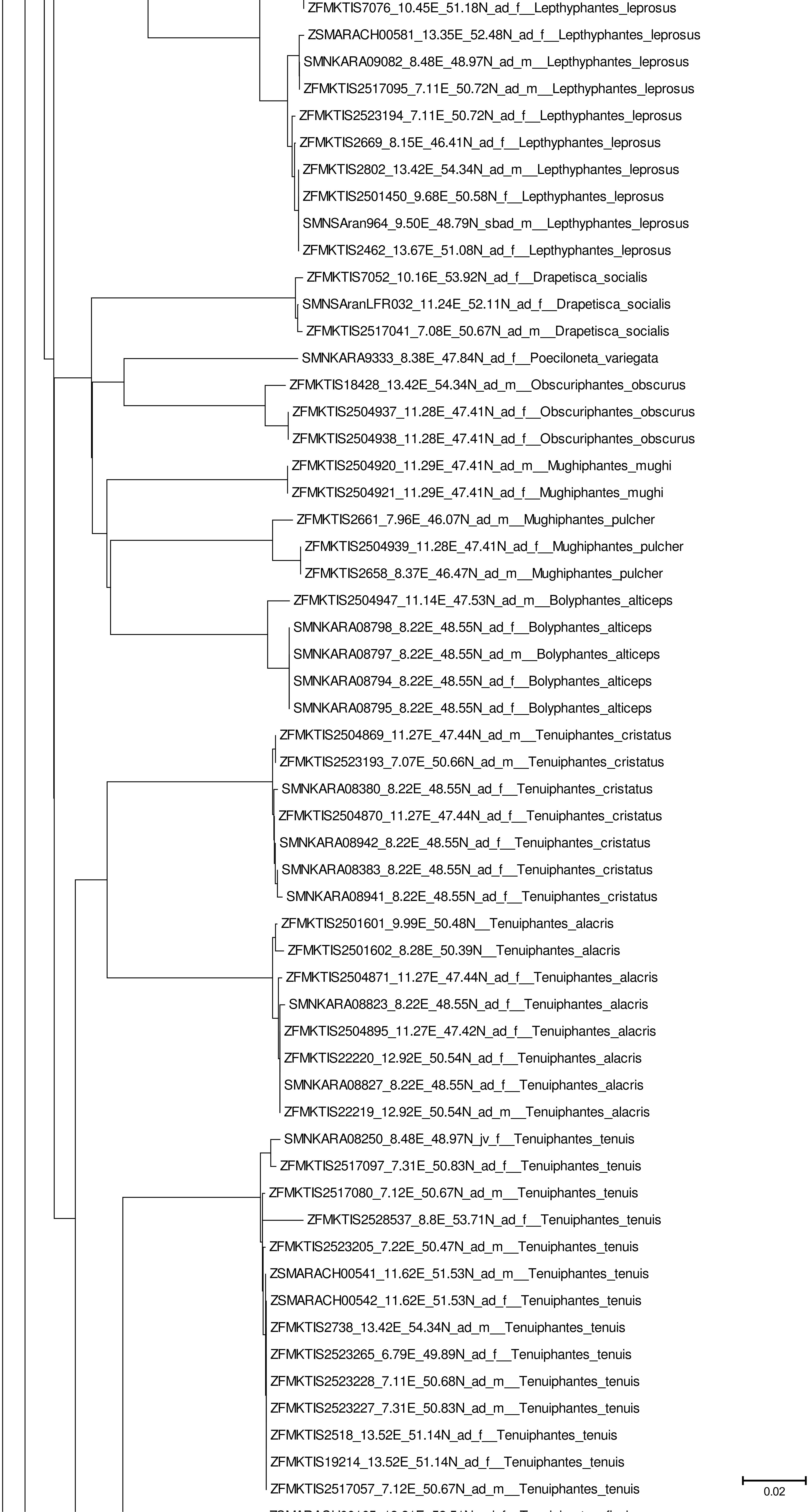
ZFMKTIS7076_10.45E_51.18N_ad_f__Lepthyphantes_leprosus
ZSMARACH00581_13.35E_52.48N_ad_f__Lepthyphantes_leprosus
SMNKARA09082_8.48E_48.97N_ad_m__Lepthyphantes_leprosus
ZFMKTIS2517095_7.11E_50.72N_ad_m__Lepthyphantes_leprosus
ZFMKTIS2523194_7.11E_50.72N_ad_f__Lepthyphantes_leprosus
ZFMKTIS2669_8.15E_46.41N_ad_f__Lepthyphantes_leprosus
ZFMKTIS2802_13.42E_54.34N_ad_m__Lepthyphantes_leprosus
ZFMKTIS2501450_9.68E_50.58N_f__Lepthyphantes_leprosus
SMNSAran964_9.50E_48.79N_sbad_m__Lepthyphantes_leprosus
ZFMKTIS2462_13.67E_51.08N_ad_f__Lepthyphantes_leprosus
ZFMKTIS7052_10.16E_53.92N_ad_f__Drapetisca_socialis
SMNSAranLFR032_11.24E_52.11N_ad_f__Drapetisca_socialis
ZFMKTIS2517041_7.08E_50.67N_ad_m__Drapetisca_socialis
SMNKARA9333_8.38E_47.84N_ad_f__Poeciloneta_variegata
ZFMKTIS18428_13.42E_54.34N_ad_m__Obscuriphantes_obscurus
ZFMKTIS2504937_11.28E_47.41N_ad_f__Obscuriphantes_obscurus
ZFMKTIS2504938_11.28E_47.41N_ad_f__Obscuriphantes_obscurus
ZFMKTIS2504920_11.29E_47.41N_ad_m__Mughiphantes_mughi
ZFMKTIS2504921_11.29E_47.41N_ad_f__Mughiphantes_mughi
ZFMKTIS2661_7.96E_46.07N_ad_m__Mughiphantes_pulcher
ZFMKTIS2504939_11.28E_47.41N_ad_f__Mughiphantes_pulcher
ZFMKTIS2658_8.37E_46.47N_ad_m__Mughiphantes_pulcher
ZFMKTIS2504947_11.14E_47.53N_ad_m__Bolyphantes_alticeps
SMNKARA08798_8.22E_48.55N_ad_f__Bolyphantes_alticeps
SMNKARA08797_8.22E_48.55N_ad_m__Bolyphantes_alticeps
SMNKARA08794_8.22E_48.55N_ad_f__Bolyphantes_alticeps
SMNKARA08795_8.22E_48.55N_ad_f__Bolyphantes_alticeps
ZFMKTIS2504869_11.27E_47.44N_ad_m__Tenuiphantes_cristatus
ZFMKTIS2523193_7.07E_50.66N_ad_m__Tenuiphantes_cristatus
SMNKARA08380_8.22E_48.55N_ad_f__Tenuiphantes_cristatus
ZFMKTIS2504870_11.27E_47.44N_ad_f__Tenuiphantes_cristatus
SMNKARA08942_8.22E_48.55N_ad_f__Tenuiphantes_cristatus
SMNKARA08383_8.22E_48.55N_ad_f__Tenuiphantes_cristatus
SMNKARA08941_8.22E_48.55N_ad_f__Tenuiphantes_cristatus
ZFMKTIS2501601_9.99E_50.48N__Tenuiphantes_alacris
ZFMKTIS2501602_8.28E_50.39N__Tenuiphantes_alacris
ZFMKTIS2504871_11.27E_47.44N_ad_f__Tenuiphantes_alacris
SMNKARA08823_8.22E_48.55N_ad_f__Tenuiphantes_alacris
ZFMKTIS2504895_11.27E_47.42N_ad_f__Tenuiphantes_alacris
ZFMKTIS22220_12.92E_50.54N_ad_f__Tenuiphantes_alacris
SMNKARA08827_8.22E_48.55N_ad_f__Tenuiphantes_alacris
ZFMKTIS22219_12.92E_50.54N_ad_m__Tenuiphantes_alacris
SMNKARA08250_8.48E_48.97N_jv_f__Tenuiphantes_tenuis
ZFMKTIS2517097_7.31E_50.83N_ad_f__Tenuiphantes_tenuis
ZFMKTIS2517080_7.12E_50.67N_ad_m__Tenuiphantes_tenuis
ZFMKTIS2528537_8.8E_53.71N_ad_f__Tenuiphantes_tenuis
ZFMKTIS2523205_7.22E_50.47N_ad_m__Tenuiphantes_tenuis
ZSMARACH00541_11.62E_51.53N_ad_m__Tenuiphantes_tenuis
ZSMARACH00542_11.62E_51.53N_ad_f__Tenuiphantes_tenuis
ZFMKTIS2738_13.42E_54.34N_ad_m__Tenuiphantes_tenuis
ZFMKTIS2523265_6.79E_49.89N_ad_f__Tenuiphantes_tenuis
ZFMKTIS2523228_7.11E_50.68N_ad_m__Tenuiphantes_tenuis
ZFMKTIS2523227_7.31E_50.83N_ad_m__Tenuiphantes_tenuis
ZFMKTIS2518_13.52E_51.14N_ad_f__Tenuiphantes_tenuis
ZFMKTIS19214_13.52E_51.14N_ad_f__Tenuiphantes_tenuis
ZFMKTIS2517057_7.12E_50.67N_ad_m__Tenuiphantes_tenuis
0.02

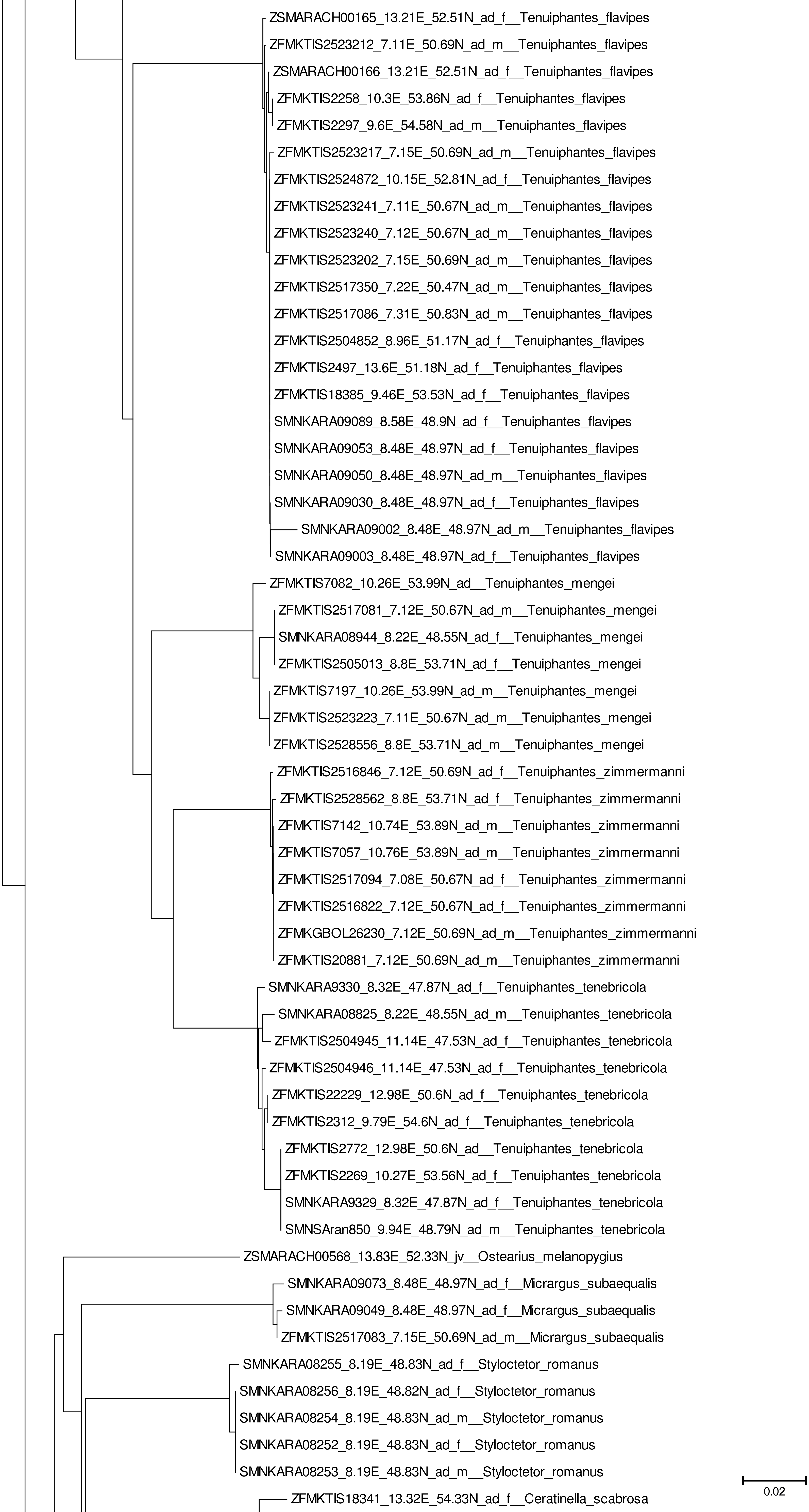
ZSMARACH00165_13.21E_52.51N_ad_f__Tenuiphantes_flavipes
ZFMKTIS2523212_7.11E_50.69N_ad_m__Tenuiphantes_flavipes
ZSMARACH00166_13.21E_52.51N_ad_f__Tenuiphantes_flavipes
ZFMKTIS2258_10.3E_53.86N_ad_f__Tenuiphantes_flavipes
ZFMKTIS2297_9.6E_54.58N_ad_m__Tenuiphantes_flavipes
ZFMKTIS2523217_7.15E_50.69N_ad_m__Tenuiphantes_flavipes
ZFMKTIS2524872_10.15E_52.81N_ad_f__Tenuiphantes_flavipes
ZFMKTIS2523241_7.11E_50.67N_ad_m__Tenuiphantes_flavipes
ZFMKTIS2523240_7.12E_50.67N_ad_m__Tenuiphantes_flavipes
ZFMKTIS2523202_7.15E_50.69N_ad_m__Tenuiphantes_flavipes
ZFMKTIS2517350_7.22E_50.47N_ad_m__Tenuiphantes_flavipes
ZFMKTIS2517086_7.31E_50.83N_ad_m__Tenuiphantes_flavipes
ZFMKTIS2504852_8.96E_51.17N_ad_f__Tenuiphantes_flavipes
ZFMKTIS2497_13.6E_51.18N_ad_f__Tenuiphantes_flavipes
ZFMKTIS18385_9.46E_53.53N_ad_f__Tenuiphantes_flavipes
SMNKARA09089_8.58E_48.9N_ad_f__Tenuiphantes_flavipes
SMNKARA09053_8.48E_48.97N_ad_f__Tenuiphantes_flavipes
SMNKARA09050_8.48E_48.97N_ad_m__Tenuiphantes_flavipes
SMNKARA09030_8.48E_48.97N_ad_f__Tenuiphantes_flavipes
SMNKARA09002_8.48E_48.97N_ad_m__Tenuiphantes_flavipes
SMNKARA09003_8.48E_48.97N_ad_f__Tenuiphantes_flavipes
ZFMKTIS7082_10.26E_53.99N_ad__Tenuiphantes_mengei
ZFMKTIS2517081_7.12E_50.67N_ad_m__Tenuiphantes_mengei
SMNKARA08944_8.22E_48.55N_ad_f__Tenuiphantes_mengei
ZFMKTIS2505013_8.8E_53.71N_ad_f__Tenuiphantes_mengei
ZFMKTIS7197_10.26E_53.99N_ad_m__Tenuiphantes_mengei
ZFMKTIS2523223_7.11E_50.67N_ad_m__Tenuiphantes_mengei
ZFMKTIS2528556_8.8E_53.71N_ad_m__Tenuiphantes_mengei
ZFMKTIS2516846_7.12E_50.69N_ad_f__Tenuiphantes_zimmermanni
ZFMKTIS2528562_8.8E_53.71N_ad_f__Tenuiphantes_zimmermanni
ZFMKTIS7142_10.74E_53.89N_ad_m__Tenuiphantes_zimmermanni
ZFMKTIS7057_10.76E_53.89N_ad_m__Tenuiphantes_zimmermanni
ZFMKTIS2517094_7.08E_50.67N_ad_f__Tenuiphantes_zimmermanni
ZFMKTIS2516822_7.12E_50.67N_ad_f__Tenuiphantes_zimmermanni
ZFMKGBOL26230_7.12E_50.69N_ad_m__Tenuiphantes_zimmermanni
ZFMKTIS20881_7.12E_50.69N_ad_m__Tenuiphantes_zimmermanni
SMNKARA9330_8.32E_47.87N_ad_f__Tenuiphantes_tenebricola
SMNKARA08825_8.22E_48.55N_ad_m__Tenuiphantes_tenebricola
ZFMKTIS2504945_11.14E_47.53N_ad_f__Tenuiphantes_tenebricola
ZFMKTIS2504946_11.14E_47.53N_ad_f__Tenuiphantes_tenebricola
ZFMKTIS22229_12.98E_50.6N_ad_f__Tenuiphantes_tenebricola
ZFMKTIS2312_9.79E_54.6N_ad_f__Tenuiphantes_tenebricola
ZFMKTIS2772_12.98E_50.6N_ad__Tenuiphantes_tenebricola
ZFMKTIS2269_10.27E_53.56N_ad_f__Tenuiphantes_tenebricola
SMNKARA9329_8.32E_47.87N_ad_f__Tenuiphantes_tenebricola
SMNSAran850_9.94E_48.79N_ad_m__Tenuiphantes_tenebricola
ZSMARACH00568_13.83E_52.33N_jv__Ostearius_melanopygius
SMNKARA09073_8.48E_48.97N_ad_f__Micrargus_subaequalis
SMNKARA09049_8.48E_48.97N_ad_f__Micrargus_subaequalis
ZFMKTIS2517083_7.15E_50.69N_ad_m__Micrargus_subaequalis
SMNKARA08255_8.19E_48.83N_ad_f__Styloctetor_romanus
SMNKARA08256_8.19E_48.82N_ad_f__Styloctetor_romanus
SMNKARA08254_8.19E_48.83N_ad_m__Styloctetor_romanus
SMNKARA08252_8.19E_48.83N_ad_f__Styloctetor_romanus
SMNKARA08253_8.19E_48.83N_ad_m__Styloctetor_romanus
0.02
ZFMKTIS18341_13.32E_54.33N_ad_f__Ceratinella_scabrosa

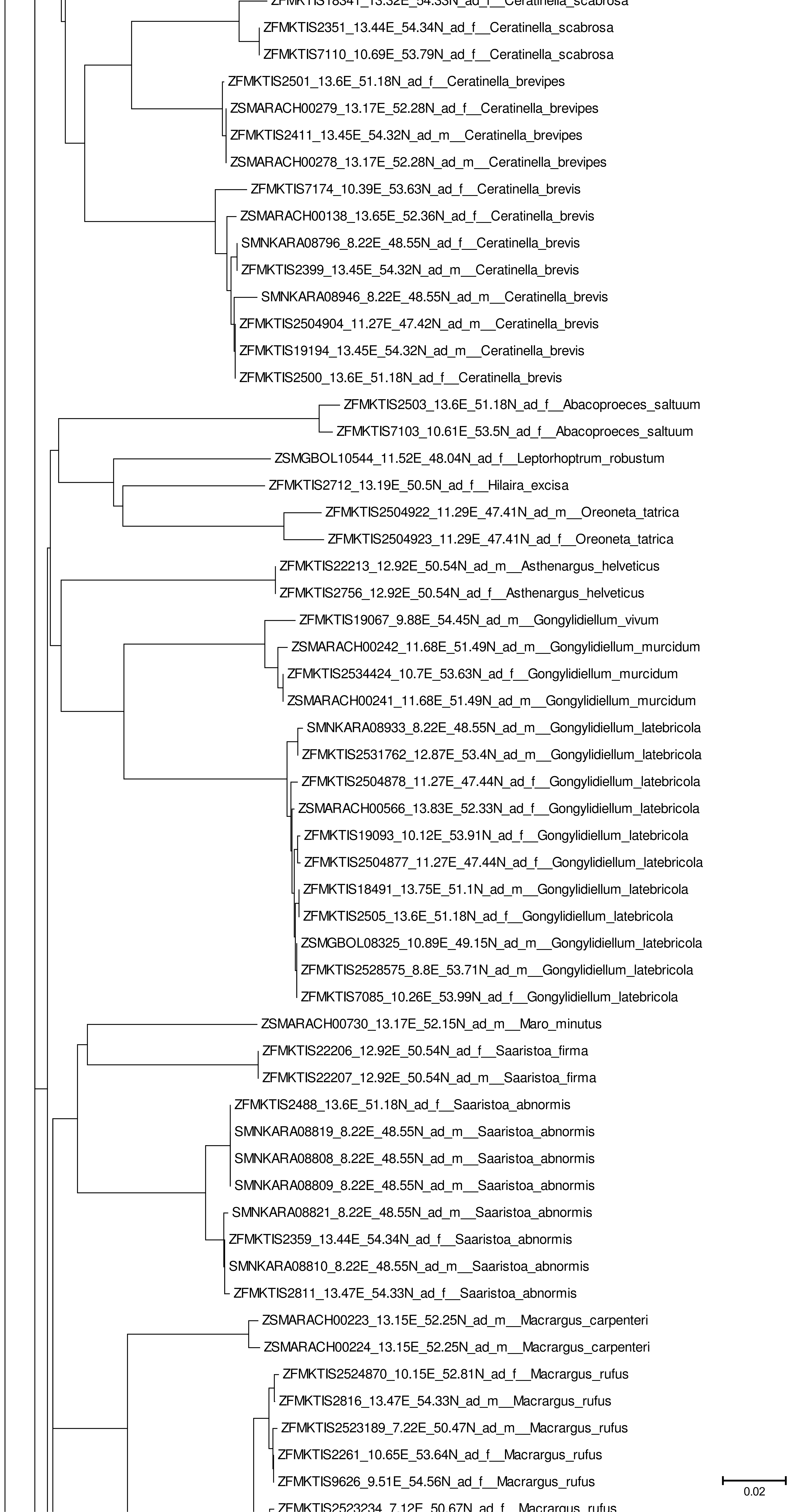
ZFMKTIS18341_13.32E_54.33N_ad_f__Ceratinella_scabrosa
ZFMKTIS2351_13.44E_54.34N_ad_f__Ceratinella_scabrosa
ZFMKTIS7110_10.69E_53.79N_ad_f__Ceratinella_scabrosa
ZFMKTIS2501_13.6E_51.18N_ad_f__Ceratinella_brevipes
ZSMARACH00279_13.17E_52.28N_ad_f__Ceratinella_brevipes
ZFMKTIS2411_13.45E_54.32N_ad_m__Ceratinella_brevipes
ZSMARACH00278_13.17E_52.28N_ad_m__Ceratinella_brevipes
ZFMKTIS7174_10.39E_53.63N_ad_f__Ceratinella_brevis
ZSMARACH00138_13.65E_52.36N_ad_f__Ceratinella_brevis
SMNKARA08796_8.22E_48.55N_ad_f__Ceratinella_brevis
ZFMKTIS2399_13.45E_54.32N_ad_m__Ceratinella_brevis
SMNKARA08946_8.22E_48.55N_ad_m__Ceratinella_brevis
ZFMKTIS2504904_11.27E_47.42N_ad_m__Ceratinella_brevis
ZFMKTIS19194_13.45E_54.32N_ad_m__Ceratinella_brevis
ZFMKTIS2500_13.6E_51.18N_ad_f__Ceratinella_brevis
ZFMKTIS2503_13.6E_51.18N_ad_f__Abacoproeces_saltuum
ZFMKTIS7103_10.61E_53.5N_ad_f__Abacoproeces_saltuum
ZSMGBOL10544_11.52E_48.04N_ad_f__Leptorhoptrum_robustum
ZFMKTIS2712_13.19E_50.5N_ad_f__Hilaira_excisa
ZFMKTIS2504922_11.29E_47.41N_ad_m__Oreoneta_tatrica
ZFMKTIS2504923_11.29E_47.41N_ad_f__Oreoneta_tatrica
ZFMKTIS22213_12.92E_50.54N_ad_m__Asthenargus_helveticus
ZFMKTIS2756_12.92E_50.54N_ad_f__Asthenargus_helveticus
ZFMKTIS19067_9.88E_54.45N_ad_m__Gongylidiellum_vivum
ZSMARACH00242_11.68E_51.49N_ad_m__Gongylidiellum_murcidum
ZFMKTIS2534424_10.7E_53.63N_ad_f__Gongylidiellum_murcidum
ZSMARACH00241_11.68E_51.49N_ad_m__Gongylidiellum_murcidum
SMNKARA08933_8.22E_48.55N_ad_m__Gongylidiellum_latebricola
ZFMKTIS2531762_12.87E_53.4N_ad_m__Gongylidiellum_latebricola
ZFMKTIS2504878_11.27E_47.44N_ad_f__Gongylidiellum_latebricola
ZSMARACH00566_13.83E_52.33N_ad_f__Gongylidiellum_latebricola
ZFMKTIS19093_10.12E_53.91N_ad_f__Gongylidiellum_latebricola
ZFMKTIS2504877_11.27E_47.44N_ad_f__Gongylidiellum_latebricola
ZFMKTIS18491_13.75E_51.1N_ad_m__Gongylidiellum_latebricola
ZFMKTIS2505_13.6E_51.18N_ad_f__Gongylidiellum_latebricola
ZSMGBOL08325_10.89E_49.15N_ad_m__Gongylidiellum_latebricola
ZFMKTIS2528575_8.8E_53.71N_ad_m__Gongylidiellum_latebricola
ZFMKTIS7085_10.26E_53.99N_ad_f__Gongylidiellum_latebricola
ZSMARACH00730_13.17E_52.15N_ad_m__Maro_minutus
ZFMKTIS22206_12.92E_50.54N_ad_f__Saaristoa_firma
ZFMKTIS22207_12.92E_50.54N_ad_m__Saaristoa_firma
ZFMKTIS2488_13.6E_51.18N_ad_f__Saaristoa_abnormis
SMNKARA08819_8.22E_48.55N_ad_m__Saaristoa_abnormis
SMNKARA08808_8.22E_48.55N_ad_m__Saaristoa_abnormis
SMNKARA08809_8.22E_48.55N_ad_m__Saaristoa_abnormis
SMNKARA08821_8.22E_48.55N_ad_m__Saaristoa_abnormis
ZFMKTIS2359_13.44E_54.34N_ad_f__Saaristoa_abnormis
SMNKARA08810_8.22E_48.55N_ad_m__Saaristoa_abnormis
ZFMKTIS2811_13.47E_54.33N_ad_f__Saaristoa_abnormis
ZSMARACH00223_13.15E_52.25N_ad_m__Macrargus_carpenteri
ZSMARACH00224_13.15E_52.25N_ad_m__Macrargus_carpenteri
ZFMKTIS2524870_10.15E_52.81N_ad_f__Macrargus_rufus
ZFMKTIS2816_13.47E_54.33N_ad_m__Macrargus_rufus
ZFMKTIS2523189_7.22E_50.47N_ad_m__Macrargus_rufus
ZFMKTIS2261_10.65E_53.64N_ad_f__Macrargus_rufus
ZFMKTIS9626_9.51E_54.56N_ad_f__Macrargus_rufus
ZFMKTIS2523234_7.12E_50.67N_ad_f__Macrargus_rufus
0.02

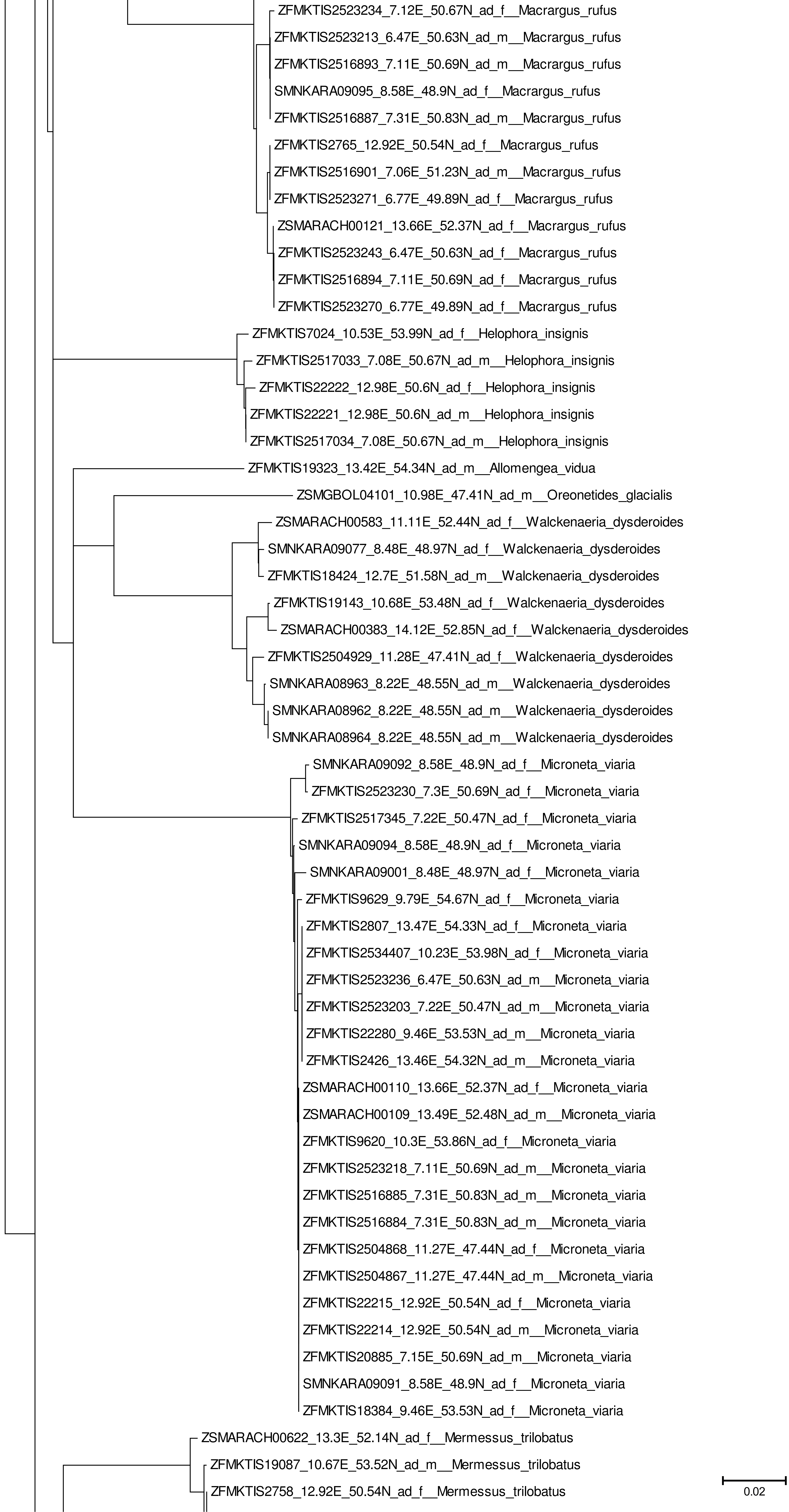
ZFMKTIS2523234_7.12E_50.67N_ad_f__Macrargus_rufus
ZFMKTIS2523213_6.47E_50.63N_ad_m__Macrargus_rufus
ZFMKTIS2516893_7.11E_50.69N_ad_m__Macrargus_rufus
SMNKARA09095_8.58E_48.9N_ad_f__Macrargus_rufus
ZFMKTIS2516887_7.31E_50.83N_ad_m__Macrargus_rufus
ZFMKTIS2765_12.92E_50.54N_ad_f__Macrargus_rufus
ZFMKTIS2516901_7.06E_51.23N_ad_m__Macrargus_rufus
ZFMKTIS2523271_6.77E_49.89N_ad_f__Macrargus_rufus
ZSMARACH00121_13.66E_52.37N_ad_f__Macrargus_rufus
ZFMKTIS2523243_6.47E_50.63N_ad_f__Macrargus_rufus
ZFMKTIS2516894_7.11E_50.69N_ad_f__Macrargus_rufus
ZFMKTIS2523270_6.77E_49.89N_ad_f__Macrargus_rufus
ZFMKTIS7024_10.53E_53.99N_ad_f__Helophora_insignis
ZFMKTIS2517033_7.08E_50.67N_ad_m__Helophora_insignis
ZFMKTIS22222_12.98E_50.6N_ad_f__Helophora_insignis
ZFMKTIS22221_12.98E_50.6N_ad_m__Helophora_insignis
ZFMKTIS2517034_7.08E_50.67N_ad_m__Helophora_insignis
ZFMKTIS19323_13.42E_54.34N_ad_m__Allomengea_vidua
ZSMGBOL04101_10.98E_47.41N_ad_m__Oreonetides_glacialis
ZSMARACH00583_11.11E_52.44N_ad_f__Walckenaeria_dysderoides
SMNKARA09077_8.48E_48.97N_ad_f__Walckenaeria_dysderoides
ZFMKTIS18424_12.7E_51.58N_ad_m__Walckenaeria_dysderoides
ZFMKTIS19143_10.68E_53.48N_ad_f__Walckenaeria_dysderoides
ZSMARACH00383_14.12E_52.85N_ad_f__Walckenaeria_dysderoides
ZFMKTIS2504929_11.28E_47.41N_ad_f__Walckenaeria_dysderoides
SMNKARA08963_8.22E_48.55N_ad_m__Walckenaeria_dysderoides
SMNKARA08962_8.22E_48.55N_ad_m__Walckenaeria_dysderoides
SMNKARA08964_8.22E_48.55N_ad_m__Walckenaeria_dysderoides
SMNKARA09092_8.58E_48.9N_ad_f__Microneta_viaria
ZFMKTIS2523230_7.3E_50.69N_ad_f__Microneta_viaria
ZFMKTIS2517345_7.22E_50.47N_ad_f__Microneta_viaria
SMNKARA09094_8.58E_48.9N_ad_f__Microneta_viaria
SMNKARA09001_8.48E_48.97N_ad_f__Microneta_viaria
ZFMKTIS9629_9.79E_54.67N_ad_f__Microneta_viaria
ZFMKTIS2807_13.47E_54.33N_ad_f__Microneta_viaria
ZFMKTIS2534407_10.23E_53.98N_ad_f__Microneta_viaria
ZFMKTIS2523236_6.47E_50.63N_ad_m__Microneta_viaria
ZFMKTIS2523203_7.22E_50.47N_ad_m__Microneta_viaria
ZFMKTIS22280_9.46E_53.53N_ad_m__Microneta_viaria
ZFMKTIS2426_13.46E_54.32N_ad_m__Microneta_viaria
ZSMARACH00110_13.66E_52.37N_ad_f__Microneta_viaria
ZSMARACH00109_13.49E_52.48N_ad_m__Microneta_viaria
ZFMKTIS9620_10.3E_53.86N_ad_f__Microneta_viaria
ZFMKTIS2523218_7.11E_50.69N_ad_m__Microneta_viaria
ZFMKTIS2516885_7.31E_50.83N_ad_m__Microneta_viaria
ZFMKTIS2516884_7.31E_50.83N_ad_m__Microneta_viaria
ZFMKTIS2504868_11.27E_47.44N_ad_f__Microneta_viaria
ZFMKTIS2504867_11.27E_47.44N_ad_m__Microneta_viaria
ZFMKTIS22215_12.92E_50.54N_ad_f__Microneta_viaria
ZFMKTIS22214_12.92E_50.54N_ad_m__Microneta_viaria
ZFMKTIS20885_7.15E_50.69N_ad_m__Microneta_viaria
SMNKARA09091_8.58E_48.9N_ad_f__Microneta_viaria
ZFMKTIS18384_9.46E_53.53N_ad_f__Microneta_viaria
ZSMARACH00622_13.3E_52.14N_ad_f__Mermessus_trilobatus
ZFMKTIS19087_10.67E_53.52N_ad_m__Mermessus_trilobatus
ZFMKTIS2758_12.92E_50.54N_ad_f__Mermessus_trilobatus
0.02

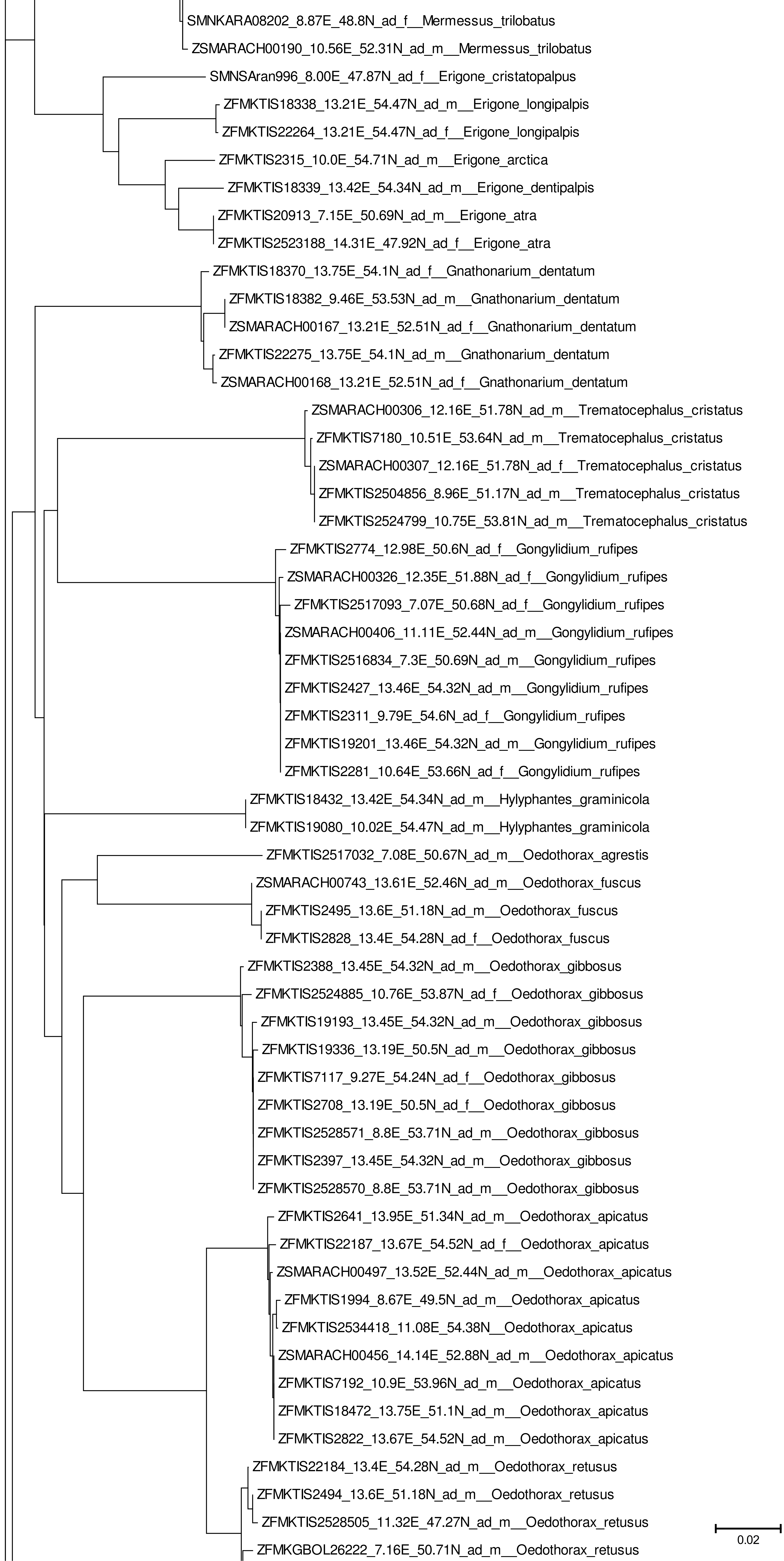
SMNKARA08202_8.87E_48.8N_ad_f__Mermessus_trilobatus
ZSMARACH00190_10.56E_52.31N_ad_m__Mermessus_trilobatus
SMNSAran996_8.00E_47.87N_ad_f__Erigone_cristatopalpus
ZFMKTIS18338_13.21E_54.47N_ad_m__Erigone_longipalpis
ZFMKTIS22264_13.21E_54.47N_ad_f__Erigone_longipalpis
ZFMKTIS2315_10.0E_54.71N_ad_m__Erigone_arctica
ZFMKTIS18339_13.42E_54.34N_ad_m__Erigone_dentipalpis
ZFMKTIS20913_7.15E_50.69N_ad_m__Erigone_atra
ZFMKTIS2523188_14.31E_47.92N_ad_f__Erigone_atra
ZFMKTIS18370_13.75E_54.1N_ad_f__Gnathonarium_dentatum
ZFMKTIS18382_9.46E_53.53N_ad_m__Gnathonarium_dentatum
ZSMARACH00167_13.21E_52.51N_ad_f__Gnathonarium_dentatum
ZFMKTIS22275_13.75E_54.1N_ad_m__Gnathonarium_dentatum
ZSMARACH00168_13.21E_52.51N_ad_f__Gnathonarium_dentatum
ZSMARACH00306_12.16E_51.78N_ad_m__Trematocephalus_cristatus
ZFMKTIS7180_10.51E_53.64N_ad_m__Trematocephalus_cristatus
ZSMARACH00307_12.16E_51.78N_ad_f__Trematocephalus_cristatus
ZFMKTIS2504856_8.96E_51.17N_ad_m__Trematocephalus_cristatus
ZFMKTIS2524799_10.75E_53.81N_ad_m__Trematocephalus_cristatus
ZFMKTIS2774_12.98E_50.6N_ad_f__Gongylidium_rufipes
ZSMARACH00326_12.35E_51.88N_ad_f__Gongylidium_rufipes
ZFMKTIS2517093_7.07E_50.68N_ad_f__Gongylidium_rufipes
ZSMARACH00406_11.11E_52.44N_ad_m__Gongylidium_rufipes
ZFMKTIS2516834_7.3E_50.69N_ad_m__Gongylidium_rufipes
ZFMKTIS2427_13.46E_54.32N_ad_m__Gongylidium_rufipes
ZFMKTIS2311_9.79E_54.6N_ad_f__Gongylidium_rufipes
ZFMKTIS19201_13.46E_54.32N_ad_m__Gongylidium_rufipes
ZFMKTIS2281_10.64E_53.66N_ad_f__Gongylidium_rufipes
ZFMKTIS18432_13.42E_54.34N_ad_m__Hylyphantes_graminicola
ZFMKTIS19080_10.02E_54.47N_ad_m__Hylyphantes_graminicola
ZFMKTIS2517032_7.08E_50.67N_ad_m__Oedothorax_agrestis
ZSMARACH00743_13.61E_52.46N_ad_m__Oedothorax_fuscus
ZFMKTIS2495_13.6E_51.18N_ad_m__Oedothorax_fuscus
ZFMKTIS2828_13.4E_54.28N_ad_f__Oedothorax_fuscus
ZFMKTIS2388_13.45E_54.32N_ad_m__Oedothorax_gibbosus
ZFMKTIS2524885_10.76E_53.87N_ad_f__Oedothorax_gibbosus
ZFMKTIS19193_13.45E_54.32N_ad_m__Oedothorax_gibbosus
ZFMKTIS19336_13.19E_50.5N_ad_m__Oedothorax_gibbosus
ZFMKTIS7117_9.27E_54.24N_ad_f__Oedothorax_gibbosus
ZFMKTIS2708_13.19E_50.5N_ad_f__Oedothorax_gibbosus
ZFMKTIS2528571_8.8E_53.71N_ad_m__Oedothorax_gibbosus
ZFMKTIS2397_13.45E_54.32N_ad_m__Oedothorax_gibbosus
ZFMKTIS2528570_8.8E_53.71N_ad_m__Oedothorax_gibbosus
ZFMKTIS2641_13.95E_51.34N_ad_m__Oedothorax_apicatus
ZFMKTIS22187_13.67E_54.52N_ad_f__Oedothorax_apicatus
ZSMARACH00497_13.52E_52.44N_ad_m__Oedothorax_apicatus
ZFMKTIS1994_8.67E_49.5N_ad_m__Oedothorax_apicatus
ZFMKTIS2534418_11.08E_54.38N__Oedothorax_apicatus
ZSMARACH00456_14.14E_52.88N_ad_m__Oedothorax_apicatus
ZFMKTIS7192_10.9E_53.96N_ad_m__Oedothorax_apicatus
ZFMKTIS18472_13.75E_51.1N_ad_m__Oedothorax_apicatus
ZFMKTIS2822_13.67E_54.52N_ad_m__Oedothorax_apicatus
ZFMKTIS22184_13.4E_54.28N_ad_m__Oedothorax_retusus
ZFMKTIS2494_13.6E_51.18N_ad_m__Oedothorax_retusus
ZFMKTIS2528505_11.32E_47.27N_ad_m__Oedothorax_retusus
ZFMKGBOL26222_7.16E_50.71N_ad_m__Oedothorax_retusus
0.02

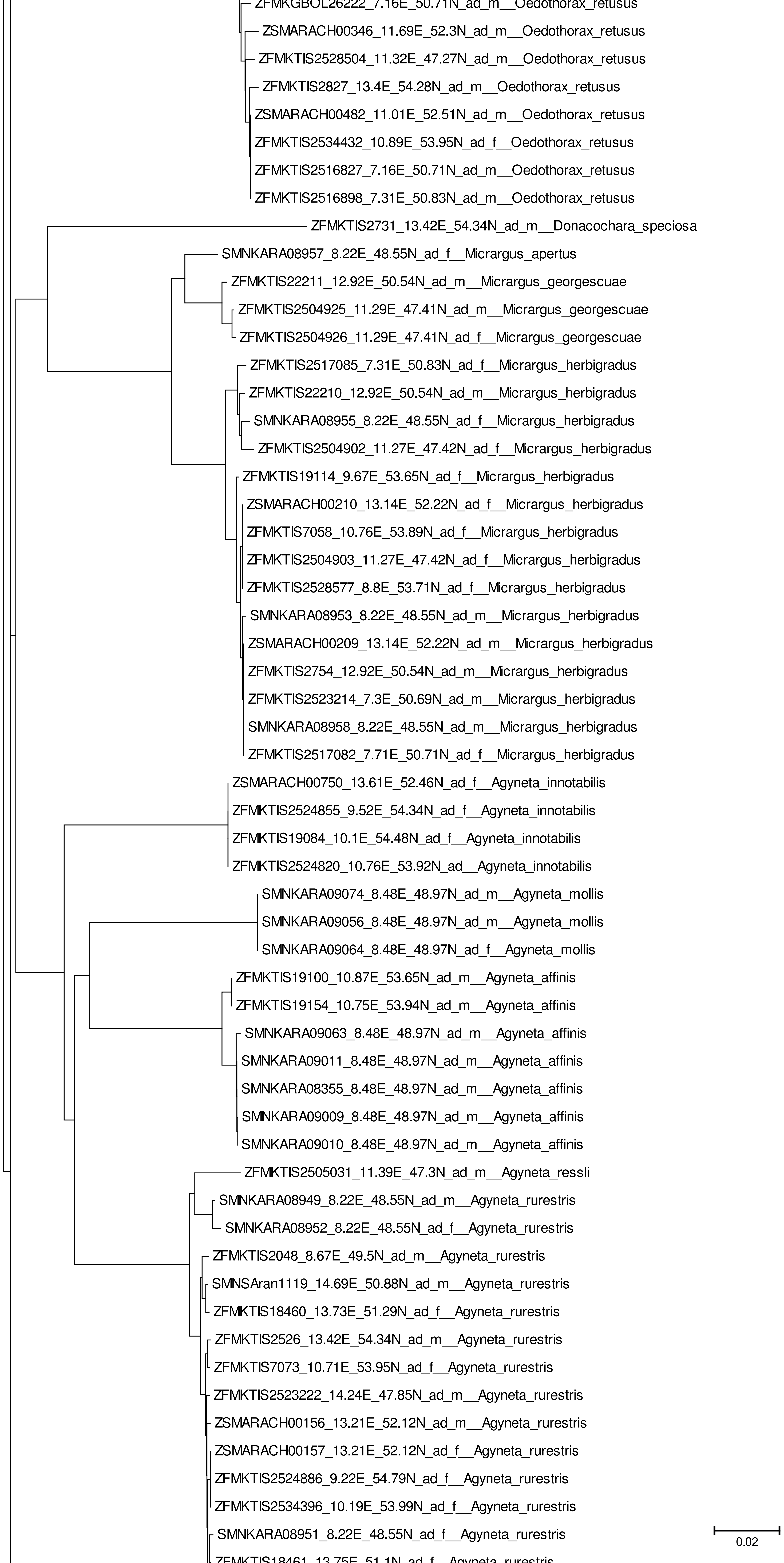
ZFMKGBOL26222_7.16E_50.71N_ad_m__Oedothorax_retusus
ZSMARACH00346_11.69E_52.3N_ad_m__Oedothorax_retusus
ZFMKTIS2528504_11.32E_47.27N_ad_m__Oedothorax_retusus
ZFMKTIS2827_13.4E_54.28N_ad_m__Oedothorax_retusus
ZSMARACH00482_11.01E_52.51N_ad_m__Oedothorax_retusus
ZFMKTIS2534432_10.89E_53.95N_ad_f__Oedothorax_retusus
ZFMKTIS2516827_7.16E_50.71N_ad_m__Oedothorax_retusus
ZFMKTIS2516898_7.31E_50.83N_ad_m__Oedothorax_retusus
ZFMKTIS2731_13.42E_54.34N_ad_m__Donacochara_speciosa
SMNKARA08957_8.22E_48.55N_ad_f__Micrargus_apertus
ZFMKTIS22211_12.92E_50.54N_ad_m__Micrargus_georgescuae
ZFMKTIS2504925_11.29E_47.41N_ad_m__Micrargus_georgescuae
ZFMKTIS2504926_11.29E_47.41N_ad_f__Micrargus_georgescuae
ZFMKTIS2517085_7.31E_50.83N_ad_f__Micrargus_herbigradus
ZFMKTIS22210_12.92E_50.54N_ad_m__Micrargus_herbigradus
SMNKARA08955_8.22E_48.55N_ad_f__Micrargus_herbigradus
ZFMKTIS2504902_11.27E_47.42N_ad_f__Micrargus_herbigradus
ZFMKTIS19114_9.67E_53.65N_ad_f__Micrargus_herbigradus
ZSMARACH00210_13.14E_52.22N_ad_f__Micrargus_herbigradus
ZFMKTIS7058_10.76E_53.89N_ad_f__Micrargus_herbigradus
ZFMKTIS2504903_11.27E_47.42N_ad_f__Micrargus_herbigradus
ZFMKTIS2528577_8.8E_53.71N_ad_f__Micrargus_herbigradus
SMNKARA08953_8.22E_48.55N_ad_m__Micrargus_herbigradus
ZSMARACH00209_13.14E_52.22N_ad_m__Micrargus_herbigradus
ZFMKTIS2754_12.92E_50.54N_ad_m__Micrargus_herbigradus
ZFMKTIS2523214_7.3E_50.69N_ad_m__Micrargus_herbigradus
SMNKARA08958_8.22E_48.55N_ad_m__Micrargus_herbigradus
ZFMKTIS2517082_7.71E_50.71N_ad_f__Micrargus_herbigradus
ZSMARACH00750_13.61E_52.46N_ad_f__Agyneta_innotabilis
ZFMKTIS2524855_9.52E_54.34N_ad_f__Agyneta_innotabilis
ZFMKTIS19084_10.1E_54.48N_ad_f__Agyneta_innotabilis
ZFMKTIS2524820_10.76E_53.92N_ad__Agyneta_innotabilis
SMNKARA09074_8.48E_48.97N_ad_m__Agyneta_mollis
SMNKARA09056_8.48E_48.97N_ad_m__Agyneta_mollis
SMNKARA09064_8.48E_48.97N_ad_f__Agyneta_mollis
ZFMKTIS19100_10.87E_53.65N_ad_m__Agyneta_affinis
ZFMKTIS19154_10.75E_53.94N_ad_m__Agyneta_affinis
SMNKARA09063_8.48E_48.97N_ad_m__Agyneta_affinis
SMNKARA09011_8.48E_48.97N_ad_m__Agyneta_affinis
SMNKARA08355_8.48E_48.97N_ad_m__Agyneta_affinis
SMNKARA09009_8.48E_48.97N_ad_m__Agyneta_affinis
SMNKARA09010_8.48E_48.97N_ad_m__Agyneta_affinis
ZFMKTIS2505031_11.39E_47.3N_ad_m__Agyneta_ressli
SMNKARA08949_8.22E_48.55N_ad_m__Agyneta_rurestris
SMNKARA08952_8.22E_48.55N_ad_f__Agyneta_rurestris
ZFMKTIS2048_8.67E_49.5N_ad_m__Agyneta_rurestris
SMNSAran1119_14.69E_50.88N_ad_m__Agyneta_rurestris
ZFMKTIS18460_13.73E_51.29N_ad_f__Agyneta_rurestris
ZFMKTIS2526_13.42E_54.34N_ad_m__Agyneta_rurestris
ZFMKTIS7073_10.71E_53.95N_ad_f__Agyneta_rurestris
ZFMKTIS2523222_14.24E_47.85N_ad_m__Agyneta_rurestris
ZSMARACH00156_13.21E_52.12N_ad_m__Agyneta_rurestris
ZSMARACH00157_13.21E_52.12N_ad_f__Agyneta_rurestris
ZFMKTIS2524886_9.22E_54.79N_ad_f__Agyneta_rurestris
ZFMKTIS2534396_10.19E_53.99N_ad_f__Agyneta_rurestris
SMNKARA08951_8.22E_48.55N_ad_f__Agyneta_rurestris
0.02

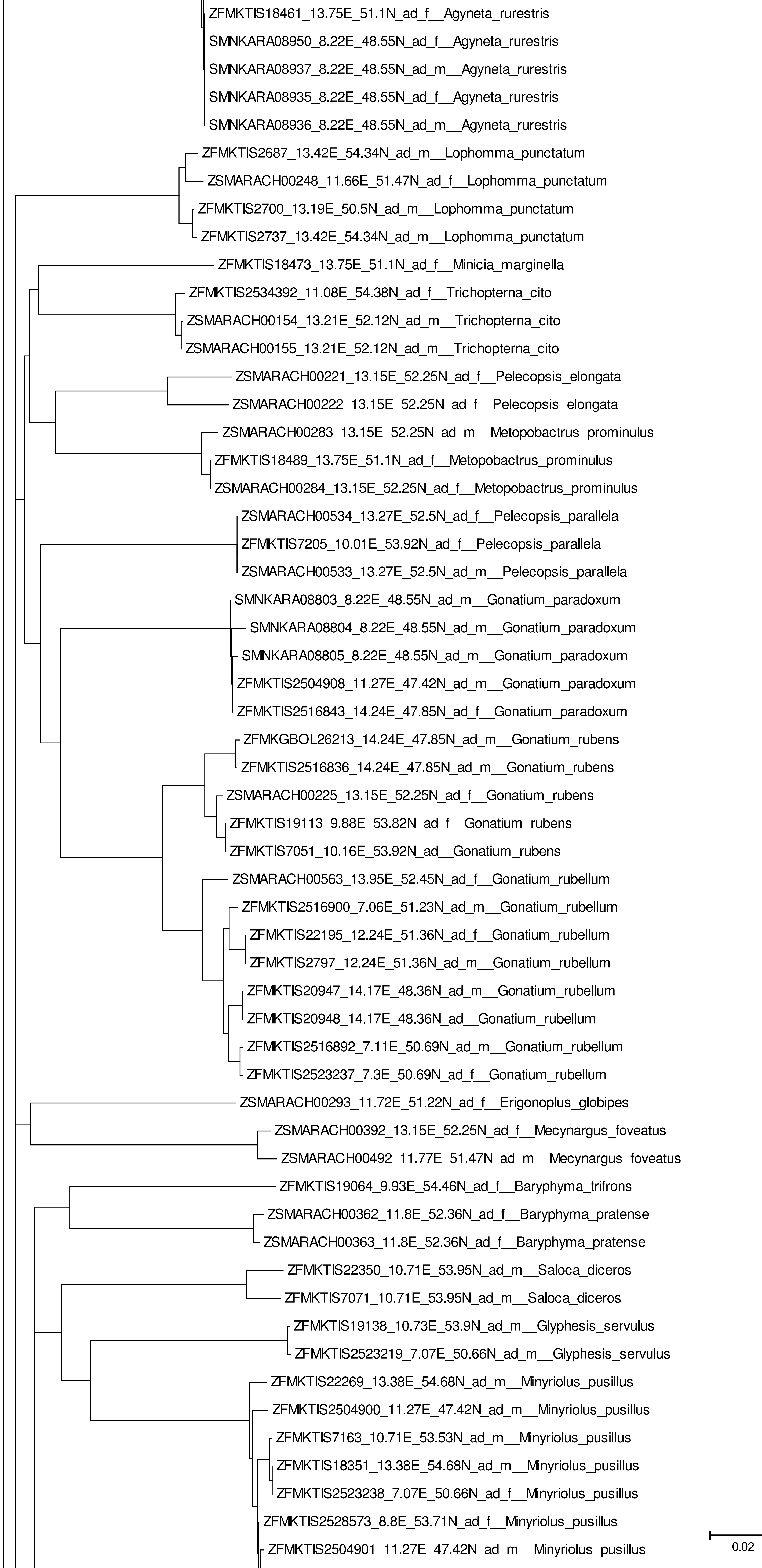
ZFMKTIS18461_13.75E_51.1N_ad_f__Agyneta_rurestris
SMNKARA08950_8.22E_48.55N_ad_f__Agyneta_rurestris
SMNKARA08937_8.22E_48.55N_ad_m__Agyneta_rurestris
SMNKARA08935_8.22E_48.55N_ad_f__Agyneta_rurestris
SMNKARA08936_8.22E_48.55N_ad_m__Agyneta_rurestris
ZFMKTIS2687_13.42E_54.34N_ad_m__Lophomma_punctatum
ZSMARACH00248_11.66E_51.47N_ad_f__Lophomma_punctatum
ZFMKTIS2700_13.19E_50.5N_ad_m__Lophomma_punctatum
ZFMKTIS2737_13.42E_54.34N_ad_m__Lophomma_punctatum
ZFMKTIS18473_13.75E_51.1N_ad_f__Minicia_marginella
ZFMKTIS2534392_11.08E_54.38N_ad_f__Trichopterna_cito
ZSMARACH00154_13.21E_52.12N_ad_m__Trichopterna_cito
ZSMARACH00155_13.21E_52.12N_ad_m__Trichopterna_cito
ZSMARACH00221_13.15E_52.25N_ad_f__Pelecopsis_elongata
ZSMARACH00222_13.15E_52.25N_ad_f__Pelecopsis_elongata
ZSMARACH00283_13.15E_52.25N_ad_m__Metopobactrus_prominulus
ZFMKTIS18489_13.75E_51.1N_ad_f__Metopobactrus_prominulus
ZSMARACH00284_13.15E_52.25N_ad_f__Metopobactrus_prominulus
ZSMARACH00534_13.27E_52.5N_ad_f__Pelecopsis_parallela
ZFMKTIS7205_10.01E_53.92N_ad_f__Pelecopsis_parallela
ZSMARACH00533_13.27E_52.5N_ad_m__Pelecopsis_parallela
SMNKARA08803_8.22E_48.55N_ad_m__Gonatium_paradoxum
SMNKARA08804_8.22E_48.55N_ad_m__Gonatium_paradoxum
SMNKARA08805_8.22E_48.55N_ad_m__Gonatium_paradoxum
ZFMKTIS2504908_11.27E_47.42N_ad_m__Gonatium_paradoxum
ZFMKTIS2516843_14.24E_47.85N_ad_f__Gonatium_paradoxum
ZFMKGBOL26213_14.24E_47.85N_ad_m__Gonatium_rubens
ZFMKTIS2516836_14.24E_47.85N_ad_m__Gonatium_rubens
ZSMARACH00225_13.15E_52.25N_ad_f__Gonatium_rubens
ZFMKTIS19113_9.88E_53.82N_ad_f__Gonatium_rubens
ZFMKTIS7051_10.16E_53.92N_ad__Gonatium_rubens
ZSMARACH00563_13.95E_52.45N_ad_f__Gonatium_rubellum
ZFMKTIS2516900_7.06E_51.23N_ad_m__Gonatium_rubellum
ZFMKTIS22195_12.24E_51.36N_ad_f__Gonatium_rubellum
ZFMKTIS2797_12.24E_51.36N_ad_m__Gonatium_rubellum
ZFMKTIS20947_14.17E_48.36N_ad_m__Gonatium_rubellum
ZFMKTIS20948_14.17E_48.36N_ad__Gonatium_rubellum
ZFMKTIS2516892_7.11E_50.69N_ad_m__Gonatium_rubellum
ZFMKTIS2523237_7.3E_50.69N_ad_f__Gonatium_rubellum
ZSMARACH00293_11.72E_51.22N_ad_f__Erigonoplus_globipes
ZSMARACH00392_13.15E_52.25N_ad_f__Mecynargus_foveatus
ZSMARACH00492_11.77E_51.47N_ad_m__Mecynargus_foveatus
ZFMKTIS19064_9.93E_54.46N_ad_f__Baryphyma_trifrons
ZSMARACH00362_11.8E_52.36N_ad_f__Baryphyma_pratense
ZSMARACH00363_11.8E_52.36N_ad_f__Baryphyma_pratense
ZFMKTIS22350_10.71E_53.95N_ad_m__Saloca_diceros
ZFMKTIS7071_10.71E_53.95N_ad_m__Saloca_diceros
ZFMKTIS19138_10.73E_53.9N_ad_m__Glyphesis_servulus
ZFMKTIS2523219_7.07E_50.66N_ad_m__Glyphesis_servulus
ZFMKTIS22269_13.38E_54.68N_ad_m__Minyriolus_pusillus
ZFMKTIS2504900_11.27E_47.42N_ad_m__Minyriolus_pusillus
ZFMKTIS7163_10.71E_53.53N_ad_m__Minyriolus_pusillus
ZFMKTIS18351_13.38E_54.68N_ad_m__Minyriolus_pusillus
ZFMKTIS2523238_7.07E_50.66N_ad_f__Minyriolus_pusillus
ZFMKTIS2528573_8.8E_53.71N_ad_f__Minyriolus_pusillus
ZFMKTIS2504901_11.27E_47.42N_ad_m__Minyriolus_pusillus
0.02

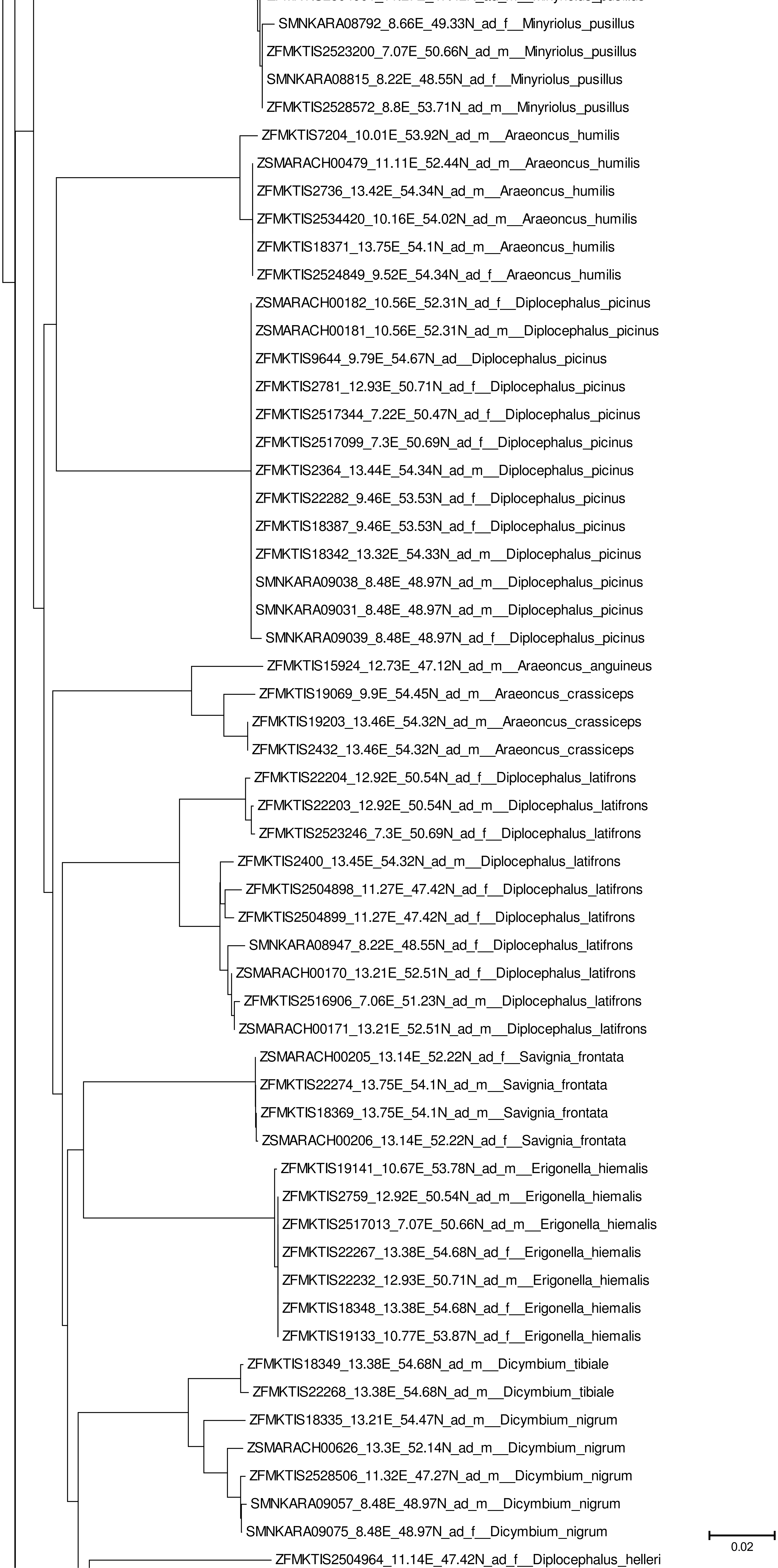
SMNKARA08792_8.66E_49.33N_ad_f__Minyriolus_pusillus
ZFMKTIS2523200_7.07E_50.66N_ad_m__Minyriolus_pusillus
SMNKARA08815_8.22E_48.55N_ad_f__Minyriolus_pusillus
ZFMKTIS2528572_8.8E_53.71N_ad_m__Minyriolus_pusillus
ZFMKTIS7204_10.01E_53.92N_ad_m__Araeoncus_humilis
ZSMARACH00479_11.11E_52.44N_ad_m__Araeoncus_humilis
ZFMKTIS2736_13.42E_54.34N_ad_m__Araeoncus_humilis
ZFMKTIS2534420_10.16E_54.02N_ad_m__Araeoncus_humilis
ZFMKTIS18371_13.75E_54.1N_ad_m__Araeoncus_humilis
ZFMKTIS2524849_9.52E_54.34N_ad_f__Araeoncus_humilis
ZSMARACH00182_10.56E_52.31N_ad_f__Diplocephalus_picinus
ZSMARACH00181_10.56E_52.31N_ad_m__Diplocephalus_picinus
ZFMKTIS9644_9.79E_54.67N_ad__Diplocephalus_picinus
ZFMKTIS2781_12.93E_50.71N_ad_f__Diplocephalus_picinus
ZFMKTIS2517344_7.22E_50.47N_ad_f__Diplocephalus_picinus
ZFMKTIS2517099_7.3E_50.69N_ad_f__Diplocephalus_picinus
ZFMKTIS2364_13.44E_54.34N_ad_m__Diplocephalus_picinus
ZFMKTIS22282_9.46E_53.53N_ad_f__Diplocephalus_picinus
ZFMKTIS18387_9.46E_53.53N_ad_f__Diplocephalus_picinus
ZFMKTIS18342_13.32E_54.33N_ad_m__Diplocephalus_picinus
SMNKARA09038_8.48E_48.97N_ad_m__Diplocephalus_picinus
SMNKARA09031_8.48E_48.97N_ad_m__Diplocephalus_picinus
SMNKARA09039_8.48E_48.97N_ad_f__Diplocephalus_picinus
ZFMKTIS15924_12.73E_47.12N_ad_m__Araeoncus_anguineus
ZFMKTIS19069_9.9E_54.45N_ad_m__Araeoncus_crassiceps
ZFMKTIS19203_13.46E_54.32N_ad_m__Araeoncus_crassiceps
ZFMKTIS2432_13.46E_54.32N_ad_m__Araeoncus_crassiceps
ZFMKTIS22204_12.92E_50.54N_ad_f__Diplocephalus_latifrons
ZFMKTIS22203_12.92E_50.54N_ad_m__Diplocephalus_latifrons
ZFMKTIS2523246_7.3E_50.69N_ad_f__Diplocephalus_latifrons
ZFMKTIS2400_13.45E_54.32N_ad_m__Diplocephalus_latifrons
ZFMKTIS2504898_11.27E_47.42N_ad_f__Diplocephalus_latifrons
ZFMKTIS2504899_11.27E_47.42N_ad_f__Diplocephalus_latifrons
SMNKARA08947_8.22E_48.55N_ad_f__Diplocephalus_latifrons
ZSMARACH00170_13.21E_52.51N_ad_f__Diplocephalus_latifrons
ZFMKTIS2516906_7.06E_51.23N_ad_m__Diplocephalus_latifrons
ZSMARACH00171_13.21E_52.51N_ad_m__Diplocephalus_latifrons
ZSMARACH00205_13.14E_52.22N_ad_f__Savignia_frontata
ZFMKTIS22274_13.75E_54.1N_ad_m__Savignia_frontata
ZFMKTIS18369_13.75E_54.1N_ad_m__Savignia_frontata
ZSMARACH00206_13.14E_52.22N_ad_f__Savignia_frontata
ZFMKTIS19141_10.67E_53.78N_ad_m__Erigonella_hiemalis
ZFMKTIS2759_12.92E_50.54N_ad_m__Erigonella_hiemalis
ZFMKTIS2517013_7.07E_50.66N_ad_m__Erigonella_hiemalis
ZFMKTIS22267_13.38E_54.68N_ad_f__Erigonella_hiemalis
ZFMKTIS22232_12.93E_50.71N_ad_m__Erigonella_hiemalis
ZFMKTIS18348_13.38E_54.68N_ad_f__Erigonella_hiemalis
ZFMKTIS19133_10.77E_53.87N_ad_f__Erigonella_hiemalis
ZFMKTIS18349_13.38E_54.68N_ad_m__Dicymbium_tibiale
ZFMKTIS22268_13.38E_54.68N_ad_m__Dicymbium_tibiale
ZFMKTIS18335_13.21E_54.47N_ad_m__Dicymbium_nigrum
ZSMARACH00626_13.3E_52.14N_ad_m__Dicymbium_nigrum
ZFMKTIS2528506_11.32E_47.27N_ad_m__Dicymbium_nigrum
SMNKARA09057_8.48E_48.97N_ad_m__Dicymbium_nigrum
SMNKARA09075_8.48E_48.97N_ad_f__Dicymbium_nigrum
0.02
ZFMKTIS2504964_11.14E_47.42N_ad_f__Diplocephalus_helleri

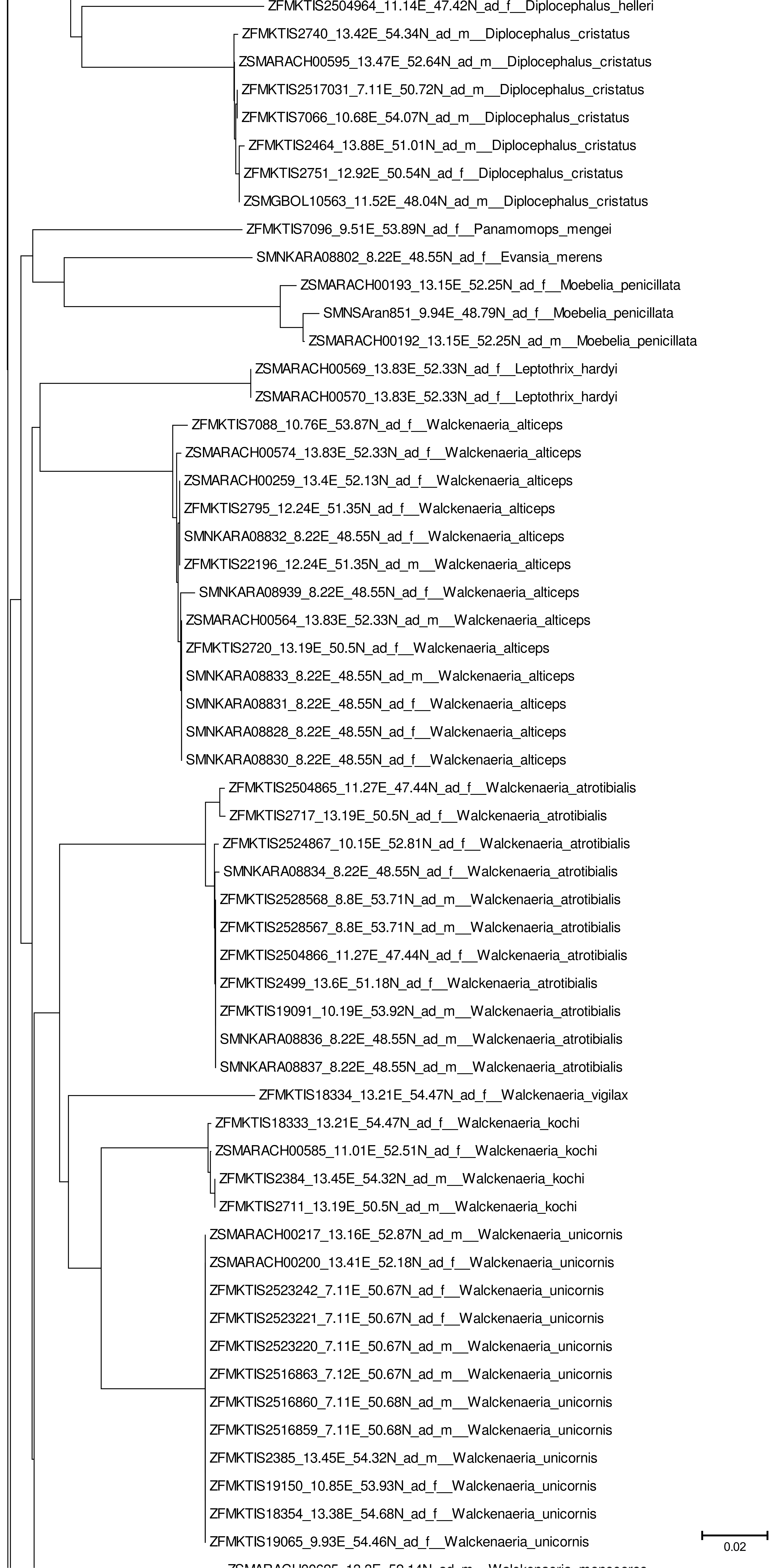
ZFMKTIS2504964_11.14E_47.42N_ad_f__Diplocephalus_helleri
ZFMKTIS2740_13.42E_54.34N_ad_m__Diplocephalus_cristatus
ZSMARACH00595_13.47E_52.64N_ad_m__Diplocephalus_cristatus
ZFMKTIS2517031_7.11E_50.72N_ad_m__Diplocephalus_cristatus
ZFMKTIS7066_10.68E_54.07N_ad_m__Diplocephalus_cristatus
ZFMKTIS2464_13.88E_51.01N_ad_m__Diplocephalus_cristatus
ZFMKTIS2751_12.92E_50.54N_ad_f__Diplocephalus_cristatus
ZSMGBOL10563_11.52E_48.04N_ad_m__Diplocephalus_cristatus
ZFMKTIS7096_9.51E_53.89N_ad_f__Panamomops_mengei
SMNKARA08802_8.22E_48.55N_ad_f__Evansia_merens
ZSMARACH00193_13.15E_52.25N_ad_f__Moebelia_penicillata
SMNSAran851_9.94E_48.79N_ad_f__Moebelia_penicillata
ZSMARACH00192_13.15E_52.25N_ad_m__Moebelia_penicillata
ZSMARACH00569_13.83E_52.33N_ad_f__Leptothrix_hardyi
ZSMARACH00570_13.83E_52.33N_ad_f__Leptothrix_hardyi
ZFMKTIS7088_10.76E_53.87N_ad_f__Walckenaeria_alticeps
ZSMARACH00574_13.83E_52.33N_ad_f__Walckenaeria_alticeps
ZSMARACH00259_13.4E_52.13N_ad_f__Walckenaeria_alticeps
ZFMKTIS2795_12.24E_51.35N_ad_f__Walckenaeria_alticeps
SMNKARA08832_8.22E_48.55N_ad_f__Walckenaeria_alticeps
ZFMKTIS22196_12.24E_51.35N_ad_m__Walckenaeria_alticeps
SMNKARA08939_8.22E_48.55N_ad_f__Walckenaeria_alticeps
ZSMARACH00564_13.83E_52.33N_ad_m__Walckenaeria_alticeps
ZFMKTIS2720_13.19E_50.5N_ad_f__Walckenaeria_alticeps
SMNKARA08833_8.22E_48.55N_ad_m__Walckenaeria_alticeps
SMNKARA08831_8.22E_48.55N_ad_f__Walckenaeria_alticeps
SMNKARA08828_8.22E_48.55N_ad_f__Walckenaeria_alticeps
SMNKARA08830_8.22E_48.55N_ad_f__Walckenaeria_alticeps
ZFMKTIS2504865_11.27E_47.44N_ad_f__Walckenaeria_atrotibialis
ZFMKTIS2717_13.19E_50.5N_ad_f__Walckenaeria_atrotibialis
ZFMKTIS2524867_10.15E_52.81N_ad_f__Walckenaeria_atrotibialis
SMNKARA08834_8.22E_48.55N_ad_f__Walckenaeria_atrotibialis
ZFMKTIS2528568_8.8E_53.71N_ad_m__Walckenaeria_atrotibialis
ZFMKTIS2528567_8.8E_53.71N_ad_m__Walckenaeria_atrotibialis
ZFMKTIS2504866_11.27E_47.44N_ad_f__Walckenaeria_atrotibialis
ZFMKTIS2499_13.6E_51.18N_ad_f__Walckenaeria_atrotibialis
ZFMKTIS19091_10.19E_53.92N_ad_m__Walckenaeria_atrotibialis
SMNKARA08836_8.22E_48.55N_ad_m__Walckenaeria_atrotibialis
SMNKARA08837_8.22E_48.55N_ad_m__Walckenaeria_atrotibialis
ZFMKTIS18334_13.21E_54.47N_ad_f__Walckenaeria_vigilax
ZFMKTIS18333_13.21E_54.47N_ad_f__Walckenaeria_kochi
ZSMARACH00585_11.01E_52.51N_ad_f__Walckenaeria_kochi
ZFMKTIS2384_13.45E_54.32N_ad_m__Walckenaeria_kochi
ZFMKTIS2711_13.19E_50.5N_ad_m__Walckenaeria_kochi
ZSMARACH00217_13.16E_52.87N_ad_m__Walckenaeria_unicornis
ZSMARACH00200_13.41E_52.18N_ad_f__Walckenaeria_unicornis
ZFMKTIS2523242_7.11E_50.67N_ad_f__Walckenaeria_unicornis
ZFMKTIS2523221_7.11E_50.67N_ad_f__Walckenaeria_unicornis
ZFMKTIS2523220_7.11E_50.67N_ad_m__Walckenaeria_unicornis
ZFMKTIS2516863_7.12E_50.67N_ad_m__Walckenaeria_unicornis
ZFMKTIS2516860_7.11E_50.68N_ad_m__Walckenaeria_unicornis
ZFMKTIS2516859_7.11E_50.68N_ad_m__Walckenaeria_unicornis
ZFMKTIS2385_13.45E_54.32N_ad_m__Walckenaeria_unicornis
ZFMKTIS19150_10.85E_53.93N_ad_f__Walckenaeria_unicornis
ZFMKTIS18354_13.38E_54.68N_ad_f__Walckenaeria_unicornis
ZFMKTIS19065_9.93E_54.46N_ad_f__Walckenaeria_unicornis
0.02

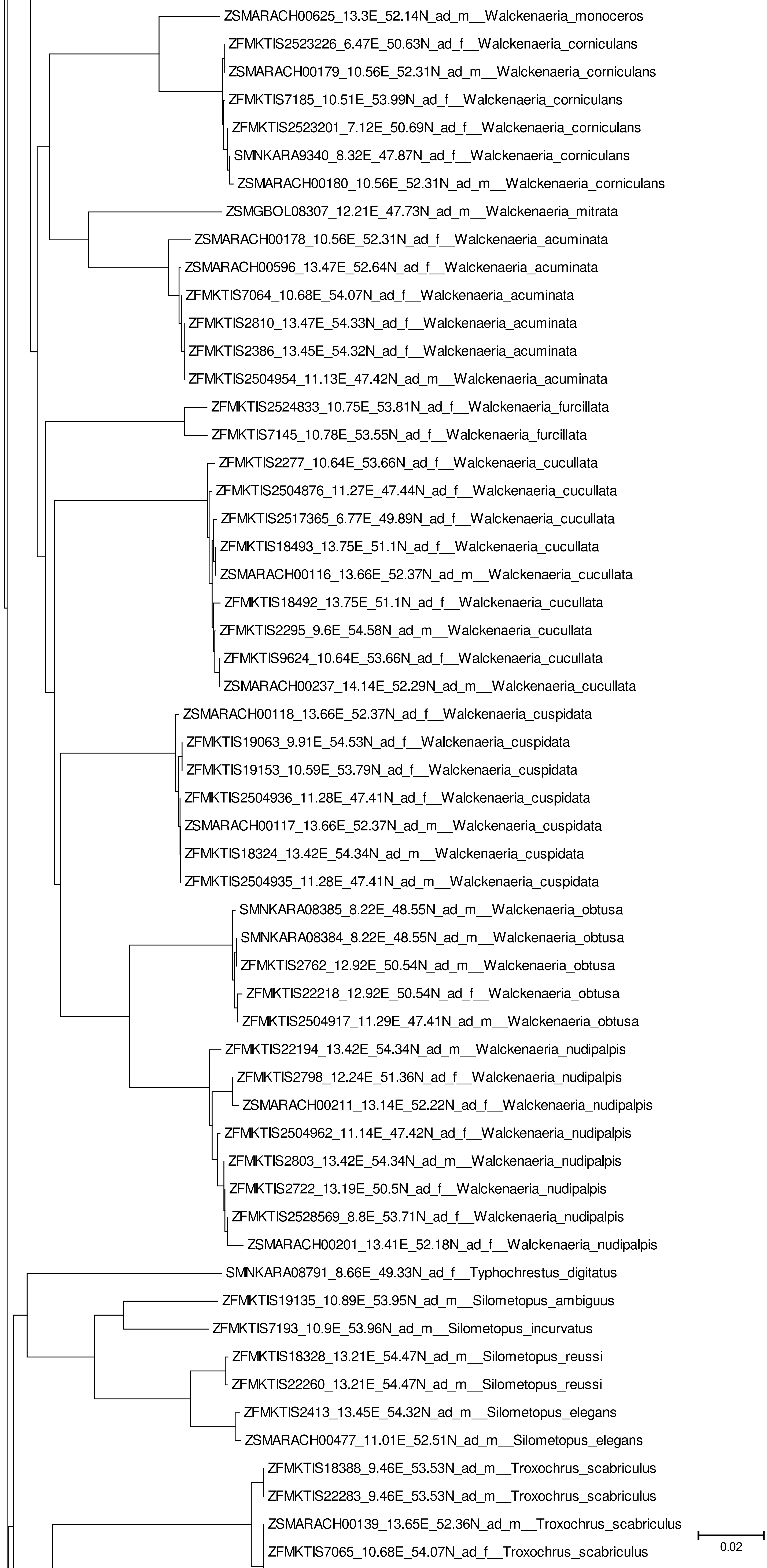
ZSMARACH00625_13.3E_52.14N_ad_m__Walckenaeria_monoceros
ZFMKTIS2523226_6.47E_50.63N_ad_f__Walckenaeria_corniculans
ZSMARACH00179_10.56E_52.31N_ad_m__Walckenaeria_corniculans
ZFMKTIS7185_10.51E_53.99N_ad_f__Walckenaeria_corniculans
ZFMKTIS2523201_7.12E_50.69N_ad_f__Walckenaeria_corniculans
SMNKARA9340_8.32E_47.87N_ad_f__Walckenaeria_corniculans
ZSMARACH00180_10.56E_52.31N_ad_m__Walckenaeria_corniculans
ZSMGBOL08307_12.21E_47.73N_ad_m__Walckenaeria_mitrata
ZSMARACH00178_10.56E_52.31N_ad_f__Walckenaeria_acuminata
ZSMARACH00596_13.47E_52.64N_ad_f__Walckenaeria_acuminata
ZFMKTIS7064_10.68E_54.07N_ad_f__Walckenaeria_acuminata
ZFMKTIS2810_13.47E_54.33N_ad_f__Walckenaeria_acuminata
ZFMKTIS2386_13.45E_54.32N_ad_f__Walckenaeria_acuminata
ZFMKTIS2504954_11.13E_47.42N_ad_m__Walckenaeria_acuminata
ZFMKTIS2524833_10.75E_53.81N_ad_f__Walckenaeria_furcillata
ZFMKTIS7145_10.78E_53.55N_ad_f__Walckenaeria_furcillata
ZFMKTIS2277_10.64E_53.66N_ad_f__Walckenaeria_cucullata
ZFMKTIS2504876_11.27E_47.44N_ad_f__Walckenaeria_cucullata
ZFMKTIS2517365_6.77E_49.89N_ad_f__Walckenaeria_cucullata
ZFMKTIS18493_13.75E_51.1N_ad_f__Walckenaeria_cucullata
ZSMARACH00116_13.66E_52.37N_ad_m__Walckenaeria_cucullata
ZFMKTIS18492_13.75E_51.1N_ad_f__Walckenaeria_cucullata
ZFMKTIS2295_9.6E_54.58N_ad_m__Walckenaeria_cucullata
ZFMKTIS9624_10.64E_53.66N_ad_f__Walckenaeria_cucullata
ZSMARACH00237_14.14E_52.29N_ad_m__Walckenaeria_cucullata
ZSMARACH00118_13.66E_52.37N_ad_f__Walckenaeria_cuspidata
ZFMKTIS19063_9.91E_54.53N_ad_f__Walckenaeria_cuspidata
ZFMKTIS19153_10.59E_53.79N_ad_f__Walckenaeria_cuspidata
ZFMKTIS2504936_11.28E_47.41N_ad_f__Walckenaeria_cuspidata
ZSMARACH00117_13.66E_52.37N_ad_m__Walckenaeria_cuspidata
ZFMKTIS18324_13.42E_54.34N_ad_m__Walckenaeria_cuspidata
ZFMKTIS2504935_11.28E_47.41N_ad_m__Walckenaeria_cuspidata
SMNKARA08385_8.22E_48.55N_ad_m__Walckenaeria_obtusa
SMNKARA08384_8.22E_48.55N_ad_m__Walckenaeria_obtusa
ZFMKTIS2762_12.92E_50.54N_ad_m__Walckenaeria_obtusa
ZFMKTIS22218_12.92E_50.54N_ad_f__Walckenaeria_obtusa
ZFMKTIS2504917_11.29E_47.41N_ad_m__Walckenaeria_obtusa
ZFMKTIS22194_13.42E_54.34N_ad_m__Walckenaeria_nudipalpis
ZFMKTIS2798_12.24E_51.36N_ad_f__Walckenaeria_nudipalpis
ZSMARACH00211_13.14E_52.22N_ad_f__Walckenaeria_nudipalpis
ZFMKTIS2504962_11.14E_47.42N_ad_f__Walckenaeria_nudipalpis
ZFMKTIS2803_13.42E_54.34N_ad_m__Walckenaeria_nudipalpis
ZFMKTIS2722_13.19E_50.5N_ad_f__Walckenaeria_nudipalpis
ZFMKTIS2528569_8.8E_53.71N_ad_f__Walckenaeria_nudipalpis
ZSMARACH00201_13.41E_52.18N_ad_f__Walckenaeria_nudipalpis
SMNKARA08791_8.66E_49.33N_ad_f__Typhochrestus_digitatus
ZFMKTIS19135_10.89E_53.95N_ad_m__Silometopus_ambiguus
ZFMKTIS7193_10.9E_53.96N_ad_m__Silometopus_incurvatus
ZFMKTIS18328_13.21E_54.47N_ad_m__Silometopus_reussi
ZFMKTIS22260_13.21E_54.47N_ad_m__Silometopus_reussi
ZFMKTIS2413_13.45E_54.32N_ad_m__Silometopus_elegans
ZSMARACH00477_11.01E_52.51N_ad_m__Silometopus_elegans
ZFMKTIS18388_9.46E_53.53N_ad_m__Troxochrus_scabriculus
ZFMKTIS22283_9.46E_53.53N_ad_m__Troxochrus_scabriculus
ZSMARACH00139_13.65E_52.36N_ad_m__Troxochrus_scabriculus
ZFMKTIS7065_10.68E_54.07N_ad_f__Troxochrus_scabriculus
0.02

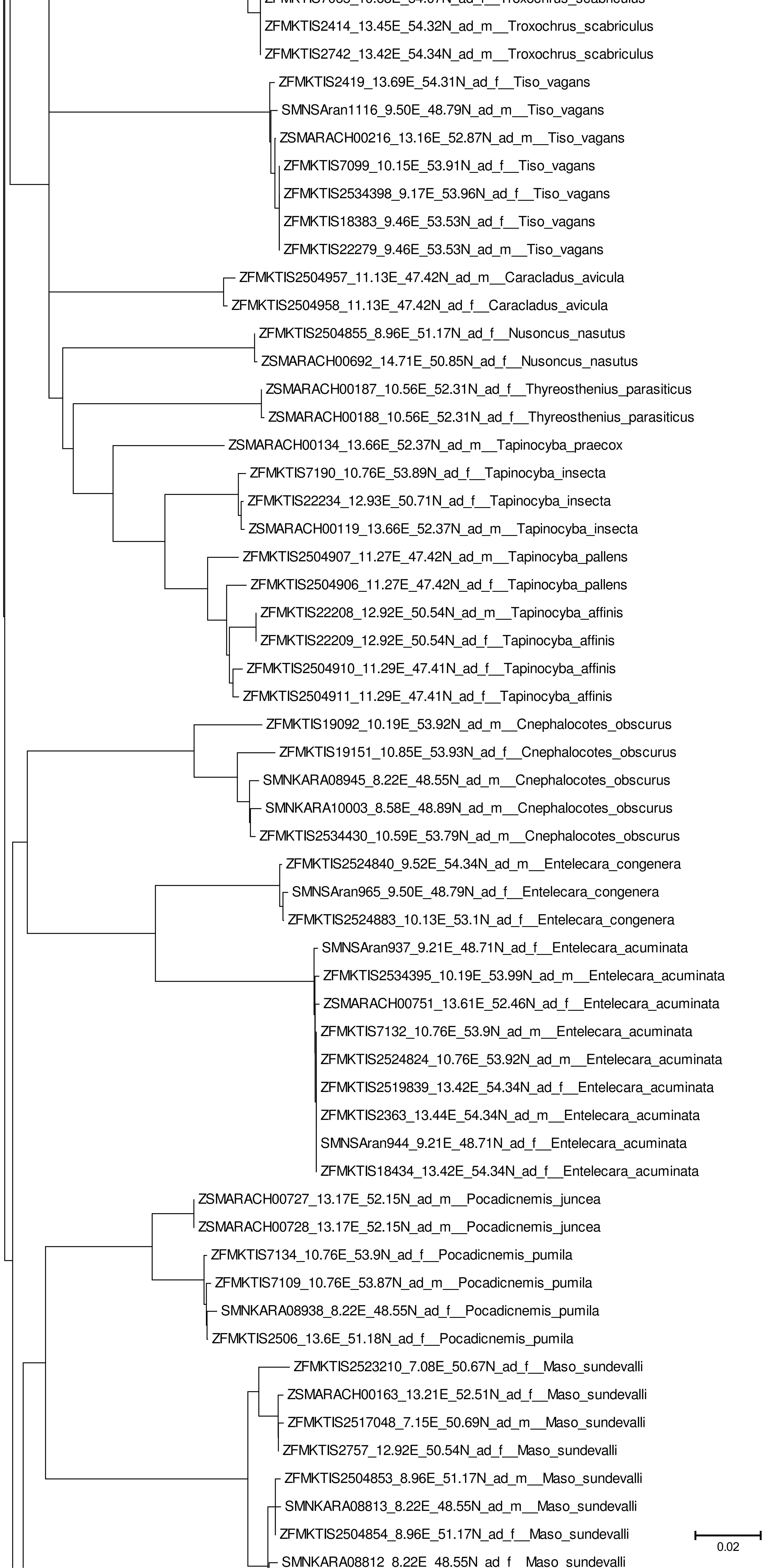
ZFMKTIS2414_13.45E_54.32N_ad_m__Troxochrus_scabriculus
ZFMKTIS2742_13.42E_54.34N_ad_m__Troxochrus_scabriculus
ZFMKTIS2419_13.69E_54.31N_ad_f__Tiso_vagans
SMNSAran1116_9.50E_48.79N_ad_m__Tiso_vagans
ZSMARACH00216_13.16E_52.87N_ad_m__Tiso_vagans
ZFMKTIS7099_10.15E_53.91N_ad_f__Tiso_vagans
ZFMKTIS2534398_9.17E_53.96N_ad_f__Tiso_vagans
ZFMKTIS18383_9.46E_53.53N_ad_f__Tiso_vagans
ZFMKTIS22279_9.46E_53.53N_ad_m__Tiso_vagans
ZFMKTIS2504957_11.13E_47.42N_ad_m__Caracladus_avicula
ZFMKTIS2504958_11.13E_47.42N_ad_f__Caracladus_avicula
ZFMKTIS2504855_8.96E_51.17N_ad_f__Nusoncus_nasutus
ZSMARACH00692_14.71E_50.85N_ad_f__Nusoncus_nasutus
ZSMARACH00187_10.56E_52.31N_ad_f__Thyreosthenius_parasiticus
ZSMARACH00188_10.56E_52.31N_ad_f__Thyreosthenius_parasiticus
ZSMARACH00134_13.66E_52.37N_ad_m__Tapinocyba_praecox
ZFMKTIS7190_10.76E_53.89N_ad_f__Tapinocyba_insecta
ZFMKTIS22234_12.93E_50.71N_ad_f__Tapinocyba_insecta
ZSMARACH00119_13.66E_52.37N_ad_m__Tapinocyba_insecta
ZFMKTIS2504907_11.27E_47.42N_ad_m__Tapinocyba_pallens
ZFMKTIS2504906_11.27E_47.42N_ad_f__Tapinocyba_pallens
ZFMKTIS22208_12.92E_50.54N_ad_m__Tapinocyba_affinis
ZFMKTIS22209_12.92E_50.54N_ad_f__Tapinocyba_affinis
ZFMKTIS2504910_11.29E_47.41N_ad_f__Tapinocyba_affinis
ZFMKTIS2504911_11.29E_47.41N_ad_f__Tapinocyba_affinis
ZFMKTIS19092_10.19E_53.92N_ad_m__Cnephalocotes_obscurus
ZFMKTIS19151_10.85E_53.93N_ad_f__Cnephalocotes_obscurus
SMNKARA08945_8.22E_48.55N_ad_m__Cnephalocotes_obscurus
SMNKARA10003_8.58E_48.89N_ad_m__Cnephalocotes_obscurus
ZFMKTIS2534430_10.59E_53.79N_ad_m__Cnephalocotes_obscurus
ZFMKTIS2524840_9.52E_54.34N_ad_m__Entelecara_congenera
SMNSAran965_9.50E_48.79N_ad_f__Entelecara_congenera
ZFMKTIS2524883_10.13E_53.1N_ad_f__Entelecara_congenera
SMNSAran937_9.21E_48.71N_ad_f__Entelecara_acuminata
ZFMKTIS2534395_10.19E_53.99N_ad_m__Entelecara_acuminata
ZSMARACH00751_13.61E_52.46N_ad_f__Entelecara_acuminata
ZFMKTIS7132_10.76E_53.9N_ad_m__Entelecara_acuminata
ZFMKTIS2524824_10.76E_53.92N_ad_m__Entelecara_acuminata
ZFMKTIS2519839_13.42E_54.34N_ad_f__Entelecara_acuminata
ZFMKTIS2363_13.44E_54.34N_ad_m__Entelecara_acuminata
SMNSAran944_9.21E_48.71N_ad_f__Entelecara_acuminata
ZFMKTIS18434_13.42E_54.34N_ad_f__Entelecara_acuminata
ZSMARACH00727_13.17E_52.15N_ad_m__Pocadicnemis_juncea
ZSMARACH00728_13.17E_52.15N_ad_m__Pocadicnemis_juncea
ZFMKTIS7134_10.76E_53.9N_ad_f__Pocadicnemis_pumila
ZFMKTIS7109_10.76E_53.87N_ad_m__Pocadicnemis_pumila
SMNKARA08938_8.22E_48.55N_ad_f__Pocadicnemis_pumila
ZFMKTIS2506_13.6E_51.18N_ad_f__Pocadicnemis_pumila
ZFMKTIS2523210_7.08E_50.67N_ad_f__Maso_sundevalli
ZSMARACH00163_13.21E_52.51N_ad_f__Maso_sundevalli
ZFMKTIS2517048_7.15E_50.69N_ad_m__Maso_sundevalli
ZFMKTIS2757_12.92E_50.54N_ad_f__Maso_sundevalli
ZFMKTIS2504853_8.96E_51.17N_ad_m__Maso_sundevalli
SMNKARA08813_8.22E_48.55N_ad_m__Maso_sundevalli
ZFMKTIS2504854_8.96E_51.17N_ad_f__Maso_sundevalli
SMNKARA08812_8.22E_48.55N_ad_f__Maso_sundevalli
0.02

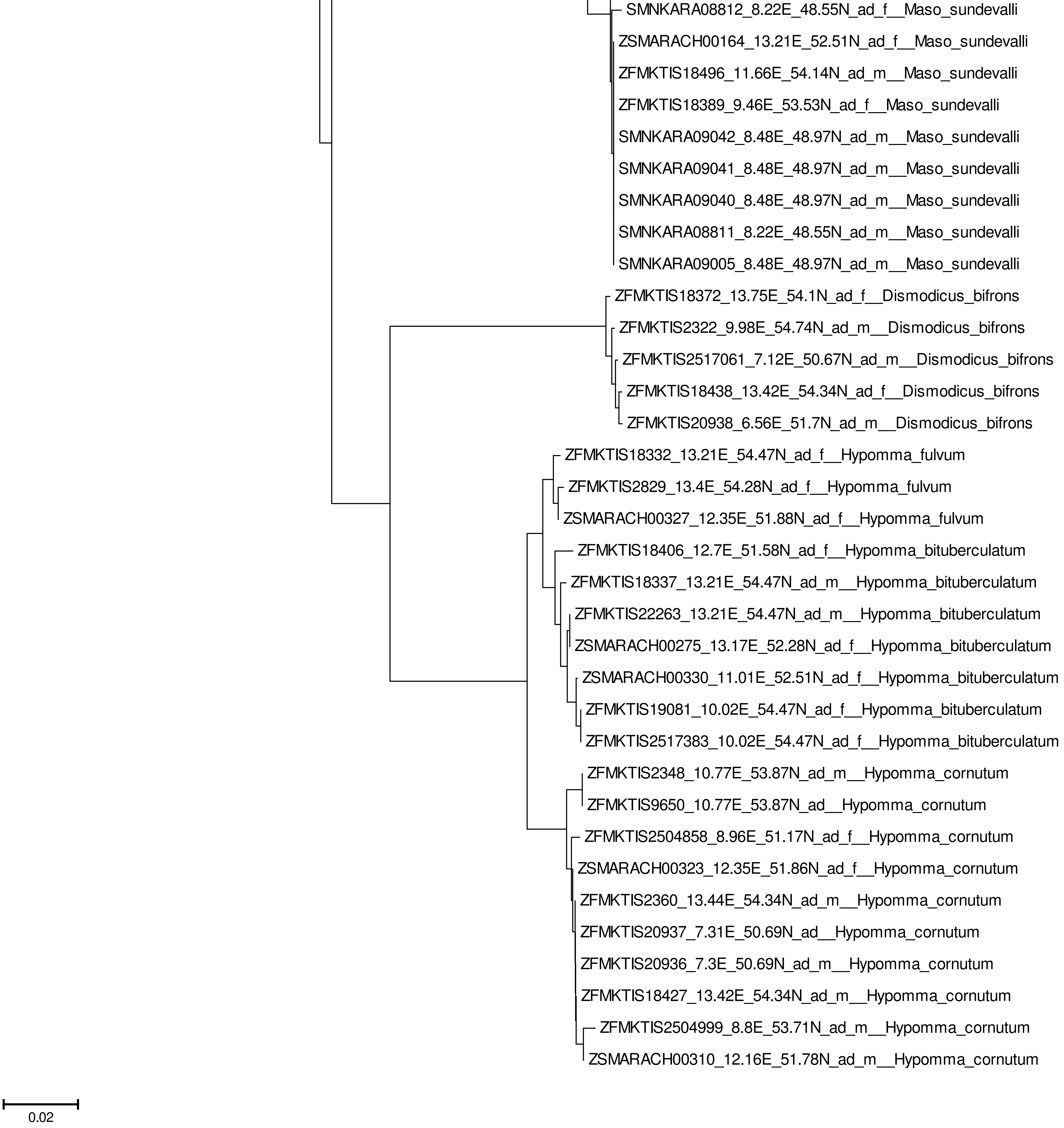
SMNKARA08812_8.22E_48.55N_ad_f__Maso_sundevalli
ZSMARACH00164_13.21E_52.51N_ad_f__Maso_sundevalli
ZFMKTIS18496_11.66E_54.14N_ad_m__Maso_sundevalli
ZFMKTIS18389_9.46E_53.53N_ad_f__Maso_sundevalli
SMNKARA09042_8.48E_48.97N_ad_m__Maso_sundevalli
SMNKARA09041_8.48E_48.97N_ad_m__Maso_sundevalli
SMNKARA09040_8.48E_48.97N_ad_m__Maso_sundevalli
SMNKARA08811_8.22E_48.55N_ad_m__Maso_sundevalli
SMNKARA09005_8.48E_48.97N_ad_m__Maso_sundevalli
ZFMKTIS18372_13.75E_54.1N_ad_f__Dismodicus_bifrons
ZFMKTIS2322_9.98E_54.74N_ad_m__Dismodicus_bifrons
ZFMKTIS2517061_7.12E_50.67N_ad_m__Dismodicus_bifrons
ZFMKTIS18438_13.42E_54.34N_ad_f__Dismodicus_bifrons
ZFMKTIS20938_6.56E_51.7N_ad_m__Dismodicus_bifrons
ZFMKTIS18332_13.21E_54.47N_ad_f__Hypomma_fulvum
ZFMKTIS2829_13.4E_54.28N_ad_f__Hypomma_fulvum
ZSMARACH00327_12.35E_51.88N_ad_f__Hypomma_fulvum
ZFMKTIS18406_12.7E_51.58N_ad_f__Hypomma_bituberculatum
ZFMKTIS18337_13.21E_54.47N_ad_m__Hypomma_bituberculatum
ZFMKTIS22263_13.21E_54.47N_ad_m__Hypomma_bituberculatum
ZSMARACH00275_13.17E_52.28N_ad_f__Hypomma_bituberculatum
ZSMARACH00330_11.01E_52.51N_ad_f__Hypomma_bituberculatum
ZFMKTIS19081_10.02E_54.47N_ad_f__Hypomma_bituberculatum
ZFMKTIS2517383_10.02E_54.47N_ad_f__Hypomma_bituberculatum
ZFMKTIS2348_10.77E_53.87N_ad_m__Hypomma_cornutum
ZFMKTIS9650_10.77E_53.87N_ad__Hypomma_cornutum
ZFMKTIS2504858_8.96E_51.17N_ad_f__Hypomma_cornutum
ZSMARACH00323_12.35E_51.86N_ad_f__Hypomma_cornutum
ZFMKTIS2360_13.44E_54.34N_ad_m__Hypomma_cornutum
ZFMKTIS20937_7.31E_50.69N_ad__Hypomma_cornutum
ZFMKTIS20936_7.3E_50.69N_ad_m__Hypomma_cornutum
ZFMKTIS18427_13.42E_54.34N_ad_m__Hypomma_cornutum
ZFMKTIS2504999_8.8E_53.71N_ad_m__Hypomma_cornutum
ZSMARACH00310_12.16E_51.78N_ad_m__Hypomma_cornutum
0.02

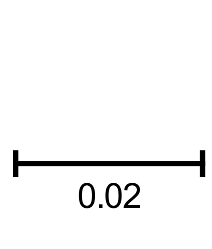
0.02
