# Supplementary material for: Towards a DNA Barcode Reference Database for Spiders and Harvestmen of Germany
Source: PLoS One. 2016 Sep 28;11(9):e0162624. doi: 10.1371/journal.pone.0162624 (PMC5040438; doi:10.1371/journal.pone.0162624)
Supplement: S2 Fig — The analysis was run for 1 million generations and includes 1000 bootstrap replicates. Apart from ID and species name, life stage, sex and coordinates of collecting locality are given. See S1 Table for more details on individual specimens in the tree. (PDF) [file pone.0162624.s003.pdf]

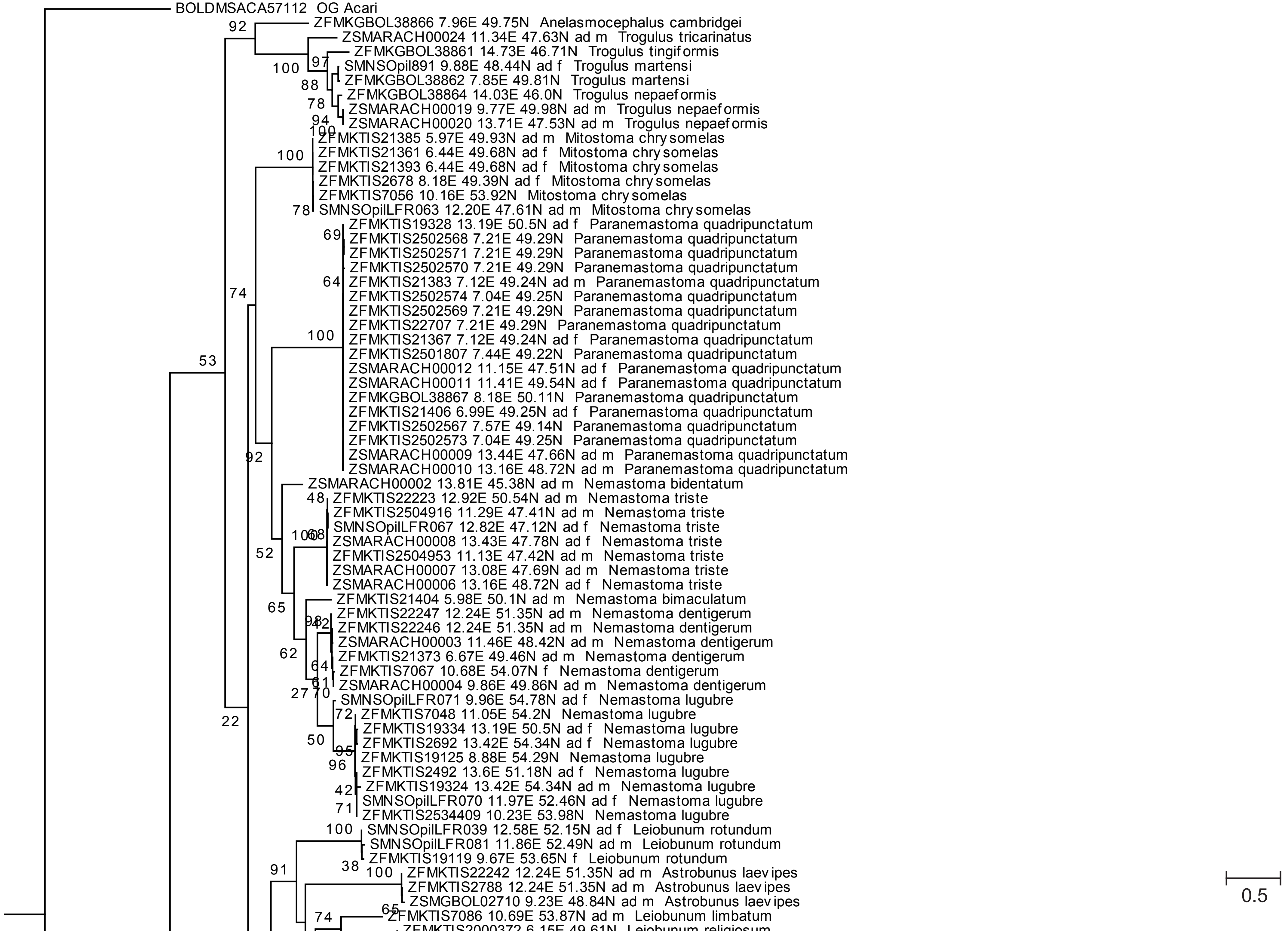
BOLDMSACA57112 OG Acari
ZFMKGBOL38866 7.96E 49.75N Anelasmocephalus cambridgei
ZSMARACH00024 11.34E 47.63N ad m Trogulus tricarinatus
ZFMKGBOL38861 14.73E 46.71N Trogulus tingiformis
SMNSOpil891 9.88E 48.44N ad f Trogulus martensi
ZFMKGBOL38862 7.85E 49.81N Trogulus martensi
ZFMKGBOL38864 14.03E 46.0N Trogulus nepaeformis
ZSMARACH00019 9.77E 49.98N ad m Trogulus nepaeformis
ZSMARACH00020 13.71E 47.53N ad m Trogulus nepaeformis
ZFMKTIS21385 5.97E 49.93N ad m Mitostoma chrysomelas
ZFMKTIS21361 6.44E 49.68N ad f Mitostoma chrysomelas
ZFMKTIS21393 6.44E 49.68N ad f Mitostoma chrysomelas
ZFMKTIS2678 8.18E 49.39N ad f Mitostoma chrysomelas
ZFMKTIS7056 10.16E 53.92N Mitostoma chrysomelas
SMNSOpilLFR063 12.20E 47.61N ad m Mitostoma chrysomelas
ZFMKTIS19328 13.19E 50.5N ad f Paranemastoma quadripunctatum
ZFMKTIS2502568 7.21E 49.29N Paranemastoma quadripunctatum
ZFMKTIS2502571 7.21E 49.29N Paranemastoma quadripunctatum
ZFMKTIS2502570 7.21E 49.29N Paranemastoma quadripunctatum
ZFMKTIS21383 7.12E 49.24N ad m Paranemastoma quadripunctatum
ZFMKTIS2502574 7.04E 49.25N Paranemastoma quadripunctatum
ZFMKTIS2502569 7.21E 49.29N Paranemastoma quadripunctatum
ZFMKTIS22707 7.21E 49.29N Paranemastoma quadripunctatum
ZFMKTIS21367 7.12E 49.24N ad f Paranemastoma quadripunctatum
ZFMKTIS2501807 7.44E 49.22N Paranemastoma quadripunctatum
ZSMARACH00012 11.15E 47.51N ad f Paranemastoma quadripunctatum
ZSMARACH00011 11.41E 49.54N ad f Paranemastoma quadripunctatum
ZFMKGBOL38867 8.18E 50.11N Paranemastoma quadripunctatum
ZFMKTIS21406 6.99E 49.25N ad f Paranemastoma quadripunctatum
ZFMKTIS2502567 7.57E 49.14N Paranemastoma quadripunctatum
ZFMKTIS2502573 7.04E 49.25N Paranemastoma quadripunctatum
ZSMARACH00009 13.44E 47.66N ad m Paranemastoma quadripunctatum
ZSMARACH00010 13.16E 48.72N ad m Paranemastoma quadripunctatum
ZSMARACH00002 13.81E 45.38N ad m Nemastoma bidentatum
ZFMKTIS22223 12.92E 50.54N ad m Nemastoma triste
ZFMKTIS2504916 11.29E 47.41N ad m Nemastoma triste
SMNSOpilLFR067 12.82E 47.12N ad f Nemastoma triste
ZSMARACH00008 13.43E 47.78N ad f Nemastoma triste
ZFMKTIS2504953 11.13E 47.42N ad m Nemastoma triste
ZSMARACH00007 13.08E 47.69N ad m Nemastoma triste
ZSMARACH00006 13.16E 48.72N ad f Nemastoma triste
ZFMKTIS21404 5.98E 50.1N ad m Nemastoma bimaculatum
ZFMKTIS22247 12.24E 51.35N ad m Nemastoma dentigerum
ZFMKTIS22246 12.24E 51.35N ad m Nemastoma dentigerum
ZSMARACH00003 11.46E 48.42N ad m Nemastoma dentigerum
ZFMKTIS21373 6.67E 49.46N ad m Nemastoma dentigerum
ZFMKTIS7067 10.68E 54.07N f Nemastoma dentigerum
ZSMARACH00004 9.86E 49.86N ad m Nemastoma dentigerum
SMNSOpilLFR071 9.96E 54.78N ad f Nemastoma lugubre
ZFMKTIS7048 11.05E 54.2N Nemastoma lugubre
ZFMKTIS19334 13.19E 50.5N ad f Nemastoma lugubre
ZFMKTIS2692 13.42E 54.34N ad f Nemastoma lugubre
ZFMKTIS19125 8.88E 54.29N Nemastoma lugubre
ZFMKTIS2492 13.6E 51.18N ad f Nemastoma lugubre
ZFMKTIS19324 13.42E 54.34N ad m Nemastoma lugubre
SMNSOpilLFR070 11.97E 52.46N ad f Nemastoma lugubre
ZFMKTIS2534409 10.23E 53.98N Nemastoma lugubre
SMNSOpilLFR039 12.58E 52.15N ad f Leiobunum rotundum
SMNSOpilLFR081 11.86E 52.49N ad m Leiobunum rotundum
ZFMKTIS19119 9.67E 53.65N f Leiobunum rotundum
ZFMKTIS22242 12.24E 51.35N ad m Astrobunus laevipes
ZFMKTIS2788 12.24E 51.35N ad m Astrobunus laevipes
ZSMGBOL02710 9.23E 48.84N ad m Astrobunus laevipes
ZFMKTIS7086 10.69E 53.87N ad m Leiobunum limbatum
0.5

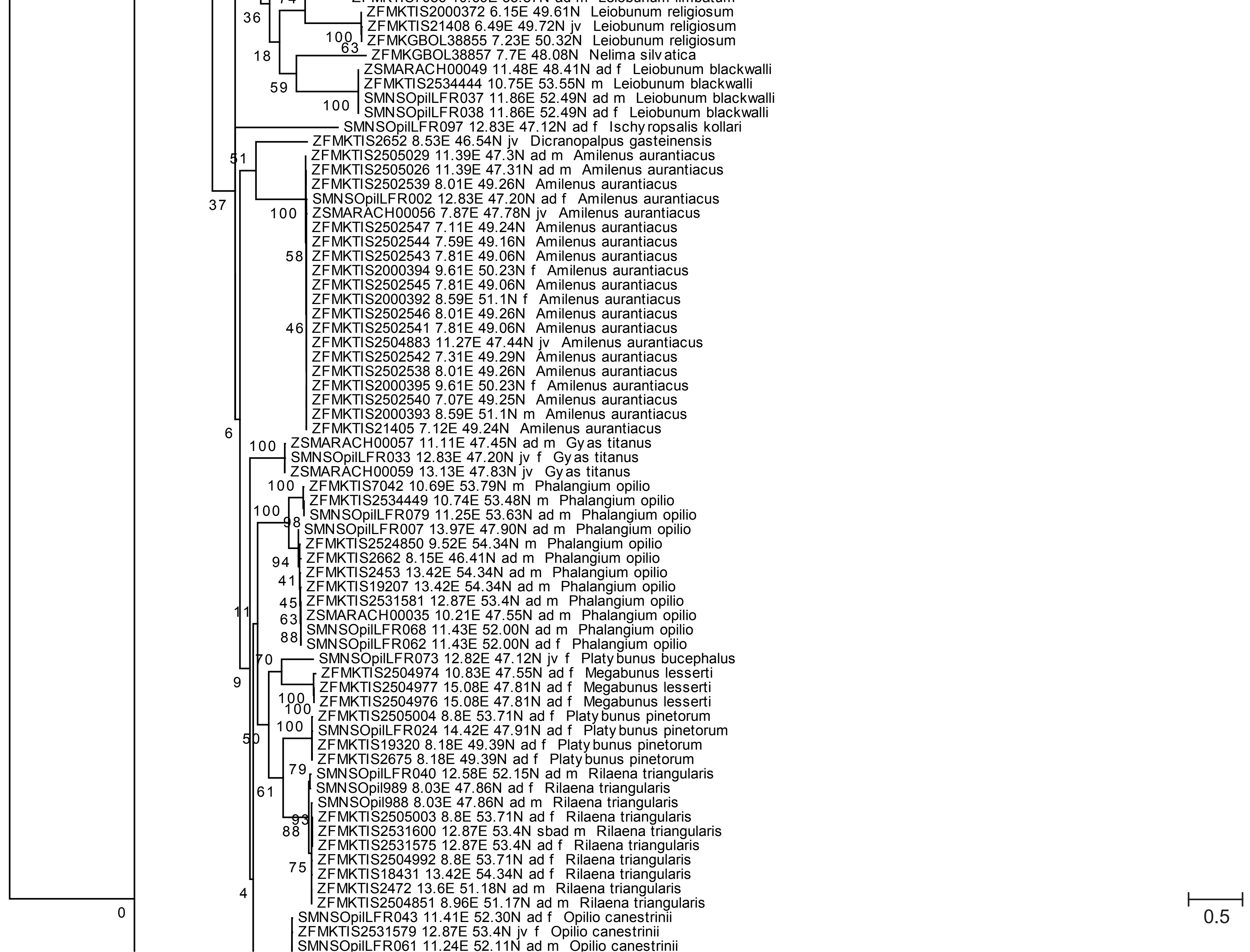
ZFMKTIS2000372 6.15E 49.61N Leiobunum religiosum
ZFMKTIS21408 6.49E 49.72N jv Leiobunum religiosum
ZFMKGBOL38855 7.23E 50.32N Leiobunum religiosum
ZFMKGBOL38857 7.7E 48.08N Nelima silvatica
ZSMARACH00049 11.48E 48.41N ad f Leiobunum blackwalli
ZFMKTIS2534444 10.75E 53.55N m Leiobunum blackwalli
SMNSOpilLFR037 11.86E 52.49N ad m Leiobunum blackwalli
SMNSOpilLFR038 11.86E 52.49N ad f Leiobunum blackwalli
SMNSOpilLFR097 12.83E 47.12N ad f Ischyropsalis kollari
ZFMKTIS2652 8.53E 46.54N jv Dicranopalpus gasteinensis
ZFMKTIS2505029 11.39E 47.3N ad m Amilenus aurantiacus
ZFMKTIS2505026 11.39E 47.31N ad m Amilenus aurantiacus
ZFMKTIS2502539 8.01E 49.26N Amilenus aurantiacus
SMNSOpilLFR002 12.83E 47.20N ad f Amilenus aurantiacus
ZSMARACH00056 7.87E 47.78N jv Amilenus aurantiacus
ZFMKTIS2502547 7.11E 49.24N Amilenus aurantiacus
ZFMKTIS2502544 7.59E 49.16N Amilenus aurantiacus
ZFMKTIS2502543 7.81E 49.06N Amilenus aurantiacus
ZFMKTIS2000394 9.61E 50.23N f Amilenus aurantiacus
ZFMKTIS2502545 7.81E 49.06N Amilenus aurantiacus
ZFMKTIS2000392 8.59E 51.1N f Amilenus aurantiacus
ZFMKTIS2502546 8.01E 49.26N Amilenus aurantiacus
ZFMKTIS2502541 7.81E 49.06N Amilenus aurantiacus
ZFMKTIS2504883 11.27E 47.44N jv Amilenus aurantiacus
ZFMKTIS2502542 7.31E 49.29N Amilenus aurantiacus
ZFMKTIS2502538 8.01E 49.26N Amilenus aurantiacus
ZFMKTIS2000395 9.61E 50.23N f Amilenus aurantiacus
ZFMKTIS2502540 7.07E 49.25N Amilenus aurantiacus
ZFMKTIS2000393 8.59E 51.1N m Amilenus aurantiacus
ZFMKTIS21405 7.12E 49.24N Amilenus aurantiacus
ZSMARACH00057 11.11E 47.45N ad m Gyas titanus
SMNSOpilLFR033 12.83E 47.20N jv f Gyas titanus
ZSMARACH00059 13.13E 47.83N jv Gyas titanus
ZFMKTIS7042 10.69E 53.79N m Phalangium opilio
ZFMKTIS2534449 10.74E 53.48N m Phalangium opilio
SMNSOpilLFR079 11.25E 53.63N ad m Phalangium opilio
SMNSOpilLFR007 13.97E 47.90N ad m Phalangium opilio
ZFMKTIS2524850 9.52E 54.34N m Phalangium opilio
ZFMKTIS2662 8.15E 46.41N ad m Phalangium opilio
ZFMKTIS2453 13.42E 54.34N ad m Phalangium opilio
ZFMKTIS19207 13.42E 54.34N ad m Phalangium opilio
ZFMKTIS2531581 12.87E 53.4N ad m Phalangium opilio
ZSMARACH00035 10.21E 47.55N ad m Phalangium opilio
SMNSOpilLFR068 11.43E 52.00N ad m Phalangium opilio
SMNSOpilLFR062 11.43E 52.00N ad f Phalangium opilio
SMNSOpilLFR073 12.82E 47.12N jv f Platybunus bucephalus
ZFMKTIS2504974 10.83E 47.55N ad f Megabunus lesserti
ZFMKTIS2504977 15.08E 47.81N ad f Megabunus lesserti
ZFMKTIS2504976 15.08E 47.81N ad f Megabunus lesserti
ZFMKTIS2505004 8.8E 53.71N ad f Platybunus pinetorum
SMNSOpilLFR024 14.42E 47.91N ad f Platybunus pinetorum
ZFMKTIS19320 8.18E 49.39N ad f Platybunus pinetorum
ZFMKTIS2675 8.18E 49.39N ad f Platybunus pinetorum
SMNSOpilLFR040 12.58E 52.15N ad m Rilaena triangularis
SMNSOpil989 8.03E 47.86N ad f Rilaena triangularis
SMNSOpil988 8.03E 47.86N ad m Rilaena triangularis
ZFMKTIS2505003 8.8E 53.71N ad f Rilaena triangularis
ZFMKTIS2531600 12.87E 53.4N sbad m Rilaena triangularis
ZFMKTIS2531575 12.87E 53.4N ad f Rilaena triangularis
ZFMKTIS2504992 8.8E 53.71N ad f Rilaena triangularis
ZFMKTIS18431 13.42E 54.34N ad f Rilaena triangularis
ZFMKTIS2472 13.6E 51.18N ad m Rilaena triangularis
ZFMKTIS2504851 8.96E 51.17N ad m Rilaena triangularis
SMNSOpilLFR043 11.41E 52.30N ad f Opilio canestrinii
ZFMKTIS2531579 12.87E 53.4N jv f Opilio canestrinii
SMNSOpilLFR061 11.24E 52.11N ad m Opilio canestrinii
0.5

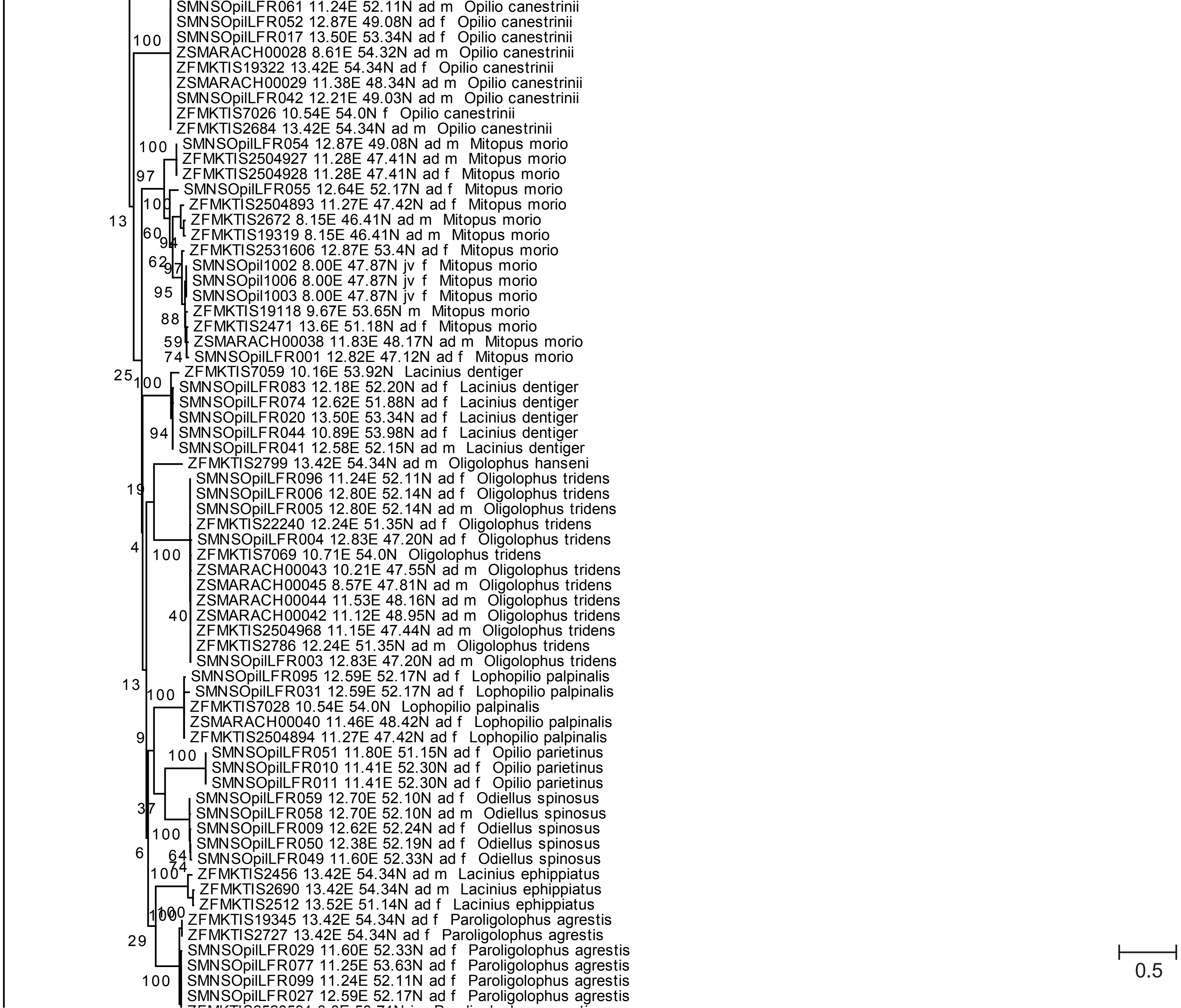

SMNSOpilLFR061 11.24E 52.11N ad m Opilio canestrinii
SMNSOpilLFR052 12.87E 49.08N ad f Opilio canestrinii
SMNSOpilLFR017 13.50E 53.34N ad f Opilio canestrinii
100
ZSMARACH00028 8.61E 54.32N ad m Opilio canestrinii
ZFMKTIS19322 13.42E 54.34N ad f Opilio canestrinii
ZSMARACH00029 11.38E 48.34N ad m Opilio canestrinii
SMNSOpilLFR042 12.21E 49.03N ad m Opilio canestrinii
ZFMKTIS7026 10.54E 54.0N f Opilio canestrinii
ZFMKTIS2684 13.42E 54.34N ad m Opilio canestrinii
100
SMNSOpilLFR054 12.87E 49.08N ad m Mitopus morio
ZFMKTIS2504927 11.28E 47.41N ad m Mitopus morio
97
ZFMKTIS2504928 11.28E 47.41N ad f Mitopus morio
SMNSOpilLFR055 12.64E 52.17N ad f Mitopus morio
100
ZFMKTIS2504893 11.27E 47.42N ad f Mitopus morio
13
ZFMKTIS2672 8.15E 46.41N ad m Mitopus morio
60
94
ZFMKTIS19319 8.15E 46.41N ad m Mitopus morio
ZFMKTIS2531606 12.87E 53.4N ad f Mitopus morio
62
97
SMNSOpil1002 8.00E 47.87N jv f Mitopus morio
SMNSOpil1006 8.00E 47.87N jv f Mitopus morio
95
SMNSOpil1003 8.00E 47.87N jv f Mitopus morio
ZFMKTIS19118 9.67E 53.65N m Mitopus morio
88
ZFMKTIS2471 13.6E 51.18N ad f Mitopus morio
59
ZSMARACH00038 11.83E 48.17N ad m Mitopus morio
74
SMNSOpilLFR001 12.82E 47.12N ad f Mitopus morio
25
ZFMKTIS7059 10.16E 53.92N Lacinius dentiger
100
SMNSOpilLFR083 12.18E 52.20N ad f Lacinius dentiger
SMNSOpilLFR074 12.62E 51.88N ad f Lacinius dentiger
SMNSOpilLFR020 13.50E 53.34N ad f Lacinius dentiger
94
SMNSOpilLFR044 10.89E 53.98N ad f Lacinius dentiger
SMNSOpilLFR041 12.58E 52.15N ad m Lacinius dentiger
ZFMKTIS2799 13.42E 54.34N ad m Oligolophus hanseni
SMNSOpilLFR096 11.24E 52.11N ad f Oligolophus tridens
19
SMNSOpilLFR006 12.80E 52.14N ad f Oligolophus tridens
SMNSOpilLFR005 12.80E 52.14N ad m Oligolophus tridens
ZFMKTIS22240 12.24E 51.35N ad f Oligolophus tridens
SMNSOpilLFR004 12.83E 47.20N ad f Oligolophus tridens
4
100
ZFMKTIS7069 10.71E 54.0N Oligolophus tridens
ZSMARACH00043 10.21E 47.55N ad m Oligolophus tridens
ZSMARACH00045 8.57E 47.81N ad m Oligolophus tridens
ZSMARACH00044 11.53E 48.16N ad m Oligolophus tridens
40
ZSMARACH00042 11.12E 48.95N ad m Oligolophus tridens
ZFMKTIS2504968 11.15E 47.44N ad m Oligolophus tridens
ZFMKTIS2786 12.24E 51.35N ad m Oligolophus tridens
SMNSOpilLFR003 12.83E 47.20N ad m Oligolophus tridens
SMNSOpilLFR095 12.59E 52.17N ad f Lophopilio palpinalis
13
100
SMNSOpilLFR031 12.59E 52.17N ad f Lophopilio palpinalis
ZFMKTIS7028 10.54E 54.0N Lophopilio palpinalis
ZSMARACH00040 11.46E 48.42N ad f Lophopilio palpinalis
9
ZFMKTIS2504894 11.27E 47.42N ad f Lophopilio palpinalis
100
SMNSOpilLFR051 11.80E 51.15N ad f Opilio parietinus
SMNSOpilLFR010 11.41E 52.30N ad f Opilio parietinus
SMNSOpilLFR011 11.41E 52.30N ad f Opilio parietinus
SMNSOpilLFR059 12.70E 52.10N ad f Odiellus spinosus
37
SMNSOpilLFR058 12.70E 52.10N ad m Odiellus spinosus
SMNSOpilLFR009 12.62E 52.24N ad f Odiellus spinosus
100
SMNSOpilLFR050 12.38E 52.19N ad f Odiellus spinosus
6
64
SMNSOpilLFR049 11.60E 52.33N ad f Odiellus spinosus
74
100
ZFMKTIS2456 13.42E 54.34N ad m Lacinius ephippiatus
ZFMKTIS2690 13.42E 54.34N ad m Lacinius ephippiatus
ZFMKTIS2512 13.52E 51.14N ad f Lacinius ephippiatus
100
100
ZFMKTIS19345 13.42E 54.34N ad f Paroligolophus agrestis
ZFMKTIS2727 13.42E 54.34N ad f Paroligolophus agrestis
29
SMNSOpilLFR029 11.60E 52.33N ad f Paroligolophus agrestis
SMNSOpilLFR077 11.25E 53.63N ad f Paroligolophus agrestis
100
SMNSOpilLFR099 11.24E 52.11N ad f Paroligolophus agrestis
SMNSOpilLFR027 12.59E 52.17N ad f Paroligolophus agrestis
0.5

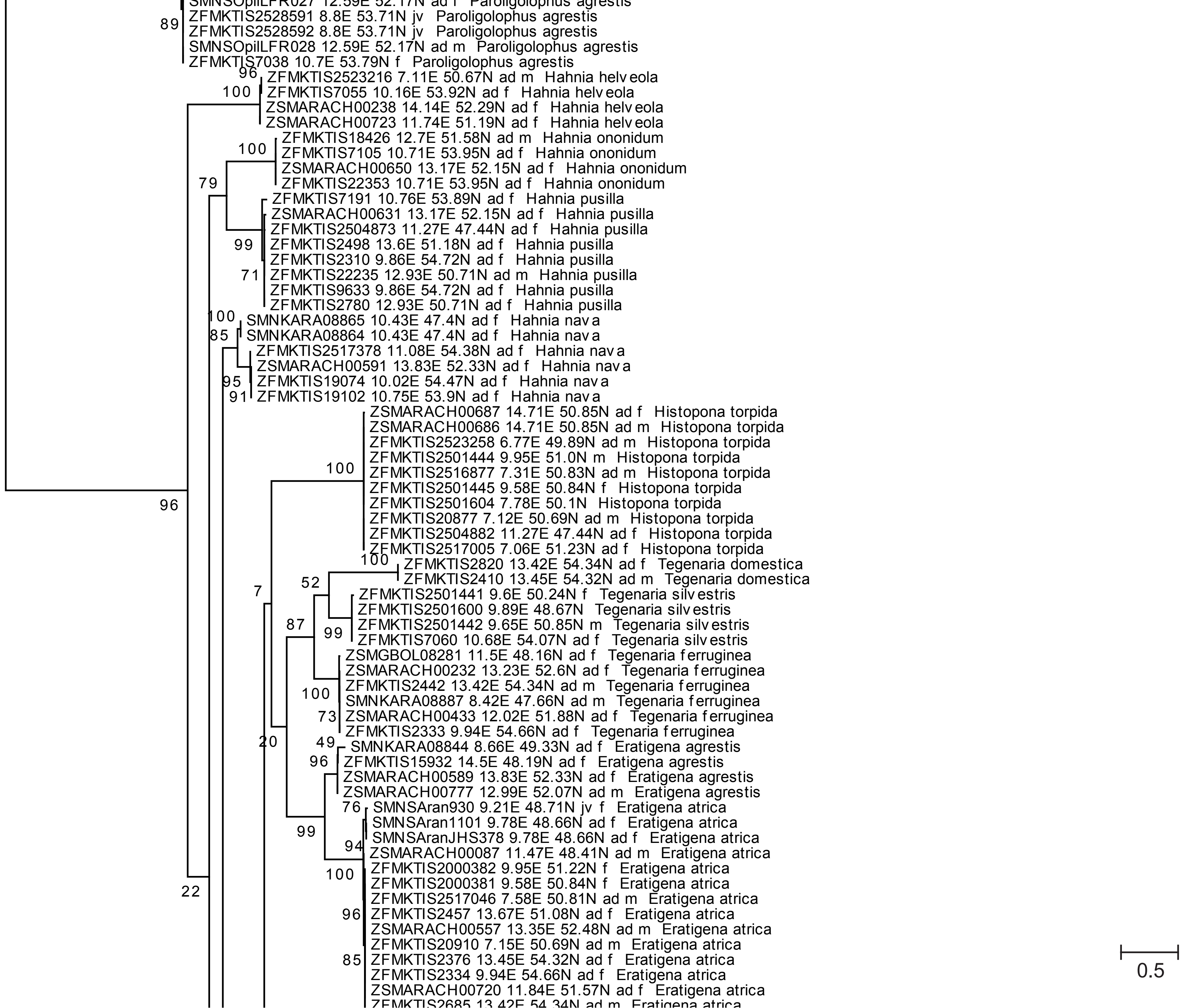

SMNSOpilLFR027 12.59E 52.17N ad f Paroligolophus agrestis
ZFMKTIS2528591 8.8E 53.71N jv Paroligolophus agrestis
ZFMKTIS2528592 8.8E 53.71N jv Paroligolophus agrestis
SMNSOpilLFR028 12.59E 52.17N ad m Paroligolophus agrestis
ZFMKTIS7038 10.7E 53.79N f Paroligolophus agrestis
ZFMKTIS2523216 7.11E 50.67N ad m Hahnia helveola
ZFMKTIS7055 10.16E 53.92N ad f Hahnia helveola
ZSMARACH00238 14.14E 52.29N ad f Hahnia helveola
ZSMARACH00723 11.74E 51.19N ad f Hahnia helveola
ZFMKTIS18426 12.7E 51.58N ad m Hahnia ononidum
ZFMKTIS7105 10.71E 53.95N ad f Hahnia ononidum
ZSMARACH00650 13.17E 52.15N ad f Hahnia ononidum
ZFMKTIS22353 10.71E 53.95N ad f Hahnia ononidum
ZFMKTIS7191 10.76E 53.89N ad f Hahnia pusilla
ZSMARACH00631 13.17E 52.15N ad f Hahnia pusilla
ZFMKTIS2504873 11.27E 47.44N ad f Hahnia pusilla
ZFMKTIS2498 13.6E 51.18N ad f Hahnia pusilla
ZFMKTIS2310 9.86E 54.72N ad f Hahnia pusilla
ZFMKTIS22235 12.93E 50.71N ad m Hahnia pusilla
ZFMKTIS9633 9.86E 54.72N ad f Hahnia pusilla
ZFMKTIS2780 12.93E 50.71N ad f Hahnia pusilla
SMNKARA08865 10.43E 47.4N ad f Hahnia nava
SMNKARA08864 10.43E 47.4N ad f Hahnia nava
ZFMKTIS2517378 11.08E 54.38N ad f Hahnia nava
ZSMARACH00591 13.83E 52.33N ad f Hahnia nava
ZFMKTIS19074 10.02E 54.47N ad f Hahnia nava
ZFMKTIS19102 10.75E 53.9N ad f Hahnia nava
ZSMARACH00687 14.71E 50.85N ad f Histopona torpida
ZSMARACH00686 14.71E 50.85N ad m Histopona torpida
ZFMKTIS2523258 6.77E 49.89N ad m Histopona torpida
ZFMKTIS2501444 9.95E 51.0N m Histopona torpida
ZFMKTIS2516877 7.31E 50.83N ad m Histopona torpida
ZFMKTIS2501445 9.58E 50.84N f Histopona torpida
ZFMKTIS2501604 7.78E 50.1N Histopona torpida
ZFMKTIS20877 7.12E 50.69N ad m Histopona torpida
ZFMKTIS2504882 11.27E 47.44N ad f Histopona torpida
ZFMKTIS2517005 7.06E 51.23N ad f Histopona torpida
ZFMKTIS2820 13.42E 54.34N ad f Tegenaria domestica
ZFMKTIS2410 13.45E 54.32N ad m Tegenaria domestica
ZFMKTIS2501441 9.6E 50.24N f Tegenaria silvestris
ZFMKTIS2501600 9.89E 48.67N Tegenaria silvestris
ZFMKTIS2501442 9.65E 50.85N m Tegenaria silvestris
ZFMKTIS7060 10.68E 54.07N ad f Tegenaria silvestris
ZSMGBOL08281 11.5E 48.16N ad f Tegenaria ferruginea
ZSMARACH00232 13.23E 52.6N ad f Tegenaria ferruginea
ZFMKTIS2442 13.42E 54.34N ad m Tegenaria ferruginea
SMNKARA08887 8.42E 47.66N ad m Tegenaria ferruginea
ZSMARACH00433 12.02E 51.88N ad f Tegenaria ferruginea
ZFMKTIS2333 9.94E 54.66N ad f Tegenaria ferruginea
SMNKARA08844 8.66E 49.33N ad f Eratigena agrestis
ZFMKTIS15932 14.5E 48.19N ad f Eratigena agrestis
ZSMARACH00589 13.83E 52.33N ad f Eratigena agrestis
ZSMARACH00777 12.99E 52.07N ad m Eratigena agrestis
SMNSAran930 9.21E 48.71N jv f Eratigena atrica
SMNSAran1101 9.78E 48.66N ad f Eratigena atrica
SMNSAranJHS378 9.78E 48.66N ad f Eratigena atrica
ZSMARACH00087 11.47E 48.41N ad m Eratigena atrica
ZFMKTIS2000382 9.95E 51.22N f Eratigena atrica
ZFMKTIS2000381 9.58E 50.84N f Eratigena atrica
ZFMKTIS2517046 7.58E 50.81N ad m Eratigena atrica
ZFMKTIS2457 13.67E 51.08N ad f Eratigena atrica
ZSMARACH00557 13.35E 52.48N ad m Eratigena atrica
ZFMKTIS20910 7.15E 50.69N ad m Eratigena atrica
ZFMKTIS2376 13.45E 54.32N ad f Eratigena atrica
ZFMKTIS2334 9.94E 54.66N ad f Eratigena atrica
ZSMARACH00720 11.84E 51.57N ad f Eratigena atrica
0.5

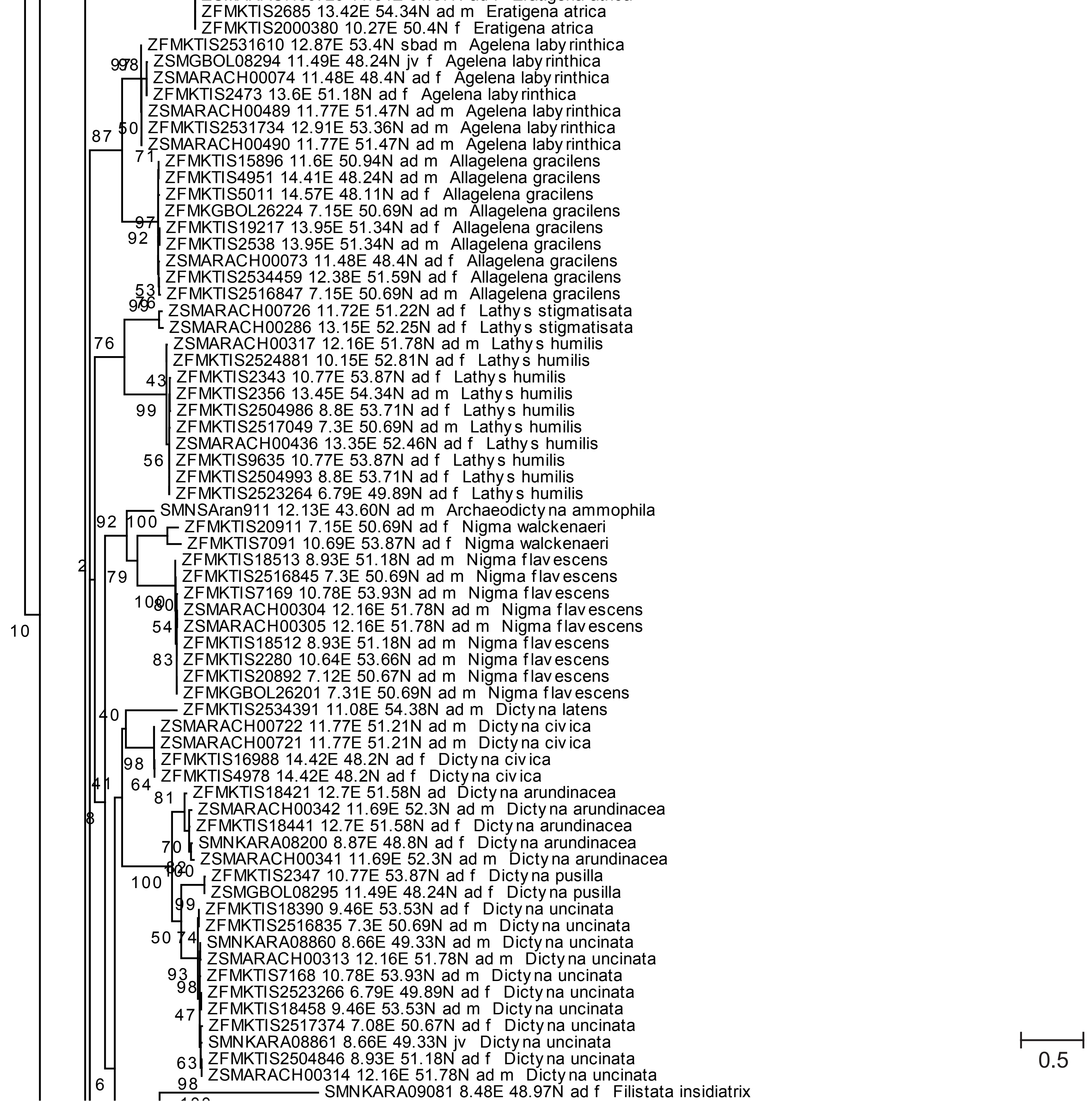
ZFMKTIS2685 13.42E 54.34N ad m Eratigena atrica
ZFMKTIS2000380 10.27E 50.4N f Eratigena atrica
ZFMKTIS2531610 12.87E 53.4N sbad m Agelena labyrinthica
ZSMGBOL08294 11.49E 48.24N jv f Agelena labyrinthica
ZSMARACH00074 11.48E 48.4N ad f Agelena labyrinthica
ZFMKTIS2473 13.6E 51.18N ad f Agelena labyrinthica
ZSMARACH00489 11.77E 51.47N ad m Agelena labyrinthica
ZFMKTIS2531734 12.91E 53.36N ad m Agelena labyrinthica
ZSMARACH00490 11.77E 51.47N ad m Agelena labyrinthica
ZFMKTIS15896 11.6E 50.94N ad m Allagelena gracilens
ZFMKTIS4951 14.41E 48.24N ad m Allagelena gracilens
ZFMKTIS5011 14.57E 48.11N ad f Allagelena gracilens
ZFMKGBOL26224 7.15E 50.69N ad m Allagelena gracilens
ZFMKTIS19217 13.95E 51.34N ad f Allagelena gracilens
ZFMKTIS2538 13.95E 51.34N ad m Allagelena gracilens
ZSMARACH00073 11.48E 48.4N ad f Allagelena gracilens
ZFMKTIS2534459 12.38E 51.59N ad f Allagelena gracilens
ZFMKTIS2516847 7.15E 50.69N ad m Allagelena gracilens
ZSMARACH00726 11.72E 51.22N ad f Lathys stigmatisata
ZSMARACH00286 13.15E 52.25N ad f Lathys stigmatisata
ZSMARACH00317 12.16E 51.78N ad m Lathys humilis
ZFMKTIS2524881 10.15E 52.81N ad f Lathys humilis
ZFMKTIS2343 10.77E 53.87N ad f Lathys humilis
ZFMKTIS2356 13.45E 54.34N ad m Lathys humilis
ZFMKTIS2504986 8.8E 53.71N ad f Lathys humilis
ZFMKTIS2517049 7.3E 50.69N ad m Lathys humilis
ZSMARACH00436 13.35E 52.46N ad f Lathys humilis
ZFMKTIS9635 10.77E 53.87N ad f Lathys humilis
ZFMKTIS2504993 8.8E 53.71N ad f Lathys humilis
ZFMKTIS2523264 6.79E 49.89N ad f Lathys humilis
SMNSAran911 12.13E 43.60N ad m Archaeodictyna ammophila
ZFMKTIS20911 7.15E 50.69N ad f Nigma walckenaeri
ZFMKTIS7091 10.69E 53.87N ad f Nigma walckenaeri
ZFMKTIS18513 8.93E 51.18N ad m Nigma flavescens
ZFMKTIS2516845 7.3E 50.69N ad m Nigma flavescens
ZFMKTIS7169 10.78E 53.93N ad m Nigma flavescens
ZSMARACH00304 12.16E 51.78N ad m Nigma flavescens
ZSMARACH00305 12.16E 51.78N ad m Nigma flavescens
ZFMKTIS18512 8.93E 51.18N ad m Nigma flavescens
ZFMKTIS2280 10.64E 53.66N ad m Nigma flavescens
ZFMKTIS20892 7.12E 50.67N ad m Nigma flavescens
ZFMKGBOL26201 7.31E 50.69N ad m Nigma flavescens
ZFMKTIS2534391 11.08E 54.38N ad m Dictyna latens
ZSMARACH00722 11.77E 51.21N ad m Dictyna civica
ZSMARACH00721 11.77E 51.21N ad m Dictyna civica
ZFMKTIS16988 14.42E 48.2N ad f Dictyna civica
ZFMKTIS4978 14.42E 48.2N ad f Dictyna civica
ZFMKTIS18421 12.7E 51.58N ad Dictyna arundinacea
ZSMARACH00342 11.69E 52.3N ad m Dictyna arundinacea
ZFMKTIS18441 12.7E 51.58N ad f Dictyna arundinacea
SMNKARA08200 8.87E 48.8N ad f Dictyna arundinacea
ZSMARACH00341 11.69E 52.3N ad m Dictyna arundinacea
ZFMKTIS2347 10.77E 53.87N ad f Dictyna pusilla
ZSMGBOL08295 11.49E 48.24N ad f Dictyna pusilla
ZFMKTIS18390 9.46E 53.53N ad f Dictyna uncinata
ZFMKTIS2516835 7.3E 50.69N ad m Dictyna uncinata
SMNKARA08860 8.66E 49.33N ad m Dictyna uncinata
ZSMARACH00313 12.16E 51.78N ad m Dictyna uncinata
ZFMKTIS7168 10.78E 53.93N ad m Dictyna uncinata
ZFMKTIS2523266 6.79E 49.89N ad f Dictyna uncinata
ZFMKTIS18458 9.46E 53.53N ad m Dictyna uncinata
ZFMKTIS2517374 7.08E 50.67N ad f Dictyna uncinata
SMNKARA08861 8.66E 49.33N jv Dictyna uncinata
ZFMKTIS2504846 8.93E 51.18N ad f Dictyna uncinata
ZSMARACH00314 12.16E 51.78N ad m Dictyna uncinata
SMNKARA09081 8.48E 48.97N ad f Filistata insidiatrix
0.5

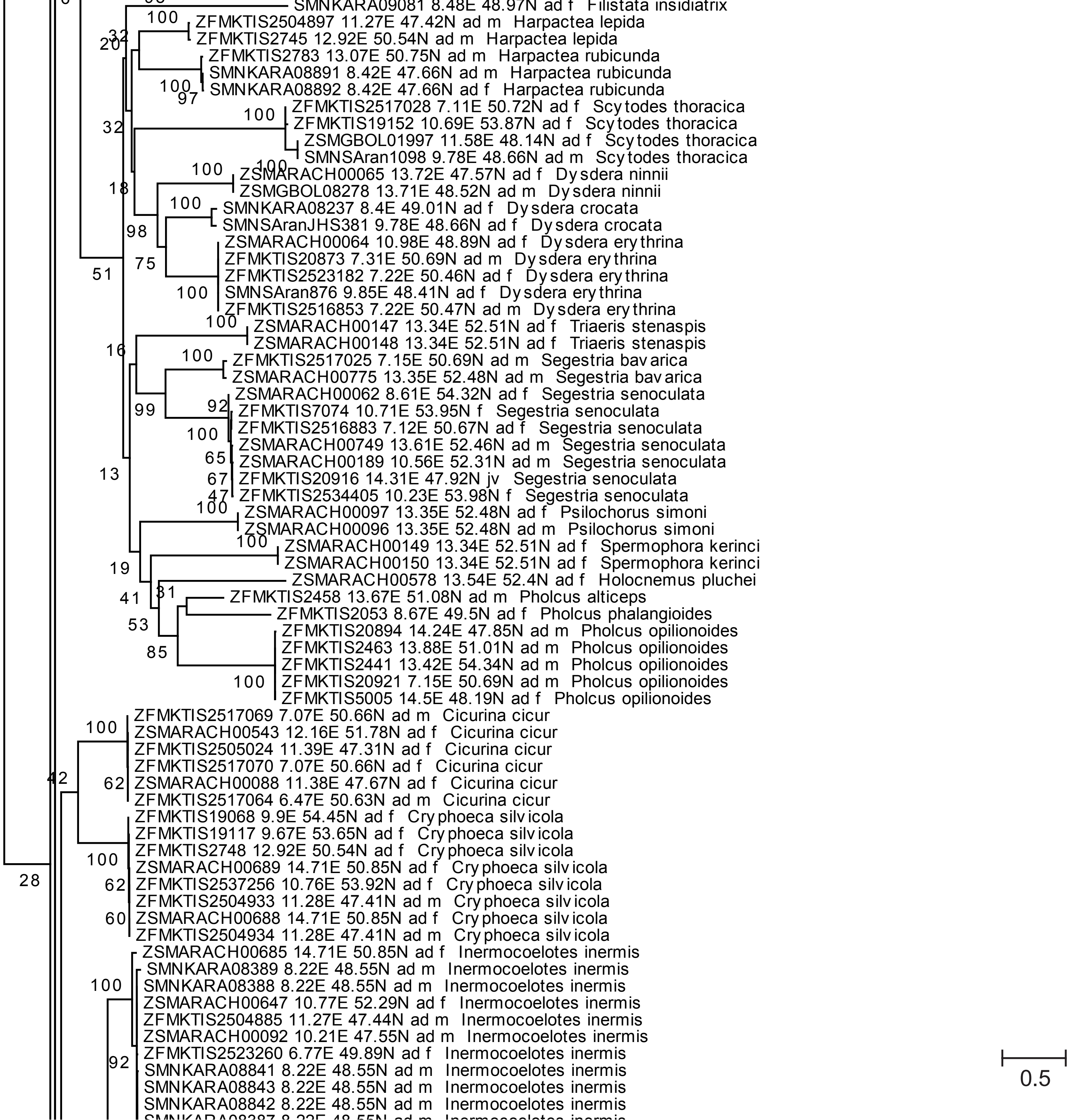
SMNKARA09081 8.48E 48.97N ad f Filistata insidiatrix
ZFMKTIS2504897 11.27E 47.42N ad m Harpactea lepida
ZFMKTIS2745 12.92E 50.54N ad m Harpactea lepida
ZFMKTIS2783 13.07E 50.75N ad m Harpactea rubicunda
SMNKARA08891 8.42E 47.66N ad m Harpactea rubicunda
SMNKARA08892 8.42E 47.66N ad f Harpactea rubicunda
ZFMKTIS2517028 7.11E 50.72N ad f Scytodes thoracica
ZFMKTIS19152 10.69E 53.87N ad f Scytodes thoracica
ZSMGBOL01997 11.58E 48.14N ad f Scytodes thoracica
SMNSAran1098 9.78E 48.66N ad m Scytodes thoracica
ZSMARACH00065 13.72E 47.57N ad f Dysdera ninnii
ZSMGBOL08278 13.71E 48.52N ad m Dysdera ninnii
SMNKARA08237 8.4E 49.01N ad f Dysdera crocata
SMNSAranJHS381 9.78E 48.66N ad f Dysdera crocata
ZSMARACH00064 10.98E 48.89N ad f Dysdera erythrina
ZFMKTIS20873 7.31E 50.69N ad m Dysdera erythrina
ZFMKTIS2523182 7.22E 50.46N ad f Dysdera erythrina
SMNSAran876 9.85E 48.41N ad f Dysdera erythrina
ZFMKTIS2516853 7.22E 50.47N ad m Dysdera erythrina
ZSMARACH00147 13.34E 52.51N ad f Triaeris stenaspis
ZSMARACH00148 13.34E 52.51N ad f Triaeris stenaspis
ZFMKTIS2517025 7.15E 50.69N ad m Segestria bavarica
ZSMARACH00775 13.35E 52.48N ad m Segestria bavarica
ZSMARACH00062 8.61E 54.32N ad f Segestria senoculata
ZFMKTIS7074 10.71E 53.95N f Segestria senoculata
ZFMKTIS2516883 7.12E 50.67N ad f Segestria senoculata
ZSMARACH00749 13.61E 52.46N ad m Segestria senoculata
ZSMARACH00189 10.56E 52.31N ad m Segestria senoculata
ZFMKTIS20916 14.31E 47.92N jv Segestria senoculata
ZFMKTIS2534405 10.23E 53.98N f Segestria senoculata
ZSMARACH00097 13.35E 52.48N ad f Psilochorus simoni
ZSMARACH00096 13.35E 52.48N ad m Psilochorus simoni
ZSMARACH00149 13.34E 52.51N ad f Spermophora kerinci
ZSMARACH00150 13.34E 52.51N ad f Spermophora kerinci
ZSMARACH00578 13.54E 52.4N ad f Holocnemus pluchei
ZFMKTIS2458 13.67E 51.08N ad m Pholcus alticeps
ZFMKTIS2053 8.67E 49.5N ad f Pholcus phalangioides
ZFMKTIS20894 14.24E 47.85N ad m Pholcus opilionoides
ZFMKTIS2463 13.88E 51.01N ad m Pholcus opilionoides
ZFMKTIS2441 13.42E 54.34N ad m Pholcus opilionoides
ZFMKTIS20921 7.15E 50.69N ad m Pholcus opilionoides
ZFMKTIS5005 14.5E 48.19N ad f Pholcus opilionoides
ZFMKTIS2517069 7.07E 50.66N ad m Cicurina cicur
ZSMARACH00543 12.16E 51.78N ad f Cicurina cicur
ZFMKTIS2505024 11.39E 47.31N ad f Cicurina cicur
ZFMKTIS2517070 7.07E 50.66N ad f Cicurina cicur
ZSMARACH00088 11.38E 47.67N ad f Cicurina cicur
ZFMKTIS2517064 6.47E 50.63N ad m Cicurina cicur
ZFMKTIS19068 9.9E 54.45N ad f Cryphoeca silvicola
ZFMKTIS19117 9.67E 53.65N ad f Cryphoeca silvicola
ZFMKTIS2748 12.92E 50.54N ad f Cryphoeca silvicola
ZSMARACH00689 14.71E 50.85N ad f Cryphoeca silvicola
ZFMKTIS2537256 10.76E 53.92N ad f Cryphoeca silvicola
ZFMKTIS2504933 11.28E 47.41N ad m Cryphoeca silvicola
ZSMARACH00688 14.71E 50.85N ad f Cryphoeca silvicola
ZFMKTIS2504934 11.28E 47.41N ad m Cryphoeca silvicola
ZSMARACH00685 14.71E 50.85N ad f Inermocoelotes inermis
SMNKARA08389 8.22E 48.55N ad m Inermocoelotes inermis
SMNKARA08388 8.22E 48.55N ad m Inermocoelotes inermis
ZSMARACH00647 10.77E 52.29N ad f Inermocoelotes inermis
ZFMKTIS2504885 11.27E 47.44N ad m Inermocoelotes inermis
ZSMARACH00092 10.21E 47.55N ad m Inermocoelotes inermis
ZFMKTIS2523260 6.77E 49.89N ad f Inermocoelotes inermis
SMNKARA08841 8.22E 48.55N ad m Inermocoelotes inermis
SMNKARA08843 8.22E 48.55N ad m Inermocoelotes inermis
SMNKARA08842 8.22E 48.55N ad m Inermocoelotes inermis
0.5

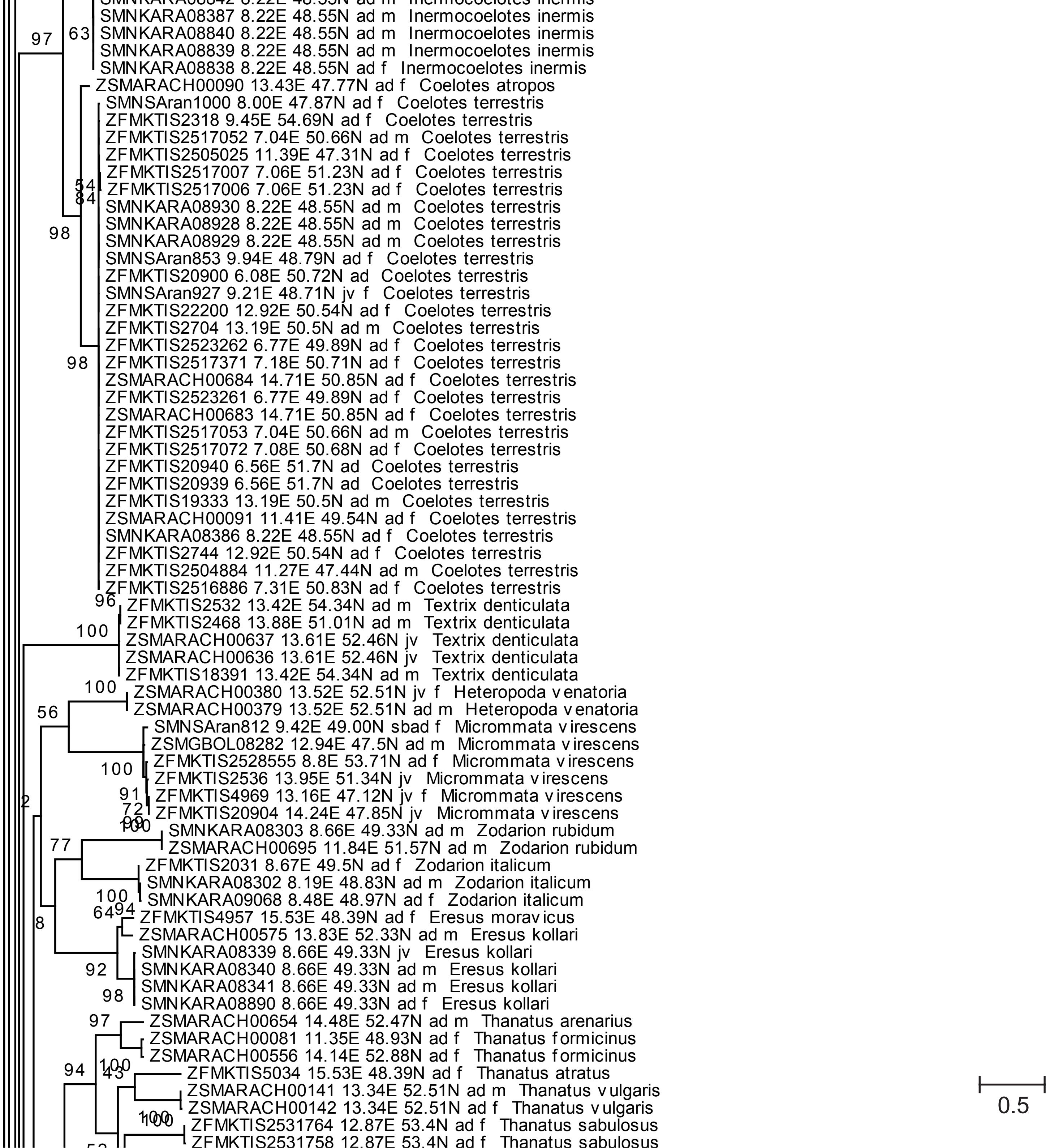

SMNKARA08387 8.22E 48.55N ad m Inermocoelotes inermis
SMNKARA08840 8.22E 48.55N ad m Inermocoelotes inermis
SMNKARA08839 8.22E 48.55N ad m Inermocoelotes inermis
SMNKARA08838 8.22E 48.55N ad f Inermocoelotes inermis
ZSMARACH00090 13.43E 47.77N ad f Coelotes atropos
SMNSAran1000 8.00E 47.87N ad f Coelotes terrestris
ZFMKTIS2318 9.45E 54.69N ad f Coelotes terrestris
ZFMKTIS2517052 7.04E 50.66N ad m Coelotes terrestris
ZFMKTIS2505025 11.39E 47.31N ad f Coelotes terrestris
ZFMKTIS2517007 7.06E 51.23N ad f Coelotes terrestris
ZFMKTIS2517006 7.06E 51.23N ad f Coelotes terrestris
SMNKARA08930 8.22E 48.55N ad m Coelotes terrestris
SMNKARA08928 8.22E 48.55N ad m Coelotes terrestris
SMNKARA08929 8.22E 48.55N ad m Coelotes terrestris
SMNSAran853 9.94E 48.79N ad f Coelotes terrestris
ZFMKTIS20900 6.08E 50.72N ad Coelotes terrestris
SMNSAran927 9.21E 48.71N jv f Coelotes terrestris
ZFMKTIS22200 12.92E 50.54N ad f Coelotes terrestris
ZFMKTIS2704 13.19E 50.5N ad m Coelotes terrestris
ZFMKTIS2523262 6.77E 49.89N ad f Coelotes terrestris
ZFMKTIS2517371 7.18E 50.71N ad f Coelotes terrestris
ZSMARACH00684 14.71E 50.85N ad f Coelotes terrestris
ZFMKTIS2523261 6.77E 49.89N ad f Coelotes terrestris
ZSMARACH00683 14.71E 50.85N ad f Coelotes terrestris
ZFMKTIS2517053 7.04E 50.66N ad m Coelotes terrestris
ZFMKTIS2517072 7.08E 50.68N ad f Coelotes terrestris
ZFMKTIS20940 6.56E 51.7N ad Coelotes terrestris
ZFMKTIS20939 6.56E 51.7N ad Coelotes terrestris
ZFMKTIS19333 13.19E 50.5N ad m Coelotes terrestris
ZSMARACH00091 11.41E 49.54N ad f Coelotes terrestris
SMNKARA08386 8.22E 48.55N ad f Coelotes terrestris
ZFMKTIS2744 12.92E 50.54N ad f Coelotes terrestris
ZFMKTIS2504884 11.27E 47.44N ad m Coelotes terrestris
ZFMKTIS2516886 7.31E 50.83N ad f Coelotes terrestris
ZFMKTIS2532 13.42E 54.34N ad m Textrix denticulata
ZFMKTIS2468 13.88E 51.01N ad m Textrix denticulata
ZSMARACH00637 13.61E 52.46N jv Textrix denticulata
ZSMARACH00636 13.61E 52.46N jv Textrix denticulata
ZFMKTIS18391 13.42E 54.34N ad m Textrix denticulata
ZSMARACH00380 13.52E 52.51N jv f Heteropoda venatoria
ZSMARACH00379 13.52E 52.51N ad m Heteropoda venatoria
SMNSAran812 9.42E 49.00N sbad f Micrommata virescens
ZSMGBOL08282 12.94E 47.5N ad m Micrommata virescens
ZFMKTIS2528555 8.8E 53.71N ad f Micrommata virescens
ZFMKTIS2536 13.95E 51.34N jv Micrommata virescens
ZFMKTIS4969 13.16E 47.12N jv f Micrommata virescens
ZFMKTIS20904 14.24E 47.85N jv Micrommata virescens
SMNKARA08303 8.66E 49.33N ad m Zodarion rubidum
ZSMARACH00695 11.84E 51.57N ad m Zodarion rubidum
ZFMKTIS2031 8.67E 49.5N ad f Zodarion italicum
SMNKARA08302 8.19E 48.83N ad m Zodarion italicum
SMNKARA09068 8.48E 48.97N ad f Zodarion italicum
ZFMKTIS4957 15.53E 48.39N ad f Eresus moravicus
ZSMARACH00575 13.83E 52.33N ad m Eresus kollari
SMNKARA08339 8.66E 49.33N jv Eresus kollari
SMNKARA08340 8.66E 49.33N ad m Eresus kollari
SMNKARA08341 8.66E 49.33N ad m Eresus kollari
SMNKARA08890 8.66E 49.33N ad f Eresus kollari
ZSMARACH00654 14.48E 52.47N ad m Thanatus arenarius
ZSMARACH00081 11.35E 48.93N ad f Thanatus formicinus
ZSMARACH00556 14.14E 52.88N ad f Thanatus formicinus
ZFMKTIS5034 15.53E 48.39N ad f Thanatus atratus
ZSMARACH00141 13.34E 52.51N ad m Thanatus vulgaris
ZSMARACH00142 13.34E 52.51N ad f Thanatus vulgaris
ZFMKTIS2531764 12.87E 53.4N ad f Thanatus sabulosus
ZFMKTIS2531758 12.87E 53.4N ad f Thanatus sabulosus
97
63
98
54
84
98
96
100
100
56
100
91
72
77
100
64
94
92
98
97
94
43
100
0.5

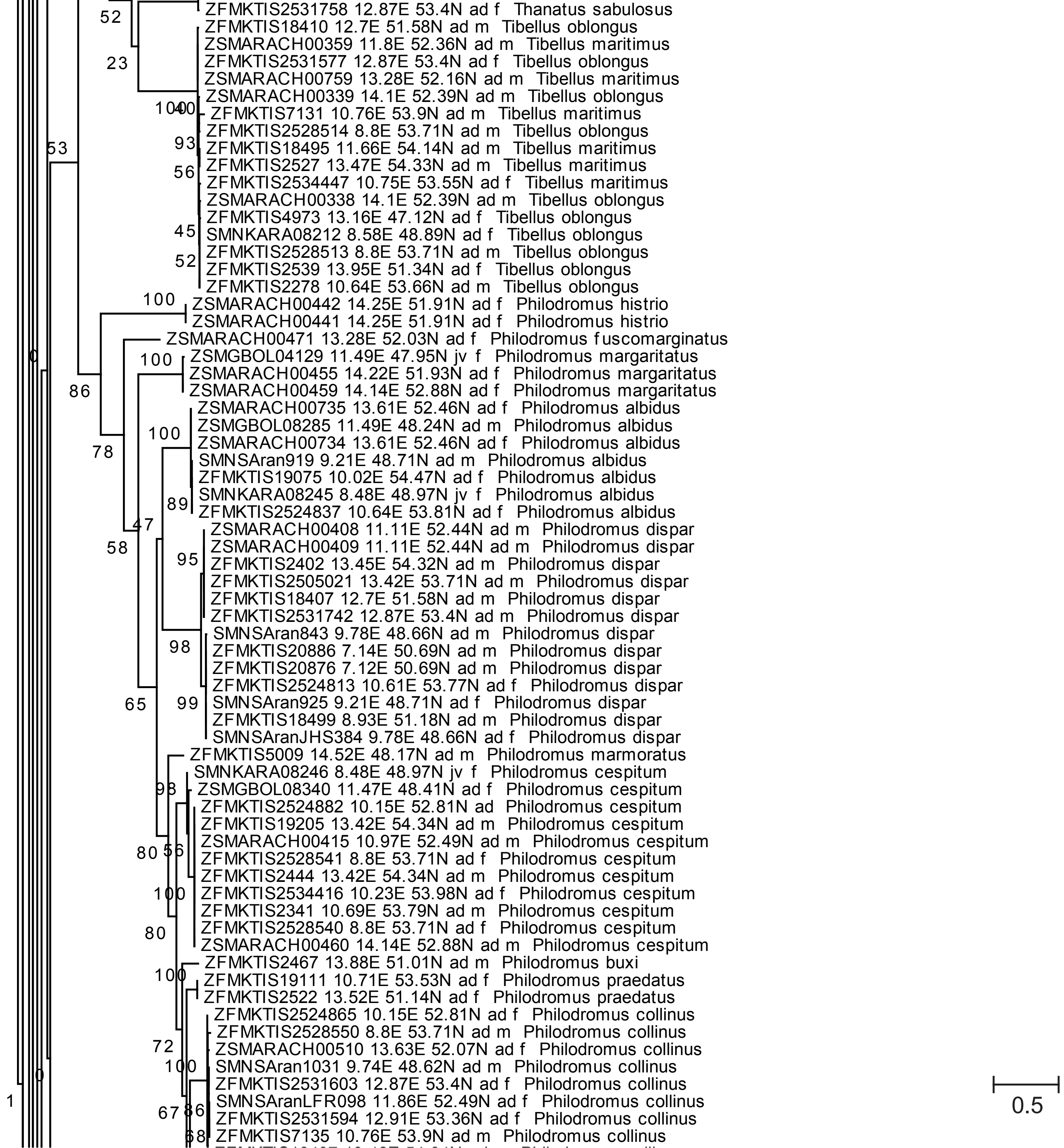
ZFMKTIS2531758 12.87E 53.4N ad f Thanatus sabulosus
ZFMKTIS18410 12.7E 51.58N ad m Tibellus oblongus
ZSMARACH00359 11.8E 52.36N ad m Tibellus maritimus
ZFMKTIS2531577 12.87E 53.4N ad f Tibellus oblongus
ZSMARACH00759 13.28E 52.16N ad m Tibellus maritimus
ZSMARACH00339 14.1E 52.39N ad m Tibellus oblongus
ZFMKTIS7131 10.76E 53.9N ad m Tibellus maritimus
ZFMKTIS2528514 8.8E 53.71N ad m Tibellus oblongus
ZFMKTIS18495 11.66E 54.14N ad m Tibellus maritimus
ZFMKTIS2527 13.47E 54.33N ad m Tibellus maritimus
ZFMKTIS2534447 10.75E 53.55N ad f Tibellus maritimus
ZSMARACH00338 14.1E 52.39N ad m Tibellus oblongus
ZFMKTIS4973 13.16E 47.12N ad f Tibellus oblongus
SMNKARA08212 8.58E 48.89N ad f Tibellus oblongus
ZFMKTIS2528513 8.8E 53.71N ad m Tibellus oblongus
ZFMKTIS2539 13.95E 51.34N ad f Tibellus oblongus
ZFMKTIS2278 10.64E 53.66N ad m Tibellus oblongus
ZSMARACH00442 14.25E 51.91N ad f Philodromus histrio
ZSMARACH00441 14.25E 51.91N ad f Philodromus histrio
ZSMARACH00471 13.28E 52.03N ad f Philodromus fuscomarginatus
ZSMGBOL04129 11.49E 47.95N jv f Philodromus margaritatus
ZSMARACH00455 14.22E 51.93N ad f Philodromus margaritatus
ZSMARACH00459 14.14E 52.88N ad f Philodromus margaritatus
ZSMARACH00735 13.61E 52.46N ad f Philodromus albidus
ZSMGBOL08285 11.49E 48.24N ad m Philodromus albidus
ZSMARACH00734 13.61E 52.46N ad f Philodromus albidus
SMNSAran919 9.21E 48.71N ad m Philodromus albidus
ZFMKTIS19075 10.02E 54.47N ad f Philodromus albidus
SMNKARA08245 8.48E 48.97N jv f Philodromus albidus
ZFMKTIS2524837 10.64E 53.81N ad f Philodromus albidus
ZSMARACH00408 11.11E 52.44N ad m Philodromus dispar
ZSMARACH00409 11.11E 52.44N ad m Philodromus dispar
ZFMKTIS2402 13.45E 54.32N ad m Philodromus dispar
ZFMKTIS2505021 13.42E 53.71N ad m Philodromus dispar
ZFMKTIS18407 12.7E 51.58N ad m Philodromus dispar
ZFMKTIS2531742 12.87E 53.4N ad m Philodromus dispar
SMNSAran843 9.78E 48.66N ad m Philodromus dispar
ZFMKTIS20886 7.14E 50.69N ad m Philodromus dispar
ZFMKTIS20876 7.12E 50.69N ad m Philodromus dispar
ZFMKTIS2524813 10.61E 53.77N ad f Philodromus dispar
SMNSAran925 9.21E 48.71N ad f Philodromus dispar
ZFMKTIS18499 8.93E 51.18N ad m Philodromus dispar
SMNSAranJHS384 9.78E 48.66N ad f Philodromus dispar
ZFMKTIS5009 14.52E 48.17N ad m Philodromus marmoratus
SMNKARA08246 8.48E 48.97N jv f Philodromus cespitum
ZSMGBOL08340 11.47E 48.41N ad f Philodromus cespitum
ZFMKTIS2524882 10.15E 52.81N ad Philodromus cespitum
ZFMKTIS19205 13.42E 54.34N ad m Philodromus cespitum
ZSMARACH00415 10.97E 52.49N ad m Philodromus cespitum
ZFMKTIS2528541 8.8E 53.71N ad f Philodromus cespitum
ZFMKTIS2444 13.42E 54.34N ad m Philodromus cespitum
ZFMKTIS2534416 10.23E 53.98N ad f Philodromus cespitum
ZFMKTIS2341 10.69E 53.79N ad m Philodromus cespitum
ZFMKTIS2528540 8.8E 53.71N ad f Philodromus cespitum
ZSMARACH00460 14.14E 52.88N ad m Philodromus cespitum
ZFMKTIS2467 13.88E 51.01N ad m Philodromus buxi
ZFMKTIS19111 10.71E 53.53N ad f Philodromus praedatus
ZFMKTIS2522 13.52E 51.14N ad f Philodromus praedatus
ZFMKTIS2524865 10.15E 52.81N ad f Philodromus collinus
ZFMKTIS2528550 8.8E 53.71N ad m Philodromus collinus
ZSMARACH00510 13.63E 52.07N ad f Philodromus collinus
SMNSAran1031 9.74E 48.62N ad m Philodromus collinus
ZFMKTIS2531603 12.87E 53.4N ad f Philodromus collinus
SMNSAranLFR098 11.86E 52.49N ad f Philodromus collinus
ZFMKTIS2531594 12.91E 53.36N ad f Philodromus collinus
ZFMKTIS7135 10.76E 53.9N ad m Philodromus collinus
0.5

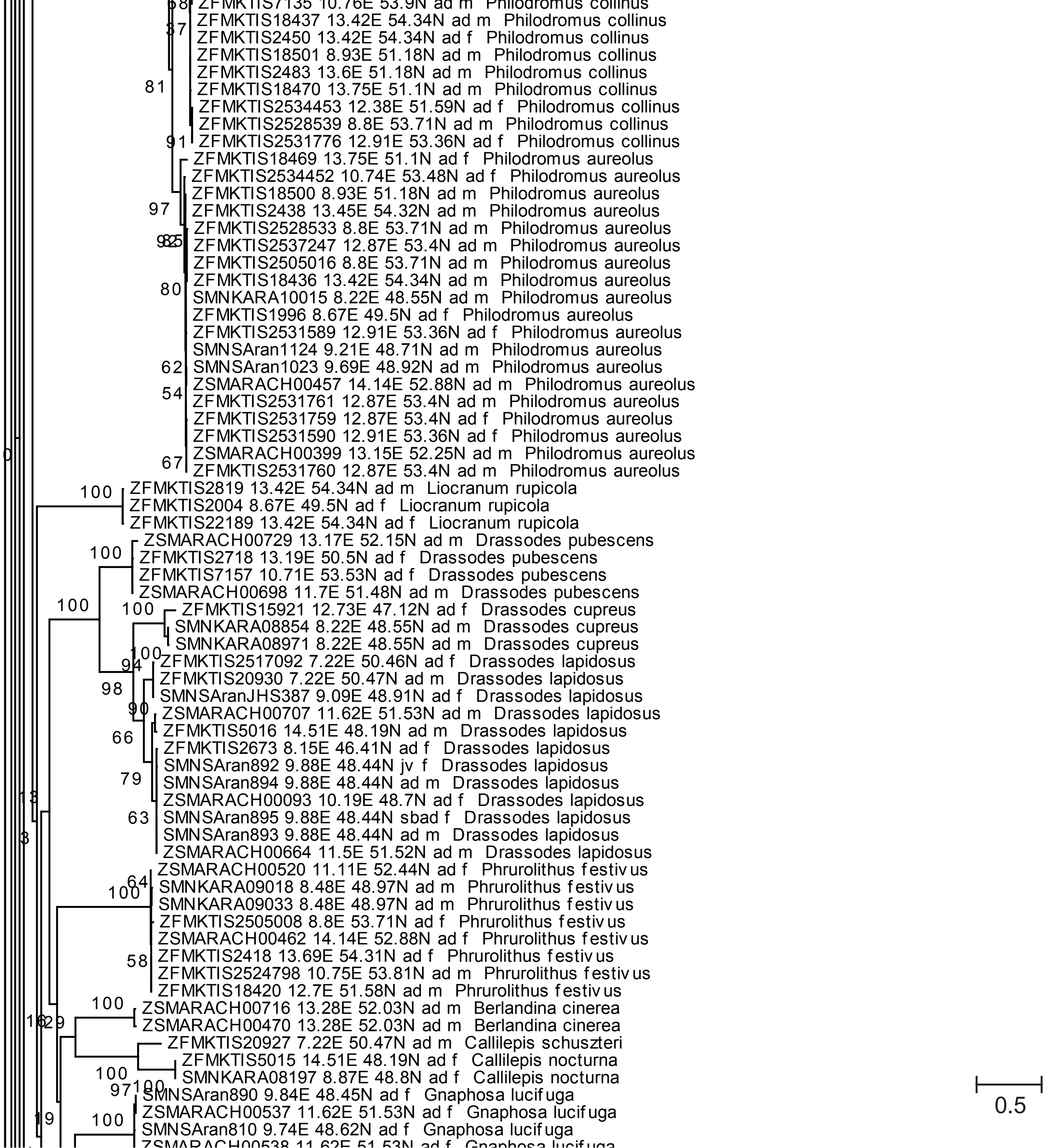
ZFMKTIS7135 10.76E 53.9N ad m Philodromus collinus
ZFMKTIS18437 13.42E 54.34N ad m Philodromus collinus
ZFMKTIS2450 13.42E 54.34N ad f Philodromus collinus
ZFMKTIS18501 8.93E 51.18N ad m Philodromus collinus
ZFMKTIS2483 13.6E 51.18N ad m Philodromus collinus
ZFMKTIS18470 13.75E 51.1N ad m Philodromus collinus
ZFMKTIS2534453 12.38E 51.59N ad f Philodromus collinus
ZFMKTIS2528539 8.8E 53.71N ad m Philodromus collinus
ZFMKTIS2531776 12.91E 53.36N ad f Philodromus collinus
ZFMKTIS18469 13.75E 51.1N ad f Philodromus aureolus
ZFMKTIS2534452 10.74E 53.48N ad f Philodromus aureolus
ZFMKTIS18500 8.93E 51.18N ad m Philodromus aureolus
ZFMKTIS2438 13.45E 54.32N ad m Philodromus aureolus
ZFMKTIS2528533 8.8E 53.71N ad m Philodromus aureolus
ZFMKTIS2537247 12.87E 53.4N ad m Philodromus aureolus
ZFMKTIS2505016 8.8E 53.71N ad m Philodromus aureolus
ZFMKTIS18436 13.42E 54.34N ad m Philodromus aureolus
SMNKARA10015 8.22E 48.55N ad m Philodromus aureolus
ZFMKTIS1996 8.67E 49.5N ad f Philodromus aureolus
ZFMKTIS2531589 12.91E 53.36N ad f Philodromus aureolus
SMNSAran1124 9.21E 48.71N ad m Philodromus aureolus
SMNSAran1023 9.69E 48.92N ad m Philodromus aureolus
ZSMARACH00457 14.14E 52.88N ad m Philodromus aureolus
ZFMKTIS2531761 12.87E 53.4N ad m Philodromus aureolus
ZFMKTIS2531759 12.87E 53.4N ad f Philodromus aureolus
ZFMKTIS2531590 12.91E 53.36N ad f Philodromus aureolus
ZSMARACH00399 13.15E 52.25N ad m Philodromus aureolus
ZFMKTIS2531760 12.87E 53.4N ad m Philodromus aureolus
ZFMKTIS2819 13.42E 54.34N ad m Liocranum rupicola
ZFMKTIS2004 8.67E 49.5N ad f Liocranum rupicola
ZFMKTIS22189 13.42E 54.34N ad f Liocranum rupicola
ZSMARACH00729 13.17E 52.15N ad m Drassodes pubescens
ZFMKTIS2718 13.19E 50.5N ad f Drassodes pubescens
ZFMKTIS7157 10.71E 53.53N ad f Drassodes pubescens
ZSMARACH00698 11.7E 51.48N ad m Drassodes pubescens
ZFMKTIS15921 12.73E 47.12N ad f Drassodes cupreus
SMNKARA08854 8.22E 48.55N ad m Drassodes cupreus
SMNKARA08971 8.22E 48.55N ad m Drassodes cupreus
ZFMKTIS2517092 7.22E 50.46N ad f Drassodes lapidosus
ZFMKTIS20930 7.22E 50.47N ad m Drassodes lapidosus
SMNSAranJHS387 9.09E 48.91N ad f Drassodes lapidosus
ZSMARACH00707 11.62E 51.53N ad m Drassodes lapidosus
ZFMKTIS5016 14.51E 48.19N ad m Drassodes lapidosus
ZFMKTIS2673 8.15E 46.41N ad f Drassodes lapidosus
SMNSAran892 9.88E 48.44N jv f Drassodes lapidosus
SMNSAran894 9.88E 48.44N ad m Drassodes lapidosus
ZSMARACH00093 10.19E 48.7N ad f Drassodes lapidosus
SMNSAran895 9.88E 48.44N sbad f Drassodes lapidosus
SMNSAran893 9.88E 48.44N ad m Drassodes lapidosus
ZSMARACH00664 11.5E 51.52N ad m Drassodes lapidosus
ZSMARACH00520 11.11E 52.44N ad f Phrurolithus festivus
SMNKARA09018 8.48E 48.97N ad m Phrurolithus festivus
SMNKARA09033 8.48E 48.97N ad m Phrurolithus festivus
ZFMKTIS2505008 8.8E 53.71N ad f Phrurolithus festivus
ZSMARACH00462 14.14E 52.88N ad f Phrurolithus festivus
ZFMKTIS2418 13.69E 54.31N ad f Phrurolithus festivus
ZFMKTIS2524798 10.75E 53.81N ad m Phrurolithus festivus
ZFMKTIS18420 12.7E 51.58N ad m Phrurolithus festivus
ZSMARACH00716 13.28E 52.03N ad m Berlandina cinerea
ZSMARACH00470 13.28E 52.03N ad m Berlandina cinerea
ZFMKTIS20927 7.22E 50.47N ad m Callilepis schuszteri
ZFMKTIS5015 14.51E 48.19N ad f Callilepis nocturna
SMNKARA08197 8.87E 48.8N ad f Callilepis nocturna
SMNSAran890 9.84E 48.45N ad f Gnaphosa lucifuga
ZSMARACH00537 11.62E 51.53N ad f Gnaphosa lucifuga
SMNSAran810 9.74E 48.62N ad f Gnaphosa lucifuga
ZSMARACH00538 11.62E 51.53N ad f Gnaphosa lucifuga
0.5

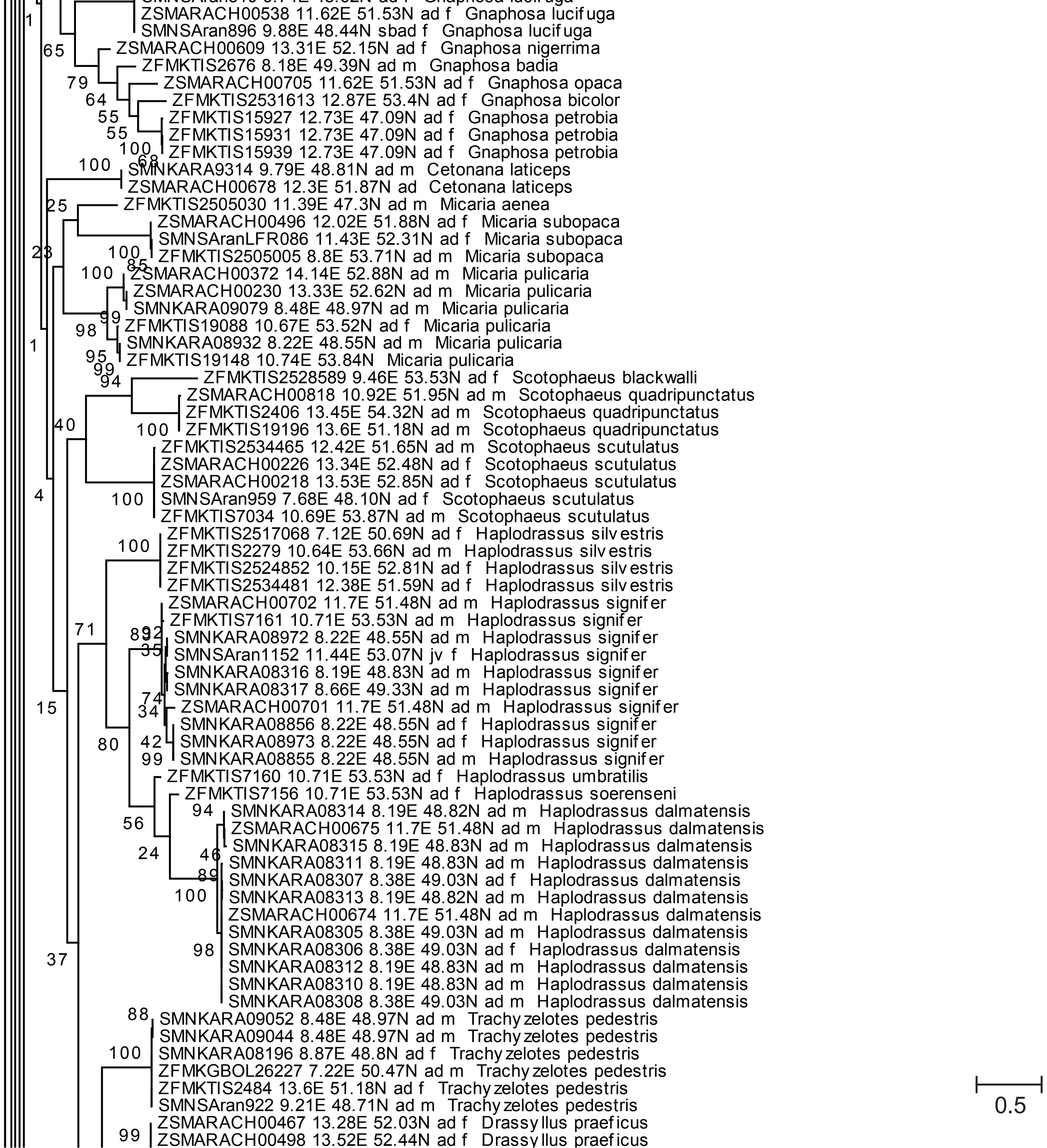
ZSMARACH00538 11.62E 51.53N ad f Gnaphosa lucifuga
SMNSAran896 9.88E 48.44N sbad f Gnaphosa lucifuga
ZSMARACH00609 13.31E 52.15N ad f Gnaphosa nigerrima
ZFMKTIS2676 8.18E 49.39N ad m Gnaphosa badia
ZSMARACH00705 11.62E 51.53N ad f Gnaphosa opaca
ZFMKTIS2531613 12.87E 53.4N ad f Gnaphosa bicolor
ZFMKTIS15927 12.73E 47.09N ad f Gnaphosa petrobia
ZFMKTIS15931 12.73E 47.09N ad f Gnaphosa petrobia
ZFMKTIS15939 12.73E 47.09N ad f Gnaphosa petrobia
SMNKARA9314 9.79E 48.81N ad m Cetonana laticeps
ZSMARACH00678 12.3E 51.87N ad Cetonana laticeps
ZFMKTIS2505030 11.39E 47.3N ad m Micaria aenea
ZSMARACH00496 12.02E 51.88N ad f Micaria subopaca
SMNSAranLFR086 11.43E 52.31N ad f Micaria subopaca
ZFMKTIS2505005 8.8E 53.71N ad m Micaria subopaca
ZSMARACH00372 14.14E 52.88N ad m Micaria pulicaria
ZSMARACH00230 13.33E 52.62N ad m Micaria pulicaria
SMNKARA09079 8.48E 48.97N ad m Micaria pulicaria
ZFMKTIS19088 10.67E 53.52N ad f Micaria pulicaria
SMNKARA08932 8.22E 48.55N ad m Micaria pulicaria
ZFMKTIS19148 10.74E 53.84N Micaria pulicaria
ZFMKTIS2528589 9.46E 53.53N ad f Scotophaeus blackwalli
ZSMARACH00818 10.92E 51.95N ad m Scotophaeus quadripunctatus
ZFMKTIS2406 13.45E 54.32N ad m Scotophaeus quadripunctatus
ZFMKTIS19196 13.6E 51.18N ad m Scotophaeus quadripunctatus
ZFMKTIS2534465 12.42E 51.65N ad m Scotophaeus scutulatus
ZSMARACH00226 13.34E 52.48N ad f Scotophaeus scutulatus
ZSMARACH00218 13.53E 52.85N ad f Scotophaeus scutulatus
SMNSAran959 7.68E 48.10N ad f Scotophaeus scutulatus
ZFMKTIS7034 10.69E 53.87N ad m Scotophaeus scutulatus
ZFMKTIS2517068 7.12E 50.69N ad f Haplodrassus silvestris
ZFMKTIS2279 10.64E 53.66N ad m Haplodrassus silvestris
ZFMKTIS2524852 10.15E 52.81N ad f Haplodrassus silvestris
ZFMKTIS2534481 12.38E 51.59N ad f Haplodrassus silvestris
ZSMARACH00702 11.7E 51.48N ad m Haplodrassus signifer
ZFMKTIS7161 10.71E 53.53N ad m Haplodrassus signifer
SMNKARA08972 8.22E 48.55N ad m Haplodrassus signifer
SMNSAran1152 11.44E 53.07N jv f Haplodrassus signifer
SMNKARA08316 8.19E 48.83N ad m Haplodrassus signifer
SMNKARA08317 8.66E 49.33N ad m Haplodrassus signifer
ZSMARACH00701 11.7E 51.48N ad m Haplodrassus signifer
SMNKARA08856 8.22E 48.55N ad f Haplodrassus signifer
SMNKARA08973 8.22E 48.55N ad f Haplodrassus signifer
SMNKARA08855 8.22E 48.55N ad m Haplodrassus signifer
ZFMKTIS7160 10.71E 53.53N ad f Haplodrassus umbratilis
ZFMKTIS7156 10.71E 53.53N ad f Haplodrassus soerenseni
SMNKARA08314 8.19E 48.82N ad m Haplodrassus dalmatensis
ZSMARACH00675 11.7E 51.48N ad m Haplodrassus dalmatensis
SMNKARA08315 8.19E 48.83N ad m Haplodrassus dalmatensis
SMNKARA08311 8.19E 48.83N ad m Haplodrassus dalmatensis
SMNKARA08307 8.38E 49.03N ad f Haplodrassus dalmatensis
SMNKARA08313 8.19E 48.82N ad m Haplodrassus dalmatensis
ZSMARACH00674 11.7E 51.48N ad m Haplodrassus dalmatensis
SMNKARA08305 8.38E 49.03N ad m Haplodrassus dalmatensis
SMNKARA08306 8.38E 49.03N ad f Haplodrassus dalmatensis
SMNKARA08312 8.19E 48.83N ad m Haplodrassus dalmatensis
SMNKARA08310 8.19E 48.83N ad m Haplodrassus dalmatensis
SMNKARA08308 8.38E 49.03N ad m Haplodrassus dalmatensis
SMNKARA09052 8.48E 48.97N ad m Trachyzelotes pedestris
SMNKARA09044 8.48E 48.97N ad m Trachyzelotes pedestris
SMNKARA08196 8.87E 48.8N ad f Trachyzelotes pedestris
ZFMKGBOL26227 7.22E 50.47N ad m Trachyzelotes pedestris
ZFMKTIS2484 13.6E 51.18N ad f Trachyzelotes pedestris
SMNSAran922 9.21E 48.71N ad m Trachyzelotes pedestris
ZSMARACH00467 13.28E 52.03N ad f Drassyllus praeficus
ZSMARACH00498 13.52E 52.44N ad f Drassyllus praeficus
0.5

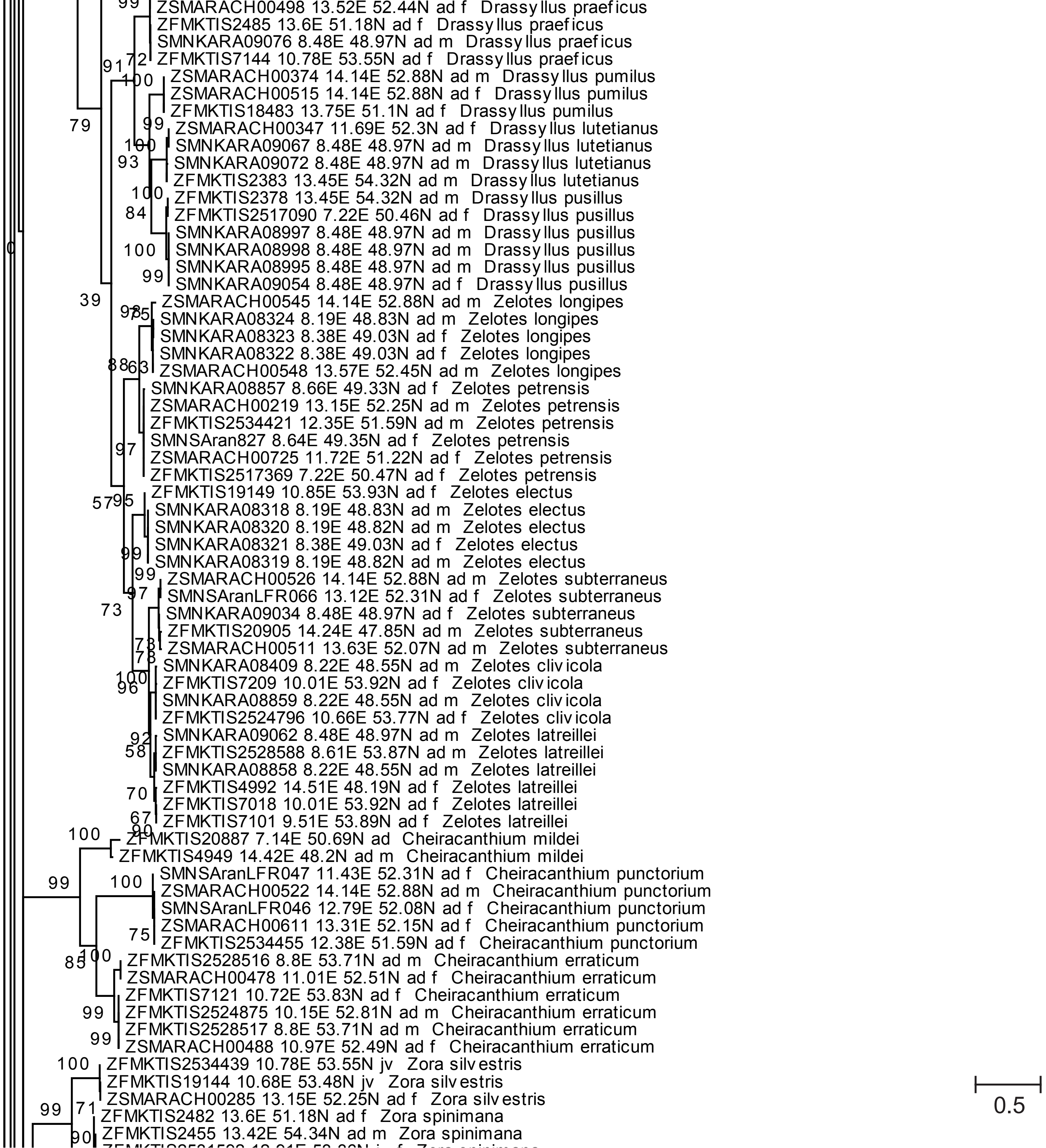
ZSMARACH00498 13.52E 52.44N ad f Drassyllus praeficus
ZFMKTIS2485 13.6E 51.18N ad f Drassyllus praeficus
SMNKARA09076 8.48E 48.97N ad m Drassyllus praeficus
ZFMKTIS7144 10.78E 53.55N ad f Drassyllus praeficus
ZSMARACH00374 14.14E 52.88N ad m Drassyllus pumilus
ZSMARACH00515 14.14E 52.88N ad f Drassyllus pumilus
ZFMKTIS18483 13.75E 51.1N ad f Drassyllus pumilus
ZSMARACH00347 11.69E 52.3N ad f Drassyllus lutetianus
SMNKARA09067 8.48E 48.97N ad m Drassyllus lutetianus
SMNKARA09072 8.48E 48.97N ad m Drassyllus lutetianus
ZFMKTIS2383 13.45E 54.32N ad m Drassyllus lutetianus
ZFMKTIS2378 13.45E 54.32N ad m Drassyllus pusillus
ZFMKTIS2517090 7.22E 50.46N ad f Drassyllus pusillus
SMNKARA08997 8.48E 48.97N ad m Drassyllus pusillus
SMNKARA08998 8.48E 48.97N ad m Drassyllus pusillus
SMNKARA08995 8.48E 48.97N ad m Drassyllus pusillus
SMNKARA09054 8.48E 48.97N ad f Drassyllus pusillus
ZSMARACH00545 14.14E 52.88N ad m Zelotes longipes
SMNKARA08324 8.19E 48.83N ad m Zelotes longipes
SMNKARA08323 8.38E 49.03N ad f Zelotes longipes
SMNKARA08322 8.38E 49.03N ad f Zelotes longipes
ZSMARACH00548 13.57E 52.45N ad m Zelotes longipes
SMNKARA08857 8.66E 49.33N ad f Zelotes petrensis
ZSMARACH00219 13.15E 52.25N ad m Zelotes petrensis
ZFMKTIS2534421 12.35E 51.59N ad m Zelotes petrensis
SMNSAran827 8.64E 49.35N ad f Zelotes petrensis
ZSMARACH00725 11.72E 51.22N ad f Zelotes petrensis
ZFMKTIS2517369 7.22E 50.47N ad f Zelotes petrensis
ZFMKTIS19149 10.85E 53.93N ad f Zelotes electus
SMNKARA08318 8.19E 48.83N ad m Zelotes electus
SMNKARA08320 8.19E 48.82N ad m Zelotes electus
SMNKARA08321 8.38E 49.03N ad f Zelotes electus
SMNKARA08319 8.19E 48.82N ad m Zelotes electus
ZSMARACH00526 14.14E 52.88N ad m Zelotes subterraneus
SMNSAranLFR066 13.12E 52.31N ad f Zelotes subterraneus
SMNKARA09034 8.48E 48.97N ad f Zelotes subterraneus
ZFMKTIS20905 14.24E 47.85N ad m Zelotes subterraneus
ZSMARACH00511 13.63E 52.07N ad m Zelotes subterraneus
SMNKARA08409 8.22E 48.55N ad m Zelotes clivicola
ZFMKTIS7209 10.01E 53.92N ad f Zelotes clivicola
SMNKARA08859 8.22E 48.55N ad m Zelotes clivicola
ZFMKTIS2524796 10.66E 53.77N ad f Zelotes clivicola
SMNKARA09062 8.48E 48.97N ad m Zelotes latreillei
ZFMKTIS2528588 8.61E 53.87N ad m Zelotes latreillei
SMNKARA08858 8.22E 48.55N ad m Zelotes latreillei
ZFMKTIS4992 14.51E 48.19N ad f Zelotes latreillei
ZFMKTIS7018 10.01E 53.92N ad f Zelotes latreillei
ZFMKTIS7101 9.51E 53.89N ad f Zelotes latreillei
ZFMKTIS20887 7.14E 50.69N ad Cheiracanthium mildei
ZFMKTIS4949 14.42E 48.2N ad m Cheiracanthium mildei
SMNSAranLFR047 11.43E 52.31N ad f Cheiracanthium punctorium
ZSMARACH00522 14.14E 52.88N ad m Cheiracanthium punctorium
SMNSAranLFR046 12.79E 52.08N ad f Cheiracanthium punctorium
ZSMARACH00611 13.31E 52.15N ad f Cheiracanthium punctorium
ZFMKTIS2534455 12.38E 51.59N ad f Cheiracanthium punctorium
ZFMKTIS2528516 8.8E 53.71N ad m Cheiracanthium erraticum
ZSMARACH00478 11.01E 52.51N ad f Cheiracanthium erraticum
ZFMKTIS7121 10.72E 53.83N ad f Cheiracanthium erraticum
ZFMKTIS2524875 10.15E 52.81N ad m Cheiracanthium erraticum
ZFMKTIS2528517 8.8E 53.71N ad m Cheiracanthium erraticum
ZSMARACH00488 10.97E 52.49N ad f Cheiracanthium erraticum
ZFMKTIS2534439 10.78E 53.55N jv Zora silvestris
ZFMKTIS19144 10.68E 53.48N jv Zora silvestris
ZSMARACH00285 13.15E 52.25N ad f Zora silvestris
ZFMKTIS2482 13.6E 51.18N ad f Zora spinimana
ZFMKTIS2455 13.42E 54.34N ad m Zora spinimana
0.5

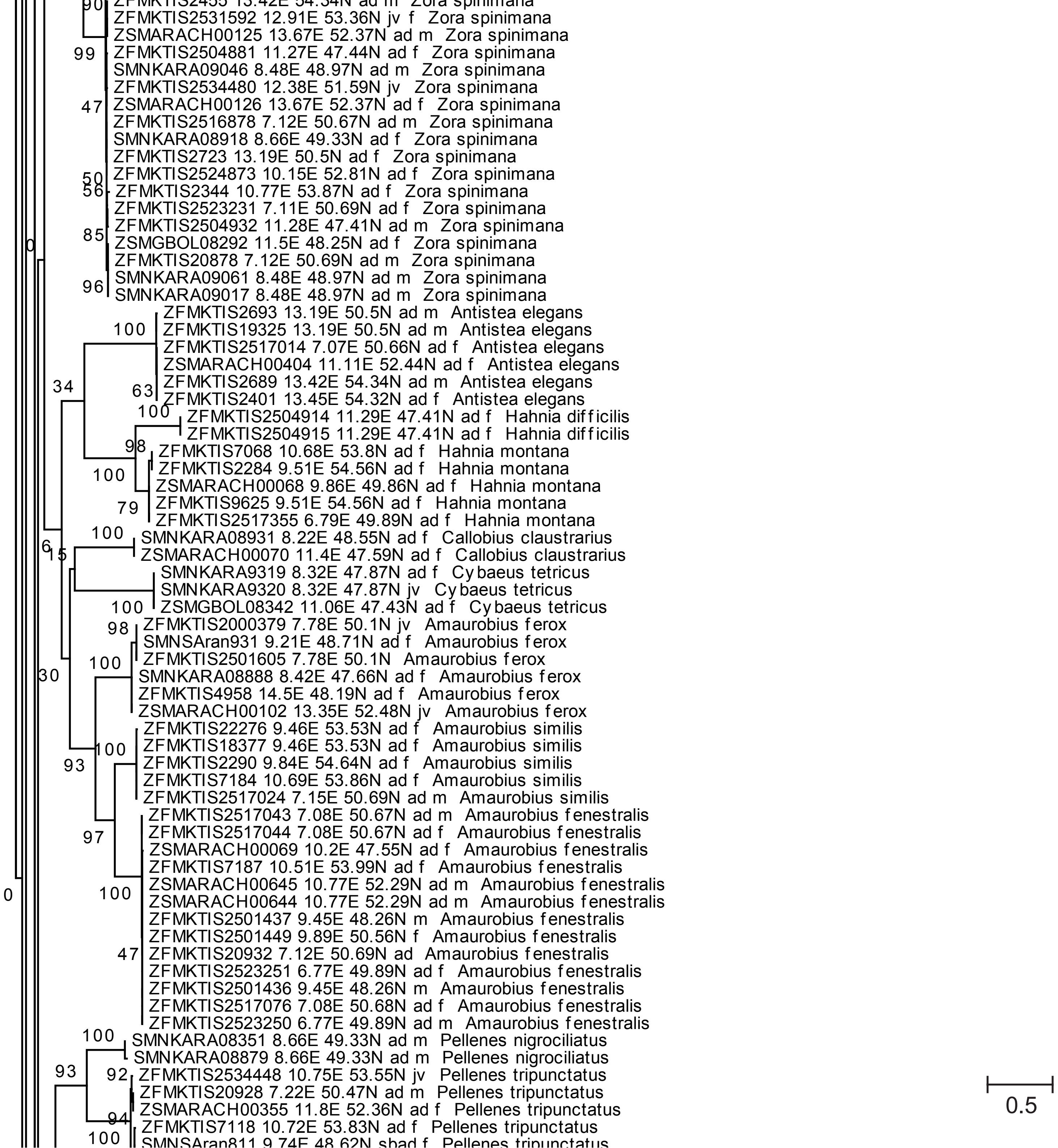
ZFMKTIS2531592 12.91E 53.36N jv f Zora spinimana
ZSMARACH00125 13.67E 52.37N ad m Zora spinimana
ZFMKTIS2504881 11.27E 47.44N ad f Zora spinimana
SMNKARA09046 8.48E 48.97N ad m Zora spinimana
ZFMKTIS2534480 12.38E 51.59N jv Zora spinimana
ZSMARACH00126 13.67E 52.37N ad f Zora spinimana
ZFMKTIS2516878 7.12E 50.67N ad m Zora spinimana
SMNKARA08918 8.66E 49.33N ad f Zora spinimana
ZFMKTIS2723 13.19E 50.5N ad f Zora spinimana
ZFMKTIS2524873 10.15E 52.81N ad f Zora spinimana
ZFMKTIS2344 10.77E 53.87N ad f Zora spinimana
ZFMKTIS2523231 7.11E 50.69N ad f Zora spinimana
ZFMKTIS2504932 11.28E 47.41N ad m Zora spinimana
ZSMGBOL08292 11.5E 48.25N ad f Zora spinimana
ZFMKTIS20878 7.12E 50.69N ad m Zora spinimana
SMNKARA09061 8.48E 48.97N ad m Zora spinimana
SMNKARA09017 8.48E 48.97N ad m Zora spinimana
ZFMKTIS2693 13.19E 50.5N ad m Antistea elegans
ZFMKTIS19325 13.19E 50.5N ad m Antistea elegans
ZFMKTIS2517014 7.07E 50.66N ad f Antistea elegans
ZSMARACH00404 11.11E 52.44N ad f Antistea elegans
ZFMKTIS2689 13.42E 54.34N ad m Antistea elegans
ZFMKTIS2401 13.45E 54.32N ad f Antistea elegans
ZFMKTIS2504914 11.29E 47.41N ad f Hahnia difficilis
ZFMKTIS2504915 11.29E 47.41N ad f Hahnia difficilis
ZFMKTIS7068 10.68E 53.8N ad f Hahnia montana
ZFMKTIS2284 9.51E 54.56N ad f Hahnia montana
ZSMARACH00068 9.86E 49.86N ad f Hahnia montana
ZFMKTIS9625 9.51E 54.56N ad f Hahnia montana
ZFMKTIS2517355 6.79E 49.89N ad f Hahnia montana
SMNKARA08931 8.22E 48.55N ad f Callobius claustrarius
ZSMARACH00070 11.4E 47.59N ad f Callobius claustrarius
SMNKARA9319 8.32E 47.87N ad f Cybaeus tetricus
SMNKARA9320 8.32E 47.87N jv Cybaeus tetricus
ZSMGBOL08342 11.06E 47.43N ad f Cybaeus tetricus
ZFMKTIS2000379 7.78E 50.1N jv Amaurobius ferox
SMNSAran931 9.21E 48.71N ad f Amaurobius ferox
ZFMKTIS2501605 7.78E 50.1N Amaurobius ferox
SMNKARA08888 8.42E 47.66N ad f Amaurobius ferox
ZFMKTIS4958 14.5E 48.19N ad f Amaurobius ferox
ZSMARACH00102 13.35E 52.48N jv Amaurobius ferox
ZFMKTIS22276 9.46E 53.53N ad f Amaurobius similis
ZFMKTIS18377 9.46E 53.53N ad f Amaurobius similis
ZFMKTIS2290 9.84E 54.64N ad f Amaurobius similis
ZFMKTIS7184 10.69E 53.86N ad f Amaurobius similis
ZFMKTIS2517024 7.15E 50.69N ad m Amaurobius similis
ZFMKTIS2517043 7.08E 50.67N ad m Amaurobius fenestralis
ZFMKTIS2517044 7.08E 50.67N ad f Amaurobius fenestralis
ZSMARACH00069 10.2E 47.55N ad f Amaurobius fenestralis
ZFMKTIS7187 10.51E 53.99N ad f Amaurobius fenestralis
ZSMARACH00645 10.77E 52.29N ad m Amaurobius fenestralis
ZSMARACH00644 10.77E 52.29N ad m Amaurobius fenestralis
ZFMKTIS2501437 9.45E 48.26N m Amaurobius fenestralis
ZFMKTIS2501449 9.89E 50.56N f Amaurobius fenestralis
ZFMKTIS20932 7.12E 50.69N ad Amaurobius fenestralis
ZFMKTIS2523251 6.77E 49.89N ad f Amaurobius fenestralis
ZFMKTIS2501436 9.45E 48.26N m Amaurobius fenestralis
ZFMKTIS2517076 7.08E 50.68N ad f Amaurobius fenestralis
ZFMKTIS2523250 6.77E 49.89N ad m Amaurobius fenestralis
SMNKARA08351 8.66E 49.33N ad m Pellenes nigrociliatus
SMNKARA08879 8.66E 49.33N ad m Pellenes nigrociliatus
ZFMKTIS2534448 10.75E 53.55N jv Pellenes tripunctatus
ZFMKTIS20928 7.22E 50.47N ad m Pellenes tripunctatus
ZSMARACH00355 11.8E 52.36N ad f Pellenes tripunctatus
ZFMKTIS7118 10.72E 53.83N ad f Pellenes tripunctatus
0.5

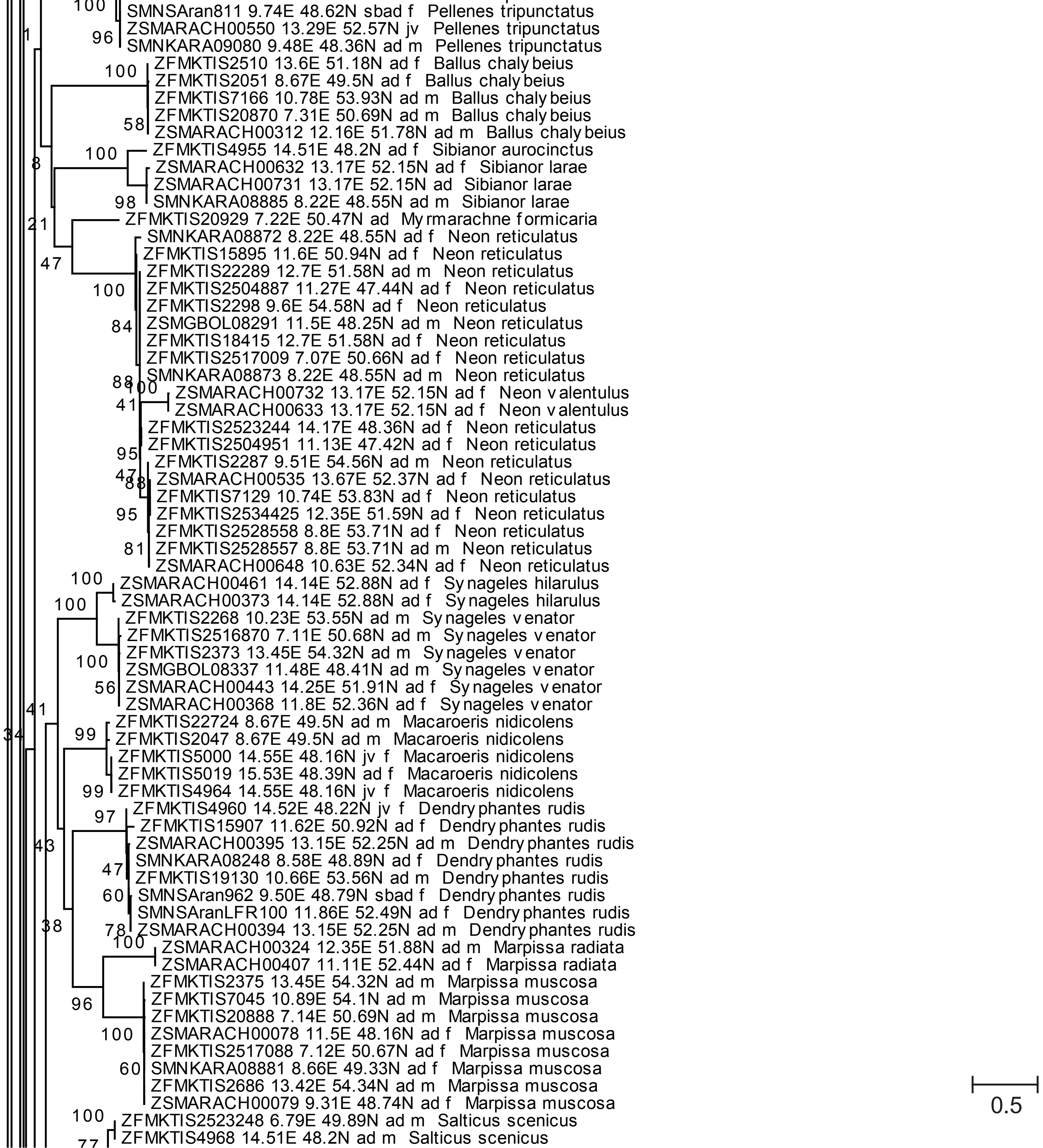

SMNSAran811 9.74E 48.62N sbad f Pellenes tripunctatus
ZSMARACH00550 13.29E 52.57N jv Pellenes tripunctatus
SMNKARA09080 9.48E 48.36N ad m Pellenes tripunctatus
ZFMKTIS2510 13.6E 51.18N ad f Ballus chaly beius
ZFMKTIS2051 8.67E 49.5N ad f Ballus chaly beius
ZFMKTIS7166 10.78E 53.93N ad m Ballus chaly beius
ZFMKTIS20870 7.31E 50.69N ad m Ballus chaly beius
ZSMARACH00312 12.16E 51.78N ad m Ballus chaly beius
ZFMKTIS4955 14.51E 48.2N ad f Sibianor aurocinctus
ZSMARACH00632 13.17E 52.15N ad f Sibianor larae
ZSMARACH00731 13.17E 52.15N ad Sibianor larae
SMNKARA08885 8.22E 48.55N ad m Sibianor larae
ZFMKTIS20929 7.22E 50.47N ad My rmarachne f ormicaria
SMNKARA08872 8.22E 48.55N ad f Neon reticulatus
ZFMKTIS15895 11.6E 50.94N ad f Neon reticulatus
ZFMKTIS22289 12.7E 51.58N ad m Neon reticulatus
ZFMKTIS2504887 11.27E 47.44N ad f Neon reticulatus
ZFMKTIS2298 9.6E 54.58N ad f Neon reticulatus
ZSMGBOL08291 11.5E 48.25N ad m Neon reticulatus
ZFMKTIS18415 12.7E 51.58N ad f Neon reticulatus
ZFMKTIS2517009 7.07E 50.66N ad f Neon reticulatus
SMNKARA08873 8.22E 48.55N ad m Neon reticulatus
ZSMARACH00732 13.17E 52.15N ad f Neon v alentulus
ZSMARACH00633 13.17E 52.15N ad f Neon v alentulus
ZFMKTIS2523244 14.17E 48.36N ad f Neon reticulatus
ZFMKTIS2504951 11.13E 47.42N ad f Neon reticulatus
ZFMKTIS2287 9.51E 54.56N ad m Neon reticulatus
ZSMARACH00535 13.67E 52.37N ad f Neon reticulatus
ZFMKTIS7129 10.74E 53.83N ad f Neon reticulatus
ZFMKTIS2534425 12.35E 51.59N ad f Neon reticulatus
ZFMKTIS2528558 8.8E 53.71N ad f Neon reticulatus
ZFMKTIS2528557 8.8E 53.71N ad m Neon reticulatus
ZSMARACH00648 10.63E 52.34N ad f Neon reticulatus
ZSMARACH00461 14.14E 52.88N ad f Sy nageles hilarulus
ZSMARACH00373 14.14E 52.88N ad f Sy nageles hilarulus
ZFMKTIS2268 10.23E 53.55N ad m Sy nageles v enator
ZFMKTIS2516870 7.11E 50.68N ad m Sy nageles v enator
ZFMKTIS2373 13.45E 54.32N ad m Sy nageles v enator
ZSMGBOL08337 11.48E 48.41N ad m Sy nageles v enator
ZSMARACH00443 14.25E 51.91N ad f Sy nageles v enator
ZSMARACH00368 11.8E 52.36N ad f Sy nageles v enator
ZFMKTIS22724 8.67E 49.5N ad m Macaroeris nidicolens
ZFMKTIS2047 8.67E 49.5N ad m Macaroeris nidicolens
ZFMKTIS5000 14.55E 48.16N jv f Macaroeris nidicolens
ZFMKTIS5019 15.53E 48.39N ad f Macaroeris nidicolens
ZFMKTIS4964 14.55E 48.16N jv f Macaroeris nidicolens
ZFMKTIS4960 14.52E 48.22N jv f Dendry phantes rudis
ZFMKTIS15907 11.62E 50.92N ad f Dendry phantes rudis
ZSMARACH00395 13.15E 52.25N ad m Dendry phantes rudis
SMNKARA08248 8.58E 48.89N ad f Dendry phantes rudis
ZFMKTIS19130 10.66E 53.56N ad m Dendry phantes rudis
SMNSAran962 9.50E 48.79N sbad f Dendry phantes rudis
SMNSAranLFR100 11.86E 52.49N ad f Dendry phantes rudis
ZSMARACH00394 13.15E 52.25N ad m Dendry phantes rudis
ZSMARACH00324 12.35E 51.88N ad m Marpissa radiata
ZSMARACH00407 11.11E 52.44N ad f Marpissa radiata
ZFMKTIS2375 13.45E 54.32N ad m Marpissa muscosa
ZFMKTIS7045 10.89E 54.1N ad m Marpissa muscosa
ZFMKTIS20888 7.14E 50.69N ad m Marpissa muscosa
ZSMARACH00078 11.5E 48.16N ad f Marpissa muscosa
ZFMKTIS2517088 7.12E 50.67N ad f Marpissa muscosa
SMNKARA08881 8.66E 49.33N ad f Marpissa muscosa
ZFMKTIS2686 13.42E 54.34N ad m Marpissa muscosa
ZSMARACH00079 9.31E 48.74N ad f Marpissa muscosa
ZFMKTIS2523248 6.79E 49.89N ad m Salticus scenicus
ZFMKTIS4968 14.51E 48.2N ad m Salticus scenicus
0.5

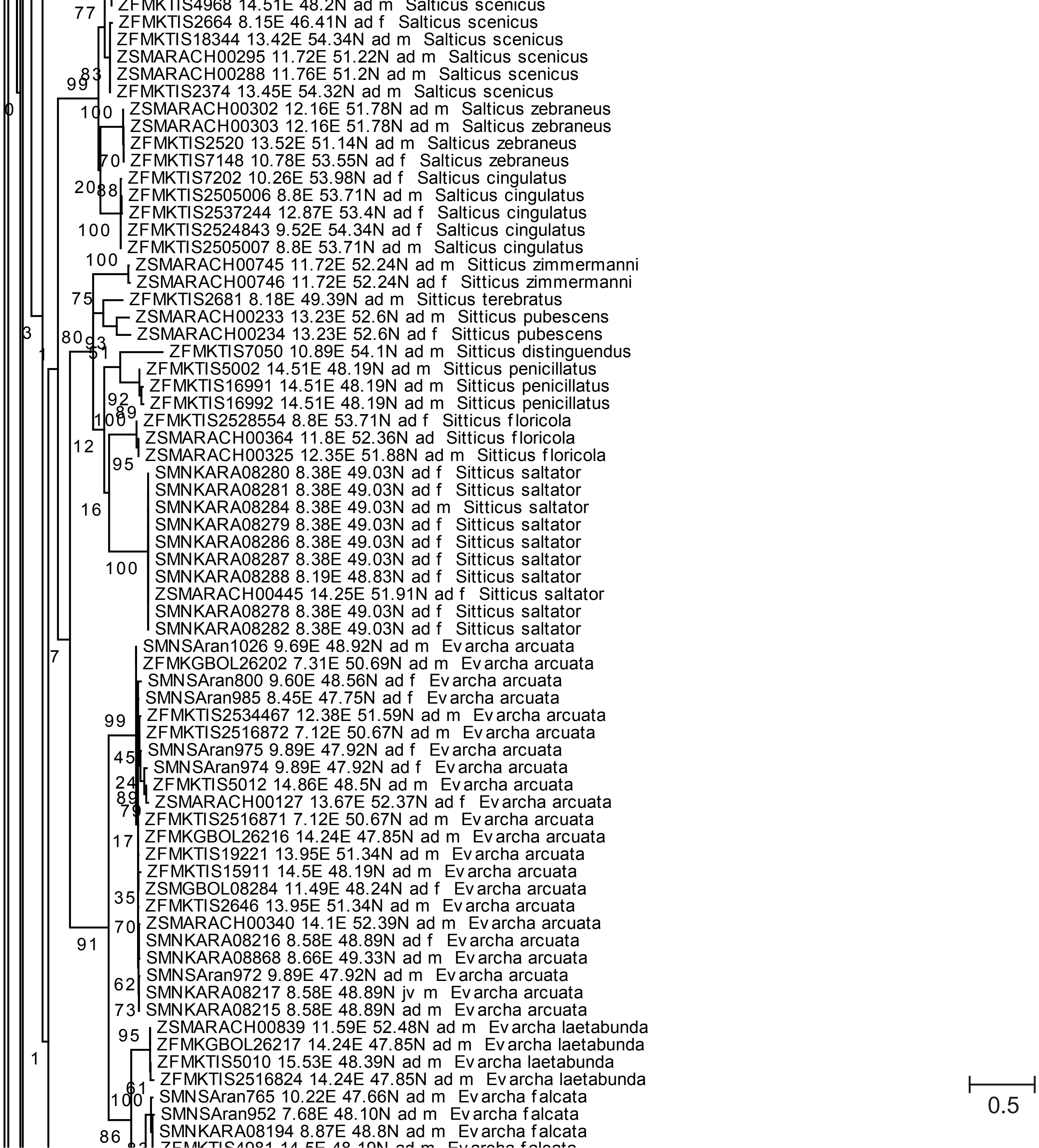
ZFMKTIS4968 14.51E 48.2N ad m Salticus scenicus
ZFMKTIS2664 8.15E 46.41N ad f Salticus scenicus
ZFMKTIS18344 13.42E 54.34N ad m Salticus scenicus
ZSMARACH00295 11.72E 51.22N ad m Salticus scenicus
ZSMARACH00288 11.76E 51.2N ad m Salticus scenicus
ZFMKTIS2374 13.45E 54.32N ad m Salticus scenicus
ZSMARACH00302 12.16E 51.78N ad m Salticus zebraneus
ZSMARACH00303 12.16E 51.78N ad m Salticus zebraneus
ZFMKTIS2520 13.52E 51.14N ad m Salticus zebraneus
ZFMKTIS7148 10.78E 53.55N ad f Salticus zebraneus
ZFMKTIS7202 10.26E 53.98N ad f Salticus cingulatus
ZFMKTIS2505006 8.8E 53.71N ad m Salticus cingulatus
ZFMKTIS2537244 12.87E 53.4N ad f Salticus cingulatus
ZFMKTIS2524843 9.52E 54.34N ad f Salticus cingulatus
ZFMKTIS2505007 8.8E 53.71N ad m Salticus cingulatus
ZSMARACH00745 11.72E 52.24N ad m Sitticus zimmermanni
ZSMARACH00746 11.72E 52.24N ad f Sitticus zimmermanni
ZFMKTIS2681 8.18E 49.39N ad m Sitticus terebratus
ZSMARACH00233 13.23E 52.6N ad m Sitticus pubescens
ZSMARACH00234 13.23E 52.6N ad f Sitticus pubescens
ZFMKTIS7050 10.89E 54.1N ad m Sitticus distinguendus
ZFMKTIS5002 14.51E 48.19N ad m Sitticus penicillatus
ZFMKTIS16991 14.51E 48.19N ad m Sitticus penicillatus
ZFMKTIS16992 14.51E 48.19N ad m Sitticus penicillatus
ZFMKTIS2528554 8.8E 53.71N ad f Sitticus floricola
ZSMARACH00364 11.8E 52.36N ad Sitticus floricola
ZSMARACH00325 12.35E 51.88N ad m Sitticus floricola
SMNKARA08280 8.38E 49.03N ad f Sitticus saltator
SMNKARA08281 8.38E 49.03N ad f Sitticus saltator
SMNKARA08284 8.38E 49.03N ad m Sitticus saltator
SMNKARA08279 8.38E 49.03N ad f Sitticus saltator
SMNKARA08286 8.38E 49.03N ad f Sitticus saltator
SMNKARA08287 8.38E 49.03N ad f Sitticus saltator
SMNKARA08288 8.19E 48.83N ad f Sitticus saltator
ZSMARACH00445 14.25E 51.91N ad f Sitticus saltator
SMNKARA08278 8.38E 49.03N ad f Sitticus saltator
SMNKARA08282 8.38E 49.03N ad f Sitticus saltator
SMNSAran1026 9.69E 48.92N ad m Evarcha arcuata
ZFMKGBOL26202 7.31E 50.69N ad m Evarcha arcuata
SMNSAran800 9.60E 48.56N ad f Evarcha arcuata
SMNSAran985 8.45E 47.75N ad f Evarcha arcuata
ZFMKTIS2534467 12.38E 51.59N ad m Evarcha arcuata
ZFMKTIS2516872 7.12E 50.67N ad m Evarcha arcuata
SMNSAran975 9.89E 47.92N ad f Evarcha arcuata
SMNSAran974 9.89E 47.92N ad f Evarcha arcuata
ZFMKTIS5012 14.86E 48.5N ad m Evarcha arcuata
ZSMARACH00127 13.67E 52.37N ad f Evarcha arcuata
ZFMKTIS2516871 7.12E 50.67N ad m Evarcha arcuata
ZFMKGBOL26216 14.24E 47.85N ad m Evarcha arcuata
ZFMKTIS19221 13.95E 51.34N ad m Evarcha arcuata
ZFMKTIS15911 14.5E 48.19N ad m Evarcha arcuata
ZSMGBOL08284 11.49E 48.24N ad f Evarcha arcuata
ZFMKTIS2646 13.95E 51.34N ad m Evarcha arcuata
ZSMARACH00340 14.1E 52.39N ad m Evarcha arcuata
SMNKARA08216 8.58E 48.89N ad f Evarcha arcuata
SMNKARA08868 8.66E 49.33N ad m Evarcha arcuata
SMNSAran972 9.89E 47.92N ad m Evarcha arcuata
SMNKARA08217 8.58E 48.89N jv m Evarcha arcuata
SMNKARA08215 8.58E 48.89N ad m Evarcha arcuata
ZSMARACH00839 11.59E 52.48N ad m Evarcha laetabunda
ZFMKGBOL26217 14.24E 47.85N ad m Evarcha laetabunda
ZFMKTIS5010 15.53E 48.39N ad m Evarcha laetabunda
ZFMKTIS2516824 14.24E 47.85N ad m Evarcha laetabunda
SMNSAran765 10.22E 47.66N ad m Evarcha falcata
SMNSAran952 7.68E 48.10N ad m Evarcha falcata
SMNKARA08194 8.87E 48.8N ad m Evarcha falcata
0.5

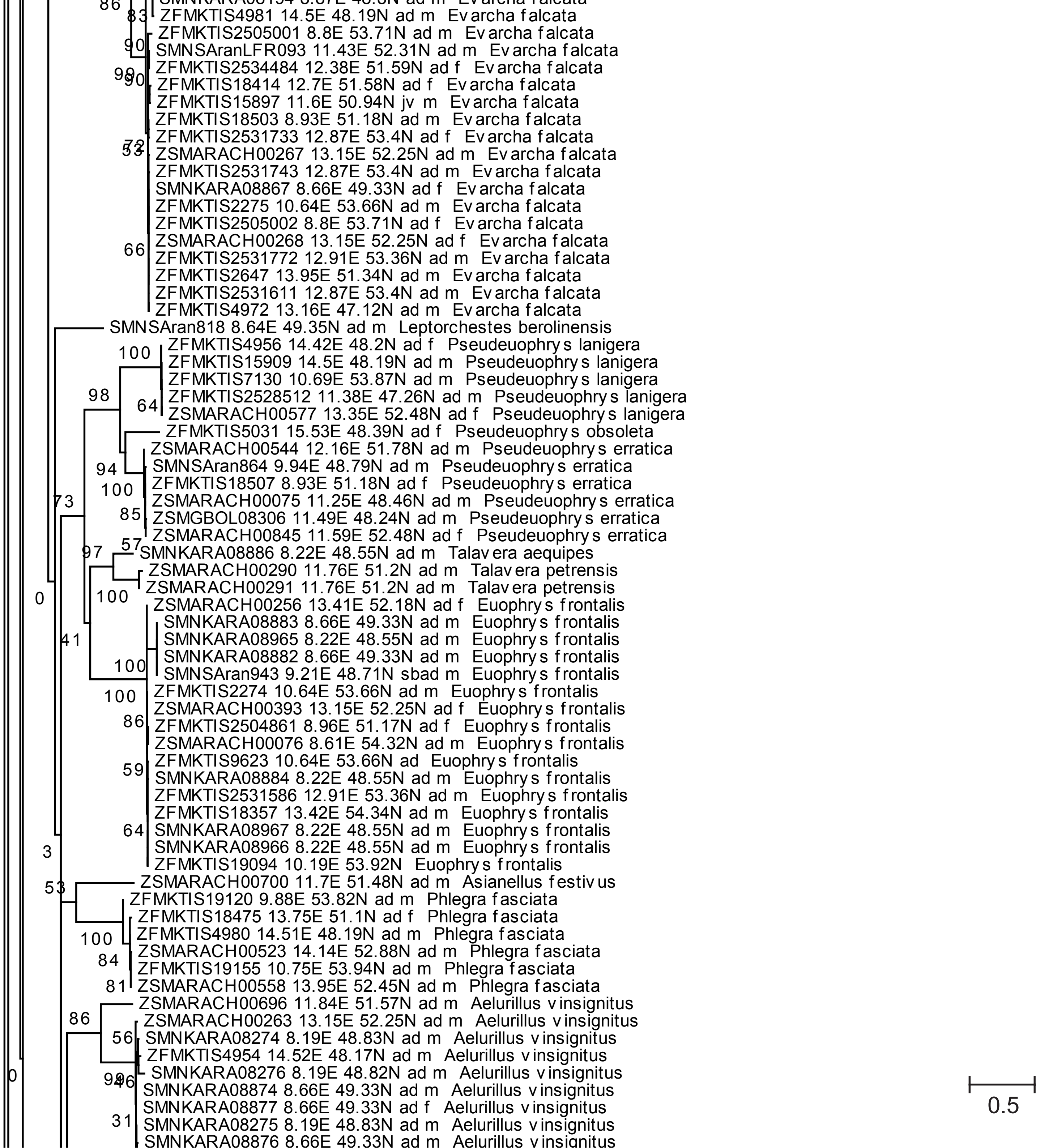
ZFMKTIS4981 14.5E 48.19N ad m Evarcha falcata
ZFMKTIS2505001 8.8E 53.71N ad m Evarcha falcata
SMNSAranLFR093 11.43E 52.31N ad m Evarcha falcata
ZFMKTIS2534484 12.38E 51.59N ad f Evarcha falcata
ZFMKTIS18414 12.7E 51.58N ad f Evarcha falcata
ZFMKTIS15897 11.6E 50.94N jv m Evarcha falcata
ZFMKTIS18503 8.93E 51.18N ad m Evarcha falcata
ZFMKTIS2531733 12.87E 53.4N ad f Evarcha falcata
ZSMARACH00267 13.15E 52.25N ad m Evarcha falcata
ZFMKTIS2531743 12.87E 53.4N ad m Evarcha falcata
SMNKARA08867 8.66E 49.33N ad f Evarcha falcata
ZFMKTIS2275 10.64E 53.66N ad m Evarcha falcata
ZFMKTIS2505002 8.8E 53.71N ad f Evarcha falcata
ZSMARACH00268 13.15E 52.25N ad f Evarcha falcata
ZFMKTIS2531772 12.91E 53.36N ad m Evarcha falcata
ZFMKTIS2647 13.95E 51.34N ad m Evarcha falcata
ZFMKTIS2531611 12.87E 53.4N ad m Evarcha falcata
ZFMKTIS4972 13.16E 47.12N ad m Evarcha falcata
SMNSAran818 8.64E 49.35N ad m Leptorchestes berolinensis
ZFMKTIS4956 14.42E 48.2N ad f Pseudeuophrys lanigera
ZFMKTIS15909 14.5E 48.19N ad m Pseudeuophrys lanigera
ZFMKTIS7130 10.69E 53.87N ad m Pseudeuophrys lanigera
ZFMKTIS2528512 11.38E 47.26N ad m Pseudeuophrys lanigera
ZSMARACH00577 13.35E 52.48N ad f Pseudeuophrys lanigera
ZFMKTIS5031 15.53E 48.39N ad f Pseudeuophrys obsoleta
ZSMARACH00544 12.16E 51.78N ad m Pseudeuophrys erratica
SMNSAran864 9.94E 48.79N ad m Pseudeuophrys erratica
ZFMKTIS18507 8.93E 51.18N ad f Pseudeuophrys erratica
ZSMARACH00075 11.25E 48.46N ad m Pseudeuophrys erratica
ZSMGBOL08306 11.49E 48.24N ad m Pseudeuophrys erratica
ZSMARACH00845 11.59E 52.48N ad f Pseudeuophrys erratica
SMNKARA08886 8.22E 48.55N ad m Talavera aequipes
ZSMARACH00290 11.76E 51.2N ad m Talavera petrensis
ZSMARACH00291 11.76E 51.2N ad m Talavera petrensis
ZSMARACH00256 13.41E 52.18N ad f Euophrys frontalis
SMNKARA08883 8.66E 49.33N ad m Euophrys frontalis
SMNKARA08965 8.22E 48.55N ad m Euophrys frontalis
SMNKARA08882 8.66E 49.33N ad m Euophrys frontalis
SMNSAran943 9.21E 48.71N sbad m Euophrys frontalis
ZFMKTIS2274 10.64E 53.66N ad m Euophrys frontalis
ZSMARACH00393 13.15E 52.25N ad f Euophrys frontalis
ZFMKTIS2504861 8.96E 51.17N ad f Euophrys frontalis
ZSMARACH00076 8.61E 54.32N ad m Euophrys frontalis
ZFMKTIS9623 10.64E 53.66N ad Euophrys frontalis
SMNKARA08884 8.22E 48.55N ad m Euophrys frontalis
ZFMKTIS2531586 12.91E 53.36N ad m Euophrys frontalis
ZFMKTIS18357 13.42E 54.34N ad m Euophrys frontalis
SMNKARA08967 8.22E 48.55N ad m Euophrys frontalis
SMNKARA08966 8.22E 48.55N ad m Euophrys frontalis
ZFMKTIS19094 10.19E 53.92N Euophrys frontalis
ZSMARACH00700 11.7E 51.48N ad m Asianellus festivus
ZFMKTIS19120 9.88E 53.82N ad m Phlegra fasciata
ZFMKTIS18475 13.75E 51.1N ad f Phlegra fasciata
ZFMKTIS4980 14.51E 48.19N ad m Phlegra fasciata
ZSMARACH00523 14.14E 52.88N ad m Phlegra fasciata
ZFMKTIS19155 10.75E 53.94N ad m Phlegra fasciata
ZSMARACH00558 13.95E 52.45N ad m Phlegra fasciata
ZSMARACH00696 11.84E 51.57N ad m Aelurillus vinsignitus
ZSMARACH00263 13.15E 52.25N ad m Aelurillus vinsignitus
SMNKARA08274 8.19E 48.83N ad m Aelurillus vinsignitus
ZFMKTIS4954 14.52E 48.17N ad m Aelurillus vinsignitus
SMNKARA08276 8.19E 48.82N ad m Aelurillus vinsignitus
SMNKARA08874 8.66E 49.33N ad m Aelurillus vinsignitus
SMNKARA08877 8.66E 49.33N ad f Aelurillus vinsignitus
SMNKARA08275 8.19E 48.83N ad m Aelurillus vinsignitus
SMNKARA08876 8.66E 49.33N ad m Aelurillus vinsignitus
0.5

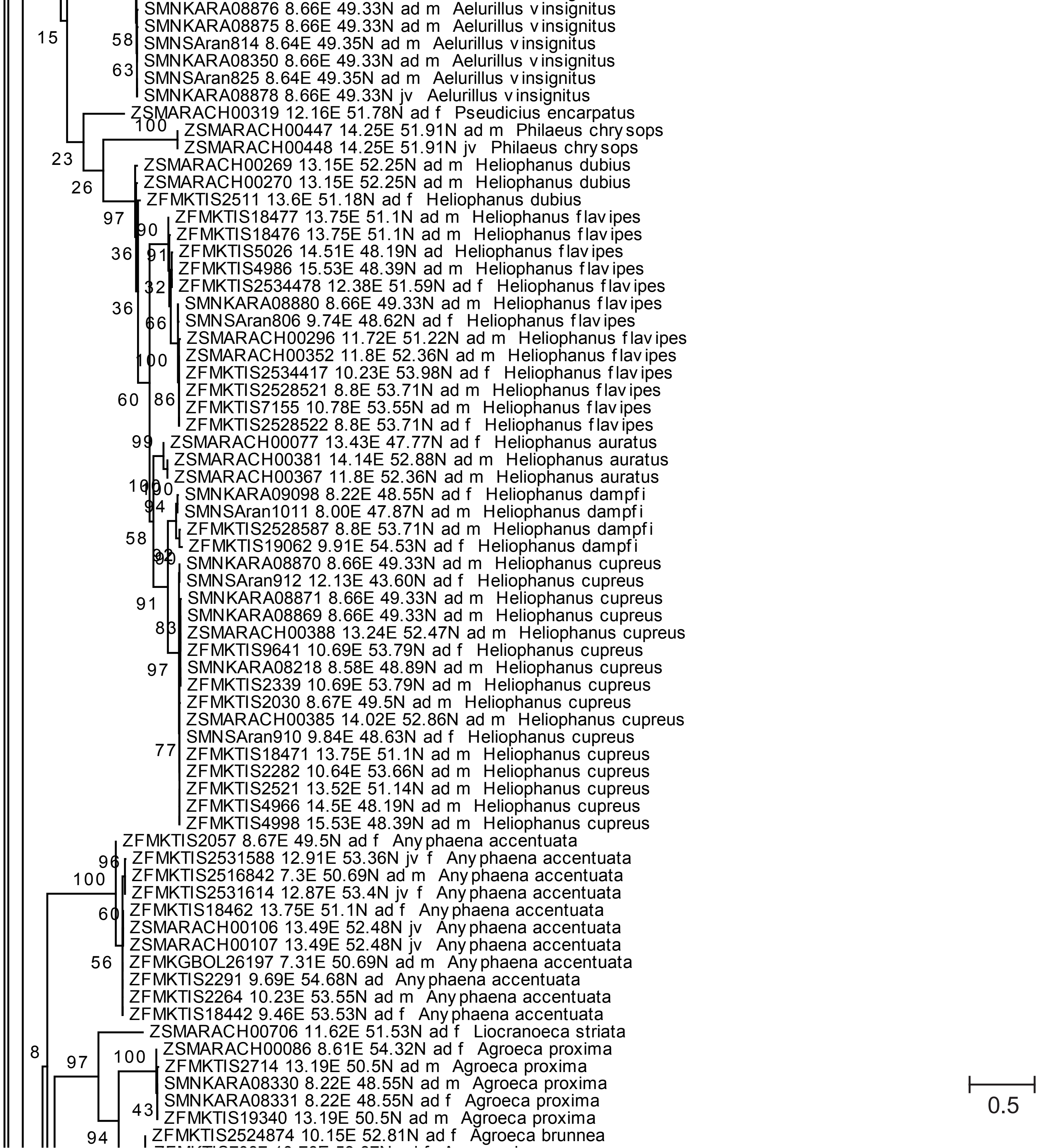
SMNKARA08876 8.66E 49.33N ad m Aelurillus v insignitus
SMNKARA08875 8.66E 49.33N ad m Aelurillus v insignitus
SMNSAran814 8.64E 49.35N ad m Aelurillus v insignitus
SMNKARA08350 8.66E 49.33N ad m Aelurillus v insignitus
SMNSAran825 8.64E 49.35N ad m Aelurillus v insignitus
SMNKARA08878 8.66E 49.33N jv Aelurillus v insignitus
ZSMARACH00319 12.16E 51.78N ad f Pseudicius encarpatus
ZSMARACH00447 14.25E 51.91N ad m Philaeus chrysops
ZSMARACH00448 14.25E 51.91N jv Philaeus chrysops
ZSMARACH00269 13.15E 52.25N ad m Heliophanus dubius
ZSMARACH00270 13.15E 52.25N ad m Heliophanus dubius
ZFMKTIS2511 13.6E 51.18N ad f Heliophanus dubius
ZFMKTIS18477 13.75E 51.1N ad m Heliophanus flavipes
ZFMKTIS18476 13.75E 51.1N ad m Heliophanus flavipes
ZFMKTIS5026 14.51E 48.19N ad Heliophanus flavipes
ZFMKTIS4986 15.53E 48.39N ad m Heliophanus flavipes
ZFMKTIS2534478 12.38E 51.59N ad f Heliophanus flavipes
SMNKARA08880 8.66E 49.33N ad m Heliophanus flavipes
SMNSAran806 9.74E 48.62N ad f Heliophanus flavipes
ZSMARACH00296 11.72E 51.22N ad m Heliophanus flavipes
ZSMARACH00352 11.8E 52.36N ad m Heliophanus flavipes
ZFMKTIS2534417 10.23E 53.98N ad f Heliophanus flavipes
ZFMKTIS2528521 8.8E 53.71N ad m Heliophanus flavipes
ZFMKTIS7155 10.78E 53.55N ad m Heliophanus flavipes
ZFMKTIS2528522 8.8E 53.71N ad f Heliophanus flavipes
ZSMARACH00077 13.43E 47.77N ad f Heliophanus auratus
ZSMARACH00381 14.14E 52.88N ad m Heliophanus auratus
ZSMARACH00367 11.8E 52.36N ad m Heliophanus auratus
SMNKARA09098 8.22E 48.55N ad f Heliophanus dampfi
SMNSAran1011 8.00E 47.87N ad m Heliophanus dampfi
ZFMKTIS2528587 8.8E 53.71N ad m Heliophanus dampfi
ZFMKTIS19062 9.91E 54.53N ad f Heliophanus dampfi
SMNKARA08870 8.66E 49.33N ad m Heliophanus cupreus
SMNSAran912 12.13E 43.60N ad f Heliophanus cupreus
SMNKARA08871 8.66E 49.33N ad m Heliophanus cupreus
SMNKARA08869 8.66E 49.33N ad m Heliophanus cupreus
ZSMARACH00388 13.24E 52.47N ad m Heliophanus cupreus
ZFMKTIS9641 10.69E 53.79N ad f Heliophanus cupreus
SMNKARA08218 8.58E 48.89N ad m Heliophanus cupreus
ZFMKTIS2339 10.69E 53.79N ad m Heliophanus cupreus
ZFMKTIS2030 8.67E 49.5N ad m Heliophanus cupreus
ZSMARACH00385 14.02E 52.86N ad m Heliophanus cupreus
SMNSAran910 9.84E 48.63N ad f Heliophanus cupreus
ZFMKTIS18471 13.75E 51.1N ad m Heliophanus cupreus
ZFMKTIS2282 10.64E 53.66N ad m Heliophanus cupreus
ZFMKTIS2521 13.52E 51.14N ad m Heliophanus cupreus
ZFMKTIS4966 14.5E 48.19N ad m Heliophanus cupreus
ZFMKTIS4998 15.53E 48.39N ad m Heliophanus cupreus
ZFMKTIS2057 8.67E 49.5N ad f Anyphaena accentuata
ZFMKTIS2531588 12.91E 53.36N jv f Anyphaena accentuata
ZFMKTIS2516842 7.3E 50.69N ad m Anyphaena accentuata
ZFMKTIS2531614 12.87E 53.4N jv f Anyphaena accentuata
ZFMKTIS18462 13.75E 51.1N ad f Anyphaena accentuata
ZSMARACH00106 13.49E 52.48N jv Anyphaena accentuata
ZSMARACH00107 13.49E 52.48N jv Anyphaena accentuata
ZFMKGBOL26197 7.31E 50.69N ad m Anyphaena accentuata
ZFMKTIS2291 9.69E 54.68N ad Anyphaena accentuata
ZFMKTIS2264 10.23E 53.55N ad m Anyphaena accentuata
ZFMKTIS18442 9.46E 53.53N ad f Anyphaena accentuata
ZSMARACH00706 11.62E 51.53N ad f Liocranoeca striata
ZSMARACH00086 8.61E 54.32N ad f Agroeca proxima
ZFMKTIS2714 13.19E 50.5N ad m Agroeca proxima
SMNKARA08330 8.22E 48.55N ad m Agroeca proxima
SMNKARA08331 8.22E 48.55N ad f Agroeca proxima
ZFMKTIS19340 13.19E 50.5N ad m Agroeca proxima
ZFMKTIS2524874 10.15E 52.81N ad f Agroeca brunnea
0.5

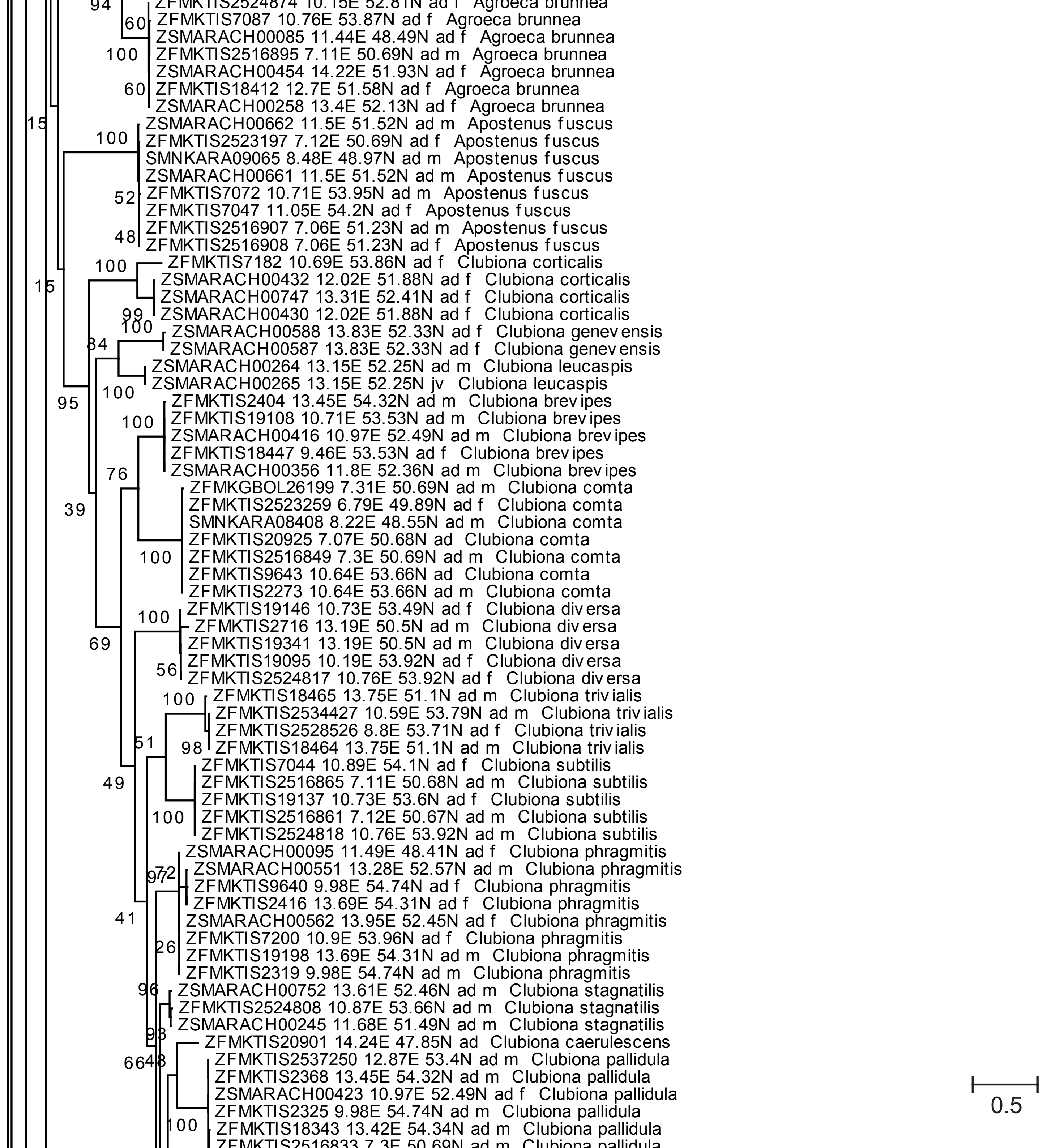

ZFMKTIS2524874 10.15E 52.81N ad f Agroeca brunnea
ZFMKTIS7087 10.76E 53.87N ad f Agroeca brunnea
ZSMARACH00085 11.44E 48.49N ad f Agroeca brunnea
ZFMKTIS2516895 7.11E 50.69N ad m Agroeca brunnea
ZSMARACH00454 14.22E 51.93N ad f Agroeca brunnea
ZFMKTIS18412 12.7E 51.58N ad f Agroeca brunnea
ZSMARACH00258 13.4E 52.13N ad f Agroeca brunnea
ZSMARACH00662 11.5E 51.52N ad m Apostenus fuscus
ZFMKTIS2523197 7.12E 50.69N ad f Apostenus fuscus
SMNKARA09065 8.48E 48.97N ad m Apostenus fuscus
ZSMARACH00661 11.5E 51.52N ad m Apostenus fuscus
ZFMKTIS7072 10.71E 53.95N ad m Apostenus fuscus
ZFMKTIS7047 11.05E 54.2N ad f Apostenus fuscus
ZFMKTIS2516907 7.06E 51.23N ad m Apostenus fuscus
ZFMKTIS2516908 7.06E 51.23N ad f Apostenus fuscus
ZFMKTIS7182 10.69E 53.86N ad f Clubiona corticalis
ZSMARACH00432 12.02E 51.88N ad f Clubiona corticalis
ZSMARACH00747 13.31E 52.41N ad f Clubiona corticalis
ZSMARACH00430 12.02E 51.88N ad f Clubiona corticalis
ZSMARACH00588 13.83E 52.33N ad f Clubiona genevensis
ZSMARACH00587 13.83E 52.33N ad f Clubiona genevensis
ZSMARACH00264 13.15E 52.25N ad m Clubiona leucaspis
ZSMARACH00265 13.15E 52.25N jv Clubiona leucaspis
ZFMKTIS2404 13.45E 54.32N ad m Clubiona brevipes
ZFMKTIS19108 10.71E 53.53N ad m Clubiona brevipes
ZSMARACH00416 10.97E 52.49N ad m Clubiona brevipes
ZFMKTIS18447 9.46E 53.53N ad f Clubiona brevipes
ZSMARACH00356 11.8E 52.36N ad m Clubiona brevipes
ZFMKGBOL26199 7.31E 50.69N ad m Clubiona comta
ZFMKTIS2523259 6.79E 49.89N ad f Clubiona comta
SMNKARA08408 8.22E 48.55N ad m Clubiona comta
ZFMKTIS20925 7.07E 50.68N ad Clubiona comta
ZFMKTIS2516849 7.3E 50.69N ad m Clubiona comta
ZFMKTIS9643 10.64E 53.66N ad Clubiona comta
ZFMKTIS2273 10.64E 53.66N ad m Clubiona comta
ZFMKTIS19146 10.73E 53.49N ad f Clubiona diversa
ZFMKTIS2716 13.19E 50.5N ad m Clubiona diversa
ZFMKTIS19341 13.19E 50.5N ad m Clubiona diversa
ZFMKTIS19095 10.19E 53.92N ad f Clubiona diversa
ZFMKTIS2524817 10.76E 53.92N ad f Clubiona diversa
ZFMKTIS18465 13.75E 51.1N ad m Clubiona trivialis
ZFMKTIS2534427 10.59E 53.79N ad m Clubiona trivialis
ZFMKTIS2528526 8.8E 53.71N ad f Clubiona trivialis
ZFMKTIS18464 13.75E 51.1N ad m Clubiona trivialis
ZFMKTIS7044 10.89E 54.1N ad f Clubiona subtilis
ZFMKTIS2516865 7.11E 50.68N ad m Clubiona subtilis
ZFMKTIS19137 10.73E 53.6N ad f Clubiona subtilis
ZFMKTIS2516861 7.12E 50.67N ad m Clubiona subtilis
ZFMKTIS2524818 10.76E 53.92N ad m Clubiona subtilis
ZSMARACH00095 11.49E 48.41N ad f Clubiona phragmitis
ZSMARACH00551 13.28E 52.57N ad m Clubiona phragmitis
ZFMKTIS9640 9.98E 54.74N ad f Clubiona phragmitis
ZFMKTIS2416 13.69E 54.31N ad f Clubiona phragmitis
ZSMARACH00562 13.95E 52.45N ad f Clubiona phragmitis
ZFMKTIS7200 10.9E 53.96N ad f Clubiona phragmitis
ZFMKTIS19198 13.69E 54.31N ad m Clubiona phragmitis
ZFMKTIS2319 9.98E 54.74N ad m Clubiona phragmitis
ZSMARACH00752 13.61E 52.46N ad m Clubiona stagnatilis
ZFMKTIS2524808 10.87E 53.66N ad m Clubiona stagnatilis
ZSMARACH00245 11.68E 51.49N ad m Clubiona stagnatilis
ZFMKTIS20901 14.24E 47.85N ad Clubiona caerulescens
ZFMKTIS2537250 12.87E 53.4N ad m Clubiona pallidula
ZFMKTIS2368 13.45E 54.32N ad m Clubiona pallidula
ZSMARACH00423 10.97E 52.49N ad f Clubiona pallidula
ZFMKTIS2325 9.98E 54.74N ad m Clubiona pallidula
ZFMKTIS18343 13.42E 54.34N ad m Clubiona pallidula
0.5

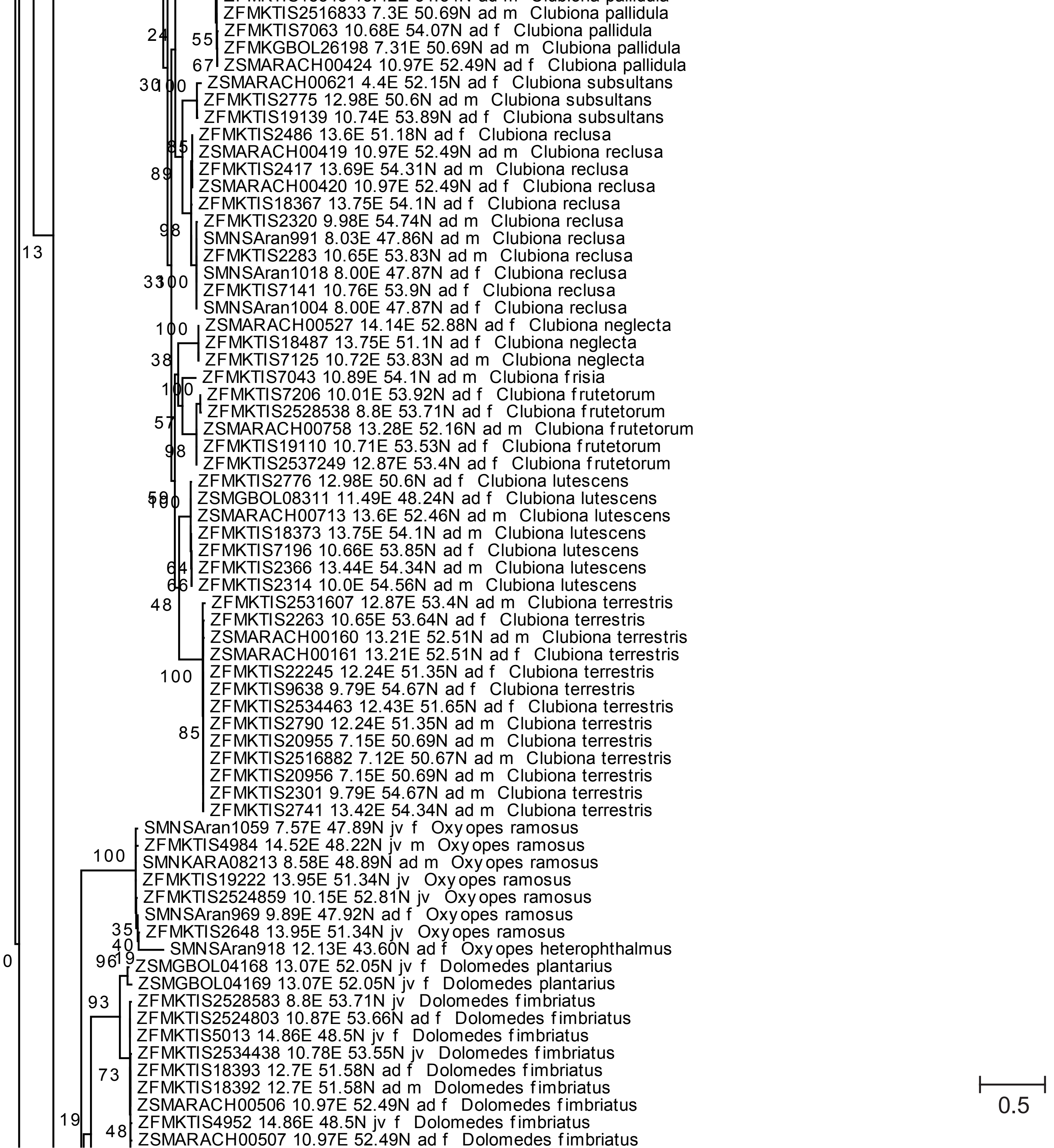
ZFMKTIS2516833 7.3E 50.69N ad m Clubiona pallidula
ZFMKTIS7063 10.68E 54.07N ad f Clubiona pallidula
ZFMKGBOL26198 7.31E 50.69N ad m Clubiona pallidula
ZSMARACH00424 10.97E 52.49N ad f Clubiona pallidula
ZSMARACH00621 4.4E 52.15N ad f Clubiona subsultans
ZFMKTIS2775 12.98E 50.6N ad m Clubiona subsultans
ZFMKTIS19139 10.74E 53.89N ad f Clubiona subsultans
ZFMKTIS2486 13.6E 51.18N ad f Clubiona reclusa
ZSMARACH00419 10.97E 52.49N ad m Clubiona reclusa
ZFMKTIS2417 13.69E 54.31N ad m Clubiona reclusa
ZSMARACH00420 10.97E 52.49N ad f Clubiona reclusa
ZFMKTIS18367 13.75E 54.1N ad f Clubiona reclusa
ZFMKTIS2320 9.98E 54.74N ad m Clubiona reclusa
SMNSAran991 8.03E 47.86N ad m Clubiona reclusa
ZFMKTIS2283 10.65E 53.83N ad m Clubiona reclusa
SMNSAran1018 8.00E 47.87N ad f Clubiona reclusa
ZFMKTIS7141 10.76E 53.9N ad f Clubiona reclusa
SMNSAran1004 8.00E 47.87N ad f Clubiona reclusa
ZSMARACH00527 14.14E 52.88N ad f Clubiona neglecta
ZFMKTIS18487 13.75E 51.1N ad f Clubiona neglecta
ZFMKTIS7125 10.72E 53.83N ad m Clubiona neglecta
ZFMKTIS7043 10.89E 54.1N ad m Clubiona frisia
ZFMKTIS7206 10.01E 53.92N ad f Clubiona frutetorum
ZFMKTIS2528538 8.8E 53.71N ad f Clubiona frutetorum
ZSMARACH00758 13.28E 52.16N ad m Clubiona frutetorum
ZFMKTIS19110 10.71E 53.53N ad f Clubiona frutetorum
ZFMKTIS2537249 12.87E 53.4N ad f Clubiona frutetorum
ZFMKTIS2776 12.98E 50.6N ad f Clubiona lutescens
ZSMGBOL08311 11.49E 48.24N ad f Clubiona lutescens
ZSMARACH00713 13.6E 52.46N ad m Clubiona lutescens
ZFMKTIS18373 13.75E 54.1N ad m Clubiona lutescens
ZFMKTIS7196 10.66E 53.85N ad f Clubiona lutescens
ZFMKTIS2366 13.44E 54.34N ad m Clubiona lutescens
ZFMKTIS2314 10.0E 54.56N ad m Clubiona lutescens
ZFMKTIS2531607 12.87E 53.4N ad m Clubiona terrestris
ZFMKTIS2263 10.65E 53.64N ad f Clubiona terrestris
ZSMARACH00160 13.21E 52.51N ad m Clubiona terrestris
ZSMARACH00161 13.21E 52.51N ad f Clubiona terrestris
ZFMKTIS22245 12.24E 51.35N ad f Clubiona terrestris
ZFMKTIS9638 9.79E 54.67N ad f Clubiona terrestris
ZFMKTIS2534463 12.43E 51.65N ad f Clubiona terrestris
ZFMKTIS2790 12.24E 51.35N ad m Clubiona terrestris
ZFMKTIS20955 7.15E 50.69N ad m Clubiona terrestris
ZFMKTIS2516882 7.12E 50.67N ad m Clubiona terrestris
ZFMKTIS20956 7.15E 50.69N ad m Clubiona terrestris
ZFMKTIS2301 9.79E 54.67N ad m Clubiona terrestris
ZFMKTIS2741 13.42E 54.34N ad m Clubiona terrestris
SMNSAran1059 7.57E 47.89N jv f Oxyopes ramosus
ZFMKTIS4984 14.52E 48.22N jv m Oxyopes ramosus
SMNKARA08213 8.58E 48.89N ad m Oxyopes ramosus
ZFMKTIS19222 13.95E 51.34N jv Oxyopes ramosus
ZFMKTIS2524859 10.15E 52.81N jv Oxyopes ramosus
SMNSAran969 9.89E 47.92N ad f Oxyopes ramosus
ZFMKTIS2648 13.95E 51.34N jv Oxyopes ramosus
SMNSAran918 12.13E 43.60N ad f Oxyopes heterophthalmus
ZSMGBOL04168 13.07E 52.05N jv f Dolomedes plantarius
ZSMGBOL04169 13.07E 52.05N jv f Dolomedes plantarius
ZFMKTIS2528583 8.8E 53.71N jv Dolomedes fimbriatus
ZFMKTIS2524803 10.87E 53.66N ad f Dolomedes fimbriatus
ZFMKTIS5013 14.86E 48.5N jv f Dolomedes fimbriatus
ZFMKTIS2534438 10.78E 53.55N jv Dolomedes fimbriatus
ZFMKTIS18393 12.7E 51.58N ad f Dolomedes fimbriatus
ZFMKTIS18392 12.7E 51.58N ad m Dolomedes fimbriatus
ZSMARACH00506 10.97E 52.49N ad f Dolomedes fimbriatus
ZFMKTIS4952 14.86E 48.5N jv f Dolomedes fimbriatus
ZSMARACH00507 10.97E 52.49N ad f Dolomedes fimbriatus
0.5

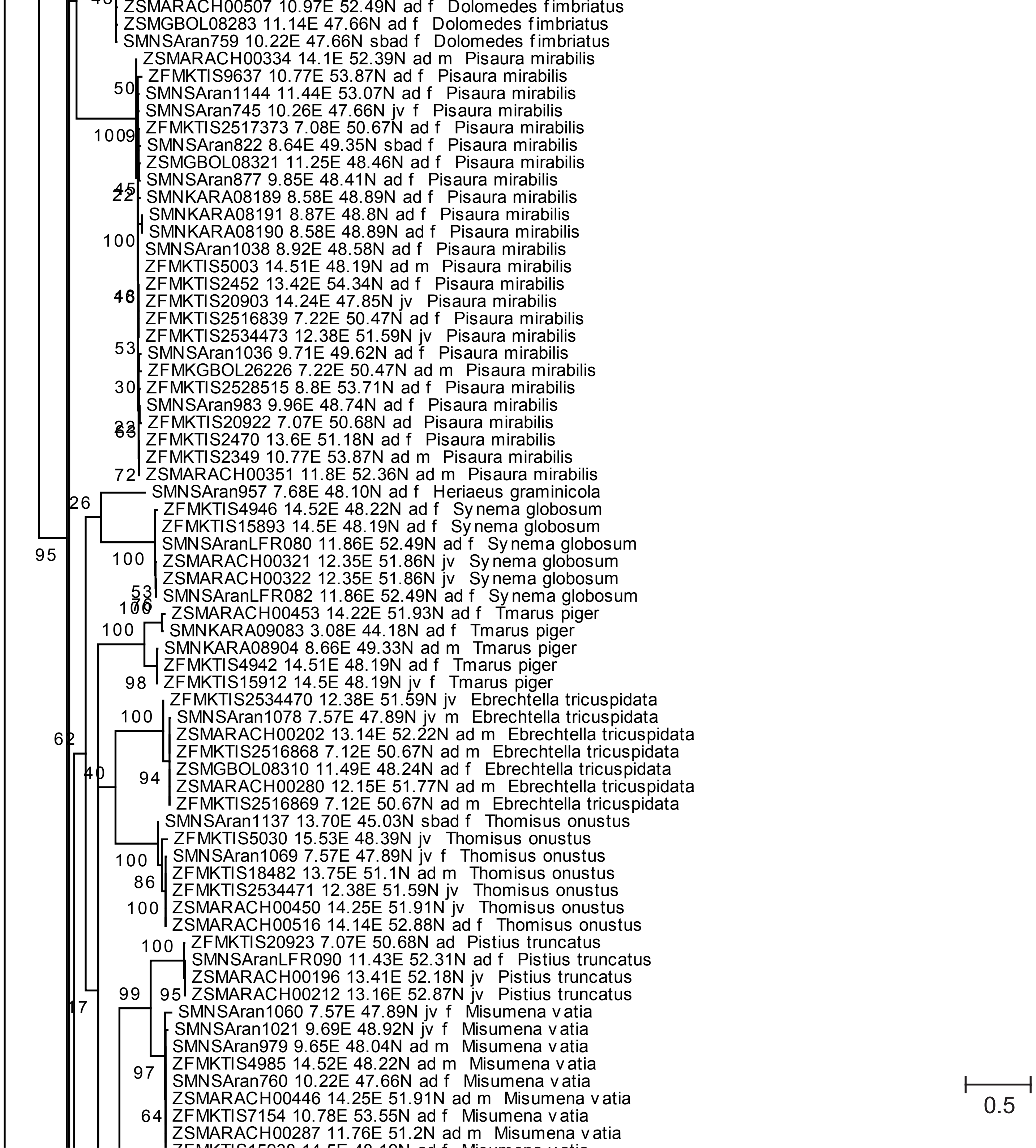
ZSMARACH00507 10.97E 52.49N ad f Dolomedes fimbriatus
ZSMGBOL08283 11.14E 47.66N ad f Dolomedes fimbriatus
SMNSAran759 10.22E 47.66N sbad f Dolomedes fimbriatus
ZSMARACH00334 14.1E 52.39N ad m Pisaura mirabilis
ZFMKTIS9637 10.77E 53.87N ad f Pisaura mirabilis
SMNSAran1144 11.44E 53.07N ad f Pisaura mirabilis
SMNSAran745 10.26E 47.66N jv f Pisaura mirabilis
ZFMKTIS2517373 7.08E 50.67N ad f Pisaura mirabilis
SMNSAran822 8.64E 49.35N sbad f Pisaura mirabilis
ZSMGBOL08321 11.25E 48.46N ad f Pisaura mirabilis
SMNSAran877 9.85E 48.41N ad f Pisaura mirabilis
SMNKARA08189 8.58E 48.89N ad f Pisaura mirabilis
SMNKARA08191 8.87E 48.8N ad f Pisaura mirabilis
SMNKARA08190 8.58E 48.89N ad f Pisaura mirabilis
SMNSAran1038 8.92E 48.58N ad f Pisaura mirabilis
ZFMKTIS5003 14.51E 48.19N ad m Pisaura mirabilis
ZFMKTIS2452 13.42E 54.34N ad f Pisaura mirabilis
ZFMKTIS20903 14.24E 47.85N jv Pisaura mirabilis
ZFMKTIS2516839 7.22E 50.47N ad f Pisaura mirabilis
ZFMKTIS2534473 12.38E 51.59N jv Pisaura mirabilis
SMNSAran1036 9.71E 49.62N ad f Pisaura mirabilis
ZFMKGBOL26226 7.22E 50.47N ad m Pisaura mirabilis
ZFMKTIS2528515 8.8E 53.71N ad f Pisaura mirabilis
SMNSAran983 9.96E 48.74N ad f Pisaura mirabilis
ZFMKTIS20922 7.07E 50.68N ad Pisaura mirabilis
ZFMKTIS2470 13.6E 51.18N ad f Pisaura mirabilis
ZFMKTIS2349 10.77E 53.87N ad m Pisaura mirabilis
ZSMARACH00351 11.8E 52.36N ad m Pisaura mirabilis
SMNSAran957 7.68E 48.10N ad f Heriaeus graminicola
ZFMKTIS4946 14.52E 48.22N ad f Synema globosum
ZFMKTIS15893 14.5E 48.19N ad f Synema globosum
SMNSAranLFR080 11.86E 52.49N ad f Synema globosum
ZSMARACH00321 12.35E 51.86N jv Synema globosum
ZSMARACH00322 12.35E 51.86N jv Synema globosum
SMNSAranLFR082 11.86E 52.49N ad f Synema globosum
ZSMARACH00453 14.22E 51.93N ad f Tmarus piger
SMNKARA09083 3.08E 44.18N ad f Tmarus piger
SMNKARA08904 8.66E 49.33N ad m Tmarus piger
ZFMKTIS4942 14.51E 48.19N ad f Tmarus piger
ZFMKTIS15912 14.5E 48.19N jv f Tmarus piger
ZFMKTIS2534470 12.38E 51.59N jv Ebrechtella tricuspidata
SMNSAran1078 7.57E 47.89N jv m Ebrechtella tricuspidata
ZSMARACH00202 13.14E 52.22N ad m Ebrechtella tricuspidata
ZFMKTIS2516868 7.12E 50.67N ad m Ebrechtella tricuspidata
ZSMGBOL08310 11.49E 48.24N ad f Ebrechtella tricuspidata
ZSMARACH00280 12.15E 51.77N ad m Ebrechtella tricuspidata
ZFMKTIS2516869 7.12E 50.67N ad m Ebrechtella tricuspidata
SMNSAran1137 13.70E 45.03N sbad f Thomisus onustus
ZFMKTIS5030 15.53E 48.39N jv Thomisus onustus
SMNSAran1069 7.57E 47.89N jv f Thomisus onustus
ZFMKTIS18482 13.75E 51.1N ad m Thomisus onustus
ZFMKTIS2534471 12.38E 51.59N jv Thomisus onustus
ZSMARACH00450 14.25E 51.91N jv Thomisus onustus
ZSMARACH00516 14.14E 52.88N ad f Thomisus onustus
ZFMKTIS20923 7.07E 50.68N ad Pistius truncatus
SMNSAranLFR090 11.43E 52.31N ad f Pistius truncatus
ZSMARACH00196 13.41E 52.18N jv Pistius truncatus
ZSMARACH00212 13.16E 52.87N jv Pistius truncatus
SMNSAran1060 7.57E 47.89N jv f Misumena vatia
SMNSAran1021 9.69E 48.92N jv f Misumena vatia
SMNSAran979 9.65E 48.04N ad m Misumena vatia
ZFMKTIS4985 14.52E 48.22N ad m Misumena vatia
SMNSAran760 10.22E 47.66N ad f Misumena vatia
ZSMARACH00446 14.25E 51.91N ad m Misumena vatia
ZFMKTIS7154 10.78E 53.55N ad f Misumena vatia
ZSMARACH00287 11.76E 51.2N ad m Misumena vatia
0.5

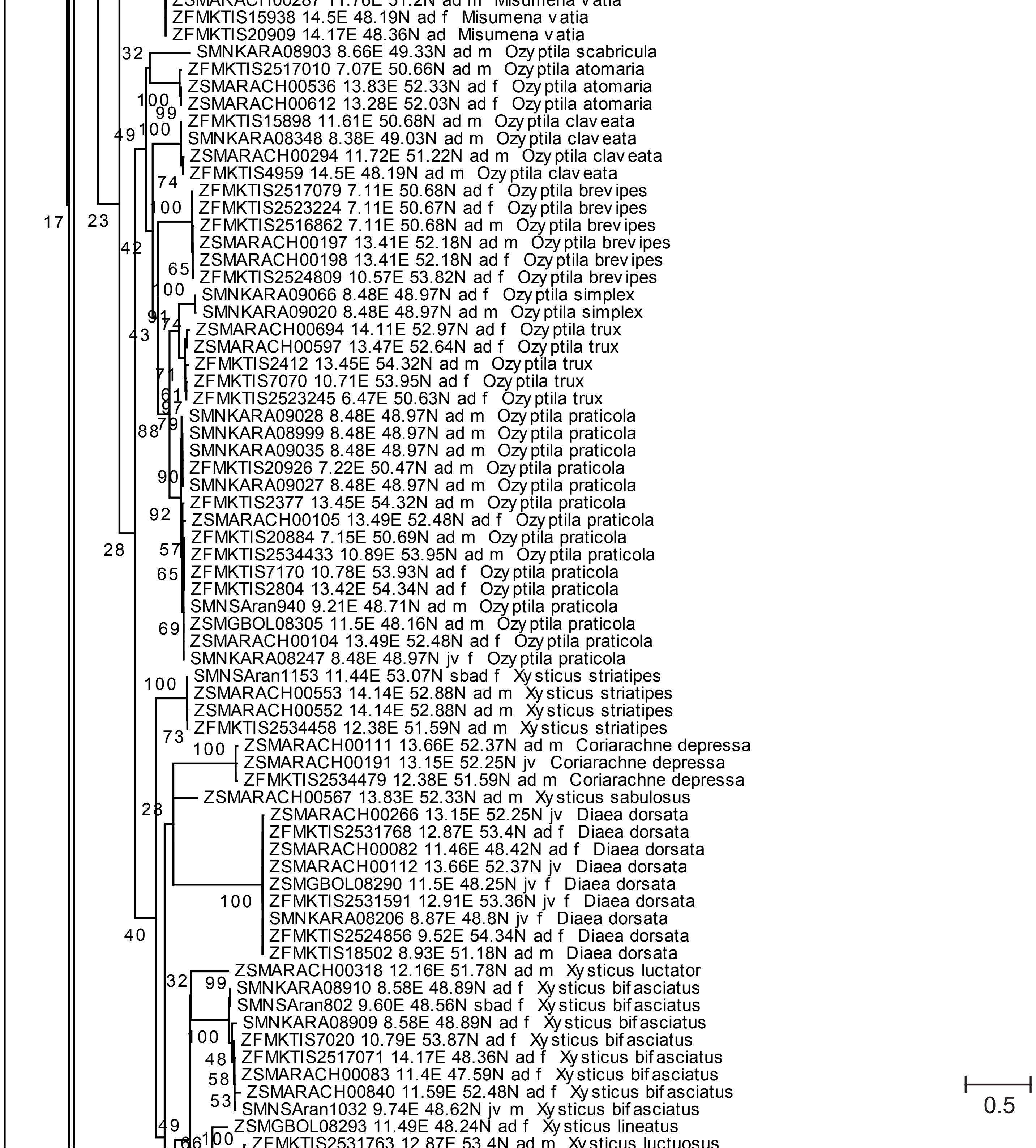

ZFMKTIS15938 14.5E 48.19N ad f Misumena vatia
ZFMKTIS20909 14.17E 48.36N ad Misumena vatia
SMNKARA08903 8.66E 49.33N ad m Ozyptila scabricula
ZFMKTIS2517010 7.07E 50.66N ad m Ozyptila atomaria
ZSMARACH00536 13.83E 52.33N ad f Ozyptila atomaria
ZSMARACH00612 13.28E 52.03N ad f Ozyptila atomaria
ZFMKTIS15898 11.61E 50.68N ad m Ozyptila claveata
SMNKARA08348 8.38E 49.03N ad m Ozyptila claveata
ZSMARACH00294 11.72E 51.22N ad m Ozyptila claveata
ZFMKTIS4959 14.5E 48.19N ad m Ozyptila claveata
ZFMKTIS2517079 7.11E 50.68N ad f Ozyptila brevipes
ZFMKTIS2523224 7.11E 50.67N ad f Ozyptila brevipes
ZFMKTIS2516862 7.11E 50.68N ad m Ozyptila brevipes
ZSMARACH00197 13.41E 52.18N ad m Ozyptila brevipes
ZSMARACH00198 13.41E 52.18N ad f Ozyptila brevipes
ZFMKTIS2524809 10.57E 53.82N ad f Ozyptila brevipes
SMNKARA09066 8.48E 48.97N ad f Ozyptila simplex
SMNKARA09020 8.48E 48.97N ad m Ozyptila simplex
ZSMARACH00694 14.11E 52.97N ad f Ozyptila trux
ZSMARACH00597 13.47E 52.64N ad f Ozyptila trux
ZFMKTIS2412 13.45E 54.32N ad m Ozyptila trux
ZFMKTIS7070 10.71E 53.95N ad f Ozyptila trux
ZFMKTIS2523245 6.47E 50.63N ad f Ozyptila trux
SMNKARA09028 8.48E 48.97N ad m Ozyptila praticola
SMNKARA08999 8.48E 48.97N ad m Ozyptila praticola
SMNKARA09035 8.48E 48.97N ad m Ozyptila praticola
ZFMKTIS20926 7.22E 50.47N ad m Ozyptila praticola
SMNKARA09027 8.48E 48.97N ad m Ozyptila praticola
ZFMKTIS2377 13.45E 54.32N ad m Ozyptila praticola
ZSMARACH00105 13.49E 52.48N ad f Ozyptila praticola
ZFMKTIS20884 7.15E 50.69N ad m Ozyptila praticola
ZFMKTIS2534433 10.89E 53.95N ad m Ozyptila praticola
ZFMKTIS7170 10.78E 53.93N ad f Ozyptila praticola
ZFMKTIS2804 13.42E 54.34N ad f Ozyptila praticola
SMNSAran940 9.21E 48.71N ad m Ozyptila praticola
ZSMGBOL08305 11.5E 48.16N ad m Ozyptila praticola
ZSMARACH00104 13.49E 52.48N ad f Ozyptila praticola
SMNKARA08247 8.48E 48.97N jv f Ozyptila praticola
SMNSAran1153 11.44E 53.07N sbad f Xysticus striatipes
ZSMARACH00553 14.14E 52.88N ad m Xysticus striatipes
ZSMARACH00552 14.14E 52.88N ad m Xysticus striatipes
ZFMKTIS2534458 12.38E 51.59N ad m Xysticus striatipes
ZSMARACH00111 13.66E 52.37N ad m Coriarachne depressa
ZSMARACH00191 13.15E 52.25N jv Coriarachne depressa
ZFMKTIS2534479 12.38E 51.59N ad m Coriarachne depressa
ZSMARACH00567 13.83E 52.33N ad m Xysticus sabulosus
ZSMARACH00266 13.15E 52.25N jv Diaea dorsata
ZFMKTIS2531768 12.87E 53.4N ad f Diaea dorsata
ZSMARACH00082 11.46E 48.42N ad f Diaea dorsata
ZSMARACH00112 13.66E 52.37N jv Diaea dorsata
ZSMGBOL08290 11.5E 48.25N jv f Diaea dorsata
ZFMKTIS2531591 12.91E 53.36N jv f Diaea dorsata
SMNKARA08206 8.87E 48.8N jv f Diaea dorsata
ZFMKTIS2524856 9.52E 54.34N ad f Diaea dorsata
ZFMKTIS18502 8.93E 51.18N ad m Diaea dorsata
ZSMARACH00318 12.16E 51.78N ad m Xysticus luctator
SMNKARA08910 8.58E 48.89N ad f Xysticus bifasciatus
SMNSAran802 9.60E 48.56N sbad f Xysticus bifasciatus
SMNKARA08909 8.58E 48.89N ad f Xysticus bifasciatus
ZFMKTIS7020 10.79E 53.87N ad f Xysticus bifasciatus
ZFMKTIS2517071 14.17E 48.36N ad f Xysticus bifasciatus
ZSMARACH00083 11.4E 47.59N ad f Xysticus bifasciatus
ZSMARACH00840 11.59E 52.48N ad f Xysticus bifasciatus
SMNSAran1032 9.74E 48.62N jv m Xysticus bifasciatus
ZSMGBOL08293 11.49E 48.24N ad f Xysticus lineatus
0.5

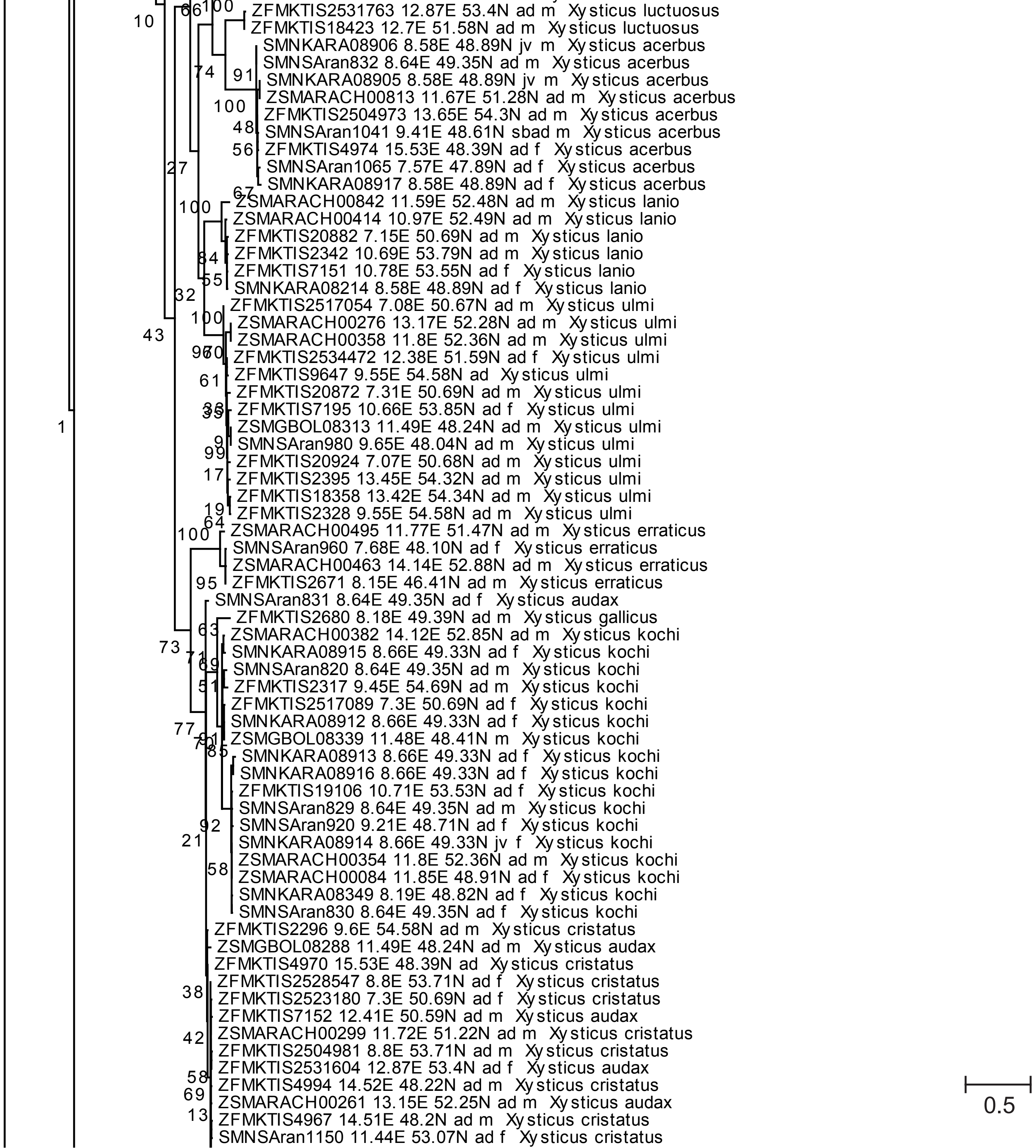
ZFMKTIS2531763 12.87E 53.4N ad m Xysticus luctuosus
ZFMKTIS18423 12.7E 51.58N ad m Xysticus luctuosus
SMNKARA08906 8.58E 48.89N jv m Xysticus acerbus
SMNSAran832 8.64E 49.35N ad m Xysticus acerbus
SMNKARA08905 8.58E 48.89N jv m Xysticus acerbus
ZSMARACH00813 11.67E 51.28N ad m Xysticus acerbus
ZFMKTIS2504973 13.65E 54.3N ad m Xysticus acerbus
SMNSAran1041 9.41E 48.61N sbad m Xysticus acerbus
ZFMKTIS4974 15.53E 48.39N ad f Xysticus acerbus
SMNSAran1065 7.57E 47.89N ad f Xysticus acerbus
SMNKARA08917 8.58E 48.89N ad f Xysticus acerbus
ZSMARACH00842 11.59E 52.48N ad m Xysticus lanio
ZSMARACH00414 10.97E 52.49N ad m Xysticus lanio
ZFMKTIS20882 7.15E 50.69N ad m Xysticus lanio
ZFMKTIS2342 10.69E 53.79N ad m Xysticus lanio
ZFMKTIS7151 10.78E 53.55N ad f Xysticus lanio
SMNKARA08214 8.58E 48.89N ad f Xysticus lanio
ZFMKTIS2517054 7.08E 50.67N ad m Xysticus ulmi
ZSMARACH00276 13.17E 52.28N ad m Xysticus ulmi
ZSMARACH00358 11.8E 52.36N ad m Xysticus ulmi
ZFMKTIS2534472 12.38E 51.59N ad f Xysticus ulmi
ZFMKTIS9647 9.55E 54.58N ad Xysticus ulmi
ZFMKTIS20872 7.31E 50.69N ad m Xysticus ulmi
ZFMKTIS7195 10.66E 53.85N ad f Xysticus ulmi
ZSMGBOL08313 11.49E 48.24N ad m Xysticus ulmi
SMNSAran980 9.65E 48.04N ad m Xysticus ulmi
ZFMKTIS20924 7.07E 50.68N ad m Xysticus ulmi
ZFMKTIS2395 13.45E 54.32N ad m Xysticus ulmi
ZFMKTIS18358 13.42E 54.34N ad m Xysticus ulmi
ZFMKTIS2328 9.55E 54.58N ad m Xysticus ulmi
ZSMARACH00495 11.77E 51.47N ad m Xysticus erraticus
SMNSAran960 7.68E 48.10N ad f Xysticus erraticus
ZSMARACH00463 14.14E 52.88N ad m Xysticus erraticus
ZFMKTIS2671 8.15E 46.41N ad m Xysticus erraticus
SMNSAran831 8.64E 49.35N ad f Xysticus audax
ZFMKTIS2680 8.18E 49.39N ad m Xysticus gallicus
ZSMARACH00382 14.12E 52.85N ad m Xysticus kochi
SMNKARA08915 8.66E 49.33N ad f Xysticus kochi
SMNSAran820 8.64E 49.35N ad m Xysticus kochi
ZFMKTIS2317 9.45E 54.69N ad m Xysticus kochi
ZFMKTIS2517089 7.3E 50.69N ad f Xysticus kochi
SMNKARA08912 8.66E 49.33N ad f Xysticus kochi
ZSMGBOL08339 11.48E 48.41N m Xysticus kochi
SMNKARA08913 8.66E 49.33N ad f Xysticus kochi
SMNKARA08916 8.66E 49.33N ad f Xysticus kochi
ZFMKTIS19106 10.71E 53.53N ad f Xysticus kochi
SMNSAran829 8.64E 49.35N ad m Xysticus kochi
SMNSAran920 9.21E 48.71N ad f Xysticus kochi
SMNKARA08914 8.66E 49.33N jv f Xysticus kochi
ZSMARACH00354 11.8E 52.36N ad m Xysticus kochi
ZSMARACH00084 11.85E 48.91N ad f Xysticus kochi
SMNKARA08349 8.19E 48.82N ad f Xysticus kochi
SMNSAran830 8.64E 49.35N ad f Xysticus kochi
ZFMKTIS2296 9.6E 54.58N ad m Xysticus cristatus
ZSMGBOL08288 11.49E 48.24N ad m Xysticus audax
ZFMKTIS4970 15.53E 48.39N ad Xysticus cristatus
ZFMKTIS2528547 8.8E 53.71N ad f Xysticus cristatus
ZFMKTIS2523180 7.3E 50.69N ad f Xysticus cristatus
ZFMKTIS7152 12.41E 50.59N ad m Xysticus audax
ZSMARACH00299 11.72E 51.22N ad m Xysticus cristatus
ZFMKTIS2504981 8.8E 53.71N ad m Xysticus cristatus
ZFMKTIS2531604 12.87E 53.4N ad f Xysticus audax
ZFMKTIS4994 14.52E 48.22N ad m Xysticus cristatus
ZSMARACH00261 13.15E 52.25N ad m Xysticus audax
ZFMKTIS4967 14.51E 48.2N ad m Xysticus cristatus
SMNSAran1150 11.44E 53.07N ad f Xysticus cristatus
0.5

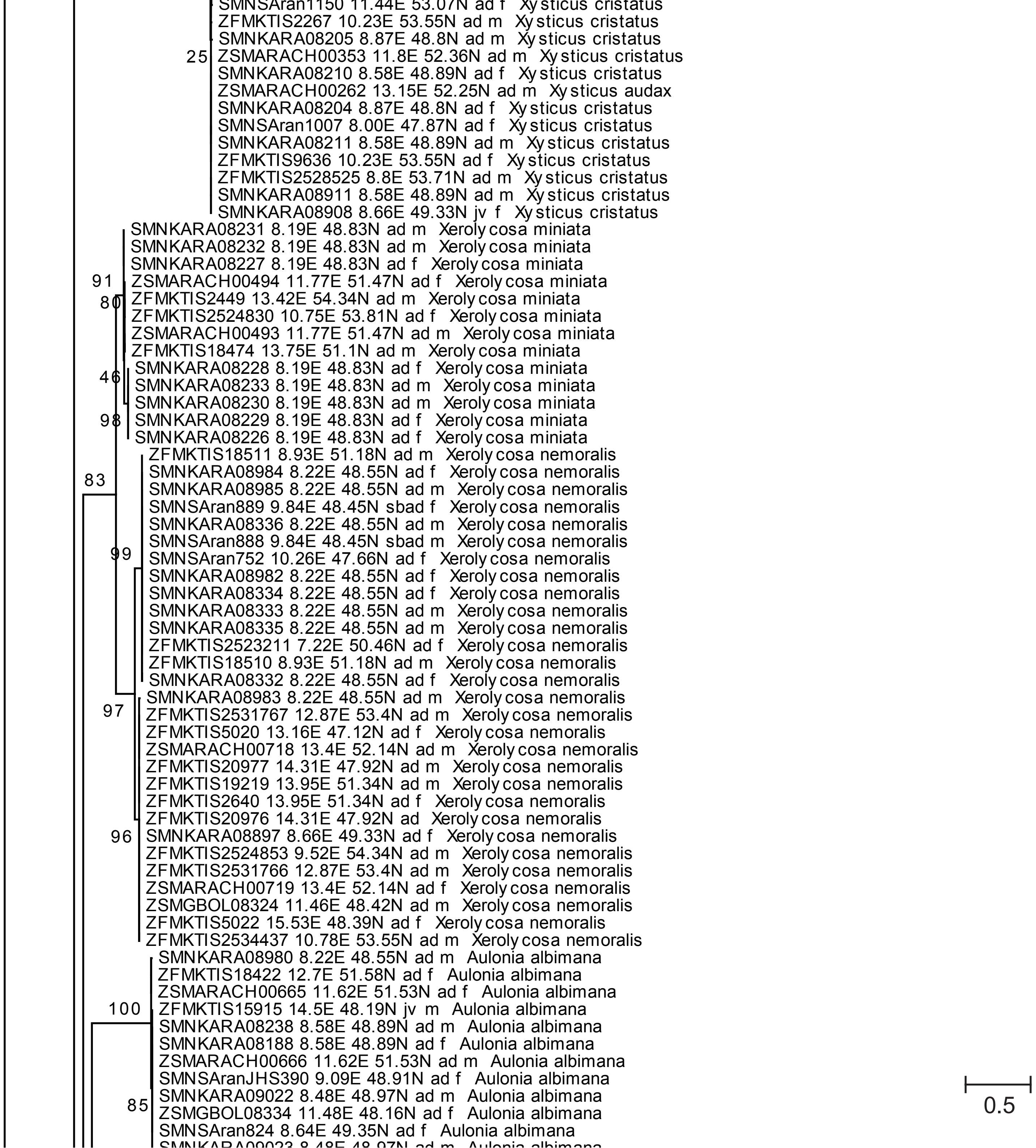

SMNSAran1150 11.44E 53.07N ad f Xysticus cristatus
ZFMKTIS2267 10.23E 53.55N ad m Xysticus cristatus
SMNKARA08205 8.87E 48.8N ad m Xysticus cristatus
25
ZSMARACH00353 11.8E 52.36N ad m Xysticus cristatus
SMNKARA08210 8.58E 48.89N ad f Xysticus cristatus
ZSMARACH00262 13.15E 52.25N ad m Xysticus audax
SMNKARA08204 8.87E 48.8N ad f Xysticus cristatus
SMNSAran1007 8.00E 47.87N ad f Xysticus cristatus
SMNKARA08211 8.58E 48.89N ad m Xysticus cristatus
ZFMKTIS9636 10.23E 53.55N ad f Xysticus cristatus
ZFMKTIS2528525 8.8E 53.71N ad m Xysticus cristatus
SMNKARA08911 8.58E 48.89N ad m Xysticus cristatus
SMNKARA08908 8.66E 49.33N jv f Xysticus cristatus
SMNKARA08231 8.19E 48.83N ad m Xerolycosa miniata
SMNKARA08232 8.19E 48.83N ad m Xerolycosa miniata
SMNKARA08227 8.19E 48.83N ad f Xerolycosa miniata
91
ZSMARACH00494 11.77E 51.47N ad f Xerolycosa miniata
80
ZFMKTIS2449 13.42E 54.34N ad m Xerolycosa miniata
ZFMKTIS2524830 10.75E 53.81N ad f Xerolycosa miniata
ZSMARACH00493 11.77E 51.47N ad m Xerolycosa miniata
ZFMKTIS18474 13.75E 51.1N ad m Xerolycosa miniata
SMNKARA08228 8.19E 48.83N ad f Xerolycosa miniata
46
SMNKARA08233 8.19E 48.83N ad m Xerolycosa miniata
SMNKARA08230 8.19E 48.83N ad m Xerolycosa miniata
98
SMNKARA08229 8.19E 48.83N ad f Xerolycosa miniata
SMNKARA08226 8.19E 48.83N ad f Xerolycosa miniata
ZFMKTIS18511 8.93E 51.18N ad m Xerolycosa nemoralis
SMNKARA08984 8.22E 48.55N ad f Xerolycosa nemoralis
83
SMNKARA08985 8.22E 48.55N ad m Xerolycosa nemoralis
SMNSAran889 9.84E 48.45N sbad f Xerolycosa nemoralis
SMNKARA08336 8.22E 48.55N ad m Xerolycosa nemoralis
SMNSAran888 9.84E 48.45N sbad m Xerolycosa nemoralis
99
SMNSAran752 10.26E 47.66N ad f Xerolycosa nemoralis
SMNKARA08982 8.22E 48.55N ad f Xerolycosa nemoralis
SMNKARA08334 8.22E 48.55N ad f Xerolycosa nemoralis
SMNKARA08333 8.22E 48.55N ad m Xerolycosa nemoralis
SMNKARA08335 8.22E 48.55N ad m Xerolycosa nemoralis
ZFMKTIS2523211 7.22E 50.46N ad f Xerolycosa nemoralis
ZFMKTIS18510 8.93E 51.18N ad m Xerolycosa nemoralis
SMNKARA08332 8.22E 48.55N ad f Xerolycosa nemoralis
SMNKARA08983 8.22E 48.55N ad m Xerolycosa nemoralis
97
ZFMKTIS2531767 12.87E 53.4N ad m Xerolycosa nemoralis
ZFMKTIS5020 13.16E 47.12N ad f Xerolycosa nemoralis
ZSMARACH00718 13.4E 52.14N ad m Xerolycosa nemoralis
ZFMKTIS20977 14.31E 47.92N ad m Xerolycosa nemoralis
ZFMKTIS19219 13.95E 51.34N ad m Xerolycosa nemoralis
ZFMKTIS2640 13.95E 51.34N ad f Xerolycosa nemoralis
ZFMKTIS20976 14.31E 47.92N ad Xerolycosa nemoralis
96
SMNKARA08897 8.66E 49.33N ad f Xerolycosa nemoralis
ZFMKTIS2524853 9.52E 54.34N ad m Xerolycosa nemoralis
ZFMKTIS2531766 12.87E 53.4N ad m Xerolycosa nemoralis
ZSMARACH00719 13.4E 52.14N ad f Xerolycosa nemoralis
ZSMGBOL08324 11.46E 48.42N ad m Xerolycosa nemoralis
ZFMKTIS5022 15.53E 48.39N ad f Xerolycosa nemoralis
ZFMKTIS2534437 10.78E 53.55N ad m Xerolycosa nemoralis
SMNKARA08980 8.22E 48.55N ad m Aulonia albimana
ZFMKTIS18422 12.7E 51.58N ad f Aulonia albimana
ZSMARACH00665 11.62E 51.53N ad f Aulonia albimana
100
ZFMKTIS15915 14.5E 48.19N jv m Aulonia albimana
SMNKARA08238 8.58E 48.89N ad m Aulonia albimana
SMNKARA08188 8.58E 48.89N ad f Aulonia albimana
ZSMARACH00666 11.62E 51.53N ad m Aulonia albimana
SMNSAranJHS390 9.09E 48.91N ad f Aulonia albimana
SMNKARA09022 8.48E 48.97N ad m Aulonia albimana
85
ZSMGBOL08334 11.48E 48.16N ad f Aulonia albimana
SMNSAran824 8.64E 49.35N ad f Aulonia albimana
0.5

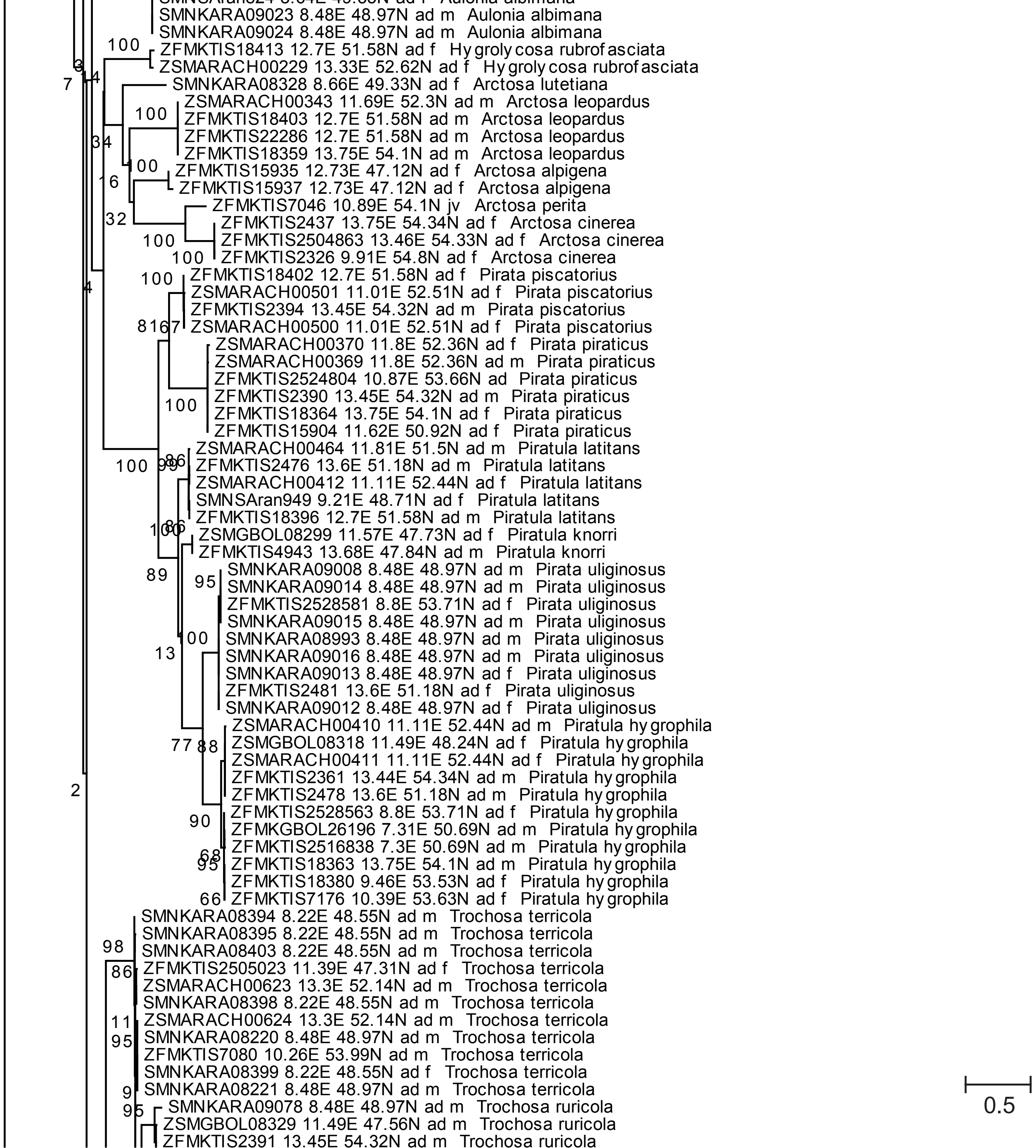
SMNKARA09023 8.48E 48.97N ad m Aulonia albimana
SMNKARA09024 8.48E 48.97N ad m Aulonia albimana
ZFMKTIS18413 12.7E 51.58N ad f Hygrolycosa rubrofasciata
ZSMARACH00229 13.33E 52.62N ad f Hygrolycosa rubrofasciata
SMNKARA08328 8.66E 49.33N ad f Arctosa lutetiana
ZSMARACH00343 11.69E 52.3N ad m Arctosa leopardus
ZFMKTIS18403 12.7E 51.58N ad m Arctosa leopardus
ZFMKTIS22286 12.7E 51.58N ad m Arctosa leopardus
ZFMKTIS18359 13.75E 54.1N ad m Arctosa leopardus
ZFMKTIS15935 12.73E 47.12N ad f Arctosa alpigena
ZFMKTIS15937 12.73E 47.12N ad f Arctosa alpigena
ZFMKTIS7046 10.89E 54.1N jv Arctosa perita
ZFMKTIS2437 13.75E 54.34N ad f Arctosa cinerea
ZFMKTIS2504863 13.46E 54.33N ad f Arctosa cinerea
ZFMKTIS2326 9.91E 54.8N ad f Arctosa cinerea
ZFMKTIS18402 12.7E 51.58N ad f Pirata piscatorius
ZSMARACH00501 11.01E 52.51N ad f Pirata piscatorius
ZFMKTIS2394 13.45E 54.32N ad m Pirata piscatorius
ZSMARACH00500 11.01E 52.51N ad f Pirata piscatorius
ZSMARACH00370 11.8E 52.36N ad f Pirata piraticus
ZSMARACH00369 11.8E 52.36N ad m Pirata piraticus
ZFMKTIS2524804 10.87E 53.66N ad Pirata piraticus
ZFMKTIS2390 13.45E 54.32N ad m Pirata piraticus
ZFMKTIS18364 13.75E 54.1N ad f Pirata piraticus
ZFMKTIS15904 11.62E 50.92N ad f Pirata piraticus
ZSMARACH00464 11.81E 51.5N ad m Piratula latitans
ZFMKTIS2476 13.6E 51.18N ad m Piratula latitans
ZSMARACH00412 11.11E 52.44N ad f Piratula latitans
SMNSAran949 9.21E 48.71N ad f Piratula latitans
ZFMKTIS18396 12.7E 51.58N ad m Piratula latitans
ZSMGBOL08299 11.57E 47.73N ad f Piratula knorri
ZFMKTIS4943 13.68E 47.84N ad m Piratula knorri
SMNKARA09008 8.48E 48.97N ad m Pirata uliginosus
SMNKARA09014 8.48E 48.97N ad m Pirata uliginosus
ZFMKTIS2528581 8.8E 53.71N ad f Pirata uliginosus
SMNKARA09015 8.48E 48.97N ad m Pirata uliginosus
SMNKARA08993 8.48E 48.97N ad m Pirata uliginosus
SMNKARA09016 8.48E 48.97N ad m Pirata uliginosus
SMNKARA09013 8.48E 48.97N ad f Pirata uliginosus
ZFMKTIS2481 13.6E 51.18N ad f Pirata uliginosus
SMNKARA09012 8.48E 48.97N ad f Pirata uliginosus
ZSMARACH00410 11.11E 52.44N ad m Piratula hygrophila
ZSMGBOL08318 11.49E 48.24N ad f Piratula hygrophila
ZSMARACH00411 11.11E 52.44N ad f Piratula hygrophila
ZFMKTIS2361 13.44E 54.34N ad m Piratula hygrophila
ZFMKTIS2478 13.6E 51.18N ad m Piratula hygrophila
ZFMKTIS2528563 8.8E 53.71N ad f Piratula hygrophila
ZFMKGBOL26196 7.31E 50.69N ad m Piratula hygrophila
ZFMKTIS2516838 7.3E 50.69N ad m Piratula hygrophila
ZFMKTIS18363 13.75E 54.1N ad m Piratula hygrophila
ZFMKTIS18380 9.46E 53.53N ad f Piratula hygrophila
ZFMKTIS7176 10.39E 53.63N ad f Piratula hygrophila
SMNKARA08394 8.22E 48.55N ad m Trochosa terricola
SMNKARA08395 8.22E 48.55N ad m Trochosa terricola
SMNKARA08403 8.22E 48.55N ad m Trochosa terricola
ZFMKTIS2505023 11.39E 47.31N ad f Trochosa terricola
ZSMARACH00623 13.3E 52.14N ad m Trochosa terricola
SMNKARA08398 8.22E 48.55N ad m Trochosa terricola
ZSMARACH00624 13.3E 52.14N ad m Trochosa terricola
SMNKARA08220 8.48E 48.97N ad m Trochosa terricola
ZFMKTIS7080 10.26E 53.99N ad m Trochosa terricola
SMNKARA08399 8.22E 48.55N ad f Trochosa terricola
SMNKARA08221 8.48E 48.97N ad m Trochosa terricola
SMNKARA09078 8.48E 48.97N ad m Trochosa ruricola
ZSMGBOL08329 11.49E 47.56N ad m Trochosa ruricola
ZFMKTIS2391 13.45E 54.32N ad m Trochosa ruricola
0.5

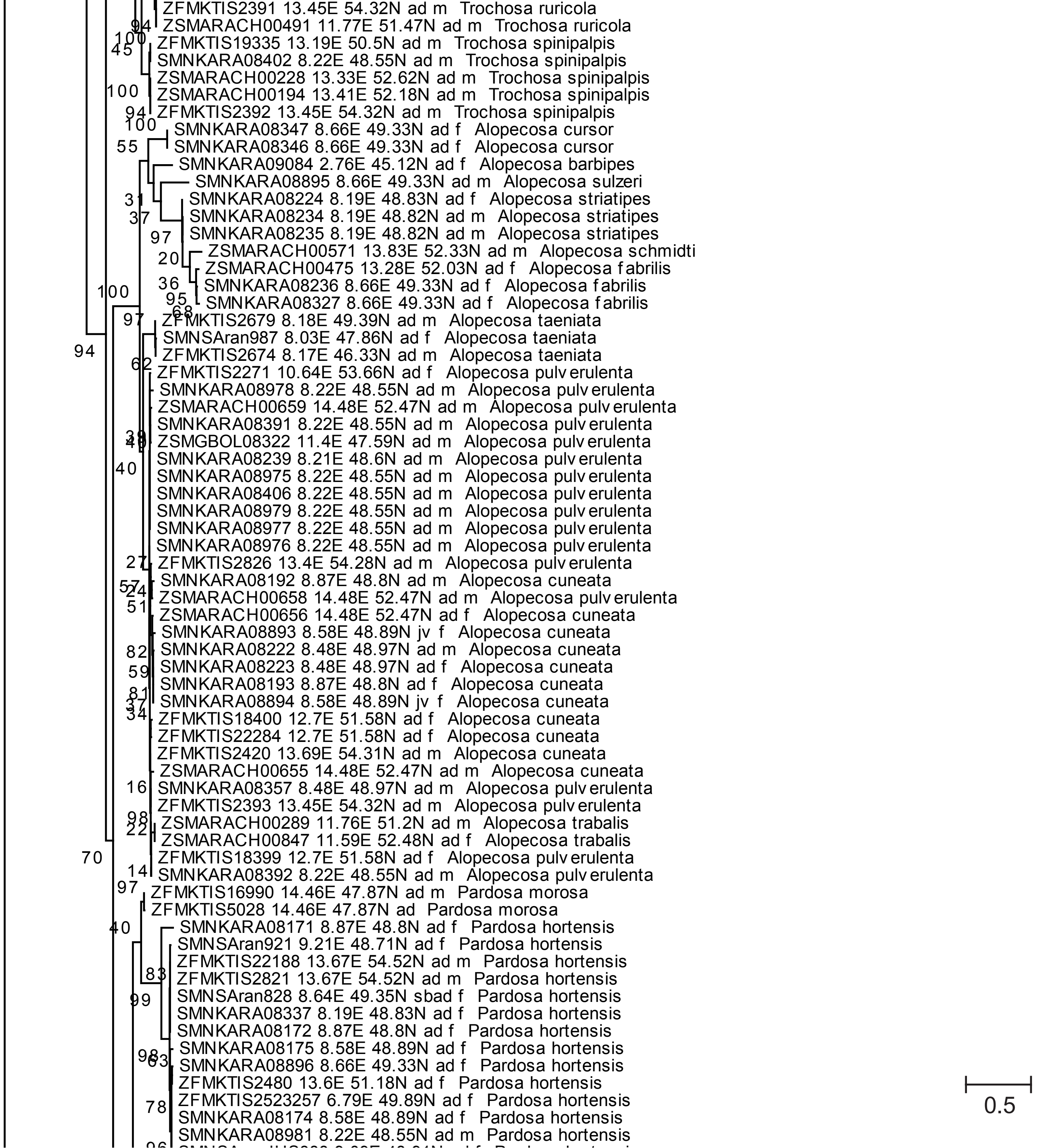
ZFMKTIS2391 13.45E 54.32N ad m Trochosa ruricola
ZSMARACH00491 11.77E 51.47N ad m Trochosa ruricola
ZFMKTIS19335 13.19E 50.5N ad m Trochosa spinipalpis
SMNKARA08402 8.22E 48.55N ad m Trochosa spinipalpis
ZSMARACH00228 13.33E 52.62N ad m Trochosa spinipalpis
ZSMARACH00194 13.41E 52.18N ad m Trochosa spinipalpis
ZFMKTIS2392 13.45E 54.32N ad m Trochosa spinipalpis
SMNKARA08347 8.66E 49.33N ad f Alopecosa cursor
SMNKARA08346 8.66E 49.33N ad f Alopecosa cursor
SMNKARA09084 2.76E 45.12N ad f Alopecosa barbipes
SMNKARA08895 8.66E 49.33N ad m Alopecosa sulzeri
SMNKARA08224 8.19E 48.83N ad f Alopecosa striatipes
SMNKARA08234 8.19E 48.82N ad m Alopecosa striatipes
SMNKARA08235 8.19E 48.82N ad m Alopecosa striatipes
ZSMARACH00571 13.83E 52.33N ad m Alopecosa schmidti
ZSMARACH00475 13.28E 52.03N ad f Alopecosa fabrilis
SMNKARA08236 8.66E 49.33N ad f Alopecosa fabrilis
SMNKARA08327 8.66E 49.33N ad f Alopecosa fabrilis
ZFMKTIS2679 8.18E 49.39N ad m Alopecosa taeniata
SMNSAran987 8.03E 47.86N ad f Alopecosa taeniata
ZFMKTIS2674 8.17E 46.33N ad m Alopecosa taeniata
ZFMKTIS2271 10.64E 53.66N ad f Alopecosa pulverulenta
SMNKARA08978 8.22E 48.55N ad m Alopecosa pulverulenta
ZSMARACH00659 14.48E 52.47N ad m Alopecosa pulverulenta
SMNKARA08391 8.22E 48.55N ad m Alopecosa pulverulenta
ZSMGBOL08322 11.4E 47.59N ad m Alopecosa pulverulenta
SMNKARA08239 8.21E 48.6N ad m Alopecosa pulverulenta
SMNKARA08975 8.22E 48.55N ad m Alopecosa pulverulenta
SMNKARA08406 8.22E 48.55N ad m Alopecosa pulverulenta
SMNKARA08979 8.22E 48.55N ad m Alopecosa pulverulenta
SMNKARA08977 8.22E 48.55N ad m Alopecosa pulverulenta
SMNKARA08976 8.22E 48.55N ad m Alopecosa pulverulenta
ZFMKTIS2826 13.4E 54.28N ad m Alopecosa pulverulenta
SMNKARA08192 8.87E 48.8N ad m Alopecosa cuneata
ZSMARACH00658 14.48E 52.47N ad m Alopecosa pulverulenta
ZSMARACH00656 14.48E 52.47N ad f Alopecosa cuneata
SMNKARA08893 8.58E 48.89N jv f Alopecosa cuneata
SMNKARA08222 8.48E 48.97N ad m Alopecosa cuneata
SMNKARA08223 8.48E 48.97N ad f Alopecosa cuneata
SMNKARA08193 8.87E 48.8N ad f Alopecosa cuneata
SMNKARA08894 8.58E 48.89N jv f Alopecosa cuneata
ZFMKTIS18400 12.7E 51.58N ad f Alopecosa cuneata
ZFMKTIS22284 12.7E 51.58N ad f Alopecosa cuneata
ZFMKTIS2420 13.69E 54.31N ad m Alopecosa cuneata
ZSMARACH00655 14.48E 52.47N ad m Alopecosa cuneata
SMNKARA08357 8.48E 48.97N ad m Alopecosa pulverulenta
ZFMKTIS2393 13.45E 54.32N ad m Alopecosa pulverulenta
ZSMARACH00289 11.76E 51.2N ad m Alopecosa trabalis
ZSMARACH00847 11.59E 52.48N ad f Alopecosa trabalis
ZFMKTIS18399 12.7E 51.58N ad f Alopecosa pulverulenta
SMNKARA08392 8.22E 48.55N ad m Alopecosa pulverulenta
ZFMKTIS16990 14.46E 47.87N ad m Pardosa morosa
ZFMKTIS5028 14.46E 47.87N ad Pardosa morosa
SMNKARA08171 8.87E 48.8N ad f Pardosa hortensis
SMNSAran921 9.21E 48.71N ad f Pardosa hortensis
ZFMKTIS22188 13.67E 54.52N ad m Pardosa hortensis
ZFMKTIS2821 13.67E 54.52N ad m Pardosa hortensis
SMNSAran828 8.64E 49.35N sbad f Pardosa hortensis
SMNKARA08337 8.19E 48.83N ad f Pardosa hortensis
SMNKARA08172 8.87E 48.8N ad f Pardosa hortensis
SMNKARA08175 8.58E 48.89N ad f Pardosa hortensis
SMNKARA08896 8.66E 49.33N ad f Pardosa hortensis
ZFMKTIS2480 13.6E 51.18N ad f Pardosa hortensis
ZFMKTIS2523257 6.79E 49.89N ad f Pardosa hortensis
SMNKARA08174 8.58E 48.89N ad f Pardosa hortensis
SMNKARA08981 8.22E 48.55N ad m Pardosa hortensis
0.5

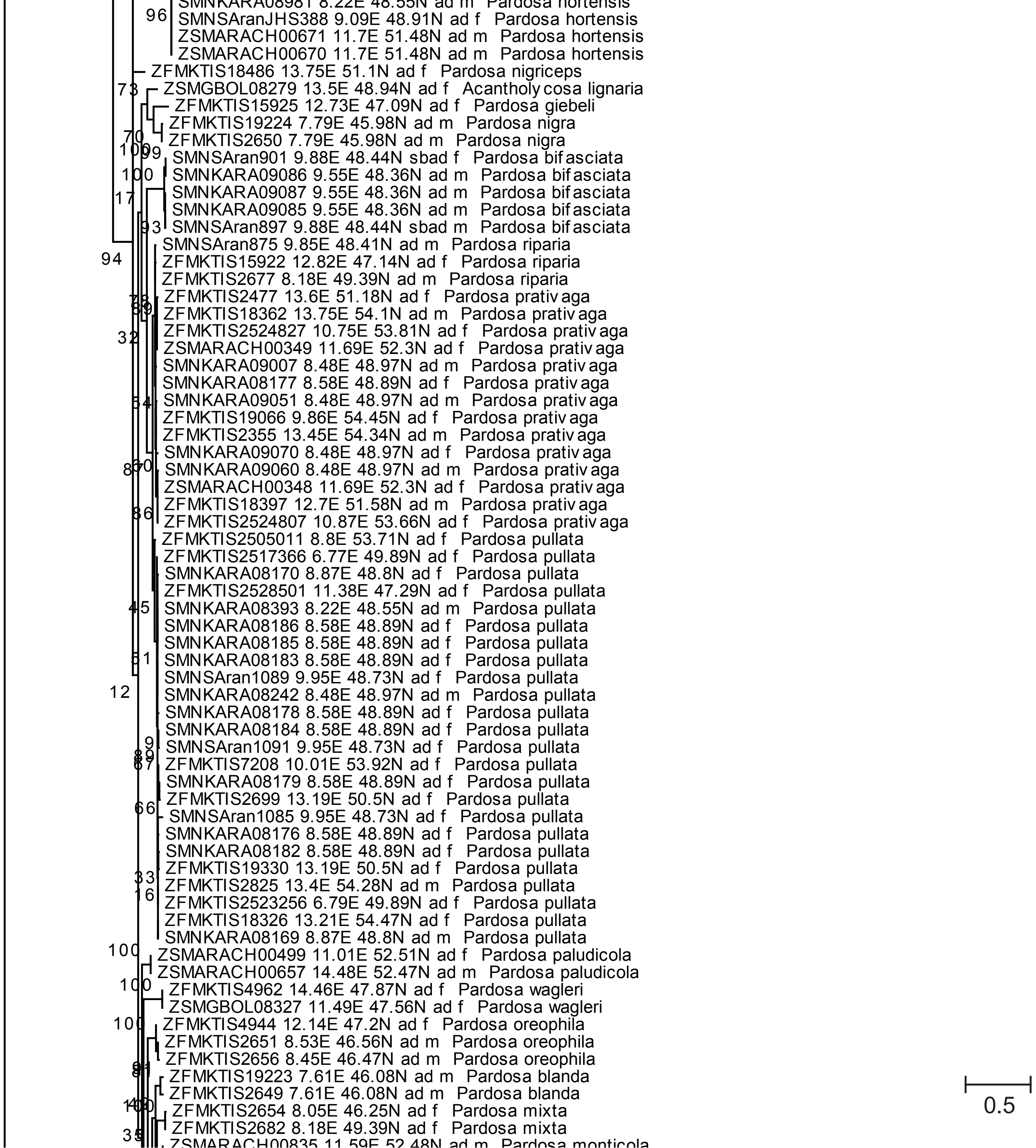
SMNKARA08981 8.22E 48.55N ad m Pardosa hortensis
SMNSAranJHS388 9.09E 48.91N ad f Pardosa hortensis
ZSMARACH00671 11.7E 51.48N ad m Pardosa hortensis
ZSMARACH00670 11.7E 51.48N ad m Pardosa hortensis
ZFMKTIS18486 13.75E 51.1N ad f Pardosa nigriceps
ZSMGBOL08279 13.5E 48.94N ad f Acantholycosa lignaria
ZFMKTIS15925 12.73E 47.09N ad f Pardosa giebeli
ZFMKTIS19224 7.79E 45.98N ad m Pardosa nigra
ZFMKTIS2650 7.79E 45.98N ad m Pardosa nigra
SMNSAran901 9.88E 48.44N sbad f Pardosa bifasciata
SMNKARA09086 9.55E 48.36N ad m Pardosa bifasciata
SMNKARA09087 9.55E 48.36N ad m Pardosa bifasciata
SMNKARA09085 9.55E 48.36N ad m Pardosa bifasciata
SMNSAran897 9.88E 48.44N sbad m Pardosa bifasciata
SMNSAran875 9.85E 48.41N ad m Pardosa riparia
ZFMKTIS15922 12.82E 47.14N ad f Pardosa riparia
ZFMKTIS2677 8.18E 49.39N ad m Pardosa riparia
ZFMKTIS2477 13.6E 51.18N ad f Pardosa prativaga
ZFMKTIS18362 13.75E 54.1N ad m Pardosa prativaga
ZFMKTIS2524827 10.75E 53.81N ad f Pardosa prativaga
ZSMARACH00349 11.69E 52.3N ad f Pardosa prativaga
SMNKARA09007 8.48E 48.97N ad m Pardosa prativaga
SMNKARA08177 8.58E 48.89N ad f Pardosa prativaga
SMNKARA09051 8.48E 48.97N ad m Pardosa prativaga
ZFMKTIS19066 9.86E 54.45N ad f Pardosa prativaga
ZFMKTIS2355 13.45E 54.34N ad m Pardosa prativaga
SMNKARA09070 8.48E 48.97N ad f Pardosa prativaga
SMNKARA09060 8.48E 48.97N ad m Pardosa prativaga
ZSMARACH00348 11.69E 52.3N ad f Pardosa prativaga
ZFMKTIS18397 12.7E 51.58N ad m Pardosa prativaga
ZFMKTIS2524807 10.87E 53.66N ad f Pardosa prativaga
ZFMKTIS2505011 8.8E 53.71N ad f Pardosa pullata
ZFMKTIS2517366 6.77E 49.89N ad f Pardosa pullata
SMNKARA08170 8.87E 48.8N ad f Pardosa pullata
ZFMKTIS2528501 11.38E 47.29N ad f Pardosa pullata
SMNKARA08393 8.22E 48.55N ad m Pardosa pullata
SMNKARA08186 8.58E 48.89N ad f Pardosa pullata
SMNKARA08185 8.58E 48.89N ad f Pardosa pullata
SMNKARA08183 8.58E 48.89N ad f Pardosa pullata
SMNSAran1089 9.95E 48.73N ad f Pardosa pullata
SMNKARA08242 8.48E 48.97N ad m Pardosa pullata
SMNKARA08178 8.58E 48.89N ad f Pardosa pullata
SMNKARA08184 8.58E 48.89N ad f Pardosa pullata
SMNSAran1091 9.95E 48.73N ad f Pardosa pullata
ZFMKTIS7208 10.01E 53.92N ad f Pardosa pullata
SMNKARA08179 8.58E 48.89N ad f Pardosa pullata
ZFMKTIS2699 13.19E 50.5N ad f Pardosa pullata
SMNSAran1085 9.95E 48.73N ad f Pardosa pullata
SMNKARA08176 8.58E 48.89N ad f Pardosa pullata
SMNKARA08182 8.58E 48.89N ad f Pardosa pullata
ZFMKTIS19330 13.19E 50.5N ad f Pardosa pullata
ZFMKTIS2825 13.4E 54.28N ad m Pardosa pullata
ZFMKTIS2523256 6.79E 49.89N ad f Pardosa pullata
ZFMKTIS18326 13.21E 54.47N ad f Pardosa pullata
SMNKARA08169 8.87E 48.8N ad m Pardosa pullata
ZSMARACH00499 11.01E 52.51N ad f Pardosa paludicola
ZSMARACH00657 14.48E 52.47N ad m Pardosa paludicola
ZFMKTIS4962 14.46E 47.87N ad f Pardosa wagleri
ZSMGBOL08327 11.49E 47.56N ad f Pardosa wagleri
ZFMKTIS4944 12.14E 47.2N ad f Pardosa oreophila
ZFMKTIS2651 8.53E 46.56N ad m Pardosa oreophila
ZFMKTIS2656 8.45E 46.47N ad m Pardosa oreophila
ZFMKTIS19223 7.61E 46.08N ad m Pardosa blanda
ZFMKTIS2649 7.61E 46.08N ad m Pardosa blanda
ZFMKTIS2654 8.05E 46.25N ad f Pardosa mixta
ZFMKTIS2682 8.18E 49.39N ad f Pardosa mixta
ZSMARACH00835 11.59E 52.48N ad m Pardosa monticola
0.5

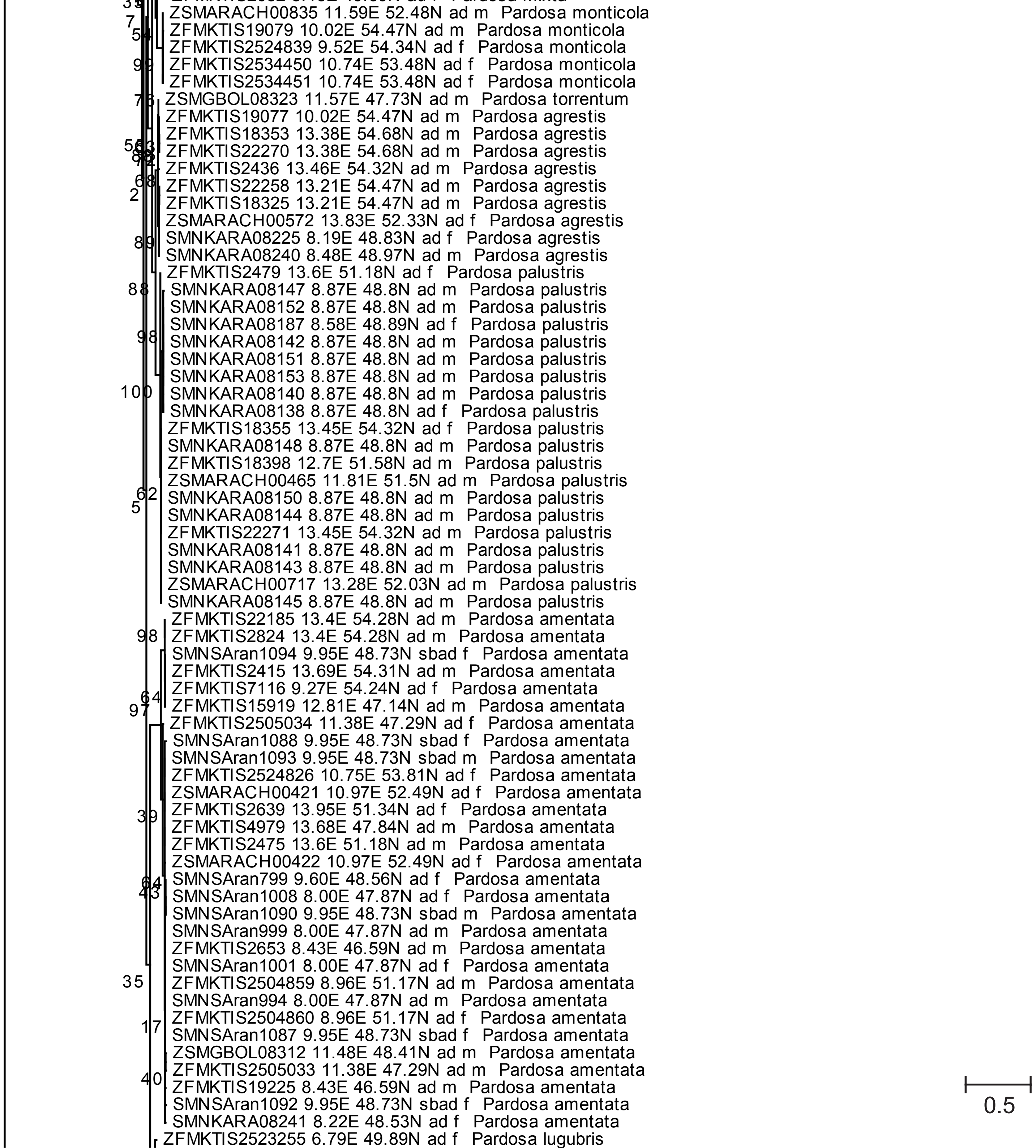
ZSMARACH00835 11.59E 52.48N ad m Pardosa monticola
ZFMKTIS19079 10.02E 54.47N ad m Pardosa monticola
ZFMKTIS2524839 9.52E 54.34N ad f Pardosa monticola
ZFMKTIS2534450 10.74E 53.48N ad f Pardosa monticola
ZFMKTIS2534451 10.74E 53.48N ad f Pardosa monticola
ZSMGBOL08323 11.57E 47.73N ad m Pardosa torrentum
ZFMKTIS19077 10.02E 54.47N ad m Pardosa agrestis
ZFMKTIS18353 13.38E 54.68N ad m Pardosa agrestis
ZFMKTIS22270 13.38E 54.68N ad m Pardosa agrestis
ZFMKTIS2436 13.46E 54.32N ad m Pardosa agrestis
ZFMKTIS22258 13.21E 54.47N ad m Pardosa agrestis
ZFMKTIS18325 13.21E 54.47N ad m Pardosa agrestis
ZSMARACH00572 13.83E 52.33N ad f Pardosa agrestis
SMNKARA08225 8.19E 48.83N ad f Pardosa agrestis
SMNKARA08240 8.48E 48.97N ad m Pardosa agrestis
ZFMKTIS2479 13.6E 51.18N ad f Pardosa palustris
SMNKARA08147 8.87E 48.8N ad m Pardosa palustris
SMNKARA08152 8.87E 48.8N ad m Pardosa palustris
SMNKARA08187 8.58E 48.89N ad f Pardosa palustris
SMNKARA08142 8.87E 48.8N ad m Pardosa palustris
SMNKARA08151 8.87E 48.8N ad m Pardosa palustris
SMNKARA08153 8.87E 48.8N ad m Pardosa palustris
SMNKARA08140 8.87E 48.8N ad m Pardosa palustris
SMNKARA08138 8.87E 48.8N ad f Pardosa palustris
ZFMKTIS18355 13.45E 54.32N ad f Pardosa palustris
SMNKARA08148 8.87E 48.8N ad m Pardosa palustris
ZFMKTIS18398 12.7E 51.58N ad m Pardosa palustris
ZSMARACH00465 11.81E 51.5N ad m Pardosa palustris
SMNKARA08150 8.87E 48.8N ad m Pardosa palustris
SMNKARA08144 8.87E 48.8N ad m Pardosa palustris
ZFMKTIS22271 13.45E 54.32N ad m Pardosa palustris
SMNKARA08141 8.87E 48.8N ad m Pardosa palustris
SMNKARA08143 8.87E 48.8N ad m Pardosa palustris
ZSMARACH00717 13.28E 52.03N ad m Pardosa palustris
SMNKARA08145 8.87E 48.8N ad m Pardosa palustris
ZFMKTIS22185 13.4E 54.28N ad m Pardosa amentata
ZFMKTIS2824 13.4E 54.28N ad m Pardosa amentata
SMNSAran1094 9.95E 48.73N sbad f Pardosa amentata
ZFMKTIS2415 13.69E 54.31N ad m Pardosa amentata
ZFMKTIS7116 9.27E 54.24N ad f Pardosa amentata
ZFMKTIS15919 12.81E 47.14N ad m Pardosa amentata
ZFMKTIS2505034 11.38E 47.29N ad f Pardosa amentata
SMNSAran1088 9.95E 48.73N sbad f Pardosa amentata
SMNSAran1093 9.95E 48.73N sbad m Pardosa amentata
ZFMKTIS2524826 10.75E 53.81N ad f Pardosa amentata
ZSMARACH00421 10.97E 52.49N ad f Pardosa amentata
ZFMKTIS2639 13.95E 51.34N ad f Pardosa amentata
ZFMKTIS4979 13.68E 47.84N ad m Pardosa amentata
ZFMKTIS2475 13.6E 51.18N ad m Pardosa amentata
ZSMARACH00422 10.97E 52.49N ad f Pardosa amentata
SMNSAran799 9.60E 48.56N ad f Pardosa amentata
SMNSAran1008 8.00E 47.87N ad f Pardosa amentata
SMNSAran1090 9.95E 48.73N sbad m Pardosa amentata
SMNSAran999 8.00E 47.87N ad m Pardosa amentata
ZFMKTIS2653 8.43E 46.59N ad m Pardosa amentata
SMNSAran1001 8.00E 47.87N ad f Pardosa amentata
ZFMKTIS2504859 8.96E 51.17N ad m Pardosa amentata
SMNSAran994 8.00E 47.87N ad m Pardosa amentata
ZFMKTIS2504860 8.96E 51.17N ad f Pardosa amentata
SMNSAran1087 9.95E 48.73N sbad f Pardosa amentata
ZSMGBOL08312 11.48E 48.41N ad m Pardosa amentata
ZFMKTIS2505033 11.38E 47.29N ad m Pardosa amentata
ZFMKTIS19225 8.43E 46.59N ad m Pardosa amentata
SMNSAran1092 9.95E 48.73N sbad f Pardosa amentata
SMNKARA08241 8.22E 48.53N ad f Pardosa amentata
ZFMKTIS2523255 6.79E 49.89N ad f Pardosa lugubris
0.5

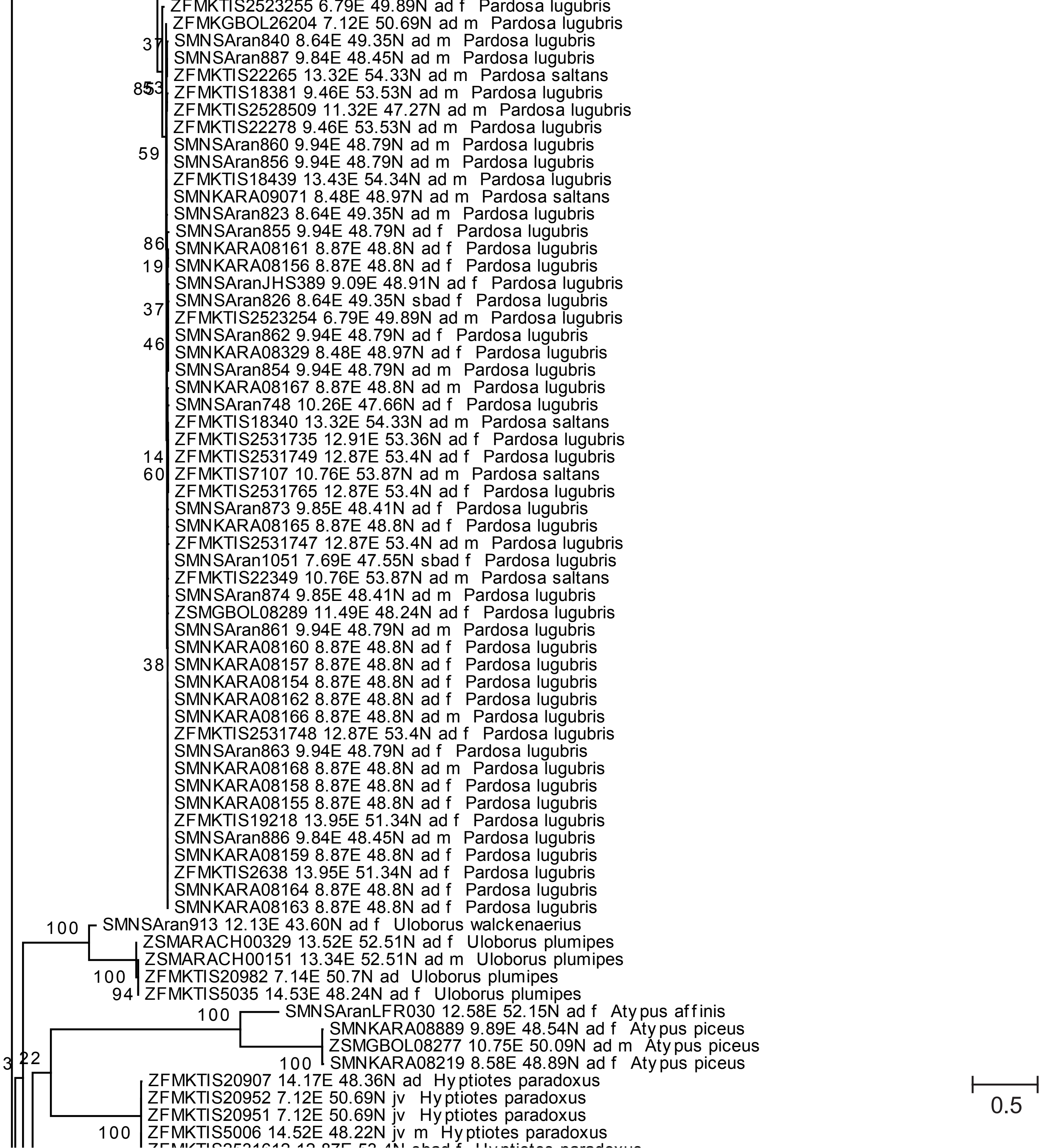

ZFMKTIS2523255 6.79E 49.89N ad f Pardosa lugubris
ZFMKGBOL26204 7.12E 50.69N ad m Pardosa lugubris
37
SMNSAran840 8.64E 49.35N ad m Pardosa lugubris
SMNSAran887 9.84E 48.45N ad m Pardosa lugubris
ZFMKTIS22265 13.32E 54.33N ad m Pardosa saltans
85
53
ZFMKTIS18381 9.46E 53.53N ad m Pardosa lugubris
ZFMKTIS2528509 11.32E 47.27N ad m Pardosa lugubris
ZFMKTIS22278 9.46E 53.53N ad m Pardosa lugubris
59
SMNSAran860 9.94E 48.79N ad m Pardosa lugubris
SMNSAran856 9.94E 48.79N ad m Pardosa lugubris
ZFMKTIS18439 13.43E 54.34N ad m Pardosa lugubris
SMNKARA09071 8.48E 48.97N ad m Pardosa saltans
SMNSAran823 8.64E 49.35N ad m Pardosa lugubris
SMNSAran855 9.94E 48.79N ad f Pardosa lugubris
86
SMNKARA08161 8.87E 48.8N ad f Pardosa lugubris
19
SMNKARA08156 8.87E 48.8N ad f Pardosa lugubris
SMNSAranJHS389 9.09E 48.91N ad f Pardosa lugubris
SMNSAran826 8.64E 49.35N sbad f Pardosa lugubris
37
ZFMKTIS2523254 6.79E 49.89N ad m Pardosa lugubris
SMNSAran862 9.94E 48.79N ad f Pardosa lugubris
46
SMNKARA08329 8.48E 48.97N ad f Pardosa lugubris
SMNSAran854 9.94E 48.79N ad m Pardosa lugubris
SMNKARA08167 8.87E 48.8N ad m Pardosa lugubris
SMNSAran748 10.26E 47.66N ad f Pardosa lugubris
ZFMKTIS18340 13.32E 54.33N ad m Pardosa saltans
ZFMKTIS2531735 12.91E 53.36N ad f Pardosa lugubris
14
ZFMKTIS2531749 12.87E 53.4N ad f Pardosa lugubris
60
ZFMKTIS7107 10.76E 53.87N ad m Pardosa saltans
ZFMKTIS2531765 12.87E 53.4N ad f Pardosa lugubris
SMNSAran873 9.85E 48.41N ad f Pardosa lugubris
SMNKARA08165 8.87E 48.8N ad f Pardosa lugubris
ZFMKTIS2531747 12.87E 53.4N ad m Pardosa lugubris
SMNSAran1051 7.69E 47.55N sbad f Pardosa lugubris
ZFMKTIS22349 10.76E 53.87N ad m Pardosa saltans
SMNSAran874 9.85E 48.41N ad m Pardosa lugubris
ZSMGBOL08289 11.49E 48.24N ad f Pardosa lugubris
SMNSAran861 9.94E 48.79N ad m Pardosa lugubris
SMNKARA08160 8.87E 48.8N ad f Pardosa lugubris
38
SMNKARA08157 8.87E 48.8N ad f Pardosa lugubris
SMNKARA08154 8.87E 48.8N ad f Pardosa lugubris
SMNKARA08162 8.87E 48.8N ad f Pardosa lugubris
SMNKARA08166 8.87E 48.8N ad m Pardosa lugubris
ZFMKTIS2531748 12.87E 53.4N ad f Pardosa lugubris
SMNSAran863 9.94E 48.79N ad f Pardosa lugubris
SMNKARA08168 8.87E 48.8N ad m Pardosa lugubris
SMNKARA08158 8.87E 48.8N ad f Pardosa lugubris
SMNKARA08155 8.87E 48.8N ad f Pardosa lugubris
ZFMKTIS19218 13.95E 51.34N ad f Pardosa lugubris
SMNSAran886 9.84E 48.45N ad m Pardosa lugubris
SMNKARA08159 8.87E 48.8N ad f Pardosa lugubris
ZFMKTIS2638 13.95E 51.34N ad f Pardosa lugubris
SMNKARA08164 8.87E 48.8N ad f Pardosa lugubris
SMNKARA08163 8.87E 48.8N ad f Pardosa lugubris
100
SMNSAran913 12.13E 43.60N ad f Uloborus walckenaerius
ZSMARACH00329 13.52E 52.51N ad f Uloborus plumipes
ZSMARACH00151 13.34E 52.51N ad m Uloborus plumipes
100
ZFMKTIS20982 7.14E 50.7N ad Uloborus plumipes
94
ZFMKTIS5035 14.53E 48.24N ad f Uloborus plumipes
100
SMNSAranLFR030 12.58E 52.15N ad f Atypus affinis
SMNKARA08889 9.89E 48.54N ad f Atypus piceus
ZSMGBOL08277 10.75E 50.09N ad m Atypus piceus
100
SMNKARA08219 8.58E 48.89N ad f Atypus piceus
3
22
ZFMKTIS20907 14.17E 48.36N ad Hyptiotes paradoxus
ZFMKTIS20952 7.12E 50.69N jv Hyptiotes paradoxus
ZFMKTIS20951 7.12E 50.69N jv Hyptiotes paradoxus
100
ZFMKTIS5006 14.52E 48.22N jv m Hyptiotes paradoxus
0.5

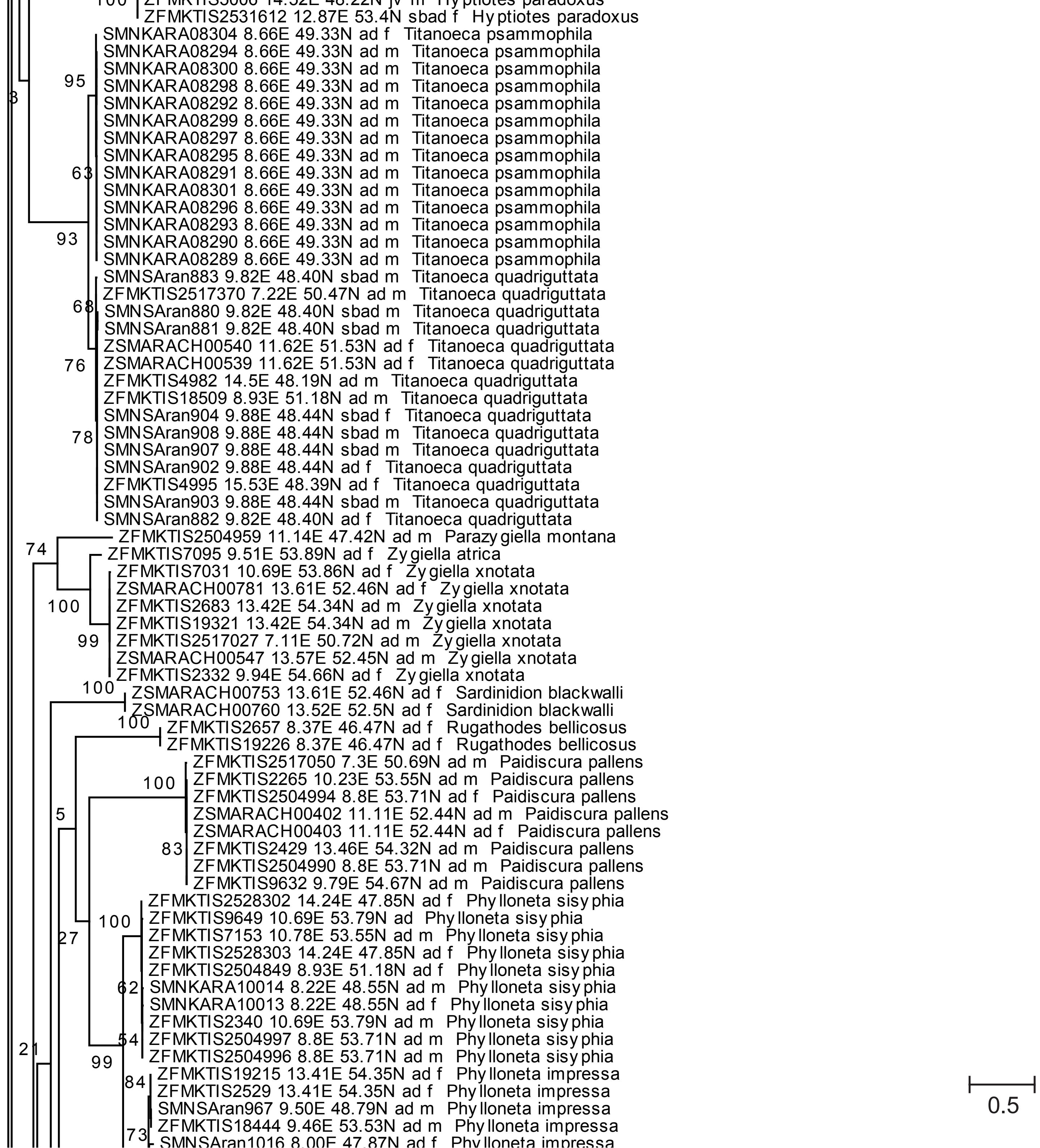

100
ZFMKTIS2531612 12.87E 53.4N sbad f Hyptiotes paradoxus
SMNKARA08304 8.66E 49.33N ad f Titanoeca psammophila
SMNKARA08294 8.66E 49.33N ad m Titanoeca psammophila
SMNKARA08300 8.66E 49.33N ad m Titanoeca psammophila
95
SMNKARA08298 8.66E 49.33N ad m Titanoeca psammophila
8
SMNKARA08292 8.66E 49.33N ad m Titanoeca psammophila
SMNKARA08299 8.66E 49.33N ad m Titanoeca psammophila
SMNKARA08297 8.66E 49.33N ad m Titanoeca psammophila
SMNKARA08295 8.66E 49.33N ad m Titanoeca psammophila
63
SMNKARA08291 8.66E 49.33N ad m Titanoeca psammophila
SMNKARA08301 8.66E 49.33N ad m Titanoeca psammophila
SMNKARA08296 8.66E 49.33N ad m Titanoeca psammophila
SMNKARA08293 8.66E 49.33N ad m Titanoeca psammophila
93
SMNKARA08290 8.66E 49.33N ad m Titanoeca psammophila
SMNKARA08289 8.66E 49.33N ad m Titanoeca psammophila
SMNSAran883 9.82E 48.40N sbad m Titanoeca quadriguttata
ZFMKTIS2517370 7.22E 50.47N ad m Titanoeca quadriguttata
68
SMNSAran880 9.82E 48.40N sbad m Titanoeca quadriguttata
SMNSAran881 9.82E 48.40N sbad m Titanoeca quadriguttata
ZSMARACH00540 11.62E 51.53N ad f Titanoeca quadriguttata
76
ZSMARACH00539 11.62E 51.53N ad f Titanoeca quadriguttata
ZFMKTIS4982 14.5E 48.19N ad m Titanoeca quadriguttata
ZFMKTIS18509 8.93E 51.18N ad m Titanoeca quadriguttata
SMNSAran904 9.88E 48.44N sbad f Titanoeca quadriguttata
SMNSAran908 9.88E 48.44N sbad m Titanoeca quadriguttata
78
SMNSAran907 9.88E 48.44N sbad m Titanoeca quadriguttata
SMNSAran902 9.88E 48.44N ad f Titanoeca quadriguttata
ZFMKTIS4995 15.53E 48.39N ad f Titanoeca quadriguttata
SMNSAran903 9.88E 48.44N sbad m Titanoeca quadriguttata
SMNSAran882 9.82E 48.40N ad f Titanoeca quadriguttata
ZFMKTIS2504959 11.14E 47.42N ad m Parazygiella montana
74
ZFMKTIS7095 9.51E 53.89N ad f Zygiella atrica
ZFMKTIS7031 10.69E 53.86N ad f Zygiella xnotata
ZSMARACH00781 13.61E 52.46N ad f Zygiella xnotata
100
ZFMKTIS2683 13.42E 54.34N ad m Zygiella xnotata
ZFMKTIS19321 13.42E 54.34N ad m Zygiella xnotata
99
ZFMKTIS2517027 7.11E 50.72N ad m Zygiella xnotata
ZSMARACH00547 13.57E 52.45N ad m Zygiella xnotata
ZFMKTIS2332 9.94E 54.66N ad f Zygiella xnotata
100
ZSMARACH00753 13.61E 52.46N ad f Sardinidion blackwalli
ZSMARACH00760 13.52E 52.5N ad f Sardinidion blackwalli
100
ZFMKTIS2657 8.37E 46.47N ad f Rugathodes bellicosus
ZFMKTIS19226 8.37E 46.47N ad f Rugathodes bellicosus
ZFMKTIS2517050 7.3E 50.69N ad m Paidiscura pallens
100
ZFMKTIS2265 10.23E 53.55N ad m Paidiscura pallens
ZFMKTIS2504994 8.8E 53.71N ad f Paidiscura pallens
ZSMARACH00402 11.11E 52.44N ad m Paidiscura pallens
5
ZSMARACH00403 11.11E 52.44N ad f Paidiscura pallens
83
ZFMKTIS2429 13.46E 54.32N ad m Paidiscura pallens
ZFMKTIS2504990 8.8E 53.71N ad m Paidiscura pallens
ZFMKTIS9632 9.79E 54.67N ad m Paidiscura pallens
ZFMKTIS2528302 14.24E 47.85N ad f Phylloneta sisyphia
100
ZFMKTIS9649 10.69E 53.79N ad Phylloneta sisyphia
ZFMKTIS7153 10.78E 53.55N ad m Phylloneta sisyphia
27
ZFMKTIS2528303 14.24E 47.85N ad f Phylloneta sisyphia
ZFMKTIS2504849 8.93E 51.18N ad f Phylloneta sisyphia
62
SMNKARA10014 8.22E 48.55N ad m Phylloneta sisyphia
SMNKARA10013 8.22E 48.55N ad f Phylloneta sisyphia
ZFMKTIS2340 10.69E 53.79N ad m Phylloneta sisyphia
54
ZFMKTIS2504997 8.8E 53.71N ad m Phylloneta sisyphia
21
ZFMKTIS2504996 8.8E 53.71N ad m Phylloneta sisyphia
99
ZFMKTIS19215 13.41E 54.35N ad f Phylloneta impressa
84
ZFMKTIS2529 13.41E 54.35N ad f Phylloneta impressa
SMNSAran967 9.50E 48.79N ad m Phylloneta impressa
ZFMKTIS18444 9.46E 53.53N ad m Phylloneta impressa
73
SMNSAran1016 8.00E 47.87N ad f Phylloneta impressa
0.5

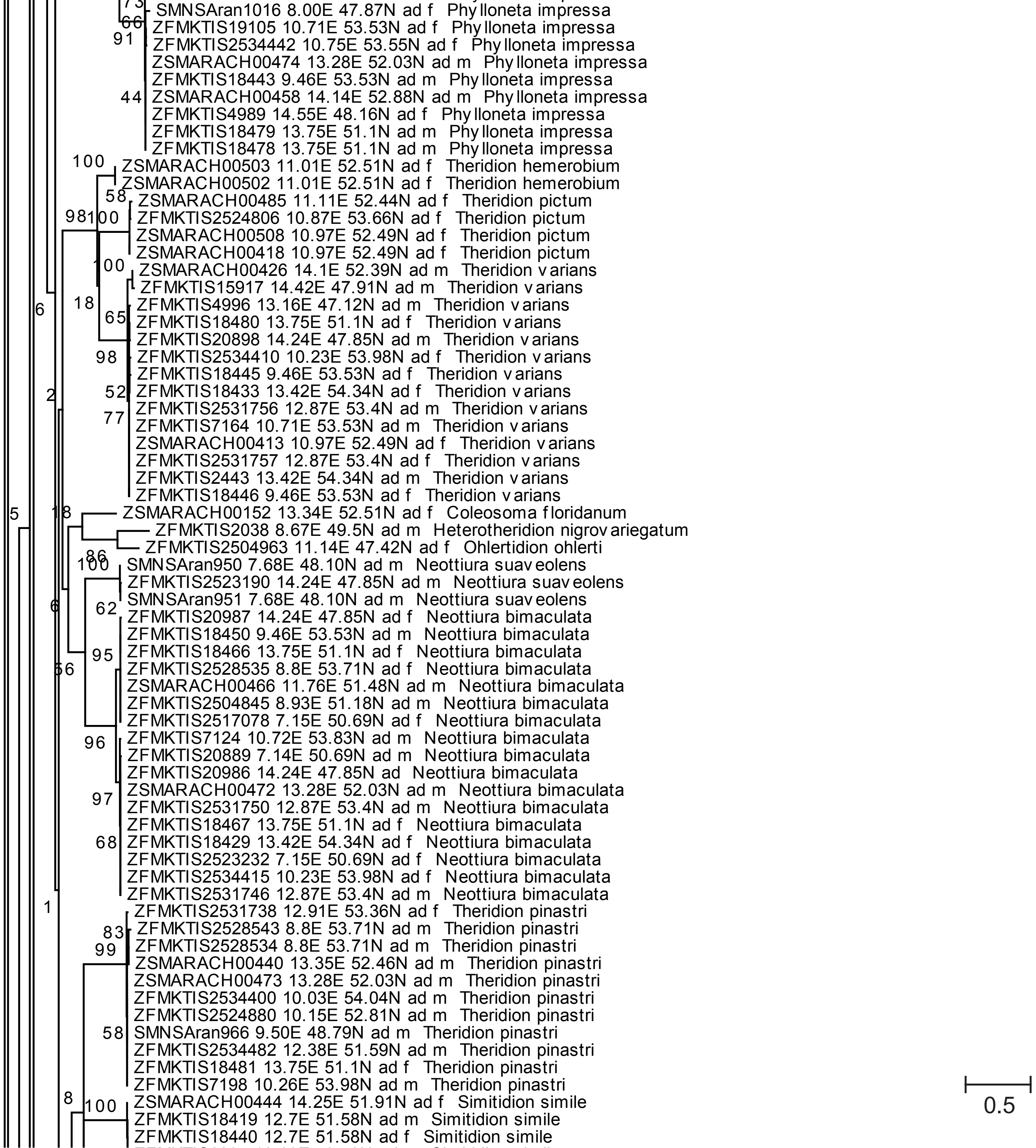
SMNSAran1016 8.00E 47.87N ad f Phylloneta impressa
ZFMKTIS19105 10.71E 53.53N ad f Phylloneta impressa
ZFMKTIS2534442 10.75E 53.55N ad f Phylloneta impressa
ZSMARACH00474 13.28E 52.03N ad m Phylloneta impressa
ZFMKTIS18443 9.46E 53.53N ad m Phylloneta impressa
ZSMARACH00458 14.14E 52.88N ad m Phylloneta impressa
ZFMKTIS4989 14.55E 48.16N ad f Phylloneta impressa
ZFMKTIS18479 13.75E 51.1N ad m Phylloneta impressa
ZFMKTIS18478 13.75E 51.1N ad m Phylloneta impressa
ZSMARACH00503 11.01E 52.51N ad f Theridion hemerobium
ZSMARACH00502 11.01E 52.51N ad f Theridion hemerobium
ZSMARACH00485 11.11E 52.44N ad f Theridion pictum
ZFMKTIS2524806 10.87E 53.66N ad f Theridion pictum
ZSMARACH00508 10.97E 52.49N ad f Theridion pictum
ZSMARACH00418 10.97E 52.49N ad f Theridion pictum
ZSMARACH00426 14.1E 52.39N ad m Theridion varians
ZFMKTIS15917 14.42E 47.91N ad m Theridion varians
ZFMKTIS4996 13.16E 47.12N ad m Theridion varians
ZFMKTIS18480 13.75E 51.1N ad f Theridion varians
ZFMKTIS20898 14.24E 47.85N ad m Theridion varians
ZFMKTIS2534410 10.23E 53.98N ad f Theridion varians
ZFMKTIS18445 9.46E 53.53N ad f Theridion varians
ZFMKTIS18433 13.42E 54.34N ad f Theridion varians
ZFMKTIS2531756 12.87E 53.4N ad m Theridion varians
ZFMKTIS7164 10.71E 53.53N ad m Theridion varians
ZSMARACH00413 10.97E 52.49N ad f Theridion varians
ZFMKTIS2531757 12.87E 53.4N ad f Theridion varians
ZFMKTIS2443 13.42E 54.34N ad m Theridion varians
ZFMKTIS18446 9.46E 53.53N ad f Theridion varians
ZSMARACH00152 13.34E 52.51N ad f Coleosoma floridanum
ZFMKTIS2038 8.67E 49.5N ad m Heterotheridion nigrovariegatum
ZFMKTIS2504963 11.14E 47.42N ad f Ohlertidion ohlerti
SMNSAran950 7.68E 48.10N ad m Neottiura suaveolens
ZFMKTIS2523190 14.24E 47.85N ad m Neottiura suaveolens
SMNSAran951 7.68E 48.10N ad m Neottiura suaveolens
ZFMKTIS20987 14.24E 47.85N ad f Neottiura bimaculata
ZFMKTIS18450 9.46E 53.53N ad m Neottiura bimaculata
ZFMKTIS18466 13.75E 51.1N ad f Neottiura bimaculata
ZFMKTIS2528535 8.8E 53.71N ad f Neottiura bimaculata
ZSMARACH00466 11.76E 51.48N ad m Neottiura bimaculata
ZFMKTIS2504845 8.93E 51.18N ad m Neottiura bimaculata
ZFMKTIS2517078 7.15E 50.69N ad f Neottiura bimaculata
ZFMKTIS7124 10.72E 53.83N ad m Neottiura bimaculata
ZFMKTIS20889 7.14E 50.69N ad m Neottiura bimaculata
ZFMKTIS20986 14.24E 47.85N ad Neottiura bimaculata
ZSMARACH00472 13.28E 52.03N ad m Neottiura bimaculata
ZFMKTIS2531750 12.87E 53.4N ad m Neottiura bimaculata
ZFMKTIS18467 13.75E 51.1N ad f Neottiura bimaculata
ZFMKTIS18429 13.42E 54.34N ad f Neottiura bimaculata
ZFMKTIS2523232 7.15E 50.69N ad f Neottiura bimaculata
ZFMKTIS2534415 10.23E 53.98N ad f Neottiura bimaculata
ZFMKTIS2531746 12.87E 53.4N ad m Neottiura bimaculata
ZFMKTIS2531738 12.91E 53.36N ad f Theridion pinastri
ZFMKTIS2528543 8.8E 53.71N ad m Theridion pinastri
ZFMKTIS2528534 8.8E 53.71N ad m Theridion pinastri
ZSMARACH00440 13.35E 52.46N ad m Theridion pinastri
ZSMARACH00473 13.28E 52.03N ad m Theridion pinastri
ZFMKTIS2534400 10.03E 54.04N ad m Theridion pinastri
ZFMKTIS2524880 10.15E 52.81N ad m Theridion pinastri
SMNSAran966 9.50E 48.79N ad m Theridion pinastri
ZFMKTIS2534482 12.38E 51.59N ad m Theridion pinastri
ZFMKTIS18481 13.75E 51.1N ad f Theridion pinastri
ZFMKTIS7198 10.26E 53.98N ad m Theridion pinastri
ZSMARACH00444 14.25E 51.91N ad f Simitidion simile
ZFMKTIS18419 12.7E 51.58N ad m Simitidion simile
ZFMKTIS18440 12.7E 51.58N ad f Simitidion simile
0.5

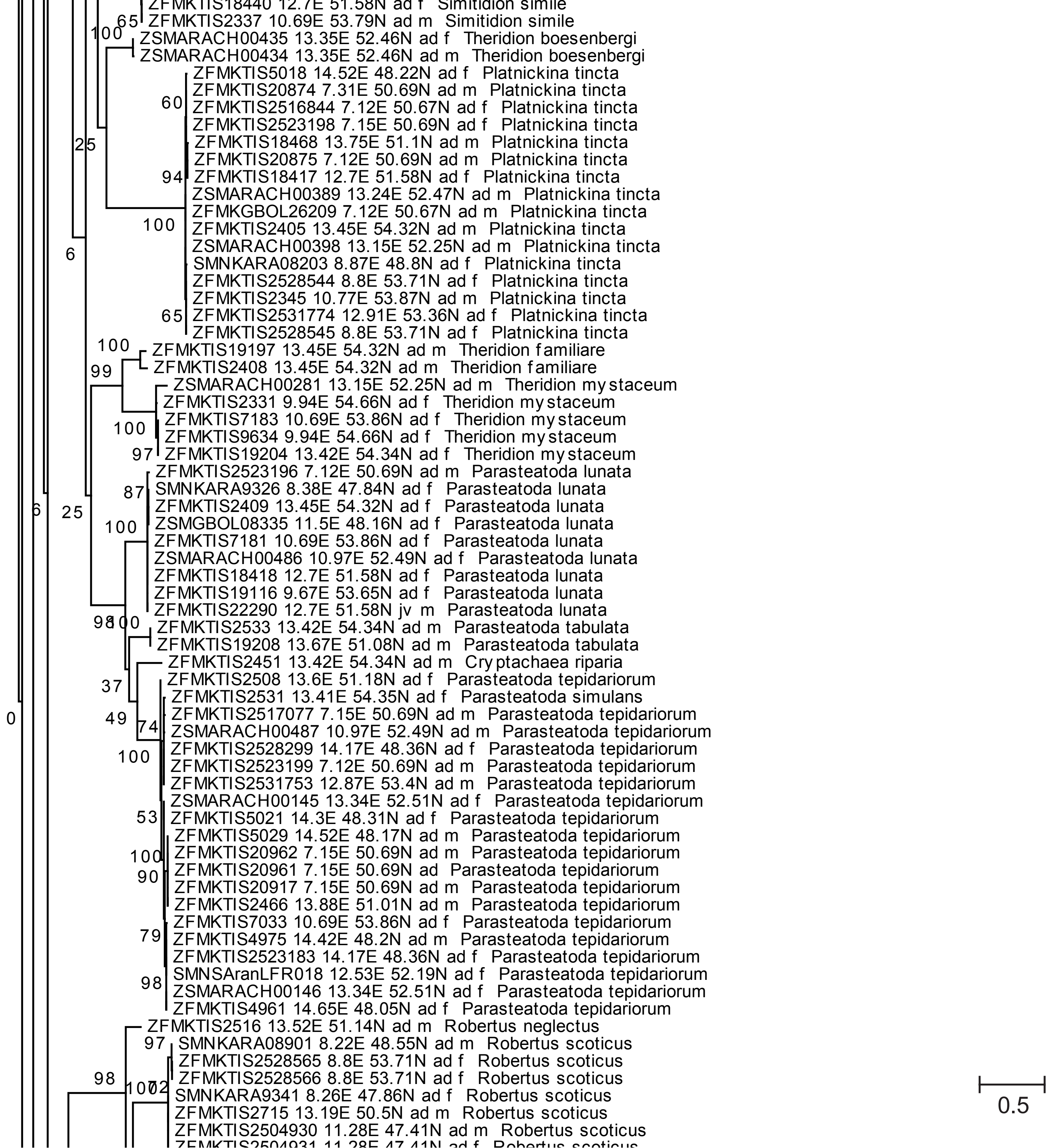
ZFMKTIS18440 12.7E 51.58N ad f Simitidion simile
ZFMKTIS2337 10.69E 53.79N ad m Simitidion simile
ZSMARACH00435 13.35E 52.46N ad f Theridion boesenbergi
ZSMARACH00434 13.35E 52.46N ad m Theridion boesenbergi
ZFMKTIS5018 14.52E 48.22N ad f Platnickina tincta
ZFMKTIS20874 7.31E 50.69N ad m Platnickina tincta
ZFMKTIS2516844 7.12E 50.67N ad f Platnickina tincta
ZFMKTIS2523198 7.15E 50.69N ad f Platnickina tincta
ZFMKTIS18468 13.75E 51.1N ad m Platnickina tincta
ZFMKTIS20875 7.12E 50.69N ad m Platnickina tincta
ZFMKTIS18417 12.7E 51.58N ad f Platnickina tincta
ZSMARACH00389 13.24E 52.47N ad m Platnickina tincta
ZFMKGBOL26209 7.12E 50.67N ad m Platnickina tincta
ZFMKTIS2405 13.45E 54.32N ad m Platnickina tincta
ZSMARACH00398 13.15E 52.25N ad m Platnickina tincta
SMNKARA08203 8.87E 48.8N ad f Platnickina tincta
ZFMKTIS2528544 8.8E 53.71N ad f Platnickina tincta
ZFMKTIS2345 10.77E 53.87N ad m Platnickina tincta
ZFMKTIS2531774 12.91E 53.36N ad f Platnickina tincta
ZFMKTIS2528545 8.8E 53.71N ad f Platnickina tincta
ZFMKTIS19197 13.45E 54.32N ad m Theridion familiare
ZFMKTIS2408 13.45E 54.32N ad m Theridion familiare
ZSMARACH00281 13.15E 52.25N ad m Theridion mystaceum
ZFMKTIS2331 9.94E 54.66N ad f Theridion mystaceum
ZFMKTIS7183 10.69E 53.86N ad f Theridion mystaceum
ZFMKTIS9634 9.94E 54.66N ad f Theridion mystaceum
ZFMKTIS19204 13.42E 54.34N ad f Theridion mystaceum
ZFMKTIS2523196 7.12E 50.69N ad m Parasteatoda lunata
SMNKARA9326 8.38E 47.84N ad f Parasteatoda lunata
ZFMKTIS2409 13.45E 54.32N ad f Parasteatoda lunata
ZSMGBOL08335 11.5E 48.16N ad f Parasteatoda lunata
ZFMKTIS7181 10.69E 53.86N ad f Parasteatoda lunata
ZSMARACH00486 10.97E 52.49N ad f Parasteatoda lunata
ZFMKTIS18418 12.7E 51.58N ad f Parasteatoda lunata
ZFMKTIS19116 9.67E 53.65N ad f Parasteatoda lunata
ZFMKTIS22290 12.7E 51.58N jv m Parasteatoda lunata
ZFMKTIS2533 13.42E 54.34N ad m Parasteatoda tabulata
ZFMKTIS19208 13.67E 51.08N ad m Parasteatoda tabulata
ZFMKTIS2451 13.42E 54.34N ad m Cryptachaea riparia
ZFMKTIS2508 13.6E 51.18N ad f Parasteatoda tepidariorum
ZFMKTIS2531 13.41E 54.35N ad f Parasteatoda simulans
ZFMKTIS2517077 7.15E 50.69N ad m Parasteatoda tepidariorum
ZSMARACH00487 10.97E 52.49N ad m Parasteatoda tepidariorum
ZFMKTIS2528299 14.17E 48.36N ad f Parasteatoda tepidariorum
ZFMKTIS2523199 7.12E 50.69N ad m Parasteatoda tepidariorum
ZFMKTIS2531753 12.87E 53.4N ad m Parasteatoda tepidariorum
ZSMARACH00145 13.34E 52.51N ad f Parasteatoda tepidariorum
ZFMKTIS5021 14.3E 48.31N ad f Parasteatoda tepidariorum
ZFMKTIS5029 14.52E 48.17N ad m Parasteatoda tepidariorum
ZFMKTIS20962 7.15E 50.69N ad m Parasteatoda tepidariorum
ZFMKTIS20961 7.15E 50.69N ad Parasteatoda tepidariorum
ZFMKTIS20917 7.15E 50.69N ad m Parasteatoda tepidariorum
ZFMKTIS2466 13.88E 51.01N ad m Parasteatoda tepidariorum
ZFMKTIS7033 10.69E 53.86N ad f Parasteatoda tepidariorum
ZFMKTIS4975 14.42E 48.2N ad m Parasteatoda tepidariorum
ZFMKTIS2523183 14.17E 48.36N ad f Parasteatoda tepidariorum
SMNSAranLFR018 12.53E 52.19N ad f Parasteatoda tepidariorum
ZSMARACH00146 13.34E 52.51N ad f Parasteatoda tepidariorum
ZFMKTIS4961 14.65E 48.05N ad f Parasteatoda tepidariorum
ZFMKTIS2516 13.52E 51.14N ad m Robertus neglectus
SMNKARA08901 8.22E 48.55N ad m Robertus scoticus
ZFMKTIS2528565 8.8E 53.71N ad f Robertus scoticus
ZFMKTIS2528566 8.8E 53.71N ad f Robertus scoticus
SMNKARA9341 8.26E 47.86N ad f Robertus scoticus
ZFMKTIS2715 13.19E 50.5N ad m Robertus scoticus
ZFMKTIS2504930 11.28E 47.41N ad m Robertus scoticus
ZFMKTIS2504931 11.28E 47.41N ad f Robertus scoticus
0.5

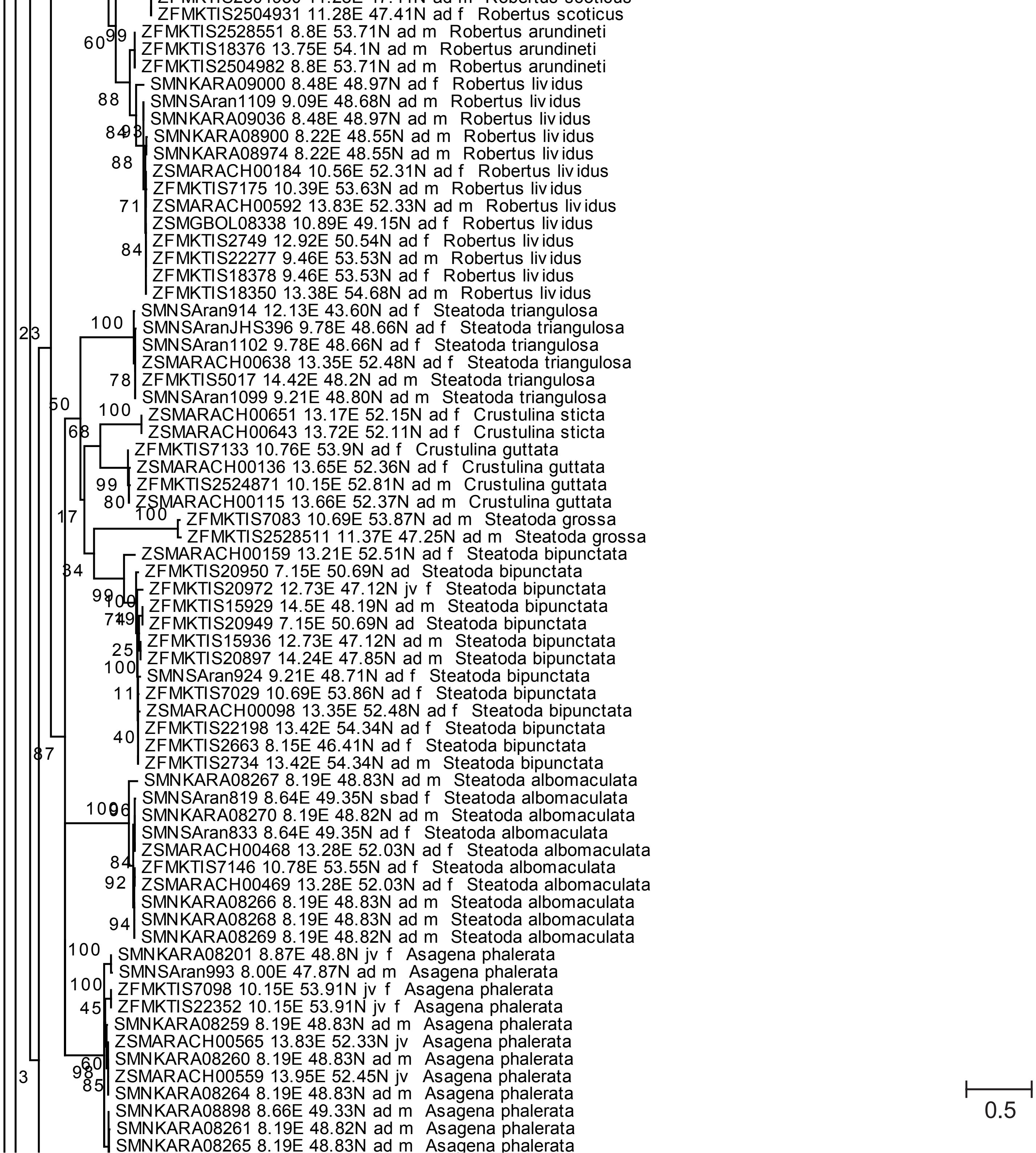

ZFMKTIS2504931 11.28E 47.41N ad f Robertus scoticus
ZFMKTIS2528551 8.8E 53.71N ad m Robertus arundineti
ZFMKTIS18376 13.75E 54.1N ad m Robertus arundineti
ZFMKTIS2504982 8.8E 53.71N ad m Robertus arundineti
SMNKARA09000 8.48E 48.97N ad f Robertus lividus
SMNSAran1109 9.09E 48.68N ad m Robertus lividus
SMNKARA09036 8.48E 48.97N ad m Robertus lividus
SMNKARA08900 8.22E 48.55N ad m Robertus lividus
SMNKARA08974 8.22E 48.55N ad m Robertus lividus
ZSMARACH00184 10.56E 52.31N ad f Robertus lividus
ZFMKTIS7175 10.39E 53.63N ad m Robertus lividus
ZSMARACH00592 13.83E 52.33N ad m Robertus lividus
ZSMGBOL08338 10.89E 49.15N ad f Robertus lividus
ZFMKTIS2749 12.92E 50.54N ad f Robertus lividus
ZFMKTIS22277 9.46E 53.53N ad m Robertus lividus
ZFMKTIS18378 9.46E 53.53N ad f Robertus lividus
ZFMKTIS18350 13.38E 54.68N ad m Robertus lividus
SMNSAran914 12.13E 43.60N ad f Steatoda triangulosa
SMNSAranJHS396 9.78E 48.66N ad f Steatoda triangulosa
SMNSAran1102 9.78E 48.66N ad f Steatoda triangulosa
ZSMARACH00638 13.35E 52.48N ad f Steatoda triangulosa
ZFMKTIS5017 14.42E 48.2N ad m Steatoda triangulosa
SMNSAran1099 9.21E 48.80N ad m Steatoda triangulosa
ZSMARACH00651 13.17E 52.15N ad f Crustulina sticta
ZSMARACH00643 13.72E 52.11N ad f Crustulina sticta
ZFMKTIS7133 10.76E 53.9N ad f Crustulina guttata
ZSMARACH00136 13.65E 52.36N ad f Crustulina guttata
ZFMKTIS2524871 10.15E 52.81N ad m Crustulina guttata
ZSMARACH00115 13.66E 52.37N ad m Crustulina guttata
ZFMKTIS7083 10.69E 53.87N ad m Steatoda grossa
ZFMKTIS2528511 11.37E 47.25N ad m Steatoda grossa
ZSMARACH00159 13.21E 52.51N ad f Steatoda bipunctata
ZFMKTIS20950 7.15E 50.69N ad Steatoda bipunctata
ZFMKTIS20972 12.73E 47.12N jv f Steatoda bipunctata
ZFMKTIS15929 14.5E 48.19N ad m Steatoda bipunctata
ZFMKTIS20949 7.15E 50.69N ad Steatoda bipunctata
ZFMKTIS15936 12.73E 47.12N ad m Steatoda bipunctata
ZFMKTIS20897 14.24E 47.85N ad m Steatoda bipunctata
SMNSAran924 9.21E 48.71N ad f Steatoda bipunctata
ZFMKTIS7029 10.69E 53.86N ad f Steatoda bipunctata
ZSMARACH00098 13.35E 52.48N ad f Steatoda bipunctata
ZFMKTIS22198 13.42E 54.34N ad f Steatoda bipunctata
ZFMKTIS2663 8.15E 46.41N ad f Steatoda bipunctata
ZFMKTIS2734 13.42E 54.34N ad m Steatoda bipunctata
SMNKARA08267 8.19E 48.83N ad m Steatoda albomaculata
SMNSAran819 8.64E 49.35N sbad f Steatoda albomaculata
SMNKARA08270 8.19E 48.82N ad m Steatoda albomaculata
SMNSAran833 8.64E 49.35N ad f Steatoda albomaculata
ZSMARACH00468 13.28E 52.03N ad f Steatoda albomaculata
ZFMKTIS7146 10.78E 53.55N ad f Steatoda albomaculata
ZSMARACH00469 13.28E 52.03N ad f Steatoda albomaculata
SMNKARA08266 8.19E 48.83N ad m Steatoda albomaculata
SMNKARA08268 8.19E 48.83N ad m Steatoda albomaculata
SMNKARA08269 8.19E 48.82N ad m Steatoda albomaculata
SMNKARA08201 8.87E 48.8N jv f Asagena phalerata
SMNSAran993 8.00E 47.87N ad m Asagena phalerata
ZFMKTIS7098 10.15E 53.91N jv f Asagena phalerata
ZFMKTIS22352 10.15E 53.91N jv f Asagena phalerata
SMNKARA08259 8.19E 48.83N ad m Asagena phalerata
ZSMARACH00565 13.83E 52.33N jv Asagena phalerata
SMNKARA08260 8.19E 48.83N ad m Asagena phalerata
ZSMARACH00559 13.95E 52.45N jv Asagena phalerata
SMNKARA08264 8.19E 48.83N ad m Asagena phalerata
SMNKARA08898 8.66E 49.33N ad m Asagena phalerata
SMNKARA08261 8.19E 48.82N ad m Asagena phalerata
SMNKARA08265 8.19E 48.83N ad m Asagena phalerata
0.5

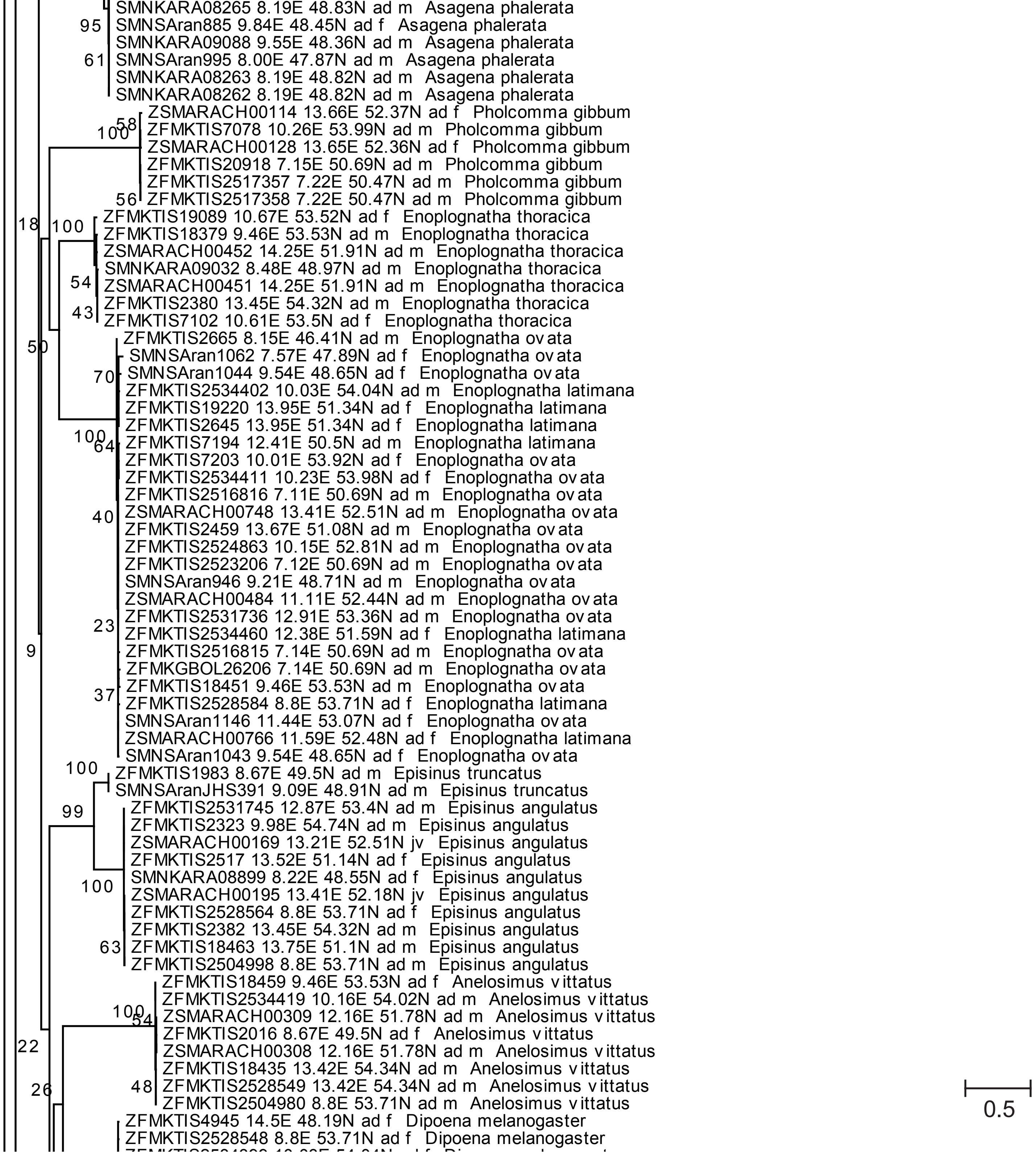
SMNKARA08265 8.19E 48.83N ad m Asagena phalerata
SMNSAran885 9.84E 48.45N ad f Asagena phalerata
SMNKARA09088 9.55E 48.36N ad m Asagena phalerata
SMNSAran995 8.00E 47.87N ad m Asagena phalerata
SMNKARA08263 8.19E 48.82N ad m Asagena phalerata
SMNKARA08262 8.19E 48.82N ad m Asagena phalerata
ZSMARACH00114 13.66E 52.37N ad f Pholcomma gibbum
ZFMKTIS7078 10.26E 53.99N ad m Pholcomma gibbum
ZSMARACH00128 13.65E 52.36N ad f Pholcomma gibbum
ZFMKTIS20918 7.15E 50.69N ad m Pholcomma gibbum
ZFMKTIS2517357 7.22E 50.47N ad m Pholcomma gibbum
ZFMKTIS2517358 7.22E 50.47N ad m Pholcomma gibbum
ZFMKTIS19089 10.67E 53.52N ad f Enoplognatha thoracica
ZFMKTIS18379 9.46E 53.53N ad m Enoplognatha thoracica
ZSMARACH00452 14.25E 51.91N ad m Enoplognatha thoracica
SMNKARA09032 8.48E 48.97N ad m Enoplognatha thoracica
ZSMARACH00451 14.25E 51.91N ad m Enoplognatha thoracica
ZFMKTIS2380 13.45E 54.32N ad m Enoplognatha thoracica
ZFMKTIS7102 10.61E 53.5N ad f Enoplognatha thoracica
ZFMKTIS2665 8.15E 46.41N ad m Enoplognatha ovata
SMNSAran1062 7.57E 47.89N ad f Enoplognatha ovata
SMNSAran1044 9.54E 48.65N ad f Enoplognatha ovata
ZFMKTIS2534402 10.03E 54.04N ad m Enoplognatha latimana
ZFMKTIS19220 13.95E 51.34N ad f Enoplognatha latimana
ZFMKTIS2645 13.95E 51.34N ad f Enoplognatha latimana
ZFMKTIS7194 12.41E 50.5N ad m Enoplognatha latimana
ZFMKTIS7203 10.01E 53.92N ad f Enoplognatha ovata
ZFMKTIS2534411 10.23E 53.98N ad f Enoplognatha ovata
ZFMKTIS2516816 7.11E 50.69N ad m Enoplognatha ovata
ZSMARACH00748 13.41E 52.51N ad m Enoplognatha ovata
ZFMKTIS2459 13.67E 51.08N ad m Enoplognatha ovata
ZFMKTIS2524863 10.15E 52.81N ad m Enoplognatha ovata
ZFMKTIS2523206 7.12E 50.69N ad m Enoplognatha ovata
SMNSAran946 9.21E 48.71N ad m Enoplognatha ovata
ZSMARACH00484 11.11E 52.44N ad m Enoplognatha ovata
ZFMKTIS2531736 12.91E 53.36N ad m Enoplognatha ovata
ZFMKTIS2534460 12.38E 51.59N ad f Enoplognatha latimana
ZFMKTIS2516815 7.14E 50.69N ad m Enoplognatha ovata
ZFMKGBOL26206 7.14E 50.69N ad m Enoplognatha ovata
ZFMKTIS18451 9.46E 53.53N ad m Enoplognatha ovata
ZFMKTIS2528584 8.8E 53.71N ad f Enoplognatha latimana
SMNSAran1146 11.44E 53.07N ad f Enoplognatha ovata
ZSMARACH00766 11.59E 52.48N ad f Enoplognatha latimana
SMNSAran1043 9.54E 48.65N ad f Enoplognatha ovata
ZFMKTIS1983 8.67E 49.5N ad m Episinus truncatus
SMNSAranJHS391 9.09E 48.91N ad m Episinus truncatus
ZFMKTIS2531745 12.87E 53.4N ad m Episinus angulatus
ZFMKTIS2323 9.98E 54.74N ad m Episinus angulatus
ZSMARACH00169 13.21E 52.51N jv Episinus angulatus
ZFMKTIS2517 13.52E 51.14N ad f Episinus angulatus
SMNKARA08899 8.22E 48.55N ad f Episinus angulatus
ZSMARACH00195 13.41E 52.18N jv Episinus angulatus
ZFMKTIS2528564 8.8E 53.71N ad f Episinus angulatus
ZFMKTIS2382 13.45E 54.32N ad m Episinus angulatus
ZFMKTIS18463 13.75E 51.1N ad m Episinus angulatus
ZFMKTIS2504998 8.8E 53.71N ad m Episinus angulatus
ZFMKTIS18459 9.46E 53.53N ad f Anelosimus vittatus
ZFMKTIS2534419 10.16E 54.02N ad m Anelosimus vittatus
ZSMARACH00309 12.16E 51.78N ad m Anelosimus vittatus
ZFMKTIS2016 8.67E 49.5N ad f Anelosimus vittatus
ZSMARACH00308 12.16E 51.78N ad m Anelosimus vittatus
ZFMKTIS18435 13.42E 54.34N ad m Anelosimus vittatus
ZFMKTIS2528549 13.42E 54.34N ad m Anelosimus vittatus
ZFMKTIS2504980 8.8E 53.71N ad m Anelosimus vittatus
ZFMKTIS4945 14.5E 48.19N ad f Dipoena melanogaster
ZFMKTIS2528548 8.8E 53.71N ad f Dipoena melanogaster
0.5

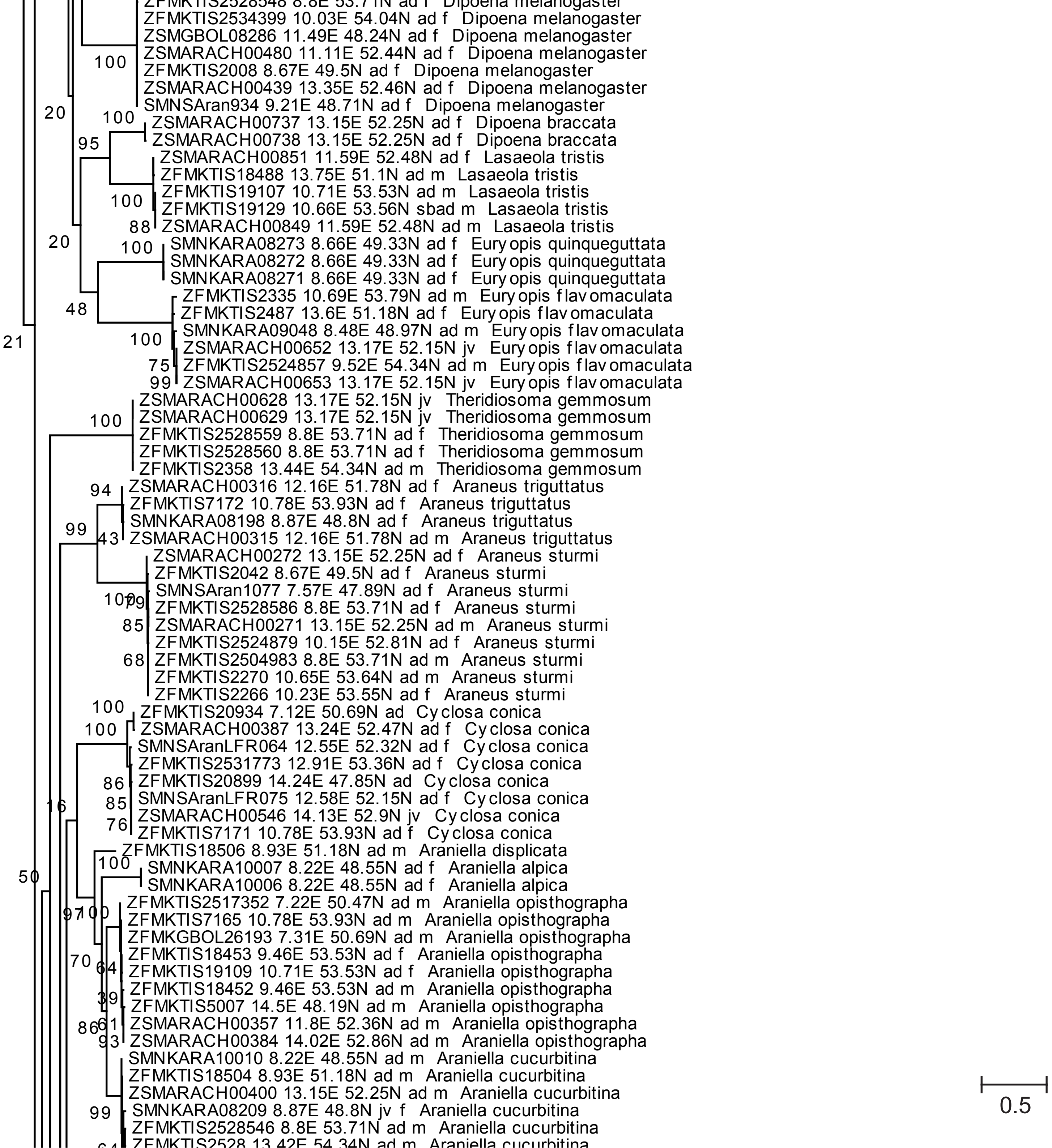
ZFMKTIS2528548 8.8E 53.71N ad f Dipoena melanogaster
ZFMKTIS2534399 10.03E 54.04N ad f Dipoena melanogaster
ZSMGBOL08286 11.49E 48.24N ad f Dipoena melanogaster
ZSMARACH00480 11.11E 52.44N ad f Dipoena melanogaster
ZFMKTIS2008 8.67E 49.5N ad f Dipoena melanogaster
ZSMARACH00439 13.35E 52.46N ad f Dipoena melanogaster
SMNSAran934 9.21E 48.71N ad f Dipoena melanogaster
ZSMARACH00737 13.15E 52.25N ad f Dipoena braccata
ZSMARACH00738 13.15E 52.25N ad f Dipoena braccata
ZSMARACH00851 11.59E 52.48N ad f Lasaeola tristis
ZFMKTIS18488 13.75E 51.1N ad m Lasaeola tristis
ZFMKTIS19107 10.71E 53.53N ad m Lasaeola tristis
ZFMKTIS19129 10.66E 53.56N sbad m Lasaeola tristis
ZSMARACH00849 11.59E 52.48N ad m Lasaeola tristis
SMNKARA08273 8.66E 49.33N ad f Euryopis quinqueguttata
SMNKARA08272 8.66E 49.33N ad f Euryopis quinqueguttata
SMNKARA08271 8.66E 49.33N ad f Euryopis quinqueguttata
ZFMKTIS2335 10.69E 53.79N ad m Euryopis flavomaculata
ZFMKTIS2487 13.6E 51.18N ad f Euryopis flavomaculata
SMNKARA09048 8.48E 48.97N ad m Euryopis flavomaculata
ZSMARACH00652 13.17E 52.15N jv Euryopis flavomaculata
ZFMKTIS2524857 9.52E 54.34N ad m Euryopis flavomaculata
ZSMARACH00653 13.17E 52.15N jv Euryopis flavomaculata
ZSMARACH00628 13.17E 52.15N jv Theridiosoma gemmosum
ZSMARACH00629 13.17E 52.15N jv Theridiosoma gemmosum
ZFMKTIS2528559 8.8E 53.71N ad f Theridiosoma gemmosum
ZFMKTIS2528560 8.8E 53.71N ad f Theridiosoma gemmosum
ZFMKTIS2358 13.44E 54.34N ad m Theridiosoma gemmosum
ZSMARACH00316 12.16E 51.78N ad f Araneus triguttatus
ZFMKTIS7172 10.78E 53.93N ad f Araneus triguttatus
SMNKARA08198 8.87E 48.8N ad f Araneus triguttatus
ZSMARACH00315 12.16E 51.78N ad m Araneus triguttatus
ZSMARACH00272 13.15E 52.25N ad f Araneus sturmi
ZFMKTIS2042 8.67E 49.5N ad f Araneus sturmi
SMNSAran1077 7.57E 47.89N ad f Araneus sturmi
ZFMKTIS2528586 8.8E 53.71N ad f Araneus sturmi
ZSMARACH00271 13.15E 52.25N ad m Araneus sturmi
ZFMKTIS2524879 10.15E 52.81N ad f Araneus sturmi
ZFMKTIS2504983 8.8E 53.71N ad m Araneus sturmi
ZFMKTIS2270 10.65E 53.64N ad m Araneus sturmi
ZFMKTIS2266 10.23E 53.55N ad f Araneus sturmi
ZFMKTIS20934 7.12E 50.69N ad Cyclosa conica
ZSMARACH00387 13.24E 52.47N ad f Cyclosa conica
SMNSAranLFR064 12.55E 52.32N ad f Cyclosa conica
ZFMKTIS2531773 12.91E 53.36N ad f Cyclosa conica
ZFMKTIS20899 14.24E 47.85N ad Cyclosa conica
SMNSAranLFR075 12.58E 52.15N ad f Cyclosa conica
ZSMARACH00546 14.13E 52.9N jv Cyclosa conica
ZFMKTIS7171 10.78E 53.93N ad f Cyclosa conica
ZFMKTIS18506 8.93E 51.18N ad m Araniella displicata
SMNKARA10007 8.22E 48.55N ad f Araniella alpica
SMNKARA10006 8.22E 48.55N ad f Araniella alpica
ZFMKTIS2517352 7.22E 50.47N ad m Araniella opisthographa
ZFMKTIS7165 10.78E 53.93N ad m Araniella opisthographa
ZFMKGBOL26193 7.31E 50.69N ad m Araniella opisthographa
ZFMKTIS18453 9.46E 53.53N ad f Araniella opisthographa
ZFMKTIS19109 10.71E 53.53N ad f Araniella opisthographa
ZFMKTIS18452 9.46E 53.53N ad m Araniella opisthographa
ZFMKTIS5007 14.5E 48.19N ad m Araniella opisthographa
ZSMARACH00357 11.8E 52.36N ad m Araniella opisthographa
ZSMARACH00384 14.02E 52.86N ad m Araniella opisthographa
SMNKARA10010 8.22E 48.55N ad m Araniella cucurbitina
ZFMKTIS18504 8.93E 51.18N ad m Araniella cucurbitina
ZSMARACH00400 13.15E 52.25N ad m Araniella cucurbitina
SMNKARA08209 8.87E 48.8N jv f Araniella cucurbitina
ZFMKTIS2528546 8.8E 53.71N ad m Araniella cucurbitina
ZFMKTIS2528 13.42E 54.34N ad m Araniella cucurbitina
0.5

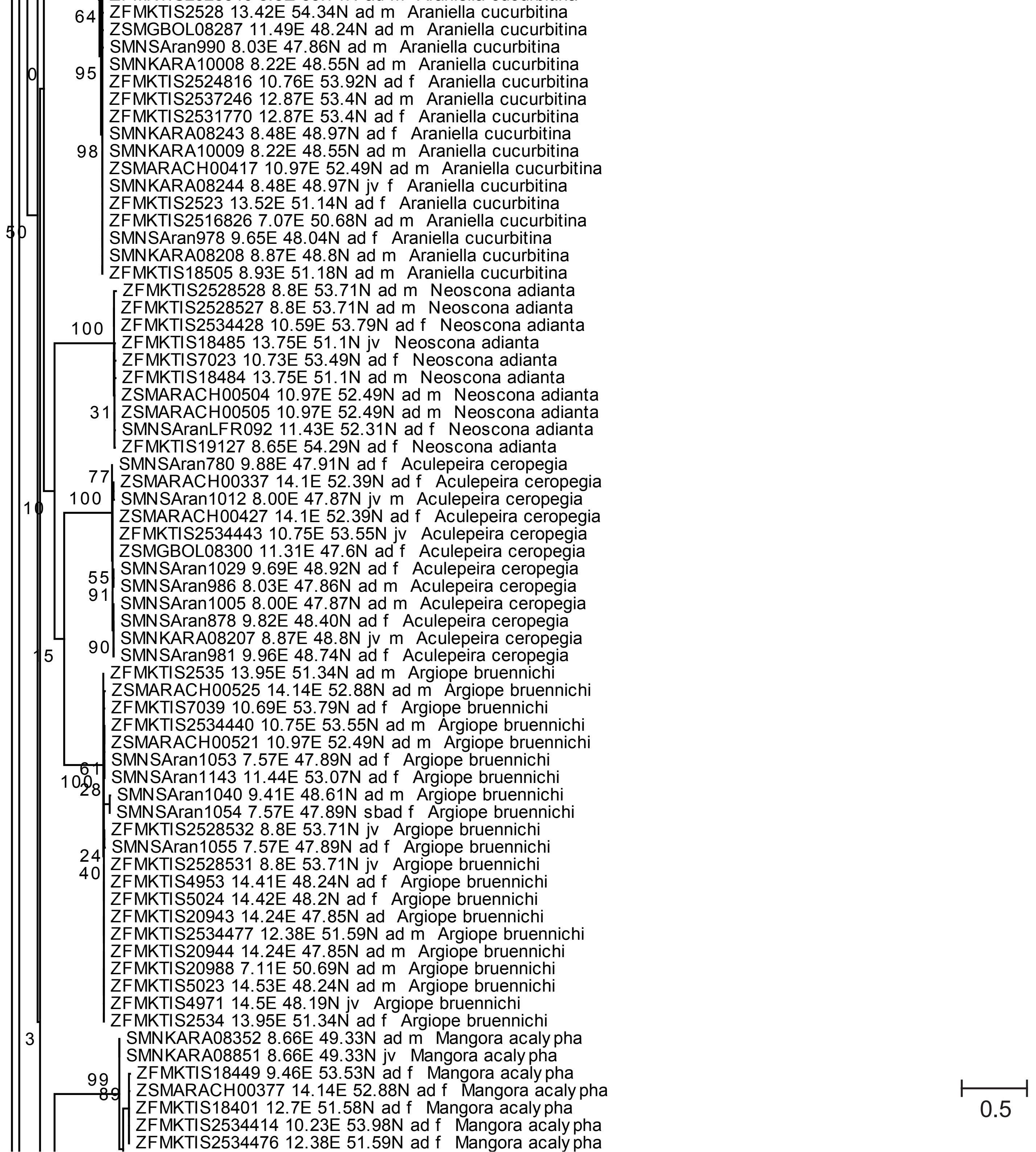

ZFMKTIS2528 13.42E 54.34N ad m Araniella cucurbitina
ZSMGBOL08287 11.49E 48.24N ad m Araniella cucurbitina
SMNSAran990 8.03E 47.86N ad m Araniella cucurbitina
SMNKARA10008 8.22E 48.55N ad m Araniella cucurbitina
ZFMKTIS2524816 10.76E 53.92N ad f Araniella cucurbitina
ZFMKTIS2537246 12.87E 53.4N ad m Araniella cucurbitina
ZFMKTIS2531770 12.87E 53.4N ad f Araniella cucurbitina
SMNKARA08243 8.48E 48.97N ad f Araniella cucurbitina
SMNKARA10009 8.22E 48.55N ad m Araniella cucurbitina
ZSMARACH00417 10.97E 52.49N ad m Araniella cucurbitina
SMNKARA08244 8.48E 48.97N jv f Araniella cucurbitina
ZFMKTIS2523 13.52E 51.14N ad f Araniella cucurbitina
ZFMKTIS2516826 7.07E 50.68N ad m Araniella cucurbitina
SMNSAran978 9.65E 48.04N ad f Araniella cucurbitina
SMNKARA08208 8.87E 48.8N ad m Araniella cucurbitina
ZFMKTIS18505 8.93E 51.18N ad m Araniella cucurbitina
ZFMKTIS2528528 8.8E 53.71N ad m Neoscona adianta
ZFMKTIS2528527 8.8E 53.71N ad m Neoscona adianta
ZFMKTIS2534428 10.59E 53.79N ad f Neoscona adianta
ZFMKTIS18485 13.75E 51.1N jv Neoscona adianta
ZFMKTIS7023 10.73E 53.49N ad f Neoscona adianta
ZFMKTIS18484 13.75E 51.1N ad m Neoscona adianta
ZSMARACH00504 10.97E 52.49N ad m Neoscona adianta
ZSMARACH00505 10.97E 52.49N ad m Neoscona adianta
SMNSAranLFR092 11.43E 52.31N ad f Neoscona adianta
ZFMKTIS19127 8.65E 54.29N ad f Neoscona adianta
SMNSAran780 9.88E 47.91N ad f Aculepeira ceropegia
ZSMARACH00337 14.1E 52.39N ad f Aculepeira ceropegia
SMNSAran1012 8.00E 47.87N jv m Aculepeira ceropegia
ZSMARACH00427 14.1E 52.39N ad f Aculepeira ceropegia
ZFMKTIS2534443 10.75E 53.55N jv Aculepeira ceropegia
ZSMGBOL08300 11.31E 47.6N ad f Aculepeira ceropegia
SMNSAran1029 9.69E 48.92N ad f Aculepeira ceropegia
SMNSAran986 8.03E 47.86N ad m Aculepeira ceropegia
SMNSAran1005 8.00E 47.87N ad m Aculepeira ceropegia
SMNSAran878 9.82E 48.40N ad f Aculepeira ceropegia
SMNKARA08207 8.87E 48.8N jv m Aculepeira ceropegia
SMNSAran981 9.96E 48.74N ad f Aculepeira ceropegia
ZFMKTIS2535 13.95E 51.34N ad m Argiope bruennichi
ZSMARACH00525 14.14E 52.88N ad m Argiope bruennichi
ZFMKTIS7039 10.69E 53.79N ad f Argiope bruennichi
ZFMKTIS2534440 10.75E 53.55N ad m Argiope bruennichi
ZSMARACH00521 10.97E 52.49N ad m Argiope bruennichi
SMNSAran1053 7.57E 47.89N ad f Argiope bruennichi
SMNSAran1143 11.44E 53.07N ad f Argiope bruennichi
SMNSAran1040 9.41E 48.61N ad m Argiope bruennichi
SMNSAran1054 7.57E 47.89N sbad f Argiope bruennichi
ZFMKTIS2528532 8.8E 53.71N jv Argiope bruennichi
SMNSAran1055 7.57E 47.89N ad f Argiope bruennichi
ZFMKTIS2528531 8.8E 53.71N jv Argiope bruennichi
ZFMKTIS4953 14.41E 48.24N ad f Argiope bruennichi
ZFMKTIS5024 14.42E 48.2N ad f Argiope bruennichi
ZFMKTIS20943 14.24E 47.85N ad Argiope bruennichi
ZFMKTIS2534477 12.38E 51.59N ad m Argiope bruennichi
ZFMKTIS20944 14.24E 47.85N ad m Argiope bruennichi
ZFMKTIS20988 7.11E 50.69N ad m Argiope bruennichi
ZFMKTIS5023 14.53E 48.24N ad m Argiope bruennichi
ZFMKTIS4971 14.5E 48.19N jv Argiope bruennichi
ZFMKTIS2534 13.95E 51.34N ad f Argiope bruennichi
SMNKARA08352 8.66E 49.33N ad m Mangora acalypha
SMNKARA08851 8.66E 49.33N jv Mangora acalypha
ZFMKTIS18449 9.46E 53.53N ad f Mangora acalypha
ZSMARACH00377 14.14E 52.88N ad f Mangora acalypha
ZFMKTIS18401 12.7E 51.58N ad f Mangora acalypha
ZFMKTIS2534414 10.23E 53.98N ad f Mangora acalypha
ZFMKTIS2534476 12.38E 51.59N ad f Mangora acalypha
64
0
95
98
50
100
31
77
100
10
55
91
90
15
61
100
28
24
40
3
99
89
0.5

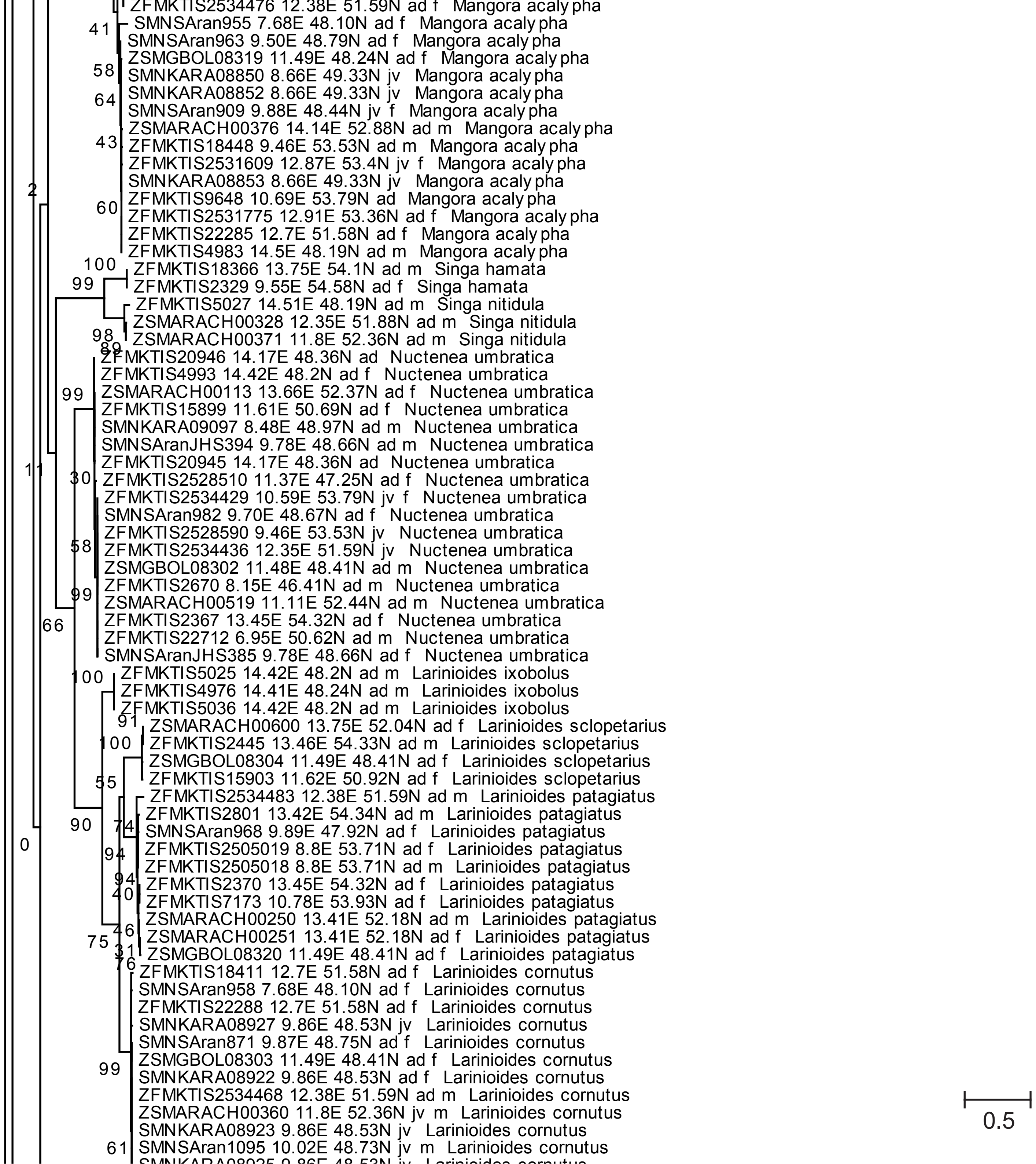
ZFMKTIS2534476 12.38E 51.59N ad f Mangora acalypha
SMNSAran955 7.68E 48.10N ad f Mangora acalypha
SMNSAran963 9.50E 48.79N ad f Mangora acalypha
ZSMGBOL08319 11.49E 48.24N ad f Mangora acalypha
SMNKARA08850 8.66E 49.33N jv Mangora acalypha
SMNKARA08852 8.66E 49.33N jv Mangora acalypha
SMNSAran909 9.88E 48.44N jv f Mangora acalypha
ZSMARACH00376 14.14E 52.88N ad m Mangora acalypha
ZFMKTIS18448 9.46E 53.53N ad m Mangora acalypha
ZFMKTIS2531609 12.87E 53.4N jv f Mangora acalypha
SMNKARA08853 8.66E 49.33N jv Mangora acalypha
ZFMKTIS9648 10.69E 53.79N ad Mangora acalypha
ZFMKTIS2531775 12.91E 53.36N ad f Mangora acalypha
ZFMKTIS22285 12.7E 51.58N ad f Mangora acalypha
ZFMKTIS4983 14.5E 48.19N ad m Mangora acalypha
ZFMKTIS18366 13.75E 54.1N ad m Singa hamata
ZFMKTIS2329 9.55E 54.58N ad f Singa hamata
ZFMKTIS5027 14.51E 48.19N ad m Singa nitidula
ZSMARACH00328 12.35E 51.88N ad m Singa nitidula
ZSMARACH00371 11.8E 52.36N ad m Singa nitidula
ZFMKTIS20946 14.17E 48.36N ad Nuctenea umbratica
ZFMKTIS4993 14.42E 48.2N ad f Nuctenea umbratica
ZSMARACH00113 13.66E 52.37N ad f Nuctenea umbratica
ZFMKTIS15899 11.61E 50.69N ad f Nuctenea umbratica
SMNKARA09097 8.48E 48.97N ad m Nuctenea umbratica
SMNSAranJHS394 9.78E 48.66N ad m Nuctenea umbratica
ZFMKTIS20945 14.17E 48.36N ad Nuctenea umbratica
ZFMKTIS2528510 11.37E 47.25N ad f Nuctenea umbratica
ZFMKTIS2534429 10.59E 53.79N jv f Nuctenea umbratica
SMNSAran982 9.70E 48.67N ad f Nuctenea umbratica
ZFMKTIS2528590 9.46E 53.53N jv Nuctenea umbratica
ZFMKTIS2534436 12.35E 51.59N jv Nuctenea umbratica
ZSMGBOL08302 11.48E 48.41N ad m Nuctenea umbratica
ZFMKTIS2670 8.15E 46.41N ad m Nuctenea umbratica
ZSMARACH00519 11.11E 52.44N ad m Nuctenea umbratica
ZFMKTIS2367 13.45E 54.32N ad f Nuctenea umbratica
ZFMKTIS22712 6.95E 50.62N ad m Nuctenea umbratica
SMNSAranJHS385 9.78E 48.66N ad f Nuctenea umbratica
ZFMKTIS5025 14.42E 48.2N ad m Larinioides ixobolus
ZFMKTIS4976 14.41E 48.24N ad m Larinioides ixobolus
ZFMKTIS5036 14.42E 48.2N ad m Larinioides ixobolus
ZSMARACH00600 13.75E 52.04N ad f Larinioides sclopetarius
ZFMKTIS2445 13.46E 54.33N ad m Larinioides sclopetarius
ZSMGBOL08304 11.49E 48.41N ad f Larinioides sclopetarius
ZFMKTIS15903 11.62E 50.92N ad f Larinioides sclopetarius
ZFMKTIS2534483 12.38E 51.59N ad m Larinioides patagiatus
ZFMKTIS2801 13.42E 54.34N ad m Larinioides patagiatus
SMNSAran968 9.89E 47.92N ad f Larinioides patagiatus
ZFMKTIS2505019 8.8E 53.71N ad f Larinioides patagiatus
ZFMKTIS2505018 8.8E 53.71N ad m Larinioides patagiatus
ZFMKTIS2370 13.45E 54.32N ad f Larinioides patagiatus
ZFMKTIS7173 10.78E 53.93N ad f Larinioides patagiatus
ZSMARACH00250 13.41E 52.18N ad m Larinioides patagiatus
ZSMARACH00251 13.41E 52.18N ad f Larinioides patagiatus
ZSMGBOL08320 11.49E 48.41N ad f Larinioides patagiatus
ZFMKTIS18411 12.7E 51.58N ad f Larinioides cornutus
SMNSAran958 7.68E 48.10N ad f Larinioides cornutus
ZFMKTIS22288 12.7E 51.58N ad f Larinioides cornutus
SMNKARA08927 9.86E 48.53N jv Larinioides cornutus
SMNSAran871 9.87E 48.75N ad f Larinioides cornutus
ZSMGBOL08303 11.49E 48.41N ad f Larinioides cornutus
SMNKARA08922 9.86E 48.53N ad f Larinioides cornutus
ZFMKTIS2534468 12.38E 51.59N ad m Larinioides cornutus
ZSMARACH00360 11.8E 52.36N jv m Larinioides cornutus
SMNKARA08923 9.86E 48.53N jv Larinioides cornutus
SMNSAran1095 10.02E 48.73N jv m Larinioides cornutus
0.5

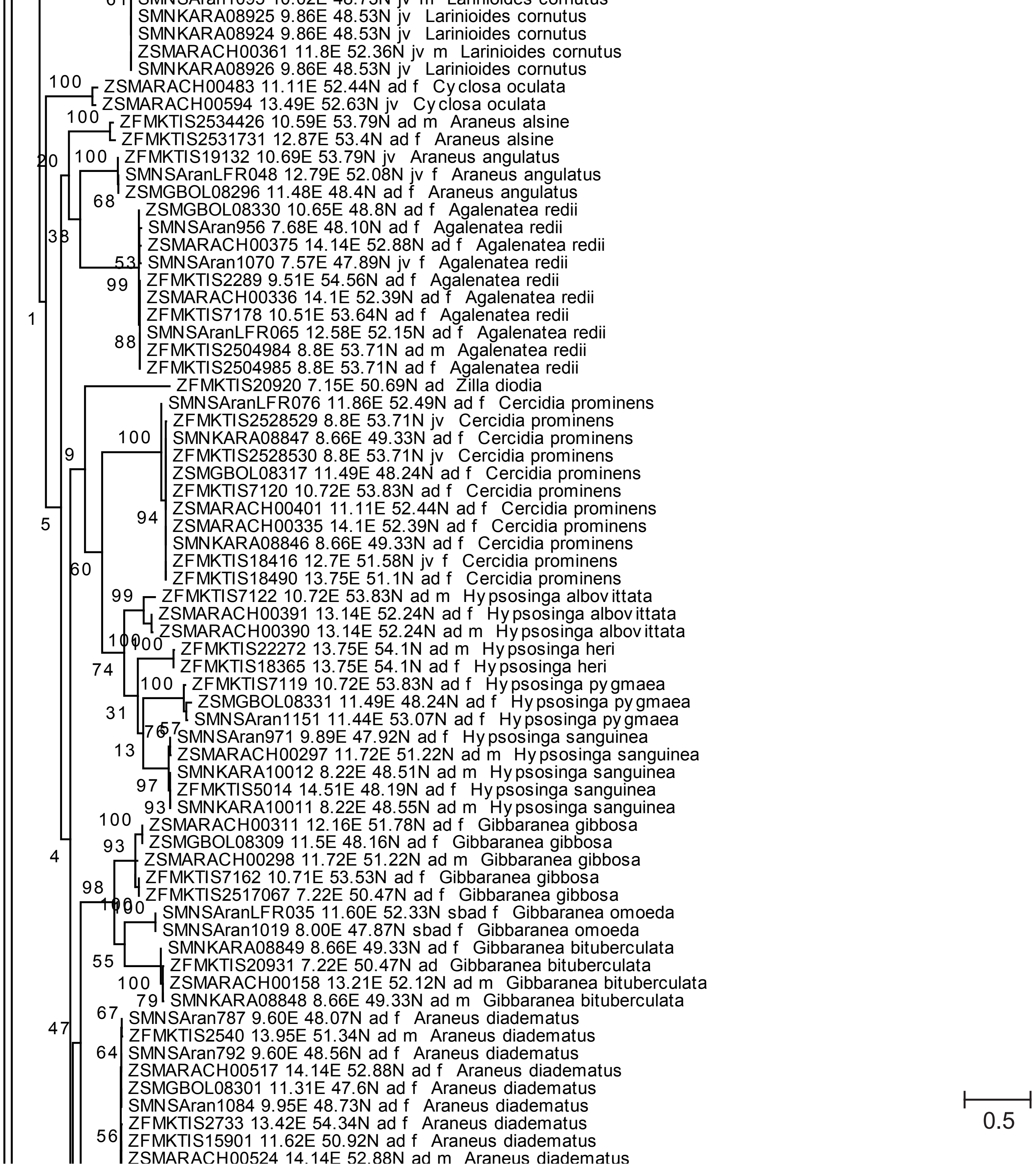
SMNKARA08925 9.86E 48.53N jv Larinioides cornutus
SMNKARA08924 9.86E 48.53N jv Larinioides cornutus
ZSMARACH00361 11.8E 52.36N jv m Larinioides cornutus
SMNKARA08926 9.86E 48.53N jv Larinioides cornutus
ZSMARACH00483 11.11E 52.44N ad f Cyclosa oculata
ZSMARACH00594 13.49E 52.63N jv Cyclosa oculata
ZFMKTIS2534426 10.59E 53.79N ad m Araneus alsine
ZFMKTIS2531731 12.87E 53.4N ad f Araneus alsine
ZFMKTIS19132 10.69E 53.79N jv Araneus angulatus
SMNSAranLFR048 12.79E 52.08N jv f Araneus angulatus
ZSMGBOL08296 11.48E 48.4N ad f Araneus angulatus
ZSMGBOL08330 10.65E 48.8N ad f Agalenatea redii
SMNSAran956 7.68E 48.10N ad f Agalenatea redii
ZSMARACH00375 14.14E 52.88N ad f Agalenatea redii
SMNSAran1070 7.57E 47.89N jv f Agalenatea redii
ZFMKTIS2289 9.51E 54.56N ad f Agalenatea redii
ZSMARACH00336 14.1E 52.39N ad f Agalenatea redii
ZFMKTIS7178 10.51E 53.64N ad f Agalenatea redii
SMNSAranLFR065 12.58E 52.15N ad f Agalenatea redii
ZFMKTIS2504984 8.8E 53.71N ad m Agalenatea redii
ZFMKTIS2504985 8.8E 53.71N ad f Agalenatea redii
ZFMKTIS20920 7.15E 50.69N ad Zilla diodia
SMNSAranLFR076 11.86E 52.49N ad f Cercidia prominens
ZFMKTIS2528529 8.8E 53.71N jv Cercidia prominens
SMNKARA08847 8.66E 49.33N ad f Cercidia prominens
ZFMKTIS2528530 8.8E 53.71N jv Cercidia prominens
ZSMGBOL08317 11.49E 48.24N ad f Cercidia prominens
ZFMKTIS7120 10.72E 53.83N ad f Cercidia prominens
ZSMARACH00401 11.11E 52.44N ad f Cercidia prominens
ZSMARACH00335 14.1E 52.39N ad f Cercidia prominens
SMNKARA08846 8.66E 49.33N ad f Cercidia prominens
ZFMKTIS18416 12.7E 51.58N jv f Cercidia prominens
ZFMKTIS18490 13.75E 51.1N ad f Cercidia prominens
ZFMKTIS7122 10.72E 53.83N ad m Hypsosinga albovittata
ZSMARACH00391 13.14E 52.24N ad f Hypsosinga albovittata
ZSMARACH00390 13.14E 52.24N ad m Hypsosinga albovittata
ZFMKTIS22272 13.75E 54.1N ad m Hypsosinga heri
ZFMKTIS18365 13.75E 54.1N ad f Hypsosinga heri
ZFMKTIS7119 10.72E 53.83N ad f Hypsosinga pygmaea
ZSMGBOL08331 11.49E 48.24N ad f Hypsosinga pygmaea
SMNSAran1151 11.44E 53.07N ad f Hypsosinga pygmaea
SMNSAran971 9.89E 47.92N ad f Hypsosinga sanguinea
ZSMARACH00297 11.72E 51.22N ad m Hypsosinga sanguinea
SMNKARA10012 8.22E 48.51N ad m Hypsosinga sanguinea
ZFMKTIS5014 14.51E 48.19N ad f Hypsosinga sanguinea
SMNKARA10011 8.22E 48.55N ad m Hypsosinga sanguinea
ZSMARACH00311 12.16E 51.78N ad f Gibbaranea gibbosa
ZSMGBOL08309 11.5E 48.16N ad f Gibbaranea gibbosa
ZSMARACH00298 11.72E 51.22N ad m Gibbaranea gibbosa
ZFMKTIS7162 10.71E 53.53N ad f Gibbaranea gibbosa
ZFMKTIS2517067 7.22E 50.47N ad f Gibbaranea gibbosa
SMNSAranLFR035 11.60E 52.33N sbad f Gibbaranea omoeda
SMNSAran1019 8.00E 47.87N sbad f Gibbaranea omoeda
SMNKARA08849 8.66E 49.33N ad f Gibbaranea bituberculata
ZFMKTIS20931 7.22E 50.47N ad Gibbaranea bituberculata
ZSMARACH00158 13.21E 52.12N ad m Gibbaranea bituberculata
SMNKARA08848 8.66E 49.33N ad m Gibbaranea bituberculata
SMNSAran787 9.60E 48.07N ad f Araneus diadematus
ZFMKTIS2540 13.95E 51.34N ad m Araneus diadematus
SMNSAran792 9.60E 48.56N ad f Araneus diadematus
ZSMARACH00517 14.14E 52.88N ad f Araneus diadematus
ZSMGBOL08301 11.31E 47.6N ad f Araneus diadematus
SMNSAran1084 9.95E 48.73N ad f Araneus diadematus
ZFMKTIS2733 13.42E 54.34N ad f Araneus diadematus
ZFMKTIS15901 11.62E 50.92N ad f Araneus diadematus
ZSMARACH00524 14.14E 52.88N ad m Araneus diadematus
0.5

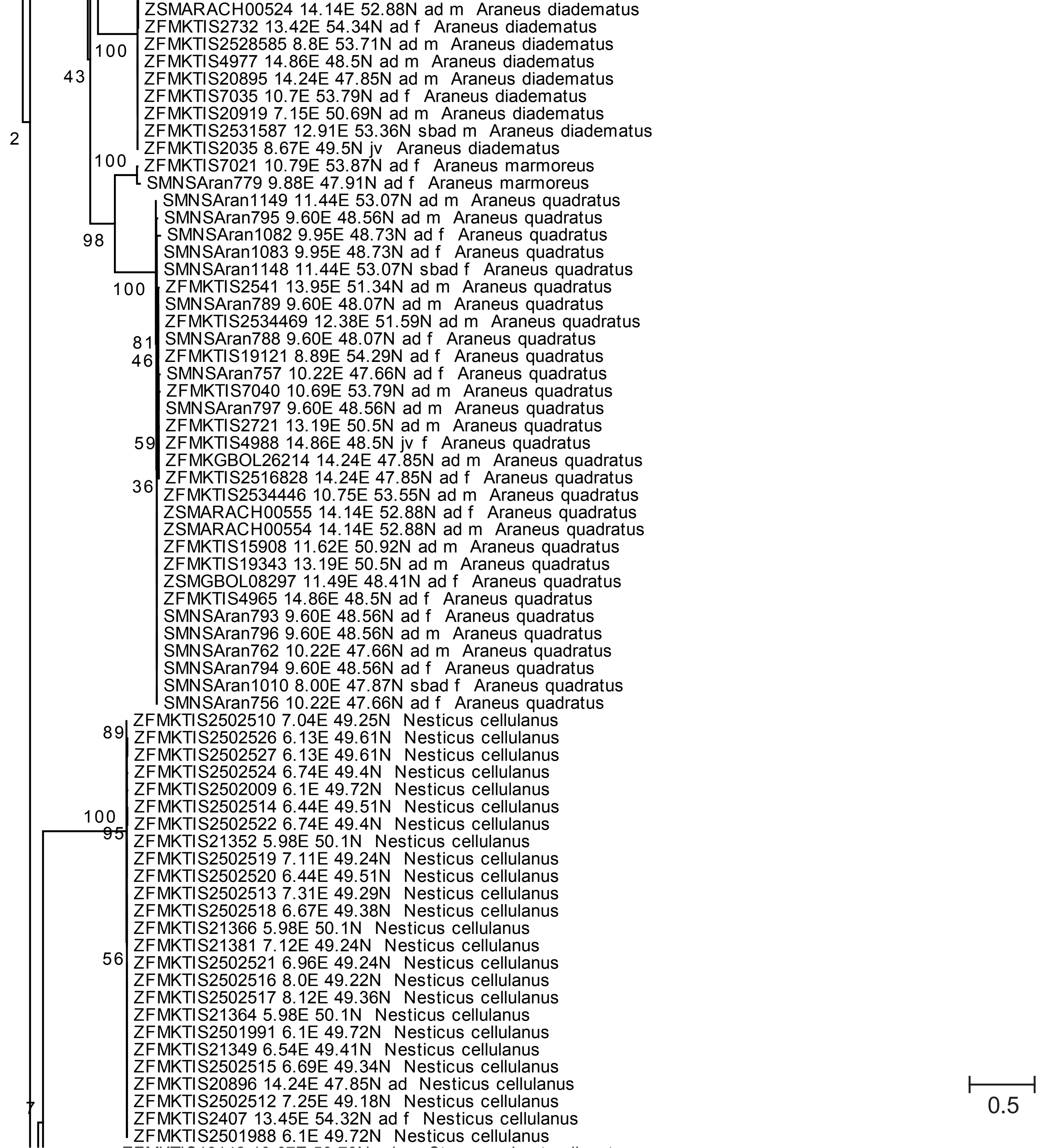
ZSMARACH00524 14.14E 52.88N ad m Araneus diadematus
ZFMKTIS2732 13.42E 54.34N ad f Araneus diadematus
ZFMKTIS2528585 8.8E 53.71N ad m Araneus diadematus
ZFMKTIS4977 14.86E 48.5N ad m Araneus diadematus
ZFMKTIS20895 14.24E 47.85N ad m Araneus diadematus
ZFMKTIS7035 10.7E 53.79N ad f Araneus diadematus
ZFMKTIS20919 7.15E 50.69N ad m Araneus diadematus
ZFMKTIS2531587 12.91E 53.36N sbad m Araneus diadematus
ZFMKTIS2035 8.67E 49.5N jv Araneus diadematus
ZFMKTIS7021 10.79E 53.87N ad f Araneus marmoreus
SMNSAran779 9.88E 47.91N ad f Araneus marmoreus
SMNSAran1149 11.44E 53.07N ad m Araneus quadratus
SMNSAran795 9.60E 48.56N ad m Araneus quadratus
SMNSAran1082 9.95E 48.73N ad f Araneus quadratus
SMNSAran1083 9.95E 48.73N ad f Araneus quadratus
SMNSAran1148 11.44E 53.07N sbad f Araneus quadratus
ZFMKTIS2541 13.95E 51.34N ad m Araneus quadratus
SMNSAran789 9.60E 48.07N ad m Araneus quadratus
ZFMKTIS2534469 12.38E 51.59N ad m Araneus quadratus
SMNSAran788 9.60E 48.07N ad f Araneus quadratus
ZFMKTIS19121 8.89E 54.29N ad f Araneus quadratus
SMNSAran757 10.22E 47.66N ad f Araneus quadratus
ZFMKTIS7040 10.69E 53.79N ad m Araneus quadratus
SMNSAran797 9.60E 48.56N ad m Araneus quadratus
ZFMKTIS2721 13.19E 50.5N ad m Araneus quadratus
ZFMKTIS4988 14.86E 48.5N jv f Araneus quadratus
ZFMKGBOL26214 14.24E 47.85N ad m Araneus quadratus
ZFMKTIS2516828 14.24E 47.85N ad f Araneus quadratus
ZFMKTIS2534446 10.75E 53.55N ad m Araneus quadratus
ZSMARACH00555 14.14E 52.88N ad f Araneus quadratus
ZSMARACH00554 14.14E 52.88N ad m Araneus quadratus
ZFMKTIS15908 11.62E 50.92N ad m Araneus quadratus
ZFMKTIS19343 13.19E 50.5N ad m Araneus quadratus
ZSMGBOL08297 11.49E 48.41N ad f Araneus quadratus
ZFMKTIS4965 14.86E 48.5N ad f Araneus quadratus
SMNSAran793 9.60E 48.56N ad f Araneus quadratus
SMNSAran796 9.60E 48.56N ad m Araneus quadratus
SMNSAran762 10.22E 47.66N ad m Araneus quadratus
SMNSAran794 9.60E 48.56N ad f Araneus quadratus
SMNSAran1010 8.00E 47.87N sbad f Araneus quadratus
SMNSAran756 10.22E 47.66N ad f Araneus quadratus
ZFMKTIS2502510 7.04E 49.25N Nesticus cellulanus
ZFMKTIS2502526 6.13E 49.61N Nesticus cellulanus
ZFMKTIS2502527 6.13E 49.61N Nesticus cellulanus
ZFMKTIS2502524 6.74E 49.4N Nesticus cellulanus
ZFMKTIS2502009 6.1E 49.72N Nesticus cellulanus
ZFMKTIS2502514 6.44E 49.51N Nesticus cellulanus
ZFMKTIS2502522 6.74E 49.4N Nesticus cellulanus
ZFMKTIS21352 5.98E 50.1N Nesticus cellulanus
ZFMKTIS2502519 7.11E 49.24N Nesticus cellulanus
ZFMKTIS2502520 6.44E 49.51N Nesticus cellulanus
ZFMKTIS2502513 7.31E 49.29N Nesticus cellulanus
ZFMKTIS2502518 6.67E 49.38N Nesticus cellulanus
ZFMKTIS21366 5.98E 50.1N Nesticus cellulanus
ZFMKTIS21381 7.12E 49.24N Nesticus cellulanus
ZFMKTIS2502521 6.96E 49.24N Nesticus cellulanus
ZFMKTIS2502516 8.0E 49.22N Nesticus cellulanus
ZFMKTIS2502517 8.12E 49.36N Nesticus cellulanus
ZFMKTIS21364 5.98E 50.1N Nesticus cellulanus
ZFMKTIS2501991 6.1E 49.72N Nesticus cellulanus
ZFMKTIS21349 6.54E 49.41N Nesticus cellulanus
ZFMKTIS2502515 6.69E 49.34N Nesticus cellulanus
ZFMKTIS20896 14.24E 47.85N ad Nesticus cellulanus
ZFMKTIS2502512 7.25E 49.18N Nesticus cellulanus
ZFMKTIS2407 13.45E 54.32N ad f Nesticus cellulanus
ZFMKTIS2501988 6.1E 49.72N Nesticus cellulanus
100
43
2
100
98
100
81
46
59
36
89
100
95
56
7
0.5

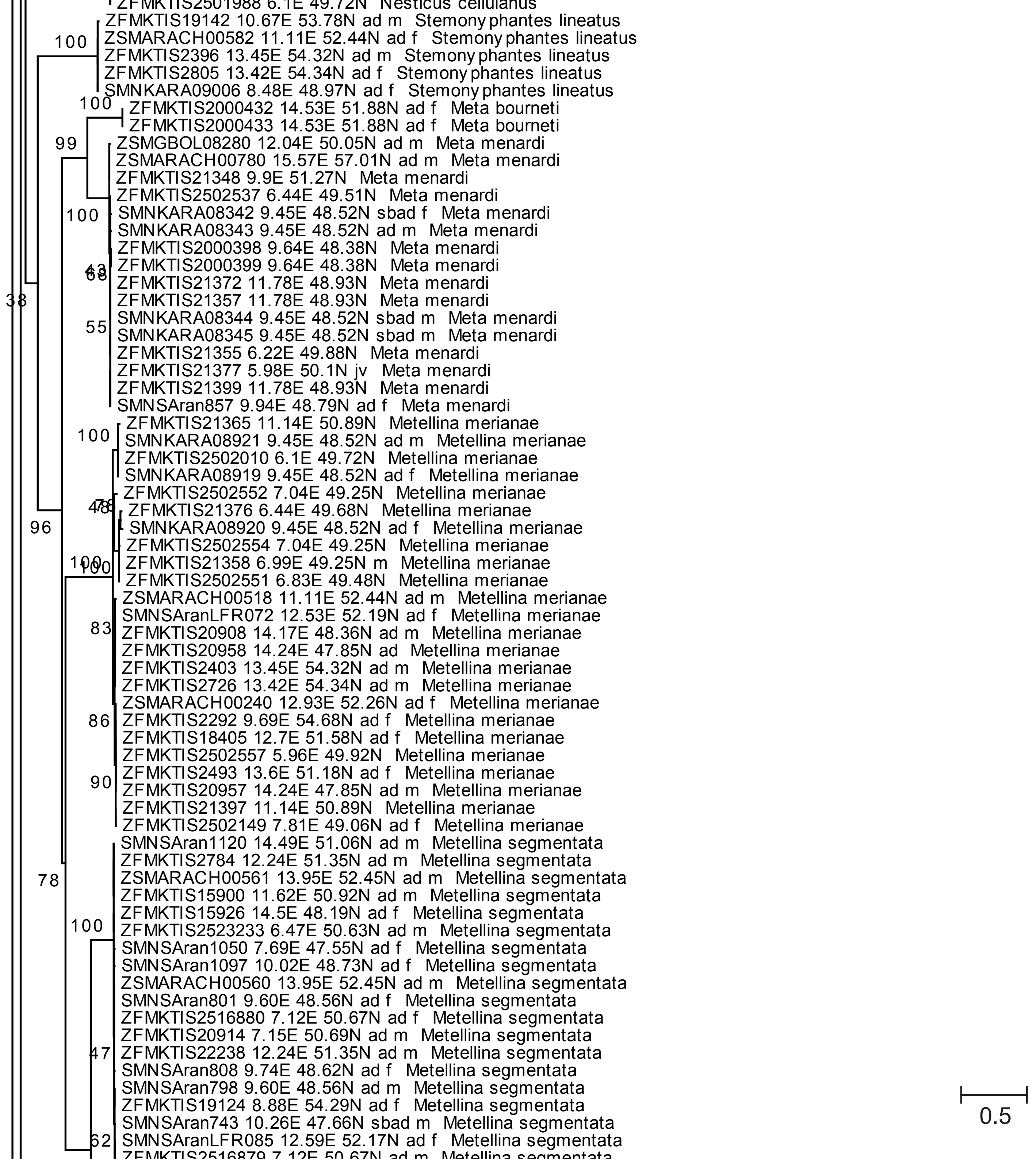
ZFMKTIS19142 10.67E 53.78N ad m Stemonyphantes lineatus
ZSMARACH00582 11.11E 52.44N ad f Stemonyphantes lineatus
ZFMKTIS2396 13.45E 54.32N ad m Stemonyphantes lineatus
ZFMKTIS2805 13.42E 54.34N ad f Stemonyphantes lineatus
SMNKARA09006 8.48E 48.97N ad f Stemonyphantes lineatus
ZFMKTIS2000432 14.53E 51.88N ad f Meta bourneti
ZFMKTIS2000433 14.53E 51.88N ad f Meta bourneti
ZSMGBOL08280 12.04E 50.05N ad m Meta menardi
ZSMARACH00780 15.57E 57.01N ad m Meta menardi
ZFMKTIS21348 9.9E 51.27N Meta menardi
ZFMKTIS2502537 6.44E 49.51N Meta menardi
SMNKARA08342 9.45E 48.52N sbad f Meta menardi
SMNKARA08343 9.45E 48.52N ad m Meta menardi
ZFMKTIS2000398 9.64E 48.38N Meta menardi
ZFMKTIS2000399 9.64E 48.38N Meta menardi
ZFMKTIS21372 11.78E 48.93N Meta menardi
ZFMKTIS21357 11.78E 48.93N Meta menardi
SMNKARA08344 9.45E 48.52N sbad m Meta menardi
SMNKARA08345 9.45E 48.52N sbad m Meta menardi
ZFMKTIS21355 6.22E 49.88N Meta menardi
ZFMKTIS21377 5.98E 50.1N jv Meta menardi
ZFMKTIS21399 11.78E 48.93N Meta menardi
SMNSAran857 9.94E 48.79N ad f Meta menardi
ZFMKTIS21365 11.14E 50.89N Metellina merianae
SMNKARA08921 9.45E 48.52N ad m Metellina merianae
ZFMKTIS2502010 6.1E 49.72N Metellina merianae
SMNKARA08919 9.45E 48.52N ad f Metellina merianae
ZFMKTIS2502552 7.04E 49.25N Metellina merianae
ZFMKTIS21376 6.44E 49.68N Metellina merianae
SMNKARA08920 9.45E 48.52N ad f Metellina merianae
ZFMKTIS2502554 7.04E 49.25N Metellina merianae
ZFMKTIS21358 6.99E 49.25N m Metellina merianae
ZFMKTIS2502551 6.83E 49.48N Metellina merianae
ZSMARACH00518 11.11E 52.44N ad m Metellina merianae
SMNSAranLFR072 12.53E 52.19N ad f Metellina merianae
ZFMKTIS20908 14.17E 48.36N ad m Metellina merianae
ZFMKTIS20958 14.24E 47.85N ad Metellina merianae
ZFMKTIS2403 13.45E 54.32N ad m Metellina merianae
ZFMKTIS2726 13.42E 54.34N ad m Metellina merianae
ZSMARACH00240 12.93E 52.26N ad f Metellina merianae
ZFMKTIS2292 9.69E 54.68N ad f Metellina merianae
ZFMKTIS18405 12.7E 51.58N ad f Metellina merianae
ZFMKTIS2502557 5.96E 49.92N Metellina merianae
ZFMKTIS2493 13.6E 51.18N ad f Metellina merianae
ZFMKTIS20957 14.24E 47.85N ad m Metellina merianae
ZFMKTIS21397 11.14E 50.89N Metellina merianae
ZFMKTIS2502149 7.81E 49.06N ad f Metellina merianae
SMNSAran1120 14.49E 51.06N ad m Metellina segmentata
ZFMKTIS2784 12.24E 51.35N ad m Metellina segmentata
ZSMARACH00561 13.95E 52.45N ad m Metellina segmentata
ZFMKTIS15900 11.62E 50.92N ad m Metellina segmentata
ZFMKTIS15926 14.5E 48.19N ad f Metellina segmentata
ZFMKTIS2523233 6.47E 50.63N ad m Metellina segmentata
SMNSAran1050 7.69E 47.55N ad f Metellina segmentata
SMNSAran1097 10.02E 48.73N ad f Metellina segmentata
ZSMARACH00560 13.95E 52.45N ad m Metellina segmentata
SMNSAran801 9.60E 48.56N ad f Metellina segmentata
ZFMKTIS2516880 7.12E 50.67N ad f Metellina segmentata
ZFMKTIS20914 7.15E 50.69N ad m Metellina segmentata
ZFMKTIS22238 12.24E 51.35N ad m Metellina segmentata
SMNSAran808 9.74E 48.62N ad f Metellina segmentata
SMNSAran798 9.60E 48.56N ad m Metellina segmentata
ZFMKTIS19124 8.88E 54.29N ad f Metellina segmentata
SMNSAran743 10.26E 47.66N sbad m Metellina segmentata
SMNSAranLFR085 12.59E 52.17N ad f Metellina segmentata
100
100
99
100
38
55
100
96
83
86
90
78
100
47
62
0.5

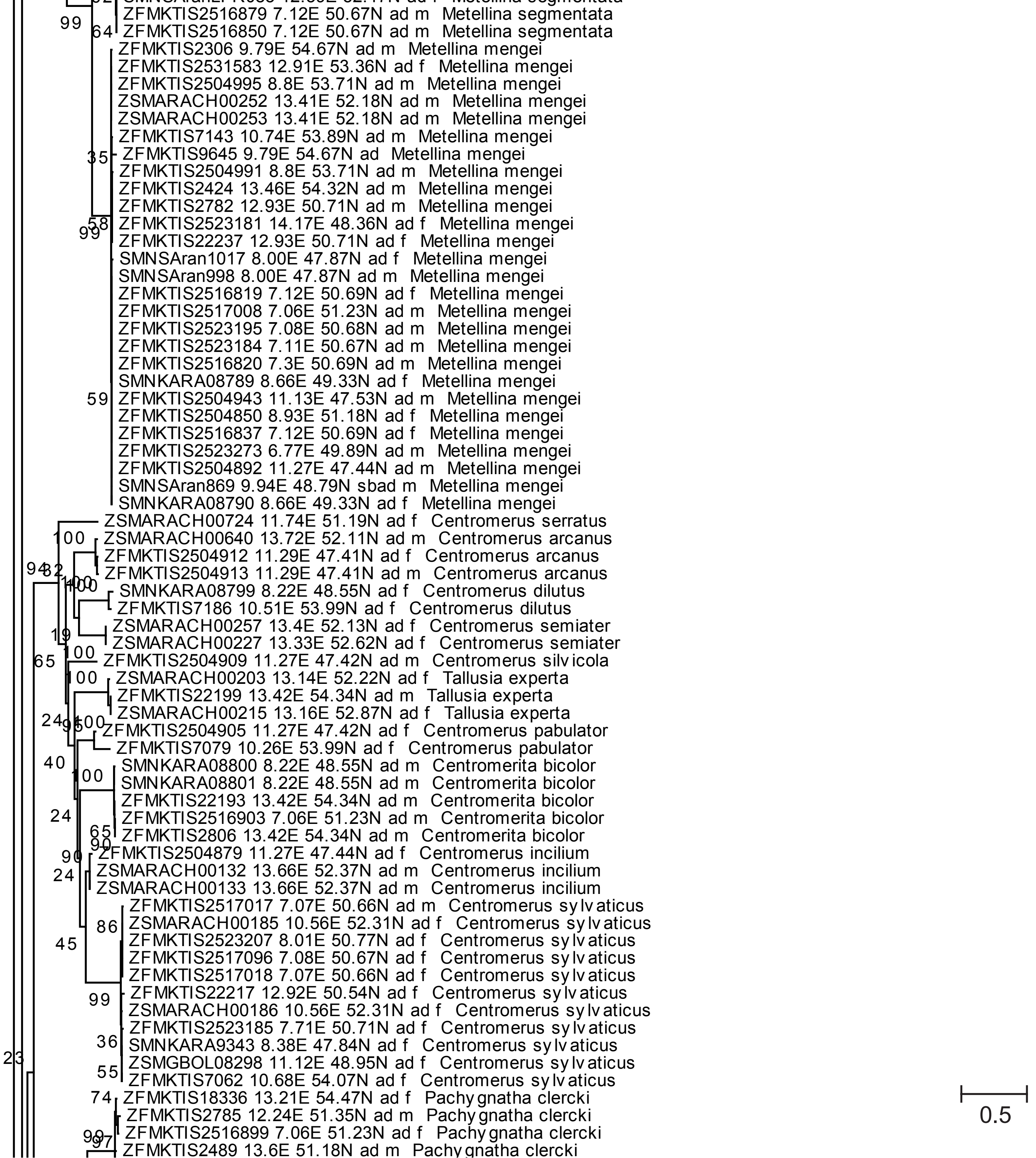

ZFMKTIS2516879 7.12E 50.67N ad m Metellina segmentata
ZFMKTIS2516850 7.12E 50.67N ad m Metellina segmentata
ZFMKTIS2306 9.79E 54.67N ad m Metellina mengei
ZFMKTIS2531583 12.91E 53.36N ad f Metellina mengei
ZFMKTIS2504995 8.8E 53.71N ad m Metellina mengei
ZSMARACH00252 13.41E 52.18N ad m Metellina mengei
ZSMARACH00253 13.41E 52.18N ad m Metellina mengei
ZFMKTIS7143 10.74E 53.89N ad m Metellina mengei
ZFMKTIS9645 9.79E 54.67N ad Metellina mengei
ZFMKTIS2504991 8.8E 53.71N ad m Metellina mengei
ZFMKTIS2424 13.46E 54.32N ad m Metellina mengei
ZFMKTIS2782 12.93E 50.71N ad m Metellina mengei
ZFMKTIS2523181 14.17E 48.36N ad f Metellina mengei
ZFMKTIS22237 12.93E 50.71N ad f Metellina mengei
SMNSAran1017 8.00E 47.87N ad f Metellina mengei
SMNSAran998 8.00E 47.87N ad m Metellina mengei
ZFMKTIS2516819 7.12E 50.69N ad f Metellina mengei
ZFMKTIS2517008 7.06E 51.23N ad m Metellina mengei
ZFMKTIS2523195 7.08E 50.68N ad m Metellina mengei
ZFMKTIS2523184 7.11E 50.67N ad m Metellina mengei
ZFMKTIS2516820 7.3E 50.69N ad m Metellina mengei
SMNKARA08789 8.66E 49.33N ad f Metellina mengei
ZFMKTIS2504943 11.13E 47.53N ad m Metellina mengei
ZFMKTIS2504850 8.93E 51.18N ad f Metellina mengei
ZFMKTIS2516837 7.12E 50.69N ad f Metellina mengei
ZFMKTIS2523273 6.77E 49.89N ad m Metellina mengei
ZFMKTIS2504892 11.27E 47.44N ad m Metellina mengei
SMNSAran869 9.94E 48.79N sbad m Metellina mengei
SMNKARA08790 8.66E 49.33N ad f Metellina mengei
ZSMARACH00724 11.74E 51.19N ad f Centromerus serratus
ZSMARACH00640 13.72E 52.11N ad m Centromerus arcanus
ZFMKTIS2504912 11.29E 47.41N ad f Centromerus arcanus
ZFMKTIS2504913 11.29E 47.41N ad m Centromerus arcanus
SMNKARA08799 8.22E 48.55N ad f Centromerus dilutus
ZFMKTIS7186 10.51E 53.99N ad f Centromerus dilutus
ZSMARACH00257 13.4E 52.13N ad f Centromerus semiater
ZSMARACH00227 13.33E 52.62N ad f Centromerus semiater
ZFMKTIS2504909 11.27E 47.42N ad m Centromerus silvicola
ZSMARACH00203 13.14E 52.22N ad f Tallusia experta
ZFMKTIS22199 13.42E 54.34N ad m Tallusia experta
ZSMARACH00215 13.16E 52.87N ad f Tallusia experta
ZFMKTIS2504905 11.27E 47.42N ad f Centromerus pabulator
ZFMKTIS7079 10.26E 53.99N ad f Centromerus pabulator
SMNKARA08800 8.22E 48.55N ad m Centromerita bicolor
SMNKARA08801 8.22E 48.55N ad m Centromerita bicolor
ZFMKTIS22193 13.42E 54.34N ad m Centromerita bicolor
ZFMKTIS2516903 7.06E 51.23N ad m Centromerita bicolor
ZFMKTIS2806 13.42E 54.34N ad m Centromerita bicolor
ZFMKTIS2504879 11.27E 47.44N ad f Centromerus incilium
ZSMARACH00132 13.66E 52.37N ad m Centromerus incilium
ZSMARACH00133 13.66E 52.37N ad m Centromerus incilium
ZFMKTIS2517017 7.07E 50.66N ad m Centromerus sylvaticus
ZSMARACH00185 10.56E 52.31N ad f Centromerus sylvaticus
ZFMKTIS2523207 8.01E 50.77N ad f Centromerus sylvaticus
ZFMKTIS2517096 7.08E 50.67N ad f Centromerus sylvaticus
ZFMKTIS2517018 7.07E 50.66N ad f Centromerus sylvaticus
ZFMKTIS22217 12.92E 50.54N ad f Centromerus sylvaticus
ZSMARACH00186 10.56E 52.31N ad f Centromerus sylvaticus
ZFMKTIS2523185 7.71E 50.71N ad f Centromerus sylvaticus
SMNKARA9343 8.38E 47.84N ad f Centromerus sylvaticus
ZSMGBOL08298 11.12E 48.95N ad f Centromerus sylvaticus
ZFMKTIS7062 10.68E 54.07N ad f Centromerus sylvaticus
ZFMKTIS18336 13.21E 54.47N ad f Pachygnatha clercki
ZFMKTIS2785 12.24E 51.35N ad m Pachygnatha clercki
ZFMKTIS2516899 7.06E 51.23N ad f Pachygnatha clercki
ZFMKTIS2489 13.6E 51.18N ad m Pachygnatha clercki
0.5

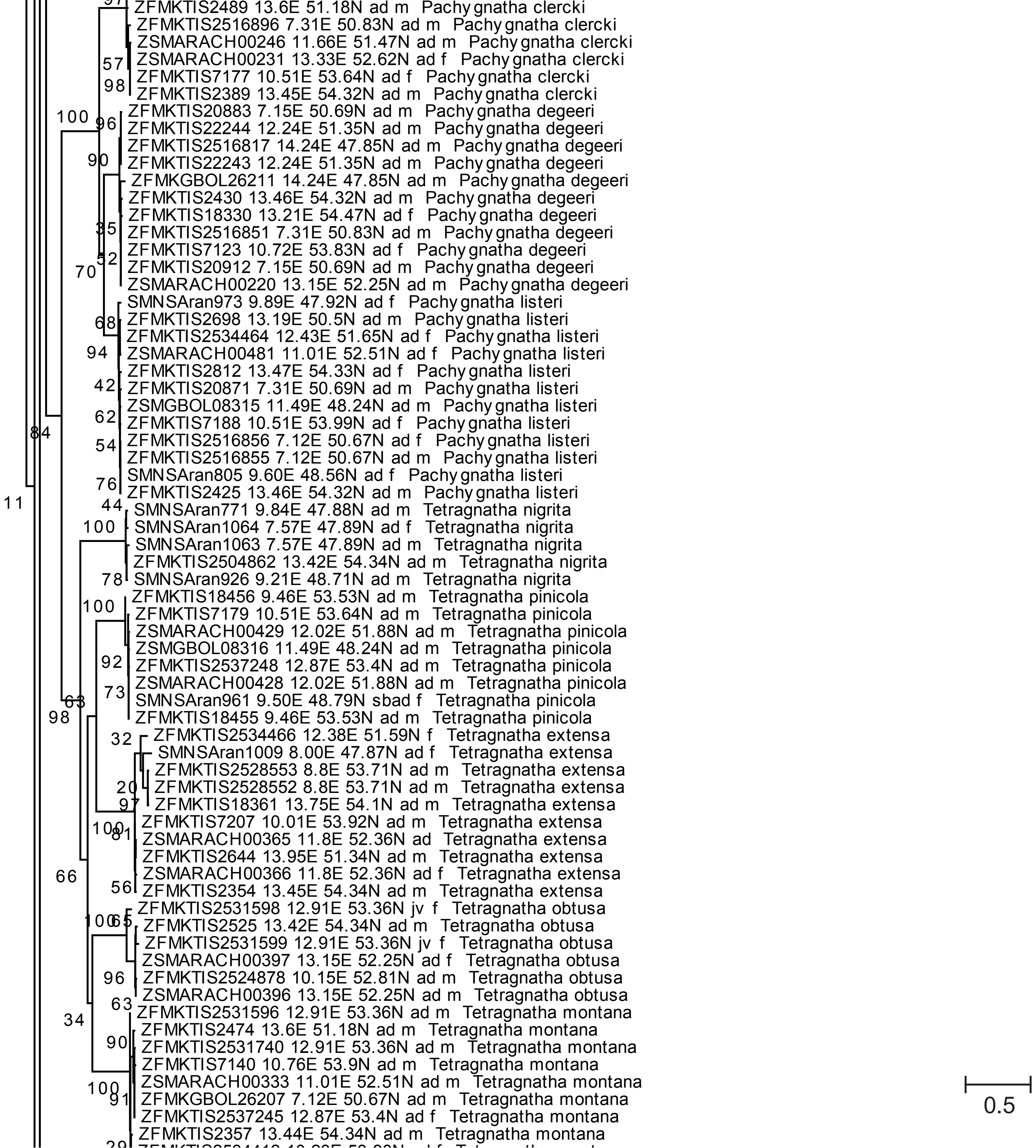
ZFMKTIS2489 13.6E 51.18N ad m Pachygnatha clercki
ZFMKTIS2516896 7.31E 50.83N ad m Pachygnatha clercki
ZSMARACH00246 11.66E 51.47N ad m Pachygnatha clercki
ZSMARACH00231 13.33E 52.62N ad f Pachygnatha clercki
ZFMKTIS7177 10.51E 53.64N ad f Pachygnatha clercki
ZFMKTIS2389 13.45E 54.32N ad m Pachygnatha clercki
ZFMKTIS20883 7.15E 50.69N ad m Pachygnatha degeeri
ZFMKTIS22244 12.24E 51.35N ad m Pachygnatha degeeri
ZFMKTIS2516817 14.24E 47.85N ad m Pachygnatha degeeri
ZFMKTIS22243 12.24E 51.35N ad m Pachygnatha degeeri
ZFMKGBOL26211 14.24E 47.85N ad m Pachygnatha degeeri
ZFMKTIS2430 13.46E 54.32N ad m Pachygnatha degeeri
ZFMKTIS18330 13.21E 54.47N ad f Pachygnatha degeeri
ZFMKTIS2516851 7.31E 50.83N ad m Pachygnatha degeeri
ZFMKTIS7123 10.72E 53.83N ad f Pachygnatha degeeri
ZFMKTIS20912 7.15E 50.69N ad m Pachygnatha degeeri
ZSMARACH00220 13.15E 52.25N ad m Pachygnatha degeeri
SMNSAran973 9.89E 47.92N ad f Pachygnatha listeri
ZFMKTIS2698 13.19E 50.5N ad m Pachygnatha listeri
ZFMKTIS2534464 12.43E 51.65N ad f Pachygnatha listeri
ZSMARACH00481 11.01E 52.51N ad f Pachygnatha listeri
ZFMKTIS2812 13.47E 54.33N ad f Pachygnatha listeri
ZFMKTIS20871 7.31E 50.69N ad m Pachygnatha listeri
ZSMGBOL08315 11.49E 48.24N ad m Pachygnatha listeri
ZFMKTIS7188 10.51E 53.99N ad f Pachygnatha listeri
ZFMKTIS2516856 7.12E 50.67N ad f Pachygnatha listeri
ZFMKTIS2516855 7.12E 50.67N ad m Pachygnatha listeri
SMNSAran805 9.60E 48.56N ad f Pachygnatha listeri
ZFMKTIS2425 13.46E 54.32N ad m Pachygnatha listeri
SMNSAran771 9.84E 47.88N ad m Tetragnatha nigrita
SMNSAran1064 7.57E 47.89N ad f Tetragnatha nigrita
SMNSAran1063 7.57E 47.89N ad m Tetragnatha nigrita
ZFMKTIS2504862 13.42E 54.34N ad m Tetragnatha nigrita
SMNSAran926 9.21E 48.71N ad m Tetragnatha nigrita
ZFMKTIS18456 9.46E 53.53N ad m Tetragnatha pinicola
ZFMKTIS7179 10.51E 53.64N ad m Tetragnatha pinicola
ZSMARACH00429 12.02E 51.88N ad m Tetragnatha pinicola
ZSMGBOL08316 11.49E 48.24N ad m Tetragnatha pinicola
ZFMKTIS2537248 12.87E 53.4N ad m Tetragnatha pinicola
ZSMARACH00428 12.02E 51.88N ad m Tetragnatha pinicola
SMNSAran961 9.50E 48.79N sbad f Tetragnatha pinicola
ZFMKTIS18455 9.46E 53.53N ad m Tetragnatha pinicola
ZFMKTIS2534466 12.38E 51.59N f Tetragnatha extensa
SMNSAran1009 8.00E 47.87N ad f Tetragnatha extensa
ZFMKTIS2528553 8.8E 53.71N ad m Tetragnatha extensa
ZFMKTIS2528552 8.8E 53.71N ad m Tetragnatha extensa
ZFMKTIS18361 13.75E 54.1N ad m Tetragnatha extensa
ZFMKTIS7207 10.01E 53.92N ad m Tetragnatha extensa
ZSMARACH00365 11.8E 52.36N ad Tetragnatha extensa
ZFMKTIS2644 13.95E 51.34N ad m Tetragnatha extensa
ZSMARACH00366 11.8E 52.36N ad f Tetragnatha extensa
ZFMKTIS2354 13.45E 54.34N ad m Tetragnatha extensa
ZFMKTIS2531598 12.91E 53.36N jv f Tetragnatha obtusa
ZFMKTIS2525 13.42E 54.34N ad m Tetragnatha obtusa
ZFMKTIS2531599 12.91E 53.36N jv f Tetragnatha obtusa
ZSMARACH00397 13.15E 52.25N ad f Tetragnatha obtusa
ZFMKTIS2524878 10.15E 52.81N ad m Tetragnatha obtusa
ZSMARACH00396 13.15E 52.25N ad m Tetragnatha obtusa
ZFMKTIS2531596 12.91E 53.36N ad m Tetragnatha montana
ZFMKTIS2474 13.6E 51.18N ad m Tetragnatha montana
ZFMKTIS2531740 12.91E 53.36N ad m Tetragnatha montana
ZFMKTIS7140 10.76E 53.9N ad m Tetragnatha montana
ZSMARACH00333 11.01E 52.51N ad m Tetragnatha montana
ZFMKGBOL26207 7.12E 50.67N ad m Tetragnatha montana
ZFMKTIS2537245 12.87E 53.4N ad f Tetragnatha montana
ZFMKTIS2357 13.44E 54.34N ad m Tetragnatha montana
0.5

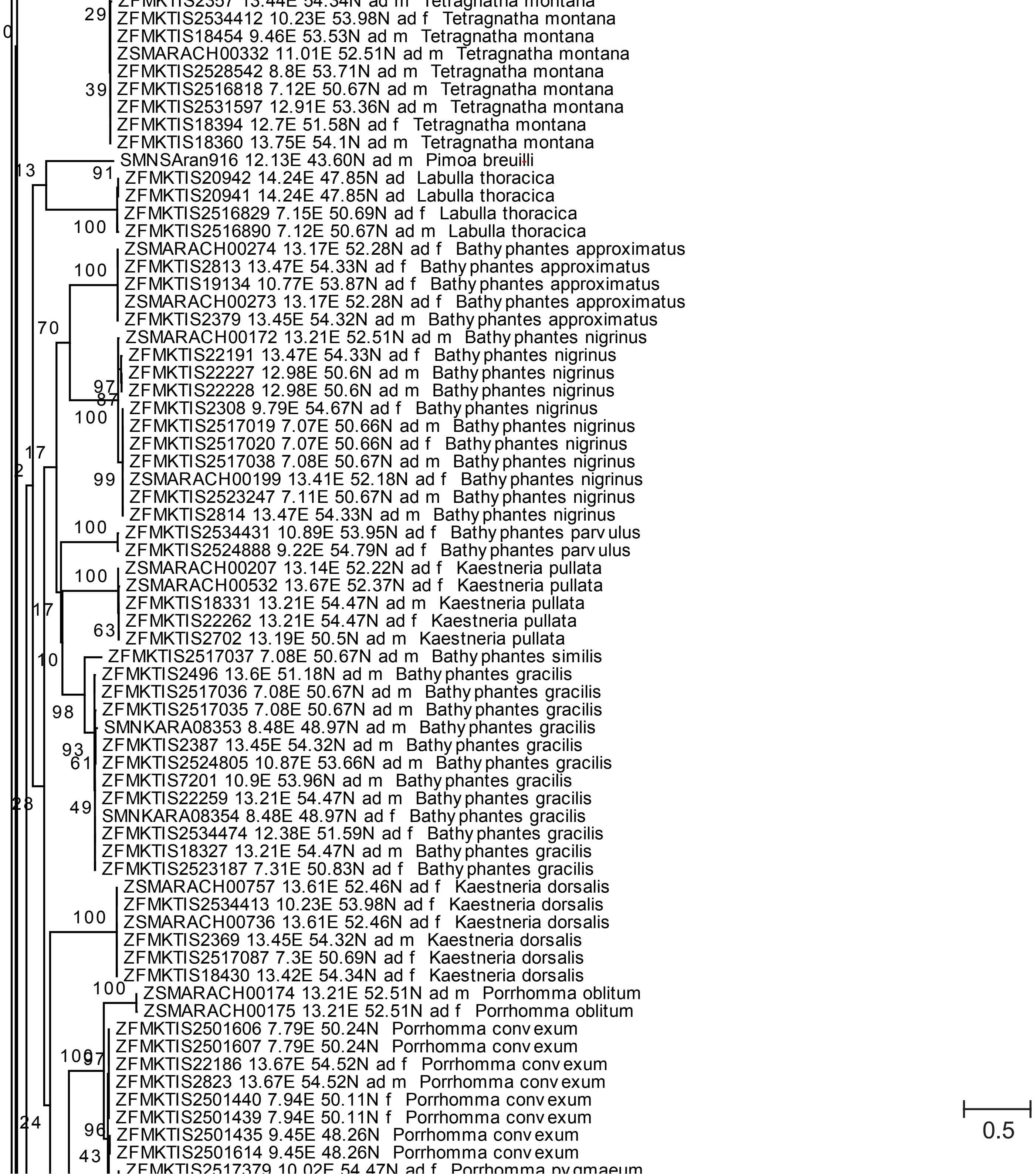
ZFMKTIS2534412 10.23E 53.98N ad f Tetragnatha montana
ZFMKTIS18454 9.46E 53.53N ad m Tetragnatha montana
ZSMARACH00332 11.01E 52.51N ad m Tetragnatha montana
ZFMKTIS2528542 8.8E 53.71N ad m Tetragnatha montana
ZFMKTIS2516818 7.12E 50.67N ad m Tetragnatha montana
ZFMKTIS2531597 12.91E 53.36N ad m Tetragnatha montana
ZFMKTIS18394 12.7E 51.58N ad f Tetragnatha montana
ZFMKTIS18360 13.75E 54.1N ad m Tetragnatha montana
SMNSAran916 12.13E 43.60N ad m Pimoa breuilli
ZFMKTIS20942 14.24E 47.85N ad Labulla thoracica
ZFMKTIS20941 14.24E 47.85N ad Labulla thoracica
ZFMKTIS2516829 7.15E 50.69N ad f Labulla thoracica
ZFMKTIS2516890 7.12E 50.67N ad m Labulla thoracica
ZSMARACH00274 13.17E 52.28N ad f Bathyphantes approximatus
ZFMKTIS2813 13.47E 54.33N ad f Bathyphantes approximatus
ZFMKTIS19134 10.77E 53.87N ad f Bathyphantes approximatus
ZSMARACH00273 13.17E 52.28N ad f Bathyphantes approximatus
ZFMKTIS2379 13.45E 54.32N ad m Bathyphantes approximatus
ZSMARACH00172 13.21E 52.51N ad m Bathyphantes nigrinus
ZFMKTIS22191 13.47E 54.33N ad f Bathyphantes nigrinus
ZFMKTIS22227 12.98E 50.6N ad m Bathyphantes nigrinus
ZFMKTIS22228 12.98E 50.6N ad m Bathyphantes nigrinus
ZFMKTIS2308 9.79E 54.67N ad f Bathyphantes nigrinus
ZFMKTIS2517019 7.07E 50.66N ad m Bathyphantes nigrinus
ZFMKTIS2517020 7.07E 50.66N ad f Bathyphantes nigrinus
ZFMKTIS2517038 7.08E 50.67N ad m Bathyphantes nigrinus
ZSMARACH00199 13.41E 52.18N ad f Bathyphantes nigrinus
ZFMKTIS2523247 7.11E 50.67N ad m Bathyphantes nigrinus
ZFMKTIS2814 13.47E 54.33N ad m Bathyphantes nigrinus
ZFMKTIS2534431 10.89E 53.95N ad f Bathyphantes parvulus
ZFMKTIS2524888 9.22E 54.79N ad f Bathyphantes parvulus
ZSMARACH00207 13.14E 52.22N ad f Kaestneria pullata
ZSMARACH00532 13.67E 52.37N ad f Kaestneria pullata
ZFMKTIS18331 13.21E 54.47N ad m Kaestneria pullata
ZFMKTIS22262 13.21E 54.47N ad f Kaestneria pullata
ZFMKTIS2702 13.19E 50.5N ad m Kaestneria pullata
ZFMKTIS2517037 7.08E 50.67N ad m Bathyphantes similis
ZFMKTIS2496 13.6E 51.18N ad m Bathyphantes gracilis
ZFMKTIS2517036 7.08E 50.67N ad m Bathyphantes gracilis
ZFMKTIS2517035 7.08E 50.67N ad m Bathyphantes gracilis
SMNKARA08353 8.48E 48.97N ad m Bathyphantes gracilis
ZFMKTIS2387 13.45E 54.32N ad m Bathyphantes gracilis
ZFMKTIS2524805 10.87E 53.66N ad m Bathyphantes gracilis
ZFMKTIS7201 10.9E 53.96N ad m Bathyphantes gracilis
ZFMKTIS22259 13.21E 54.47N ad m Bathyphantes gracilis
SMNKARA08354 8.48E 48.97N ad f Bathyphantes gracilis
ZFMKTIS2534474 12.38E 51.59N ad f Bathyphantes gracilis
ZFMKTIS18327 13.21E 54.47N ad m Bathyphantes gracilis
ZFMKTIS2523187 7.31E 50.83N ad f Bathyphantes gracilis
ZSMARACH00757 13.61E 52.46N ad f Kaestneria dorsalis
ZFMKTIS2534413 10.23E 53.98N ad f Kaestneria dorsalis
ZSMARACH00736 13.61E 52.46N ad f Kaestneria dorsalis
ZFMKTIS2369 13.45E 54.32N ad m Kaestneria dorsalis
ZFMKTIS2517087 7.3E 50.69N ad f Kaestneria dorsalis
ZFMKTIS18430 13.42E 54.34N ad f Kaestneria dorsalis
ZSMARACH00174 13.21E 52.51N ad m Porrhomma oblitum
ZSMARACH00175 13.21E 52.51N ad f Porrhomma oblitum
ZFMKTIS2501606 7.79E 50.24N Porrhomma convexum
ZFMKTIS2501607 7.79E 50.24N Porrhomma convexum
ZFMKTIS22186 13.67E 54.52N ad f Porrhomma convexum
ZFMKTIS2823 13.67E 54.52N ad m Porrhomma convexum
ZFMKTIS2501440 7.94E 50.11N f Porrhomma convexum
ZFMKTIS2501439 7.94E 50.11N f Porrhomma convexum
ZFMKTIS2501435 9.45E 48.26N Porrhomma convexum
ZFMKTIS2501614 9.45E 48.26N Porrhomma convexum
0.5

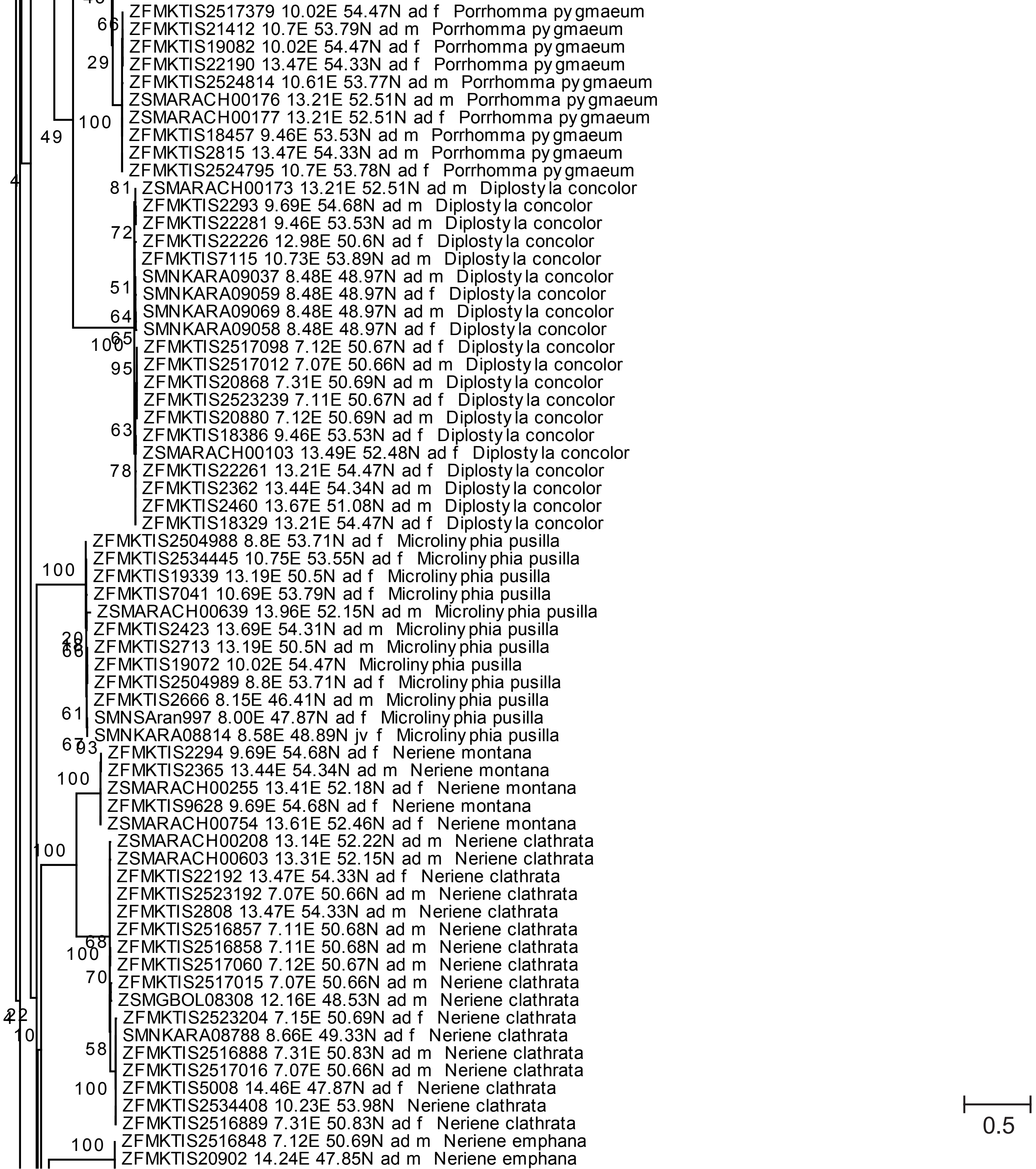

ZFMKTIS2517379 10.02E 54.47N ad f Porrhomma pygmaeum
ZFMKTIS21412 10.7E 53.79N ad m Porrhomma pygmaeum
ZFMKTIS19082 10.02E 54.47N ad f Porrhomma pygmaeum
ZFMKTIS22190 13.47E 54.33N ad f Porrhomma pygmaeum
ZFMKTIS2524814 10.61E 53.77N ad m Porrhomma pygmaeum
ZSMARACH00176 13.21E 52.51N ad m Porrhomma pygmaeum
ZSMARACH00177 13.21E 52.51N ad f Porrhomma pygmaeum
ZFMKTIS18457 9.46E 53.53N ad m Porrhomma pygmaeum
ZFMKTIS2815 13.47E 54.33N ad m Porrhomma pygmaeum
ZFMKTIS2524795 10.7E 53.78N ad f Porrhomma pygmaeum
ZSMARACH00173 13.21E 52.51N ad m Diplostyla concolor
ZFMKTIS2293 9.69E 54.68N ad m Diplostyla concolor
ZFMKTIS22281 9.46E 53.53N ad m Diplostyla concolor
ZFMKTIS22226 12.98E 50.6N ad f Diplostyla concolor
ZFMKTIS7115 10.73E 53.89N ad m Diplostyla concolor
SMNKARA09037 8.48E 48.97N ad m Diplostyla concolor
SMNKARA09059 8.48E 48.97N ad f Diplostyla concolor
SMNKARA09069 8.48E 48.97N ad m Diplostyla concolor
SMNKARA09058 8.48E 48.97N ad f Diplostyla concolor
ZFMKTIS2517098 7.12E 50.67N ad f Diplostyla concolor
ZFMKTIS2517012 7.07E 50.66N ad m Diplostyla concolor
ZFMKTIS20868 7.31E 50.69N ad m Diplostyla concolor
ZFMKTIS2523239 7.11E 50.67N ad f Diplostyla concolor
ZFMKTIS20880 7.12E 50.69N ad m Diplostyla concolor
ZFMKTIS18386 9.46E 53.53N ad f Diplostyla concolor
ZSMARACH00103 13.49E 52.48N ad f Diplostyla concolor
ZFMKTIS22261 13.21E 54.47N ad f Diplostyla concolor
ZFMKTIS2362 13.44E 54.34N ad m Diplostyla concolor
ZFMKTIS2460 13.67E 51.08N ad m Diplostyla concolor
ZFMKTIS18329 13.21E 54.47N ad f Diplostyla concolor
ZFMKTIS2504988 8.8E 53.71N ad f Microlinyphia pusilla
ZFMKTIS2534445 10.75E 53.55N ad f Microlinyphia pusilla
ZFMKTIS19339 13.19E 50.5N ad f Microlinyphia pusilla
ZFMKTIS7041 10.69E 53.79N ad f Microlinyphia pusilla
ZSMARACH00639 13.96E 52.15N ad m Microlinyphia pusilla
ZFMKTIS2423 13.69E 54.31N ad m Microlinyphia pusilla
ZFMKTIS2713 13.19E 50.5N ad m Microlinyphia pusilla
ZFMKTIS19072 10.02E 54.47N Microlinyphia pusilla
ZFMKTIS2504989 8.8E 53.71N ad f Microlinyphia pusilla
ZFMKTIS2666 8.15E 46.41N ad m Microlinyphia pusilla
SMNSAran997 8.00E 47.87N ad f Microlinyphia pusilla
SMNKARA08814 8.58E 48.89N jv f Microlinyphia pusilla
ZFMKTIS2294 9.69E 54.68N ad f Neriene montana
ZFMKTIS2365 13.44E 54.34N ad m Neriene montana
ZSMARACH00255 13.41E 52.18N ad f Neriene montana
ZFMKTIS9628 9.69E 54.68N ad f Neriene montana
ZSMARACH00754 13.61E 52.46N ad f Neriene montana
ZSMARACH00208 13.14E 52.22N ad m Neriene clathrata
ZSMARACH00603 13.31E 52.15N ad m Neriene clathrata
ZFMKTIS22192 13.47E 54.33N ad f Neriene clathrata
ZFMKTIS2523192 7.07E 50.66N ad m Neriene clathrata
ZFMKTIS2808 13.47E 54.33N ad m Neriene clathrata
ZFMKTIS2516857 7.11E 50.68N ad m Neriene clathrata
ZFMKTIS2516858 7.11E 50.68N ad m Neriene clathrata
ZFMKTIS2517060 7.12E 50.67N ad m Neriene clathrata
ZFMKTIS2517015 7.07E 50.66N ad m Neriene clathrata
ZSMGBOL08308 12.16E 48.53N ad m Neriene clathrata
ZFMKTIS2523204 7.15E 50.69N ad f Neriene clathrata
SMNKARA08788 8.66E 49.33N ad f Neriene clathrata
ZFMKTIS2516888 7.31E 50.83N ad m Neriene clathrata
ZFMKTIS2517016 7.07E 50.66N ad m Neriene clathrata
ZFMKTIS5008 14.46E 47.87N ad f Neriene clathrata
ZFMKTIS2534408 10.23E 53.98N Neriene clathrata
ZFMKTIS2516889 7.31E 50.83N ad f Neriene clathrata
ZFMKTIS2516848 7.12E 50.69N ad m Neriene emphana
ZFMKTIS20902 14.24E 47.85N ad m Neriene emphana
0.5

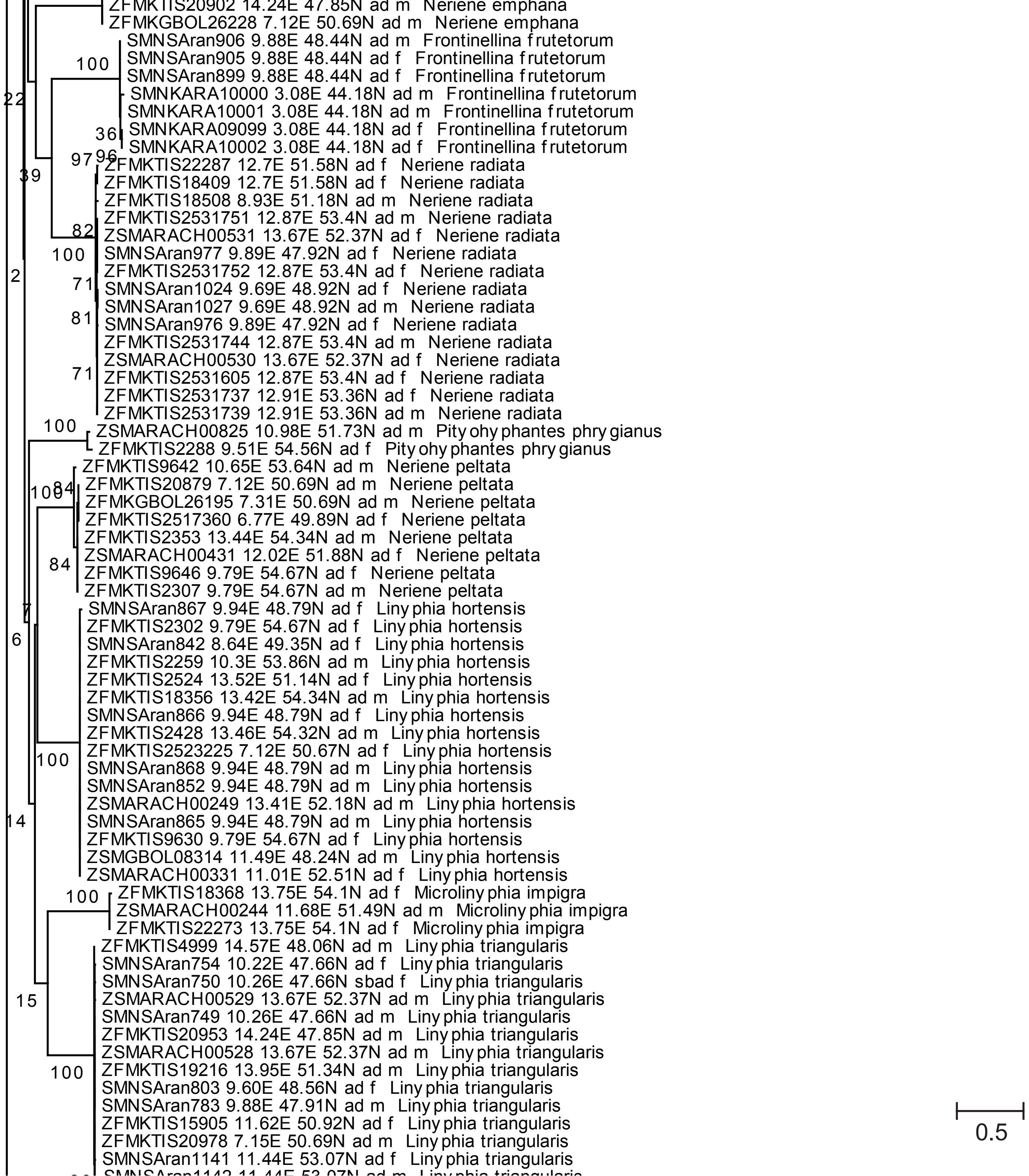

ZFMKTIS20902 14.24E 47.85N ad m Neriene emphana
ZFMKGBOL26228 7.12E 50.69N ad m Neriene emphana
SMNSAran906 9.88E 48.44N ad m Frontinellina frutetorum
SMNSAran905 9.88E 48.44N ad f Frontinellina frutetorum
SMNSAran899 9.88E 48.44N ad f Frontinellina frutetorum
SMNKARA10000 3.08E 44.18N ad m Frontinellina frutetorum
SMNKARA10001 3.08E 44.18N ad m Frontinellina frutetorum
SMNKARA09099 3.08E 44.18N ad f Frontinellina frutetorum
SMNKARA10002 3.08E 44.18N ad f Frontinellina frutetorum
ZFMKTIS22287 12.7E 51.58N ad f Neriene radiata
ZFMKTIS18409 12.7E 51.58N ad f Neriene radiata
ZFMKTIS18508 8.93E 51.18N ad m Neriene radiata
ZFMKTIS2531751 12.87E 53.4N ad m Neriene radiata
ZSMARACH00531 13.67E 52.37N ad f Neriene radiata
SMNSAran977 9.89E 47.92N ad f Neriene radiata
ZFMKTIS2531752 12.87E 53.4N ad f Neriene radiata
SMNSAran1024 9.69E 48.92N ad f Neriene radiata
SMNSAran1027 9.69E 48.92N ad m Neriene radiata
SMNSAran976 9.89E 47.92N ad f Neriene radiata
ZFMKTIS2531744 12.87E 53.4N ad m Neriene radiata
ZSMARACH00530 13.67E 52.37N ad f Neriene radiata
ZFMKTIS2531605 12.87E 53.4N ad f Neriene radiata
ZFMKTIS2531737 12.91E 53.36N ad f Neriene radiata
ZFMKTIS2531739 12.91E 53.36N ad m Neriene radiata
ZSMARACH00825 10.98E 51.73N ad m Pityohyphantes phrygianus
ZFMKTIS2288 9.51E 54.56N ad f Pityohyphantes phrygianus
ZFMKTIS9642 10.65E 53.64N ad m Neriene peltata
ZFMKTIS20879 7.12E 50.69N ad m Neriene peltata
ZFMKGBOL26195 7.31E 50.69N ad m Neriene peltata
ZFMKTIS2517360 6.77E 49.89N ad f Neriene peltata
ZFMKTIS2353 13.44E 54.34N ad m Neriene peltata
ZSMARACH00431 12.02E 51.88N ad f Neriene peltata
ZFMKTIS9646 9.79E 54.67N ad f Neriene peltata
ZFMKTIS2307 9.79E 54.67N ad m Neriene peltata
SMNSAran867 9.94E 48.79N ad f Linyphia hortensis
ZFMKTIS2302 9.79E 54.67N ad f Linyphia hortensis
SMNSAran842 8.64E 49.35N ad f Linyphia hortensis
ZFMKTIS2259 10.3E 53.86N ad m Linyphia hortensis
ZFMKTIS2524 13.52E 51.14N ad f Linyphia hortensis
ZFMKTIS18356 13.42E 54.34N ad m Linyphia hortensis
SMNSAran866 9.94E 48.79N ad f Linyphia hortensis
ZFMKTIS2428 13.46E 54.32N ad m Linyphia hortensis
ZFMKTIS2523225 7.12E 50.67N ad f Linyphia hortensis
SMNSAran868 9.94E 48.79N ad m Linyphia hortensis
SMNSAran852 9.94E 48.79N ad m Linyphia hortensis
ZSMARACH00249 13.41E 52.18N ad m Linyphia hortensis
SMNSAran865 9.94E 48.79N ad m Linyphia hortensis
ZFMKTIS9630 9.79E 54.67N ad f Linyphia hortensis
ZSMGBOL08314 11.49E 48.24N ad m Linyphia hortensis
ZSMARACH00331 11.01E 52.51N ad f Linyphia hortensis
ZFMKTIS18368 13.75E 54.1N ad f Microlinyphia impigra
ZSMARACH00244 11.68E 51.49N ad m Microlinyphia impigra
ZFMKTIS22273 13.75E 54.1N ad f Microlinyphia impigra
ZFMKTIS4999 14.57E 48.06N ad m Linyphia triangularis
SMNSAran754 10.22E 47.66N ad f Linyphia triangularis
SMNSAran750 10.26E 47.66N sbad f Linyphia triangularis
ZSMARACH00529 13.67E 52.37N ad m Linyphia triangularis
SMNSAran749 10.26E 47.66N ad m Linyphia triangularis
ZFMKTIS20953 14.24E 47.85N ad m Linyphia triangularis
ZSMARACH00528 13.67E 52.37N ad m Linyphia triangularis
ZFMKTIS19216 13.95E 51.34N ad m Linyphia triangularis
SMNSAran803 9.60E 48.56N ad f Linyphia triangularis
SMNSAran783 9.88E 47.91N ad m Linyphia triangularis
ZFMKTIS15905 11.62E 50.92N ad f Linyphia triangularis
ZFMKTIS20978 7.15E 50.69N ad m Linyphia triangularis
SMNSAran1141 11.44E 53.07N ad f Linyphia triangularis
0.5

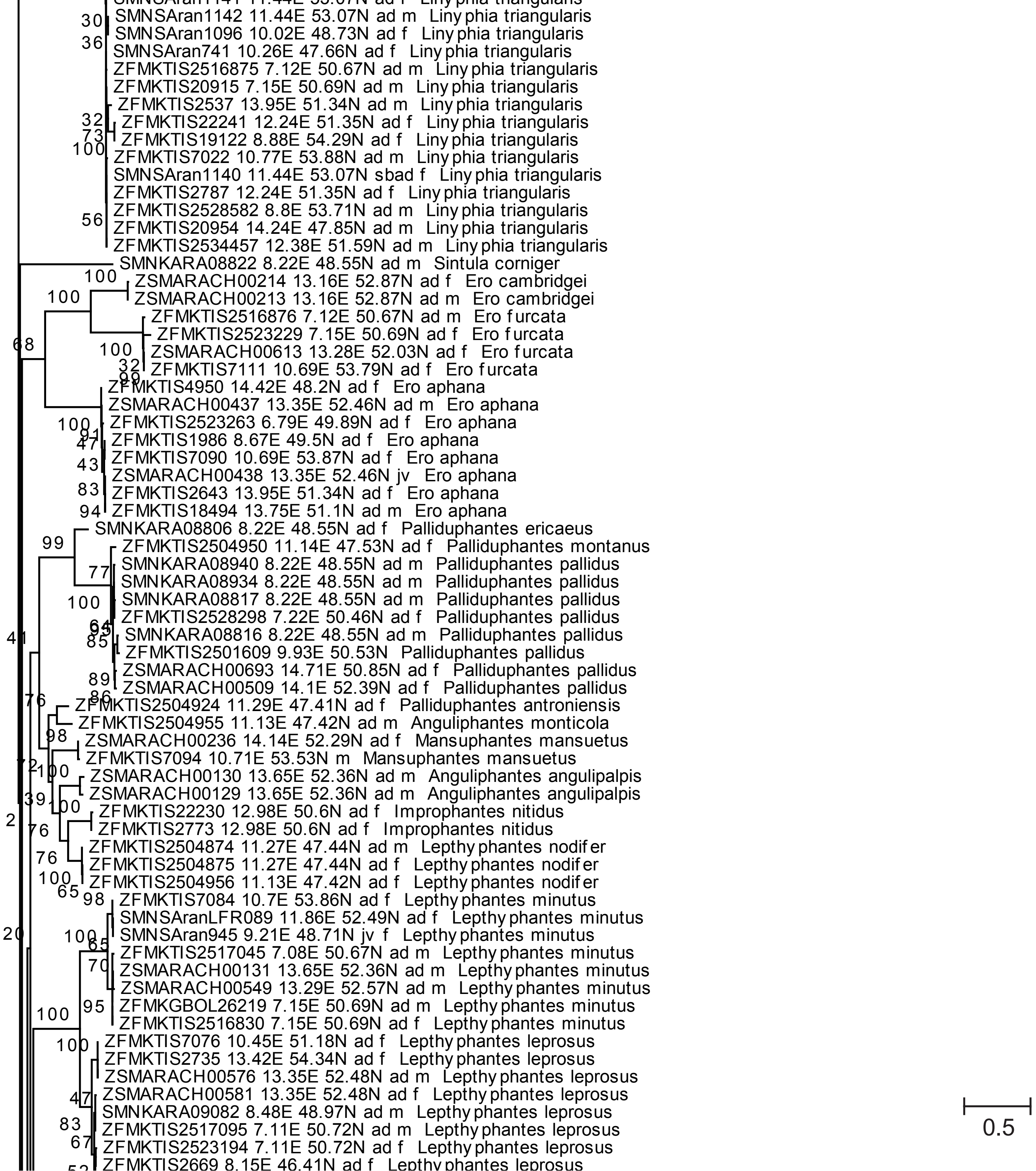
SMNSAran1142 11.44E 53.07N ad m Linyphia triangularis
SMNSAran1096 10.02E 48.73N ad f Linyphia triangularis
SMNSAran741 10.26E 47.66N ad f Linyphia triangularis
ZFMKTIS2516875 7.12E 50.67N ad m Linyphia triangularis
ZFMKTIS20915 7.15E 50.69N ad m Linyphia triangularis
ZFMKTIS2537 13.95E 51.34N ad m Linyphia triangularis
ZFMKTIS22241 12.24E 51.35N ad f Linyphia triangularis
ZFMKTIS19122 8.88E 54.29N ad f Linyphia triangularis
ZFMKTIS7022 10.77E 53.88N ad m Linyphia triangularis
SMNSAran1140 11.44E 53.07N sbad f Linyphia triangularis
ZFMKTIS2787 12.24E 51.35N ad f Linyphia triangularis
ZFMKTIS2528582 8.8E 53.71N ad m Linyphia triangularis
ZFMKTIS20954 14.24E 47.85N ad m Linyphia triangularis
ZFMKTIS2534457 12.38E 51.59N ad m Linyphia triangularis
SMNKARA08822 8.22E 48.55N ad m Sintula corniger
ZSMARACH00214 13.16E 52.87N ad f Ero cambridgei
ZSMARACH00213 13.16E 52.87N ad m Ero cambridgei
ZFMKTIS2516876 7.12E 50.67N ad m Ero furcata
ZFMKTIS2523229 7.15E 50.69N ad f Ero furcata
ZSMARACH00613 13.28E 52.03N ad f Ero furcata
ZFMKTIS7111 10.69E 53.79N ad f Ero furcata
ZFMKTIS4950 14.42E 48.2N ad f Ero aphana
ZSMARACH00437 13.35E 52.46N ad m Ero aphana
ZFMKTIS2523263 6.79E 49.89N ad f Ero aphana
ZFMKTIS1986 8.67E 49.5N ad f Ero aphana
ZFMKTIS7090 10.69E 53.87N ad f Ero aphana
ZSMARACH00438 13.35E 52.46N jv Ero aphana
ZFMKTIS2643 13.95E 51.34N ad f Ero aphana
ZFMKTIS18494 13.75E 51.1N ad m Ero aphana
SMNKARA08806 8.22E 48.55N ad f Palliduphantes ericaeus
ZFMKTIS2504950 11.14E 47.53N ad f Palliduphantes montanus
SMNKARA08940 8.22E 48.55N ad m Palliduphantes pallidus
SMNKARA08934 8.22E 48.55N ad m Palliduphantes pallidus
SMNKARA08817 8.22E 48.55N ad m Palliduphantes pallidus
ZFMKTIS2528298 7.22E 50.46N ad f Palliduphantes pallidus
SMNKARA08816 8.22E 48.55N ad m Palliduphantes pallidus
ZFMKTIS2501609 9.93E 50.53N Palliduphantes pallidus
ZSMARACH00693 14.71E 50.85N ad f Palliduphantes pallidus
ZSMARACH00509 14.1E 52.39N ad f Palliduphantes pallidus
ZFMKTIS2504924 11.29E 47.41N ad f Palliduphantes antroniensis
ZFMKTIS2504955 11.13E 47.42N ad m Anguliphantes monticola
ZSMARACH00236 14.14E 52.29N ad f Mansuphantes mansuetus
ZFMKTIS7094 10.71E 53.53N m Mansuphantes mansuetus
ZSMARACH00130 13.65E 52.36N ad m Anguliphantes angulipalpis
ZSMARACH00129 13.65E 52.36N ad m Anguliphantes angulipalpis
ZFMKTIS22230 12.98E 50.6N ad f Improphantes nitidus
ZFMKTIS2773 12.98E 50.6N ad f Improphantes nitidus
ZFMKTIS2504874 11.27E 47.44N ad m Lepthyphantes nodifer
ZFMKTIS2504875 11.27E 47.44N ad f Lepthyphantes nodifer
ZFMKTIS2504956 11.13E 47.42N ad f Lepthyphantes nodifer
ZFMKTIS7084 10.7E 53.86N ad f Lepthyphantes minutus
SMNSAranLFR089 11.86E 52.49N ad f Lepthyphantes minutus
SMNSAran945 9.21E 48.71N jv f Lepthyphantes minutus
ZFMKTIS2517045 7.08E 50.67N ad m Lepthyphantes minutus
ZSMARACH00131 13.65E 52.36N ad m Lepthyphantes minutus
ZSMARACH00549 13.29E 52.57N ad m Lepthyphantes minutus
ZFMKGBOL26219 7.15E 50.69N ad m Lepthyphantes minutus
ZFMKTIS2516830 7.15E 50.69N ad f Lepthyphantes minutus
ZFMKTIS7076 10.45E 51.18N ad f Lepthyphantes leprosus
ZFMKTIS2735 13.42E 54.34N ad f Lepthyphantes leprosus
ZSMARACH00576 13.35E 52.48N ad m Lepthyphantes leprosus
ZSMARACH00581 13.35E 52.48N ad f Lepthyphantes leprosus
SMNKARA09082 8.48E 48.97N ad m Lepthyphantes leprosus
ZFMKTIS2517095 7.11E 50.72N ad m Lepthyphantes leprosus
ZFMKTIS2523194 7.11E 50.72N ad f Lepthyphantes leprosus
ZFMKTIS2669 8.15E 46.41N ad f Lepthyphantes leprosus
0.5

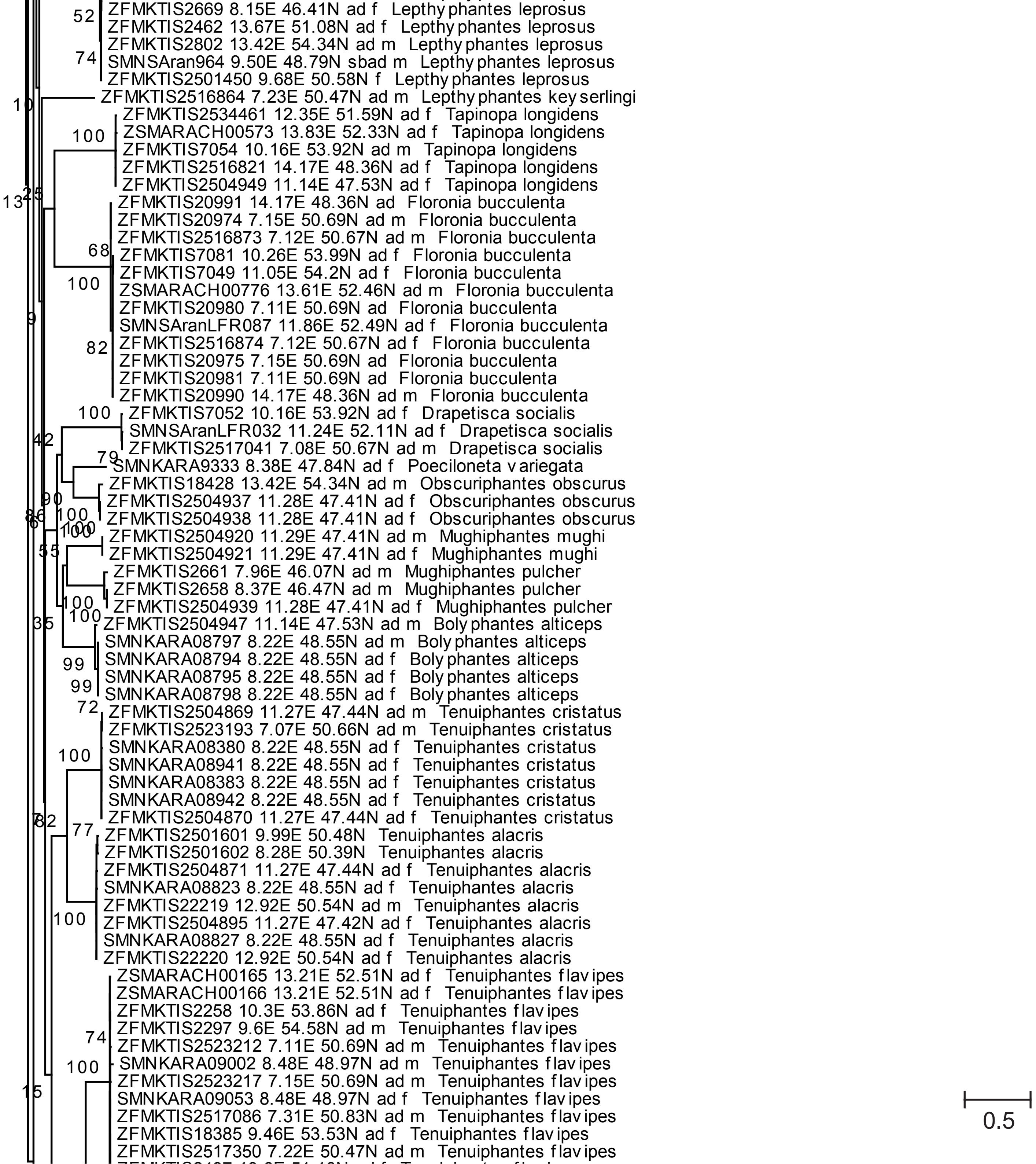
ZFMKTIS2669 8.15E 46.41N ad f Lepthyphantes leprosus
ZFMKTIS2462 13.67E 51.08N ad f Lepthyphantes leprosus
ZFMKTIS2802 13.42E 54.34N ad m Lepthyphantes leprosus
SMNSAran964 9.50E 48.79N sbad m Lepthyphantes leprosus
ZFMKTIS2501450 9.68E 50.58N f Lepthyphantes leprosus
ZFMKTIS2516864 7.23E 50.47N ad m Lepthyphantes keyserlingi
ZFMKTIS2534461 12.35E 51.59N ad f Tapinopa longidens
ZSMARACH00573 13.83E 52.33N ad f Tapinopa longidens
ZFMKTIS7054 10.16E 53.92N ad m Tapinopa longidens
ZFMKTIS2516821 14.17E 48.36N ad f Tapinopa longidens
ZFMKTIS2504949 11.14E 47.53N ad f Tapinopa longidens
ZFMKTIS20991 14.17E 48.36N ad Floronia bucculenta
ZFMKTIS20974 7.15E 50.69N ad m Floronia bucculenta
ZFMKTIS2516873 7.12E 50.67N ad m Floronia bucculenta
ZFMKTIS7081 10.26E 53.99N ad f Floronia bucculenta
ZFMKTIS7049 11.05E 54.2N ad f Floronia bucculenta
ZSMARACH00776 13.61E 52.46N ad m Floronia bucculenta
ZFMKTIS20980 7.11E 50.69N ad Floronia bucculenta
SMNSAranLFR087 11.86E 52.49N ad f Floronia bucculenta
ZFMKTIS2516874 7.12E 50.67N ad f Floronia bucculenta
ZFMKTIS20975 7.15E 50.69N ad Floronia bucculenta
ZFMKTIS20981 7.11E 50.69N ad Floronia bucculenta
ZFMKTIS20990 14.17E 48.36N ad m Floronia bucculenta
ZFMKTIS7052 10.16E 53.92N ad f Drapetisca socialis
SMNSAranLFR032 11.24E 52.11N ad f Drapetisca socialis
ZFMKTIS2517041 7.08E 50.67N ad m Drapetisca socialis
SMNKARA9333 8.38E 47.84N ad f Poeciloneta variegata
ZFMKTIS18428 13.42E 54.34N ad m Obscuriphantes obscurus
ZFMKTIS2504937 11.28E 47.41N ad f Obscuriphantes obscurus
ZFMKTIS2504938 11.28E 47.41N ad f Obscuriphantes obscurus
ZFMKTIS2504920 11.29E 47.41N ad m Mughiphantes mughi
ZFMKTIS2504921 11.29E 47.41N ad f Mughiphantes mughi
ZFMKTIS2661 7.96E 46.07N ad m Mughiphantes pulcher
ZFMKTIS2658 8.37E 46.47N ad m Mughiphantes pulcher
ZFMKTIS2504939 11.28E 47.41N ad f Mughiphantes pulcher
ZFMKTIS2504947 11.14E 47.53N ad m Bolyphantes alticeps
SMNKARA08797 8.22E 48.55N ad m Bolyphantes alticeps
SMNKARA08794 8.22E 48.55N ad f Bolyphantes alticeps
SMNKARA08795 8.22E 48.55N ad f Bolyphantes alticeps
SMNKARA08798 8.22E 48.55N ad f Bolyphantes alticeps
ZFMKTIS2504869 11.27E 47.44N ad m Tenuiphantes cristatus
ZFMKTIS2523193 7.07E 50.66N ad m Tenuiphantes cristatus
SMNKARA08380 8.22E 48.55N ad f Tenuiphantes cristatus
SMNKARA08941 8.22E 48.55N ad f Tenuiphantes cristatus
SMNKARA08383 8.22E 48.55N ad f Tenuiphantes cristatus
SMNKARA08942 8.22E 48.55N ad f Tenuiphantes cristatus
ZFMKTIS2504870 11.27E 47.44N ad f Tenuiphantes cristatus
ZFMKTIS2501601 9.99E 50.48N Tenuiphantes alacris
ZFMKTIS2501602 8.28E 50.39N Tenuiphantes alacris
ZFMKTIS2504871 11.27E 47.44N ad f Tenuiphantes alacris
SMNKARA08823 8.22E 48.55N ad f Tenuiphantes alacris
ZFMKTIS22219 12.92E 50.54N ad m Tenuiphantes alacris
ZFMKTIS2504895 11.27E 47.42N ad f Tenuiphantes alacris
SMNKARA08827 8.22E 48.55N ad f Tenuiphantes alacris
ZFMKTIS22220 12.92E 50.54N ad f Tenuiphantes alacris
ZSMARACH00165 13.21E 52.51N ad f Tenuiphantes flavipes
ZSMARACH00166 13.21E 52.51N ad f Tenuiphantes flavipes
ZFMKTIS2258 10.3E 53.86N ad f Tenuiphantes flavipes
ZFMKTIS2297 9.6E 54.58N ad m Tenuiphantes flavipes
ZFMKTIS2523212 7.11E 50.69N ad m Tenuiphantes flavipes
SMNKARA09002 8.48E 48.97N ad m Tenuiphantes flavipes
ZFMKTIS2523217 7.15E 50.69N ad m Tenuiphantes flavipes
SMNKARA09053 8.48E 48.97N ad f Tenuiphantes flavipes
ZFMKTIS2517086 7.31E 50.83N ad m Tenuiphantes flavipes
ZFMKTIS18385 9.46E 53.53N ad f Tenuiphantes flavipes
ZFMKTIS2517350 7.22E 50.47N ad m Tenuiphantes flavipes
0.5

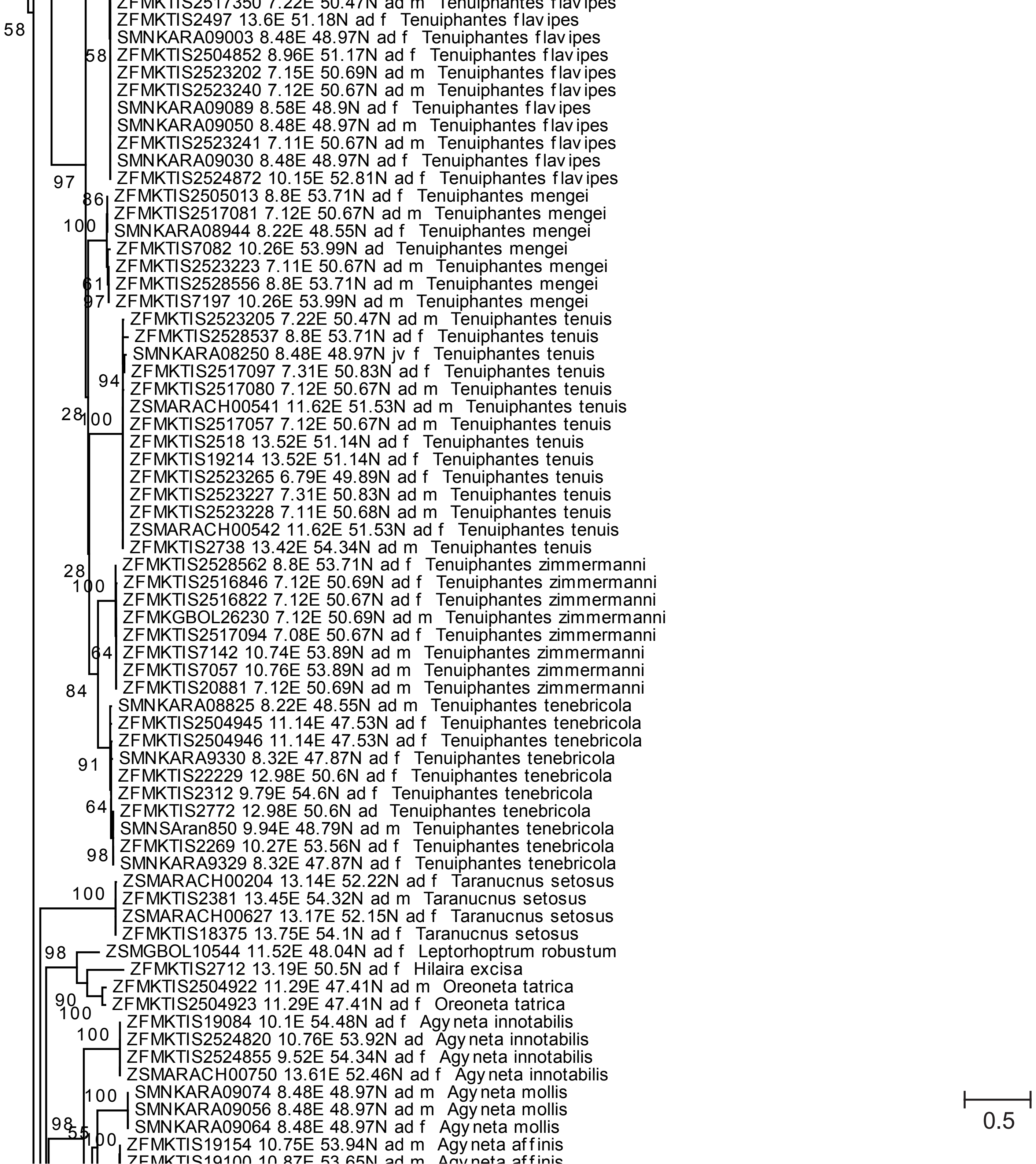

ZFMKTIS2517350 7.22E 50.47N ad m Tenuiphantes flavipes
58
ZFMKTIS2497 13.6E 51.18N ad f Tenuiphantes flavipes
SMNKARA09003 8.48E 48.97N ad f Tenuiphantes flavipes
58
ZFMKTIS2504852 8.96E 51.17N ad f Tenuiphantes flavipes
ZFMKTIS2523202 7.15E 50.69N ad m Tenuiphantes flavipes
ZFMKTIS2523240 7.12E 50.67N ad m Tenuiphantes flavipes
SMNKARA09089 8.58E 48.9N ad f Tenuiphantes flavipes
SMNKARA09050 8.48E 48.97N ad m Tenuiphantes flavipes
ZFMKTIS2523241 7.11E 50.67N ad m Tenuiphantes flavipes
SMNKARA09030 8.48E 48.97N ad f Tenuiphantes flavipes
ZFMKTIS2524872 10.15E 52.81N ad f Tenuiphantes flavipes
97
86
ZFMKTIS2505013 8.8E 53.71N ad f Tenuiphantes mengei
ZFMKTIS2517081 7.12E 50.67N ad m Tenuiphantes mengei
100
SMNKARA08944 8.22E 48.55N ad f Tenuiphantes mengei
ZFMKTIS7082 10.26E 53.99N ad Tenuiphantes mengei
ZFMKTIS2523223 7.11E 50.67N ad m Tenuiphantes mengei
61
ZFMKTIS2528556 8.8E 53.71N ad m Tenuiphantes mengei
97
ZFMKTIS7197 10.26E 53.99N ad m Tenuiphantes mengei
ZFMKTIS2523205 7.22E 50.47N ad m Tenuiphantes tenuis
ZFMKTIS2528537 8.8E 53.71N ad f Tenuiphantes tenuis
SMNKARA08250 8.48E 48.97N jv f Tenuiphantes tenuis
ZFMKTIS2517097 7.31E 50.83N ad f Tenuiphantes tenuis
94
ZFMKTIS2517080 7.12E 50.67N ad m Tenuiphantes tenuis
ZSMARACH00541 11.62E 51.53N ad m Tenuiphantes tenuis
28
100
ZFMKTIS2517057 7.12E 50.67N ad m Tenuiphantes tenuis
ZFMKTIS2518 13.52E 51.14N ad f Tenuiphantes tenuis
ZFMKTIS19214 13.52E 51.14N ad f Tenuiphantes tenuis
ZFMKTIS2523265 6.79E 49.89N ad f Tenuiphantes tenuis
ZFMKTIS2523227 7.31E 50.83N ad m Tenuiphantes tenuis
ZFMKTIS2523228 7.11E 50.68N ad m Tenuiphantes tenuis
ZSMARACH00542 11.62E 51.53N ad f Tenuiphantes tenuis
ZFMKTIS2738 13.42E 54.34N ad m Tenuiphantes tenuis
ZFMKTIS2528562 8.8E 53.71N ad f Tenuiphantes zimmermanni
28
100
ZFMKTIS2516846 7.12E 50.69N ad f Tenuiphantes zimmermanni
ZFMKTIS2516822 7.12E 50.67N ad f Tenuiphantes zimmermanni
ZFMKGBOL26230 7.12E 50.69N ad m Tenuiphantes zimmermanni
ZFMKTIS2517094 7.08E 50.67N ad f Tenuiphantes zimmermanni
64
ZFMKTIS7142 10.74E 53.89N ad m Tenuiphantes zimmermanni
ZFMKTIS7057 10.76E 53.89N ad m Tenuiphantes zimmermanni
ZFMKTIS20881 7.12E 50.69N ad m Tenuiphantes zimmermanni
84
SMNKARA08825 8.22E 48.55N ad m Tenuiphantes tenebricola
ZFMKTIS2504945 11.14E 47.53N ad f Tenuiphantes tenebricola
ZFMKTIS2504946 11.14E 47.53N ad f Tenuiphantes tenebricola
SMNKARA9330 8.32E 47.87N ad f Tenuiphantes tenebricola
91
ZFMKTIS22229 12.98E 50.6N ad f Tenuiphantes tenebricola
ZFMKTIS2312 9.79E 54.6N ad f Tenuiphantes tenebricola
64
ZFMKTIS2772 12.98E 50.6N ad Tenuiphantes tenebricola
SMNSAran850 9.94E 48.79N ad m Tenuiphantes tenebricola
ZFMKTIS2269 10.27E 53.56N ad f Tenuiphantes tenebricola
98
SMNKARA9329 8.32E 47.87N ad f Tenuiphantes tenebricola
ZSMARACH00204 13.14E 52.22N ad f Taranucnus setosus
100
ZFMKTIS2381 13.45E 54.32N ad m Taranucnus setosus
ZSMARACH00627 13.17E 52.15N ad f Taranucnus setosus
ZFMKTIS18375 13.75E 54.1N ad f Taranucnus setosus
98
ZSMGBOL10544 11.52E 48.04N ad f Leptorhoptrum robustum
ZFMKTIS2712 13.19E 50.5N ad f Hilaira excisa
ZFMKTIS2504922 11.29E 47.41N ad m Oreoneta tatrica
90
ZFMKTIS2504923 11.29E 47.41N ad f Oreoneta tatrica
100
ZFMKTIS19084 10.1E 54.48N ad f Agyneta innotabilis
100
ZFMKTIS2524820 10.76E 53.92N ad Agyneta innotabilis
ZFMKTIS2524855 9.52E 54.34N ad f Agyneta innotabilis
ZSMARACH00750 13.61E 52.46N ad f Agyneta innotabilis
100
SMNKARA09074 8.48E 48.97N ad m Agyneta mollis
SMNKARA09056 8.48E 48.97N ad m Agyneta mollis
SMNKARA09064 8.48E 48.97N ad f Agyneta mollis
98
55
100
ZFMKTIS19154 10.75E 53.94N ad m Agyneta affinis
ZFMKTIS19100 10.87E 53.65N ad m Agyneta affinis
0.5

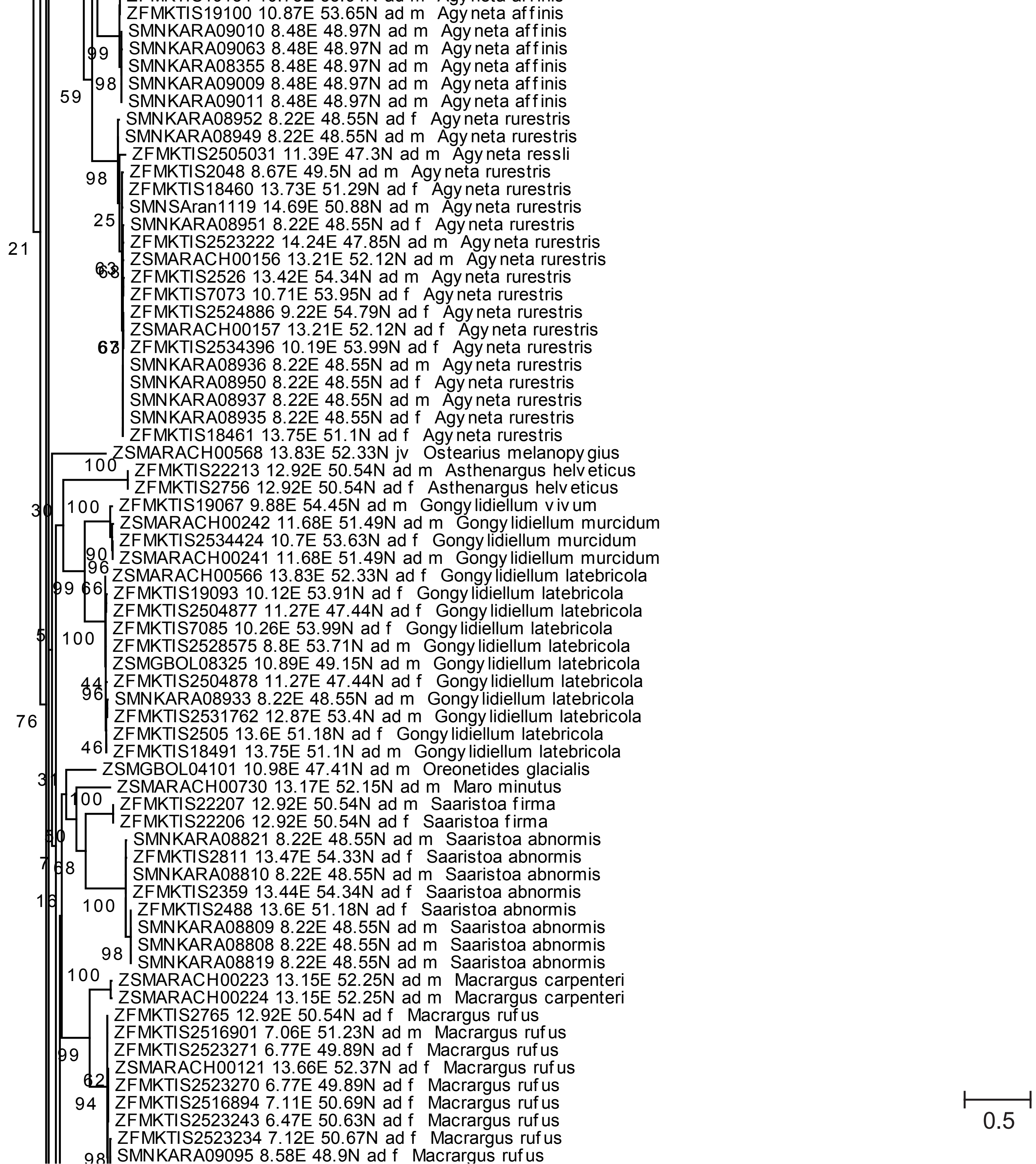
ZFMKTIS19100 10.87E 53.65N ad m Agyneta affinis
SMNKARA09010 8.48E 48.97N ad m Agyneta affinis
SMNKARA09063 8.48E 48.97N ad m Agyneta affinis
SMNKARA08355 8.48E 48.97N ad m Agyneta affinis
SMNKARA09009 8.48E 48.97N ad m Agyneta affinis
SMNKARA09011 8.48E 48.97N ad m Agyneta affinis
SMNKARA08952 8.22E 48.55N ad f Agyneta rurestris
SMNKARA08949 8.22E 48.55N ad m Agyneta rurestris
ZFMKTIS2505031 11.39E 47.3N ad m Agyneta ressli
ZFMKTIS2048 8.67E 49.5N ad m Agyneta rurestris
ZFMKTIS18460 13.73E 51.29N ad f Agyneta rurestris
SMNSAran1119 14.69E 50.88N ad m Agyneta rurestris
SMNKARA08951 8.22E 48.55N ad f Agyneta rurestris
ZFMKTIS2523222 14.24E 47.85N ad m Agyneta rurestris
ZSMARACH00156 13.21E 52.12N ad m Agyneta rurestris
ZFMKTIS2526 13.42E 54.34N ad m Agyneta rurestris
ZFMKTIS7073 10.71E 53.95N ad f Agyneta rurestris
ZFMKTIS2524886 9.22E 54.79N ad f Agyneta rurestris
ZSMARACH00157 13.21E 52.12N ad f Agyneta rurestris
ZFMKTIS2534396 10.19E 53.99N ad f Agyneta rurestris
SMNKARA08936 8.22E 48.55N ad m Agyneta rurestris
SMNKARA08950 8.22E 48.55N ad f Agyneta rurestris
SMNKARA08937 8.22E 48.55N ad m Agyneta rurestris
SMNKARA08935 8.22E 48.55N ad f Agyneta rurestris
ZFMKTIS18461 13.75E 51.1N ad f Agyneta rurestris
ZSMARACH00568 13.83E 52.33N jv Ostearius melanopygius
ZFMKTIS22213 12.92E 50.54N ad m Asthenargus helveticus
ZFMKTIS2756 12.92E 50.54N ad f Asthenargus helveticus
ZFMKTIS19067 9.88E 54.45N ad m Gongylidiellum vivum
ZSMARACH00242 11.68E 51.49N ad m Gongylidiellum murcidum
ZFMKTIS2534424 10.7E 53.63N ad f Gongylidiellum murcidum
ZSMARACH00241 11.68E 51.49N ad m Gongylidiellum murcidum
ZSMARACH00566 13.83E 52.33N ad f Gongylidiellum latebricola
ZFMKTIS19093 10.12E 53.91N ad f Gongylidiellum latebricola
ZFMKTIS2504877 11.27E 47.44N ad f Gongylidiellum latebricola
ZFMKTIS7085 10.26E 53.99N ad f Gongylidiellum latebricola
ZFMKTIS2528575 8.8E 53.71N ad m Gongylidiellum latebricola
ZSMGBOL08325 10.89E 49.15N ad m Gongylidiellum latebricola
ZFMKTIS2504878 11.27E 47.44N ad f Gongylidiellum latebricola
SMNKARA08933 8.22E 48.55N ad m Gongylidiellum latebricola
ZFMKTIS2531762 12.87E 53.4N ad m Gongylidiellum latebricola
ZFMKTIS2505 13.6E 51.18N ad f Gongylidiellum latebricola
ZFMKTIS18491 13.75E 51.1N ad m Gongylidiellum latebricola
ZSMGBOL04101 10.98E 47.41N ad m Oreonetides glacialis
ZSMARACH00730 13.17E 52.15N ad m Maro minutus
ZFMKTIS22207 12.92E 50.54N ad m Saaristoa firma
ZFMKTIS22206 12.92E 50.54N ad f Saaristoa firma
SMNKARA08821 8.22E 48.55N ad m Saaristoa abnormis
ZFMKTIS2811 13.47E 54.33N ad f Saaristoa abnormis
SMNKARA08810 8.22E 48.55N ad m Saaristoa abnormis
ZFMKTIS2359 13.44E 54.34N ad f Saaristoa abnormis
ZFMKTIS2488 13.6E 51.18N ad f Saaristoa abnormis
SMNKARA08809 8.22E 48.55N ad m Saaristoa abnormis
SMNKARA08808 8.22E 48.55N ad m Saaristoa abnormis
SMNKARA08819 8.22E 48.55N ad m Saaristoa abnormis
ZSMARACH00223 13.15E 52.25N ad m Macrargus carpenteri
ZSMARACH00224 13.15E 52.25N ad m Macrargus carpenteri
ZFMKTIS2765 12.92E 50.54N ad f Macrargus rufus
ZFMKTIS2516901 7.06E 51.23N ad m Macrargus rufus
ZFMKTIS2523271 6.77E 49.89N ad f Macrargus rufus
ZSMARACH00121 13.66E 52.37N ad f Macrargus rufus
ZFMKTIS2523270 6.77E 49.89N ad f Macrargus rufus
ZFMKTIS2516894 7.11E 50.69N ad f Macrargus rufus
ZFMKTIS2523243 6.47E 50.63N ad f Macrargus rufus
ZFMKTIS2523234 7.12E 50.67N ad f Macrargus rufus
SMNKARA09095 8.58E 48.9N ad f Macrargus rufus
0.5

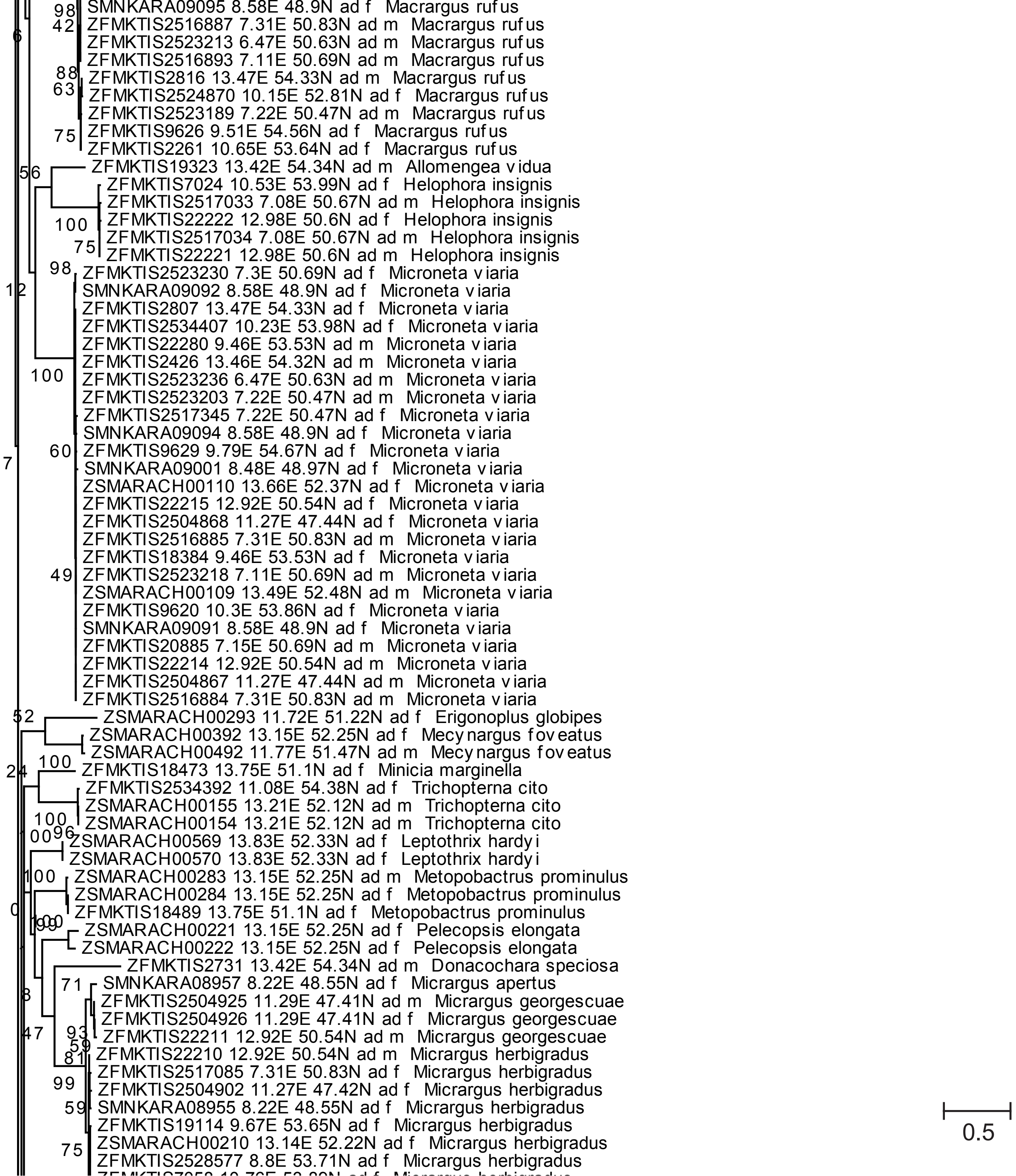

SMNKARA09095 8.58E 48.9N ad f Macrargus rufus
ZFMKTIS2516887 7.31E 50.83N ad m Macrargus rufus
ZFMKTIS2523213 6.47E 50.63N ad m Macrargus rufus
ZFMKTIS2516893 7.11E 50.69N ad m Macrargus rufus
ZFMKTIS2816 13.47E 54.33N ad m Macrargus rufus
ZFMKTIS2524870 10.15E 52.81N ad f Macrargus rufus
ZFMKTIS2523189 7.22E 50.47N ad m Macrargus rufus
ZFMKTIS9626 9.51E 54.56N ad f Macrargus rufus
ZFMKTIS2261 10.65E 53.64N ad f Macrargus rufus
ZFMKTIS19323 13.42E 54.34N ad m Allomengea vidua
ZFMKTIS7024 10.53E 53.99N ad f Helophora insignis
ZFMKTIS2517033 7.08E 50.67N ad m Helophora insignis
ZFMKTIS22222 12.98E 50.6N ad f Helophora insignis
ZFMKTIS2517034 7.08E 50.67N ad m Helophora insignis
ZFMKTIS22221 12.98E 50.6N ad m Helophora insignis
ZFMKTIS2523230 7.3E 50.69N ad f Microneta viaria
SMNKARA09092 8.58E 48.9N ad f Microneta viaria
ZFMKTIS2807 13.47E 54.33N ad f Microneta viaria
ZFMKTIS2534407 10.23E 53.98N ad f Microneta viaria
ZFMKTIS22280 9.46E 53.53N ad m Microneta viaria
ZFMKTIS2426 13.46E 54.32N ad m Microneta viaria
ZFMKTIS2523236 6.47E 50.63N ad m Microneta viaria
ZFMKTIS2523203 7.22E 50.47N ad m Microneta viaria
ZFMKTIS2517345 7.22E 50.47N ad f Microneta viaria
SMNKARA09094 8.58E 48.9N ad f Microneta viaria
ZFMKTIS9629 9.79E 54.67N ad f Microneta viaria
SMNKARA09001 8.48E 48.97N ad f Microneta viaria
ZSMARACH00110 13.66E 52.37N ad f Microneta viaria
ZFMKTIS22215 12.92E 50.54N ad f Microneta viaria
ZFMKTIS2504868 11.27E 47.44N ad f Microneta viaria
ZFMKTIS2516885 7.31E 50.83N ad m Microneta viaria
ZFMKTIS18384 9.46E 53.53N ad f Microneta viaria
ZFMKTIS2523218 7.11E 50.69N ad m Microneta viaria
ZSMARACH00109 13.49E 52.48N ad m Microneta viaria
ZFMKTIS9620 10.3E 53.86N ad f Microneta viaria
SMNKARA09091 8.58E 48.9N ad f Microneta viaria
ZFMKTIS20885 7.15E 50.69N ad m Microneta viaria
ZFMKTIS22214 12.92E 50.54N ad m Microneta viaria
ZFMKTIS2504867 11.27E 47.44N ad m Microneta viaria
ZFMKTIS2516884 7.31E 50.83N ad m Microneta viaria
ZSMARACH00293 11.72E 51.22N ad f Erigonoplus globipes
ZSMARACH00392 13.15E 52.25N ad f Mecynargus foveatus
ZSMARACH00492 11.77E 51.47N ad m Mecynargus foveatus
ZFMKTIS18473 13.75E 51.1N ad f Minicia marginella
ZFMKTIS2534392 11.08E 54.38N ad f Trichopterna cito
ZSMARACH00155 13.21E 52.12N ad m Trichopterna cito
ZSMARACH00154 13.21E 52.12N ad m Trichopterna cito
ZSMARACH00569 13.83E 52.33N ad f Leptothrix hardyi
ZSMARACH00570 13.83E 52.33N ad f Leptothrix hardyi
ZSMARACH00283 13.15E 52.25N ad m Metopobactrus prominulus
ZSMARACH00284 13.15E 52.25N ad f Metopobactrus prominulus
ZFMKTIS18489 13.75E 51.1N ad f Metopobactrus prominulus
ZSMARACH00221 13.15E 52.25N ad f Pelecopsis elongata
ZSMARACH00222 13.15E 52.25N ad f Pelecopsis elongata
ZFMKTIS2731 13.42E 54.34N ad m Donacochara speciosa
SMNKARA08957 8.22E 48.55N ad f Micrargus apertus
ZFMKTIS2504925 11.29E 47.41N ad m Micrargus georgescuae
ZFMKTIS2504926 11.29E 47.41N ad f Micrargus georgescuae
ZFMKTIS22211 12.92E 50.54N ad m Micrargus georgescuae
ZFMKTIS22210 12.92E 50.54N ad m Micrargus herbigradus
ZFMKTIS2517085 7.31E 50.83N ad f Micrargus herbigradus
ZFMKTIS2504902 11.27E 47.42N ad f Micrargus herbigradus
SMNKARA08955 8.22E 48.55N ad f Micrargus herbigradus
ZFMKTIS19114 9.67E 53.65N ad f Micrargus herbigradus
ZSMARACH00210 13.14E 52.22N ad f Micrargus herbigradus
ZFMKTIS2528577 8.8E 53.71N ad f Micrargus herbigradus
0.5

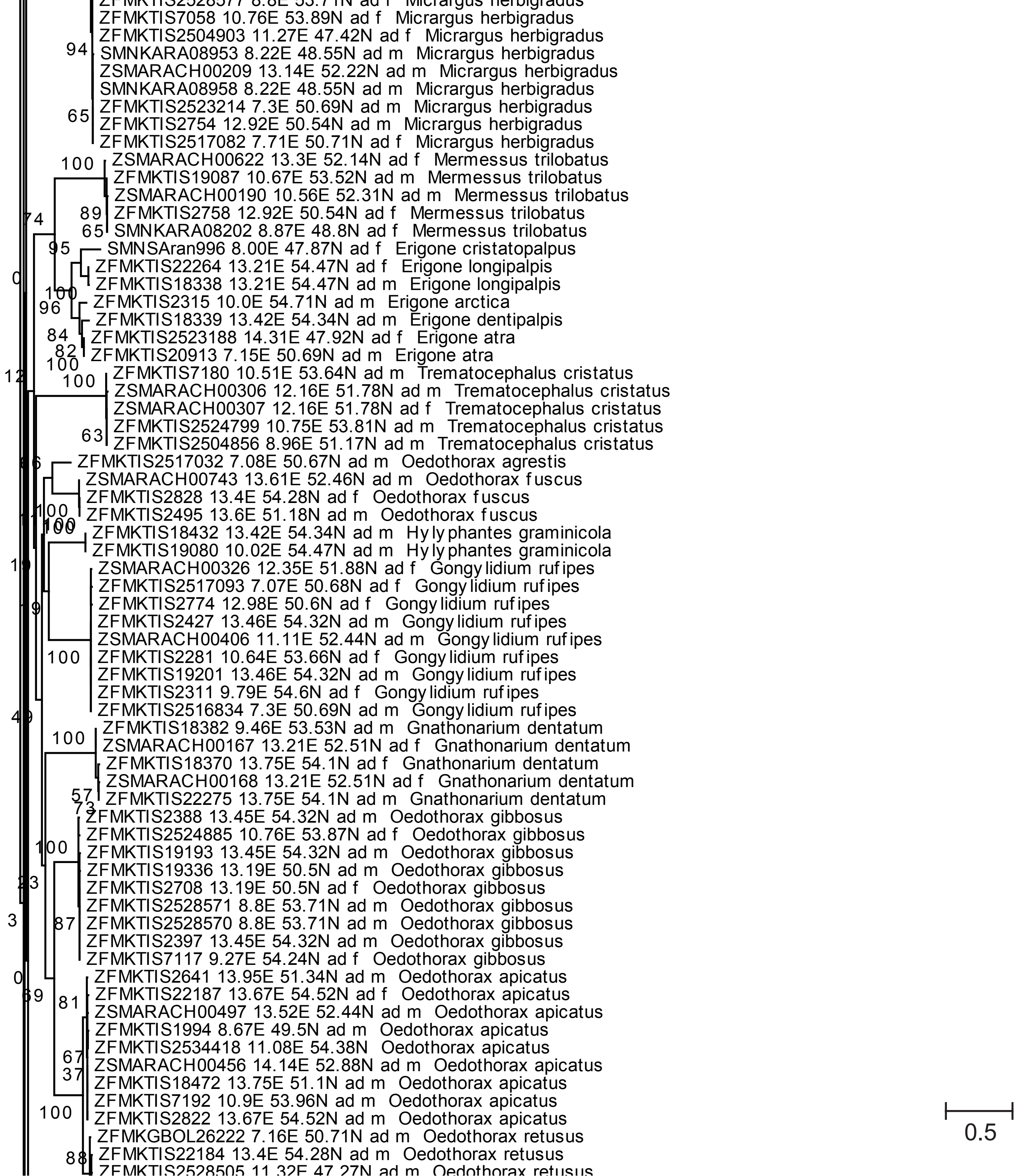
ZFMKTIS7058 10.76E 53.89N ad f Micrargus herbigradus
ZFMKTIS2504903 11.27E 47.42N ad f Micrargus herbigradus
SMNKARA08953 8.22E 48.55N ad m Micrargus herbigradus
ZSMARACH00209 13.14E 52.22N ad m Micrargus herbigradus
SMNKARA08958 8.22E 48.55N ad m Micrargus herbigradus
ZFMKTIS2523214 7.3E 50.69N ad m Micrargus herbigradus
ZFMKTIS2754 12.92E 50.54N ad m Micrargus herbigradus
ZFMKTIS2517082 7.71E 50.71N ad f Micrargus herbigradus
ZSMARACH00622 13.3E 52.14N ad f Mermessus trilobatus
ZFMKTIS19087 10.67E 53.52N ad m Mermessus trilobatus
ZSMARACH00190 10.56E 52.31N ad m Mermessus trilobatus
ZFMKTIS2758 12.92E 50.54N ad f Mermessus trilobatus
SMNKARA08202 8.87E 48.8N ad f Mermessus trilobatus
SMNSAran996 8.00E 47.87N ad f Erigone cristatopalpus
ZFMKTIS22264 13.21E 54.47N ad f Erigone longipalpis
ZFMKTIS18338 13.21E 54.47N ad m Erigone longipalpis
ZFMKTIS2315 10.0E 54.71N ad m Erigone arctica
ZFMKTIS18339 13.42E 54.34N ad m Erigone dentipalpis
ZFMKTIS2523188 14.31E 47.92N ad f Erigone atra
ZFMKTIS20913 7.15E 50.69N ad m Erigone atra
ZFMKTIS7180 10.51E 53.64N ad m Trematocephalus cristatus
ZSMARACH00306 12.16E 51.78N ad m Trematocephalus cristatus
ZSMARACH00307 12.16E 51.78N ad f Trematocephalus cristatus
ZFMKTIS2524799 10.75E 53.81N ad m Trematocephalus cristatus
ZFMKTIS2504856 8.96E 51.17N ad m Trematocephalus cristatus
ZFMKTIS2517032 7.08E 50.67N ad m Oedothorax agrestis
ZSMARACH00743 13.61E 52.46N ad m Oedothorax fuscus
ZFMKTIS2828 13.4E 54.28N ad f Oedothorax fuscus
ZFMKTIS2495 13.6E 51.18N ad m Oedothorax fuscus
ZFMKTIS18432 13.42E 54.34N ad m Hylyphantes graminicola
ZFMKTIS19080 10.02E 54.47N ad m Hylyphantes graminicola
ZSMARACH00326 12.35E 51.88N ad f Gongylidium rufipes
ZFMKTIS2517093 7.07E 50.68N ad f Gongylidium rufipes
ZFMKTIS2774 12.98E 50.6N ad f Gongylidium rufipes
ZFMKTIS2427 13.46E 54.32N ad m Gongylidium rufipes
ZSMARACH00406 11.11E 52.44N ad m Gongylidium rufipes
ZFMKTIS2281 10.64E 53.66N ad f Gongylidium rufipes
ZFMKTIS19201 13.46E 54.32N ad m Gongylidium rufipes
ZFMKTIS2311 9.79E 54.6N ad f Gongylidium rufipes
ZFMKTIS2516834 7.3E 50.69N ad m Gongylidium rufipes
ZFMKTIS18382 9.46E 53.53N ad m Gnathonarium dentatum
ZSMARACH00167 13.21E 52.51N ad f Gnathonarium dentatum
ZFMKTIS18370 13.75E 54.1N ad f Gnathonarium dentatum
ZSMARACH00168 13.21E 52.51N ad f Gnathonarium dentatum
ZFMKTIS22275 13.75E 54.1N ad m Gnathonarium dentatum
ZFMKTIS2388 13.45E 54.32N ad m Oedothorax gibbosus
ZFMKTIS2524885 10.76E 53.87N ad f Oedothorax gibbosus
ZFMKTIS19193 13.45E 54.32N ad m Oedothorax gibbosus
ZFMKTIS19336 13.19E 50.5N ad m Oedothorax gibbosus
ZFMKTIS2708 13.19E 50.5N ad f Oedothorax gibbosus
ZFMKTIS2528571 8.8E 53.71N ad m Oedothorax gibbosus
ZFMKTIS2528570 8.8E 53.71N ad m Oedothorax gibbosus
ZFMKTIS2397 13.45E 54.32N ad m Oedothorax gibbosus
ZFMKTIS7117 9.27E 54.24N ad f Oedothorax gibbosus
ZFMKTIS2641 13.95E 51.34N ad m Oedothorax apicatus
ZFMKTIS22187 13.67E 54.52N ad f Oedothorax apicatus
ZSMARACH00497 13.52E 52.44N ad m Oedothorax apicatus
ZFMKTIS1994 8.67E 49.5N ad m Oedothorax apicatus
ZFMKTIS2534418 11.08E 54.38N Oedothorax apicatus
ZSMARACH00456 14.14E 52.88N ad m Oedothorax apicatus
ZFMKTIS18472 13.75E 51.1N ad m Oedothorax apicatus
ZFMKTIS7192 10.9E 53.96N ad m Oedothorax apicatus
ZFMKTIS2822 13.67E 54.52N ad m Oedothorax apicatus
ZFMKGBOL26222 7.16E 50.71N ad m Oedothorax retusus
ZFMKTIS22184 13.4E 54.28N ad m Oedothorax retusus
0.5

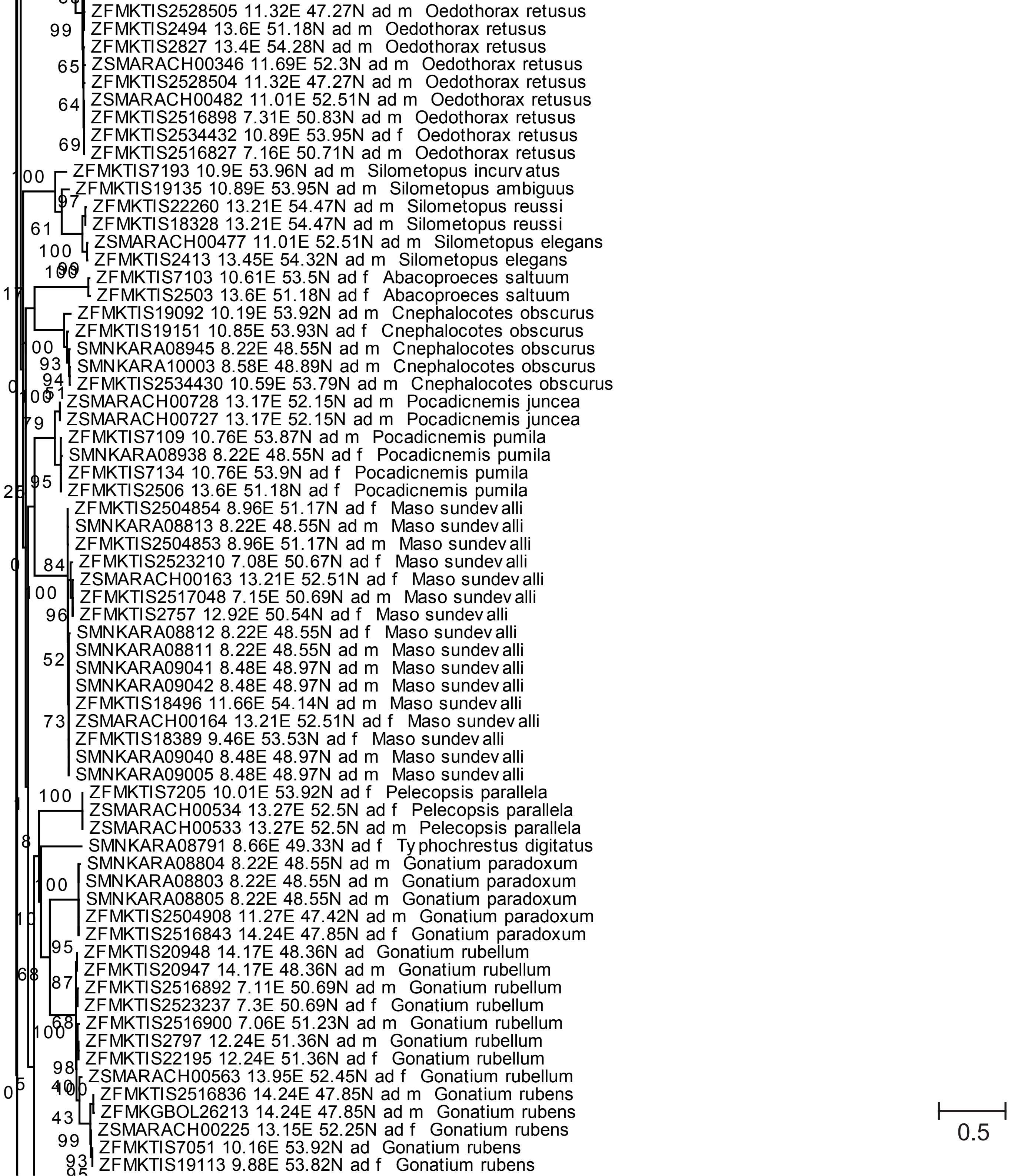
ZFMKTIS2528505 11.32E 47.27N ad m Oedothorax retusus
ZFMKTIS2494 13.6E 51.18N ad m Oedothorax retusus
ZFMKTIS2827 13.4E 54.28N ad m Oedothorax retusus
ZSMARACH00346 11.69E 52.3N ad m Oedothorax retusus
ZFMKTIS2528504 11.32E 47.27N ad m Oedothorax retusus
ZSMARACH00482 11.01E 52.51N ad m Oedothorax retusus
ZFMKTIS2516898 7.31E 50.83N ad m Oedothorax retusus
ZFMKTIS2534432 10.89E 53.95N ad f Oedothorax retusus
ZFMKTIS2516827 7.16E 50.71N ad m Oedothorax retusus
ZFMKTIS7193 10.9E 53.96N ad m Silometopus incurvatus
ZFMKTIS19135 10.89E 53.95N ad m Silometopus ambiguus
ZFMKTIS22260 13.21E 54.47N ad m Silometopus reussi
ZFMKTIS18328 13.21E 54.47N ad m Silometopus reussi
ZSMARACH00477 11.01E 52.51N ad m Silometopus elegans
ZFMKTIS2413 13.45E 54.32N ad m Silometopus elegans
ZFMKTIS7103 10.61E 53.5N ad f Abacoproeces saltuum
ZFMKTIS2503 13.6E 51.18N ad f Abacoproeces saltuum
ZFMKTIS19092 10.19E 53.92N ad m Cnephalocotes obscurus
ZFMKTIS19151 10.85E 53.93N ad f Cnephalocotes obscurus
SMNKARA08945 8.22E 48.55N ad m Cnephalocotes obscurus
SMNKARA10003 8.58E 48.89N ad m Cnephalocotes obscurus
ZFMKTIS2534430 10.59E 53.79N ad m Cnephalocotes obscurus
ZSMARACH00728 13.17E 52.15N ad m Pocadicnemis juncea
ZSMARACH00727 13.17E 52.15N ad m Pocadicnemis juncea
ZFMKTIS7109 10.76E 53.87N ad m Pocadicnemis pumila
SMNKARA08938 8.22E 48.55N ad f Pocadicnemis pumila
ZFMKTIS7134 10.76E 53.9N ad f Pocadicnemis pumila
ZFMKTIS2506 13.6E 51.18N ad f Pocadicnemis pumila
ZFMKTIS2504854 8.96E 51.17N ad f Maso sundevalli
SMNKARA08813 8.22E 48.55N ad m Maso sundevalli
ZFMKTIS2504853 8.96E 51.17N ad m Maso sundevalli
ZFMKTIS2523210 7.08E 50.67N ad f Maso sundevalli
ZSMARACH00163 13.21E 52.51N ad f Maso sundevalli
ZFMKTIS2517048 7.15E 50.69N ad m Maso sundevalli
ZFMKTIS2757 12.92E 50.54N ad f Maso sundevalli
SMNKARA08812 8.22E 48.55N ad f Maso sundevalli
SMNKARA08811 8.22E 48.55N ad m Maso sundevalli
SMNKARA09041 8.48E 48.97N ad m Maso sundevalli
SMNKARA09042 8.48E 48.97N ad m Maso sundevalli
ZFMKTIS18496 11.66E 54.14N ad m Maso sundevalli
ZSMARACH00164 13.21E 52.51N ad f Maso sundevalli
ZFMKTIS18389 9.46E 53.53N ad f Maso sundevalli
SMNKARA09040 8.48E 48.97N ad m Maso sundevalli
SMNKARA09005 8.48E 48.97N ad m Maso sundevalli
ZFMKTIS7205 10.01E 53.92N ad f Pelecopsis parallela
ZSMARACH00534 13.27E 52.5N ad f Pelecopsis parallela
ZSMARACH00533 13.27E 52.5N ad m Pelecopsis parallela
SMNKARA08791 8.66E 49.33N ad f Typhochrestus digitatus
SMNKARA08804 8.22E 48.55N ad m Gonatium paradoxum
SMNKARA08803 8.22E 48.55N ad m Gonatium paradoxum
SMNKARA08805 8.22E 48.55N ad m Gonatium paradoxum
ZFMKTIS2504908 11.27E 47.42N ad m Gonatium paradoxum
ZFMKTIS2516843 14.24E 47.85N ad f Gonatium paradoxum
ZFMKTIS20948 14.17E 48.36N ad Gonatium rubellum
ZFMKTIS20947 14.17E 48.36N ad m Gonatium rubellum
ZFMKTIS2516892 7.11E 50.69N ad m Gonatium rubellum
ZFMKTIS2523237 7.3E 50.69N ad f Gonatium rubellum
ZFMKTIS2516900 7.06E 51.23N ad m Gonatium rubellum
ZFMKTIS2797 12.24E 51.36N ad m Gonatium rubellum
ZFMKTIS22195 12.24E 51.36N ad f Gonatium rubellum
ZSMARACH00563 13.95E 52.45N ad f Gonatium rubellum
ZFMKTIS2516836 14.24E 47.85N ad m Gonatium rubens
ZFMKGBOL26213 14.24E 47.85N ad m Gonatium rubens
ZSMARACH00225 13.15E 52.25N ad f Gonatium rubens
ZFMKTIS7051 10.16E 53.92N ad Gonatium rubens
ZFMKTIS19113 9.88E 53.82N ad f Gonatium rubens
0.5

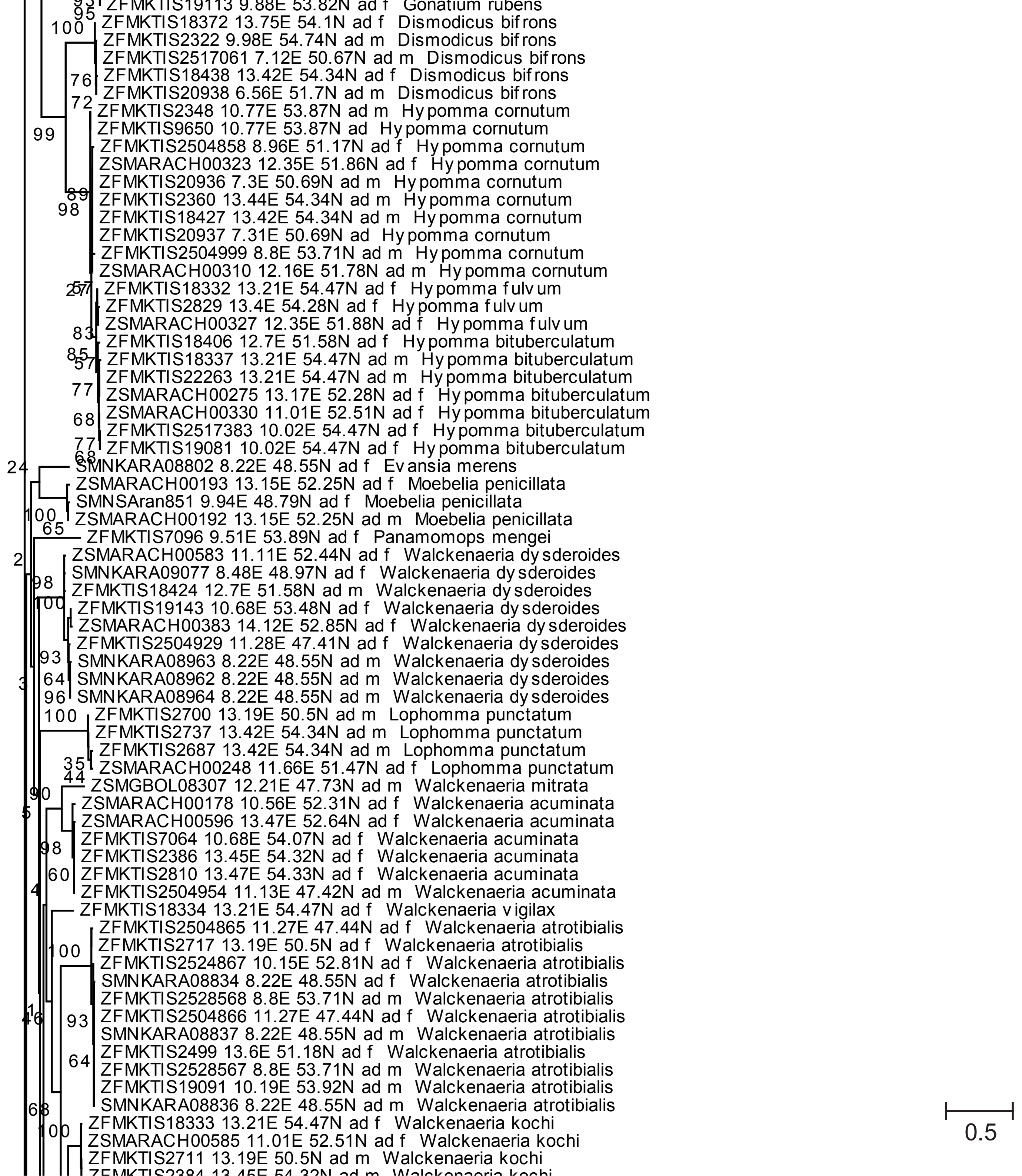
ZFMKTIS19113 9.88E 53.82N ad f Gonatium rubens
ZFMKTIS18372 13.75E 54.1N ad f Dismodicus bifrons
ZFMKTIS2322 9.98E 54.74N ad m Dismodicus bifrons
ZFMKTIS2517061 7.12E 50.67N ad m Dismodicus bifrons
ZFMKTIS18438 13.42E 54.34N ad f Dismodicus bifrons
ZFMKTIS20938 6.56E 51.7N ad m Dismodicus bifrons
ZFMKTIS2348 10.77E 53.87N ad m Hypomma cornutum
ZFMKTIS9650 10.77E 53.87N ad Hypomma cornutum
ZFMKTIS2504858 8.96E 51.17N ad f Hypomma cornutum
ZSMARACH00323 12.35E 51.86N ad f Hypomma cornutum
ZFMKTIS20936 7.3E 50.69N ad m Hypomma cornutum
ZFMKTIS2360 13.44E 54.34N ad m Hypomma cornutum
ZFMKTIS18427 13.42E 54.34N ad m Hypomma cornutum
ZFMKTIS20937 7.31E 50.69N ad Hypomma cornutum
ZFMKTIS2504999 8.8E 53.71N ad m Hypomma cornutum
ZSMARACH00310 12.16E 51.78N ad m Hypomma cornutum
ZFMKTIS18332 13.21E 54.47N ad f Hypomma fulvum
ZFMKTIS2829 13.4E 54.28N ad f Hypomma fulvum
ZSMARACH00327 12.35E 51.88N ad f Hypomma fulvum
ZFMKTIS18406 12.7E 51.58N ad f Hypomma bituberculatum
ZFMKTIS18337 13.21E 54.47N ad m Hypomma bituberculatum
ZFMKTIS22263 13.21E 54.47N ad m Hypomma bituberculatum
ZSMARACH00275 13.17E 52.28N ad f Hypomma bituberculatum
ZSMARACH00330 11.01E 52.51N ad f Hypomma bituberculatum
ZFMKTIS2517383 10.02E 54.47N ad f Hypomma bituberculatum
ZFMKTIS19081 10.02E 54.47N ad f Hypomma bituberculatum
SMNKARA08802 8.22E 48.55N ad f Evansia merens
ZSMARACH00193 13.15E 52.25N ad f Moebelia penicillata
SMNSAran851 9.94E 48.79N ad f Moebelia penicillata
ZSMARACH00192 13.15E 52.25N ad m Moebelia penicillata
ZFMKTIS7096 9.51E 53.89N ad f Panamomops mengei
ZSMARACH00583 11.11E 52.44N ad f Walckenaeria dysderoides
SMNKARA09077 8.48E 48.97N ad f Walckenaeria dysderoides
ZFMKTIS18424 12.7E 51.58N ad m Walckenaeria dysderoides
ZFMKTIS19143 10.68E 53.48N ad f Walckenaeria dysderoides
ZSMARACH00383 14.12E 52.85N ad f Walckenaeria dysderoides
ZFMKTIS2504929 11.28E 47.41N ad f Walckenaeria dysderoides
SMNKARA08963 8.22E 48.55N ad m Walckenaeria dysderoides
SMNKARA08962 8.22E 48.55N ad m Walckenaeria dysderoides
SMNKARA08964 8.22E 48.55N ad m Walckenaeria dysderoides
ZFMKTIS2700 13.19E 50.5N ad m Lophomma punctatum
ZFMKTIS2737 13.42E 54.34N ad m Lophomma punctatum
ZFMKTIS2687 13.42E 54.34N ad m Lophomma punctatum
ZSMARACH00248 11.66E 51.47N ad f Lophomma punctatum
ZSMGBOL08307 12.21E 47.73N ad m Walckenaeria mitrata
ZSMARACH00178 10.56E 52.31N ad f Walckenaeria acuminata
ZSMARACH00596 13.47E 52.64N ad f Walckenaeria acuminata
ZFMKTIS7064 10.68E 54.07N ad f Walckenaeria acuminata
ZFMKTIS2386 13.45E 54.32N ad f Walckenaeria acuminata
ZFMKTIS2810 13.47E 54.33N ad f Walckenaeria acuminata
ZFMKTIS2504954 11.13E 47.42N ad m Walckenaeria acuminata
ZFMKTIS18334 13.21E 54.47N ad f Walckenaeria vigilax
ZFMKTIS2504865 11.27E 47.44N ad f Walckenaeria atrotibialis
ZFMKTIS2717 13.19E 50.5N ad f Walckenaeria atrotibialis
ZFMKTIS2524867 10.15E 52.81N ad f Walckenaeria atrotibialis
SMNKARA08834 8.22E 48.55N ad f Walckenaeria atrotibialis
ZFMKTIS2528568 8.8E 53.71N ad m Walckenaeria atrotibialis
ZFMKTIS2504866 11.27E 47.44N ad f Walckenaeria atrotibialis
SMNKARA08837 8.22E 48.55N ad m Walckenaeria atrotibialis
ZFMKTIS2499 13.6E 51.18N ad f Walckenaeria atrotibialis
ZFMKTIS2528567 8.8E 53.71N ad m Walckenaeria atrotibialis
ZFMKTIS19091 10.19E 53.92N ad m Walckenaeria atrotibialis
SMNKARA08836 8.22E 48.55N ad m Walckenaeria atrotibialis
ZFMKTIS18333 13.21E 54.47N ad f Walckenaeria kochi
ZSMARACH00585 11.01E 52.51N ad f Walckenaeria kochi
ZFMKTIS2711 13.19E 50.5N ad m Walckenaeria kochi
0.5

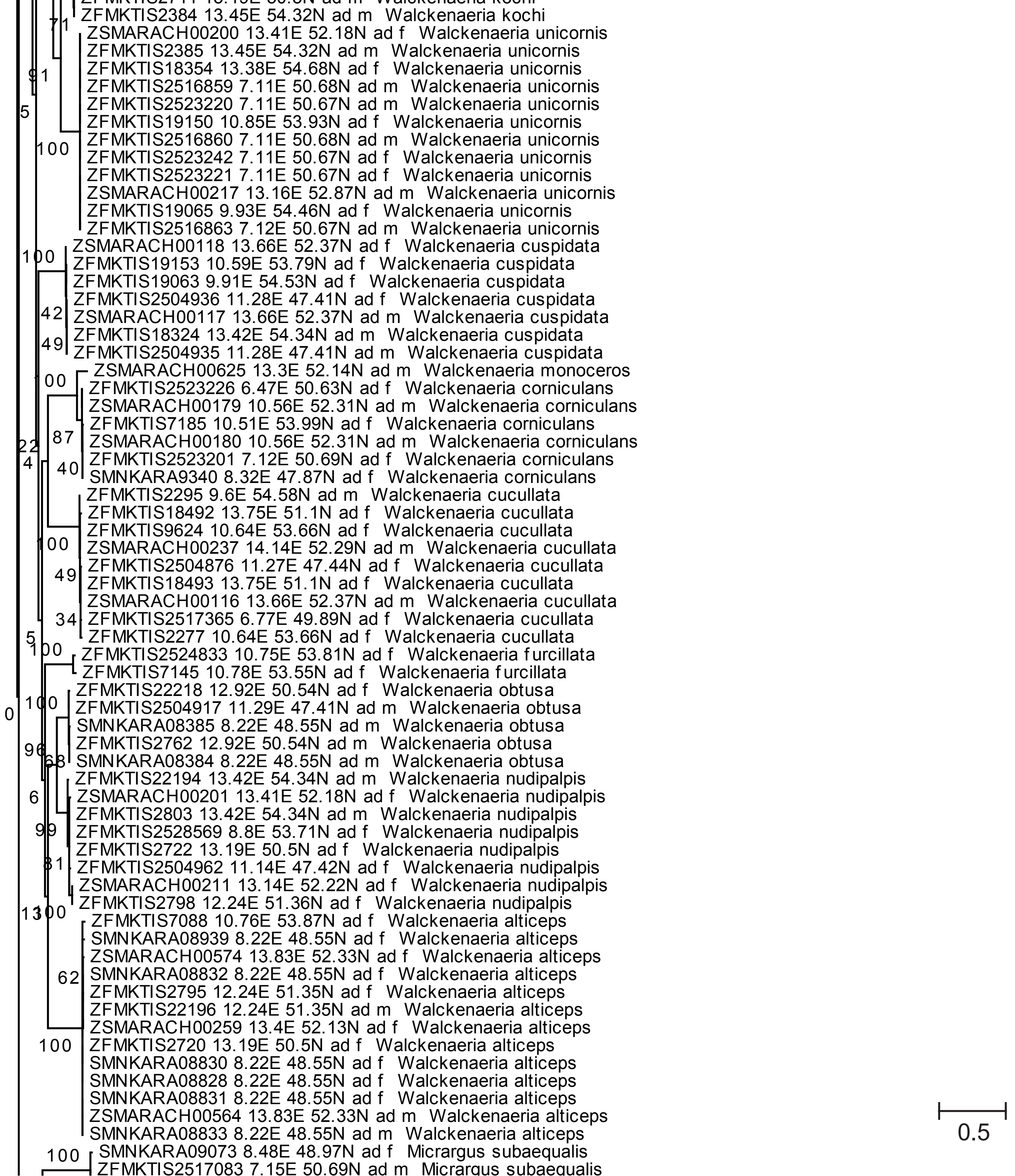

ZFMKTIS2384 13.45E 54.32N ad m Walckenaeria kochi
ZSMARACH00200 13.41E 52.18N ad f Walckenaeria unicornis
ZFMKTIS2385 13.45E 54.32N ad m Walckenaeria unicornis
ZFMKTIS18354 13.38E 54.68N ad f Walckenaeria unicornis
ZFMKTIS2516859 7.11E 50.68N ad m Walckenaeria unicornis
ZFMKTIS2523220 7.11E 50.67N ad m Walckenaeria unicornis
ZFMKTIS19150 10.85E 53.93N ad f Walckenaeria unicornis
ZFMKTIS2516860 7.11E 50.68N ad m Walckenaeria unicornis
ZFMKTIS2523242 7.11E 50.67N ad f Walckenaeria unicornis
ZFMKTIS2523221 7.11E 50.67N ad f Walckenaeria unicornis
ZSMARACH00217 13.16E 52.87N ad m Walckenaeria unicornis
ZFMKTIS19065 9.93E 54.46N ad f Walckenaeria unicornis
ZFMKTIS2516863 7.12E 50.67N ad m Walckenaeria unicornis
ZSMARACH00118 13.66E 52.37N ad f Walckenaeria cuspidata
ZFMKTIS19153 10.59E 53.79N ad f Walckenaeria cuspidata
ZFMKTIS19063 9.91E 54.53N ad f Walckenaeria cuspidata
ZFMKTIS2504936 11.28E 47.41N ad f Walckenaeria cuspidata
ZSMARACH00117 13.66E 52.37N ad m Walckenaeria cuspidata
ZFMKTIS18324 13.42E 54.34N ad m Walckenaeria cuspidata
ZFMKTIS2504935 11.28E 47.41N ad m Walckenaeria cuspidata
ZSMARACH00625 13.3E 52.14N ad m Walckenaeria monoceros
ZFMKTIS2523226 6.47E 50.63N ad f Walckenaeria corniculans
ZSMARACH00179 10.56E 52.31N ad m Walckenaeria corniculans
ZFMKTIS7185 10.51E 53.99N ad f Walckenaeria corniculans
ZSMARACH00180 10.56E 52.31N ad m Walckenaeria corniculans
ZFMKTIS2523201 7.12E 50.69N ad f Walckenaeria corniculans
SMNKARA9340 8.32E 47.87N ad f Walckenaeria corniculans
ZFMKTIS2295 9.6E 54.58N ad m Walckenaeria cucullata
ZFMKTIS18492 13.75E 51.1N ad f Walckenaeria cucullata
ZFMKTIS9624 10.64E 53.66N ad f Walckenaeria cucullata
ZSMARACH00237 14.14E 52.29N ad m Walckenaeria cucullata
ZFMKTIS2504876 11.27E 47.44N ad f Walckenaeria cucullata
ZFMKTIS18493 13.75E 51.1N ad f Walckenaeria cucullata
ZSMARACH00116 13.66E 52.37N ad m Walckenaeria cucullata
ZFMKTIS2517365 6.77E 49.89N ad f Walckenaeria cucullata
ZFMKTIS2277 10.64E 53.66N ad f Walckenaeria cucullata
ZFMKTIS2524833 10.75E 53.81N ad f Walckenaeria furcillata
ZFMKTIS7145 10.78E 53.55N ad f Walckenaeria furcillata
ZFMKTIS22218 12.92E 50.54N ad f Walckenaeria obtusa
ZFMKTIS2504917 11.29E 47.41N ad m Walckenaeria obtusa
SMNKARA08385 8.22E 48.55N ad m Walckenaeria obtusa
ZFMKTIS2762 12.92E 50.54N ad m Walckenaeria obtusa
SMNKARA08384 8.22E 48.55N ad m Walckenaeria obtusa
ZFMKTIS22194 13.42E 54.34N ad m Walckenaeria nudipalpis
ZSMARACH00201 13.41E 52.18N ad f Walckenaeria nudipalpis
ZFMKTIS2803 13.42E 54.34N ad m Walckenaeria nudipalpis
ZFMKTIS2528569 8.8E 53.71N ad f Walckenaeria nudipalpis
ZFMKTIS2722 13.19E 50.5N ad f Walckenaeria nudipalpis
ZFMKTIS2504962 11.14E 47.42N ad f Walckenaeria nudipalpis
ZSMARACH00211 13.14E 52.22N ad f Walckenaeria nudipalpis
ZFMKTIS2798 12.24E 51.36N ad f Walckenaeria nudipalpis
ZFMKTIS7088 10.76E 53.87N ad f Walckenaeria alticeps
SMNKARA08939 8.22E 48.55N ad f Walckenaeria alticeps
ZSMARACH00574 13.83E 52.33N ad f Walckenaeria alticeps
SMNKARA08832 8.22E 48.55N ad f Walckenaeria alticeps
ZFMKTIS2795 12.24E 51.35N ad f Walckenaeria alticeps
ZFMKTIS22196 12.24E 51.35N ad m Walckenaeria alticeps
ZSMARACH00259 13.4E 52.13N ad f Walckenaeria alticeps
ZFMKTIS2720 13.19E 50.5N ad f Walckenaeria alticeps
SMNKARA08830 8.22E 48.55N ad f Walckenaeria alticeps
SMNKARA08828 8.22E 48.55N ad f Walckenaeria alticeps
SMNKARA08831 8.22E 48.55N ad f Walckenaeria alticeps
ZSMARACH00564 13.83E 52.33N ad m Walckenaeria alticeps
SMNKARA08833 8.22E 48.55N ad m Walckenaeria alticeps
SMNKARA09073 8.48E 48.97N ad f Micrargus subaequalis
0.5

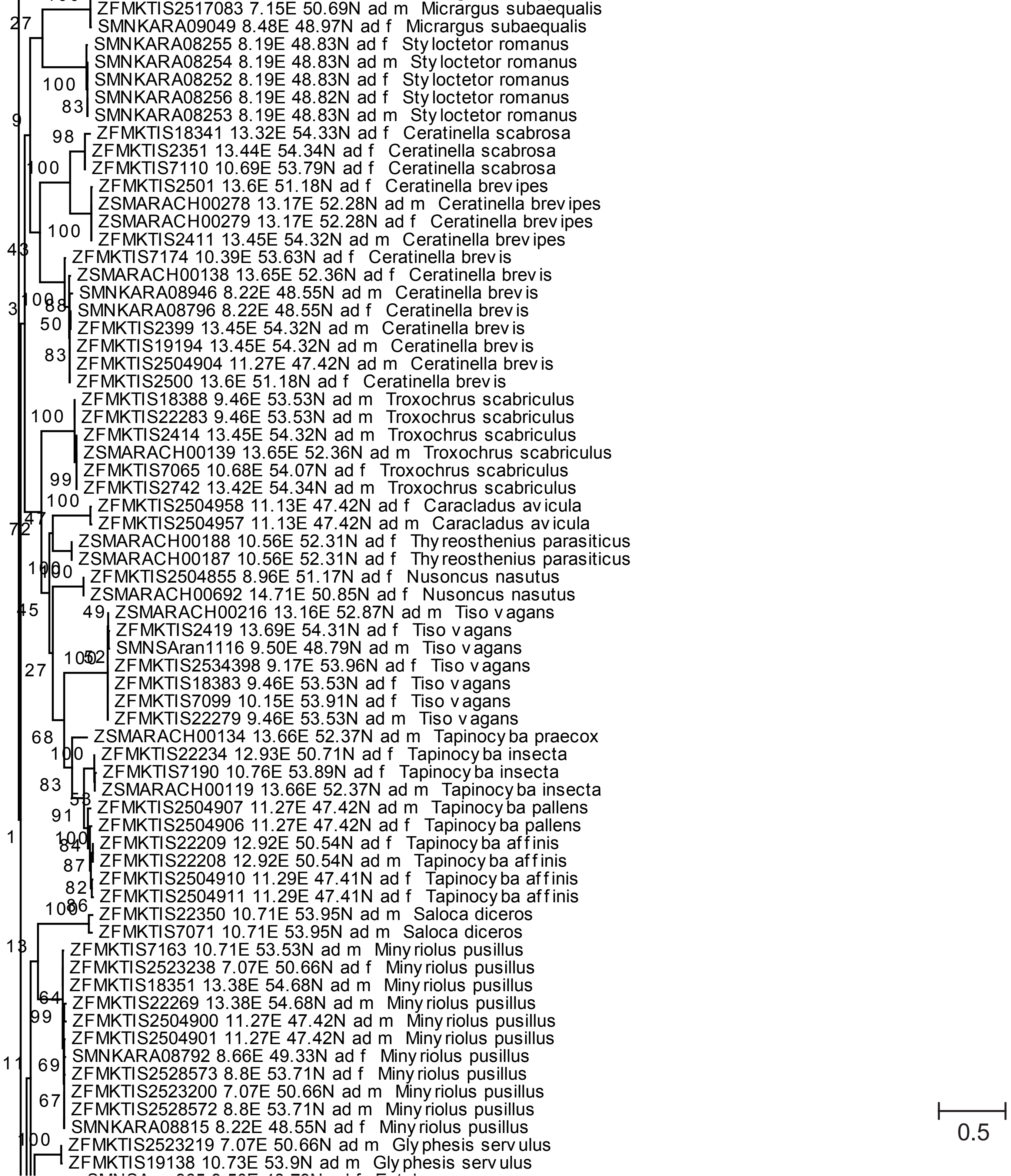
ZFMKTIS2517083 7.15E 50.69N ad m Micrargus subaequalis
SMNKARA09049 8.48E 48.97N ad f Micrargus subaequalis
SMNKARA08255 8.19E 48.83N ad f Styloctetor romanus
SMNKARA08254 8.19E 48.83N ad m Styloctetor romanus
SMNKARA08252 8.19E 48.83N ad f Styloctetor romanus
SMNKARA08256 8.19E 48.82N ad f Styloctetor romanus
SMNKARA08253 8.19E 48.83N ad m Styloctetor romanus
ZFMKTIS18341 13.32E 54.33N ad f Ceratinella scabrosa
ZFMKTIS2351 13.44E 54.34N ad f Ceratinella scabrosa
ZFMKTIS7110 10.69E 53.79N ad f Ceratinella scabrosa
ZFMKTIS2501 13.6E 51.18N ad f Ceratinella brevipes
ZSMARACH00278 13.17E 52.28N ad m Ceratinella brevipes
ZSMARACH00279 13.17E 52.28N ad f Ceratinella brevipes
ZFMKTIS2411 13.45E 54.32N ad m Ceratinella brevipes
ZFMKTIS7174 10.39E 53.63N ad f Ceratinella brevis
ZSMARACH00138 13.65E 52.36N ad f Ceratinella brevis
SMNKARA08946 8.22E 48.55N ad m Ceratinella brevis
SMNKARA08796 8.22E 48.55N ad f Ceratinella brevis
ZFMKTIS2399 13.45E 54.32N ad m Ceratinella brevis
ZFMKTIS19194 13.45E 54.32N ad m Ceratinella brevis
ZFMKTIS2504904 11.27E 47.42N ad m Ceratinella brevis
ZFMKTIS2500 13.6E 51.18N ad f Ceratinella brevis
ZFMKTIS18388 9.46E 53.53N ad m Troxochrus scabriculus
ZFMKTIS22283 9.46E 53.53N ad m Troxochrus scabriculus
ZFMKTIS2414 13.45E 54.32N ad m Troxochrus scabriculus
ZSMARACH00139 13.65E 52.36N ad m Troxochrus scabriculus
ZFMKTIS7065 10.68E 54.07N ad f Troxochrus scabriculus
ZFMKTIS2742 13.42E 54.34N ad m Troxochrus scabriculus
ZFMKTIS2504958 11.13E 47.42N ad f Caracladus avicula
ZFMKTIS2504957 11.13E 47.42N ad m Caracladus avicula
ZSMARACH00188 10.56E 52.31N ad f Thyreosthenius parasiticus
ZSMARACH00187 10.56E 52.31N ad f Thyreosthenius parasiticus
ZFMKTIS2504855 8.96E 51.17N ad f Nusoncus nasutus
ZSMARACH00692 14.71E 50.85N ad f Nusoncus nasutus
ZSMARACH00216 13.16E 52.87N ad m Tiso vagans
ZFMKTIS2419 13.69E 54.31N ad f Tiso vagans
SMNSAran1116 9.50E 48.79N ad m Tiso vagans
ZFMKTIS2534398 9.17E 53.96N ad f Tiso vagans
ZFMKTIS18383 9.46E 53.53N ad f Tiso vagans
ZFMKTIS7099 10.15E 53.91N ad f Tiso vagans
ZFMKTIS22279 9.46E 53.53N ad m Tiso vagans
ZSMARACH00134 13.66E 52.37N ad m Tapinocyba praecox
ZFMKTIS22234 12.93E 50.71N ad f Tapinocyba insecta
ZFMKTIS7190 10.76E 53.89N ad f Tapinocyba insecta
ZSMARACH00119 13.66E 52.37N ad m Tapinocyba insecta
ZFMKTIS2504907 11.27E 47.42N ad m Tapinocyba pallens
ZFMKTIS2504906 11.27E 47.42N ad f Tapinocyba pallens
ZFMKTIS22209 12.92E 50.54N ad f Tapinocyba affinis
ZFMKTIS22208 12.92E 50.54N ad m Tapinocyba affinis
ZFMKTIS2504910 11.29E 47.41N ad f Tapinocyba affinis
ZFMKTIS2504911 11.29E 47.41N ad f Tapinocyba affinis
ZFMKTIS22350 10.71E 53.95N ad m Saloca diceros
ZFMKTIS7071 10.71E 53.95N ad m Saloca diceros
ZFMKTIS7163 10.71E 53.53N ad m Minyriolus pusillus
ZFMKTIS2523238 7.07E 50.66N ad f Minyriolus pusillus
ZFMKTIS18351 13.38E 54.68N ad m Minyriolus pusillus
ZFMKTIS22269 13.38E 54.68N ad m Minyriolus pusillus
ZFMKTIS2504900 11.27E 47.42N ad m Minyriolus pusillus
ZFMKTIS2504901 11.27E 47.42N ad m Minyriolus pusillus
SMNKARA08792 8.66E 49.33N ad f Minyriolus pusillus
ZFMKTIS2528573 8.8E 53.71N ad f Minyriolus pusillus
ZFMKTIS2523200 7.07E 50.66N ad m Minyriolus pusillus
ZFMKTIS2528572 8.8E 53.71N ad m Minyriolus pusillus
SMNKARA08815 8.22E 48.55N ad f Minyriolus pusillus
ZFMKTIS2523219 7.07E 50.66N ad m Glyphesis servulus
ZFMKTIS19138 10.73E 53.9N ad m Glyphesis servulus
0.5

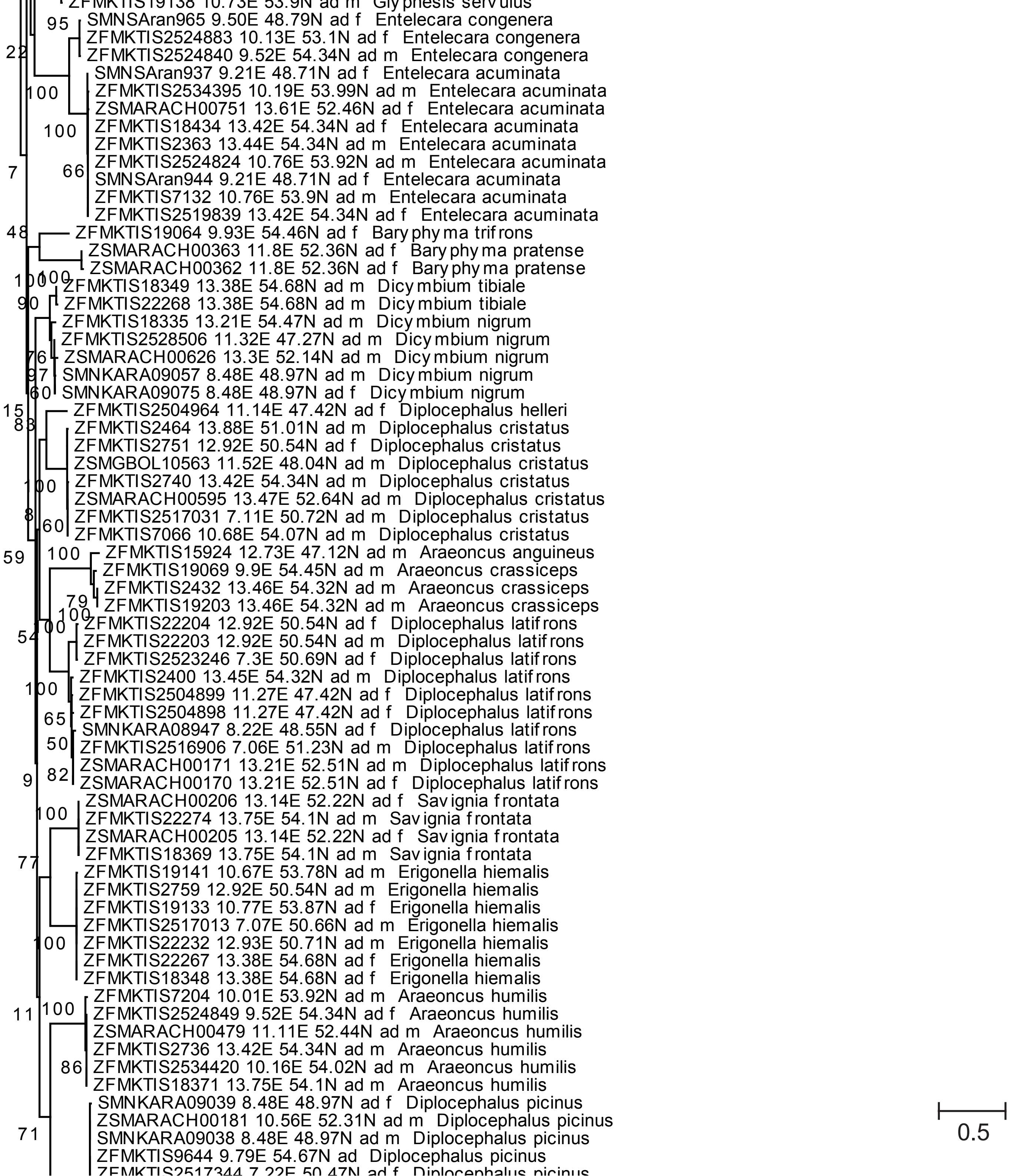
ZFMKTIS19138 10.73E 53.9N ad m Glyphesis servulus
SMNSAran965 9.50E 48.79N ad f Entelecara congenera
ZFMKTIS2524883 10.13E 53.1N ad f Entelecara congenera
ZFMKTIS2524840 9.52E 54.34N ad m Entelecara congenera
SMNSAran937 9.21E 48.71N ad f Entelecara acuminata
ZFMKTIS2534395 10.19E 53.99N ad m Entelecara acuminata
ZSMARACH00751 13.61E 52.46N ad f Entelecara acuminata
ZFMKTIS18434 13.42E 54.34N ad f Entelecara acuminata
ZFMKTIS2363 13.44E 54.34N ad m Entelecara acuminata
ZFMKTIS2524824 10.76E 53.92N ad m Entelecara acuminata
SMNSAran944 9.21E 48.71N ad f Entelecara acuminata
ZFMKTIS7132 10.76E 53.9N ad m Entelecara acuminata
ZFMKTIS2519839 13.42E 54.34N ad f Entelecara acuminata
ZFMKTIS19064 9.93E 54.46N ad f Baryphyma trifrons
ZSMARACH00363 11.8E 52.36N ad f Baryphyma pratense
ZSMARACH00362 11.8E 52.36N ad f Baryphyma pratense
ZFMKTIS18349 13.38E 54.68N ad m Dicymbium tibiale
ZFMKTIS22268 13.38E 54.68N ad m Dicymbium tibiale
ZFMKTIS18335 13.21E 54.47N ad m Dicymbium nigrum
ZFMKTIS2528506 11.32E 47.27N ad m Dicymbium nigrum
ZSMARACH00626 13.3E 52.14N ad m Dicymbium nigrum
SMNKARA09057 8.48E 48.97N ad m Dicymbium nigrum
SMNKARA09075 8.48E 48.97N ad f Dicymbium nigrum
ZFMKTIS2504964 11.14E 47.42N ad f Diplocephalus helleri
ZFMKTIS2464 13.88E 51.01N ad m Diplocephalus cristatus
ZFMKTIS2751 12.92E 50.54N ad f Diplocephalus cristatus
ZSMGBOL10563 11.52E 48.04N ad m Diplocephalus cristatus
ZFMKTIS2740 13.42E 54.34N ad m Diplocephalus cristatus
ZSMARACH00595 13.47E 52.64N ad m Diplocephalus cristatus
ZFMKTIS2517031 7.11E 50.72N ad m Diplocephalus cristatus
ZFMKTIS7066 10.68E 54.07N ad m Diplocephalus cristatus
ZFMKTIS15924 12.73E 47.12N ad m Araeoncus anguineus
ZFMKTIS19069 9.9E 54.45N ad m Araeoncus crassiceps
ZFMKTIS2432 13.46E 54.32N ad m Araeoncus crassiceps
ZFMKTIS19203 13.46E 54.32N ad m Araeoncus crassiceps
ZFMKTIS22204 12.92E 50.54N ad f Diplocephalus latifrons
ZFMKTIS22203 12.92E 50.54N ad m Diplocephalus latifrons
ZFMKTIS2523246 7.3E 50.69N ad f Diplocephalus latifrons
ZFMKTIS2400 13.45E 54.32N ad m Diplocephalus latifrons
ZFMKTIS2504899 11.27E 47.42N ad f Diplocephalus latifrons
ZFMKTIS2504898 11.27E 47.42N ad f Diplocephalus latifrons
SMNKARA08947 8.22E 48.55N ad f Diplocephalus latifrons
ZFMKTIS2516906 7.06E 51.23N ad m Diplocephalus latifrons
ZSMARACH00171 13.21E 52.51N ad m Diplocephalus latifrons
ZSMARACH00170 13.21E 52.51N ad f Diplocephalus latifrons
ZSMARACH00206 13.14E 52.22N ad f Savignia frontata
ZFMKTIS22274 13.75E 54.1N ad m Savignia frontata
ZSMARACH00205 13.14E 52.22N ad f Savignia frontata
ZFMKTIS18369 13.75E 54.1N ad m Savignia frontata
ZFMKTIS19141 10.67E 53.78N ad m Erigonella hiemalis
ZFMKTIS2759 12.92E 50.54N ad m Erigonella hiemalis
ZFMKTIS19133 10.77E 53.87N ad f Erigonella hiemalis
ZFMKTIS2517013 7.07E 50.66N ad m Erigonella hiemalis
ZFMKTIS22232 12.93E 50.71N ad m Erigonella hiemalis
ZFMKTIS22267 13.38E 54.68N ad f Erigonella hiemalis
ZFMKTIS18348 13.38E 54.68N ad f Erigonella hiemalis
ZFMKTIS7204 10.01E 53.92N ad m Araeoncus humilis
ZFMKTIS2524849 9.52E 54.34N ad f Araeoncus humilis
ZSMARACH00479 11.11E 52.44N ad m Araeoncus humilis
ZFMKTIS2736 13.42E 54.34N ad m Araeoncus humilis
ZFMKTIS2534420 10.16E 54.02N ad m Araeoncus humilis
ZFMKTIS18371 13.75E 54.1N ad m Araeoncus humilis
SMNKARA09039 8.48E 48.97N ad f Diplocephalus picinus
ZSMARACH00181 10.56E 52.31N ad m Diplocephalus picinus
SMNKARA09038 8.48E 48.97N ad m Diplocephalus picinus
ZFMKTIS9644 9.79E 54.67N ad Diplocephalus picinus
ZFMKTIS2517344 7.22E 50.47N ad f Diplocephalus picinus
0.5

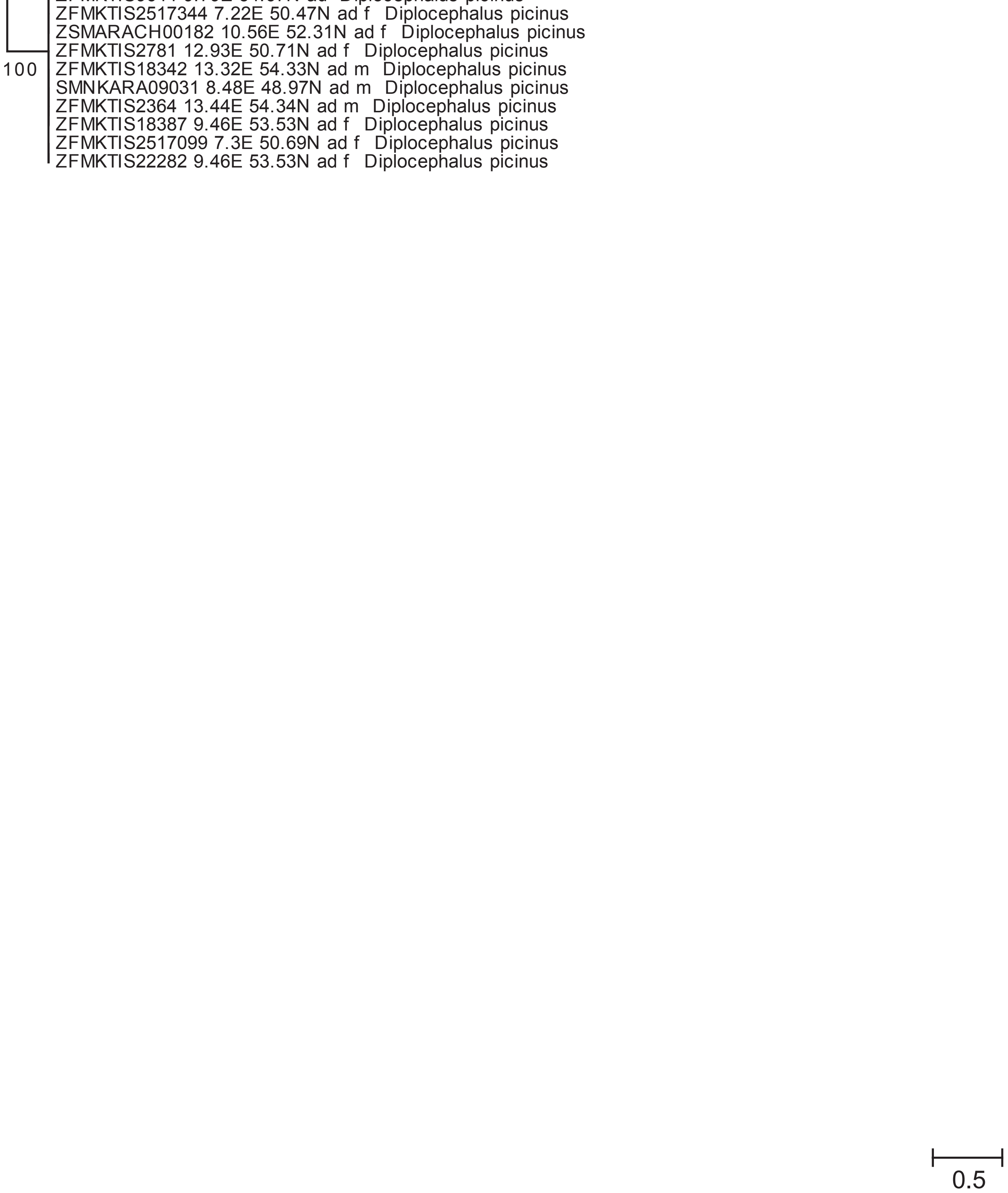

ZFMKTIS2517344 7.22E 50.47N ad f Diplocephalus picinus
ZSMARACH00182 10.56E 52.31N ad f Diplocephalus picinus
ZFMKTIS2781 12.93E 50.71N ad f Diplocephalus picinus
100
ZFMKTIS18342 13.32E 54.33N ad m Diplocephalus picinus
SMNKARA09031 8.48E 48.97N ad m Diplocephalus picinus
ZFMKTIS2364 13.44E 54.34N ad m Diplocephalus picinus
ZFMKTIS18387 9.46E 53.53N ad f Diplocephalus picinus
ZFMKTIS2517099 7.3E 50.69N ad f Diplocephalus picinus
ZFMKTIS22282 9.46E 53.53N ad f Diplocephalus picinus
0.5
