# Supplementary figures and images for: Towards a DNA Barcode Reference Database for Spiders and Harvestmen of Germany
Source: PLoS One. 2016 Sep 28;11(9):e0162624. doi: 10.1371/journal.pone.0162624 (PMC5040438; doi:10.1371/journal.pone.0162624)

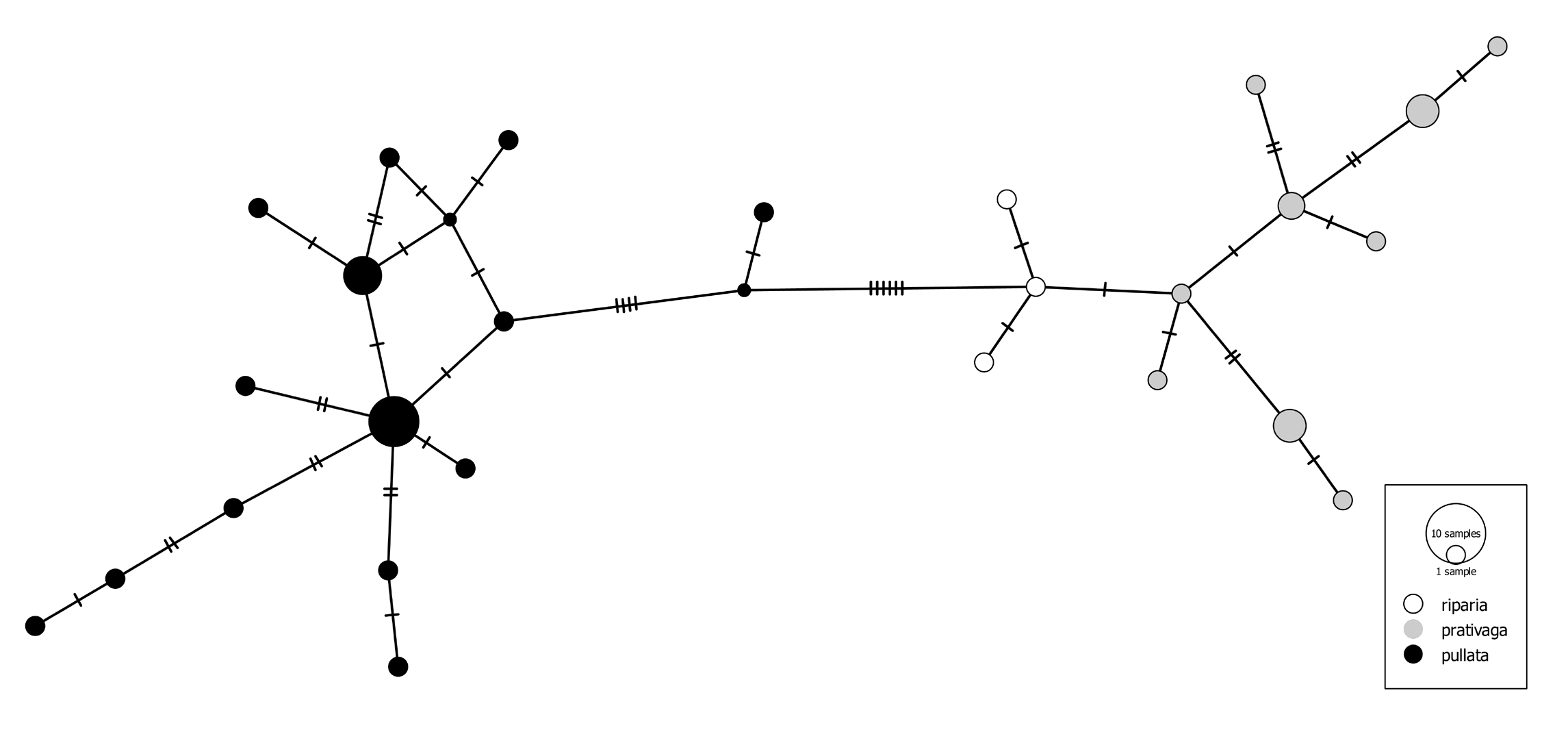

Supplement: S3 Fig — Small black dots indicate hypothetical haplotypes. (TIF) [file pone.0162624.s004.tif]

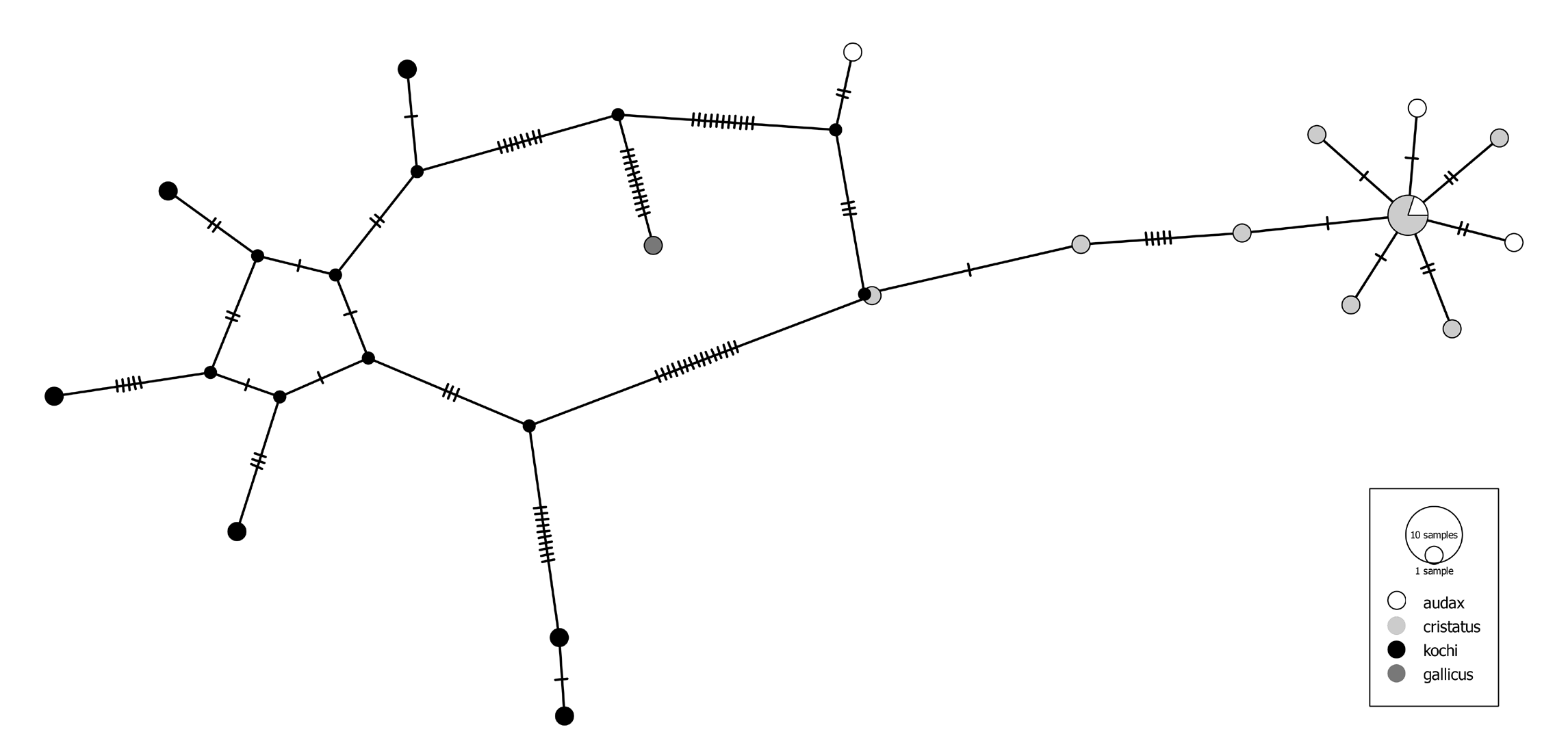

Supplement: S4 Fig — To guarantee unequivocal morphological determination, only males were included. Small black dots indicate hypothetical haplotypes. (TIF) [file pone.0162624.s005.tif]

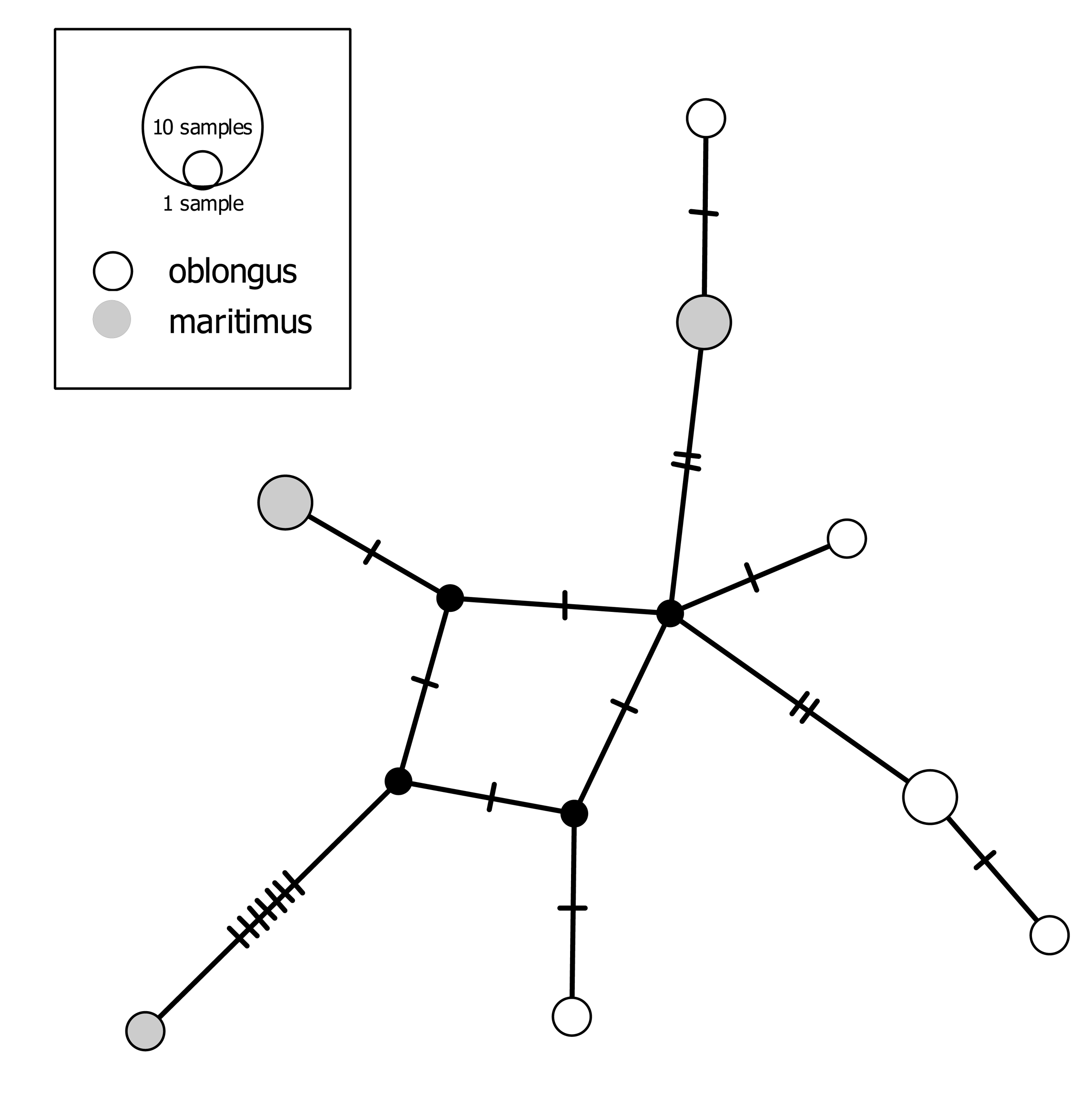

Supplement: S5 Fig — Small black dots indicate hypothetical haplotypes. (TIF) [file pone.0162624.s006.tif]
